# Discovery of a Novel Selective PAK1/HDAC6/HDAC10 Inhibitor ZMF-25 that Induces Mitochondrial Metabolic Breakdown and Autophagy-Related Cell Death in Triple-Negative Breast Cancer

**DOI:** 10.34133/research.0670

**Published:** 2025-04-29

**Authors:** Jin Zhang, Xiaoling Cheng, Gang Chen, Xiya Chen, Xi Zhao, Weiji Chen, Wei Du, Zhendan He, Xiaojun Yao, Bo Han, Dahong Yao

**Affiliations:** ^1^ School of Pharmaceutical Sciences, Health Science Center, Shenzhen University, Shenzhen 518060, China.; ^2^ School of Pharmaceutical Sciences, Shenzhen Technology University, Shenzhen 518118, China.; ^3^ State Key Laboratory of Southwestern Chinese Medicine Resources, Hospital of Chengdu University of Traditional Chinese Medicine, School of Pharmacy, Chengdu University of Traditional Chinese Medicine, Chengdu 611137, China.; ^4^ Centre for Artificial Intelligence Driven Drug Discovery, Faculty of Applied Sciences, Macao Polytechnic University, Macao 999078, China.; ^5^ West China School of Pharmacy, Sichuan University, Chengdu 610000, China.

## Abstract

Triple-negative breast cancer (TNBC) is the most aggressive breast cancer subtype, and addressing its intrinsic heterogeneity has emerged as a valuable avenue for novel clinical treatment strategy. Here, we put forward an innovative strategy for TNBC treatment by simultaneously suppressing both p21-activated kinase 1 (PAK1) and histone deacetylase (HDAC) class IIb (HDAC6/10). A series of pyrido [2,3-d]pyrimidin-7(8*H*)-one moiety derivatives was successfully designed and synthesized to target PAK1/HDAC6/HDAC10 by utilizing structure-based screening and pharmacophore integration. ZMF-25 demonstrates marked inhibitory activity against PAK1, HDAC6, and HDAC10 with respective IC_50_ values of 33, 64, and 41 nM, remarkable selectivity over HDACs and PAKs, as well as prominent antiproliferative efficiency in MDA-MB-231 cells. Additionally, ZMF-25 effectively suppresses TNBC proliferation and migration by inhibiting PAK1/HDAC6/HDAC10. Moreover, it was found to impair glycolysis and trigger reactive oxygen species generation, resulting in autophagy-related cell death by inhibiting the AKT/mTOR/ULK1 signaling. Furthermore, ZMF-25 exhibits remarkable therapeutic potential with no obvious toxicity in vivo and good pharmacokinetics. In summary, these observations indicate that ZMF-25 is a novel and potent triple-targeting PAK1/HDAC6/HDAC10 inhibitor, which is expected to provide a novel and effective strategy for TNBC treatment.

## Introduction

Triple-negative breast cancer (TNBC), a highly aggressive breast cancer subtype, faces treatment challenges due to absent therapeutic targets and no approved targeted therapies, representing a critical unmet medical need [1]. Emerging evidence links TNBC’s heterogeneity in energy metabolism, epigenetic modifications, and microenvironment to limited therapeutic efficacy across endocrine, chemo-, immuno-, and targeted therapies [2–5]. Shao and colleagues [6] identified 3 TNBC metabolic subtypes via multi-omics analysis: lipogenic (elevated lipid synthesis), glycolytic (enhanced carbohydrate and nucleotide metabolism), and mixed dysregulation. TNBC’s metabolic reprogramming drives aerobic glycolysis (Warburg effect) for proliferation and stress survival despite oxygen availability, increasing sensitivity to glycolytic inhibitors [7,8]. Additionally, the distinct tumor microenvironment of TNBC drives proliferation, angiogenesis, apoptosis resistance, immune suppression, and drug resistance, fueling progression and metastasis [9,10]. Dysregulated epigenetic mechanisms critically drive TNBC progression through key oncogenic processes including proliferation, immune evasion, metastasis, and drug resistance [11,12]. Accumulating translational evidence highlights the therapeutic potential of epigenetic-targeted approaches in clinical oncology [13,14]. Meanwhile, the development of therapeutic strategies specific to the metabolic characteristics of cancer is also in line with the contemporary development of precision therapy [15]. In addition, autophagy has been a research focus in cancer [16], and autophagy-associated cell death is also a potentially emerging important therapeutic strategy for TNBC treatment. Collectively, the targeted disruption of intricate signaling networks by modulating mitochondrial metabolism and autophagy to address the heterogeneity holds great promise as a strategy for developing innovative therapies that specifically target TNBC.

PAK1 represents a strategic therapeutic target in oncology, given its central role in governing tumorigenesis, progression, angiogenesis, metastasis, therapy resistance, and metabolic reprogramming [17,18]. The acetylation of PAK1, which is reliant on ELP3, facilitates the initiation of autophagy under hypoxic conditions and sustains brain tumorigenesis [19]. PAK1 convergently regulates oncogenic signaling hubs, as evidenced by its inhibition inducing Ras-driven tumor regression and AKT suppression in vivo, validating its mechanistic role in KRAS-dependent phosphatidylinositol 3-kinase (PI3K)/AKT activation [20]. In recent years, there has been a notable emergence of numerous highly effective inhibitors targeting PAK1, including **1** (Roche) [21], **2** (FP-3758309, Pfizer) [22], **3** (FRAX597, Afraxis) [23], and **4** (AZ-13711265, AstraZeneca) [24]. In addition, we have also meticulously designed and synthesized a multitude of potent PAK1 inhibitors in the early stages, which have exhibited remarkable potential in the treatment of TNBC through their adept regulation of energy metabolism and the tumor microenvironment [25–27]. These adenosine triphosphate (ATP)-competitive scaffolds employ conserved aminopyrimidine moieties as adenine-mimetic anchors, forming critical hinge-region hydrogen bonds in PAK1’s catalytic pocket. While demonstrating potent PAK1 inhibition, most derivatives exhibit suboptimal cellular penetration, target promiscuity, and pharmacokinetic (PK) challenges that compromise therapeutic utility [28,29].

Histone deacetylases (HDACs), chromatin-modifying enzymes regulating gene expression via histone tail lysine deacetylation, additionally target nonhistone substrates to expand epigenetic control [30,31]. HDACs are classified into 4 groups: I (HDAC1 to HDAC3 and HDAC8), IIa (HDAC4, HDAC5, HDAC7, and HDAC9), IIb (HDAC6 and HDAC10), III (Sirt1 to Sirt7), and IV (HDAC11) [32]. Clinically approved HDAC inhibitors demonstrate therapeutic efficacy in oncology, yet existing pan-HDAC inhibitors’ nonselective activity across subtypes correlates with dose-limiting off-target toxicities [33,34]. Recently, the development of subtype-specific HDAC inhibitors has emerged as a new strategy in pharmaceutical research. Class IIb HDACs exhibit unique cytoplasmic localization and minimal HDAC activity compared to nuclear class I isoforms (HDAC1 to HDAC3 and HDAC8), primarily regulating acetylation homeostasis via nonhistone substrates. HDAC6 uniquely harbors dual catalytic domains and a ZnF-UBP domain, mediating ubiquitin–proteasome interactions and aggresome formation to enable misfolded protein clearance [35]. In addition, HDAC6 can target several key nonhistone substrates, including α-tubulin, SHP, HSP90, HSF1, Runx2, Smad7, tau, cortactin, and peroxiredoxin [34]. Due to its distinctive structure and wide range of substrates, HDAC6 plays a crucial role in multiple cellular pathways in cancer, including autophagy, apoptosis, metastasis, and drug resistance [36]. HDAC10 specifically hydrolyzes N^8^-acetylspermidine (N^8^-AcSpd) without affecting histone acetylation levels [37]. HDAC10 targets identified substrates including HSP90 and LcoR. Emerging evidence indicates that N^8^-AcSpd serves as a metabolic reservoir for proliferating cells with heightened polyamine demand—a hallmark of neoplastic proliferation [37,38]. HDAC10 critically regulates key cancer processes including proliferation, apoptosis, metastasis, angiogenesis, autophagy, DNA repair, drug resistance, and epigenetic regulation [39,40]. A study demonstrated that the expression of HDAC10 was significantly and positively correlated with the expression of PD-L1 in tumor cells, suggesting that HDAC10 may be involved in tumor immunity [41]. HDAC6/10 exhibit functional synergy in tumor signaling despite physiological divergence. Dual inhibition may demonstrate therapeutic potential against metastatic malignancies, particularly TNBC. In recent years, several selective inhibitors targeting HDAC6 or HDAC10 have been developed, including **5** (tubastatin A) [42], **6** (CAY10603) [42], **7** (DKFZ-748) [43], and **8** [44] (Fig. S1). Current HDAC inhibitors predominantly employ hydroxamic acid-based zinc chelation mechanisms. While clinically effective in hematological malignancies, their therapeutic limitations in solid tumors motivate our proposal: Pharmacokinetically optimized dual PAK1/HDAC IIb inhibitors may overcome TNBC heterogeneity through multi-mechanistic modulation of energy metabolism pathways, PAK1/HDAC10-mediated tumor microenvironment dynamics, and their functional cross-talk.

In this study, our objective was to develop potent inhibitors that can effectively target both PAK1 and HDAC IIb for the treatment of TNBC. We successfully designed and synthesized a series of derivatives containing pyrido[2,3-d] pyrimidin-7(8*H*)-one-coupled hydroxamic acid by utilizing virtual screening and pharmacophore fusion. Among these compounds, **32c** (named ZMF-25) emerged as an extremely promising inhibitor with exceptional potency against PAK1 (IC_50_ = 33 nM), HDAC6 (IC_50_ = 64 nM), and HDAC10 (IC_50_ = 41 nM). It also exhibited significant antiproliferative potency with an IC_50_ value of 0.76 μM while maintaining good subtype selectivity of PAKs and HDACs in MDA-MB-231 cells. We conducted surface plasmon resonance (SPR) and cellular thermal shift assay (CETSA) to confirm its targeted binding with PAK1 and HDAC IIb. Molecular docking and kinetic simulations further provided insights into its potential binding mode and structural basis of subtype selectivity. In addition to inhibiting PAK1/HDAC6/10 to suppress proliferation and migration, ZMF-25 induces mitochondrial damage and mitochondrial metabolic breakdown by promoting reactive oxygen species (ROS) generation in MDA-MB-231 cells. Furthermore, ZMF-25 enhances autophagy by inhibiting the AKT/mTOR/ULK1 signaling and exhibits therapeutic potential through the inhibition of PAK1/HDAC6/10. Notably, it demonstrates no significant toxicity in vivo and possesses favorable PK properties. Overall, these findings highlight ZMF-25 as an innovative dual inhibitor targeting both PAK1/HDAC6/10 that holds great promise for addressing the heterogeneity associated with TNBC.

## Results

### Triple inhibition of PAK1/HDAC6/10 demonstrates novel promising potential in breast cancer

**Fig. 1.**
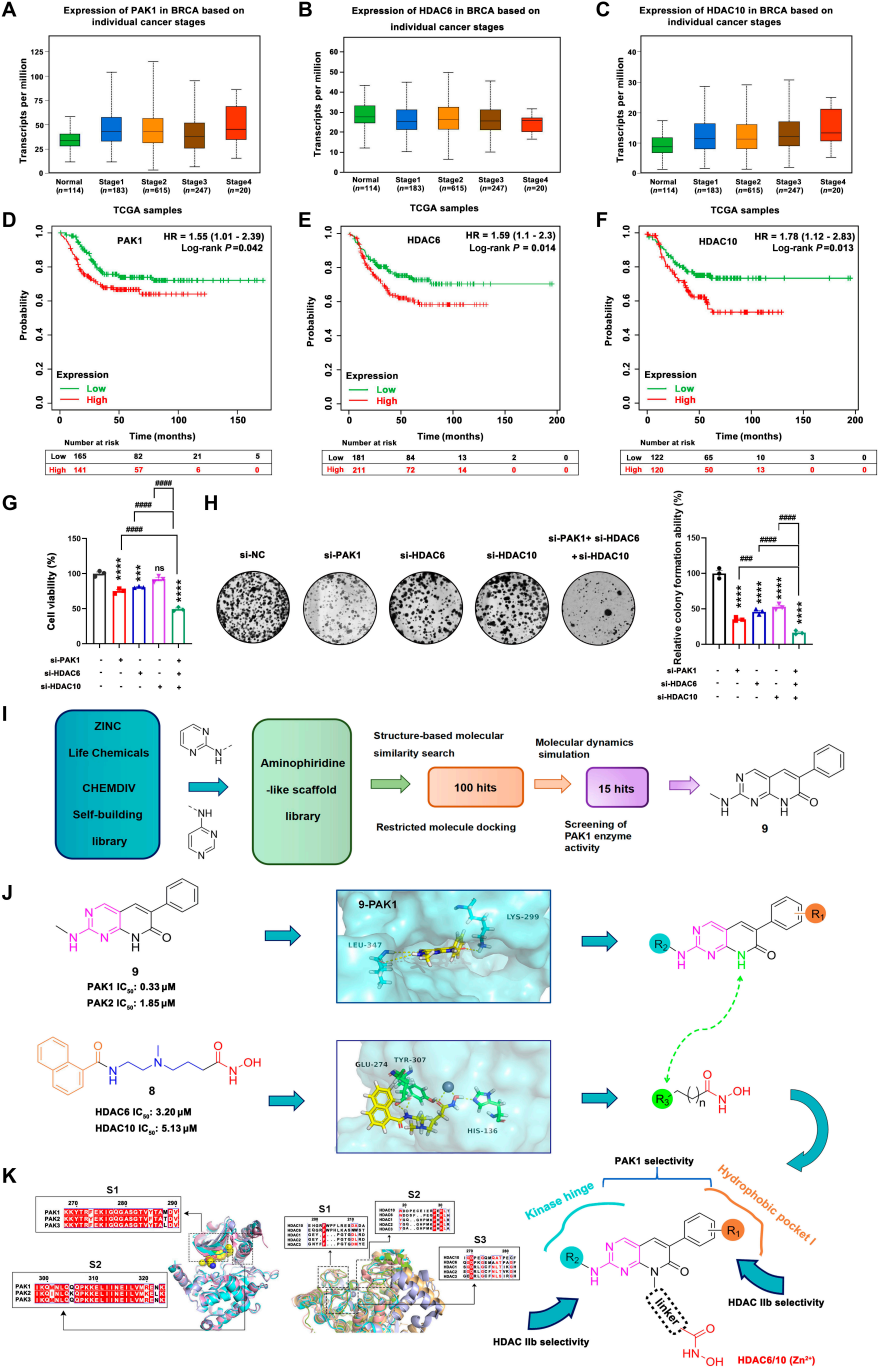
Expression and prognostic analysis of PAK1 and HDAC IIb and design of novel PAK1 and HDAC IIb inhibitors. (A to C) Analyzing the expression of PAK1, HDAC6, and HDAC10 in patients of breast cancer using the Betastasis website (https://www.betastasis.com/). (D to F) Cox proportional risk modeling for survival analysis of PAK1, HDAC6, and HDAC10. (G) Transfected MDA-MB-231 cells with si-PAK1, si-HDAC6, and si-HDAC10 plasmids for 48 h, and after which the viability of the MDA-MB-231 cells was assessed. (H) Transfected MDA-MB-231 cells with si-PAK1, si-HDAC6, and si-HDAC10 plasmids for 2 weeks. The cell proliferation ability was measured by colony formation assay. (I) Flow chart of lead compound screening. (J) Analysis of key active groups based on PAK1 and HDAC IIb structures. (K) Structure optimization strategy based on sequence alignment and structure overlap.

To confirm PAK1 and HDAC IIb as potential therapeutic targets for breast cancer, we analyzed their expression levels and survival curves during different stages of tumor development. First, we examined the levels of *PAK1*, *HDAC6*, and *HDAC10* in breast cancer tissues at different disease stages. Our findings revealed that the levels of *PAK1* and *HDAC10* were elevated compared to those observed in individuals without breast cancer across various stages of the disease, while the expression level of *HDAC6* displayed no significant change (Fig. 1A to C). Subsequently, the survival analysis of *PAK1* and *HDAC6/10* was performed by COX proportional risk modeling, which displayed that patient with high expression of *PAK1* and *HDAC6/10* had hazard ratios (HRs) of 1.55 (1.01 to 2.39), 1.59 (1.10 to 2.30), and 1.78 (1.12 to 2.83), respectively, compared with those with low expression (Fig. 1D to F). The log-rank *P* values were all less than 0.05, suggesting that patients with high expression of *PAK1* and *HDAC6/10* had higher mortality rates than those with low expression, indicating the potential of *PAK1* and *HDAC10* as therapeutic targets for breast cancer. Furthermore, the individual knockdown of *PAK1* or *HDAC6* markedly reduced the viability and proliferation of MDA-MB-231 cells. Simultaneous knockdown of *PAK1*, *HDAC6*, and *HDAC10* resulted in an even more pronounced inhibition of cell viability and proliferation (Fig. 1G and H). We further investigated the anti-proliferative effects of the combination of FRAX486 (PAK1 inhibitor) [45], tubastatin A (HDAC6 inhibitor, also exhibits inhibitory effects on HDAC10) [46], and DKFZ-748 (HDAC10 inhibitor) [43] on MDA-MB-231 cells and observed synergistic effects at most concentrations (Tables S1 and S2). Collectively, inhibiting PAK1 and HDAC IIb may be a potentially promising strategy for TNBC treatment.

### Rational design of novel triple-targeting PAK1/HDAC6/HDAC10 inhibitors

The acquisition of a potent PAK1 inhibitor with a simplistic structure and facile synthesis is pivotal for the discovery of inhibitors against PAK1 and HDAC IIb. In our prior study, we performed the development and production of a wide range of compounds targeting PAK1, and conducted a comprehensive review of PAK1 inhibitors [17,25–27,47]. The majority of PAK1 inhibitors share a structural characteristic, an aminopyrimidine moiety, which is crucial for maintaining PAK1 inhibitory activity by establishing the indispensable hydrogen bonds with the residue Leu^347^ located in the kinase hinge. A collection of compounds with aminopyridine moiety was generated by employing a structural similarity searching of commercially accessible databases (ZINC, CHEMDIV, and Life Chemicals), as well as our internal library. The subsequent step involved a restricted molecular docking to identify potential hits with the capability of forming key hydrogen interactions with the kinase hinge of PAK1, leading to the identification of the top 100 candidates based on their docking scores. Furthermore, a molecular dynamics (MD) simulation was employed to reevaluate the 100 hits, resulting in the acquisition of 15 compounds through procurement and synthesis (Fig. 1I). Fortunately, 2-(methylamino)-6-phenylpyrido[2,3-d] pyrimidin-7(8*H*)-one (**9**) displayed potent inhibitory activity against PAK1 with an IC_50_ value of 0.33 μM, and PAK2 with an IC_50_ value of 1.85 μM. However, compound **9** displayed a weak anti-proliferative potency with an IC_50_ value of 42.3 μM in MDA-MB-231 cells. The weak cellular activity is potentially attributed to its poor solubility with inadequate cellular uptake, which does not impede its potential as a promising starting point for further optimization owing to its good potential selectivity and straightforward chemical scaffold. The docking of **9** and PAK1 reveals that the aminopyridine moiety initiates 2 anticipative hydrogen interactions with residue Leu^347^, while the phenyl group is effectively positioned to a hydrophobic pocket. Notably, the caprolactam group of **9** is oriented toward the solvent-exposed region without affecting PAK1 binding, which is an appropriate linker attachment site for introducing the HDAC10-binding moiety—hydroxamic acid group (Fig. 1J). To achieve the desired selectivity toward PAK1 and HDAC IIb over other PAKs and HDACs, a structural analysis of PAKs and HDACs was systematically conducted. Although the sequences of PAKs show a high homology, there are still some differences in the 3-dimensional (3D) structure, which provides potential to achieve the selectivity of isoforms. The selectivity of PAKs primarily originates from the hydrophobic pockets composed of S1 and S2. Therefore, achieving selectivity for PAK1 can be anticipated through structural modifications of the R_1_ and R_2_ groups.

Based on the reported HDAC IIb inhibitors, we found that alkylated hydroxamic acid groups are still the optimal choice to achieve dual-targeting HDAC6/10. Through sequence comparison and protein crystal structure superposition (HDAC1 to HDAC3, HDAC6, and HDAC10), we have identified 3 potential sites (S1 to S3) that could achieve the isoforms selectivity by interactions with the “CAP” (surface recognition) groups. The structural distinction provides the basis for the selectivity of HDAC6/10 (Fig. 1K). Consequently, a hydroxamic acid moiety was grafted into the hexanolactam scaffold through various types and lengths of side chains to serve as a zinc-binding group (ZBG) that chelates the catalytic zinc ion in a bidentate manner. The modification of the 2-(methylamino)-6-phenylpyrido[2,3-d] pyrimidin-7(8*H*)-one core is tolerant of the size and substitution patterns of the CAP group. In addition, given the generally poor PK properties of HDAC inhibitors, we selected metabolically stable alkanes by directly derivatizing the N atom of the lactam of the CAP group to enhance their metabolic stability. Collectively, a series of 2-(methylamino)-6-phenylpyrido[2,3-d] pyrimidin-7(8*H*)-one derivatives were designed to be potent triple-targeting PAK1/HDAC6/HDAC10 inhibitors, as illustrated in Fig. 1.

### Chemical synthesis and structure–activity relationship

**Fig. 2.**
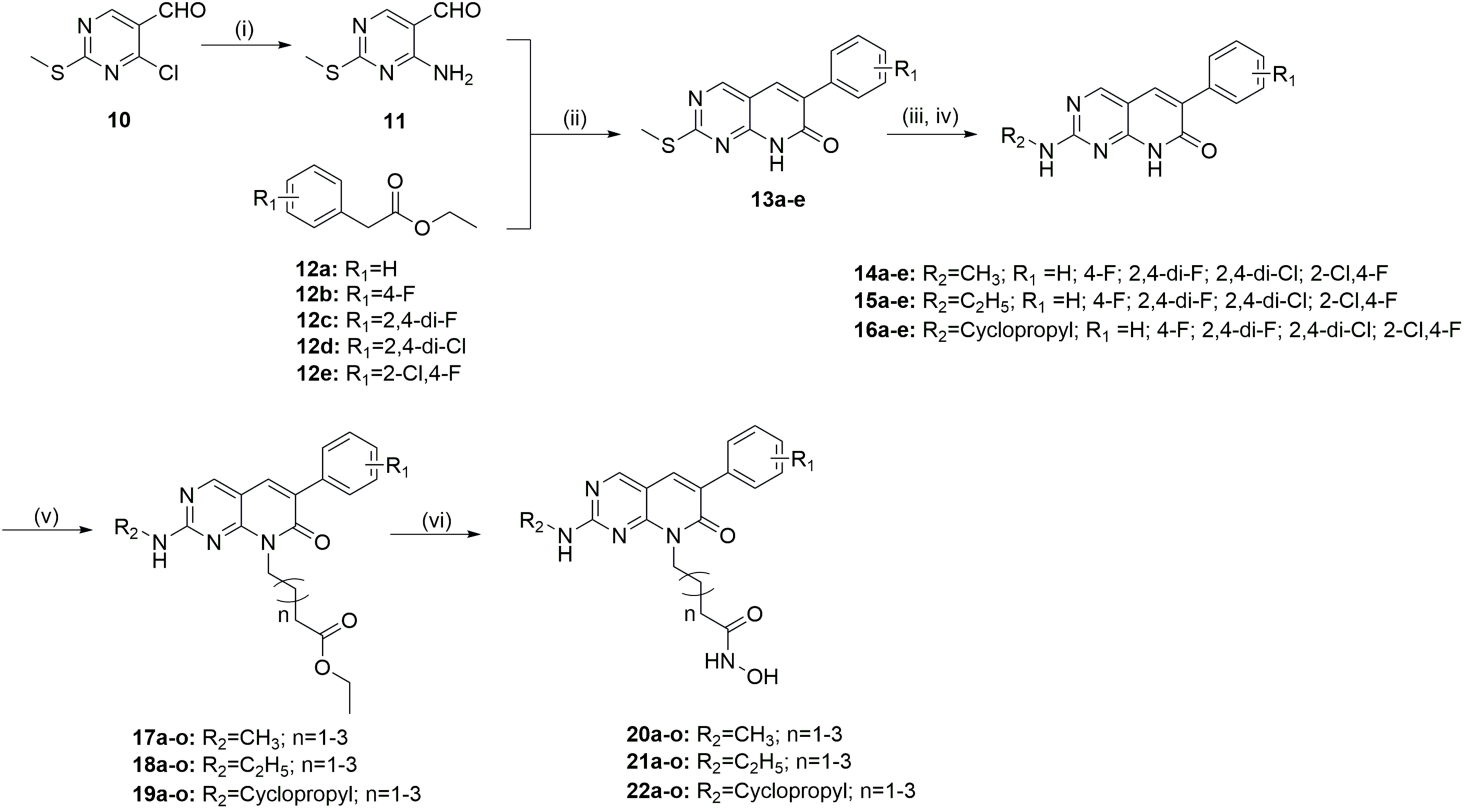
General synthesis of compounds 20a to 20o, 21a to 21o, and 22a to 22o. Reagents and experimental conditions: (i) NH_4_OH, triethylamine (TEA), tetrahydrofuran (THF), room temperature, 2 h; (ii) K_2_CO_3_, *N*,*N*-dimethylformamide (DMF), 120 °C; (iii) m-chloroperbenzoic acid (m-CPBA), CH_2_Cl_2_, overnight, room temperature; (iv) amine derivatives, DIEA, isopropyl alcohol, 90 °C, overnight; (v) halogenated carboxylic acid esters, K_2_CO_3_, DMF, overnight; (vi) NH_2_OH.HCl, NaOH, CH_3_OH, 1 to 3 h.

**Fig. 3.**
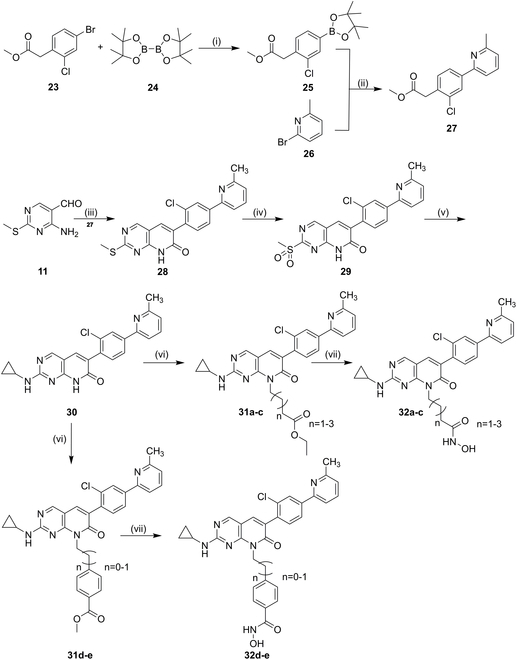
General synthesis of compounds 32a to 32e. Reagents and conditions: (i) Pd(dppf)Cl_2_, KOAc, dioxane, 4 h, 80 °C; (ii) Pd(PPh_3_)_4_, K_2_CO_3_, 24 h, 85 °C; (iii) K_2_CO_3_, DMF, 120 °C; (iv) m-CPBA, CH_2_Cl_2_, overnight, room temperature; (v) amine derivatives, DIEA, isopropyl alcohol, 90 °C, overnight; (vi) halogenated carboxylic acid esters, K_2_CO_3_, DMF, overnight; (vii) NH_2_OH.HCl, NaOH, CH_3_OH, 1 to 3 h.

The synthetic routes for compounds **20a** to **20o**, **21a** to **21o, 22a** to **22o**, and **32a** to **32e** were displayed in Figs. 2 and 3. Intermediate **11** was prepared by an ammonolysis of 4-chloro-2-(methylthio) pyrimidine-5-carbaldehyde (**10**). The key intermediates **13a** to **13e** were obtained by cyclization reaction of intermediate **11** and commercially available ethyl phenylacetate derivatives (**12a** to **12e**). To prepare intermediates **14a** to **14e**, **15a** to **15e**, and **16a** to **16e**, intermediates **13a** to **13e** were oxidized by 3-chloroperoxybenzoic acid and then aminolyzed with amine derivative. Next, an alkyl side chain was induced by the reaction of halogenated carboxylic acid esters with intermediates **14**, **15**, and **16**, yielding intermediates **17a** to **17o**, **18a** to **18o**, and **19a** to **19o**. The desired compounds **20a** to **20o**, **21a** to **21o**, and **22a** to **22o** were obtained by reacting with hydroxylamine. Additionally, intermediate **27** was prepared by a Suzuki reaction of arylboronic acid (**25**) and 2-bromo-6-methylpyridine (**26**), and cyclized with intermediate **11** to yield **28**. The intermediate **30** was obtained by oxidation and amolysis in turn, and the target products were further prepared by substitution reaction and hydroxylamine hydrolysis.

To obtain potent PAK1 and HDAC IIb inhibitors under the scrutiny of docking **9** and PAK1, we first incorporated a butyl hydroxamic acid group into the N atom of lactam moiety, yielding **20a**. Unfortunately, **20a** displayed no activity against HDAC6/10 and low activity against PAK1, and we reasoned that the side chain was too short to bind the ZBG pocket. Meanwhile, the side chain might disturb the active conformation of the ligand to bind the ATP pocket of PAK1. Hence, several longer side-chain derivatives (**20b** and **20c**) were synthesized, and **20b** showed a slight improvement against PAK1, but not still for HDAC6/10 (Table 1). While, the inhibitory activities of **20c** against HDAC6/10 markedly increased, but **20c** displayed a weak inhibition potency against PAK1. We have observed that the substituents on the benzene ring and the length of the side chain concurrently influence the molecular configuration, thereby impacting the inhibitory activities of PAK1 and HDAC6/10. Consequently, it becomes imperative to identify a structurally optimal equilibrium point for triple-targeted activity. Next, we introduced different substituent groups into the phenyl group to explore the balance in activities. Installation of a fluorine atom at the para-site of the phenyl yielded **20d** to **20f**, **20e** is 4 times more potent than **20a** in PAK1 inhibition, while **20f** displayed inhibition activity against HDAC6/10 with IC_50_ values of 0.44 and 0.27 μM, respectively. The addition of fluorine at C2 of **20d** to **20f** led to **20g** to **20i**, the inhibitory activity against HDAC6/10 increased with the increase of side-chain length, but inhibitory activity against PAK1 showed a slight decrease compared with **20a**. Additionally, 2,4-dichloro- and 2-chloro-4-fluoro substituted derivatives (**20j** to **20o**) were synthesized, and only **20o** showed potent inhibitory activity against PAK1 with IC_50_ = 0.21 μM, a moderate inhibition activity against HDAC10 with IC_50_ = 0.61 μM, and a weak inhibition activity against HDAC6 with IC_50_ = 1.32 μM.

**Table 1.** In vitro PAK1 and HDAC IIb inhibition and antitumor activity FX1

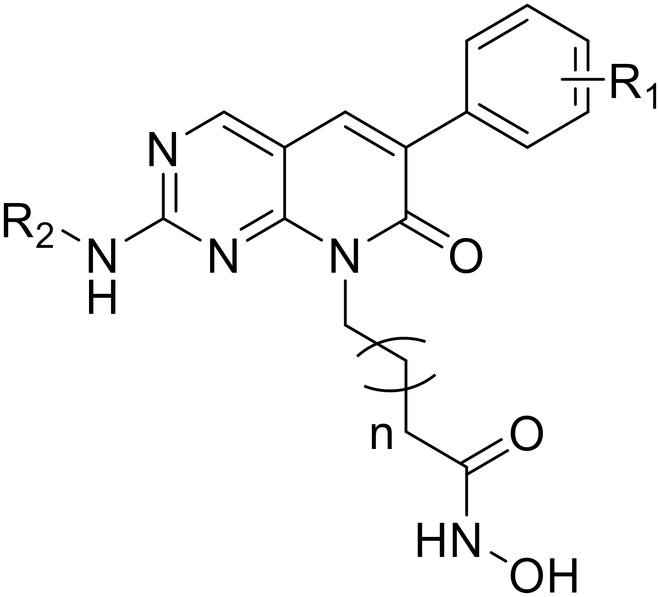							
Compound	R_2_	R_1_	*n*	Inhibitory activity (IC_50_, μM)^a^			Anti-proliferative activity (IC_50_, μM)^a^
				HDAC6	HDAC10	PAK1	MDA-MB-231 cells
**20a**	CH_3_	H	1	>10	>10	3.31 ± 0.64	>50
**20b**	CH_3_	H	2	>10	>10	1.08 ± 0.17	49.42 ± 4.74
**20c**	CH_3_	H	3	0.13 ± 0.04	0.62 ± 0.11	>10	27.27 ± 1.91
**20d**	CH_3_	4-F	1	6.25 ± 1.62	>10	5.11 ± 0.92	>50
**20e**	CH_3_	4-F	2	3.34 ± 0.98	5.25 ± 0.91	0.77 ± 0.14	>50
**20f**	CH_3_	4-F	3	0.44 ± 0.11	0.27 ± 0.06	1.92 ± 0.48	19.22 ± 1.45
**20g**	CH_3_	2,4-di-F	1	>10	>10	5.13 ± 0.91	45.56 ± 5.32
**20h**	CH_3_	2,4-di-F	2	4.21 ± 1.37	1.32 ± 0.25	7.23 ± 1.35	43.22 ± 3.80
**20i**	CH_3_	2,4-di-F	3	0.19 ± 0.05	0.21 ± 0.04	4.91 ± 0.72	21.60 ± 2.31
**20j**	CH_3_	2,4-di-Cl	1	>10	>10	1.58 ± 0.37	24.75 ± 2.71
**20k**	CH_3_	2,4-di-Cl	2	5.69 ± 1.17	>10	5.11 ± 0.83	>50
**20l**	CH_3_	2,4-di-Cl	3	0.74 ± 0.21	0.46 ± 0.07	4.36 ± 0.70	>50
**20m**	CH_3_	2-Cl,4-F	1	>10	>10	1.07 ± 0.14	>50
**20n**	CH_3_	2-Cl,4-F	2	5.34 ± 1.75	>10	1.82 ± 0.27	>50
**20o**	CH_3_	2-Cl,4-F	3	1.32 ± 0.31	0.61 ± 0.09	0.21 ± 0.04	27.60 ± 1.93
**21a**	C_2_H_5_	H	1	>10	>10	2.15 ± 0.36	>50
**21b**	C_2_H_5_	H	2	0.87 ± 0.28	1.46 ± 0.21	5.42 ± 0.84	47.61 ± 6.30
**21c**	C_2_H_5_	H	3	0.19 ± 0.05	0.33 ± 0.05	0.25 ± 0.06	17.11 ± 1.25
**21d**	C_2_H_5_	4-F	1	>10	7.39 ± 1.07	0.72 ± 0.14	>50
**21e**	C_2_H_5_	4-F	2	6.78 ± 2.01	1.18 ± 0.21	2.54 ± 0.43	>50
**21f**	C_2_H_5_	4-F	3	0.23 ± 0.05	0.13 ± 0.03	1.06 ± 0.18	16.20 ± 1.11
**21g**	C_2_H_5_	2,4-di-F	1	>10	>10	0.36 ± 0.05	43.53 ± 5.35
**21h**	C_2_H_5_	2,4-di-F	2	>10	5.98 ± 0.63	0.24 ± 0.05	35.47 ± 2.93
**21i**	C_2_H_5_	2,4-di-F	3	1.25 ± 0.36	0.37 ± 0.04	5.33 ± 0.84	26.72 ± 1.38
**21j**	C_2_H_5_	2,4-di-Cl	1	>10	>10	0.11 ± 0.03	>50
**21k**	C_2_H_5_	2,4-di-Cl	2	2.07 ± 0.49	1.14 ± 0.21	0.26 ± 0.06	33.29 ± 2.23
**21l**	C_2_H_5_	2,4-di-Cl	3	1.05 ± 0.27	0.11 ± 0.03	0.21 ± 0.05	9.12 ± 0.84
**21m**	C_2_H_5_	2-Cl,4-F	1	>10	>10	>10	>50
**21n**	C_2_H_5_	2-Cl,4-F	2	1.49 ± 0.55	2.34 ± 0.45	7.31 ± 1.67	>50
**21o**	C_2_H_5_	2-Cl,4-F	3	0.78 ± 0.17	0.12 ± 0.03	>10	24.56 ± 3.72
**22a**	Cyclopropyl	H	1	6.83 ± 2.11	>10	0.13 ± 0.04	26.12 ± 2.11
**22b**	Cyclopropyl	H	2	0.32 ± 0.06	0.11 ± 0.03	0.35 ± .06	4.57 ± 0.33
**22c**	Cyclopropyl	H	3	0.11 ± 0.03	0.09 ± 0.02	0.54 ± 0.11	11.20 ± 0.72
**22d**	Cyclopropyl	4-F	1	>10	7.45 ± 1.32	1.78 ± 0.35	34.32 ± 1.86
**22e**	Cyclopropyl	4-F	2	0.23 ± 0.05	0.12 ± 0.03	4.24 ± 0.79	38.13 ± 3.32
**22f**	Cyclopropyl	4-F	3	0.78 ± 0.29	0.28 ± 0.06	7.12 ± 1.15	24.22 ± 1.24
**22g**	Cyclopropyl	2,4-di-F	1	>10	4.14 ± 0.97	0.13 ± 0.04	9.12 ± 0.71
**22h**	Cyclopropyl	2,4-di-F	2	1.12 ± 0.34	0.15 ± 0.03	0.11 ± 0.04	3.66 ± 0.53
**22i**	Cyclopropyl	2,4-di-F	3	0.25 ± 0.06	0.04 ± 0.01	0.09 ± 0.03	2.22 ± 0.78
**22j**	Cyclopropyl	2,4-di-Cl	1	>10	6.25 ± 1.34	2.14 ± 0.55	34.43 ± 5.22
**22k**	Cyclopropyl	2,4-di-Cl	2	0.42 ± 0.13	0.15 ± 0.03	0.12 ± 0.03	4.75 ± 0.36
**22l**	Cyclopropyl	2,4-di-Cl	3	0.04 ± 0.01	0.20 ± 0.04	0.01 ± 0.003	0.98 ± 0.22
**22m**	Cyclopropyl	2-Cl,4-F	1	>10	>10	1.24 ± 0.31	42.41 ± 4.35
**22n**	Cyclopropyl	2-Cl,4-F	2	0.13 ± 0.04	0.43 ± 0.08	1.35 ± 0.27	13.24 ± 1.67
**22o**	Cyclopropyl	2-Cl,4-F	3	0.09 ± 0.03	0.18 ± 0.06	0.21 ± 0.04	6.41 ± 0.86
SAHA	-	-	-	0.02 ± 0.01	0.06 ± 0.01	-	8.82 ± 0.52
FRAX597	-	-	-	-	-	0.01 ± 0.003	2.52 ± 0.74

^a^ Each compound was tested in triplicate; the data are presented as the mean ± SD.

Given the structure–activity relationship (SAR) of the phenyl substituent groups and that the length of the side chain was not clear, we hypothesized that there must be some other factors involved in regulating the balance of PAK1 and HDAC6/10 inhibition activity. According to our previous experience and report, an extra hydrophobic interaction site occupied by methyl of **9** is also important for the maintenance of kinase inhibitor activity and elevation of selectivity against HDAC IIb. A series of ethyl-substituted derivatives (**21a** to **21o**) were synthesized, and most of the derivatives containing 6 carbon atom side chains displayed potent inhibitory activity against HDAC IIb and 2,4-dichloro substitution (**21j** to **21l**) contributing to the increase of PAK1 activity. Encouraged by the finding, cyclopropyl, a bigger group, was coupled into the aminopyrimidine core to occupy the additional hydrophobic site. **22b** showed potent inhibitory activity against PAK1 and HDAC6/10 with IC_50_ values of 0.35, 0.32, and 0.11 μM, respectively, as well as potent antiproliferatory activity with an IC_50_ value of 4.57 μM in MDA-MB-231 cells. Further extension of the side chain resulted in **22c**, which showed a slight improvement of HDAC IIb inhibition potency compared to **22b**, but a slight decline in activity against PAK1. To clarify the structure–activity relationship, we further explored the effects of different substituted benzene rings and different length side chains on the activity, leading to compounds **22d** to **22o**. **22i** achieved a very potent inhibitory potency against both PAK1 and HDAC10 with IC_50_ values of 0.09 and 0.04 μM, but a relatively weak inhibition activity against HDAC6, still resulting in potent inhibitory activity in MDA-MB-231 cells with an IC_50_ value of 2.22 μM. Furthermore, compound **22l** exhibited the most potent inhibitory activity with IC_50_ values of 0.01 and 0.04 μM against PAK1 and HDAC6, respectively, and an IC_50_ of 0.20 μM for HDAC10. This compound effectively inhibited cell growth in MDA-MB-231 cells with an IC_50_ value of 0.98 μM, demonstrating superior efficacy compared to the reference compounds (SAHA and FRAX597) (Table 1). The cyclopropyl group plays an essential role in maintaining inhibition against PAK1, and the substituted groups of the benzene ring could function to regulate the balance of PAK1 and HDAC IIb inhibition activity by delicately modulating the conformation of ligands. Furthermore, we observed inadequate occupancy of the adjacent hydrophobic pocket by the 4-position substituent (Cl or F atom) on the benzene ring within the PAK1 kinase domain. Introducing a larger hydrophobic group could potentially enhance both PKA1 inhibitory activity and selectivity, aligning with the characteristics exhibited by type II kinase inhibitors (Table 2). Hence, 2-methylpyridine moiety was induced into the 4-site of the benzene ring and the length of the side chains ranges from 4 to 6 carbon atoms, yielding **32a** to **32c**. As expected, **32c** (named as ZMF-25) exhibited the most potent inhibitory activity against PAK1/HDAC6/10, with IC_50_ values of 0.03, 0.06, and 0.04 μM, respectively. Additionally, it effectively inhibited cell growth in MDA-MB-231 cells, with an IC_50_ value of 0.76 μM. Interestingly, ZMF-25 had very low cytotoxicity on normal breast cells MCF-10A (IC_50_ > 100 μM; Fig. S2). Next, replacements of alkyl side chains with aromatic groups were implemented to yield compounds **32d** and **32e**, and a significant decrease was observed in inhibitory potency of both PAK1 and HDAC10, but the 2 compounds still exhibited a potent inhibitory potency against HDAC6. Further enzyme inhibition selectivity tests indicated that ZMF-25 displayed good selectivity for HDAC IIb and PAK1 (Table 3). Therefore, ZMF-25 represents a novel and potent inhibitor of PAK1, HDAC6, and HDAC10.

**Table 2.** In vitro PAK1 and HDAC IIb inhibition and antitumor activity

Compound	*n*	Inhibitory activity (IC_50_, μM) ^a^		Anti-proliferative activity (IC_50_, μM)^a^	
		HDAC6	HDAC10	PAK1	MDA-MB-231 cells
**32a**	1	>10	4.82 ± 0.76	0.21 ± 0.22	19.41 ± 3.52
**32b**	2	0.12 ± 0.04	0.09 ± 0.02	0.15 ± 0.03	1.73 ± 0.36
**32c** (ZMF-25)	3	0.06 ± 0.02	0.04 ± 0.01	0.03 ± 0.006	0.76 ± 0.12
**32d**	0	0.07 ± 0.03	6.38 ± 1.27	0.34 ± 0.07	7.45 ± 1.03
**32e**	1	0.03 ± 0.01	1.29 ± 0.31	0.12 ± 0.03	5.39 ± 0.87
SAHA	-	0.02 ± 0.01	0.06 ± 0.01	-	8.82 ± 0.52
FRAX597	-	-	-	0.01 ± 0.003	2.52 ± 0.74

^a^ Each compound was tested in triplicate; the data are presented as the mean ± SD.

**Table 3.** Inhibitory activity of ZMF-25 against different HDACs and PAK family members

Isoforms	HDACs/PAKs	IC_50_ (μM)^a^
HDAC class I	HDAC1	2.33 ± 0.65
	HDAC2	0.85 ± 0.24
	HDAC3	3.45 ± 0.78
	HDAC8	7.12 ± 2.35
HDAC class IIb	HDAC6	0.064 ± 0.025
	HDAC10	0.041 ± 0.010
PAKs	PAK1	0.033 ± 0.006
	PAK2	0.37 ± 0.06
	PAK3	1.46 ± 0.44

^a^ Each compound was tested in triplicate; the data are presented as the mean ± SD.

### Docking and MD simulation reveals the binding between ZMF-25 and PAK1/HDAC6/HDAC10

**Fig. 4.**
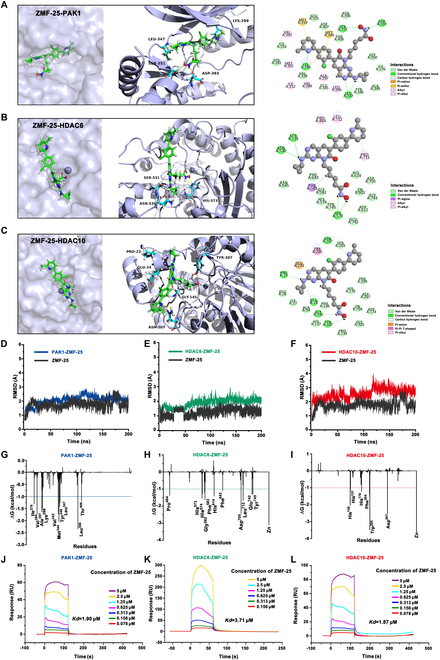
Molecular docking analysis of ZMF-25 with PAK1, HDAC6, and HDAC10. (A to C) Surface, cartoon views, and detailed interactions of ZMF-25 and PAK1/HDAC6/HDAC10. (D to F) RMSD of the atoms within 5 Å of ZMF-25 for PAK1 (blue), HDAC6 (green), and HDAC10 (red). (G to I) Residue-based energy decomposition analysis of ZMF-25 and interactions with PAK1 (represented in blue), HDAC6 (represented in green), and HDAC10 (represented in red). (J to L) SPR assay of ZMF-25 with PAK1, HDAC6, and HDAC10.

To elucidate the binding mechanisms of ZMF-25 with PAK1 and HDAC IIb, molecular docking studies were conducted. In docking of ZMF-25 and PAK1, the aminopyrimidine moiety initiates 2 crucial hydrogen interactions with Leu^347^ residue located in the kinase hinge of PAK1, thereby playing a pivotal role in maintaining inhibitory activity (Fig. 4A). The pyrido[2,3-d] pyrimidin-7(8*H*)-one moiety occupied a hydrophobic pocket composed of Val^284^, Ala^297^, Met^301^, Ile^312^, and Val^342^ residues. Two π–sulfur interactions initiated by Met^319^ and Met^344^ were observed, and a π–cation interaction and hydrogen bond were formed with Lys^299^. Additionally, the methylpyridine moiety occupied an extra hydrophobic pocket typical of type II kinase inhibitors with good selectivity. Despite the high sequence similarity between PAK1 and PAK2, a critical amino acid difference of residues Arg^320^ and Asn^322^ at the bottom of the α helix in PAK1 causes an outward shift, resulting in a larger hydrophobic pocket that precisely accommodates the methylpyridine group. This structural alteration may explain the selective inhibition of PAK1 by ZMF-25. The hydroxamic acid side chain, located in the solvent-exposed region, forms 2 hydrogen bonds with the Ser^351^ and Asp^393^ residues, thereby stabilizing its active conformation. For HDAC6-ZMF-25 complex, the hydroxamic acid group chelated the catalytic zinc ion, and 2 hydrogen bonds were formed by residues His^573^. Additionally, the cyclopropylamine group initiated 2 key hydrogen bonds with residues Ser^531^ and Asn^530^ (Fig. 4B). The docking of ZMF-25 and HDAC10 revealed that the side chain of compound ZMF-25 was fully inserted into the substrate deacetylation binding site of HDAC10 via a narrow, hydrophobic channel, and the 6-phenylpyrido[2,3-d]pyrimidin-7(8H)-one core served as the CAP group, unshakably anchored to the substrate recognition site, resembling a nail. The hydroxamic acid group of ZMF-25 chelated the catalytic zinc ion in a bidentate manner, thereby establishing 2 conserved hydrogen bonds with residues Gly^145^ and Tyr^307^, respectively. Additionally, 2 additional hydrogen bonds were observed between the cyclopropylamine moiety with residues Pro^23^ and Glu^24^. Notably, the pyrido[2,3-d] pyrimidin-7(8*H*)-one core establishes 2 crucial π–anion interactions with the key gatekeeper residue Glu^274^ (Fig. 4C). Furthermore, the 2-chloro-4-(6-methylpyridin-2-yl) phenyl moiety of ZMF-25 formed a π–π interaction with residue Trp^205^ and a hydrogen bond with residue Asn^207^, facilitating the maintenance of the stable conformation of the CAP group. Furthermore, MD simulations and free energy binding calculations were also performed. During a 200-ns MD simulation, the root mean square deviation (RMSD) of the protein’s αC atoms, within a 5-Å radius of the ligand-binding region, was evaluated. The RMSD values displayed minor fluctuations, suggesting that the system maintained stable behavior throughout the simulation (Fig. 4D to F). The subsequent energy decomposition analysis of the residues yielded results consistent with those obtained from molecular docking studies (Fig. 4G to I). Therefore, ZMF-25 represents a novel and potent dual inhibition of PAK1 and HDAC IIb. To further investigate the direct binding of ZMF-25 with PAK1 and HDAC6/10, we performed SPR assay and CETSA assays. The SPR results showed (Fig. 4J to L) that ZMF-25 was well combined with PAK1, HDAC6, and HDAC10 [PAK1 dissociation constant (*K*_d_) = 1.90 μM; HDAC6 *K*_d_ = 3.71 μM; HDAC10 *K*_d_ = 1.87 μM].

**Fig. 5.**
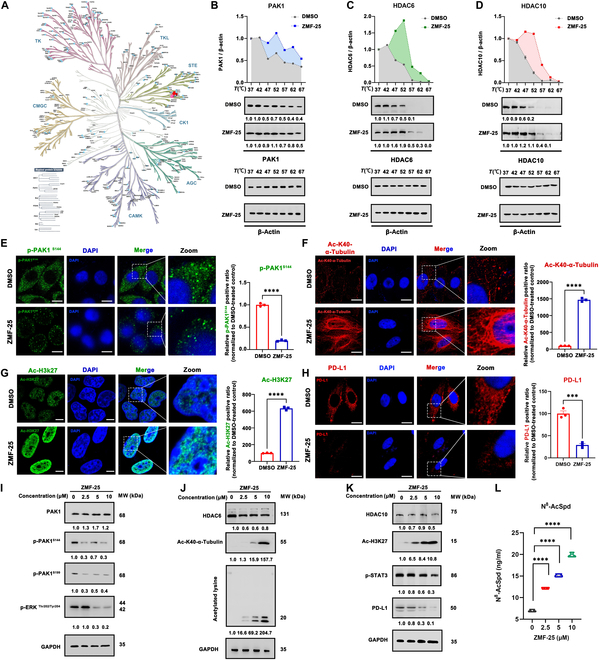
ZMF-25 blocks the activity of PAK1 and HDAC class IIb in MDA-MB-231 cells. (A) Single-point broad panel kinome screening against 1.0 μM ZMF-25. (B to D) The CETSA assay was conducted separately on ZMF-25 and the proteins PAK1, HDAC6, and HDAC10 in MDA-MB-231 cells. (E and F) The levels of phospho-PAK1 (Ser^144^) and acetylated α-tubulin (Lys^40^) following treatment with 5 μM ZMF-25 were assessed using immunofluorescence. Scale bar, 15 μm. ****P < 0.0001. (G) The levels of Ac-H3K27 in MDA-MB-231 cells following treatment with 5 μM ZMF-25 were assessed using immunofluorescence. Scale bar, 10 μm. ****P < 0.0001. (H) The expression levels of PD-L1 in MDA-MB-231 cells following treatment with 5 μM ZMF-25 were quantitatively assessed using immunofluorescence staining. Scale bar, 15 μm. ***P < 0.001. (I to K) Western blot analysis was employed to quantify the expression levels of PAK1, p-PAK1^S144^, p-PAK1^S199^, p-ERK^Thr202/Tyr204^, HDAC6, Ac-K40-α-Tubulin, acetylated lysine, HDAC10, Ac-H3K27, p-STAT3^Tyr705^, and PD-L1. (L) The content of N^8^-AcSpd in MDA-MB-231 cells treated with different concentrations of ZMF-25 for 24 h was measured by high-performance liquid chromatography (HPLC)–tandem mass spectrometry (MS/MS). ****P < 0.0001.

Furthermore, ZMF-25 exhibits significant selectivity for PAK1, demonstrating moderate inhibitory activity against PAK2, as evaluated across an extensive panel of 378 human protein kinases (Fig. 5A). As depicted in Fig. 5B to D, ZMF-25 demonstrated a significant enhancement in the thermal stability of PAK1/HDAC6/10 proteins in MDA-MB-231 cells, thereby suggesting a direct interaction between ZMF-25 and HDAC6/10 as well as PAK1 in MDA-MB-231 cells. In addition, to investigate the role of HDAC10 in anti-TNBC proliferation of ZMF-25, we employed HDAC10 inhibitor DKFZ-748 and si-HDAC10, respectively. The results showed that ZMF-25 demonstrated comparable inhibitory activity of HDAC10 as DKFZ-748 in MDA-MB-231 cells, and the inhibition of HDAC10 played a significant role in the anti-proliferation activity of ZMF-25 (Fig. S2). Consequently, these findings establish ZMF-25 as a novel selective potent triple inhibitor of PAK1/HDAC6/HDAC10.

### ZMF-25 inhibits the proliferation and migration of MDA-MB-231 cells through suppression of PAK1/HDAC6/HDAC10

Next, the inhibitory effects of ZMF-25 on PAK1/HDAC6/HDAC10 and its anti-proliferative activity against MDA-MB-231 cells were investigated in vitro. First, the expression of p-PAK1^S144^, Ac-K40-α-Tubulin, and Ac-H3K27 was measured by immunofluorescence. The level of p-PAK1^S144^ is indicative of the activation status of PAK1, while Ac-K40-α-Tubulin serves as a marker for HDAC6 activity and Ac-H3K27 reflects the activation degree of HDAC10. As illustrated in Fig. 5E to G, PAK1 displayed a homogeneous distribution throughout the cytoplasm in the control group. Following treatment with ZMF-25, a significant reduction in cytoplasmic PAK1 levels was observed, indicating robust inhibition of PAK1 activity. After ZMF-25 treatment, the contents of acetylated α-Tubulin(K40) and acetylated H3K27 in MDA-MB-231 cells were significantly increased, suggesting that ZMF-25 has an inhibitory effect on HDAC6 and HDAC10. Concurrently, we evaluated the impact of ZMF-25 on PD-L1 expression. The results demonstrated that treatment with ZMF-25 led to a significant reduction in PD-L1 expression in MDA-MB-231 cells (Fig. 5H). Subsequently, we examined the changes in substrate levels of PAK1, HDAC6, and HDAC10 in MDA-MB-231 cells following exposure to different concentrations of ZMF-25. For PAK1, we detected the phosphorylation of PAK1 at Ser^144^ and Ser^199^, and detected the phosphorylation of extracellular signal–regulated kinase 1/2 (ERK1/2) at Thr^202^/Tyr^204^. The results demonstrated that ZMF-25 markedly attenuated phosphorylation at these sites, indicating a PAK1-mediated suppression of the phosphorylation activation pathway (Fig. 5I). As for HDAC6, we examined the degree of acetylation of α-Tubulin(K40) in cells, as well as changes in total acetylated lysine in cells. Treatment with ZMF-25 significantly promoted the sites of α-Tubulin(K40) in cells and the degree of acetylation of total acetylated lysine in cells (Fig. 5J), confirming the inhibition of HDAC6. For HDAC10, we assessed the levels of H3K27 acetylation and signal transducer and activator of transcription 3 (STAT3) phosphorylation, as well as the expression of PD-L1. The results demonstrated that ZMF-25 markedly inhibited the acetylation of H3K27, the phosphorylation of STAT3, and the expression of PD-L1 (Fig. 5K). Importantly, we also examined the effect of ZMF-25 on the N^8^-AcSpd content in MDA-MB-231 cells, and the results showed that ZMF-25 significantly increased the N^8^-AcSpd content in MDA-MB-231 cells, further confirming the inhibitory effect of ZMF-25 on HDAC10 (Fig. 5L). Collectively, ZMF-25 was found to markedly suppress the activity of PAK1, HDAC6, and HDAC10 in TNBC cells.

**Fig. 6.**
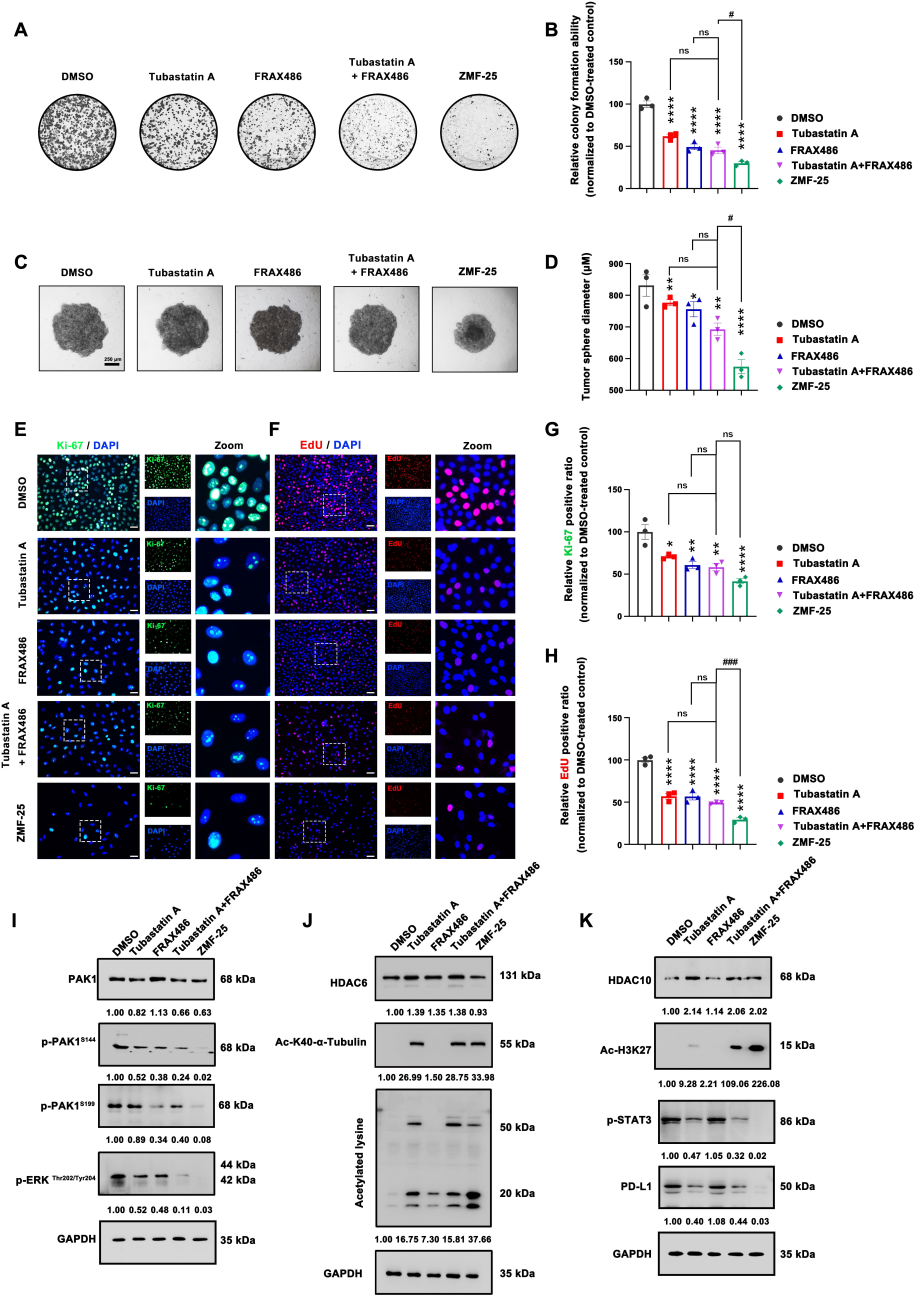
ZMF-25 inhibits the proliferation of MDA-MB-231 cells obviously. (A and B) Effect of ZMF-25 treatment on the colony formation capability of MDA-MB-231 cells. (C and D) Impact of ZMF-25 on 3D spheroid formation assay. (E and G) The relative intensity of Ki-67 was quantified using immunofluorescence. Scale bar, 200 μm. (F and H) Relative cell proliferation was assessed via the EdU assay. Scale bar, 200 μm. (I to K) Western blot analysis was employed to evaluate the expression levels of PAK1, HDAC6, HDAC10, p-PAK1^S144^, p-PAK1^S199^, p-ERK^Thr202/Tyr204^, Ac-K40-α-Tubulin, Ac-H3K27, p-STAT3^Tyr705^, PD-L1, and acetylated lysine after tubastatin A, FRAX486, tubastatin A + FRAX486, and ZMF-25 treatment.

The impact of ZMF-25 on the proliferation of MDA-MB-231 cells was subsequently evaluated. The antiproliferative efficacy of ZMF-25 was first assessed using a colony formation assay, which demonstrated that ZMF-25 exhibited significant anti-proliferative activity against MDA-MB-231 cells (Fig. 6A and B). The growth of MDA-MB-231 cells was simulated by 3D culture, and it was found that ZMF-25 treatment hindered the formation of 3D spheroids (Fig. 6C and D). Ki-67 is a cell proliferation marker, which can indirectly indicate the proportion of cells that are in the cell cycle by detecting its expression. The higher the positive rate of Ki-67, the faster the cell growth [48]. The expression levels of Ki-67 were assessed using immunofluorescence staining. The results demonstrated a significant reduction in Ki-67 intensity in MDA-MB-231 cells following ZMF-25 treatment (Fig. 6E and G). 5-Ethynyl-2’-deoxyuridine (EdU), a thymine nucleoside analog, can be integrated into the DNA double strand during DNA synthesis, and the detection of DNA replicative activity through the specific reaction based on the reaction of EdU with Apollo fluorescent dyes can accurately reflect cell proliferation [49]. These results demonstrated that ZMF-25 markedly reduced the green fluorescence intensity of EdU, further indicating that ZMF-25 effectively inhibited the proliferative activity of MDA-MB-231 cells (Fig. 6F and H). In addition, the anti-proliferation activities of tubastatin A and FRAX486 were also assayed, and the results showed that tubastatin A and FRAX486, as well as both of them, inhibited the colony formation ability and the formation of 3D spheroids, and the expression of Ki-67 as well as the staining of EdU also confirmed the anti-proliferation activity. Among these, ZMF-25 demonstrated the most remarkable inhibitory effect on MDA-MB-231 cell proliferation. Next, through Western blot analysis of PAK1, HDAC6, and HDAC10 along with their downstream substrates, we observed that ZMF-25 exhibited more potent inhibitory effects on PAK1, HDAC6, and HDAC10 compared to the combination of tubastatin A and FRAX486. This indicates that ZMF-25 possesses robust intracellular inhibitory activity against PAK1/HDAC6/HDAC10 in MDA-MB-231 cells (Fig. 6I to K). Taken together, the findings indicate that ZMF-25 can effectively inhibit the proliferation of MDA-MB-231 cells by inhibiting PAK1/HDAC6/HDAC10.

**Fig. 7.**
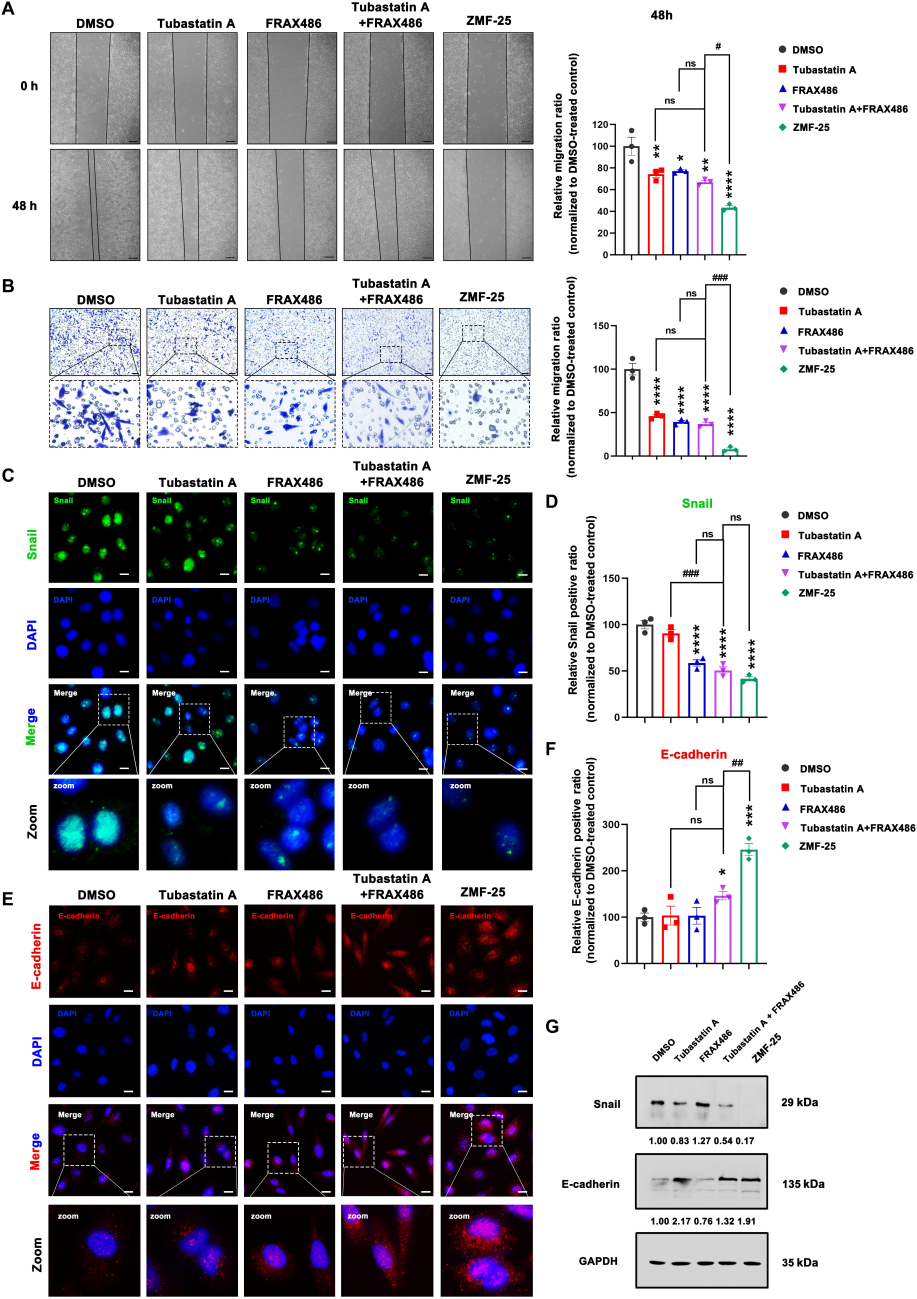
ZMF-25 inhibits the migratory capacity of MDA-MB-231 cells. (A) Wound healing assay (scale bar, 100 μm) and (B) Transwell assay of MDA-MB-231 cells following ZMF-25 treatment. Scale bar, 50 μm. (C to F) The relative fluorescence intensity of Snail and E-cadherin was quantified by immunofluorescence analysis. Scale bar, 10 μm. (G) Western blot analysis to evaluate the protein expression levels of E-cadherin and Snail in MDA-MB-231 cells.

To further investigate the antitumor effects of ZMF-25, we subsequently evaluated its anti-migratory effects. First, the wound healing assay results demonstrated that ZMF-25 significantly inhibited tumor cell migration (Fig. 7A). Furthermore, the transwell assay also demonstrated that ZMF-25 significantly inhibited the migratory capability of TNBC cells (Fig. 7B). Notably, it has been demonstrated that the expression of Snail is up-regulated in recurrent breast cancer tumors. This up-regulation is associated with metastasis and a reduced recurrence-free survival rate [50]. E-cadherin, a well-characterized tumor suppressor, plays a critical role in the processes of tumorigenesis and metastasis [51]. We examined the expression of Snail and E-cadherin after ZMF-25 treatment by immunofluorescence (Fig. 7C to F), and the results displayed that the fluorescence intensity of Snail was weaker and the fluorescence range was reduced compared with the Control group, indicating that ZMF-25 inhibited the expression of Snail. Meanwhile, the fluorescence intensity of E-cadherin was stronger and the fluorescence range increased compared with the control group, indicating that ZMF-25 promoted the expression of E-cadherin. The above results indicated that ZMF-25 effectively suppresses the migratory capability of MDA-MB-231 cells. Ultimately, the protein expression levels of Snail and E-cadherin were assessed via Western blot analysis. The findings further corroborated that ZMF-25 effectively inhibited the migratory capacity of MDA-MB-231 cells (Fig. 7G). The effects of tubastatin A and FRAX486 were also investigated, and the findings demonstrated that tubastatin A alone did not inhibit the expression of Snail in the cells, while FRAX486 and the simultaneous use of the two inhibited the expression of Snail. Neither tubastatin A nor FRAX486 alone promoted the expression of E-cadherin; only their combined use resulted in increased E-cadherin expression. However, results from the wound healing and transwell assays demonstrated that both compounds, whether used individually or in combination, could inhibit migration of MDA-MB-231 cells. Western blot analysis demonstrated that both tubastatin A alone and the combination of tubastatin A with FRAX486 effectively inhibited the expression of Snail while promoting the expression of E-cadherin. Furthermore, ZMF-25 exhibited superior inhibitory effects on migration compared to both tubastatin A and the combination of tubastatin A and FRAX486. In summary, ZMF-25 exhibits a potent migration inhibition activity in MDA-MB-231 cells.

### Transcriptomics-based analysis identifies the mechanism of ZMF-25 was involved in autophagy, glycolysis, and oxidative stress regulation

**Fig. 8.**
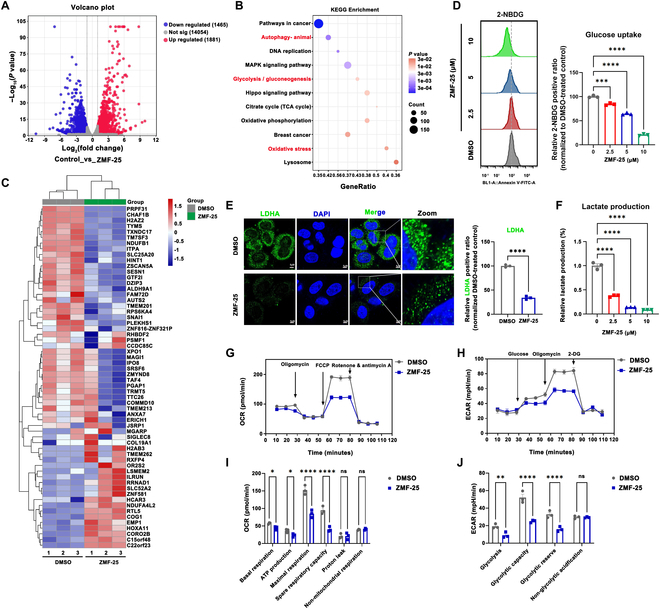
Transcriptomics-based analysis identifies the mechanism of ZMF-25-induced cell death. (A) The volcano plot of differentially expressed genes in MDA-MB-231 cells treated with ZMF-25 was analyzed. (B) Cluster analysis of differentially expressed genes based on Kyoto Encyclopedia of Genes and Genomes (KEGG) Pathway enrichment was conducted. (C) A heatmap of the top 60 differentially expressed genes in MDA-MB-231 cells treated with ZMF-25 was generated. (D) Flow cytometry with 2-deoxy-2-[(7-nitro-2,1,3-benzoxadiazol-4-yl)amino]-D-glucose (2-NBDG) staining was used to investigate the effect of different concentrations of ZMF-25 on the glucose uptake capacity of MDA-MB-231 cells. (E) Immunofluorescence staining was used to detect the expression and distribution of LDHA in MDA-MB-231 cells after 24 h of ZMF-25 treatment. (F) Lactate levels in MDA-MB-231 cells were measured following 24-h exposure to varying concentrations of ZMF-25. (G and I) Seahorse assay to assess the OCR of MDA-MB-231 cells after ZMF-25 treatment for 24 h. (H and J) Seahorse assay to assess the ECAR of MDA-MB-231 cells after ZMF-25 treatment for 24 h.

To elucidate the underlying mechanisms of ZMF-25-induced cell death in MDA-MB-231 cells, we conducted a transcriptomic analysis of TNBC cells following a 24-h treatment with ZMF-25. The results showed that 5,763 differential genes were found (Fig. 8A), and the major pathways with significant changes were autophagy, DNA replication, mitogen-activated protein kinase (MAPK) pathway, glycolysis, Hippo pathway, oxidative stress, etc. (Fig. 8B and C). Given that glycolysis is a critical survival mechanism for TNBC, we first examined the effect of ZMF-25 on the glucose uptake capacity of MDA-MB-231 cells. The results demonstrated a significant reduction in the glucose uptake capacity of MDA-MB-231 cells following treatment with ZMF-25 (Fig. 8D). Then, we examined the expression of lactate dehydrogenase A (LDHA), the rate-limiting enzyme of lactate production, in MDA-MB-231 cells after ZMF-25 treatment. Using immunofluorescence staining, we observed that LDHA was highly expressed in MDA-MB-231 cells and predominantly localized in the cytoplasm. Following treatment with ZMF-25, the expression level of LDHA was markedly decreased, indicating that ZMF-25 exerts an inhibitory effect on LDHA (Fig. 8E). Subsequently, through quantification of lactate content, we observed that ZMF-25 markedly suppressed lactate production in MDA-MB-231 cells (Fig. 8F). To further confirm the effects of ZMF-25 on mitochondrial respiration and energy metabolism of MDA-MB-231 cells, we performed the Seahorse assay to measure the oxygen consumption rate (OCR) (Fig. 8G and I) and extracellular acidification rate (ECAR) of TNBC cells (Fig. 8H and J). ZMF-25 significantly inhibited the basal respiration, ATP production, maximal respiration, and spare respiratory capacity of MDA-MB-231 cells, indicating the breakdown of mitochondrial respiration and energy metabolism. ECAR results demonstrated that ZMF-25 markedly reduced glycolytic activity in MDA-MB-231 cells. Specifically, both glycolytic capacity and glycolytic reserve were significantly diminished, indicating that ZMF-25 effectively inhibited MDA-MB-231 cells from deriving energy via glycolysis.

### ZMF-25 promotes ROS generation to facilitate mitochondrial damage and inhibit cell migration in MDA-MB-231 cells

**Fig. 9.**
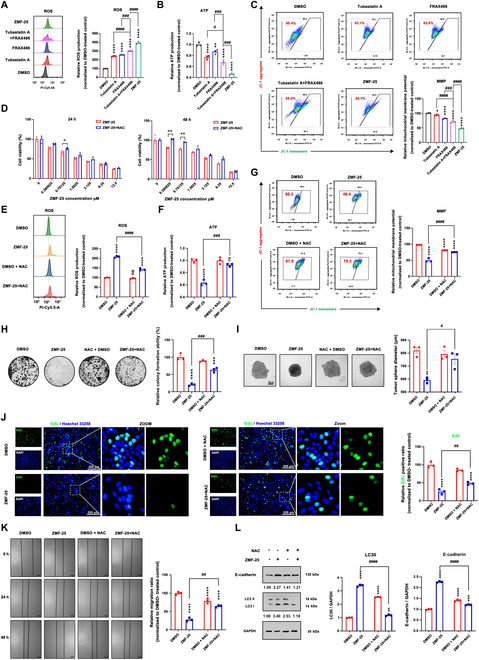
ZMF-25 promotes ROS generation and induces mitochondrial damage. (A and E) ROS production was assessed using flow cytometry. (B) ATP production was evaluated after ZMF-25 treatment. (C and G) Changes in mitochondrial membrane potential were detected via JC-1 staining. (D) Cell viability was checked by methylthiazolyldiphenyl-tetrazolium bromide (MTT) assay. (F) ATP production after NAC and ZMF-25 treatment was measured. (H) Colony formation ability of MDA-MB-231 cells following ZMF-25 or NAC treatment was examined. (I) 3D spheroid formation capacity of MDA-MB-231 cells after ZMF-25 treatment was assessed. (J) Relative EdU intensity was measured using the EdU incorporation assay. (K) Wound healing assays of cells treated with ZMF-25 or NAC. Scale bar, 100 μm. (L) Western blot analysis to measure E-cadherin and LC3 levels.

The mitochondria serve as the primary site for oxidative phosphorylation and ATP synthesis within cells, thus representing the pivotal hub of energy metabolism in organisms. The roles of mitochondria in cellular energy metabolism, ROS generation, and apoptosis are intricately linked to tumorigenesis. It has been demonstrated that PAK1 influences mitochondrial function via its interaction with the electron transport chain (ETC) [52] and that reducing PAK1 activity leads to an enhancement in NOX2-dependent ROS production [53]. We therefore examined mitochondrial function in ZMF-25-treated cells. ZMF-25 treatment induced a significant increase in ROS production (Fig. 9A), and a remarkable decrease in ATP production in MDA-MB-231 cells, indicating that the energy metabolism of mitochondria was impaired (Fig. 9B). Furthermore, mitochondrial membrane potential assays demonstrated that ZMF-25 induced mitochondrial damage in MDA-MB-231 cells (Fig. 9C). Both tubastatin A and FRAX486, either individually or in combination, were found to increase ROS production and induce mitochondrial dysfunction. In comparison, ZMF-25 demonstrated superior efficacy relative to tubastatin A and FRAX486.

To further explore the effect of ZMF-25-induced ROS production on TNBC cell survival, we investigated the effects of ZMF-25 using simultaneous treatment of cells with ZMF-25 and NAC (a ROS inhibitor). First, we investigated the alterations in cell viability of MDA-MB-231 cells following ZMF-25 treatment in the presence of NAC. The results showed that after adding NAC, the decrease of cell viability induced by different concentrations of ZMF-25 was improved to different degrees (Fig. 9D). Subsequently, the ROS assay results demonstrated a significant reduction in ROS levels in the NAC + ZMF-25 group compared to the ZMF-25 treatment group alone. This indicates that NAC effectively mitigated the ROS production in MDA-MB-231 cells following ZMF-25 administration (Fig. 9E). Later, detection of changes in ATP and JC-1 also showed that the ZMF-25-induced reduction in mitochondrial membrane potential and ATP production was significantly reversed after ROS inhibition with NAC, further demonstrating that ZMF-25-induced ROS accumulation in large amounts promotes the reduction in TNBC mitochondrial membrane potential and ATP production (Fig. 9F and G). Together, ZMF-25 could induce mitochondrial damage by promoting ROS generation in MDA-MB-231 cells.

Next, we investigated the correlation between ZMF-25-induced ROS accumulation and its effects on cell proliferation and metastasis. Through colony formation and 3D spheroid formation experiments (Fig. 9H and I), it was found that inhibiting ROS could significantly restore the clonogenesis and tumor spheroid formation ability. The EdU proliferation experiment also proved that inhibiting ROS could significantly restore the proliferating activity of TNBC cells (Fig. 9J), and ZMF-25-induced ROS accumulation significantly enhanced the inhibition of TNBC cell proliferation. Through a wound healing assay (Fig. 9K), we observed that inhibiting ROS markedly restored the migratory capacity of MDA-MB-231 cells. Additionally, by assessing the expression levels of E-cadherin, we found that ROS inhibition significantly prevented the ZMF-25-induced up-regulation of E-cadherin (Fig. 9L). These findings further confirm that ROS generation facilitates the migratory capacity induced by ZMF-25. Interestingly, ROS production typically induces autophagy in TNBC cells [54]. Therefore, we also investigated the effect of ZMF-25 on the autophagy marker protein LC3. The results demonstrated that ZMF-25 significantly promoted the accumulation of LC3-II, whereas inhibition of ROS markedly suppressed this accumulation. This prompted us to investigate the effect of ZMF-25 on autophagy.

### ZMF-25 promotes autophagy-related cell death in MDA-MB-231 cells

**Fig. 10.**
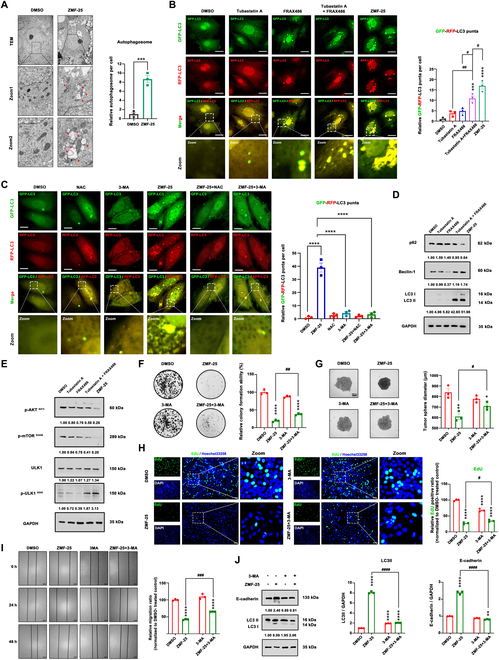
ZMF-25 promotes autophagy-related cell death in MDA-MB-231 cells. (A) The submicrostructure of MDA-MB-231 cells treated with ZMF-25 was checked by transmission electron microscopy (TEM). Scale bar, 5 μm. (B and C) The expression of LC3 was assessed via mRFP-GFP-LC3 transfection. Scale bar, 15 μm. (D) Western blot analysis to evaluate p62, LC3, and beclin-1 levels. (E) Western blot analysis to measure the phosphorylation levels of AKT (S473), mTOR (S2448), ULK1, and p-ULK1 (S555) in MDA-MB-231 cells. (F) Colony formation ability of MDA-MB-231 cells following ZMF-25 or 3-MA treatment. (G) The 3D spheroid formation ability of MDA-MB-231 cells after ZMF-25 or 3-MA treatment was assessed. (H) The relative intensity of EdU incorporation was quantified using the EdU assay. Scale bar, 100 μm. (I and J) Wound healing assays were conducted to examine the migratory capacity of MDA-MB-231 cells after ZMF-25 or 3-MA treatment. Scale bar, 100 μm.

Next, we further investigated whether ZMF-25 could induce autophagy in MDA-MB-231 cells. A significant increase in autophagic vesicles was observed in ZMF-25-treated MDA-MB-231 cells, indicating that ZMF-25 induces autophagy (Fig. 10A). Furthermore, ZMF-25 treatment initiated a significant decrease of p62 and strong regulation of LC3-II/I and beclin-1 using immunoblotting compared with the control group (Fig. 10D). The autophagy induced by ZMF-25 was further confirmed using a fluorescence assay, which showed a significant increase in both red and yellow spots in MDA-MB-231 cells transfected with monomeric red fluorescent protein (mRFP)-green fluorescent protein (GFP)-LC3 following ZMF-25 treatment (Fig. 10B). Next, we investigated the effects of ZMF-25 on the expression of key autophagy proteins p62, beclin-1, and LC3. Interestingly, tubastatin A and FRAX486 individually had little effect on LC3, p62, and beclin-1. However, their combined use resulted in a more pronounced impact on these markers. It was shown that both tubastatin A and FRAX486, either alone or in combination, induced autophagy to a limited extent. In comparison, ZMF-25 demonstrated a markedly stronger effect on autophagy than either compound individually or their combined use (Fig. 10D). Notably, the activation of the autophagy signaling pathway is facilitated by the ULK1 complex, which acts as an intermediary between the upstream nutrient or energy receptors mTOR and AMPK, and the subsequent formation of autophagosomes in vivo [55]. The phosphorylation of ULK1 plays a crucial role in regulating autophagy, exerting a facilitative impact on the process [56]. Then, we further detected the expression of p-AKT^S473^, p-mTOR^S2448^, ULK1, and p-ULK1^S555^. The results demonstrated that ZMF-25 inhibited AKT activation, thereby suppressing mTOR activation and promoting ULK1 phosphorylation, which ultimately led to autophagy (Fig. 10E). In addition, the AKT-mTOR pathway is a critical regulator of TNBC proliferation [57], and these findings further demonstrate the inhibitory effect of ZMF-25 on TNBC cell proliferation signaling. Together, ZMF-25 triggered autophagy in MDA-MB-231 cells by inhibiting the AKT/mTOR/ULK1 signaling pathway.

Subsequently, we examined the relationship between ZMF-25-induced autophagy and its effects on cellular proliferation and metastatic behavior. The results demonstrated that the addition of either NAC or 3-methyladenine (3-MA) inhibited LC3 aggregation, further corroborating that ZMF-25-induced ROS enhances autophagy (Fig. 10C). Through colony formation experiments and 3D-tumor ball formation experiments, we found that inhibiting autophagy with 3-MA significantly restored the proliferative capacity of MDA-MB-231 cells following ZMF-25 treatment (Fig. 10F and G). The EdU assay also demonstrated that the inhibition of autophagy can attenuate the inhibitory effect on proliferation induced by ZMF-25 (Fig. 10H). Wound healing assay showed that inhibition of autophagy could partially restore the migration ability of ZMF-25-treated MDA-MB-231 cells (Fig. 10I). Additionally, immunoblotting experiments demonstrated that ZMF-25 significantly enhances the up-regulation of E-cadherin, whereas the addition of 3-MA markedly impedes the increase of E-cadherin by ZMF-25 (Fig. 10J). In summary, ZMF-25-induced autophagy led to increased cell death and suppressed their migratory capacity in MDA-MB-231 cells.

### ZMF-25 shows good PK and therapeutic potential in vivo

Given the promising activity of ZMF-25 against TNBC, the PK properties were further evaluated. Upon intraperitoneal administration, ZMF-25 possessed an acceptable half-life of 1.4 h, a high exposure level (AUC_0-∞_ = 9,100.7 μg*h/l), as well as a moderate maximum plasma concentration (*C*_max_ = 5,113.1 μg/l) that was achieved in a short time (*T*_max_ = 0.5 h) (Table 4). Collectively, these findings revealed that ZMF-25 showed good PK properties.

**Table 4.** The PK parameters of ZMF-25 in Sprague–Dawley rats (*n* = 3, male)

Parameters	ZMF-25 (10 mg/kg, *n* = 3, intraperitoneally)
*T*_1/2_ (h)	1.4 ± 0.3
AUC(0-*t*) (μg*h/l)	9,071.8 ± 3,722.5
AUC(0-∞) (μg*h/l)	9,100.7 ± 3,745.2
*T*_max_ (h)	0.5 ± 0.2
CL/F (l/h/kg)	1.8 ± 0.9
V/F (l/kg)	3.2 ± 0.9
*C*_max_ (μg/l)	5,113.1 ± 1,555.8

**Fig. 11.**
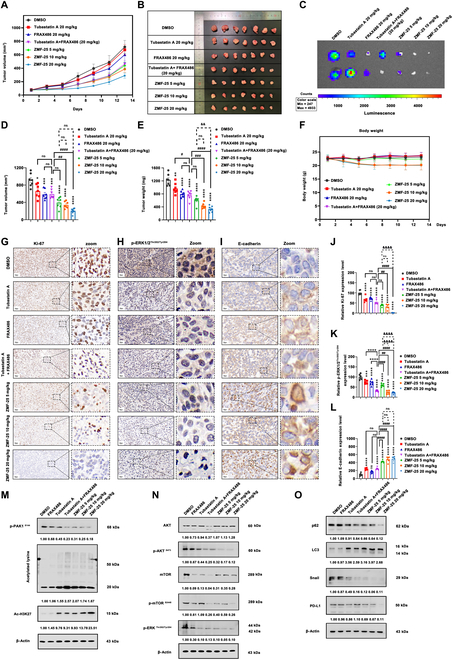
ZMF-25 inhibits TNBC in xenograft and nude mice. (A and B) ZMF-25 inhibits TNBC proliferation in xenograft and nude mice. (C) In vivo imaging was performed to detect the fluorescence intensity of each group of tumors on the day of dissection. (D and E) The tumor volume and tumor weight of each group were measured on the day of anatomy. Data were shown as mean ± SEM. *n* = 7. *P < 0.05, **P < 0.01, ****P < 0.0001, ^##^*P* < 0.01, ^###^*P* < 0.001, ^####^*P* < 0.0001, ^&&^*P* < 0.01; ns, no significant difference. (F) Body weight of each group on each day of the experiment. (G to L) Immunohistochemistry was used to check the expression of Ki-67, p-ERK1/2^Thr202/Tyr204^, and E-cadherin. Data were shown as mean ± SEM. *n* = 3. ***P < 0.001, ****P < 0.0001, ^##^*P* < 0.01, ^####^*P* < 0.0001. ^&&&&^*P* < 0.0001. (M to O) Western blot to measure the expression of p-PAK1^S144^, p-AKT^S473^, p-mTOR^S2448^, LC3, Snail, and PD-L1 level in MDA-MB-231 xenograft tumor.

Next, the therapeutic effects of ZMF-25 in vivo were further evaluated by an MDA-MB-231 xenograft nude mouse model. The inhibition of tumor growth increased progressively with prolonged ZMF-25 treatment (Fig. 11A and B). Statistical analysis of tumor volume and weight demonstrated that ZMF-25 inhibited tumor growth in a dose-dependent manner (Fig. 11D and E). Additionally, ZMF-25 exhibited superior inhibitory effects on tumor growth compared to both tubastatin A and FRAX486 when used individually, as well as when they were used in combination. ZMF-25 affected little the body weight of the mice, suggesting a low toxicity (Fig. 11F). Furthermore, the therapeutic potential of ZMF-25 was confirmed by a subcutaneous inoculation model with luciferase-labeled MDA-MB-231 cells in vivo (Fig. 11C). In addition, no obvious pathological changes were detected in the heart, liver, spleen, lungs, and kidneys of the mice after ZMF-25 treatment by hematoxylin and eosin (H&E) staining, indicating that ZMF-25 had no toxic effects on the organs of the mice (Fig. S3).

Next, the protein expression of Ki-67, p-ERK1/2^Thr202/Tyr204^, and E-cadherin in the tissues was detected by immunohistochemistry (Fig. 11G to L), showing that ZMF-25 could inhibit the expression of Ki-67, which was superior to those of tubastatin A and FRAX486 alone and concomitantly in 10 and 20 mg/kg dosages, while it was not significant in 5 mg/kg dosage. ZMF-25 also promoted E-cadherin expression in a dose-dependent manner, which was superior to that of tubastatin A and FRAX486 alone and concomitantly. The detection of p-ERK1/2^Thr202/Tyr204^ expression indicated that ZMF-25 could inhibit ERK activation. Subsequently, the analysis of p-PAK1^S144^ and total lysine acetylation levels demonstrated that ZMF-25 suppressed tumor growth by inhibiting PAK1 and HDAC class IIb. Additionally, key proteins in the AKT-mTOR and ERK1/2 pathways and PD-L1 further confirmed that ZMF-25 inhibited tumor growth by blocking PAK1 and HDAC class IIb-mediated cell growth mechanisms (Fig. 11M to O).

**Fig. 12.**
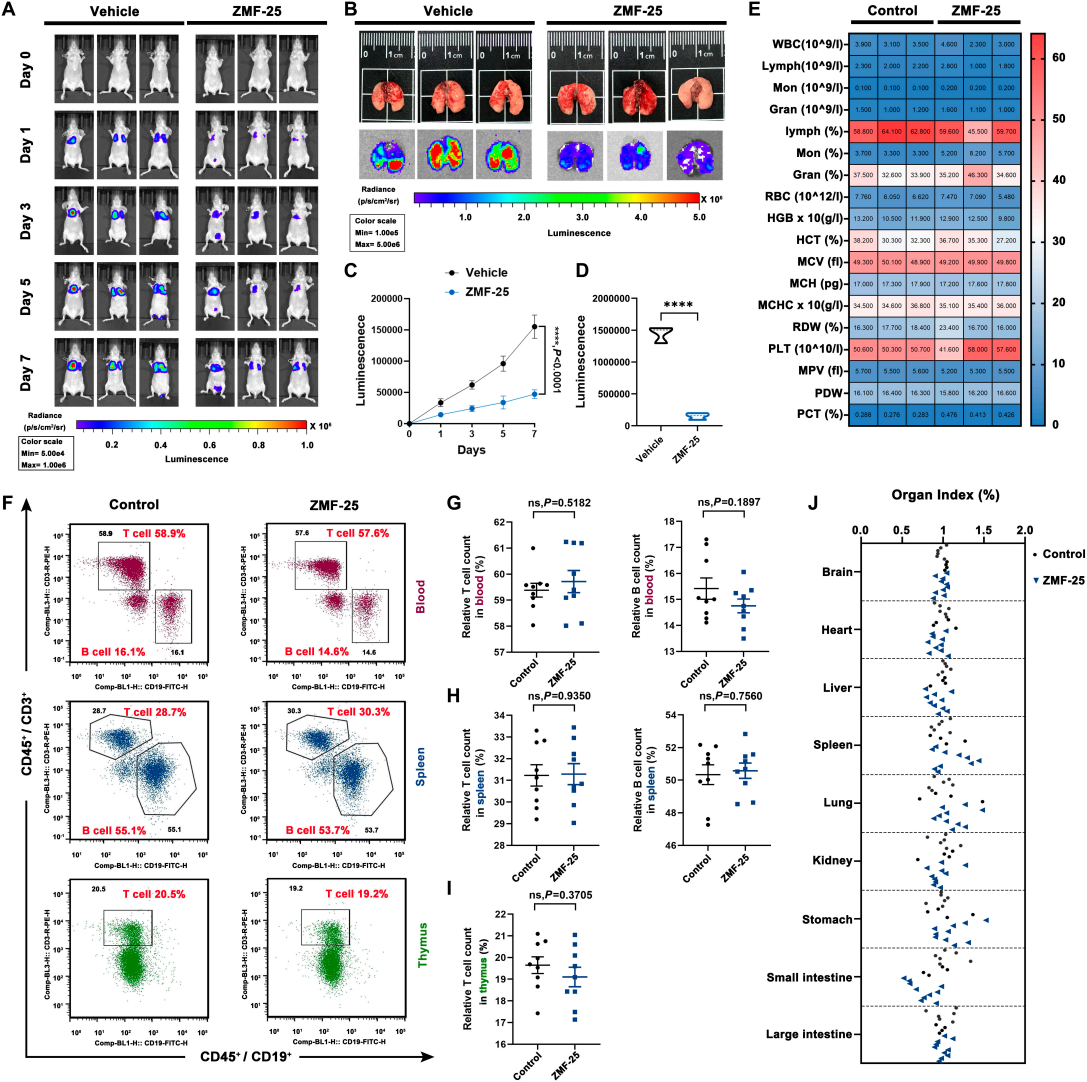
ZMF-25 inhibits TNBC metastasis and shows safety in vivo. (A and C) ZMF-25 inhibits TNBC metastasis in xenograft and nude mice. (B and D) In vivo imaging was performed to detect the fluorescence intensity of TNBC cell metastasis to lungs in every group on the day of dissection. (E) Effect of ZMF-25 treatment on blood routine in mice. The tumor volume and tumor weight of different groups were measured on the day of anatomy. (F to I) Effect of ZMF-25 treatment on T cell and B cell ratios in blood, spleen, and thymus of mice, respectively. Data were shown as mean ± SEM. *n* = 9. (J) The effects of ZMF-25 on brain, heart, liver, spleen, lung, kidney, stomach, small intestine, and large intestine were calculated. Data were shown as mean ± SEM. *n* = 10.

To further investigate the effect of ZMF-25 on TNBC cell metastasis, a tail vein injection model using MDA-MB-231-Luc cells was conducted to evaluate metastasis of TNBC in vivo. A significant reduction in lung metastasis of TNBC cells was observed in the ZMF-25 treatment group following euthanasia of the mice on day 7, confirming that ZMF-25 could significantly inhibit TNBC cell metastasis (Fig. 12A to D). Considering that antitumor drugs often cause immune suppression or gastrointestinal damage, we next investigated the potential toxicity of ZMF-25 in Balb/c mice. After administering 10 mg/kg of ZMF-25 for 12 d, we detected changes in the blood routine and the proportion of T cells and B cells in the blood, spleen, and thymus of mice. The results showed that there was no statistical difference between ZMF-25 treatment group and the control group (Fig. 12E to I). Furthermore, H&E staining was employed to examine the morphological changes in the major organs of mice. In comparison with nude mice, we included an additional evaluation of the brain, stomach, and small intestine. The findings indicated that ZMF-25 exhibited a relatively high safety profile, with no significant differences observed across all examined organs relative to the control group (Fig. S4). Accordingly, there was no significant difference in organ index (Fig. 12J).

## Discussion

TNBC is a subtype of breast cancer characterized by high incidence and poor prognosis, posing significant clinical challenges. Compared to other subtypes, TNBC demonstrates a more aggressive clinical course, earlier onset age, higher metastatic potential, less favorable clinical outcomes, increased recurrence rates, and lower survival rates [58,59]. Currently, surgery and chemotherapy remain the primary treatment modalities for TNBC, and drugs such as anthracycline, paclitaxel, platinum drugs, gemcitabine, capecitabine, and other drugs have performed well in the chemotherapy treatment of TNBC. However, these drugs are easy to develop resistance, and the increase in dose is also easy to cause a series of organ toxicity [60]. To overcome these shortcomings, new therapeutic modalities such as targeted therapy, immunotherapy, and other new therapeutic modalities are still being explored and developed. Targeted therapies hold significant potential for the treatment of TNBC. Agents such as poly ADP-ribose polymerase (PARP) inhibitors, PI3K inhibitors, androgen receptor inhibitors, cyclin-dependent kinase (CDK) inhibitors, epidermal growth factor receptor (EGFR) signaling pathway inhibitors, fibroblast growth factor receptor (FGFR) inhibitors, and vascular endothelial growth factor receptor (VEGFR) inhibitors have demonstrated promising therapeutic effects in breast cancer management [61]. Among these, PARP inhibitors have demonstrated the highest efficacy in the study of targeted therapies. While PARP inhibitors are effective against TNBC, they exhibit clinically significant resistance that cannot be overlooked. Their therapeutic efficacy is primarily observed in *BRCA1/2*-mutated TNBC, with limited effectiveness in other subtypes of TNBC [62]. Other inhibitors, such as AKT inhibitors and androgen receptor (AR) inhibitors, have shown certain therapeutic effects on advanced TNBC, but most of them have been terminated in clinical studies, and the specific mechanism of action needs to be further improved. Other targeted therapeutic drugs that are effective on TNBC are also mostly in clinical studies [63]. Hence, there is an urgent need to identify novel targeted therapeutic strategies for TNBC.

PAK1 is an important cancer-promoting protein that regulates several important cancer proliferation and metastasis pathways, and strongly contributes to metabolic reprogramming and malignant metastasis of cancer [17]. HDAC6 and HDAC10, which belong to class IIb HDAC, can regulate cell proliferation, malignant progression, and metastasis through their epigenetic function [64]. In our study, we found for the first time that simultaneous targeted inhibition of PAK1/HDAC6/HDAC10 has strong TNBC therapeutic potential. Applying computer-aided drug design strategies to develop promising disease treatment drugs is an important approach to drug discovery [65]. Here, utilizing structure-based screening and pharmacophore integration strategy, we have obtained a novel dual-target PAK1/HDAC IIb inhibitor with strong binding and enzyme inhibition effects on PAK1 and HDAC6/10. Importantly, it is possible to inhibit the proliferation and metastasis of TNBC by inhibiting PAK1/HDAC IIb in vitro and in vivo*.* Interestingly, ZMF-25 facilitated the generation and accumulation of ROS by inhibiting PAK/HDAC6/10, thereby disrupting mitochondrial function, leading to a substantial decrease in ATP production and a marked decline in mitochondrial membrane potential. Furthermore, our findings indicate that ROS play a crucial role in mediating ZMF-25-induced TNBC cell death. Notably, the anticancer efficacy of ZMF-25 was markedly diminished upon ROS inhibition. It is important to highlight that ROS, as a critical cellular metabolite, exerts concentration-dependent effects on carcinogenesis, tumor progression, and cancer therapy. Induction of ROS production has been demonstrated to enhance the therapeutic efficacy in TNBC treatment [2,66,67]. In addition, ZMF-25 has been demonstrated to inhibit the proliferation of TNBC cells and promote autophagy by suppressing the AKT-mTOR signaling pathway. This pathway not only is co-regulated by PAK1/HDAC6/HDAC10 in cell proliferation and metastasis but also plays a crucial role in the regulation of autophagy. Notably, targeting autophagy represents an emerging and promising strategy for the treatment of TNBC [68]. By comparing the basal autophagy levels of several TNBC cells and the anti-proliferation effects of ZMF-25 on these cells, we found that TNBC cells with lower basal autophagy levels appear to exhibit a more pronounced response to ZMF-25 (Fig. S5). This is interesting and illustrates the potential of induced autophagy in TNBC therapy. Importantly, ZMF-25 synergistically targets tumor energy metabolism, polyamine regulation, epigenetic regulation, and immunity. After ZMF-25 treatment, PAK1 suppression reduces glycolysis and HDAC6/10 inhibition down-regulates glycolysis gene expression and disrupts mitochondrial metabolism/DNA repair, amplifying metabolic stress. This multi-pathway intervention may constrain metabolic adaptability in high-metabolism tumors. ZMF-25 may reverse epigenetic dysregulation to restore differentiation and improve treatment sensitivity in epigenetically altered cancers. Reshaping the immunosuppressive microenvironment could enhance PD-1/PD-L1 efficacy in “cold” tumors while addressing resistance from metabolic plasticity and epigenetic heterogeneity. Safety challenges encompass neurotoxicity (HDAC6), hematopoietic risks (HDAC10), and central nervous system effects (PAK1). While our current findings indicate that ZMF-25 does not exhibit substantial side effects, the long-term safety of this treatment warrants further investigation. Although promising for refractory cancers, successful clinical translation necessitates optimized mechanistic synergy, stringent toxicity management, and precise patient stratification.

In summary, we have designed and synthesized a novel class of pyrido[2,3-d] pyrimidin-7(8*H*)-one-coupled-hydroxamic acid-containing compounds to develop potent dual-targeting PAK1/HDAC IIb inhibitors for TNBC treatment. These efforts have led to the discovery of ZMF-25, which presents low nanomolar inhibitory potency against PAK1/HDAC6/10 and demonstrates good isoform selectivity over PAKs and HDACs. ZMF-25 achieves excellent anti-proliferation and anti-migration potency by inhibiting both PAK1 and HDAC6/10 in vitro and in vivo. ZMF-25 is a potent and selective inhibitor of PAK1, as well as HDAC6 and HDAC10, exhibiting favorable PK properties with no obvious toxicity. This compound holds promise as a novel therapeutic strategy for the treatment of TNBC.

## Materials and Methods

Information about the Materials and Methods used in this article are provided in the Supplementary Materials.

## Data Availability

All data associated with this study are present in the paper or the Supplementary Materials.

## References

[1] Bianchini G, Balko JM, Mayer IA, Sanders ME, Gianni L. Triple-negative breast cancer: Challenges and opportunities of a heterogeneous disease. Nat Rev Clin Oncol. 2016;13(11):674–690. 27184417 10.1038/nrclinonc.2016.66 PMC5461122

[2] Zhang Y, Feng G, He T, Yang M, Lin J, Huang P. Traceable lactate-fueled self-acting photodynamic therapy against triple-negative breast cancer. Research. 2024;7:0277. 10.34133/research.0277 PMC12326368 40771576

[3] Zhou Y, Yu M, Tie C, Deng Y, Wang J, Yi Y, Zhang F, Huang C, Zheng H, Mei L, et al. Tumor microenvironment-specific chemical internalization for enhanced gene therapy of metastatic breast cancer. Research. 2021;2021:9760398. 38617380 10.34133/2021/9760398 PMC11014676

[4] Emens LA. Breast cancer immunotherapy: Facts and hopes. Clin Cancer Res. 2018;24(3):511–520. 28801472 10.1158/1078-0432.CCR-16-3001 PMC5796849

[5] Bianchini G, De Angelis C, Licata L, Gianni L. Treatment landscape of triple-negative breast cancer—Expanded options, evolving needs. Nat Rev Clin Oncol. 2022;19(2):91–113. 34754128 10.1038/s41571-021-00565-2

[6] Gong Y, Ji P, Yang Y-S, Xie S, Yu T-J, Xiao Y, Jin M-L, Ma D, Guo L-W, Pei Y-C, et al. Metabolic-pathway-based subtyping of triple-negative breast cancer reveals potential therapeutic targets. Cell Metab. 2021;33(1):51–64.e9. 33181091 10.1016/j.cmet.2020.10.012

[7] Xiao Y, Ma D, Yang Y-S, Yang F, Ding J-H, Gong Y, Jiang L, Ge L-P, Wu S-Y, Yu Q, et al. Comprehensive metabolomics expands precision medicine for triple-negative breast cancer. Cell Res. 2022;32(5):477–490. 35105939 10.1038/s41422-022-00614-0 PMC9061756

[8] Zhang H, Zhai X, Liu Y, Xia Z, Xia T, Du G, Zhou H, Franziska Strohmer D, Bazhin AV, Li Z, et al. NOP2-mediated m5C modification of c-Myc in an EIF3A-dependent manner to reprogram glucose metabolism and promote hepatocellular carcinoma progression. Research. 2023;6:0184. 37398932 10.34133/research.0184 PMC10313139

[9] Jia H, Truica CI, Wang B, Wang Y, Ren X, Harvey HA, Song J, Yang J-M. Immunotherapy for triple-negative breast cancer: Existing challenges and exciting prospects. Drug Resist Updat. 2017;32:1–15. 29145974 10.1016/j.drup.2017.07.002

[10] Costa A, Kieffer Y, Scholer-Dahirel A, Pelon F, Bourachot B, Cardon M, Sirven P, Magagna I, Fuhrmann L, Bernard C, et al. Fibroblast heterogeneity and immunosuppressive environment in human breast cancer. Cancer Cell. 2018;33(3):463–479.e10. 29455927 10.1016/j.ccell.2018.01.011

[11] So JY, Ohm J, Lipkowitz S, Yang L. Triple negative breast cancer (TNBC): Non-genetic tumor heterogeneity and immune microenvironment: Emerging treatment options. Pharmacol Ther. 2022;237: Article 108253. 35872332 10.1016/j.pharmthera.2022.108253 PMC9378710

[12] Shu S, Wu H-J, Ge JY, Zeid R, Harris IS, Jovanović B, Murphy K, Wang B, Qiu X, Endress JE, et al. Synthetic lethal and resistance interactions with BET bromodomain inhibitors in triple-negative breast cancer. Mol Cell. 2020;78(6):1096–1113.e8. 32416067 10.1016/j.molcel.2020.04.027 PMC7306005

[13] Zhang K, Liu Z, Yao Y, Qiu Y, Li F, Chen D, Hamilton DJ, Li Z, Jiang S. Structure-based design of a selective class I histone deacetylase (HDAC) near-infrared (NIR) probe for epigenetic regulation detection in triple-negative breast cancer (TNBC). J Med Chem. 2021;64(7):4020–4033. 33745280 10.1021/acs.jmedchem.0c02161

[14] Huang X, Ma Y, Ma G, Xia Y. Unlocking the therapeutic applicability of LNP-mRNA: Chemistry, formulation, and clinical strategies. Research. 2024;7:0370. 38894715 10.34133/research.0370 PMC11185168

[15] Wu J, Liu N, Chen J, Tao Q, Li Q, Li J, Chen X, Peng C. The tricarboxylic acid cycle metabolites for cancer: Friend or enemy. Research. 2024;7:0351. 38867720 10.34133/research.0351 PMC11168306

[16] Lu D, Li Y, Niu X, Sun J, Zhan W, Shi Y, Yu K, Huang S, Liu X, Xie L, et al. STAT2/SLC27A3/PINK1-mediated mitophagy remodeling lipid metabolism contributes to pazopanib resistance in clear cell renal cell carcinoma. Research. 2024;7:0539. 39600540 10.34133/research.0539 PMC11588985

[17] Yao D, Li C, Rajoka MSR, He Z, Huang J, Wang J, Zhang J. P21-activated kinase 1: Emerging biological functions and potential therapeutic targets in cancer. Theranostics. 2020;10(21):9741–9766. 32863957 10.7150/thno.46913 PMC7449905

[18] Eswaran J, Li D-Q, Shah A, Kumar R. Molecular pathways: Targeting p21-activated kinase 1 signaling in cancer—Opportunities, challenges, and limitations. Clin Cancer Res. 2012;18(14):3743–3749. 22595609 10.1158/1078-0432.CCR-11-1952 PMC3399091

[19] Feng X, Zhang H, Meng L, Song H, Zhou Q, Qu C, Zhao P, Li Q, Zou C, Liu X, et al. Hypoxia-induced acetylation of PAK1 enhances autophagy and promotes brain tumorigenesis via phosphorylating ATG5. Autophagy. 2021;17(3):723–742. 32186433 10.1080/15548627.2020.1731266 PMC8032228

[20] Chow HY, Jubb AM, Koch JN, Jaffer ZM, Stepanova D, Campbell DA, Duron SG, O’Farrell M, Cai KQ, Klein-Szanto AJP, et al. p21-activated kinase 1 is required for efficient tumor formation and progression in a Ras-mediated skin cancer model. Cancer Res. 2012;72(22):5966–5975. 22983922 10.1158/0008-5472.CAN-12-2246 PMC3500416

[21] Crawford JJ, Lee W, Aliagas I, Mathieu S, Hoeflich KP, Zhou W, Wang W, Rouge L, Murray L, La H, et al. Structure-guided design of group I selective p21-activated kinase inhibitors. J Med Chem. 2015;58(12):5121–5136. 26030457 10.1021/acs.jmedchem.5b00572

[22] Murray BW, Guo C, Piraino J, Westwick JK, Zhang C, Lamerdin J, Dagostino E, Knighton D, Loi C-M, Zager M, et al. Small-molecule p21-activated kinase inhibitor PF-3758309 is a potent inhibitor of oncogenic signaling and tumor growth. Proc Natl Acad Sci USA. 2010;107(20):9446–9451. 20439741 10.1073/pnas.0911863107 PMC2889050

[23] Licciulli S, Maksimoska J, Zhou C, Troutman S, Kota S, Liu Q, Duron S, Campbell D, Chernoff J, Field J, et al. FRAX597, a small molecule inhibitor of the p21-activated kinases, inhibits tumorigenesis of neurofibromatosis type 2(NF2)-associated Schwannomas. J Biol Chem. 2013;288(40):29105–29114. 23960073 10.1074/jbc.M113.510933 PMC3790009

[24] McCoull W, Hennessy EJ, Blades K, Chuaqui C, Dowling JE, Ferguson AD, Goldberg FW, Howe N, Jones CR, Kemmitt PD, et al. Optimization of highly kinase selective bis-anilino pyrimidine PAK1 inhibitors. ACS Med Chem Lett. 2016;7(12):1118–1123. 27994749 10.1021/acsmedchemlett.6b00322 PMC5150691

[25] Yao D, Huang J, Wang J, He Z, Zhang J. Design, synthesis and biological evaluation of novel tetrahydrothieno [2,3-c]pyridine substitued benzoyl thiourea derivatives as PAK1 inhibitors in triple negative breast cancer. J Enzyme Inhib Med Chem. 2020;35(1):1524–1538. 32752894 10.1080/14756366.2020.1797710 PMC7470115

[26] Yao D, Ruhan A, Jiang J, Huang J, Wang J, Han W. Design, synthesis and biological evaluation of 2-indolinone derivatives as PAK1 inhibitors in MDA-MB-231 cells. Bioorg Med Chem Lett. 2020;30(17): Article 127355. 32738980 10.1016/j.bmcl.2020.127355

[27] Zhang J, Zou L, Tang P, Pan D, He Z, Yao D. Design, synthesis and biological evaluation of 1H-pyrazolo [3,4-d]pyrimidine derivatives as PAK1 inhibitors that trigger apoptosis, ER stress and anti-migration effect in MDA-MB-231 cells. Eur J Med Chem. 2020;194: Article 112220. 32222676 10.1016/j.ejmech.2020.112220

[28] Rudolph J, Murray LJ, Ndubaku CO, O’Brien T, Blackwood E, Wang W, Aliagas I, Gazzard L, Crawford JJ, Drobnick J, et al. Chemically diverse group I p21-activated kinase(PAK) inhibitors impart acute cardiovascular toxicity with a narrow therapeutic window. J Med Chem. 2016;59(11):5520–5541. 27167326 10.1021/acs.jmedchem.6b00638

[29] Radu M, Semenova G, Kosoff R, Chernoff J. PAK signalling during the development and progression of cancer. Nat Rev Cancer. 2014;14(1):13–25. 24505617 10.1038/nrc3645 PMC4115244

[30] Rahhal R, Seto E. Emerging roles of histone modifications and HDACs in RNA splicing. Nucleic Acids Res. 2019;47(10):4911–4926. 31162605 10.1093/nar/gkz292 PMC6547430

[31] Buchwald M, Krämer OH, Heinzel T. HDACi—Targets beyond chromatin. Cancer Lett. 2009;280(2):160–167. 19342155 10.1016/j.canlet.2009.02.028

[32] Varricchio L, Dell’Aversana C, Nebbioso A, Migliaccio G, Altucci L, Mai A, Grazzini G, Bieker JJ, Migliaccio AR. Identification of NuRSERY, a new functional HDAC complex composed by HDAC5, GATA1, EKLF and pERK present in human erythroid cells. Int J Biochem Cell Biol. 2014;50:112–122. 24594363 10.1016/j.biocel.2014.02.019 PMC4003889

[33] Balasubramanian S, Verner E, Buggy JJ. Isoform-specific histone deacetylase inhibitors: The next step? Cancer Lett. 2009;280(2):211–221. 19289255 10.1016/j.canlet.2009.02.013

[34] Liang T, Wang F, Elhassan RM, Cheng Y, Tang X, Chen W, Fang H, Hou X. Targeting histone deacetylases for cancer therapy: Trends and challenges. Acta Pharm Sin B. 2023;13(6):2425–2463. 37425042 10.1016/j.apsb.2023.02.007 PMC10326266

[35] Ouyang H, Ali YO, Ravichandran M, Dong A, Qiu W, MacKenzie F, Dhe-Paganon S, Arrowsmith CH, Zhai RG. Protein aggregates are recruited to aggresome by histone deacetylase 6 via unanchored ubiquitin C termini. J Biol Chem. 2012;287(4):2317–2327. 22069321 10.1074/jbc.M111.273730 PMC3268394

[36] Li T, Zhang C, Hassan S, Liu X, Song F, Chen K, Zhang W, Yang J. Histone deacetylase 6 in cancer. J Hematol Oncol. 2018;11(1):111. 30176876 10.1186/s13045-018-0654-9 PMC6122547

[37] Hai Y, Shinsky SA, Porter NJ, Christianson DW. Histone deacetylase 10 structure and molecular function as a polyamine deacetylase. Nat Commun. 2017;8:15368. 28516954 10.1038/ncomms15368 PMC5454378

[38] Stewart TM, Foley JR, Holbert CE, Klinke G, Poschet G, Steimbach RR, Miller AK, Casero RA. Histone deacetylase-10 liberates spermidine to support polyamine homeostasis and tumor cell growth. J Biol Chem. 2022;298(10): Article 102407. 35988653 10.1016/j.jbc.2022.102407 PMC9486564

[39] Cheng F, Zheng B, Wang J, Zhao G, Yao Z, Niu Z, He W. Histone deacetylase 10, a potential epigenetic target for therapy. Biosci Rep. 2021;41(6):BSR20210462. 33997894 10.1042/BSR20210462 PMC8182986

[40] Oehme I, Linke J-P, Böck BC, Milde T, Lodrini M, Hartenstein B, Wiegand I, Eckert C, Roth W, Kool M, et al. Histone deacetylase 10 promotes autophagy-mediated cell survival. Proc Natl Acad Sci USA. 2013;110(28):E2592–E2601. 23801752 10.1073/pnas.1300113110 PMC3710791

[41] Liu X, Wang Y, Zhang R, Jin T, Qu L, Jin Q, Zheng J, Sun J, Wu Z, Wang L, et al. HDAC10 is positively associated with PD-L1 expression and poor prognosis in patients with NSCLC. Front Oncol. 2020;10:485. 32373519 10.3389/fonc.2020.00485 PMC7186423

[42] Shen S, Svoboda M, Zhang G, Cavasin MA, Motlova L, McKinsey TA, Eubanks JH, Bařinka C, Kozikowski AP. Structural and in vivo characterization of tubastatin A, a widely used histone deacetylase 6 inhibitor. ACS Med Chem Lett. 2020;11(5):706–712. 32435374 10.1021/acsmedchemlett.9b00560 PMC7236036

[43] Steimbach RR, Herbst-Gervasoni CJ, Lechner S, Stewart TM, Klinke G, Ridinger J, Géraldy MNE, Tihanyi G, Foley JR, Uhrig U, et al. Aza-SAHA derivatives are selective histone deacetylase 10 chemical probes that inhibit polyamine deacetylation and phenocopy HDAC10 knockout. J Am Chem Soc. 2022;144(41):18861–18875. 36200994 10.1021/jacs.2c05030 PMC9588710

[44] Yang EG, Mustafa N, Tan EC, Poulsen A, Ramanujulu PM, Chng WJ, Yen JJY, Dymock BW. Design and synthesis of Janus kinase 2 (JAK2) and histone deacetlyase (HDAC) bispecific inhibitors based on pacritinib and evidence of dual pathway inhibition in hematological cell lines. J Med Chem. 2016;59(18):8233–8262. 27541357 10.1021/acs.jmedchem.6b00157

[45] Dolan BM, Duron SG, Campbell DA, Vollrath B, Shankaranarayana Rao BS, Ko H-Y, Lin GG, Govindarajan A, Choi S-Y, Tonegawa S. Rescue of fragile X syndrome phenotypes in Fmr1 KO mice by the small-molecule PAK inhibitor FRAX486. Proc Natl Acad Sci USA. 2013;110(14):5671–5676. 23509247 10.1073/pnas.1219383110 PMC3619302

[46] Butler KV, Kalin J, Brochier C, Vistoli G, Langley B, Kozikowski AP. Rational design and simple chemistry yield a superior, neuroprotective HDAC6 inhibitor, tubastatin A. J Am Chem Soc. 2010;132(31):10842–10846. 20614936 10.1021/ja102758v PMC2916045

[47] Zhang J, Chen X, Chen G, Wang H, Jia L, Hao Y, Yao D. Identification of a novel PAK1/HDAC6 dual inhibitor ZMF-23 that triggers tubulin-stathmin regulated cell death in triple negative breast cancer. Int J Biol Macromol. 2023;251: Article 126348. 37586623 10.1016/j.ijbiomac.2023.126348

[48] Nielsen TO, Leung SCY, Rimm DL, Dodson A, Acs B, Badve S, Denkert C, Ellis MJ, Fineberg S, Flowers M, et al. Assessment of Ki67 in breast cancer: Updated recommendations from the International Ki67 in Breast Cancer Working Group. J Natl Cancer Inst. 2021;113(7):808–819. 33369635 10.1093/jnci/djaa201 PMC8487652

[49] Angelozzi M, de Charleroy CR, Lefebvre V. EdU-based assay of cell proliferation and stem cell quiescence in skeletal tissue sections. Methods Mol Biol. 2021;2230:357–365. 33197025 10.1007/978-1-0716-1028-2_21 PMC11783623

[50] Xie W, Jiang Q, Wu X, Wang L, Gao B, Sun Z, Zhang X, Bu L, Lin Y, Huang Q, et al. IKBKE phosphorylates and stabilizes snail to promote breast cancer invasion and metastasis. Cell Death Differ. 2022;29(8):1528–1540. 35066576 10.1038/s41418-022-00940-1 PMC9345937

[51] Padmanaban V, Krol I, Suhail Y, Szczerba BM, Aceto N, Bader JS, Ewald AJ. E-cadherin is required for metastasis in multiple models of breast cancer. Nature. 2019;573(7774):439–444. 31485072 10.1038/s41586-019-1526-3 PMC7365572

[52] Ahn M, Oh E, McCown EM, Wang X, Veluthakal R, Thurmond DC. A requirement for PAK1 to support mitochondrial function and maintain cellular redox balance via electron transport chain proteins to prevent β-cell apoptosis. Metabolism. 2021;115: Article 154431. 33181191 10.1016/j.metabol.2020.154431 PMC8123936

[53] DeSantiago J, Bare DJ, Xiao L, Ke Y, Solaro RJ, Banach K. p21-activated kinase1 (Pak1) is a negative regulator of NADPH-oxidase 2 in ventricular myocytes. J Mol Cell Cardiol. 2014;67:77–85. 24380729 10.1016/j.yjmcc.2013.12.017 PMC3930036

[54] Chen Y, Gibson SB. Is mitochondrial generation of reactive oxygen species a trigger for autophagy? Autophagy. 2008;4(2):246–248. 18094624 10.4161/auto.5432

[55] Kim J, Kundu M, Viollet B, Guan K-L. AMPK and mTOR regulate autophagy through direct phosphorylation of Ulk1. Nat Cell Biol. 2011;13(2):132–141. 21258367 10.1038/ncb2152 PMC3987946

[56] Zachari M, Ganley IG. The mammalian ULK1 complex and autophagy initiation. Essays Biochem. 2017;61(6):585–596. 29233870 10.1042/EBC20170021 PMC5869855

[57] Nunnery SE, Mayer IA. Targeting the PI3K/AKT/mTOR pathway in hormone-positive breast cancer. Drugs. 2020;80(16):1685–1697. 32894420 10.1007/s40265-020-01394-w PMC7572750

[58] Tarantino P, Antonarelli G, Ascione L, Curigliano G. Investigational immunomodulatory drugs for enhancement of triple negative breast cancer(TNBC) immunotherapy: Early phase development. Expert Opin Investig Drugs. 2022;31(6):499–513. 10.1080/13543784.2021.1972968 34569405

[59] Garrido-Castro AC, Lin NU, Polyak K. Insights into molecular classifications of triple-negative breast cancer: Improving patient selection for treatment. Cancer Discov. 2019;9(2):176–198. 30679171 10.1158/2159-8290.CD-18-1177 PMC6387871

[60] Sharma P, Stecklein SR, Yoder R, Staley JM, Schwensen K, O’Dea A, Nye L, Satelli D, Crane G, Madan R, et al. Clinical and biomarker findings of neoadjuvant pembrolizumab and carboplatin plus docetaxel in triple-negative breast cancer: NeoPACT phase 2 clinical trial. JAMA Oncol. 2024;10(2):227–235. 37991778 10.1001/jamaoncol.2023.5033 PMC10666040

[61] Zhu S, Wu Y, Song B, Yi M, Yan Y, Mei Q, Wu K. Recent advances in targeted strategies for triple-negative breast cancer. J Hematol Oncol. 2023;16(1):100. 37641116 10.1186/s13045-023-01497-3 PMC10464091

[62] Shome R, Ghosh SS. Tweaking EMT and MDR dynamics to constrain triple-negative breast cancer invasiveness by EGFR and Wnt/β-catenin signaling regulation. Cell Oncol (Dordr). 2021;44(2):405–422. 33398673 10.1007/s13402-020-00576-8 PMC12980752

[63] Li Y, Zhang H, Merkher Y, Chen L, Liu N, Leonov S, Chen Y. Recent advances in therapeutic strategies for triple-negative breast cancer. J Hematol Oncol. 2022;15(1):121. 36038913 10.1186/s13045-022-01341-0 PMC9422136

[64] Zhong Y, Huang T, Huang J, Quan J, Su G, Xiong Z, Lv Y, Li S, Lai X, Xiang Y, et al. The HDAC10 instructs macrophage M2 program via deacetylation of STAT3 and promotes allergic airway inflammation. Theranostics. 2023;13(11):3568–3581. 37441601 10.7150/thno.82535 PMC10334828

[65] Yu Y, Wang Z, Wang L, Wang Q, Tang R, Xiang S, Deng Q, Hou T, Sun H. Deciphering the shared and specific drug resistance mechanisms of anaplastic lymphoma kinase via binding free energy computation. Research. 2023;6:0170. 37342628 10.34133/research.0170 PMC10278961

[66] Hu J, Zhang Y, Jiang X, Zhang H, Gao Z, Li Y, Fu R, Li L, Li J, Cui H, et al. ROS-mediated activation and mitochondrial translocation of CaMKII contributes to Drp1-dependent mitochondrial fission and apoptosis in triple-negative breast cancer cells by isorhamnetin and chloroquine. J Exp Clin Cancer Res. 2019;38(1):225. 31138329 10.1186/s13046-019-1201-4 PMC6540563

[67] Kong F, He P, Jiang J, Zhu W, Lei Q. Spatiotemporally-controlled hydrophobic drug delivery via photosensitizer-driven assembly-disassembly for enhanced triple-negative breast cancer treatment. J Control Release. 2024;369:53–62. 38513728 10.1016/j.jconrel.2024.03.035

[68] Abd El-Aziz YS, Gillson J, Jansson PJ, Sahni S. Autophagy: A promising target for triple negative breast cancers. Pharmacol Res. 2022;175: Article 106006. 34843961 10.1016/j.phrs.2021.106006

