# Supplementary material for: Discovery of a Novel Selective PAK1/HDAC6/HDAC10 Inhibitor ZMF-25 that Induces Mitochondrial Metabolic Breakdown and Autophagy-Related Cell Death in Triple-Negative Breast Cancer
Source: Research (Wash D C). 2025 Apr 29;8:0670. doi: 10.34133/research.0670 (PMC12038163; doi:10.34133/research.0670)
Supplement: Supplementary 1 — Materials and Methods Figs. S1 to S9 Tables S1 and S2 [file research.0670.f1.zip › R1-Supplementary Material-0315 Clean Version.pdf]

Supporting Information for

Discovery of a Novel Selective PAK1/HDAC6/HDAC10 Inhibitor ZMF-25 that Induces Mitochondrial Metabolic Breakdown and Autophagy-Related Cell Death in Triple Negative Breast Cancer

Authors

Jin Zhang[1†], Xiaoling Cheng[1, 2†], Gang Chen,[1,2†], Xiya Chen[1,2], Xi Zhao[1,3], Weiji Chen[1,2,4], Wei Du[5], Zhendan He[1,2], Xiaojun Yao[4]*, Bo Han[3]*, Dahong Yao[2]*

Affiliations

[1]*School of Pharmaceutical Sciences, Health Science Center, Shenzhen University, Shenzhen 518060, China*

[2]*School of Pharmaceutical Sciences, Shenzhen Technology University, Shenzhen 518118, China*

[3]*State Key Laboratory of Southwestern Chinese Medicine Resources, Hospital of Chengdu University of Traditional Chinese Medicine, School of Pharmacy, Chengdu University of Traditional Chinese Medicine, Chengdu 611137, China.*

[4]*Centre for Artificial Intelligence Driven Drug Discovery, Faculty of Applied Sciences, Macao Polytechnic University, Macao 999078, China*

[5]*West China School of Pharmacy, Sichuan University, Chengdu 610000, China*

*Address correspondence to: hanbo@cdutcm.edu.cn (Bo Han); xjyao@mpu.edu.mo (Xiaojun Yao); yaodahong@sztu.edu.cn (Dahong Yao). Tel/Tax: +86-0755-86671989.

†These authors contributed equally to this work.

CONTENTS

Materials and Methods 3

Chemistry 3

Cell culture and regents 30

Molecular Docking 30

Molecular Dynamics (MD) Simulations 30

Binding-free Energy Calculation (MM-GBSA) 31

The Anzymatic Assay of HDACs 31

The Enzymatic Assay of PAKs 32

KinomeScan 32

SPR Assay 33

CETSA Assay 33

Cell Migration Assay 33

Plate Cloning Experiment 34

Cell Viability Assay 34

Flow Cytometry Analysis of 2-NBDG 34

Flow Cytometry Analysis of ROS 35

Flow Cytometry Analysis of Mitochondrial Membrane Potential 35

Immunofluorescence Analysis 35

Plasmid Transfection 35

Animal experiments 36

Western Blot 36

HE Staining 37

Immunohistochemistry Analysis (IHC) 37

Oxygen Consumption Rate 37

Extracellular Acidification Rate 38

N^8-acetylspermidine(N8-AcSpd) content detection 38

Statistical Analysis 40

Supplementary schemes, tables and figures 40

Figure S1 Structure of PAK1 and HDAC IIb Inhibitors 40

Figure S2 Effect of DFZK-748 and si-HDAC10 on cell viability of MDA-MB-231 cells. 40

Figure S3 HE staining of the heart, liver, spleen, lung and kidney of the mice in different groups. 41

Figure S4 Effect of ZMF-25 treatment on mice organs. 41

Figure S5 Effect of ZMF-25 on cell viability and the detection of basal autophagy level. 42

Table S1 CI values of FRAX486, Tubastatin A and DKFZ-748 for 24h 43

Table S2 CI values of FRAX486, Tubastatin A and DKFZ-748 for 48h 50

The original images of Western blots 57

^{1}H and ^{13}C NMR, HR-MS and HPLC Spectrum of Compounds 62

Materials and Methods

Chemistry

All reagents and solvents used in this study were obtained commercially without further purification. The ^{1}H-NMR spectra were recorded at either 400 or 600 MHz, while the ^{13}C-NMR data were collected at either 100 or 150 MHz with complete proton decoupling. ESI-MS spectra of all compounds were obtained using a TSQ Altis™ Plus instrument equipped with high-pressure liquid chromatography (Waters Acquity I-ClassTM). The purities of the compounds were determined to be above 95% through HPLC analysis. HPLC measurements were performed using an Agilent 1260 instrument with a Phenomenex Luna C18 column (5μm particle size, pore size:100A, dimensions:250x4.6mm; S/N:H19-293790). Elution was carried out using a mixture of methanol and water, and the flow rate was set at 1.0 mL/min. The purities of the compounds were determined by HPLC to be above 95%. HPLC instrument: Agilent 1260, Column: Phenomenex Luna 5μm C18 (2) 100A, 250x4.6mm S/N: H19-293790. Elution: MeOH in water; Flow rate: 1.0 mL/min.

*Synthesis of intermediate **11**. 4 - amino - 2 - (methylthio)pyrimidine - 5 - carbaldehyde (**11**)* To a solution of 4 - chloro - 2 - (methylthio)pyrimidine - 5 - carbaldehyde (**10**) (500 mg, 2.15 mmol) in anhydrous THF (12 mL) was added triethylamine (1.0 mL, 6.45 mmol) and ammonium hydroxide (2.5 mL). The resulting mixture was stirred at room temperature for about 2 h until the starting material was consumed completely. After evaporation to remove THF under reduced pressure, the residue was partitioned between water (30 mL) and EtOAc (30 mL). The organic layer was washed with water, brine, dried over anhydrous Na_2SO_4, filtered and concentrated *in vacuo*. The crude mixture was purified by column chromatography on silica gel (13-15% EtOAc/hexane) to afford intermediate **11** (440 mg, 97%) as a white solid. ^{1}H-NMR (400 MHz, DMSO-*d*6), δ(ppm): 9.77 (1H, s), 8.57 (1H, s), 8.28 (1H, s), 8.03 (1H, s), 2.50 (3H, s); ^{13}C-NMR (100 MHz, DMSO-*d*6), δ(ppm): 192.2, 176.1, 164.0, 160.4, 109.5, 14.0;

General Procedure for the Preparation of 13a−13e. To a solution of 4-amino-2-(methylthio)pyrimidine-5-carbaldehyde (**11**) (846.0 mg, 5.0 mmol) and potassium carbonate (2.07 g 15.0 mmol) in DMAc (12 mL) and Ethyl phenylacetate derivatives **12** (4.75 mmol, 0.95 eq). The mixture was heated at 120 °C and stirred for 10 h. The hot reaction mixture was poured onto ice and solids precipitated. The slurry was stirred for 1 h, filtered and the filter cake was washed with water, dried in vacuum at 55 °C

to afford intermediate **13a**-**13e** as white solid, yield 78-93%.

*2-(methylthio)-6-phenylpyrido[2,3-d]pyrimidin-7(8H)-one (**13a**)* ^{1}H-NMR (400 MHz, DMSO-d_6), δ(ppm): 12.58 (1H, s), 8.89 (1H, s), 8.09 (1H, s), 7.70 (2H, m), 7.37-7.46 (3H, m), 2.58 (3H, s); ^{13}C-NMR (100 MHz, DMSO-d_6), δ(ppm): 171.9, 162.6, 157.1, 154.2, 135.7, 134.9, 132.5, 129.0, 129.0, 128.7, 128.5, 128.5, 109.8, 14.1.

*6-(4-fluorophenyl)-2-(methylthio)pyrido[2,3-d]pyrimidin-7(8H)-one (**13b**)* ^{1}H-NMR (400 MHz, DMSO-d_6), δ(ppm): 12.60 (1H, s), 8.88 (1H, s), 8.10 (1H, s), 7.76 (2H, m), 7.28 (2H, m), 2.58 (3H, s); ^{13}C NMR (100 MHz, DMSO-d_6), δ(ppm): 171.9, 162.6, 157.1, 154.2, 135.7, 134.9, 132.5, 129.0, 129.0, 128.7, 128.5, 128.5, 109.8, 14.1.

6-(2,4-difluorophenyl)-2-(methylthio)pyrido[2,3-d]pyrimidin-7(8H)-one *(**13c**)* ^{1}H NMR (400 MHz, DMSO-d_6), δ(ppm): 12.66 (1H, s), 8.90 (1H, s), 8.04 (1H, s), 7.57 (1H, m), 7.34 (1H, m), 7.18 (1H, m), 2.59 (3H, s); ^{13}C NMR (100 MHz, DMSO-d_6), δ(ppm): 172.6, 164.0, 161.9, 159.0, 157.4 154.5, 137.3, 133.2, 127.6, 120.2, 111.7, 109.4, 104.6, 14.1.

*6-(2,4-dichlorophenyl)-2-(methylthio)pyrido[2,3-d]pyrimidin-7(8H)-one (**13d**)* ^{1}H-NMR (400 MHz, DMSO-d_6), δ(ppm): 12.68 (1H, s), 8.90 (1H, s), 7.98 (1H, s), 7.73 (1H, d, J = 2.0 Hz), 7.52 (1H, dd, J = 8.3, 2.0 Hz), 7.45 (1H, d, J = 8.3 Hz), 2.58 (3H, s); ^{13}C-NMR (100 MHz, DMSO-d_6), δ(ppm): 172.8, 161.7, 157.5, 154.7, 137.2, 134.4, 134.2, 134.2, 133.5, 131.1, 129.3, 127.7, 109.2, 14.2.

*6-(2-chloro-4-fluorophenyl)-2-(methylthio)pyrido[2,3-d]pyrimidin-7(8H)-one (**13e**)* ^{1}H-NMR (400 MHz, DMSO-d_6), δ(ppm): 12.67 (1H, s), 8.90 (1H, s), 7.96 (1H, s), 7.56 (1H, m), 7.48 (1H, m), 7.32 (1H, m), 2.59 (3H, s); ^{13}C-NMR (100 MHz, DMSO-d_6), δ(ppm): 172.7, 163.4, 161.9, 157.4, 154.7, 137.2, 134.4, 133.7, 131.8, 131.3, 116.9, 114.9, 109.2, 14.2;

*General Procedure for the Preparation of Intermediates **17-19**.* A mixture of **13** (1.0 mmol), 3-chloroperoxybenzoic acid (80%, 2.4 mmol) in dichloromethane (15 mL) was stirred at room temperature overnight. The reaction mixture was washed with saturated sodium bicarbonate solution (2×20 mL) and saturated sodium chloride solution (2×20 mL). The organic layer was dried over anhydrous magnesium sulfate, filtered and concentrated to afford crude *intermediate* as yellow solid for the next step without purification. To a solution of *intermediate* (2.0 mmol) in isopropanol (10 mL) was added amine derivatives (40.0 mmol, 20 eq). The residue was allowed to heat overnight at 90 ºC the material was consumed completely. The reaction mixture was concentrated and purified by silica gel chromatography eluting with (DCM/MeOH=100/1-30/1) to afford *intermediates* **14** (**15** or **16**) as white solid, yield 66-

75%.

To a solution of intermediates **14** (**15** or **16**) (1.0 mmol) in DMF (8.0 ml), K_2CO_3 (2.0 mmol), halogenated carboxylic acid esters, and catalytic amount potassium iodide were added, and the resulting mixture was allowed to heat for 6 hours at 80 °C. Upon the starting material was consumed completely, the hot reaction suspension was poured into water (30 mL), extracted with dichloromethane (3 × 30 mL). The combined organic layers were washed with saturated aqueous sodium bicarbonate and brine, and then dried over anhydrous sodium sulfate. After removing the solvent under reduced pressure to give the crude mixture, and purified by silica sel flash chromatography (dichloromethane/methanol 50 to 30:1) as white solid, yield 58-74%.

Ethyl 4-(2-(methylamino)-7-oxo-6-phenylpyrido[2,3-d]pyrimidin-8(7H)-yl)butanoate ***(17a)*** ^{1}H-NMR (400 MHz, DMSO-d_6), δ(ppm): 8.63 (1H, s), 7.92 (1H, s), 7.81 (1H, d, J = 4.5 Hz), 7.66 (2H, m), 7.41 (2H, m), 7.34 (1H, m), 4.39 (2H, t, J = 6.6 Hz), 3.97 (2H, q, J = 7.1 Hz), 2.91 (3H, d, J = 4.5 Hz), 2.38 (2H, t, J = 7.0 Hz), 2.00 (2H, m), 1.12 (3H, t, J = 7.1 Hz); ^{13}C-NMR (100 MHz, DMSO-d_6), δ(ppm): 172.8, 162.2, 159.8, 155.2, 137.0, 135.1, 129.0, 129.0, 128.4, 128.4, 127.9, 125.8, 105.0, 60.2, 40.6, 31.8, 28.3, 23.1, 14.5.

Ethyl 5-(2-(methylamino)-7-oxo-6-phenylpyrido[2,3-d]pyrimidin-8(7H)-yl)pentanoate ***(17b)*** ^{1}H-NMR (600 MHz, DMSO-d_6), δ(ppm): 8.60 (1H, s), 7.88 (1H, s), 7.82 (1H, d, J = 4.8 Hz), 7.67 (2H, m), 7.41 (2H, m), 7.34 (1H, m), 4.32 (2H, t, J = 6.9 Hz), 4.05 (2H, q, J = 7.1 Hz), 2.92 (3H, d, J = 4.8 Hz), 2.37 (2H, t, J = 7.2 Hz), 1.72 (2H, m), 1.63 (2H, m), 1.17 (3H, t, J = 7.1 Hz); ^{13}C-NMR (150 MHz, DMSO-d_6), δ(ppm): 173.1, 162.2, 162.0, 159.6, 154.9, 137.0, 134.9, 129.0, 129.0, 128.3, 128.3, 127.7, 125.7, 104.9, 60.1, 40.4, 40.3, 40.2, 40.1, 40.0, 39.8, 39.7, 39.5, 33.5, 28.2, 27.0, 22.5, 14.5.

Ethyl 6-(2-(methylamino)-7-oxo-6-phenylpyrido[2,3-d]pyrimidin-8(7H)-yl)hexanoate ***(17c)*** ^{1}H-NMR (400 MHz, DMSO-d_6), δ(ppm): 8.63 (1H, s), 7.92 (1H, s), 7.81 (1H, d, J = 4.8 Hz), 7.65 (2H, m), 7.41 (2H, m), 7.33 (1H, m), 4.32 (2H, t, J = 7.0 Hz), 4.03 (2H, q, J = 7.1 Hz), 2.90 (3H, d, J = 4.8 Hz), 2.29 (2H, t, J = 7.3 Hz), 1.59-1.71 (4H, m), 1.36 (2H, m), 1.15 (3H, t, J = 7.1 Hz); ^{13}C-NMR (100 MHz, DMSO-d_6), δ(ppm): 173.3, 162.3, 162.1, 159.8, 155.0, 137.0, 135.1, 129.0, 129.0, 128.4, 128.4, 127.9, 125.8, 105.0, 60.1, 40.7, 33.8, 28.3, 27.2, 26.4, 24.6, 14.6.

Ethyl 4-(6-(4-fluorophenyl)-2-(methylamino)-7-oxopyrido[2,3-d]pyrimidin-8(7H)-yl)butanoate ***(17d)*** ^{1}H-NMR (400 MHz, DMSO-d_6), δ(ppm): 8.62 (1H, s), 7.93 (1H, s), 7.82 (1H, d, J = 4.8 Hz), 7.70 (2H, m), 7.24 (2H, m), 4.39 (2H, t, J = 6.5 Hz), 3.97 (2H, q, J = 7.1 Hz), 2.91 (3H, d, J = 4.8 Hz), 2.37 (2H,

t, *J* = 7.0 Hz), 1.99 (2H, m), 1.12 (3H, t, *J* = 7.1 Hz); ^{13}C-NMR (100 MHz, DMSO-d_6), δ(ppm): 171.8, 162.1, 161.2, 159.7, 158.7, 154.1, 134.1, 132.2, 130.0, 129.9, 123.6, 114.2, 114.0, 103.9, 59.2, 39.6, 30.8, 27.2, 22.0, 13.4.

Ethyl 5-(6-(4-fluorophenyl)-2-(methylamino)-7-oxopyrido[2,3-d]pyrimidin-8(7H)-yl)pentanoate ***(17e)*** ^{1}H-NMR (400 MHz, DMSO-d_6), δ(ppm): 8.63 (1H, s), 7.93 (1H, s), 7.82 (1H, d, *J* = 4.7 Hz), 7.70 (2H, m), 7.24 (2H, m), 4.33 (2H, t, *J* = 6.8 Hz), 4.03 (2H, q, *J* = 7.1 Hz), 2.91 (3H, d, *J* = 4.7 Hz), 2.36 (2H, t, *J* = 7.2 Hz), 1.58-1.73 (4H, m), 1.15 (3H, t, *J* = 7.1 Hz); ^{13}C NMR (100 MHz, DMSO-d_6), δ(ppm): 173.2, 163.2, 162.3, 160.8, 159.8, 155.0, 135.1, 133.3, 131.1, 131.0, 124.7, 115.3, 115.1, 104.9, 60.2, 40.6, 33.6, 28.3, 27.1, 22.5, 14.5.

Ethyl 6-(6-(4-fluorophenyl)-2-(methylamino)-7-oxopyrido[2,3-d]pyrimidin-8(7H)-yl)hexanoate ***(17f)*** ^{1}H NMR (400 MHz, DMSO-d_6), δ(ppm): 8.62 (1H, s), 7.92 (1H, s), 7.82 (1H, d, *J* = 4.8 Hz), 7.70 (2H, m), 7.23 (2H, m), 4.31 (2H, t, *J* = 7.2 Hz), 4.03 (2H, q, *J* = 7.1 Hz), 2.90 (3H, d, *J* = 4.8 Hz), 2.29 (2H, t, *J* = 7.4 Hz), 1.38-1.68 (4H, m), 1.35 (2H, m), 1.15 (3H, t, *J* = 7.1 Hz); ^{13}C-NMR (100 MHz, DMSO-d_6), δ(ppm): 173.3, 163.2, 162.3, 162.0, 159.8, 155.0, 135.0, 133.3, 131.1, 131.0, 124.7, 115.3, 115.1, 104.9, 60.1, 40.7, 33.8, 28.3, 27.2, 26.4, 24.6, 14.6.

Ethyl 4-(6-(2,4-difluorophenyl)-2-(methylamino)-7-oxopyrido[2,3-d]pyrimidin-8(7H)-yl)butanoate ***(17g)*** ^{1}H-NMR (400 MHz, DMSO-d_6), δ(ppm): 8.62 (1H, s), 7.88 (1H, d, *J* = 4.7 Hz), 7.85 (1H, s), 7.52 (1H, m), 7.30 (1H, m), 7.15 (1H, m), 4.37 (2H, t, *J* = 6.5 Hz), 3.98 (2H, q, *J* = 7.1 Hz), 2.92 (3H, d, *J* = 4.7 Hz), 2.37 (2H, t, *J* = 7.1 Hz), 1.98 (2H, m), 1.13 (3H, t, *J* = 7.1 Hz); ^{13}C-NMR (125 MHz, DMSO-d_6), δ(ppm): 172.8, 163.3, 162.4, 161.5, 161.3, 159.9, 159.4, 155.5, 137.3, 133.4, 121.4, 120.5, 111.5, 104.4, 60.2, 40.6, 31.8, 28.3, 23.1, 14.5.

Ethyl 5-(6-(2,4-difluorophenyl)-2-(methylamino)-7-oxopyrido[2,3-d]pyrimidin-8(7H)-yl)pentanoate ***(17h)*** ^{1}H-NMR (400 MHz, DMSO-d_6), δ(ppm): 8.62 (1H, s), 7.88 (1H, d, *J* = 4.7 Hz), 7.85 (1H, s), 7.51 (1H, m), 7.30 (1H, m), 7.14 (1H, m), 4.31 (2H, t, *J* = 7.0 Hz), 4.03 (2H, q, *J* = 7.1 Hz), 2.92 (3H, d, *J* = 4.7 Hz), 2.36 (2H, t, *J* = 7.2 Hz), 1.57-1.73 (4H, m), 1.15 (3H, t, *J* = 7.1 Hz); ^{13}C-NMR (125 MHz, DMSO-d_6), δ(ppm): 173.2, 163.4, 162.4, 161.4, 161.4, 159.9, 159.4, 155.4, 137.2, 133.3, 121.5, 120.5, 111.5, 104.4, 60.1, 40.6, 33.6, 28.3, 27.1, 22.5, 14.5.

Ethyl 6-(6-(2,4-difluorophenyl)-2-(methylamino)-7-oxopyrido[2,3-d]pyrimidin-8(7H)-yl)hexanoate ***(17i)*** ^{1}H-NMR (400 MHz, DMSO-d_6), δ(ppm): 8.62 (1H, s), 7.88 (1H, d, *J* = 4.7 Hz), 7.85 (1H, s), 7.52 (1H, m), 7.30 (1H, m), 7.14 (1H, m), 4.29 (2H, t, *J* = 7.1 Hz), 4.03 (2H, q, *J* = 7.1 Hz), 2.91 (3H, d, *J* =

4.7 Hz), 2.29 (2H, t, J = 7.3 Hz), 1.58-1.70 (4H, m), 1.35 (2H, m), 1.15 (3H, t, J = 7.1 Hz); ^{13}C-NMR (125 MHz, DMSO-d_6), δ(ppm): 173.3, 163.4, 162.4, 161.4, 161.3, 159.9, 159.3, 155.4, 137.2, 133.3, 121.4, 120.5, 111.5, 104.3, 60.1, 40.6, 33.8, 28.3, 27.1, 26.4, 24.6, 14.5.

Ethyl 4-(6-(2,4-dichlorophenyl)-2-(methylamino)-7-oxopyrido[2,3-d]pyrimidin-8(7H)-yl)butanoate ***(17j)*** ^{1}H-NMR (400 MHz, DMSO-d_6), δ(ppm): 8.62 (1H, s), 7.89 (1H, d, J = 4.7 Hz), 7.79 (1H, s), 7.69 (1H, d, J = 2.1 Hz), 7.49 (1H, dd, J = 8.3, 2.1 Hz), 7.43 (1H, d, J = 8.3 Hz), 4.36 (2H, t, J = 6.5 Hz), 3.99 (2H, q, J = 7.1 Hz), 2.92 (3H, d, J = 4.7 Hz), 2.36 (2H, t, J = 7.2 Hz), 1.98 (2H, m), 1.13 (3H, t, J = 7.1 Hz); ^{13}C-NMR (100 MHz, DMSO-d_6), δ(ppm): 172.8, 162.4, 161.3, 159.9, 155.6, 137.1, 135.3, 134.7, 133.7, 133.6, 129.1, 127.6, 124.0, 104.4, 60.3, 40.6, 31.7, 28.3, 23.1, 14.5.

Ethyl 5-(6-(2,4-dichlorophenyl)-2-(methylamino)-7-oxopyrido[2,3-d]pyrimidin-8(7H)-yl)pentanoate ***(17k)*** ^{1}H-NMR (400 MHz, DMSO-d_6), δ(ppm): 8.62 (1H, s), 7.89 (1H, d, J = 4.7 Hz), 7.79 (1H, s), 7.69 (1H, d, J = 2.1 Hz), 7.48 (1H, dd, J = 8.3, 2.1 Hz), 7.42 (1H, d, J = 8.3 Hz), 4.32 (2H, t, J = 6.7 Hz), 4.02 (2H, q, J = 7.1 Hz), 2.91 (3H, d, J = 4.7 Hz), 2.36 (2H, t, J = 7.2 Hz), 1.56-1.73 (4H, m), 1.15 (3H, t, J = 7.1 Hz); ^{13}C-NMR (100 MHz, DMSO-d_6), δ(ppm): 173.2, 162.4, 161.3, 159.9, 155.5, 137.0, 135.4, 134.8, 133.7, 133.6, 129.1, 127.6, 124.1, 104.3, 60.1, 40.6, 33.6, 28.3, 27.0, 22.4, 14.5.

Ethyl 6-(6-(2,4-dichlorophenyl)-2-(methylamino)-7-oxopyrido[2,3-d]pyrimidin-8(7H)-yl)hexanoate ***(17l)*** ^{1}H-NMR (400 MHz, DMSO-d_6), δ(ppm): 8.62 (1H, s), 7.89 (1H, d, J = 4.6 Hz), 7,78 (1H, s), 7.69 (1H, d, J = 2.0 Hz), 7.48 (1H, dd, J = 8.2, 2.0 Hz), 7.42 (1H, d, J = 8.2 Hz), 4.30 (2H, t, J = 7.0 Hz), 4.03 (2H, q, J = 7.1 Hz), 2.91 (3H, d, J = 4.6 Hz), 2.28 (2H, t, J = 7.3 Hz), 1.55-1.70 (4H, m), 1.34 (2H, m), 1.15 (3H, t, J = 7.1 Hz); ^{13}C-NMR (100 MHz, DMSO-d_6), δ(ppm): 173.3, 162.5, 161.2, 159.9, 155.5, 137.0, 135.4, 134.7, 133.7, 133.6, 129.1, 127.6, 124.1, 104.4, 60.1, 40.6, 33.8, 28.3, 27.2, 26.3, 24.6, 14.6.

Ethyl 4-(6-(2-chloro-4-fluorophenyl)-2-(methylamino)-7-oxopyrido[2,3-d]pyrimidin-8(7H)-yl)butanoate ***(17m)*** ^{1}H-NMR (400 MHz, DMSO-d_6), δ(ppm): 8.62 (1H, s), 7.87 (1H, d, J = 4.8 Hz), 7.78 (1H, s), 7.52 (1H, m), 7.45 (1H, m), 7.28 (1H, m), 4.37 (2H, t, J = 6.7 Hz), 3.99 (2H, q, J = 7.1 Hz), 2.92 (3H, d, J = 4.8 Hz), 2.37 (2H, t, J = 7.2 Hz), 1.98 (2H, m), 1.13 (3H, t, J = 7.1 Hz); ^{13}C-NMR (125 MHz, DMSO-d_6), δ(ppm): 172.8, 162.9, 162.4, 161.5, 159.8, 155.6, 137.1, 134.6, 133.8, 132.9, 124.2, 117.0, 114.5, 104.4, 60.2, 40.6, 31.7, 28.3, 23.1, 14.5.

Ethyl 5-(6-(2-chloro-4-fluorophenyl)-2-(methylamino)-7-oxopyrido[2,3-d]pyrimidin-8(7H)-yl)pentanoate ***(17n)*** ^{1}H-NMR (400 MHz, DMSO-d_6), δ(ppm): 8.62 (1H, s), 7.87 (1H, d, J = 4.7 Hz), 7.77

(1H, s), 7.52 (1H, m), 7.45 (1H, m), 7.28 (1H, m), 4.32 (2H, t, *J* = 6.8 Hz), 4.02 (2H, q, *J* = 7.1 Hz), 2.91 (3H, d, *J* = 4.7 Hz), 2.36 (2H, t, *J* = 7.3 Hz), 1.56-1.73 (4H, m), 1.15 (3H, t, *J* = 7.1 Hz); ^{13}C-NMR (125 MHz, DMSO-d_6), δ(ppm): 173.2, 162.9, 162.4, 161.4, 159.8, 155.5, 137.0, 134.7, 133.8, 132.9, 124.3, 117.0, 114.5, 104.4, 60.1, 40.6, 33.6, 28.3, 27.1, 22.4, 14.5.

*Ethyl 6-(6-(2-chloro-4-fluorophenyl)-2-(methylamino)-7-oxopyrido[2,3-d]pyrimidin-8(7H)-yl)hexanoate **(17o)*** ^{1}H-NMR (400 MHz, DMSO-d_6), δ(ppm): 8.61 (1H, s), 7.87 (1H, d, *J* = 4.8 Hz), 7.77 (1H, s), 7.52 (1H, m), 7.45 (1H, m), 7.28 (1H, m), 4.30 (2H, t, *J* = 7.1 Hz), 4.03 (2H, q, *J* = 7.1 Hz), 2.91 (3H, d, *J* = 4.8 Hz), 2.28 (2H, t, *J* = 7.3 Hz), 1.56-1.70 (4H, m), 1.35 (2H, m), 1.15 (3H, t, *J* = 7.1 Hz); ^{13}C-NMR (125 MHz, DMSO-d_6), δ(ppm): 173.2, 162.9, 162.5, 161.4, 159.8, 155.5, 137.0, 134.6, 133.8, 132.9, 124.3, 117.0, 114.7, 104.4, 60.1, 40.6, 33.8, 28.3, 27.2, 26.3, 24.6, 14.6.

*Ethyl 4-(2-(ethylamino)-7-oxo-6-phenylpyrido[2,3-d]pyrimidin-8(7H)-yl)butanoate **(18a)*** ^{1}H-NMR (400 MHz, DMSO-d_6), δ(ppm): 8.63 (1H, s), 7.97 (1H, t, *J* = 5.4 Hz), 7.91 (1H, s), 7.65 (2H, m), 7.41 (2H, m), 7.34 (1H, m), 4.37 (2H, t, *J* = 6.5 Hz), 3.98 (2H, q, *J* = 7.1 Hz), 3.40 (2H, m), 2.38 (2H, t, *J* = 7.1 Hz), 1.99 (2H, m), 1.19 (3H, t, *J* = 7.1 Hz), 1.13 (3H, t, *J* = 7.1 Hz); ^{13}C-NMR (100 MHz, DMSO-d_6), δ(ppm): 172.8, 162.2, 161.6, 159.8, 155.1, 137.0, 135.1, 129.0, 129.0, 128.4, 128.4, 127.9, 125.8, 60.2, 40.6, 36.1, 31.8, 23.2, 14.8, 14.5.

*Ethyl 5-(2-(ethylamino)-7-oxo-6-phenylpyrido[2,3-d]pyrimidin-8(7H)-yl)pentanoate **(18b)*** ^{1}H-NMR (400 MHz, DMSO-d_6), δ(ppm): 8.64 (1H, s), 7.98 (1H, t, *J* = 5.5 Hz), 7.92 (1H, s), 7.65 (2H, m), 7.41 (2H, m), 7.33 (1H, m), 4.32 (2H, t, *J* = 6.6 Hz), 4.03 (2H, q, *J* = 7.1 Hz), 3.40 (2H, m), 2.36 (2H, t, *J* = 7.2 Hz), 1.58-1.71 (4H, m), 1.19 (3H, t, *J* = 7.0 Hz), 1.15 (3H, t, *J* = 7.1 Hz); ^{13}C-NMR (100 MHz, DMSO-d_6), δ(ppm): 173.2, 162.1, 161.6, 159.9, 155.0, 137.0, 135.1, 129.0, 129.0, 128.4, 128.4, 127.9, 125.8, 105.1, 60.2, 40.6, 36.2, 33.7, 27.2, 22.5, 14.8, 14.5.

*Ethyl 6-(2-(ethylamino)-7-oxo-6-phenylpyrido[2,3-d]pyrimidin-8(7H)-yl)hexanoate **(18c)*** ^{1}H-NMR (400 MHz, DMSO-d_6), δ(ppm): 8.63 (1H, s), 7.98 (1H, t, *J* = 5.5 Hz), 7.91 (1H, s), 7.65 (2H, m), 7.41 (2H, m), 7.33 (1H, m), 4.30 (2H, t, *J* = 7.0 Hz), 4.03 (2H, q, *J* = 7.1 Hz), 3.39 (2H, m), 2.29 (2H, t, *J* = 7.3 Hz), 1.56-1.69 (4H, m), 1.37 (2H, m), 1.19 (3H, t, *J* = 7.1 Hz), 1.16 (3H, t, *J* = 7.1 Hz); ^{13}C NMR (100 MHz, DMSO-d_6), δ(ppm): 173.3, 162.1, 161.6, 159.8, 155.0, 137.0, 135.0, 129.0, 129.0, 128.4, 128.4, 127.9, 125.8, 60.1, 40.6, 36.2, 33.8, 27.3, 26.4, 24.6, 14.8, 14.6.

*Ethyl 4-(2-(ethylamino)-6-(4-fluorophenyl)-7-oxopyrido[2,3-d]pyrimidin-8(7H)-yl)butanoate **(18d)*** ^{1}H-NMR (400 MHz, DMSO-d_6), δ(ppm): 8.63 (1H, s), 7.99 (1H, t, *J* = 5.4 Hz), 7.92 (1H, s), 7.70 (2H,

m), 7.24 (2H, m), 4.36 (2H, t, J = 6.4 Hz), 3.97 (2H, q, J = 7.1 Hz), 3.40 (2H, m), 2.37 (2H, t, J = 7.1 Hz), 1.99 (2H, m), 1.19 (3H, t, J = 7.0 Hz), 1.12 (3H, t, J = 7.1 Hz); ^{13}C-NMR (100 MHz, DMSO-d_6), δ(ppm): 172.8, 162.2, 161.6, 160.8, 159.9, 155.1, 135.1, 133.3, 131.1, 131.0, 124.7, 115.3, 115.1, 105.0, 60.2, 40.6, 36.1, 31.8, 23.2, 14.8, 14.5.

Ethyl 5-(2-(ethylamino)-6-(4-fluorophenyl)-7-oxopyrido[2,3-d]pyrimidin-8(7H)-yl)pentanoate ***(18e)*** ^{1}H-NMR (400 MHz, DMSO-d_6), δ(ppm): 8.63 (1H, s), 7.97 (1H, t, J = 5.4 Hz), 7.92 (1H, s), 7.70 (2H, m), 7.24 (2H, m), 4.31 (2H, t, J = 6.7 Hz), 4.01 (2H, q, J = 7.1 Hz), 3.39 (2H, m), 2.35 (2H, t, J = 7.4 Hz), 1.58-1.70 (4H, m), 1.19 (3H, t, J = 7.1 Hz), 1.15 (3H, t, J = 7.1 Hz); ^{13}C-NMR (100 MHz, DMSO-d_6), δ(ppm): 173.2, 163.2, 162.1, 161.6, 160.8, 159.9, 155.0, 135.1, 131.1, 131.0, 124.6, 115.3, 115.1, 60.2, 40.6, 36.2, 33.6, 27.2, 22.5, 14.8, 14.5.

Ethyl 6-(2-(ethylamino)-6-(4-fluorophenyl)-7-oxopyrido[2,3-d]pyrimidin-8(7H)-yl)hexanoate ***(18f)*** ^{1}H-NMR (400 MHz, DMSO-d_6), δ(ppm): 8.63 (1H, s), 7.97 (1H, t, J = 5.4 Hz), 7.92 (1H, s), 7.70 (2H, m), 7.23 (2H, m), 4.29 (2H, t, J = 7.2 Hz), 4.03 (2H, q, J = 7.1 Hz), 3.39 (2H, m), 2.29 (2H, t, J = 7.4 Hz), 1.56-1.69 (4H, m), 1.36 (2H, m), 1.19 (3H, t, J = 7.1 Hz), 1.15 (3H, t, J = 7.1 Hz); ^{13}C NMR (100 MHz, DMSO-d_6), δ(ppm): 173.3, 163.2, 162.0, 161.7, 160.8, 159.9, 155.0, 135.0, 133.3, 131.1, 131.0, 115.3, 115.1, 105.0, 60.1, 40.6, 36.2, 33.8, 27.3, 26.4, 24.6, 14.8, 14.6.

Ethyl 4-(6-(2,4-difluorophenyl)-2-(ethylamino)-7-oxopyrido[2,3-d]pyrimidin-8(7H)-yl)butanoate ***(18g)*** ^{1}H-NMR (400 MHz, DMSO-d_6), δ(ppm): 8.63 (1H, s), 7.97 (1H, t, J = 5.4 Hz), 7.84 (1H, s), 7.52 (1H, m), 7.30 (1H, m), 7.15 (1H, m), 4.34 (2H, t, J = 6.7 Hz), 3.99 (2H, q, J = 7.1 Hz), 3.41 (2H, m), 2.37 (2H, t, J = 7.2 Hz), 1.98 (2H, m), 1.19 (3H, t, J = 7.1 Hz), 1.13 (3H, t, J = 7.1 Hz); ^{13}C-NMR (125 MHz, DMSO-d_6), δ(ppm): 172.8, 163.3, 161.8, 161.5, 161.4, 160.0, 159.4, 155.5, 137.2, 133.4, 121.5, 120.4, 111.5, 104.4, 60.2, 40.6, 36.1, 31.8, 23.2, 14.8, 14.5.

Ethyl 5-(6-(2,4-difluorophenyl)-2-(ethylamino)-7-oxopyrido[2,3-d]pyrimidin-8(7H)-yl)pentanoate ***(18h)*** ^{1}H-NMR (400 MHz, DMSO-d_6), δ(ppm): 8.63 (1H, s), 7.98 (1H, t, J = 5.5 Hz), 7.84 (1H, s), 7.51 (1H, m), 7.29 (1H, m), 7.14 (1H, m), 4.29 (2H, t, J = 6.8 Hz), 4.01 (2H, q, J = 7.1 Hz), 3.40 (2H, m), 2.35 (2H, t, J = 7.3 Hz), 1.57-1.70 (4H, m), 1.19 (3H, t, J = 7.2 Hz), 1.15 (3H, t, J = 7.1 Hz); ^{13}C-NMR (125 MHz, DMSO-d_6), δ(ppm): 173.1, 163.4, 161.8, 161.4, 161.3, 160.0, 159.4, 155.4, 137.2, 133.3, 121.4, 120.5, 111.5, 104.3, 60.1, 40.6, 36.2, 33.6, 27.2, 22.5, 14.7, 14.5.

Ethyl 6-(6-(2,4-difluorophenyl)-2-(ethylamino)-7-oxopyrido[2,3-d]pyrimidin-8(7H)-yl)hexanoate ***(18i)*** ^{1}H-NMR (400 MHz, DMSO-d_6), δ(ppm): 8.63 (1H, s), 7.98 (1H, t, J = 5.5 Hz), 7.84 (1H, s), 7.51

(1H, m), 7.30 (1H, m), 7.14 (1H, m), 4.27 (2H, t, J = 7.2 Hz), 4.03 (2H, q, J = 7.1 Hz), 3.39 (2H, m), 2.29 (2H, t, J = 7.3 Hz), 1.55-1.68 (4H, m), 1.35 (2H, m), 1.19 (3H, t, J = 7.1 Hz), 1.15 (3H, t, J = 7.1 Hz); ^{13}C-NMR (125 MHz, DMSO-d_6), δ(ppm): 173.2, 163.3, 161.8, 161.4, 160.0, 159.4, 155.3, 137.1, 133.4, 121.5, 120.5, 111.7, 104.4, 60.1, 40.6, 36.2, 33.8, 27.3, 26.4, 24.6, 14.7, 14.6.

*Ethyl 4-(6-(2,4-dichlorophenyl)-2-(ethylamino)-7-oxopyrido[2,3-d]pyrimidin-8(7H)-yl)butanoate **(18j)*** ^{1}H-NMR (400 MHz, DMSO-d_6), δ(ppm): 8.63 (1H, s), 7.99 (1H, t, J = 5.4 Hz), 7.78 (1H, s), 7.69 (1H, d, J = 2.0 Hz), 7.48 (1H, dd, J = 8.3, 2.0 Hz), 7.42 (1H, d, J = 8.3 Hz), 4.34 (2H, t, J = 6.7 Hz), 3.99 (2H, q, J = 7.1 Hz), 3.41 (2H, m), 2.36 (2H, t, J = 7.3 Hz), 1.97 (2H, m), 1.19 (3H, t, J = 7.2 Hz), 1.13 (3H, t, J = 7.1 Hz); ^{13}C-NMR (100 MHz, DMSO-d_6), δ(ppm): 172.8, 161.8, 161.3, 160.0, 155.6, 137.1, 135.4, 134.7, 133.7, 133.6, 129.1, 127.6, 123.9, 104.4, 60.2, 40.7, 36.1, 31.7, 23.2, 14.8, 14.5.

*Ethyl 5-(6-(2,4-dichlorophenyl)-2-(ethylamino)-7-oxopyrido[2,3-d]pyrimidin-8(7H)-yl)pentanoate **(18k)*** ^{1}H-NMR (400 MHz, DMSO-d_6), δ(ppm): 8.62 (1H, s), 7.99 (1H, t, J = 5.4 Hz), 7.78 (1H, s), 7.69 (1H, d, J = 2.1 Hz), 7.48 (1H, dd, J = 8.2, 2.1 Hz), 7.42 (1H, d, J = 8.2 Hz), 4.29 (2H, t, J = 6.8 Hz), 4.02 (2H, q, J = 7.1 Hz), 3.41 (2H, m), 2.34 (2H, t, J = 7.3 Hz), 1.56-1.72 (4H, m), 1.19 (3H, t, J = 7.1 Hz), 1.15 (3H, t, J = 7.1 Hz); ^{13}C-NMR (125 MHz, DMSO-d_6), δ(ppm): 173.1, 161.8, 161.3, 160.0, 155.5, 137.0, 135.4, 134.8, 133.7, 133.6, 129.1, 127.6, 124.0, 104.4, 60.1, 40.6, 36.2, 33.6, 27.2, 22.4, 14.8, 14.5.

*Ethyl 6-(6-(2,4-dichlorophenyl)-2-(ethylamino)-7-oxopyrido[2,3-d]pyrimidin-8(7H)-yl)hexanoate **(18l)*** ^{1}H-NMR (400 MHz, DMSO-d_6), δ(ppm): 8.62 (1H, s), 7.99 (1H, t, J = 5.4 Hz), 7.78 (1H, s), 7.69 (1H, d, J = 2.1 Hz), 7.48 (1H, dd, J = 8.3, 2.1 Hz), 7.42 (1H, d, J = 8.3 Hz), 4.27 (2H, t, J = 7.2 Hz), 4.03 (2H, q, J = 7.1 Hz), 3.39 (2H, m), 2.28 (2H, t, J = 7.3 Hz), 1.55-1.69 (4H, m), 1.34 (2H, m), 1.21 (3H, t, J = 7.0 Hz), 1.15 (3H, t, J = 7.1 Hz); ^{13}C-NMR (125 MHz, DMSO-d_6), δ(ppm): 173.2, 161.8, 161.2, 160.0, 155.5, 137.0, 135.4, 134.7, 133.7, 133.6, 129.1, 127.6, 124.0, 104.4, 60.1, 40.6, 36.2, 33.9, 27.3, 26.3, 24.6, 14.8, 14.6.

*Ethyl 4-(6-(2-chloro-4-fluorophenyl)-2-(ethylamino)-7-oxopyrido[2,3-d]pyrimidin-8(7H)-yl)butanoate **(18m)*** ^{1}H-NMR (400 MHz, DMSO-d_6), δ(ppm): 8.62 (1H, s), 7.97 (1H, t, J = 5.3 Hz), 7.77 (1H, s), 7.52 (1H, m), 7.45 (1H, m), 7.29 (1H, m), 4.34 (2H, t, J = 6.9 Hz), 3.99 (2H, q, J = 7.1 Hz), 3.41 (2H, m), 2.36 (2H, t, J = 7.2 Hz), 1.97 (2H, m), 1.19 (3H, t, J = 7.1 Hz), 1.13 (3H, t, J = 7.1 Hz); ^{13}C-NMR (125 MHz, DMSO-d_6), δ(ppm): 172.8, 162.9, 161.8, 160.9, 159.9, 155.6, 137.1, 134.5, 133.8, 132.9, 124.2, 116.8, 114.5, 104.5, 60.2, 40.6, 36.1, 31.7, 23.2, 14.8, 14.5.

Ethyl 5-(6-(2-chloro-4-fluorophenyl)-2-(ethylamino)-7-oxopyrido[2,3-d]pyrimidin-8(7H)-yl)pentanoate ***(18n)*** ^{1}H-NMR (400 MHz, DMSO-d_6), δ(ppm): 8.62 (1H, s), 7.97 (1H, t, J = 5.4 Hz), 7.76 (1H, s), 7.52 (1H, m), 7.44 (1H, m), 7.28 (1H, m), 4.29 (2H, t, J = 6.7 Hz), 4.02 (2H, q, J = 7.1 Hz), 3.40 (2H, m), 2.34 (2H, t, J = 7.2 Hz), 1.57-1.72 (4H, m), 1.20 (3H, t, J = 7.1 Hz), 1.15 (3H, t, J = 7.1 Hz); ^{13}C-NMR (125 MHz, DMSO-d_6), δ(ppm): 173.1, 162.9, 161.8, 160.9, 159.9, 155.5, 137.0, 134.7, 133.7, 132.9, 124.3, 116.8, 114.7, 104.4, 60.1, 40.6, 36.2, 33.7, 27.2, 22.4, 14.8, 14.5.

Ethyl 6-(6-(2-chloro-4-fluorophenyl)-2-(ethylamino)-7-oxopyrido[2,3-d]pyrimidin-8(7H)-yl)hexanoate ***(18o)*** ^{1}H-NMR (400 MHz, DMSO-d_6), δ(ppm): 8.62 (1H, s), 7.97 (1H, t, J = 5.4 Hz), 7.76 (1H, s), 7.52 (1H, m), 7.44 (1H, m), 7.28 (1H, m), 4.27 (2H, t, J = 7.2 Hz), 4.03 (2H, q, J = 7.1 Hz), 3.39 (2H, m), 2.28 (2H, t, J = 7.3 Hz), 1.55-1.69 (4H, m), 1.35 (2H, m), 1.19 (3H, t, J = 7.0 Hz), 1.15 (3H, t, J = 7.1 Hz); ^{13}C-NMR (125 MHz, DMSO-d_6), δ(ppm): 173.2, 162.9, 161.8, 160.9, 159.9, 155.4, 136.9, 134.6, 133.8, 132.9, 124.3, 116.8, 114.5, 104.4, 60.1, 40.6, 36.2, 33.9, 27.3, 26.3, 24.6, 14.8, 14.6.

Ethyl 4-(2-(cyclopropylamino)-7-oxo-6-phenylpyrido[2,3-d]pyrimidin-8(7H)-yl)butanoate ***(19a)*** ^{1}H-NMR (400 MHz, DMSO-d_6), δ(ppm): 8.64 (1H, s), 8.08 (1H, br s), 7.93 (1H, s), 7.66 (2H, m), 7.41 (2H, m), 7.34 (1H, m), 4.41 (2H, br s), 3.96 (2H, q, J = 7.1 Hz), 2.85 (1H, m), 2.40 (2H, br s), 2.02 (2H, m), 1.12 (3H, t, J = 7.1 Hz), 0.75 (2H, m), 0.56 (2H, m); ^{13}C-NMR (100 MHz, DMSO-d_6), δ(ppm): 172.8, 162.9, 162.2, 159.5, 155.1, 137.0, 135.0, 129.0, 129.0, 128.4, 128.4, 127.9, 126.3, 60.2, 40.6, 31.9, 24.4, 23.2, 14.5, 6.7.

Ethyl 5-(2-(cyclopropylamino)-7-oxo-6-phenylpyrido[2,3-d]pyrimidin-8(7H)-yl)pentanoate ***(19b)*** ^{1}H-NMR (400 MHz, DMSO-d_6), δ(ppm): 8.64 (1H, s), 8.08 (1H, br s), 7.93 (1H, s), 7.66 (2H, m), 7.41 (2H, m), 7.34 (1H, m), 4.36 (2H, br s), 4.02 (2H, q, J = 7.1 Hz), 2.83 (1H, br s), 2.36 (2H, t, J = 7.3 Hz), 1.61-1.73 (4H, m), 1.15 (3H, t, J = 7.1 Hz), 0.75 (2H, m), 0.56 (2H, m); ^{13}C-NMR (100 MHz, DMSO-d_6), δ(ppm): 173.2, 162.9, 162.1, 159.5, 155.0, 137.0, 135.0, 129.0, 129.0, 128.4, 128.4, 127.9, 126.2, 60.1, 40.6, 33.7, 27.2, 24.4, 22.5, 14.5, 6.7.

Ethyl 6-(2-(cyclopropylamino)-7-oxo-6-phenylpyrido[2,3-d]pyrimidin-8(7H)-yl)hexanoate ***(19c)*** ^{1}H-NMR (400 MHz, DMSO-d_6), δ(ppm): 8.64 (1H, s), 8.08 (1H, br s), 7.93 (1H, s), 7.66 (2H, m), 7.41 (2H, m), 7.34 (1H, m), 4.34 (2H, br s), 4.02 (2H, q, J = 7.1 Hz), 2.81 (1H, br s), 2.29 (2H, t, J = 7.3 Hz), 1.56-1.71 (4H, m), 1.35 (2H, m), 1.15 (3H, t, J = 7.1 Hz), 0.75 (2H, m), 0.56 (2H, m); ^{13}C-NMR (100 MHz, DMSO-d_6), δ(ppm): 173.3, 162.9, 162.1, 159.5, 154.9, 137.0, 135.0, 129.1, 129.1, 128.4, 127.4, 127.9, 60.1, 40.6, 33.9, 27.3, 26.4, 24.7, 24.4, 14.6, 6.7.

Ethyl 4-(2-(cyclopropylamino)-6-(4-fluorophenyl)-7-oxopyrido[2,3-d]pyrimidin-8(7H)-yl)butanoate ***(19d)*** ^{1}H-NMR (400 MHz, DMSO-d_6), δ(ppm): 8.63 (1H, s), 8.09 (1H, br s), 7.94 (1H, s), 7.71 (2H, m), 7.24 (2H, m), 4.40 (2H, br s), 3.96 (2H, q, J = 7.1 Hz), 2.85 (1H, m), 2.40 (2H, br s), 2.01 (2H, m), 1.11 (3H, t, J = 7.1 Hz), 0.75 (2H, m), 0.56 (2H, m); ^{13}C-NMR (100 MHz, DMSO-d_6), δ(ppm): 172.8, 163.2, 162.2, 160.8, 159.5, 155.1, 135.0, 133.3, 131.1, 131.0, 125.1, 115.3, 115.1, 60.2, 40.6, 31.9, 24.4, 23.2, 14.5, 6.7.

Ethyl 5-(2-(cyclopropylamino)-6-(4-fluorophenyl)-7-oxopyrido[2,3-d]pyrimidin-8(7H)-yl)pentanoate ***(19e)*** ^{1}H-NMR (400 MHz, DMSO-d_6), δ(ppm): 8.64 (1H, s), 8.09 (1H, br s), 7.94 (1H, s), 7.71 (2H, m), 7.24 (2H, m), 4.36 (2H, br s), 4.02 (2H, q, J = 7.1 Hz), 2.83 (1H, br s), 2.36 (2H, t, J = 7.3 Hz), 1.60-1.73 (4H, m), 1.15 (3H, t, J = 7.1 Hz), 0.75 (2H, m), 0.56 (2H, m); ^{13}C-NMR (100 MHz, DMSO-d_6), δ(ppm): 173.2, 163.2, 162.1, 160.8, 159.6, 154.9, 135.0, 133.3, 131.1, 131.0, 125.1, 115.3, 115.1, 60.1, 40.6, 33.7, 27.2, 24.4, 22.5, 14.5, 6.7.

Ethyl 6-(2-(cyclopropylamino)-6-(4-fluorophenyl)-7-oxopyrido[2,3-d]pyrimidin-8(7H)-yl)hexanoate ***(19f)*** ^{1}H-NMR (400 MHz, DMSO-d_6), δ(ppm): 8.63 (1H, s), 8.09 (1H, br s), 7.94 (1H, s), 7.71 (2H, m), 7.24 (2H, m), 4.34 (2H, br s), 4.02 (2H, q, J = 7.1 Hz), 2.81 (1H, br s), 2.29 (2H, t, J = 7.3 Hz), 1.56-1.70 (4H, m), 1.37 (2H, m), 1.15 (3H, t, J = 7.1 Hz), 0.75 (2H, m), 0.56 (2H, m); ^{13}C-NMR (100 MHz, DMSO-d_6), δ(ppm): 173.3, 163.2, 162.0, 160.8, 159.6, 154.9, 134.9, 133.3, 131.1, 131.0, 125.1, 115.3, 115.1, 60.1, 40.6, 33.8, 27.3, 26.4, 24.6, 24.4, 14.5, 6.7.

Ethyl 4-(2-(cyclopropylamino)-6-(2,4-difluorophenyl)-7-oxopyrido[2,3-d]pyrimidin-8(7H)-yl)butanoate ***(19g)*** ^{1}H-NMR (400 MHz, DMSO-d_6), δ(ppm): 8.63 (1H, s), 8.14 (1H, br s), 7.86 (1H, s), 7.52 (1H, m), 7.30 (1H, m), 7.15 (1H, m), 4.38 (2H, br s), 3.97 (2H, q, J = 7.1 Hz), 2.86 (1H, br s), 2.39 (2H, m), 2.00 (2H, m), 1.12 (3H, t, J = 7.1 Hz), 0.76 (2H, m), 0.56 (2H, m); ^{13}C NMR (100 MHz, DMSO-d_6), δ(ppm): 172.8, 163.1, 161.5, 161.4, 161.3, 159.7, 159.3, 155.4, 137.1, 133.4, 121.5, 121.0, 111.7, 104.4, 60.2, 40.5, 31.6, 30.3, 24.4, 23.2, 14.5, 6.6.

Ethyl 5-(2-(cyclopropylamino)-6-(2,4-difluorophenyl)-7-oxopyrido[2,3-d]pyrimidin-8(7H)-yl)pentanoate ***(19h)*** ^{1}H-NMR (400 MHz, DMSO-d_6), δ(ppm): 8.63 (1H, s), 8.15 (1H, br s), 7.86 (1H, s), 7.52 (1H, m), 7.30 (1H, m), 7.15 (1H, m), 4.34 (2H, br s), 4.02 (2H, q, J = 7.1 Hz), 2.83 (1H, br s), 2.35 (2H, t, J = 7.3 Hz), 1.60-1.73 (4H, m), 1.14 (3H, t, J = 7.1 Hz), 0.76 (2H, m), 0.56 (2H, m); ^{13}C-NMR (125 MHz, DMSO-d_6), δ(ppm): 173.2, 163.1, 161.4, 161.4, 161.3, 159.7, 159.4, 155.3, 137.1, 133.4, 121.3, 121.0, 111.7, 104.4, 60.1, 40.6, 33.7, 27.2, 24.4, 22.5, 14.5, 6.7.

*Ethyl 6-(2-(cyclopropylamino)-6-(2,4-difluorophenyl)-7-oxopyrido[2,3-d]pyrimidin-8(7H)-yl)hexanoate (**19i**)* ^{1}H-NMR (400 MHz, DMSO-d_6), δ(ppm): 8.63 (1H, s), 8.15 (1H, br s), 7.86 (1H, s), 7.52 (1H, m), 7.30 (1H, m), 7.15 (1H, m), 4.32 (2H, br s), 4.02 (2H, q, *J* = 7.1 Hz), 2.81 (1H, br s), 2.28 (2H, t, *J* = 7.3 Hz), 1.55-1.70 (4H, m), 1.36 (2H, m), 1.15 (3H, t, *J* = 7.1 Hz), 0.74 (2H, m), 0.56 (2H, m); ^{13}C-NMR (125 MHz, DMSO-*d*6), δ(ppm): 173.3, 163.1, 161.4, 161.4, 161.3, 159.6, 159.3, 155.3, 137.1, 133.4, 121.3, 121.0, 111.7, 104.4, 60.1, 40.6, 33.8, 27.3, 26.4, 24.6, 24.4, 14.5, 6.7.

*Ethyl 4-(2-(cyclopropylamino)-6-(2,4-dichlorophenyl)-7-oxopyrido[2,3-d]pyrimidin-8(7H)-yl)butanoate (**19j**)* ^{1}H-NMR (400 MHz, DMSO-d_6), δ(ppm): 8.63 (1H, s), 8.16 (1H, br s), 7.80 (1H, s), 7.70 (1H, d, *J* = 2.1 Hz), 7.50 (1H, dd, *J* = 8.3, 2.1 Hz), 7.43 (1H, d, *J* = 8.3 Hz), 4.38 (2H, br s), 3.99 (2H, q, *J* = 7.1 Hz), 2.85 (1H, br s), 2.38 (2H, br s), 2.00 (2H, br s), 1.12 (3H, t, *J* = 7.1 Hz), 0.76 (2H, m), 0.56 (2H, m); ^{13}C-NMR (125 MHz, DMSO-d_6), δ(ppm): 172.8, 163.1, 161.4, 159.7, 155.5, 137.0, 135.3, 134.7, 133.7, 133.6, 129.1, 127.6, 60.2, 40.6, 31.8, 24.4, 23.2, 14.5, 6.6.

*Ethyl 5-(2-(cyclopropylamino)-6-(2,4-dichlorophenyl)-7-oxopyrido[2,3-d]pyrimidin-8(7H)-yl)pentanoate (**19k**)* ^{1}H-NMR (400 MHz, DMSO-d_6), δ(ppm): 8.63 (1H, s), 8.16 (1H, br s), 7.80 (1H, s), 7.69 (1H, d, *J* = 2.1 Hz), 7.49 (1H, dd, J = 8.2, 2.1 Hz), 7.42 (1H, d, *J* = 8.2 Hz), 4.34 (2H, br s), 4.02 (2H, q, *J* = 7.1 Hz), 2.83 (1H, br s), 2.35 (2H, t, *J* = 7.3 Hz), 1.59-1.73 (4H, m), 1.14 (3H, t, *J* = 7.1 Hz), 0.76 (2H, m), 0.56 (2H, m); ^{13}C-NMR (125 MHz, DMSO-d_6), δ(ppm): 173.2, 163.1, 161.3, 159.7, 155.4, 136.9, 135.4, 134.7, 133.7, 133.6, 129.1, 127.6, 124.5, 104.9, 60.1, 40.6, 33.7, 27.2, 24.4, 22.4, 14.5, 6.7.

*Ethyl 6-(2-(cyclopropylamino)-6-(2,4-dichlorophenyl)-7-oxopyrido[2,3-d]pyrimidin-8(7H)-yl)hexanoate (**19l**)* ^{1}H-NMR (400 MHz, DMSO-d_6), δ(ppm): 8.63 (1H, s), 8.16 (1H, br s), 7.79 (1H, s), 7.69 (1H, d, *J* = 2.1 Hz), 7.49 (1H, dd, *J* = 2.1, 8.3 Hz), 7.42 (1H, d, *J* = 8.3 Hz), 4.32 (2H, br s), 4.02 (2H, q, *J* = 7.1 Hz), 2.81 (1H, br s), 2.28 (2H, t, *J* = 7.3 Hz), 1.57-1.71 (4H, m), 1.36 (2H, m), 1.15 (3H, t, *J* = 7.1 Hz), 0.76 (2H, m), 0.56 (2H, m); ^{13}C-NMR (125 MHz, DMSO-d_6), δ(ppm): 173.2, 163.1, 161.2, 159.7, 155.4, 136.9, 135.4, 134.7, 133.7, 133.6, 129.1, 127.6, 124.5, 104.9, 60.1, 40.6, 33.9, 27.3, 26.3, 24.6, 24.4, 14.5, 6.7.

*Ethyl 4-(6-(2-chloro-4-fluorophenyl)-2-(cyclopropylamino)-7-oxopyrido[2,3-d]pyrimidin-8(7H)-yl)butanoate (**19m**)* ^{1}H-NMR (400 MHz, DMSO-d_6), δ(ppm): 8.63 (1H, s), 8.15 (1H, br s), 7.79 (1H, s), 7.52 (1H, m), 7.45 (1H, m), 7.29 (1H, m), 4.38 (2H, br s), 3.98 (2H, q, *J* = 7.1 Hz), 2.86 (1H, br s), 2.38 (2H, m), 2.00 (2H, m), 1.13 (3H, t, *J* = 7.1 Hz), 0.76 (2H, m), 0.56 (2H, m); ^{13}C-NMR (125 MHz, DMSO-d_6), δ(ppm): 172.8, 163.1, 161.5, 160.9, 159.6, 155.5, 137.0, 134.6, 133.7, 132.8, 124.7, 117.0, 114.7,

104.9, 60.2, 40.6, 31.8, 24.4, 23.2, 14.5, 6.6.

*Ethyl 5-(6-(2-chloro-4-fluorophenyl)-2-(cyclopropylamino)-7-oxopyrido[2,3-d]pyrimidin-8(7H)-yl)pentanoate **(19n)*** ^{1}H-NMR (400 MHz, DMSO-d_6), δ(ppm): 8.63 (1H, s), 8.15 (1H, br s), 7.78 (1H, s), 7.52 (1H, m), 7.45 (1H, m), 7.28 (1H, m), 4.34 (2H, br s), 4.02 (2H, q, *J* = 7.1 Hz), 2.83 (1H, br s), 2.35 (2H, t, *J* = 7.3 Hz), 1.59-1.73 (4H, m), 1.14 (3H, t, *J* = 7.1 Hz), 0.76 (2H, m), 0.56 (2H, m); ^{13}C-NMR (125 MHz, DMSO-d_6), δ(ppm): 173.2, 163.1, 161.4, 160.9, 159.6, 155.4, 136.9, 134.6, 133.8, 132.9, 124.7, 117.0, 114.5, 104.9, 60.1, 40.6, 33.7, 27.2, 24.4, 22.4, 14.5, 6.7.

*Ethyl 6-(6-(2-chloro-4-fluorophenyl)-2-(cyclopropylamino)-7-oxopyrido[2,3-d]pyrimidin-8(7H)-yl)hexanoate **(19o)*** ^{1}H-NMR (400 MHz, DMSO-d_6), δ(ppm): 8.62 (1H, s), 8.14 (1H, br s), 7.78 (1H, s), 7.52 (1H, m), 7.45 (1H, m), 7.28 (1H, m), 4.32 (2H, br s), 4.02 (2H, q, *J* = 7.1 Hz), 2.81 (1H, br s), 2.28 (2H, t, *J* = 7.3 Hz), 1.57-1.70 (4H, m), 1.35 (2H, m), 1.15 (3H, t, *J* = 7.1 Hz), 0.75 (2H, m), 0.56 (2H, m); ^{13}C-NMR (125 MHz, DMSO-d_6), δ(ppm): 173.3, 163.1, 162.9, 161.4, 159.6, 155.4, 136.9, 134.6, 133.8, 132.9, 124.7, 117.0, 114.5, 104.9, 60.1, 40.6, 33.9, 27.3, 26.3, 24.6, 24.4, 14.5, 6.7.

*General Procedure for the Preparation of **20a-20o, 21a-21o and 22a-22o.*** To a solution of intermediate **17** or **18** or **19** (0.5 mmol) in dichloromethane (2 mL), the solution of hydroxylamine hydrochloride (20 mmol) and NaOH (40 mmol) were added at 0 °C in methanol (10 ml). The reaction mixture was stirred for 10-60 min. After removing the solvent under reduced pressure, the residues was diluted with 15 ml water, and acidified with 1N HCl to pH = 8, and extracted with ethyl acetate (3 × 30 mL). The combined organic layers were washed with saturated aqueous sodium bicarbonate and brine, and then dried over anhydrous sodium sulfate. After removing the solvent under reduced pressure, and purified by silica sel flash chromatography (dichloromethane/methanol 10-20%) as a white solid, yield 65-78%.

*N-hydroxy-4-(2-(methylamino)-7-oxo-6-phenylpyrido[2,3-d]pyrimidin-8(7H)-yl)butanamide **(20a)*** ^{1}H-NMR (600 MHz, DMSO-d_6), δ(ppm): 10.41 (1H, s), 8.72 (1H, s), 8.64 (1H, s), 7.93 (1H, s), 7.81 (1H, d, *J* = 4.3 Hz), 7.66 (2H, m), 7.41 (2H, m), 7.34 (1H, m), 4.35 (2H, t, *J* = 6.6 Hz), 2.91 (3H, d, *J* = 4.3 Hz), 2.05 (2H, t, *J* = 7.2 Hz), 1.92 (2H, m); ^{13}C-NMR (150 MHz, DMSO-d_6), δ(ppm): 168.9, 162.2, 162.1, 159.8, 155.1, 137.0, 135.2, 129.0, 129.0, 128.4, 128.4, 127.9, 125.8, 105.0, 40.5, 30.7, 28.3, 24.1; HRMS (ESI)+ calculated for $C_{18}H_{19}N_5O_3$, $[M+H]^+$: m/z 354.1561, found 354.2; HPLC(t_R = 4.308 min, 96.3%).

*N-hydroxy-5-(2-(methylamino)-7-oxo-6-phenylpyrido[2,3-d]pyrimidin-8(7H)-yl)pentanamide **(20b)***

^{1}H-NMR (400 MHz, DMSO-d_6), δ(ppm): 10.36 (1H, s), 8.72 (1H, s), 8.64 (1H, s), 7.93 (1H, s), 7.82 (1H, d, J = 4.7 Hz), 7.65 (2H, m), 7.40 (2H, m), 7.33 (1H, m), 4.33 (2H, t, J = 6.4 Hz), 2.91 (3H, d, J = 4.7 Hz), 2.01 (2H, t, J = 7.3 Hz), 1.57-1.70 (4H, m); ^{13}C-NMR (100 MHz, DMSO-d_6), δ(ppm): 169.4, 162.3, 162.1, 159.8, 155.0, 137.0, 135.1, 129.0, 129.0, 128.4, 128.4, 127.9, 40.6, 32.6, 28.3, 27.5, 23.5; HRMS (ESI)+ calculated for $C_{19}H_{21}N_5O_3$, $[M+H]^+$: m/z 368.1718, found 368.2; HPLC(t_R = 4.793 min, 95.7%).

N-hydroxy-6-(2-(methylamino)-7-oxo-6-phenylpyrido[2,3-d]pyrimidin-8(7H)-yl)hexanamide ***(20c)*** ^{1}H-NMR (400 MHz, DMSO-d_6), δ(ppm): 10.38 (1H, s), 8.71 (1H, s), 8.64 (1H, s), 7.92 (1H, s), 7.82 (1H, d, J = 4.6 Hz), 7.65 (2H, m), 7.40 (2H, m), 7.34 (1H, m), 4.32 (2H, t, J = 7.0 Hz), 2.90 (3H, d, J = 4.6 Hz), 1.96 (2H, t, J = 7.3 Hz), 1.56-1.68 (4H, m), 1.34 (2H, m); ^{13}C-NMR (100 MHz, DMSO-d_6), δ(ppm): 169.4, 162.3, 162.0, 159.8, 155.0, 137.0, 135.1, 129.0, 129.0, 128.4, 128.4, 127.8, 125.8, 105.0, 40.5, 40.4, 40.2, 40.1, 40.0, 39.8, 39.7, 39.5, 32.6, 28.3, 27.4, 26.7, 25.3; HRMS (ESI)+ calculated for $C_{20}H_{23}N_5O_3$, $[M+H]^+$: m/z 382.1874, found 382.2; HPLC(t_R = 4.991 min, 96.6%).

4-(6-(4-fluorophenyl)-2-(methylamino)-7-oxopyrido[2,3-d]pyrimidin-8(7H)-yl)-N-hydroxybutanamide ***(20d)*** ^{1}H-NMR (400 MHz, DMSO-d_6), δ(ppm): 10.39 (1H, s), 8.70 (1H, s), 8.62 (1H, s), 7.93 (1H, s), 7.81 (1H, d, J = 4.4 Hz), 7.71 (2H, m), 7.24 (2H, m), 4.35 (2H, t, J = 6.3 Hz), 2.91 (3H, d, J = 4.4 Hz), 2.05 (2H, t, J = 7.3 Hz), 1.93 (2H, m); ^{13}C-NMR (100 MHz, DMSO-d_6), δ(ppm): 168.9, 163.2, 162.3, 160.8, 159.8, 155.0, 135.1, 133.3, 131.1, 131.0, 124.6, 115.3, 115.1, 104.9, 40.6, 30.7, 28.3, 24.1; HRMS (ESI)+ calculated for $C_{18}H_{18}FN_5O_3$, $[M+H]^+$: m/z 372.1467, found 372.1; HPLC(t_R = 4.529 min, 99.0%).

5-(6-(4-fluorophenyl)-2-(methylamino)-7-oxopyrido[2,3-d]pyrimidin-8(7H)-yl)-N-hydroxypentanamide ***(20e)*** ^{1}H-NMR (400 MHz, DMSO-d_6), δ(ppm): 10.34 (1H, s), 8.71 (1H, s), 8.63 (1H, s), 7.93 (1H, s), 7.82 (1H, d, J = 4.7 Hz), 7.71 (2H, m), 7.24 (2H, m), 4.31 (2H, t, J = 6.6 Hz), 2,90 (3H, d, J = 4.7 Hz), 2.01 (2H, t, J = 7.2 Hz), 1.57-1.67 (4H, m); ^{13}C-NMR (100 MHz, DMSO-d_6), δ(ppm): 169.4, 162.3, 162.0, 160.8, 159.8, 155.0, 135.1, 133.3, 131.1, 131.0, 124.7, 115.3, 115.1, 104.9, 40.6, 32.6, 28.3, 27.4, 23.5; HRMS (ESI)+ calculated for $C_{19}H_{20}FN_5O_3$, $[M+H]^+$: m/z 386.1623, found 386.2; HPLC(t_R = 5.116 min, 97.3%).

6-(6-(4-fluorophenyl)-2-(methylamino)-7-oxopyrido[2,3-d]pyrimidin-8(7H)-yl)-N-hydroxyhexanamide ***(20f)*** ^{1}H-NMR (400 MHz, DMSO-d_6), δ(ppm): 10.33 (1H, s), 8.70 (1H, s), 8.62 (1H, s), 7.92 (1H, s), 7.81 (1H, d, J = 4.8 Hz), 7.71 (2H, m), 7.23 (2H, m), 4.31 (2H, t, J = 6.4 Hz), 2.90 (3H, d, J = 4.8 Hz), 1.96 (2H, t, J = 7.4 Hz), 1.55-1.67 (4H, m), 1.34 (2H, m); ^{13}C-NMR (125 MHz, DMSO-

d_6), δ(ppm): 169.5, 163.0, 162.3, 161.0, 159.8, 155.0, 135.0, 133.3, 131.1, 131.0, 124.7, 115.3, 115.1, 104.9, 40.5, 32.6, 28.3, 27.3, 26.7, 25.3; HRMS (ESI)+ calculated for $C_{20}H_{22}FN_5O_3$, $[M+H]^+$: m/z 400.1780, found 400.2; HPLC(t_R = 5.987 min, 98.6%).

*4-(6-(2,4-difluorophenyl)-2-(methylamino)-7-oxopyrido[2,3-d]pyrimidin-8(7H)-yl)-N-hydroxybutanamide (**20g**)* ^{1}H-NMR (400 MHz, DMSO-d_6), δ(ppm): 10.38 (1H, s), 8.71 (1H, s), 8.63 (1H, s), 7.88 (1H, d, *J* = 4.6 Hz), 7.86 (1H, s), 7.52 (1H, m), 7.30 (1H, m), 7.14 (1H, m), 4.33 (2H, t, *J* = 6.8 Hz), 2.92 (3H, d, *J* = 4.6 Hz), 2.04 (2H, t, *J* = 7.0 Hz), 1.91 (2H, m); ^{13}C-NMR (125 MHz, DMSO-d_6), δ(ppm): 168.9, 162.4, 161.5, 159.9, 159.4, 155.4, 137.3, 133.4, 121.5, 120.5, 111.7, 104.4, 40.6, 30.7, 28.3, 24.1; HRMS (ESI)+ calculated for $C_{18}H_{17}F_2N_5O_3$, $[M+H]^+$: m/z 390.1373, found 390.1; HPLC(t_R = 4.215 min, 96.2%).

*5-(6-(2,4-difluorophenyl)-2-(methylamino)-7-oxopyrido[2,3-d]pyrimidin-8(7H)-yl)-N-hydroxypentanamide (**20h**)* ^{1}H-NMR (400 MHz, DMSO-d_6), δ(ppm): 10.34 (1H, s), 8.71 (1H, s), 8.63 (1H, s), 7.88 (1H, d, *J* = 4.7 Hz), 7.85 (1H, s), 7.52 (1H, m), 7.30 (1H, m), 7.14 (1H, m), 4.31 (2H, t, *J* = 6.7 Hz), 2.91 (3H, d, *J* = 4.7 Hz), 2.01 (2H, t, *J* = 7.1 Hz), 1.55-1.66 (4H, m); ^{13}C-NMR (125 MHz, DMSO-d_6), δ(ppm): 169.3, 163.4, 162.4, 161.4, 159.9, 159.4, 159.3, 155.4, 137.2, 133.4, 121.5, 120.5, 111.5, 104.4, 40.6, 32.6, 28.3, 27.4, 23.4; HRMS (ESI)+ calculated for $C_{19}H_{19}F_2N_5O_3$, $[M+H]^+$: m/z 404.1529, found 404.2; HPLC(t_R = 4.617 min, 95.4%).

*6-(6-(2,4-difluorophenyl)-2-(methylamino)-7-oxopyrido[2,3-d]pyrimidin-8(7H)-yl)-N-hydroxyhexanamide (**20i**)* ^{1}H NMR (400 MHz, DMSO-d_6), δ(ppm): 10.35 (1H, s), 8.70 (1H, s), 8.62 (1H, s), 7.88 (1H, d, *J* = 4.7 Hz), 7.84 (1H, s), 7.52 (1H, m), 7.30 (1H, m), 7.14 (1H, m), 4.29 (2H, t, *J* = 7.0 Hz), 2.91 (3H, d, *J* = 4.7 Hz), 1.96 (2H, t, *J* = 7.3 Hz), 1.55-1.68 (4H, m), 1.33 (2H, m); ^{13}C-NMR (125 MHz, DMSO-d_6), δ(ppm): 169.5, 163.3, 162.4, 161.4, 159.9, 159.4, 159.3, 155.3, 137.2, 133.4, 121.4, 120.5, 111.5, 104.3, 40.5, 32.6, 28.3, 27.3, 26.6, 25.3; HRMS (ESI)+ calculated for $C_{20}H_{21}F_2N_5O_3$, $[M+H]^+$: m/z 418.1686, found 418.2; HPLC(t_R = 5.280 min, 95.9%).

*4-(6-(2,4-dichlorophenyl)-2-(methylamino)-7-oxopyrido[2,3-d]pyrimidin-8(7H)-yl)-N-hydroxybutanamide (**20j**)* ^{1}H-NMR (400 MHz, DMSO-d_6), δ(ppm): 10.39 (1H, s), 8.71 (1H, s), 8.62 (1H, s), 7.89 (1H, d, *J* = 4.5 Hz), 7.79 (1H, s), 7.69 (1H, d, *J* = 2.0 Hz), 7.48 (1H, dd, *J* = 8.2, 2.0 Hz), 7.43 (1H, d, *J* = 8.2 Hz), 4.32 (2H, t, *J* = 6.8 Hz), 2.92 (3H, d, *J* = 4.5 Hz), 2.04 (2H, t, *J* = 6.9 Hz), 1.91 (2H, m); ^{13}C-NMR (100 MHz, DMSO-d_6), δ(ppm): 169.0, 162.4, 161.3, 159.9, 155.5, 137.1, 135.4, 134.7, 133.7, 133.6, 129.1, 127.6, 124.0, 104.4, 40.4, 30.6, 28.3, 24.1; HRMS (ESI)+ calculated for

$C_{18}H_{17}Cl_2N_5O_3$, $[M+H]^+$: m/z 422.0782, found 422.1; HPLC(t_R = 6.723 min, 95.6%).

*5-(6-(2,4-dichlorophenyl)-2-(methylamino)-7-oxopyrido[2,3-d]pyrimidin-8(7H)-yl)-N-hydroxypentanamide (**20k**)* ^{1}H-NMR (400 MHz, DMSO-d_6), δ(ppm): 10.37 (1H, s), 8.70 (1H, s), 8.63 (1H, s), 7.90 (1H, d, J = 4.7 Hz), 7.79 (1H, s), 7.69 (1H, d, J = 2.0 Hz), 7.48 (1H, dd, J = 8.3 Hz, 2.0 Hz), 7.43 (1H, d, J = 8.3 Hz), 4.30 (2H, t, J = 6.8 Hz), 2.91 (3H, d, J = 4.7 Hz), 2.00 (2H, t, J = 7.3 Hz), 1.54-1.67 (4H, m); ^{13}C-NMR (100 MHz, DMSO-d_6), δ(ppm): 169.3, 162.4, 161.2, 159.9, 155.5, 137.0, 135.4, 134.7, 133.7, 133.6, 129.1, 127.6, 124.0, 104.3, 40.7, 32.6, 28.3, 27.4, 23.4; HRMS (ESI)+ calculated for $C_{19}H_{19}Cl_2N_5O_3$, $[M+H]^+$: m/z 436.0938, found 436.1; HPLC(t_R = 7.547 min, 95.4%).

*6-(6-(2,4-dichlorophenyl)-2-(methylamino)-7-oxopyrido[2,3-d]pyrimidin-8(7H)-yl)-N-hydroxyhexanamide (**20l**)* ^{1}H-NMR (400 MHz, DMSO-d_6), δ(ppm): 10.34 (1H, s), 8.70 (1H, s), 8.62 (1H, s), 7.89 (1H, d, J = 4.6 Hz), 7.78 (1H, s), 7.69 (1H, d, J = 2.0 Hz), 7.48 (1H, dd, J = 8.2, 2.0 Hz), 7.43 (1H, d, J = 8.2 Hz), 4.29 (2H, t, J = 6.7 Hz), 2.91 (3H, d, J = 4.6 Hz), 1.95 (2H, t, J = 7.2 Hz), 1.30-1.69 (6H, m); ^{13}C-NMR (125 MHz, DMSO-d_6), δ(ppm): 169.5, 162.5, 161.2, 159.9, 155.5, 137.0, 135.4, 134.7, 133.7, 133.6, 129.1, 127.6, 124.1, 104.3, 40.5, 32.6, 28.3, 27.3, 26.6, 25.3; HRMS (ESI)+ calculated for $C_{20}H_{21}Cl_2N_5O_3$, $[M+H]^+$: m/z 450.1095, found 450.1; HPLC(t_R = 8.768 min, 98.5%).

*4-(6-(2-chloro-4-fluorophenyl)-2-(methylamino)-7-oxopyrido[2,3-d]pyrimidin-8(7H)-yl)-N-hydroxybutanamide (**20m**)* ^{1}H-NMR (400 MHz, DMSO-d_6), δ(ppm): 10.37 (1H, br s), 8.70 (1H, br s), 8.62 (1H, s), 7.87 (1H, d, J = 4.3 Hz), 7.78 (1H, s), 7.52 (1H, m), 7.45 (1H, m), 7.28 (1H, m), 4.32 (2H, t, J = 6.7 Hz), 2.92 (3H, d, J = 4.3 Hz), 2.04 (2H, t, J = 7.3 Hz), 1.91 (2H, m); ^{13}C-NMR (125 MHz, DMSO-d_6), δ(ppm): 168.9, 162.9, 162.4, 161.4, 159.8, 155.5, 137.0, 134.7, 133.8, 132.9, 124.3, 116.8, 114.7, 104.4, 40.6, 32.0, 28.3, 24.1, 14.4; HRMS (ESI)+ calculated for $C_{18}H_{17}ClFN_5O_3$, $[M+H]^+$: m/z 406.1077, found 406.1; HPLC(t_R = 4.653 min, 99.4%).

*5-(6-(2-chloro-4-fluorophenyl)-2-(methylamino)-7-oxopyrido[2,3-d]pyrimidin-8(7H)-yl)-N-hydroxypentanamide (**20n**)* ^{1}H-NMR (400 MHz, DMSO-d_6), δ(ppm): 10.37 (1H, br s), 8.69 (1H, br s), 8.62 (1H, s), 7.87 (1H, d, J = 4.6 Hz), 7.77 (1H, s), 7.52 (1H, m), 7.45 (1H, m), 7.28 (1H, m), 4.30 (2H, t, J = 6.7 Hz), 2.91 (3H, d, J = 4.6 Hz), 2.00 (2H, t, J = 7.3 Hz), 1.54-1.67 (4H, m); ^{13}C-NMR (125 MHz, DMSO-d_6), δ(ppm): 169.3, 162.9, 162.4, 161.4, 159.8, 155.5, 137.0, 134.7, 133.8, 132.9, 124.3, 116.8, 114.7, 104.4, 40.5, 32.6, 28.3, 27.4, 23.1; HRMS (ESI)+ calculated for $C_{19}H_{19}ClFN_5O_3$, $[M+H]^+$: m/z 420.1234, found 420.1; HPLC(t_R = 5.057 min, 98.2%).

6-(6-(2-chloro-4-fluorophenyl)-2-(methylamino)-7-oxopyrido[2,3-d]pyrimidin-8(7H)-yl)-N-

hydroxyhexanamide ***(20o)*** ^{1}H-NMR (400 MHz, DMSO-d_6), δ(ppm): 10.30 (1H, s), 8.69 (1H, s), 8.62 (1H, s), 7.87 (1H, d, J = 4.7 Hz), 7.77 (1H, s), 7.52 (1H, m), 7.45 (1H, m), 7.28 (1H, m), 4.29 (2H, t, J = 6.8 Hz), 2.91 (3H, d, J = 4.7 Hz), 1.95 (2H, t, J = 7.5 Hz), 1.52-1.67 (4H, m), 1.34 (2H, m); ^{13}C-NMR (125 MHz, DMSO-d_6), δ(ppm): 169.5, 162.9, 162.5, 161.4, 159.8, 155.4, 137.0, 134.6, 133.8, 132.9, 124.3, 116.8, 114.7, 104.4, 40.5, 32.6, 28.3, 27.3, 26.6, 25.3; HRMS (ESI)+ calculated for $C_{20}H_{21}ClFN_5O_3$, $[M+H]^+$: m/z 434.1390, found 434.1; HPLC(t_R = 5.963 min, 95.9%).

4-(2-(ethylamino)-7-oxo-6-phenylpyrido[2,3-d]pyrimidin-8(7H)-yl)-N-hydroxybutanamide ***(21a)*** ^{1}H-NMR (400 MHz, DMSO-d_6), δ(ppm): 10.44 (1H, s), 8.71 (1H, s), 8.65 (1H, s), 7.97 (1H, t, J = 5.4 Hz), 7.92 (1H, s), 7.66 (2H, m), 7.42 (2H, m), 7.33 (1H, m), 4.33 (2H, t, J = 6.1 Hz), 3.40 (2H, m), 2.05 (2H, t, J = 7.0 Hz), 1.91 (2H, m), 1.19 (3H, t, J = 6.9 Hz); ^{13}C-NMR (100 MHz, DMSO-d_6), δ(ppm): 169.0, 162.2, 161.6, 159.9, 137.0, 135.2, 129.0, 129.0, 128.4, 128.4, 127.9, 105.1, 40.6, 36.2, 30.7, 24.2, 14.8; HRMS (ESI)+ calculated for $C_{19}H_{21}N_5O_3$, $[M+H]^+$: m/z 368.1718, found 368.2; HPLC(t_R = 5.298 min, 96.0%).

5-(2-(ethylamino)-7-oxo-6-phenylpyrido[2,3-d]pyrimidin-8(7H)-yl)-N-hydroxypentanamide ***(21b)*** ^{1}H-NMR (400 MHz, DMSO-d_6), δ(ppm): 10.36 (1H, s), 8.70 (1H, s), 8.64 (1H, s), 7.98 (1H, t, J = 5.2 Hz), 7.92 (1H, s), 7.66 (2H, m), 7.42 (2H, m), 7.33 (1H, m), 4.31 (2H, t, J = 6.3 Hz), 3.39 (2H, m), 2.01 (2H, t, J = 7.0 Hz), 1.57-1.70 (4H, m), 1.20 (3H, t, J = 7.0 Hz); ^{13}C-NMR (125 MHz, DMSO-d_6), δ(ppm): 169.3, 162.1, 161.6, 159.9, 137.0, 135.1, 129.0, 129.0, 128.4, 128.4, 127.9, 125.8, 105.1, 40.6, 36.2, 32.6, 27.4, 23.5, 14.8; HRMS (ESI)+ calculated for $C_{20}H_{23}N_5O_3$, $[M+H]^+$: m/z 382.1874, found 382.2; HPLC(t_R = 5.954 min, 96.4%).

6-(2-(ethylamino)-7-oxo-6-phenylpyrido[2,3-d]pyrimidin-8(7H)-yl)-N-hydroxyhexanamide ***(21c)*** ^{1}H-NMR (400 MHz, DMSO-d_6), δ(ppm): 10.35 (1H, s), 8.69 (1H, s), 8.63 (1H, s), 7.98 (1H, t, J = 5.1 Hz), 7.91 (1H, s), 7.65 (2H, m), 7.41 (2H, m), 7.33 (1H, m), 4.29 (2H, t, J = 6.6 Hz), 3.39 (2H, m), 1.97 (2H, t, J = 7.3 Hz), 1.53-1.66 (4H, m), 1.34 (2H, m), 1.20 (3H, t, J = 6.9 Hz); ^{13}C-NMR (100 MHz, DMSO-d_6), δ(ppm): 169.5, 162.0, 161.6, 159.8, 154.9, 137.0, 135.0, 129.0, 129.0, 128.4, 128.4, 127.8, 125.8, 40.6, 36.2, 32.7, 27.5, 26.7, 25.4, 14.8; HRMS (ESI)+ calculated for $C_{21}H_{25}N_5O_3$, $[M+H]^+$: m/z 396.2031, found 396.2; HPLC(t_R = 7.058 min, 98.8%).

4-(2-(ethylamino)-6-(4-fluorophenyl)-7-oxopyrido[2,3-d]pyrimidin-8(7H)-yl)-N-hydroxybutanamide ***(21d)*** ^{1}H-NMR (400 MHz, DMSO-d_6), δ(ppm): 10.35 (1H, s), 8.68 (1H, s), 8.63 (1H, s), 7.99 (1H, t, J = 5.1 Hz), 7.93 (1H, s), 7.71 (2H, m), 7.24 (2H, m), 4.33 (2H, t, J = 6.5 Hz), 3.40 (2H,

m), 2.04 (2H, t, *J* = 6.8 Hz), 1.91 (2H, m), 1.19 (3H, t, *J* = 7.0 Hz); ^{13}C-NMR (125 MHz, DMSO-d_6), δ(ppm): 169.0, 163.0, 161.6, 161.0, 159.9, 155.0, 135.1, 133.3, 131.1, 131.0, 124.7, 115.3, 115.1, 105.0, 40.5, 36.2, 30.7, 24.2, 14.8; HRMS (ESI)+ calculated for $C_{19}H_{20}FN_5O_3$, $[M+H]^+$: m/z 386.1623, found 386.2; HPLC(t_R = 5.561 min, 95.2%).

5-(2-(ethylamino)-6-(4-fluorophenyl)-7-oxopyrido[2,3-d]pyrimidin-8(7H)-yl)-N-hydroxypentanamide ***(21e)*** ^{1}H-NMR (400 MHz, DMSO-d_6), δ(ppm): 9.63 (1H, br s), 8.68 (1H, s), 8.63 (1H, s), 7.92 (2H, br s), 7.70 (2H, m), 7.22 (2H, m), 4.30 (2H, t, *J* = 6.5 Hz), 3.40 (2H, m), 2.00 (2H, t, *J* = 6.8 Hz), 1.56-1.64 (4H, m), 1.19 (3H, t, *J* = 6.9 Hz); ^{13}C-NMR (125 MHz, DMSO-d_6), δ(ppm): 169.2, 163.0, 162.0, 161.6, 159.8, 155.0, 135.0, 133.3, 131.1, 131.0, 124.7, 115.3, 115.1, 105.0, 40.5, 37.3, 36.2, 28.1, 24.2, 14.8; HRMS (ESI)+ calculated for $C_{20}H_{22}FN_5O_3$, $[M+H]^+$: m/z 400.1780, found 400.2; HPLC(t_R = 6.382 min, 99.3%).

*6-(2-(ethylamino)-6-(4-fluorophenyl)-7-oxopyrido[2,3-d]pyrimidin-8(7H)-yl)-N-hydroxyhexanamide **(21f)*** ^{1}H-NMR (400 MHz, DMSO-d_6), δ(ppm): 10.34 (1H, s), 8.68 (1H, s), 8.63 (1H, s), 7.98 (1H, t, *J* = 5.1 Hz), 7.91 (1H, s), 7.71 (2H, m), 7.23 (2H, m), 4.29 (2H, t, *J* = 6.7 Hz), 3.39 (2H, m), 1.96 (2H, t, *J* = 7.3 Hz), 1.53-1.66 (4H, m), 1.34 (2H, m), 1.19 (3H, t, *J* = 7.1 Hz); ^{13}C-NMR (125 MHz, DMSO-d_6), δ(ppm): 169.5, 162.9, 161.7, 161.0, 159.8, 154.9, 135.0, 133.3, 131.1, 131.0, 124.7, 115.3, 115.1, 105.0, 40.5, 36.2, 32.6, 27.5, 26.7, 25.4, 14.8; HRMS (ESI)+ calculated for $C_{21}H_{24}FN_5O_3$, $[M+H]^+$: m/z 414.1936, found 414.2; HPLC(t_R = 7.386 min, 96.9%).

*4-(6-(2,4-difluorophenyl)-2-(ethylamino)-7-oxopyrido[2,3-d]pyrimidin-8(7H)-yl)-N-hydroxybutanamide **(21g)*** ^{1}H-NMR (400 MHz, DMSO-d_6), δ(ppm): 10.39 (1H, s), 8.69 (1H, s), 8.63 (1H, s), 7.97 (1H, t, *J* = 5.2 Hz), 7.85 (1H, s), 7.52 (1H, m), 7.30 (1H, m), 7.15 (1H, m), 4.31 (2H, t, *J* = 6.8 Hz), 3.41 (2H, m), 2.04 (2H, t, *J* = 7.1 Hz), 1.90 (2H, m), 1.19 (3H, t, *J* = 7.0 Hz); ^{13}C-NMR (125 MHz, DMSO-d_6), δ(ppm): 168.9, 163.3, 161.8, 161.5, 161.4, 160.0, 159.4, 155.4, 137.2, 133.4, 121.5, 120.4, 111.7, 104.4, 40.6, 36.2, 30.7, 24.2, 14.8; HRMS (ESI)+ calculated for $C_{19}H_{19}F_2N_5O_3$, $[M+H]^+$: m/z 404.1529, found 404.1; HPLC(t_R = 4.981 min, 98.0%).

*5-(6-(2,4-difluorophenyl)-2-(ethylamino)-7-oxopyrido[2,3-d]pyrimidin-8(7H)-yl)-N-hydroxypentanamide **(21h)*** ^{1}H-NMR (400 MHz, DMSO-d_6), δ(ppm): 10.35 (1H, s), 8.69 (1H, s), 8.63 (1H, s), 7.98 (1H, t, *J* = 5.2 Hz), 7.84 (1H, s), 7.51 (1H, m), 7.30 (1H, m), 7.15 (1H, m), 4.28 (2H, t, *J* = 6.8 Hz), 3.40 (2H, m), 2.00 (2H, t, *J* = 7.0 Hz), 1.56-1.65 (4H, m), 1.20 (3H, t, *J* = 7.1 Hz); ^{13}C NMR (125 MHz, DMSO-d_6), δ(ppm): 169.3, 163.3, 161.8, 161.4, 161.3, 160.0, 159.4, 155.3, 137.2, 133.3,

121.3, 120.5, 111.5, 104.3, 40.6, 36.2, 32.6, 27.6, 23.4, 14.8; HRMS (ESI)+ calculated for $C_{20}H_{21}F_2N_5O_3$, $[M+H]^+$: m/z 418.1686, found 418.2; HPLC(t_R = 5.662 min, 96.2%).

*6-(6-(2,4-difluorophenyl)-2-(ethylamino)-7-oxopyrido[2,3-d]pyrimidin-8(7H)-yl)-N-hydroxyhexanamide **(21i)*** ^{1}H-NMR (400 MHz, DMSO-d_6), δ(ppm): 10.35 (1H, s), 8.68 (1H, s), 8.63 (1H, s), 7.98 (1H, t, J = 5.1 Hz), 7.84 (1H, s), 7.51 (1H, m), 7.30 (1H, m), 7.14 (1H, m), 4.27 (2H, t, J = 6.9 Hz), 3.39 (2H, m), 1.96 (2H, t, J = 7.3 Hz), 1.52-1.65 (4H, m), 1.33 (2H, m), 1.20 (3H, t, J = 7.1 Hz); ^{13}C-NMR (125 MHz, DMSO-d_6), δ(ppm): 169.4, 163.2, 161.8, 161.4, 161.3, 160.0, 159.4, 155.3, 137.1, 133.4, 121.4, 120.5, 111.5, 104.3, 40.5, 36.2, 32.6, 27.4, 26.7, 25.3, 14.8; HRMS (ESI)+ calculated for $C_{21}H_{23}F_2N_5O_3$, $[M+H]^+$: m/z 432.1842, found 432.2; HPLC(t_R = 6.545 min, 97.6%).

*4-(6-(2,4-dichlorophenyl)-2-(ethylamino)-7-oxopyrido[2,3-d]pyrimidin-8(7H)-yl)-N-hydroxybutanamide **(21j)*** ^{1}H-NMR (400 MHz, DMSO-d_6), δ(ppm): 10.42 (1H, s), 8.68 (1H, s), 8.63 (1H, s), 7.98 (1H, t, J = 5.1 Hz), 7.78 (1H, s), 7.69 (1H, d, J = 2.0 Hz), 7.46 (1H, dd, J = 8.2, 2.0 Hz), 7.25 (1H, d, J = 8.2 Hz), 4.29 (2H, t, J = 6.8 Hz), 3.50 (2H, m), 2.02 (2H, t, J = 7.1 Hz), 1.89 (2H, t, J = 7.1 Hz), 1.23 (3H, t, J = 7.1 Hz); ^{13}C-NMR (100 MHz, DMSO-d_6), δ(ppm): 169.3, 161.8, 161.2, 160.0, 155.5, 137.0, 134.7, 133.7, 133.6, 129.1, 127.6, 124.0, 104.4, 40.6, 36.2, 31.6, 23.3, 14.8; HRMS (ESI)+ calculated for $C_{19}H_{19}Cl_2N_5O_3$, $[M+H]^+$: m/z 436.0938, found 436.1; HPLC(t_R = 8.753 min, 99.1%).

*5-(6-(2,4-dichlorophenyl)-2-(ethylamino)-7-oxopyrido[2,3-d]pyrimidin-8(7H)-yl)-N-hydroxypentanamide **(21k)*** ^{1}H-NMR (400 MHz, DMSO-d_6), δ(ppm): 10.33 (1H, s), 8.68 (1H, s), 8.63 (1H, s), 7.99 (1H, t, J = 5.1 Hz), 7.78 (1H, s), 7.69 (1H, d, J = 2.0 Hz), 7.48 (1H, dd, J = 8.2, 2.0 Hz), 7.42 (1H, d, J = 8.2 Hz), 4.27 (2H, t, J = 6.8 Hz), 3.40 (2H, m), 1.99 (2H, t, J = 7.1 Hz), 1.55-1.65 (4H, m), 1.20 (3H, t, J = 7.1 Hz); ^{13}C-NMR (100 MHz, DMSO-d_6), δ(ppm): 169.3, 161.8, 161.2, 160.0, 155.5, 137.0, 134.8, 133.7, 133.6, 129.1, 127.6, 124.0, 104.4, 40.6, 36.2, 32.6, 27.5, 23.3, 14.8; HRMS (ESI)+ calculated for $C_{20}H_{21}Cl_2N_5O_3$, $[M+H]^+$: m/z 450.1095, found 450.1; HPLC(t_R = 9.878 min, 99.8%).

*6-(6-(2,4-dichlorophenyl)-2-(ethylamino)-7-oxopyrido[2,3-d]pyrimidin-8(7H)-yl)-N-hydroxyhexanamide **(21l)*** ^{1}H-NMR (400 MHz, DMSO-d_6), δ(ppm): 10.33 (1H, s), 8.68 (1H, s), 8.62 (1H, s), 7.99 (1H, t, J = 4.8 Hz), 7.78 (1H, s), 7.69 (1H, d, J = 2.0 Hz), 7.48 (1H, dd, J = 8.2, 2.0 Hz), 7.42 (1H, d, J = 8.2 Hz), 4.27 (2H, t, J = 7.0 Hz), 3.40 (2H, m), 1.95 (2H, t, J = 7.3 Hz), 1.52-1.66 (4H, m), 1.34 (2H, m), 1.20 (3H, t, J = 7.1 Hz); ^{13}C-NMR (100 MHz, DMSO-d_6), δ(ppm): 169.5, 161.8, 161.2, 160.0, 155.4, 137.0, 135.4, 134.7, 133.7, 133.6, 129.1, 127.6, 124.0, 104.4, 40.6, 36.2, 32.6, 27.5, 26.6, 25.3, 14.8; HRMS (ESI)+ calculated for $C_{21}H_{23}Cl_2N_5O_3$, $[M+H]^+$: m/z 464.1251, found 464.1; HPLC(t_R

= 11.889 min, 97.6%).

4-(6-(2-chloro-4-fluorophenyl)-2-(ethylamino)-7-oxopyrido[2,3-d]pyrimidin-8(7H)-yl)-N-hydroxybutanamide ***(21m)*** ^{1}H-NMR (400 MHz, DMSO-d_6), δ(ppm): 10.36 (1H, s), 8.68 (1H, s), 8.62 (1H, s), 7.97 (1H, t, J = 5.4 Hz), 7.76 (1H, s), 7.52 (1H, m), 7.45 (1H, m), 7.28 (1H, m), 4.29 (2H, t, J = 7.0 Hz), 3.42 (2H, m), 2.07 (2H, t, J = 7.1 Hz), 1.89 (2H, m), 1.18 (3H, t, J = 7.0 Hz); ^{13}C NMR (125 MHz, DMSO-d_6), δ(ppm): 168.9, 162.9, 161.8, 160.9, 159.9, 155.5, 137.0, 134.6, 133.8, 132.9, 124.2, 117.0, 114.5, 104.4, 40.5, 36.2, 30.6, 24.2, 14.8; HRMS (ESI)+ calculated for $C_{19}H_{19}ClFN_5O_3$, $[M+H]^+$: m/z 420.1234, found 420.1; HPLC(t_R = 5.759 min, 98.7%).

5-(6-(2-chloro-4-fluorophenyl)-2-(ethylamino)-7-oxopyrido[2,3-d]pyrimidin-8(7H)-yl)-N-hydroxypentanamide ***(21n)*** ^{1}H-NMR (400 MHz, DMSO-d_6), δ(ppm): 10.36 (1H, s), 8.68 (1H, s), 8.62 (1H, s), 7.97 (1H, t, J = 5.4 Hz), 7.76 (1H, s), 7.52 (1H, m), 7.45 (1H, m), 7.28 (1H, m), 4.28 (2H, t, J = 7.0 Hz), 3.39 (2H, m), 1.99 (2H, t, J = 7.1 Hz), 1.55-1.65 (4H, m), 1.20 (3H, t, J = 7.0 Hz); ^{13}C-NMR (125 MHz, DMSO-d_6), δ(ppm): 169.3, 162.9, 161.8, 161.4, 159.9, 155.4, 137.0, 134.6, 133.8, 132.9, 124.2, 117.0, 114.5, 104.4, 40.6, 36.2, 32.6, 27.5, 23.3, 14.8; HRMS (ESI)+ calculated for $C_{20}H_{21}ClFN_5O_3$, $[M+H]^+$: m/z 434.1390, found 434.1; HPLC(t_R = 6.467 min, 99.6%).

*6-(6-(2-chloro-4-fluorophenyl)-2-(ethylamino)-7-oxopyrido[2,3-d]pyrimidin-8(7H)-yl)-N-hydroxyhexanamide****(21o)*** ^{1}H-NMR (400 MHz, DMSO-d_6), δ(ppm): 10.44 (1H, s), 8.65 (1H, s), 8.62 (1H, s), 7.97 (1H, t, J = 5.4 Hz), 7.76 (1H, s), 7.52 (1H, m), 7.45 (1H, m), 7.28 (1H, m), 4.23 (2H, t, J = 7.0 Hz), 3.44 (2H, m), 1.99 (2H, t, J = 7.1 Hz), 1.64 (2H, m), 1.53 (2H, m),, 1.18 (3H, t, J = 7.0 Hz); ^{13}C-NMR (125 MHz, DMSO-d_6), δ(ppm): 169.3, 162.9, 161.8, 161.4, 159.9, 155.4, 136.9, 134.6, 133.8, 132.9, 124.2, 117.0, 114.5, 104.4, 40.6, 36.2, 32.6, 27.5, 25.4, 21.5, 14.8; HRMS (ESI)+ calculated for $C_{21}H_{23}ClFN_5O_3$, $[M+H]^+$: m/z 448.1547, found 448.2; HPLC(t_R = 7.553 min, 95.7%).

4-(2-(cyclopropylamino)-7-oxo-6-phenylpyrido[2,3-d]pyrimidin-8(7H)-yl)-N-hydroxybutanamide ***(22a)*** ^{1}H-NMR (400 MHz, DMSO-d_6), δ(ppm): 10.42 (1H, br s), 8.65 (2H, br s), 8.07 (1H, br s), 7.94 (1H, s), 7.66 (2H, m), 7.41 (2H, m), 7.34 (1H, m), 4.37 (2H, br s), 2.85 (1H, m), 2.20 (2H, br s), 1.94 (2H, m), 0.77 (2H, m), 0.55 (2H, m); ^{13}C-NMR (125 MHz, DMSO-d_6), δ(ppm): 169.0, 162.9, 162.2, 159.7, 154.9, 137.0, 135.0, 129.1, 129.1, 128.4, 128.4, 127.9, 126.3, 105.5, 40.6, 24.4, 24.2, 6.7; HRMS (ESI)+ calculated for $C_{20}H_{21}N_5O_3$, $[M+H]^+$: m/z 380.1718, found 380.1; HPLC(t_R = 5.403 min, 99.7%).

5-(2-(cyclopropylamino)-7-oxo-6-phenylpyrido[2,3-d]pyrimidin-8(7H)-yl)-N-hydroxypentanamide ***(22b)*** ^{1}H-NMR (400 MHz, DMSO-d_6), δ(ppm): 10.36 (1H, s), 8.65 (2H, br s), 8.08 (1H, br s), 7.94 (1H,

s), 7.66 (2H, m), 7.41 (2H, m), 7.34 (1H, m), 4.35 (2H, br s), 2.83 (1H, br s), 2.01 (2H, t, *J* = 7.2 Hz), 1.58-1.68 (4H, m), 0.75 (2H, m), 0.56 (2H, m); ^{13}C-NMR (125 MHz, DMSO-d_6), δ(ppm): 170.8, 169.4, 162.9, 162.2, 159.6, 154.9, 137.0, 135.0, 129.1, 129.1, 128.4, 128.4, 127.9, 126.3, 105.5, 32.6, 27.5, 24.4, 23.4, 21.2, 14.5, 6.7; HRMS (ESI)+ calculated for $C_{21}H_{23}N_5O_3$, $[M+H]^+$: m/z 394.1874, found 394.2; HPLC(t_R = 6.171 min, 99.5%).

*6-(2-(cyclopropylamino)-7-oxo-6-phenylpyrido[2,3-d]pyrimidin-8(7H)-yl)-N-hydroxyhexanamide (**22c**)* ^{1}H-NMR (400 MHz, DMSO-d_6), δ(ppm): 10.39 (1H, s), 8.71 (1H, s), 8.65 (1H, s), 8.09 (1H, br s), 7.93 (1H, s), 7.66 (2H, m), 7.41 (2H, m), 7.34 (1H, m), 4.34 (2H, br s), 2.82 (1H, br s), 1.97 (2H, t, *J* = 7.3 Hz), 1.53-1.71 (4H, m), 1.35 (2H, m), 0.75 (2H, m), 0.56 (2H, m); ^{13}C-NMR (125 MHz, DMSO-d_6), δ(ppm): 169.5, 162.9, 162.0, 159.5, 154.9, 137.0, 135.0, 129.1, 129.1, 128.4, 128.4, 127.9, 126.3, 105.5, 40.6, 32.7, 27.4, 26.7, 25.4, 24.4, 6.7; HRMS (ESI)+ calculated for $C_{22}H_{25}N_5O_3$, $[M+H]^+$: m/z 408.2031, found 408.2; HPLC(t_R = 7.361 min, 95.4%).

*4-(2-(cyclopropylamino)-6-(4-fluorophenyl)-7-oxopyrido[2,3-d]pyrimidin-8(7H)-yl)-N-hydroxybutanamide (**22d**)* ^{1}H-NMR (400 MHz, DMSO-d_6), δ(ppm): 10.46 (1H, s), 8.71 (1H, s), 8.63 (1H, s), 8.05 (1H, br s), 7.92 (1H, s), 7.30 (1H, m), 7.29 (1H, m), 7.23 (1H, m), 4.36 (2H, br s), 2.84 (1H, m), 2.05 (2H, m), 1.94 (1H, m), 0.76 (2H, m), 0.54 (2H, m); ^{13}C NMR (125 MHz, DMSO-d_6), δ(ppm): 168.9, 163.0, 162.9, 162.1, 161.0, 159.5, 154.9, 135.0, 133.3, 131.1, 131.0, 125.1, 115.3, 115.1, 40.5, 34.8, 29.1, 24.2, 6.7; HRMS (ESI)+ calculated for $C_{20}H_{20}FN_5O_3$, $[M+H]^+$: m/z 398.1623, found 398.1; HPLC (t_R = 5.558 min, 99.8%).

*5-(2-(cyclopropylamino)-6-(4-fluorophenyl)-7-oxopyrido[2,3-d]pyrimidin-8(7H)-yl)-N-hydroxypentanamide (**22e**)* ^{1}H-NMR (400 MHz, DMSO-d_6), δ(ppm): 10.43 (1H, s), 8.72 (1H, s), 8.64 (1H, s), 8.09 (1H, br s), 7.94 (1H, s), 7.71 (1H, m), 7.70 (1H, m), 7.25 (1H, m), 7.20 (1H, m), 4.36 (2H, br s), 2.84 (1H, m), 2.05 (2H, m), 1.94 (1H, m), 0.76 (2H, m), 0.54 (2H, m); ^{13}C NMR (125 MHz, DMSO-d_6), δ(ppm): 169.4, 163.4, 163.1, 162.0, 161.0, 159.5, 154.9, 135.0, 133.3, 131.1, 131.0, 125.1, 115.3, 115.1, 40.6, 32.6, 27.5, 27.3, 24.4, 6.7; HRMS (ESI)+ calculated for $C_{21}H_{22}FN_5O_3$, $[M+H]^+$: m/z 412.1780, found 412.2; HPLC(t_R = 6.656 min, 98.4%).

*6-(2-(cyclopropylamino)-6-(4-fluorophenyl)-7-oxopyrido[2,3-d]pyrimidin-8(7H)-yl)-N-hydroxyhexanamide (**22f**)* ^{1}H-NMR (400 MHz, DMSO-d_6), δ(ppm): 10.40 (1H, s), 8.71 (1H, s), 8.63 (1H, s), 8.08 (1H, br s), 7.93 (1H, s), 7.72 (1H, m), 7.70 (1H, m), 7.25 (1H, m), 7.21 (1H, m), 4.33 (2H, br s), 2.81 (1H, m), 2.02 (2H, m), 1.69 (2H, m), 1.55 (2H, m), 0.74 (2H, m), 0.55 (2H, m); ^{13}C-NMR (125

MHz, DMSO-d_6), δ(ppm): 169.4, 162.9, 162.0, 161.0, 159.5, 154.9, 135.0, 133.3, 131.1, 131.0, 125.1, 115.3, 115.1, 40.6, 27.4, 26.9, 25.5, 24.4, 6.7; HRMS (ESI)+ calculated for $C_{22}H_{24}FN_5O_3$, $[M+H]^+$: m/z 426.1936, found 426.1; HPLC(t_R = 7.992 min, 97.8%).

*4-(2-(cyclopropylamino)-6-(2,4-difluorophenyl)-7-oxopyrido[2,3-d]pyrimidin-8(7H)-yl)-N-hydroxybutanamide (**22g**)* ^{1}H-NMR (400 MHz, DMSO-d_6), δ(ppm): 10.39 (1H, s), 8.71 (1H, s), 8.64 (1H, s), 8.15 (1H, br s), 7.87 (1H, s), 7.52 (1H, m), 7.30 (1H, m), 7.15 (1H, m), 4.35 (2H, br s), 2.85 (1H, m), 1.93-2.06 (4H, m), 0.78 (2H, m), 0.55 (2H, m); ^{13}C-NMR (125 MHz, DMSO-d_6), δ(ppm): 168.3, 162.7, 162.5, 160.8, 160.7, 159.0, 158.8, 154.7, 136.5, 132.8, 120.8, 120.3, 111.1, 103.7, 40.0, 31.0, 29.7, 23.6, 6.1; HRMS (ESI)+ calculated for $C_{20}H_{19}F_2N_5O_3$, $[M+H]^+$: m/z 416.1529, found 416.2; HPLC(t_R = 5.202 min, 95.8%).

*5-(2-(cyclopropylamino)-6-(2,4-difluorophenyl)-7-oxopyrido[2,3-d]pyrimidin-8(7H)-yl)-N-hydroxypentanamide (**22h**)* ^{1}H NMR (400 MHz, DMSO-d_6), δ(ppm): 10.35 (1H, s), 8.66 (1H, s), 8.64 (1H, s), 8.14 (1H, br s), 7.86 (1H, s), 7.52 (1H, m), 7.30 (1H, m), 7.15 (1H, m), 4.33 (2H, br s), 2.82 (1H, br s), 2.00 (2H, t, J = 7.3 Hz), 1.57-1.68 (4H, m), 0.75 (2H, m), 0.56 (2H, m); ^{13}C-NMR (125 MHz, DMSO-d_6), δ(ppm): 169.4, 163.1, 161.4, 160.0, 158.8, 155.3, 137.1, 133.4, 120.8, 120.3, 111.7, 104.4, 40.0, 32.6, 27.5, 24.4, 6.7; HRMS (ESI)+ calculated for $C_{21}H_{21}F_2N_5O_3$, $[M+H]^+$: m/z 430.1686, found 430.1; HPLC(t_R = 5.827 min, 98.3%).

*6-(2-(cyclopropylamino)-6-(2,4-difluorophenyl)-7-oxopyrido[2,3-d]pyrimidin-8(7H)-yl)-N-hydroxyhexanamide (**22i**)* ^{1}H NMR (400 MHz, DMSO-d_6), δ(ppm): 10.40 (1H, s), 8.70 (1H, s), 8.63 (1H, s), 8.13 (1H, br s), 7.84 (1H, s), 7.52 (1H, m), 7.30 (1H, m), 7.14 (1H, m), 4.26 (2H, br s), 2.82 (1H, br s), 2.00 (2H, t, J = 7.3 Hz), 1.67 (2H, m), 1.55 (2H, m), 1.34 (2H, m), 0.75 (2H, m), 0.56 (2H, m); ^{13}C-NMR (125 MHz, DMSO-d_6), δ(ppm): 163.3, 163.1, 161.4, 159.6, 159.4, 159.3, 155.3, 137.0, 133.4, 121.3, 121.0, 111.5, 104.3, 40.5, 32.7, 27.4, 26.7, 25.4, 24.4, 6.7; HRMS (ESI)+ calculated for $C_{22}H_{23}F_2N_5O_3$, $[M+H]^+$: m/z 444.1842, found 444.2; HPLC(t_R = 6.805 min, 99.6%).

*4-(2-(cyclopropylamino)-6-(2,4-dichlorophenyl)-7-oxopyrido[2,3-d]pyrimidin-8(7H)-yl)-N-hydroxybutanamide (**22j**)* ^{1}H-NMR (400 MHz, DMSO-d_6), δ(ppm): 10.44 (1H, s), 8.72 (1H, s), 8.63 (1H, s), 8.11 (1H, br s), 7.79 (1H, s), 7.69 (1H, d, J = 2.0 Hz), 7.49 (1H, dd, J = 2.0, 8.3 Hz), 7.43 (1H, d, J = 8.3 Hz), 4.33 (2H, br s), 2.82 (1H, br s), 2.06 (2H, t, J = 7.4 Hz), 1.89 (2H, m), 0.75 (2H, m), 0.54 (2H, m); ^{13}C-NMR (125 MHz, DMSO-d_6), δ(ppm): 168.9, 163.1, 161.3, 159.7, 155.4, 136.9, 135.4, 134.7, 133.7, 133.6, 129.1, 127.6, 124.5, 104.9, 40.6, 31.6, 24.4, 24.2, 6.7; HRMS $(ESI)^+$ calculated for

$C_{20}H_{19}Cl_2N_5O_3$, $[M+H]^+$: m/z 448.0938, found 448.1; HPLC(t_R = 8.589 min, 96.9%).

*5-(2-(cyclopropylamino)-6-(2,4-dichlorophenyl)-7-oxopyrido[2,3-d]pyrimidin-8(7H)-yl)-N-hydroxypentanamide **(22k)*** ^{1}H-NMR (400 MHz, DMSO-d_6), δ(ppm): 10.41 (1H, s), 8.71 (1H, s), 8.63 (1H, s), 8.13 (1H, br s), 7.79 (1H, s), 7.69 (1H, d, J = 2.0 Hz), 7.49 (1H, dd, J = 2.0, 8.3 Hz), 7.43 (1H, d, J = 8.3 Hz), 4.26 (2H, br s), 2.83 (1H, br s), 2.02 (2H, t, J = 7.4 Hz), 1.68 (2H, m), 1.55 (2H, m), 0.75 (2H, m), 0.56 (2H, m); ^{13}C-NMR (125 MHz, DMSO-d_6), δ(ppm): 169.3, 163.1, 161.2, 159.7, 155.4, 136.8, 135.4, 134.7, 133.7, 133.6, 129.1, 127.6, 124.5, 40.5, 31.6, 30.3, 27.9, 24.4, 6.7; HRMS (ESI)+ calculated for $C_{21}H_{21}Cl_2N_5O_3$, $[M+H]^+$: m/z 462.1095, found 462.1; HPLC(t_R = 9.989 min, 98.6%).

*6-(2-(cyclopropylamino)-6-(2,4-dichlorophenyl)-7-oxopyrido[2,3-d]pyrimidin-8(7H)-yl)-N-hydroxyhexanamide **(22l)*** ^{1}H-NMR (400 MHz, DMSO-d_6), δ(ppm): 10.35 (1H, s), 8.71 (1H, s), 8.63 (1H, s), 8.16 (1H, br s), 7.79 (1H, s), 7.69 (1H, d, J = 2.0 Hz), 7.49 (1H, dd, J = 2.0, 8.3 Hz), 7.43 (1H, d, J = 8.3 Hz), 4.31 (2H, br s), 2.82 (1H, br s), 1.95 (2H, t, J = 7.4 Hz), 1.52-1.69 (4H, m), 1.34 (2H, m), 0.75 (2H, m), 0.57 (2H, m); ^{13}C-NMR (125 MHz, DMSO-d_6), δ(ppm): 169.4, 163.1, 161.2, 159.7, 155.4, 136.9, 135.4, 134.7, 133.7, 133.6, 129.1, 127.6, 124.5, 104.9, 40.6, 32.7, 27.4, 26.6, 25.4, 24.4, 6.7; HRMS (ESI)+ calculated for $C_{22}H_{23}Cl_2N_5O_3$, $[M+H]^+$: m/z 476.1251, found 476.1; HPLC(t_R = 12.236 min, 99.8%).

*4-(6-(2-chloro-4-fluorophenyl)-2-(cyclopropylamino)-7-oxopyrido[2,3-d]pyrimidin-8(7H)-yl)-N-hydroxybutanamide **(22m)*** ^{1}H-NMR (400 MHz, DMSO-d_6), δ(ppm): 10.44 (1H, s), 8.69 (1H, s), 8.62 (1H, s), 8.12 (1H, br s), 7.77 (1H, s), 7.51 (1H, d, J = 2.0 Hz), 7.45 (1H, dd, J = 2.0, 8.3 Hz), 7.27 (1H, d, J = 8.3 Hz), 4.27 (2H, br s), 2.82 (1H, br s), 1.67 (2H, m), 1.55 (2H, m), 0.75 (2H, m), 0.56 (2H, m); ^{13}C-NMR (125 MHz, DMSO-d_6), δ(ppm): 169.2, 163.1, 161.4, 160.9, 159.6, 155.3, 136.8, 134.6, 133.8, 132.9, 124.7, 116.9, 114.5, 40.5, 32.6, 27.4, 24.4, 6.7; HRMS (ESI)+ calculated for $C_{20}H_{19}ClFN_5O_3$, $[M+H]^+$: m/z 432.1234, found 432.1; HPLC(t_R = 5.849 min, 98.6%).

*5-(6-(2-chloro-4-fluorophenyl)-2-(cyclopropylamino)-7-oxopyrido[2,3-d]pyrimidin-8(7H)-yl)-N-hydroxypentanamide **(22n)*** ^{1}H-NMR (400 MHz, DMSO-d_6), δ(ppm): 10.49 (1H, s), 8.69 (1H, s), 8.62 (1H, s), 8.13 (1H, br s), 7.77 (1H, s), 7.51 (1H, d, J = 2.0 Hz), 7.45 (1H, dd, J = 2.0, 8.3 Hz), 7.27 (1H, d, J = 8.3 Hz), 4.25 (2H, br s), 2.82 (1H, br s), 1.97 (2H, m), 1.67 (2H, m), 1.55 (2H, m), 0.75 (2H, m), 0.56 (2H, m); ^{13}C-NMR (125 MHz, DMSO-d_6), δ(ppm): 169.5, 163.1, 161.4, 160.9, 159.6, 155.3, 136.8, 134.6, 133.8, 132.9, 124.7, 116.9, 114.5, 40.5, 32.6, 27.4, 27.1, 24.4, 6.7; HRMS (ESI)+ calculated for $C_{21}H_{21}ClFN_5O_3$, $[M+H]^+$: m/z 446.1390, found 446.2; HPLC(t_R = 6.668 min, 99.6%).

*6-(6-(2-chloro-4-fluorophenyl)-2-(cyclopropylamino)-7-oxopyrido[2,3-d]pyrimidin-8(7H)-yl)-N-hydroxyhexanamide (**22o**)* ^{1}H-NMR (400 MHz, DMSO-d_6), δ(ppm): 10.43 (1H, s), 8.70 (1H, s), 8.62 (1H, s), 8.12 (1H, br s), 7.77 (1H, s), 7.51 (1H, d, *J* = 2.0 Hz), 7.45 (1H, dd, *J* = 2.0, 8.3 Hz), 7.27 (1H, d, *J* = 8.3 Hz), 4.24 (2H, br s), 2.81 (1H, br s), 1.95 (2H, m), 1.54 (2H, m), 1.33 (2H, m), 0.75 (2H, m), 0.56 (2H, m); ^{13}C-NMR (125 MHz, DMSO-d_6), δ(ppm): 169.2, 163.1, 161.4, 160.9, 159.6, 155.4, 136.8, 134.6, 133.7, 132.9, 124.7, 116.8, 114.7, 40.5, 32.6, 31.6, 30.3, 27.8, 24.4, 6.7; HRMS (ESI)+ calculated for $C_{22}H_{23}ClFN_5O_3$, $[M+H]^+$: m/z 460.1547, found 460.2; HPLC(t_R = 7.862 min, 98.3%).

Synthesis of intermediate 27. To a mixture of methyl 2-(4-bromo-2-chlorophenyl)acetate (1 eq), bis (pinacolato) diboron (1 eq), potassium acetate (3 eq), and [1,1'-Bis(diphenylphosphino)ferrocene]dichloropalladium(II) (0.1 eq) in 1,4-dioxane was stirred for 18 h at 80 °C under anhydrous and anaerobic conditions. Without any further purification or characterization, 2-bromo-6-methylpyridine (1 eq), potassium carbonate (3 eq), H2O (4 ml), tetrakis(triphenylphosphine)palladium (0.1 eq) was directly added and the resulting mixture was stirred for additional 18 h at 80 °C continually. After completion, the mixture was diluted with 20 ml water and extracted with ethyl acetate (3 × 20 ml). The combined organic layers were washed with saturated aqueous sodium bicarbonate and brine, and then dried over anhydrous sodium sulfate. After removing the solvent under reduced pressure, the crude product was purified by flash chromatography on silica gel, eluting with petroleum ether/ethyl acetate (20 : 1) as a pale-yellow oil, yield 40%.

*methyl 2-(2-chloro-4-(6-methylpyridin-2-yl)phenyl)acetate (**27**)* ^{1}H-NMR (600 MHz, $CDCl_3$), δ(ppm): 8.05 (1H, d, *J* = 1.8 Hz), 7.82 (1H, dd, *J* = 7.9, 1.8 Hz), 7.63 (1H, t, *J* = 7.7 Hz), 7.48 (1H, dd, *J* = 7.8, 0.9 Hz), 7.37 (1H, d, *J* = 8.0 Hz), 7.11 (1H, d, *J* = 7.6 Hz), 3.82 (2H, s), 3.72 (3H, s), 2.62 (3H, s); ^{13}C-NMR (150 MHz, $CDCl_3$), δ(ppm): 171.1, 158.7, 155.2, 140.6, 137.2, 135.2, 132.7, 131.7, 128.1, 125.5, 122.3, 117.7, 52.3, 38.9, 24.8.

Synthesis of intermediate 28. To a mixture of methyl 2-(2-chloro-4-(6-methylpyridin-2-yl)phenyl)acetate (intermediate 27) (0.95 eq), 4-amino-2-(methylthio)pyrimidine-5-carbaldehyde (1 eq), and potassium carbonate (3 eq) in DMF was stirred for 12 h at 90 °C. Upon completion the mixture was cooled to room temperature and diluted with ice water. The residue was filtered and washed by DCM to yield the intermediate 28 as a pale-yellow solid without further purification yield 61%.

*6-(2-chloro-4-(6-methylpyridin-2-yl)phenyl)-2-(methylthio)pyrido[2,3-d]pyrimidin-7(8H)-one (**28**)* ^{1}H-NMR (600 MHz, DMSO-*d6*), δ(ppm): 12.66 (1H, s), 8.91 (1H, d, *J* = 1.4 Hz), 8.23 (1H, d, *J* = 1.7

Hz), 8.10 (1H, dd, *J* = 8.0, 1.8 Hz), 8.00 (1H, d, *J* = 1.4 Hz), 7.87 (1H, d, *J* = 7.8 Hz), 7.81 (1H, t, *J* = 7.7 Hz), 7.53 (1H, d, *J* = 8.0 Hz), 7.28 (1H, d, *J* = 7.6 Hz), 2.59 (3H, s), 2.56 (3H, s). ^{13}C NMR (150 MHz, DMSO-*d6*), δ(ppm): 172.2, 161.5, 158.1, 157.0, 154.3, 153.3, 140.4, 137.7, 136.6, 135.1, 133.5, 132.3, 131.4, 126.9, 124.9, 122.7, 117.7, 108.8, 24.4, 13.7.

Synthesis of intermediate 30. To a solution of 6-(2-chloro-4-(6-methylpyridin-2-yl)phenyl)-2-(methylthio)pyrido[2,3-d]pyrimidin-7(8H)-one (intermediate 28) (1 eq) in DMF, 3-chloroperoxybenzoic acid (2 eq) was added at room temperature. Until the reaction was completed detected by TLC, cyclopropanamine (10 eq) was added and the resulting mixture was stirred for additional 12 h at 90 ℃ continually. Upon completion the mixture was cooled to room temperature and diluted with ice water. The residue was filtered and washed by DCM to yield the intermediate 30 as a white solid without further purification yield 87%.

*6-(2-chloro-4-(6-methylpyridin-2-yl)phenyl)-2-(cyclopropylamino)pyrido[2,3-d]pyrimidin-7(8H)-one (**30**)* ^{1}H-NMR (600 MHz, DMSO-*d6*), δ(ppm): 12.06 (1H, d, *J* = 51.1 Hz), 8.65 (1H, d, *J* = 43.8 Hz), 8.22 (1H, d, *J* = 16.1 Hz), 8.07 (1H, d, *J* = 8.0 Hz), 8.03 - 7.90 (1H, m), 7.86 (1H, d, *J* = 8.0 Hz), 7.81 (2H, d, *J* = 10.4 Hz), 7.49 (1H, d, *J* = 8.0 Hz), 7.28 (1H, d, *J* = 7.6 Hz), 2.87 (1H, d, *J* = 14.4 Hz), 2.56 (3H, s), 0.78 – 0.64 (2H, m), 0.56 (2H, s); ^{13}C-NMR (150 MHz, DMSO-*d6*), δ(ppm): 158.5, 158.5, 158.5, 158.5, 153.9, 140.4, 138.2, 137.8, 136.6, 136.3, 134.2, 132.8, 127.3, 127.3, 125.3, 125.3, 123.1, 118.1, 24.8, 24.6, 6.8, 6.8.

Synthesis of intermediate 31a-c. To a mixture of 6-(2-chloro-4-(6-methylpyridin-2-yl)phenyl)-2-(cyclopropylamino)pyrido[2,3-d]pyrimidin-7(8H)-one (intermediate 30) (1 eq), potassium carbonate (3 eq), sodium iodide (0.1 eq), and ethyl 4- or 5- or 6-bromobutyrate (1.5 eq) in DMF was stirred for 12 h at 120 ℃. After completion, the mixture was diluted with 20 ml water and extracted with ethyl acetate (3 × 20 ml). The combined organic layers were washed with saturated aqueous sodium bicarbonate and brine, and then dried over anhydrous sodium sulfate. After removing the solvent under reduced pressure, the crude product was purified by flash chromatography on silica gel, eluting with petroleum ether/ethyl acetate (3 : 1) as a pale-yellow solid, yield 61-83%.

*Ethyl 4-(6-(2-chloro-4-(6-methylpyridin-2-yl)phenyl)-2-(cyclopropylamino)-7-oxopyrido[2,3-d]pyrimidin-8(7H)-yl)butanoate(**31a**)* ^{1}H-NMR (600 MHz, DMSO-*d6*), δ(ppm): 8.63 (1H, d, *J* = 8.0 Hz), 8.22 (1H, dd, *J* = 3.4, 1.8 Hz), 8.07 (1H, ddt, *J* = 12.2, 8.0, 1.9 Hz), 7.88 - 7.76 (3H, m), 7.52 - 7.47 (1H, m), 7.26 (1H, dd, *J* = 13.4, 7.6 Hz), 4.39 (1H, s), 4.02 - 3.92 (2H, m), 3.37 (2H, s), 2.85 (1H, s), 2.56

(3H, d, *J* = 6.3 Hz), 2.39 (2H, d, *J* = 6.8 Hz), 2.01 (2H, d, *J* = 7.2 Hz), 1.12 (3H, td, *J* = 7.1, 3.0 Hz), 0.75 (2H, s), 0.59 – 0.50 (2H, m); ^{13}C-NMR (150 MHz, DMSO-*d6*), δ(ppm): 172.4, 162.6, 161.0, 159.2, 158.0, 158.0, 155.0, 153.4, 139.9, 137.6, 136.4, 136.1, 133.7, 132.3, 126.8, 124.8, 122.6, 117.5, 104.5, 59.8, 31.4, 24.3, 24.3, 23.9, 22.8, 14.0, 6.2, 6.2.

Ethyl 5-(6-(2-chloro-4-(6-methylpyridin-2-yl)phenyl)-2-(cyclopropylamino)-7-oxopyrido[2,3-d]pyrimidin-8(7H)-yl)pentanoate ***(31b)*** ^{1}H-NMR (600 MHz, DMSO-*d6*), δ(ppm): 8.64 (1H, s), 8.21 (1H, d, *J* = 1.8 Hz), 8.08 (1H, dd, *J* = 8.0, 1.8 Hz), 7.86 (1H, d, *J* = 7.8 Hz), 7.84 - 7.77 (2H, m), 7.50 (1H, d, *J* = 7.9 Hz), 7.27 (1H, d, *J* = 7.5 Hz), 4.36 (1H, s), 4.02 (2H, q, *J* = 7.1 Hz), 3.33 (2H, d, *J* = 9.4 Hz), 2.82 (1H, s), 2.56 (3H, s), 2.36 (2H, t, *J* = 7.4 Hz), 1.75 (2H, s), 1.60 (2H, s), 1.14 (3H, t, *J* = 7.1 Hz), 0.75 (2H, d, *J* = 7.3 Hz), 0.59 - 0.52 (2H, m); ^{13}C-NMR (150 MHz, DMSO-*d6*), δ(ppm): 173.2, 163.1, 161.4, 159.6, 158.5, 158.5, 155.4, 153.8, 140.4, 138.2, 136.7, 136.6, 134.2, 132.7, 127.3, 125.3, 123.1, 118.1, 104.9, 60.2, 55.4, 33.7, 27.2, 24.8, 24.4, 22.4, 14.6, 6.7, 6.7.

Ethyl 6-(6-(2-chloro-4-(6-methylpyridin-2-yl)phenyl)-2-(cyclopropylamino)-7-oxopyrido[2,3-d]pyrimidin-8(7H)-yl)hexanoate ***(31c)*** ^{1}H-NMR (600 MHz, $CDCl_3$), δ(ppm): 8.50 (1H, d, *J* = 1.5 Hz), 8.12 (1H, d, *J* = 1.8 Hz), 7.90 (1H, dd, *J* = 7.9, 1.8 Hz), 7.65 (1H, t, *J* = 7.7 Hz), 7.56 (1H, s), 7.52 (1H, d, *J* = 7.8 Hz), 7.46 (1H, d, *J* = 8.0 Hz), 7.12 (1H, d, *J* = 7.6 Hz), 4.44 (1H, s), 4.14 - 4.07 (2H, m), 2.87 (1H, d, *J* = 8.1 Hz), 2.63 (3H, s), 2.31 (3H, q, *J* = 7.1 Hz), 1.81 (2H, s), 1.72 (4H, t, *J* = 7.4 Hz), 1.46 (2H, p, *J* = 7.8 Hz), 1.24 (2H, t, *J* = 6.8 Hz), 0.91 - 0.86 (2H, m), 0.66 - 0.61 (2H, m); ^{13}C-NMR (150 MHz, $CDCl_3$), δ(ppm): 214.7, 175.1, 162.8, 162.1, 158.72, 158.72, 155.61, 155.28, 141.10, 137.18, 135.81, 135.61, 134.45, 132.04, 128.3, 127.1, 125.2, 122.4, 117.8, 60.4, 41.2, 34.4, 29.8, 27.3, 26.7, 24.8, 24.3, 14.4, 7.4, 7.4.

Methyl 4-((6-(2-chloro-4-(6-methylpyridin-2-yl)phenyl)-2-(cyclopropylamino)-7-oxopyrido[2,3-d]pyrimidin-8(7H)-yl)methyl)benzoate ***(31d)*** ^{1}H-NMR (600 MHz, DMSO-*d6*), δ(ppm): 8.67 (1H, d, *J* = 6.9 Hz), 8.23 (1H, s), 8.12 - 8.07 (1H, m), 7.94 - 7.90 (2H, m), 7.90 (1H, s), 7.87 (1H, d, *J* = 7.8 Hz), 7.80 (1H, t, *J* = 7.7 Hz), 7.55 (3H, dd, *J* = 25.5, 8.0 Hz), 7.28 (1H, d, *J* = 7.6 Hz), 5.61 (1H, s), 3.82 (3H, s), 3.34 (2H, s), 2.79 (1H, d, *J* = 8.1 Hz), 2.57 (3H, s), 0.72 (2H, dd, *J* = 7.1, 2.2 Hz), 0.50 (2H, s); ^{13}C-NMR (150 MHz, DMSO-*d6*), δ(ppm): 166.0, 162.6, 161.1, 159.3, 158.0, 158.0, 154.9, 153.4, 147.1, 143.1, 140.0, 137.7, 136.8, 136.0, 133.8, 132.3, 129.2, 129.2, 128.4, 128.4, 126.8, 124.8, 122.7, 117.6, 104.5, 52.1, 24.3, 24.0, 13.9, 6.3, 6.3.

Methyl 4-(2-(6-(2-chloro-4-(6-methylpyridin-2-yl)phenyl)-2-(cyclopropylamino)-7-oxopyrido[2,3-

d]pyrimidin-8(7H)-yl)ethyl)benzoate ***(31e)*** ^{1}H-NMR (600 MHz, DMSO-*d6*), δ(ppm): 8.64 (1H, s), 8.24 - 8.20 (1H, m), 8.10 - 8.04 (1H, m), 7.88 (1H, s), 7.87 (2H, d, *J* = 3.6 Hz), 7.84 (1H, d, *J* = 0.9 Hz), 7.81 (1H, t, *J* = 7.7 Hz), 7.48 (1H, d, *J* = 8.0 Hz), 7.44 - 7.38 (2H, m), 7.28 (1H, d, *J* = 7.6 Hz), 4.59 (1H, d, *J* = 9.0 Hz), 3.83 (3H, s), 3.33 (2H, s), 3.11 (2H, s), 2.87 (1H, d, *J* = 16.6 Hz), 2.57 (3H, s), 0.77 (2H, t, *J* = 11.7 Hz), 0.62 - 0.55 (2H, m); ^{13}C-NMR (150 MHz, DMSO-*d6*), δ(ppm): 166.2, 162.6, 160.8, 159.2, 158.0, 158.0, 154.9, 153.4, 144.5, 139.9, 137.7, 136.4, 136.1, 133.7, 132.3, 129.3, 129.2, 127.7, 127.7, 127.7, 126.8, 124.8, 122.7, 117.6, 104.4, 52.1, 33.1, 29.8, 24.4, 24.0, 6.4, 6.4.

Synthesis of intermediate 32a-c. NaOH (40 eq) was added to a solution of hydroxyamine hydrochloride (10 eq) in methanol (10 mL) cooled by an ice bath. The mixture was stirred for another 30 min. The resulting precipitate was filtered off, and the solution of free hydroxylamine was prepared. The above freshly prepared hydroxyamine solution was placed in a round bottom flask cooled by an ice bath. 31a-e (1 eq) was added to the solution and stirred for 1 h. Upon completion the mixture was concentrated in vacuum, water and 5% HCl were added to adjust pH to 6-8. The precipitate was collected by filtration, washed with water as white solids, yield 20-67%.

4-(6-(2-chloro-4-(6-methylpyridin-2-yl)phenyl)-2-(cyclopropylamino)-7-oxopyrido[2,3-d]pyrimidin-8(7H)-yl)-N-hydroxybutanamide ***(32a)*** ^{1}H-NMR (600 MHz, DMSO-*d6*), δ(ppm): 10.39 (1H, d, *J* = 5.5 Hz), 8.70 (1H, d, *J* = 6.6 Hz), 8.64 (1H, s), 8.22 (1H, d, *J* = 1.8 Hz), 8.08 (1H, ddt, *J* = 8.2, 4.3, 1.8 Hz), 7.89 - 7.75 (3H, m), 7.54 - 7.47 (1H, m), 7.27 (1H, t, *J* = 6.9 Hz), 4.35 (1H, d, *J* = 7.5 Hz), 3.37 (2H, s), 2.85 (1H, s), 2.56 (3H, d, *J* = 3.2 Hz), 2.06 (2H, d, *J* = 8.1 Hz), 1.95 (2H, s), 0.89 - 0.71 (2H, m), 0.55 (2H, s); ^{13}C-NMR (150 MHz, DMSO-*d6*), δ(ppm): 168.5, 162.7, 160.9, 159.2, 158.0, 158.0, 154.9, 153.4, 139.9, 137.7, 136.4, 133.8, 132.3, 126.8, 124.8, 122.6, 117.6, 114.6, 104.5, 54.9, 30.3, 24.4, 24.0, 23.8, 6.3, 6.3. HR-ESI-MS m/z 505.1749 $[M + H]^{+}$ (calcd for $C_{26}H_{26}ClN_6O_3$, 505.1749).

*5-(6-(2-chloro-4-(6-methylpyridin-2-yl)phenyl)-2-(cyclopropylamino)-7-oxopyrido[2,3-d]pyrimidin-8(7H)-yl)-N-hydroxypentanamide (***32b***)* ^{1}H-NMR (600 MHz, DMSO-*d6*), δ(ppm): 10.34 (1H, s), 8.65 (2H, d, *J* = 15.3 Hz), 8.22 (1H, d, *J* = 1.7 Hz), 8.07 (1H, dt, *J* = 8.0, 2.1 Hz), 7.86 (1H, d, *J* = 7.2 Hz), 7.83 (1H, d, *J* = 4.7 Hz), 7.80 (1H, t, *J* = 7.7 Hz), 7.50 (1H, dd, *J* = 8.0, 2.4 Hz), 7.27 (1H, t, *J* = 6.5 Hz), 4.34 (1H, t, *J* = 7.3 Hz), 3.36 (2H, s), 2.90 - 2.78 (1H, m), 2.56 (3H, d, *J* = 2.4 Hz), 2.01 (2H, t, *J* = 7.2 Hz), 1.70 (2H, q, *J* = 8.2 Hz), 1.61 - 1.54 (2H, m), 0.77 (2H, d, *J* = 6.8 Hz), 0.57 (2H, p, *J* = 4.5 Hz); ^{13}C-NMR (150 MHz, DMSO-*d6*), δ(ppm): 169.4, 163.1, 161.3, 159.6, 158.5, 158.5, 155.4, 153.8, 140.4, 138.2, 136.7, 136.6, 134.2, 132.7, 127.3, 125.3, 123.1, 118.1, 104.9, 55.4, 32.6, 27.5, 24.8, 24.4,

23.4, 6.8, 6.8. HR-ESI-MS m/z 519.1903 [M + H] $^{+}$ (calcd for $C_{27}H_{28}ClN_6O_3$, 519.1906).

6-(6-(2-chloro-4-(6-methylpyridin-2-yl)phenyl)-2-(cyclopropylamino)-7-oxopyrido[2,3-d]pyrimidin-8(7H)-yl)-N-hydroxyhexanamide ***(ZMF-25)*** ^{1}H-NMR (600 MHz, DMSO-*d6*), δ(ppm): 10.33 (1H, s), 8.66 (1H, s), 8.21 (1H, d, *J* = 1.8 Hz), 8.07 (1H, dd, *J* = 8.0, 1.8 Hz), 7.95 (1H, s), 7.86 (1H, d, *J* = 7.8 Hz), 7.82 (1H, s), 7.80 (1H, t, *J* = 7.7 Hz), 7.50 (1H, d, *J* = 8.0 Hz), 7.27 (1H, d, *J* = 7.5 Hz), 4.33 (1H, s), 3.35 (2H, s), 2.81 (1H, s), 2.56 (3H, s), 1.95 (2H, t, *J* = 7.4 Hz), 1.67 (2H, d, *J* = 36.9 Hz), 1.56 (2H, p, *J* = 7.5 Hz), 1.40 - 1.29 (2H, m), 0.81 - 0.70 (2H, m), 0.56 (2H, p, *J* = 4.5 Hz); ^{13}C-NMR (150 MHz, DMSO-*d6*), δ(ppm): 169.1, 162.7, 160.9, 159.2, 158.0, 158.0, 154.9, 153.4, 139.9, 137.7, 136.3, 136.2, 133.8, 132.3, 126.8, 124.8, 122.7, 117.6, 104.5, 40.3, 32.2, 27.0, 26.2, 24.9, 24.3, 24.0, 6.3, 6.3. HR-ESI-MS m/z 533.2054 [M + H] $^{+}$ (calcd for $C_{28}H_{30}ClN_6O_3$, 533.2062).

4-((6-(2-chloro-4-(6-methylpyridin-2-yl)phenyl)-2-(cyclopropylamino)-7-oxopyrido[2,3-d]pyrimidin-8(7H)-yl)methyl)-N-hydroxybenzamide ***(32d)*** ^{1}H-NMR (600 MHz, DMSO-*d6*), δ(ppm): 8.78 (1H, d, *J* = 27.6 Hz), 8.67 (1H, s), 8.30 (1H, s), 8.22 (1H, s), 8.09 (1H, d, *J* = 8.0 Hz), 7.91 (1H, s), 7.87 (3H, dd, *J* = 7.9, 4.9 Hz), 7.81 (1H, t, *J* = 7.7 Hz), 7.43 (1H, s), 7.54 (2H, t, *J* = 8.2 Hz), 7.28 (1H, d, *J* = 7.6 Hz), 5.61 (1H, s), 3.39 (2H, s), 2.79 (1H, s), 2.56 (3H, s), 0.75 - 0.69 (2H, m), 0.52 (2H, d, *J* = 23.3 Hz); ^{13}C-NMR (150 MHz, DMSO-*d6*), δ(ppm): 167.6, 163.0, 161.6, 161.6, 158.5, 158.5, 155.4, 153.9, 146.6, 140.5, 138.2, 137.3, 134.2, 132.8, 130.1, 129.8, 129.8, 128.7, 127.3, 127.3, 125.3, 123.2, 123.2, 118.1, 103.2, 55.4, 24.8, 24.5, 6.7, 6.7. HR-ESI-MS m/z 553.1748 [M + H] $^{+}$ (calcd for $C_{30}H_{26}ClN_6O_3$, 553.1749).

4-(2-(6-(2-chloro-4-(6-methylpyridin-2-yl)phenyl)-2-(cyclopropylamino)-7-oxopyrido[2,3-d]pyrimidin-8(7H)-yl)ethyl)-N-hydroxybenzamide ***(32e)*** ^{1}H-NMR (600 MHz, DMSO-*d6*), δ(ppm): 8.76 (1H, s), 8.65 (1H, s), 8.31 (1H, s), 8.23 (1H, d, *J* = 1.8 Hz), 8.08 (1H, dd, *J* = 8.0, 1.8 Hz), 7.87 (1H, d, *J* = 7.8 Hz), 7.85 (1H, s), 7.82 (1H, d, *J* = 7.7 Hz), 7.80 - 7.75 (2H, m), 7.51 (1H, d, *J* = 7.9 Hz), 7.28 (1H, d, *J* = 7.6 Hz), 7.18 (2H, d, *J* = 7.7 Hz), 4.55 (1H, d, *J* = 8.9 Hz), 3.54 - 3.48 (2H, m), 2.98 (2H, d, *J* = 8.9 Hz), 2.89 - 2.86 (1H, m), 2.57 (3H, s), 0.83 - 0.74 (2H, m), 0.63 - 0.58 (2H, m); ^{13}C-NMR (150 MHz, DMSO-*d6*), δ(ppm): 169.4, 162.7, 160.9, 159.3, 158.1, 158.1, 154.9, 153.5, 139.9, 139.1, 137.8, 136.4, 136.2, 133.8, 132.4, 129.3, 129.3, 127.4, 127.4, 127.4, 126.9, 124.8, 122.7, 117.7, 104.5, 79.2, 33.1, 24.4, 24.1, 6.4, 6.4. HR-ESI-MS m/z 567.1981 [M + H] $^{+}$ (calcd for $C_{31}H_{28}ClN_6O_3$, 567.1906).

Cell culture and regents

MDA-MB-231, BT549, MDA-MB-231, MDA-MB-468 and MCF-10A cells were obtained from the American Type Culture Collection (ATCC, Manassas, VA). MDA-MB-231-LUC-GFP was purchased from Servicebio (Servicebio, Wuhan, China). Antibodies and regents are as follows: MTT (M2128, St. Louis, USA), PAK1 (2602, CST), p-PAK1^{Ser144} (2606, CST), p-PAK1^{Ser199} (2605, CST) HADC10 (24913-1-AP, Proteintech), Ki-67 (9449, CST), SQSTM1/p62 (88588, CST), p-ERK1/2$^{Thr202/Tyr204}$ (4370, CST), LC3 (9421, CST), E-cadherin(14472, CST), Snail (3879, CST), Acetylated-Lysine (9441, CST), p-AKT^{Ser473}(4060, CST), p-mTOR^{Ser2448}(2971, CST), ULK1(8054,CST), ERK1/2 (9102, CST), p-ULK1^{S555}(5869, CST), AKT(9272,CST), mTOR(2983, CST), Beclin1 (3738, CST), GAPDH (97166, CST), β-actin (3700, CST), Acetylcysteine (ST2524, Beyotime), Ac-H3K27(82902-1-RR, Proteintech), PD-L1(GB155736, Servicebio), D-Luciferin potassium (BD126660, Bidepharm), FITC Anti-Mouse CD19 Antibody[1D3](E-AB-F0986C, Elabscience), APC Anti-Mouse CD45 Antibody[30-F11](E-AB-F1136E, Elabscience), PE Anti-Mouse CD3 Antibody[17A2](E-AB-F1013D, Elabscience), N^{8}-AcSpd (BD142885, Bide Pharmatech Ltd.).

Molecular Docking

A comprehensive molecular docking study was performed using Discovery Studio software (version 3.5) to investigate the interactions between ZMF-25 and three targeted proteins: PAK1 (PDB code 5DEY), HDAC10 (PDB code 7U6B), and HDAC6 (PDB code 6THV). The crystallographic information for the proteins was obtained from the RCSB PDB database (https://www.rcsb.org). To define the binding site, a ligand-based approach was utilized, employing a 10 Å radius as the defining criterion. For preparing the protein structures, a standardized procedure was followed, which included removing solvent molecules, adding hydrogen atoms, and applying the CHARMM force field. Following this, energy minimization of the ligands was performed while keeping all other settings at their default values.

Molecular Dynamics (MD) Simulations

Molecular dynamics (MD) simulations were performed using the Amber 10 software suite. In the initial phase, energy minimization was performed using the steepest descent method, applying a restraint

of 0.1 kcal/mol•Å^2 to all atoms in the complex for 5000 iterations. Following this, the ligands underwent a second round of energy minimization after their restraints were removed, culminating in a final minimization step conducted without any constraints. To accurately account for long-range electrostatic interactions, the particle-mesh Ewald (PME) summation method was employed. Additionally, the SHAKE algorithm was utilized to maintain covalent bonds involving hydrogen atoms during both equilibration and production runs, allowing for an integration time step of 2 fs. The system temperature was gradually increased from 0 to 310 K over 50 picoseconds (ps) using an annealing protocol. Subsequently, the complex system was equilibrated for an additional 500 ps, during which all previously applied constraints were gradually released. Finally, production simulations were carried out for a total duration of 200 nanoseconds (ns), with no constraints imposed.

Binding-free Energy Calculation (MM-GBSA)

The binding free energy was calculated using the AMBER10 program with the MM-GBSA method. Initially, numerous snapshots were extracted from the MD trajectory of the protein-ligand complex and stripped of water molecules and counter ions. Snapshots were then selected at 10-picosecond intervals during the equilibrium phase of the MD simulation. Each snapshot was analyzed individually to evaluate the free energy of the complex, protein, and ligand components. The binding free energy was subsequently calculated as the difference between these energy values. To determine the free energy (G) of each molecular species using the MM-GBSA method, a specific protocol was applied.

$$G = E_{gas} + G_{sol} - TS$$

$$E_{gas} = E_{\mathrm{int}} + E_{ele} + E_{vdw}$$

$$E_{\mathrm{int}} = E_{bond} + E_{angle} + E_{torsion}$$

$$G_{sol} = G_{GB} + G_{nonpolar}$$

$$G_{nopolar} = \gamma SAS$$

The Anzymatic Assay of HDACs

Dose-response experiments, including internal controls, were performed to evaluate the inhibition of recombinant human HDACs using black low-binding Nunc 96-well microtiter plates. Serial dilutions

were prepared from 10 mM DMSO stock solutions in HDAC assay buffer (comprising 25 mM Tris, pH 8.0, 1 mM $MgCl_2$, 0.1 mg/mL BSA, 137 mM NaCl, and 2.7 mM KCl). A volume of 10 μL of the appropriate inhibitor dilution was added to each well, followed by 25 μL of HDAC analysis buffer containing either Ac-Leu-Gly-Lys(Ac)-AMC (substrate for HDAC1-3 and HDAC6) or Ac-Arg-His-Lys(Ac)-Lys(Ac)-AMC (substrate for HDAC10). Subsequently, 15 μL of the corresponding HDAC solution was added, and the plate was incubated at 37 °C for 30 minutes. After incubation, 50 μL of trypsin (0.4 mg/mL) was added, and the reaction was allowed to proceed for an additional 30 minutes at room temperature. Fluorescence was measured using a Perkin-Elmer Enspire plate reader with excitation at 360 nm and emission detection at 460 nm. Each experiment was conducted in duplicate, and the data were analyzed via nonlinear regression in GraphPad Prism to determine IC50 values from the dose-response curves.

The Enzymatic Assay of PAKs

The enzymatic assays for PAKs were conducted following a previously published protocol. The reactions took place at a temperature of 30°C for 40 minutes. A 50 μL reaction mixture was prepared, consisting of 40 mM Tris (pH 7.4), 10 mM $MgCl_2$, 0.1 mg/mL BSA, 1.0 mM DTT, 50 μM ATP, 0.2 μg/mL PAKs, and 100 μM lipid substrate. To ensure compatibility, the compounds were diluted in a solution containing 10% DMSO, and only 5 μL of this diluted solution was added to each 50 μL reaction volume, resulting in a final concentration of 1% DMSO for all reactions. For analysis purposes, we employed the Kinase-Glo Plus luminescence kinase assay kit and ADP-Glo Plus luminescence kinase assay kit. The emitted luminescent signal from these assays is directly proportional to the ATP quantity present and inversely proportional to the level of kinase activity demonstrated. We utilized Prism GraphPad software with nonlinear regression using normalized dose–response fit to determine IC_{50} values.

KinomeScan

The kinase engagement assay, conducted by Eurofins Discovery, assessed the binding affinity of **ZMF-25** for a panel of protein kinases at a concentration of 1 μM.

SPR Assay

Surface plasmon resonance (SPR) assays were conducted using a Biacore T200 instrument. Recombinant PAK1 and HDAC10 proteins were immobilized on a CM5 sensor chip at a flow rate of 10 μL/min for 7 minutes, achieving response units (RU) within the range of 12,000 to 19,000. Following completion of the coupling process, five concentrations (0.78μM, 1.5625 μM, 3.125 μM, 6.25 μM, 12.5 μM and 25 μM, 50μM) of compound ZMF-25 were diluted in PBS (with DMSO concentration below <0.01%) and passed through the sensor chip at a flow rate of 30 μL/min for a duration of 120 seconds before dissociation occurred over a period of 300 seconds. To regenerate the sensor chip, PBS containing NaCl (2.5 mol/L) was flowed through it for only thirty seconds at a flow rate of 30 μL/min. The KD values were determined using analysis software provided by Biacore platform.

CETSA Assay

To investigate changes in protein thermal stability subsequent to treating MDA-MS-231 cells with either DMSO or 10 μM ZMF-25 for 6 hours, the cellular thermal shift assay (CETSA) was utilized. After this treatment period, the cells were collected and heated at various temperatures ranging from 37°C to 67°C, in increments of 5°C, for three minutes each. The heated samples underwent three cycles of freezing and thawing using liquid nitrogen, followed by centrifugation at 12,000 rpm at -4°C for twenty minutes. Subsequently, protein quantification was conducted on the harvested cells using the BCA assay, with the samples being boiled at 95°C for five minutes prior to western blot analysis.

Cell Migration Assay

The migratory capacity of TNBC cells was evaluated through the implementation of both wound healing and transwell assays. In the wound healing assay, MDA-MB-231 cells were cultivated in 6-well plates until they achieved complete confluence. A scratch was introduced across the center of each well using a 1 ml pipette tip, followed by careful rinsing with PBS to eliminate non-attached cells. Subsequently, the cells were exposed to ZMF-25 for durations of either 24 or 48 hours, with images documented using an inverted microscope. For the transwell assay, MDA-MB-231 cells were seeded at

a density of 15,000 cells per well into the upper chambers of a 24-well plate fitted with transwell inserts. Various concentrations of ZMF-25 were introduced and allowed to incubate for 24 hours. Post-incubation, the cells in the upper chamber were removed, while those in the lower chamber were fixed with 4% paraformaldehyde, stained with crystal violet, and visualized under an inverted microscope.

Plate Cloning Experiment

The migratory capacity of TNBC cells was assessed using both wound healing and transwell assays. For the wound healing assay, MDA-MB-231 cells were cultured in 6-well plates until they reached full confluence. A linear scratch was created across the center of each well using a 1 ml pipette tip, followed by gentle rinsing with PBS to remove non-adherent cells. The cells were then treated with ZMF-25 for either 24 or 48 hours, and images were captured using an inverted microscope. In the transwell assay, MDA-MB-231 cells were seeded at a density of 15,000 cells per well into the upper chambers of a 24-well plate equipped with transwell inserts. Different concentrations of ZMF-25 were added, and the cells were incubated for 24 hours. After incubation, cells remaining in the upper chamber were removed, while those that had migrated to the lower chamber were fixed with 4% paraformaldehyde, stained with crystal violet, and examined under an inverted microscope.

Cell Viability Assay

Cell viability was determined by MTT assay where MDA-MB-231 and MCF10A cells were cultured in 96-well plates and allowed to proliferate for 24 hours. Then MDA-MB-231 cells were treated with different concentrations of ZMF-25 and NAC (5μM) for 24 to 48 hours and MCF10A cells were treated with different concentrations of ZMF-25 for 24 to 48 hours. After adding MTT and incubating for another 4 hours, the cells were lysed with DMSO and cell viability was measured at 490 nm.

Flow Cytometry Analysis of 2-NBDG

Cells were plated in 6-well plates and grown until they reached 70-80% confluence. Subsequently, ZMF-25(0, 2.5, 5, 10μM) were introduced separately, and the cultures were maintained for 24 hours. The cells were then stained with the 2-NBDG dye and analyzed by flow cytometry.

Flow Cytometry Analysis of ROS

The production of reactive oxygen species (ROS) in MDA-MB-231 cells was quantified using a ROS assay kit (Invitrogen, Cat. No. 2448096). Cells were plated in 6-well plates and grown until they reached 70-80% confluence. Subsequently, ZMF-25, FRAX486, Tubastatin A, and NAC were introduced separately, and the cultures were maintained for 24 hours. The cells were then stained with the ROS probe and analyzed by flow cytometry.

Flow Cytometry Analysis of Mitochondrial Membrane Potential

The mitochondrial membrane potential in MDA-MB-231 cells was assessed using JC-1 staining. Cells were plated in 6-well plates and grown to 70-80% confluence. Following the addition of ZMF-25, FRAX486, Tubastatin A, and NAC, the cultures were maintained for 24 hours. The cells were then stained with JC-1 and analyzed by flow cytometry.

Immunofluorescence Analysis

After treating cells with ZMF-25 for 24 hours, they were gently rinsed with PBS and fixed with 4% paraformaldehyde. The cells were permeabilized with 0.2% Triton for 3 minutes, washed with PBS, and blocked with 2.5% BSA at room temperature for an hour. Subsequently, the cells were incubated with primary antibodies against E-cadherin (1:200) and Snail (1:200) overnight at 4°C. After washing, a fluorescent secondary antibody was applied for an hour, followed by extensive washing with PBS. Images were captured using a laser confocal microscope.

Plasmid Transfection

MDA-MB-231 cells were plated on glass slides in 24-well plates and transfected with the mRFP-GFP-LC3 plasmid according to the manufacturer's instructions (HANBIO, Product Code: HB-AP210 0001). Twenty-four hours post-transfection, the cells were treated with ZMF-25, FRAX486, and Tubastatin A, and processed for immunofluorescence experiments as previously described.

Animal experiments

Female BALB/c nude mice and female BALB/c mice , aged between 3 and 4 weeks and weighing approximately 18 to 20 grams, were sourced from SPF (Beijing) Biotechnology Co., Ltd. (Certificate No: SCXK[Beijing]20190010). The ethical guidelines followed for animal care and experimentation conformed to the regulations set forth by Shenzhen University's Ethics Committee for Animal Experiments, in accordance with standard laboratory animal care protocols. A total of forty-nine BALB/c nude mice underwent subcutaneous injection with MDA-MB-231 cells to induce xenograft tumors. When the tumor volume attained 80 mm^3, the mice were randomized into seven groups: the Control group (n=7), the ZMF-25 treatment groups at dosages of 5 mg/kg (n=7, administered daily via intraperitoneal injection), 10 mg/kg (n=7, intraperitoneal injection daily), and 20 mg/kg (n=7, intraperitoneal injection daily), as well as the FRAX486 treatment group (n=7, intraperitoneal injection daily), the Tubastatin A treatment group (n=7, intraperitoneal injection daily), and the combined Tubastatin A + FRAX486 treatment group (n=7, intraperitoneal injection daily). Tumor size and body weight were monitored daily throughout the experiment. The medication was dissolved in a solvent consisting of 10% DMSO, 30% PEG300, and 60% physiological saline. Meanwhile female BALB/c nude mice were established as an experimental metastasis model by intravenous injection of MDA-MB-231-Luc cells (1×10^6 cells/100 μL). After modeling, the rats were divided into vehicle and ZMF-25 groups and treated with ZMF-25 (20 mg/kg). In vivo imaging was performed after intraperitoneal injection of 100 mg/kg luciferase substrate on days 0, 1, 3, 5, and 7 of treatment, respectively, and the bioluminescence intensity was recorded in each group. In addition, in order to investigate the toxicity of ZMF-25, Female BALB/c mice were given ZMF-25 (20 mg/kg/d) for 14 consecutive days, and at the end of the administration, blood was taken for routine blood tests, and organs were taken for HE staining, and blood, spleen and thymus were taken for flow-through assay of CD45, CD3, CD19.

Western Blot

MDA-MB-231 cells were harvested post-ZMF-25 treatment and lysed on ice for 45 minutes following the addition of lysate. The lysates were then centrifuged at 12,000 rpm for 20 minutes at 4°C to collect the supernatant, which underwent protein quantification using the BCA method. Subsequently, the supernatant was analyzed via SDS-PAGE electrophoresis. The resolved proteins were transferred

onto a PVDF membrane, which was blocked with 5% skimmed milk powder. The membrane was then incubated with the primary antibody overnight at 4℃, followed by a 60-minute incubation with the secondary antibody at room temperature. Finally, ECL reagent was used to visualize the target bands, which were photographed and the results were statistically analyzed.

HE Staining

Tumor tissues fixed in 4% paraformaldehyde were processed for paraffin embedding and sectioning. The sections were dewaxed using xylene and anhydrous ethanol, stained with hematoxylin and eosin, dehydrated, and mounted. Histopathological changes were then observed under a microscope.

Immunohistochemistry Analysis (IHC)

Paraffin-embedded sections of 4% paraformaldehyde-fixed tumor tissues were prepared. Antigen retrieval was achieved through microwave irradiation in citrate buffer (pH 6.0). The sections were then incubated with primary and secondary antibodies, followed by application of an HRP-conjugated secondary antibody for 30 minutes. Diaminobenzidine solution was used for color development. After hematoxylin counterstaining, the sections were mounted and examined under a microscope to assess tissue status.

Oxygen Consumption Rate

1)Seed cells: 100 μL 10^5/mL of single cell suspension of each group was taken at 37℃ and supplemented with 150 mL of medium, incubated for 24 hours, treated with ZMF-25 (10 μM) for 24 hours, and then centrifuged at 300 rpm and assayed on the machine.

2)Test change: aspirate off 175μL of the original growth medium, rinse twice utilizing 600 μL of seahorse-specific test medium, and finally add 450 μL to 525 μL to observe the continuity of cells within each well under a microscope, after which it was placed in a non-CO_2 incubator for 1h.

3)In the four dosing tanks in each well, 75 μL of various respiratory chain inhibitors were dosed according to the experimental design, and various respiratory chain inhibitors were preserved and used at the following concentrations: oligomycin was preserved at a concentration of 2.5 mmol/L, and used at a concentration of 1 μmol/L; FCCP was preserved at a concentration of 2.5 mmol/L, and used at a concentration of 1 μmol/L; and antimycin A was preserved at a concentration of 2.5 mmol/L, and used

at a concentration of 1 μmol/L.

4)Instrument method editing and calibration plate calibration;

5)Calibration plate replacement with cell plate run.

Extracellular Acidification Rate

1) Seed cells: 100 μL 10^5/mL of single cell suspension of each group was taken at 37°C and supplemented with 150 mL of medium, incubated for 24 hours, treated with ZMF-25 (10 μM) for 24 hours, and then centrifuged at 300 rpm and assayed on the machine.

2) Test change: aspirate off 175 μL of original growth medium, rinse twice utilizing 600ul of hippocampus-specific test medium, and finally add 450μL to 525μL to observe the continuity of cells within each well under the microscope, after which it was placed in a non-CO_2 incubator for 1 hour.

3) Concentration of drug use: Glucose 3,000 μL(100 mM), Oligomycin720 μL(100 μM), 2-DG 3,000 μL(500 mM).

4) Instrument method editing and calibration plate calibration;

5) Calibration plate replacement with cell plate run.

N^8-acetylspermidine(N8-AcSpd) content detection

Preparation of cell samples

The 231 cells were inoculated in 15 cm of medium, and after overnight walling of the cells, ZMF-25 (0, 2.5, 5, 10μM) was added and the cells were cultured for 24 h. The cells were then washed with 0.9% NaCl (saline) and digested with trypsin. Then the medium was removed, cells were washed with 0.9% NaCl (saline), trypsin digested, resuspended in complete medium at 4°C, centrifuged at 300 g at 4°C for 5 min, washed twice in complete medium at 4°C, and resuspended in fresh 0.9% NaCl (saline). Then, the cells were counted, 10×10^6 cells per portion, centrifuged at 1000 g, 4°C for 5 min, the supernatant was discarded, and the cells were rapidly frozen in liquid nitrogen and then stored at -80°C.

Extraction and quantification of acetylated spermine

Cell samples were extracted with 300 μL MeOH/MeCN (50:50) on ice, vortexed and sonicated for five minutes, centrifuged using 120,000 r/min at 4°C for 5 minutes, the resulting supernatant was aspirated, and the remaining cells were added to 300 μL MeOH/MeCN (50:50) vortexed and centrifuged

at 120,000 r/min at 4°C for 5 minutes, combined the supernatant was vortexed and filtered through a 0.22 μl membrane into a liquid phase vial and set aside. LC-MS/MS analysis was performed on a Agilent G6475A triple Quadrupole Mass Spectrometer (Agilent, USA) with electrospray ionization (ESI) source using the following settings: curtain gas: 30 psi; collision gas: low; ion spray: 3900 V; source temperature: 310°C; ion source gas 1:40 (GS1) and ion source gas 2:50 (GS2). Acetylated spermidines were separated by chromatography on an Acquity HSS T3 column (150 mm x2.1 mm, 1.7 um, Waters) kept at 20°C and a flow rate of 0.3 mL/min. Eluent A: water + 0.1% formic acid, eluent ， B: MeCN + 0.1% formic acid. Gradient: 0 to 1min 0%-20%B; 1 to 2min 20%B, 3-3.2nin 20%-100%B, 3.2-4min, 100%B,4-4.2 100% -0%B,4.2-5min 100%.

Identification and quantification of acetylation isomers was based on unique transitions extracted from the MS/MS fragmentation patterns of commercially available standards of N^8-AcSpd.

Below are the chromatographic traces of specific transitions for each group of acetylated isomers (from top to bottom ZMF-25 (0 μM), ZMF-25 (2.5 μM), ZMF-25 (5 μM), ZMF-25(10 μM). N-acetylspermidine: 188.2/171.2. N^8-AcSpd was configured as a 1 mg/ml master solution with double distilled water, diluted with double distilled water to the following concentration samples 800 ng/ml, 400 ng/ml, 200 ng/ml, 100 ng/ml, 50 ng/ml, 25 ng/ml, 12.5 ng/ml, 6.25 ng/ml, and 3.125 ng/ml, and injected into the test, which was detected by weighted the linear regression operation was performed by the least squares method to obtain the standard curve, and the concentration of N^8-AcSpd in each sample was calculated by the standard curve.

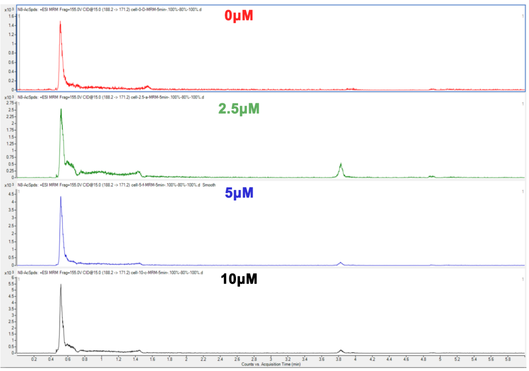

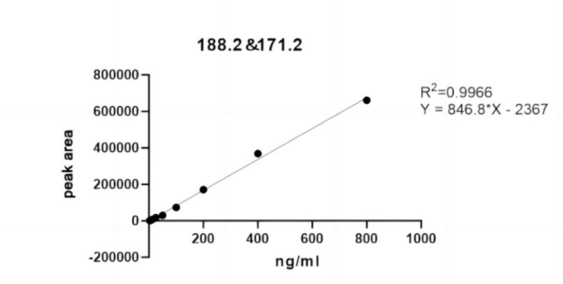

Statistical Analysis

Data were presented as mean ± SEM. Multiple group comparisons were conducted using a one-way analysis of variance (ANOVA) test, with $p<0.05$ indicating a significant distinction.

Supplementary schemes, tables and figures

Figure S1 Structure of PAK1 and HDAC IIb Inhibitors

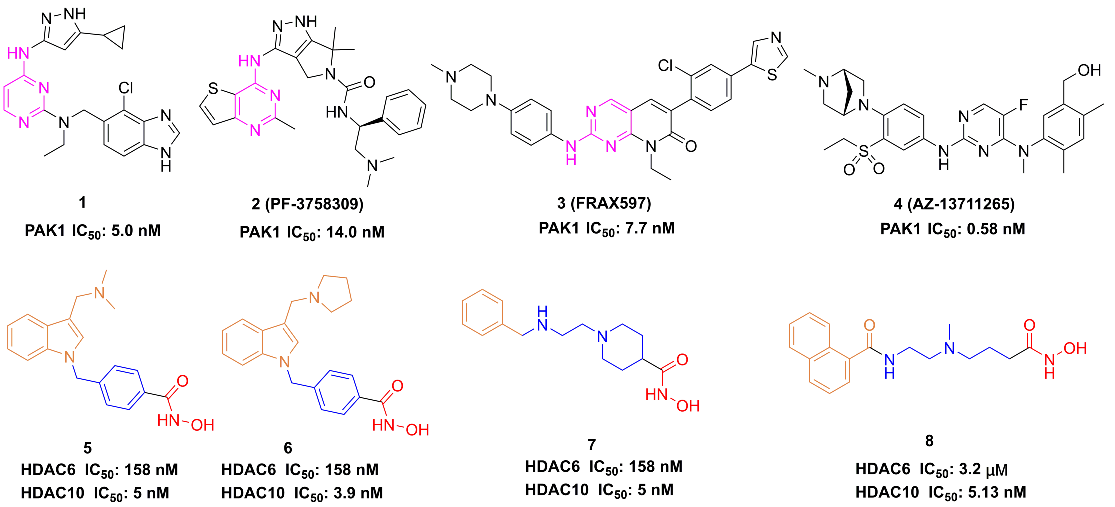

Figure S1 Structure of PAK1 and HDAC IIb Inhibitors.

Figure S2 Effect of DFZK-748 and si-HDAC10 on cell viability of MDA-MB-231 cells.

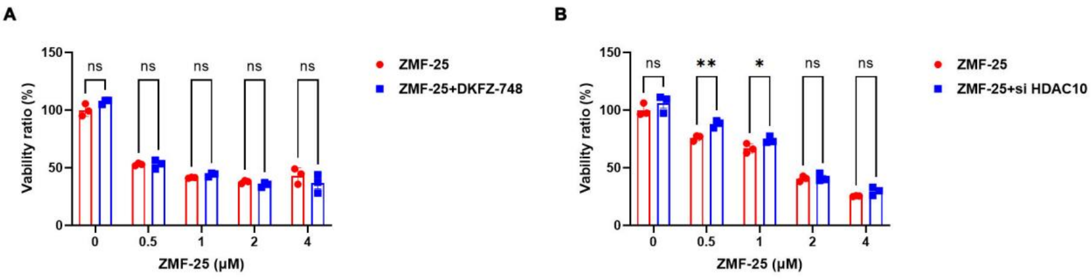

Figure S2 Effect of DFZK-748 and si-HDAC10 on cell viability. (A) MTT assay for the effects of ZMF-25 and DKFZ-748 on MDA-MB-231 cells; (B) MTT assay for the effect of ZMF-25 on cell viability after knockdown of *HDAC10*.

Figure S3 HE staining of the heart, liver, spleen, lung and kidney of the mice in different groups.

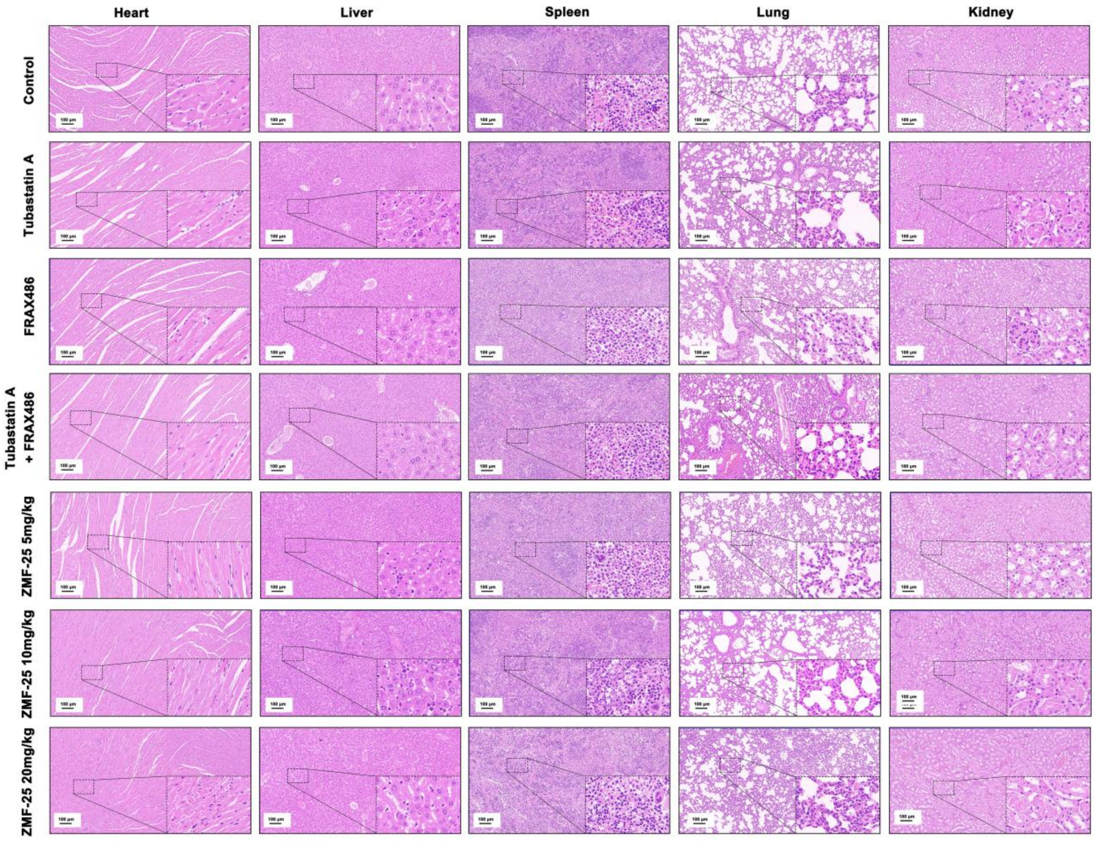

Figure S3 HE staining of the heart, liver, spleen, lung and kidney of the mice in different groups.

Figure S4 Effect of ZMF-25 treatment on mice organs.

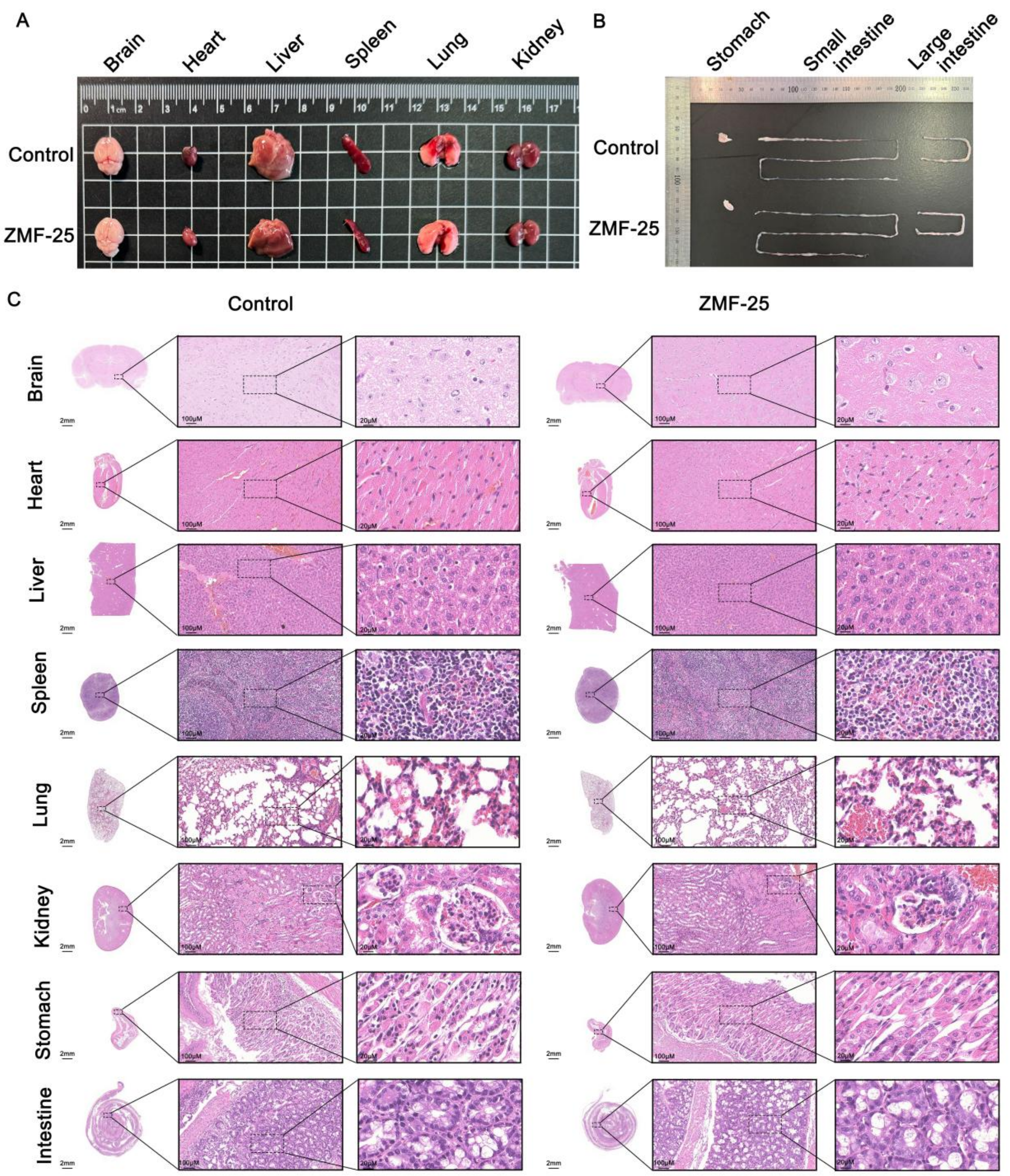

Figure S4 Effect of ZMF-25 treatment on mice organs. (A-B) Representative images of the brain, heart, liver, spleen, lungs, kidneys, stomach, small intestine, and large intestine. (C) Representative HE staining images of the brain, heart, liver, spleen, lung, kidney, stomach, and intestine.

Figure S5 Effect of ZMF-25 on cell viability and the detection of basal autophagy level.

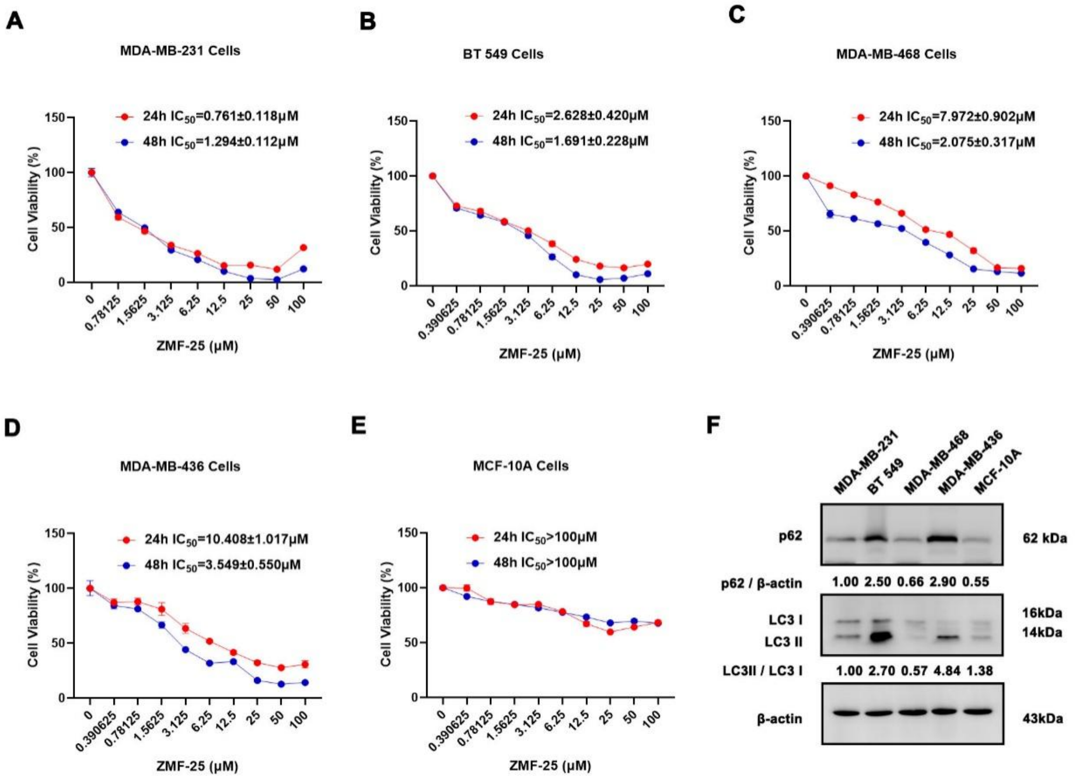

Figure S5 Effect of ZMF-25 on cell viability and the detection of basal autophagy level. (A-E) Effect of ZMF-25 on cell viability of MDA-MB-231 cells, BT-549 cells, MDA-MB-468 cells, MDA-MB-436 cells and MCF-10A cells. (F) Western blotting was performed to detect basal p62 and LC3 expression levels in MDA-MB-231 cells, BT-549 cells, MDA-MB-468 cells, MDA-MB-436 cells and MCF-10A cells.

Table S1 CI values of FRAX486, Tubastatin A and DKFZ-748 for 24h

FRAX486(μM)	Tubastatin A(μM)	DKFZ-748(μM)	Survival rate	CI
50.00	50.00	6.25	0.03	1.28
50.00	25.00	6.25	0.01	0.57
50.00	12.50	6.25	0.01	0.57
50.00	6.25	6.25	0.02	0.78
50.00	3.13	6.25	0.01	0.57
50.00	1.56	6.25	0.01	0.57
50.00	0.78	6.25	0.02	0.78
25.00	50.00	6.25	0.01	0.34

25.00	25.00	6.25	0.03	0.54
25.00	12.50	6.25	0.03	0.54
25.00	6.25	6.25	0.02	0.44
25.00	3.13	6.25	0.02	0.39
25.00	1.56	6.25	0.04	0.73
25.00	0.78	6.25	0.02	0.49
12.50	50.00	6.25	0.07	0.66
12.50	25.00	6.25	0.05	0.50
12.50	12.50	6.25	0.05	0.48
12.50	6.25	6.25	0.04	0.39
12.50	3.13	6.25	0.04	0.36
12.50	1.56	6.25	0.03	0.34
12.50	0.78	6.25	0.06	0.52
6.25	50.00	6.25	0.18	0.78
6.25	25.00	6.25	0.21	0.88
6.25	12.50	6.25	0.27	1.12
6.25	6.25	6.25	0.38	1.61
6.25	3.13	6.25	0.42	1.85
6.25	1.56	6.25	0.42	1.82
6.25	0.78	6.25	0.54	2.70
3.13	50.00	6.25	0.35	0.95
3.13	25.00	6.25	0.40	1.02
3.13	12.50	6.25	0.49	1.28
3.13	6.25	6.25	0.67	2.25
3.13	3.13	6.25	0.66	2.05
3.13	1.56	6.25	0.74	2.74
3.13	0.78	6.25	0.73	2.61
1.56	50.00	6.25	0.43	0.81
1.56	25.00	6.25	0.46	0.74

1.56	12.50	6.25	0.61	1.09
1.56	6.25	6.25	0.74	1.62
1.56	3.13	6.25	0.70	1.27
1.56	1.56	6.25	0.90	5.75
1.56	0.78	6.25	0.94	15.52
0.78	50.00	6.25	0.46	0.70
0.78	25.00	6.25	0.54	0.67
0.78	12.50	6.25	0.61	0.65
0.78	6.25	6.25	0.82	1.72
0.78	3.13	6.25	0.80	1.28
0.78	1.56	6.25	0.92	8.58
0.78	0.78	6.25	0.96	48.09
50.00	50.00	12.50	0.04	1.46
50.00	25.00	12.50	0.02	0.78
50.00	12.50	12.50	0.01	0.68
50.00	6.25	12.50	0.01	0.33
50.00	3.13	12.50	0.01	0.45
50.00	1.56	12.50	0.01	0.57
50.00	0.78	12.50	0.02	0.98
25.00	50.00	12.50	0.03	0.64
25.00	25.00	12.50	0.02	0.39
25.00	12.50	12.50	0.02	0.39
25.00	6.25	12.50	0.01	0.34
25.00	3.13	12.50	0.02	0.39
25.00	1.56	12.50	0.02	0.49
25.00	0.78	12.50	0.02	0.44
12.50	50.00	12.50	0.04	0.42
12.50	25.00	12.50	0.06	0.55
12.50	12.50	12.50	0.05	0.45

12.50	6.25	12.50	0.04	0.39
12.50	3.13	12.50	0.03	0.27
12.50	1.56	12.50	0.03	0.29
12.50	0.78	12.50	0.03	0.29
6.25	50.00	12.50	0.19	0.85
6.25	25.00	12.50	0.18	0.76
6.25	12.50	12.50	0.29	1.23
6.25	6.25	12.50	0.35	1.49
6.25	3.13	12.50	0.37	1.55
6.25	1.56	12.50	0.29	1.18
6.25	0.78	12.50	0.37	1.57
3.13	50.00	12.50	0.29	0.76
3.13	25.00	12.50	0.38	0.94
3.13	12.50	12.50	0.46	1.16
3.13	6.25	12.50	0.62	1.84
3.13	3.13	12.50	0.60	1.68
3.13	1.56	12.50	0.66	2.07
3.13	0.78	12.50	0.68	2.17
1.56	50.00	12.50	0.35	0.60
1.56	25.00	12.50	0.44	0.68
1.56	12.50	12.50	0.51	0.75
1.56	6.25	12.50	0.69	1.37
1.56	3.13	12.50	0.69	1.22
1.56	1.56	12.50	0.78	1.92
1.56	0.78	12.50	0.83	2.65
0.78	50.00	12.50	0.48	0.73
0.78	25.00	12.50	0.47	0.49
0.78	12.50	12.50	0.60	0.65
0.78	6.25	12.50	0.73	0.97

0.78	3.13	12.50	0.73	0.85
0.78	1.56	12.50	0.73	0.79
0.78	0.78	12.50	0.79	1.18
50.00	50.00	25.00	0.05	1.99
50.00	25.00	25.00	0.04	1.46
50.00	12.50	25.00	0.04	1.55
50.00	6.25	25.00	0.01	0.33
50.00	3.13	25.00	0.01	0.45
50.00	1.56	25.00	0.01	0.45
50.00	0.78	25.00	0.01	0.57
25.00	50.00	25.00	0.04	0.78
25.00	25.00	25.00	0.04	0.73
25.00	12.50	25.00	0.03	0.54
25.00	6.25	25.00	0.02	0.39
25.00	3.13	25.00	0.02	0.39
25.00	1.56	25.00	0.02	0.39
25.00	0.78	25.00	0.01	0.34
12.50	50.00	25.00	0.07	0.64
12.50	25.00	25.00	0.08	0.74
12.50	12.50	25.00	0.04	0.41
12.50	6.25	25.00	0.03	0.32
12.50	3.13	25.00	0.03	0.34
12.50	1.56	25.00	0.02	0.25
12.50	0.78	25.00	0.02	0.25
6.25	50.00	25.00	0.20	0.87
6.25	25.00	25.00	0.24	1.03
6.25	12.50	25.00	0.27	1.14
6.25	6.25	25.00	0.41	1.79
6.25	3.13	25.00	0.42	1.83

6.25	1.56	25.00	0.36	1.53
6.25	0.78	25.00	0.33	1.37
3.13	50.00	25.00	0.37	1.02
3.13	25.00	25.00	0.41	1.03
3.13	12.50	25.00	0.51	1.34
3.13	6.25	25.00	0.61	1.80
3.13	3.13	25.00	0.73	2.85
3.13	1.56	25.00	0.72	2.68
3.13	0.78	25.00	0.71	2.55
1.56	50.00	25.00	0.42	0.80
1.56	25.00	25.00	0.47	0.76
1.56	12.50	25.00	0.51	0.74
1.56	6.25	25.00	0.72	1.60
1.56	3.13	25.00	0.73	1.61
1.56	1.56	25.00	0.85	4.26
1.56	0.78	25.00	0.84	3.87
0.78	50.00	25.00	0.44	0.63
0.78	25.00	25.00	0.52	0.62
0.78	12.50	25.00	0.59	0.61
0.78	6.25	25.00	0.75	1.28
0.78	3.13	25.00	0.73	0.96
0.78	1.56	25.00	0.78	1.33
0.78	0.78	25.00	0.82	2.08
50.00	50.00	50.00	0.06	2.17
50.00	25.00	50.00	0.03	1.37
50.00	12.50	50.00	0.02	0.89
50.00	6.25	50.00	0.03	1.18
50.00	3.13	50.00	0.04	1.72
50.00	1.56	50.00	0.02	0.98

50.00	0.78	50.00	0.01	0.68
25.00	50.00	50.00	0.09	1.56
25.00	25.00	50.00	0.03	0.54
25.00	12.50	50.00	0.04	0.82
25.00	6.25	50.00	0.04	0.77
25.00	3.13	50.00	0.04	0.82
25.00	1.56	50.00	0.02	0.39
25.00	0.78	50.00	0.04	0.73
12.50	50.00	50.00	0.14	1.18
12.50	25.00	50.00	0.13	1.10
12.50	12.50	50.00	0.09	0.75
12.50	6.25	50.00	0.06	0.56
12.50	3.13	50.00	0.09	0.75
12.50	1.56	50.00	0.05	0.50
12.50	0.78	50.00	0.06	0.52
6.25	50.00	50.00	0.30	1.38
6.25	25.00	50.00	0.29	1.27
6.25	12.50	50.00	0.41	1.88
6.25	6.25	50.00	0.54	2.72
6.25	3.13	50.00	0.52	2.52
6.25	1.56	50.00	0.43	1.91
6.25	0.78	50.00	0.31	1.25
3.13	50.00	50.00	0.40	1.18
3.13	25.00	50.00	0.43	1.13
3.13	12.50	50.00	0.55	1.55
3.13	6.25	50.00	0.71	2.89
3.13	3.13	50.00	0.67	2.25
3.13	1.56	50.00	0.77	3.97
3.13	0.78	50.00	0.67	2.19

1.56	50.00	50.00	0.41	0.78
1.56	25.00	50.00	0.58	1.21
1.56	12.50	50.00	0.54	0.85
1.56	6.25	50.00	0.82	4.53
1.56	3.13	50.00	0.68	1.35
1.56	1.56	50.00	0.77	2.32
1.56	0.78	50.00	0.77	2.40
0.78	50.00	50.00	0.42	0.58
0.78	25.00	50.00	0.50	0.58
0.78	12.50	50.00	0.60	0.67
0.78	6.25	50.00	0.64	0.69
0.78	3.13	50.00	0.68	0.78
0.78	1.56	50.00	0.79	2.05
0.78	0.78	50.00	0.88	11.74

Table S2 CI values of FRAX486, Tubastatin A and DKFZ-748 for 48h

FRAX486(μM)	Tubastatin A(μM)	DKFZ-748(μM)	Survival rate	CI
50.00	50.00	6.25	0.02	0.98
50.00	25.00	6.25	0.02	0.91
50.00	12.50	6.25	0.02	0.90
50.00	6.25	6.25	0.02	0.83
50.00	3.13	6.25	0.01	0.69
50.00	1.56	6.25	0.01	0.69
50.00	0.78	6.25	0.02	0.90
25.00	50.00	6.25	0.05	1.02
25.00	25.00	6.25	0.02	0.39
25.00	12.50	6.25	0.01	0.35
25.00	6.25	6.25	0.01	0.35
25.00	3.13	6.25	0.02	0.42

25.00	1.56	6.25	0.01	0.23
25.00	0.78	6.25	0.01	0.23
12.50	50.00	6.25	0.02	0.30
12.50	25.00	6.25	0.02	0.25
12.50	12.50	6.25	0.02	0.21
12.50	6.25	6.25	0.01	0.16
12.50	3.13	6.25	0.01	0.14
12.50	1.56	6.25	0.01	0.15
12.50	0.78	6.25	0.01	0.15
6.25	50.00	6.25	0.08	0.49
6.25	25.00	6.25	0.04	0.26
6.25	12.50	6.25	0.09	0.49
6.25	6.25	6.25	0.48	3.38
6.25	3.13	6.25	0.42	2.45
6.25	1.56	6.25	0.40	2.18
6.25	0.78	6.25	0.12	0.55
3.13	50.00	6.25	0.19	1.07
3.13	25.00	6.25	0.29	1.46
3.13	12.50	6.25	0.55	3.65
3.13	6.25	6.25	0.75	7.46
3.13	3.13	6.25	0.80	7.67
3.13	1.56	6.25	0.87	11.97
3.13	0.78	6.25	0.70	3.23
1.56	50.00	6.25	0.30	1.99
1.56	25.00	6.25	0.40	2.12
1.56	12.50	6.25	0.59	3.60
1.56	6.25	6.25	0.75	5.87
1.56	3.13	6.25	0.78	4.65
1.56	1.56	6.25	0.88	10.11

1.56	0.78	6.25	0.78	2.78
0.78	50.00	6.25	0.36	2.73
0.78	25.00	6.25	0.48	3.03
0.78	12.50	6.25	0.70	6.19
0.78	6.25	6.25	0.92	78.31
0.78	3.13	6.25	0.77	3.41
0.78	1.56	6.25	0.84	4.55
0.78	0.78	6.25	0.85	3.89
50.00	50.00	12.50	0.02	0.91
50.00	25.00	12.50	0.03	1.38
50.00	12.50	12.50	0.01	0.62
50.00	6.25	12.50	0.01	0.69
50.00	3.13	12.50	0.02	1.04
50.00	1.56	12.50	0.02	0.83
50.00	0.78	12.50	0.01	0.69
25.00	50.00	12.50	0.02	0.54
25.00	25.00	12.50	0.02	0.46
25.00	12.50	12.50	0.01	0.35
25.00	6.25	12.50	0.02	0.38
25.00	3.13	12.50	0.02	0.45
25.00	1.56	12.50	0.01	0.19
25.00	0.78	12.50	0.02	0.38
12.50	50.00	12.50	0.04	0.43
12.50	25.00	12.50	0.02	0.23
12.50	12.50	12.50	0.02	0.21
12.50	6.25	12.50	0.02	0.24
12.50	3.13	12.50	0.01	0.15
12.50	1.56	12.50	0.01	0.14
12.50	0.78	12.50	0.01	0.17

6.25	50.00	12.50	0.08	0.49
6.25	25.00	12.50	0.08	0.44
6.25	12.50	12.50	0.33	2.12
6.25	6.25	12.50	0.48	3.29
6.25	3.13	12.50	0.49	3.13
6.25	1.56	12.50	0.51	3.16
6.25	0.78	12.50	0.48	2.77
3.13	50.00	12.50	0.18	0.98
3.13	25.00	12.50	0.31	1.58
3.13	12.50	12.50	0.50	2.95
3.13	6.25	12.50	0.68	4.96
3.13	3.13	12.50	0.65	3.42
3.13	1.56	12.50	0.70	3.64
3.13	0.78	12.50	0.70	3.29
1.56	50.00	12.50	0.26	1.51
1.56	25.00	12.50	0.38	1.90
1.56	12.50	12.50	0.59	3.67
1.56	6.25	12.50	0.69	3.97
1.56	3.13	12.50	0.61	1.81
1.56	1.56	12.50	0.66	1.78
1.56	0.78	12.50	0.69	1.69
0.78	50.00	12.50	0.36	2.61
0.78	25.00	12.50	0.44	2.40
0.78	12.50	12.50	0.62	3.79
0.78	6.25	12.50	0.82	9.43
0.78	3.13	12.50	0.71	2.27
0.78	1.56	12.50	0.74	1.88
0.78	0.78	12.50	0.81	2.48
50.00	50.00	25.00	0.03	1.52

50.00	25.00	25.00	0.03	1.38
50.00	12.50	25.00	0.02	0.90
50.00	6.25	25.00	0.03	1.17
50.00	3.13	25.00	0.02	1.04
50.00	1.56	25.00	0.02	0.83
50.00	0.78	25.00	0.01	0.31
25.00	50.00	25.00	0.02	0.54
25.00	25.00	25.00	0.01	0.31
25.00	12.50	25.00	0.02	0.38
25.00	6.25	25.00	0.02	0.45
25.00	3.13	25.00	0.02	0.38
25.00	1.56	25.00	0.02	0.38
25.00	0.78	25.00	0.01	0.31
12.50	50.00	25.00	0.03	0.39
12.50	25.00	25.00	0.03	0.30
12.50	12.50	25.00	0.02	0.25
12.50	6.25	25.00	0.03	0.31
12.50	3.13	25.00	0.02	0.21
12.50	1.56	25.00	0.02	0.23
12.50	0.78	25.00	0.01	0.17
6.25	50.00	25.00	0.07	0.43
6.25	25.00	25.00	0.08	0.48
6.25	12.50	25.00	0.43	3.26
6.25	6.25	25.00	0.56	4.60
6.25	3.13	25.00	0.54	3.69
6.25	1.56	25.00	0.59	4.15
6.25	0.78	25.00	0.48	2.76
3.13	50.00	25.00	0.22	1.36
3.13	25.00	25.00	0.35	2.04

3.13	12.50	25.00	0.54	3.60
3.13	6.25	25.00	0.74	7.15
3.13	3.13	25.00	0.68	3.81
3.13	1.56	25.00	0.66	2.97
3.13	0.78	25.00	0.70	3.31
1.56	50.00	25.00	0.29	1.83
1.56	25.00	25.00	0.36	1.70
1.56	12.50	25.00	0.60	3.89
1.56	6.25	25.00	0.71	4.54
1.56	3.13	25.00	0.62	1.90
1.56	1.56	25.00	0.64	1.60
1.56	0.78	25.00	0.69	1.75
0.78	50.00	25.00	0.30	1.81
0.78	25.00	25.00	0.43	2.19
0.78	12.50	25.00	0.62	3.80
0.78	6.25	25.00	0.84	13.25
0.78	3.13	25.00	0.73	2.62
0.78	1.56	25.00	0.79	3.06
0.78	0.78	25.00	0.80	2.47
50.00	50.00	50.00	0.03	1.26
50.00	25.00	50.00	0.02	0.84
50.00	12.50	50.00	0.02	0.76
50.00	6.25	50.00	0.02	1.10
50.00	3.13	50.00	0.02	1.04
50.00	1.56	50.00	0.02	0.90
50.00	0.78	50.00	0.01	0.69
25.00	50.00	50.00	0.03	0.74
25.00	25.00	50.00	0.02	0.46
25.00	12.50	50.00	0.03	0.59

25.00	6.25	50.00	0.01	0.35
25.00	3.13	50.00	0.01	0.35
25.00	1.56	50.00	0.01	0.35
25.00	0.78	50.00	0.01	0.35
12.50	50.00	50.00	0.02	0.22
12.50	25.00	50.00	0.02	0.25
12.50	12.50	50.00	0.02	0.25
12.50	6.25	50.00	0.01	0.17
12.50	3.13	50.00	0.01	0.14
12.50	1.56	50.00	0.01	0.17
12.50	0.78	50.00	0.01	0.14
6.25	50.00	50.00	0.05	0.34
6.25	25.00	50.00	0.06	0.34
6.25	12.50	50.00	0.07	0.37
6.25	6.25	50.00	0.53	4.07
6.25	3.13	50.00	0.46	2.86
6.25	1.56	50.00	0.46	2.67
6.25	0.78	50.00	0.43	2.36
3.13	50.00	50.00	0.15	0.79
3.13	25.00	50.00	0.31	1.58
3.13	12.50	50.00	0.55	3.82
3.13	6.25	50.00	0.78	9.92
3.13	3.13	50.00	0.67	3.77
3.13	1.56	50.00	0.76	5.07
3.13	0.78	50.00	0.77	4.93
1.56	50.00	50.00	0.26	1.49
1.56	25.00	50.00	0.40	2.08
1.56	12.50	50.00	0.61	4.09
1.56	6.25	50.00	0.79	8.76

1.56	3.13	50.00	0.67	2.45
1.56	1.56	50.00	0.78	4.21
1.56	0.78	50.00	0.77	2.93
0.78	50.00	50.00	0.33	2.21
0.78	25.00	50.00	0.44	2.40
0.78	12.50	50.00	0.55	2.41
0.78	6.25	50.00	0.84	14.64
0.78	3.13	50.00	0.76	3.43
0.78	1.56	50.00	0.82	5.59
0.78	0.78	50.00	0.78	2.44

The original images of Western blots

Figure 3

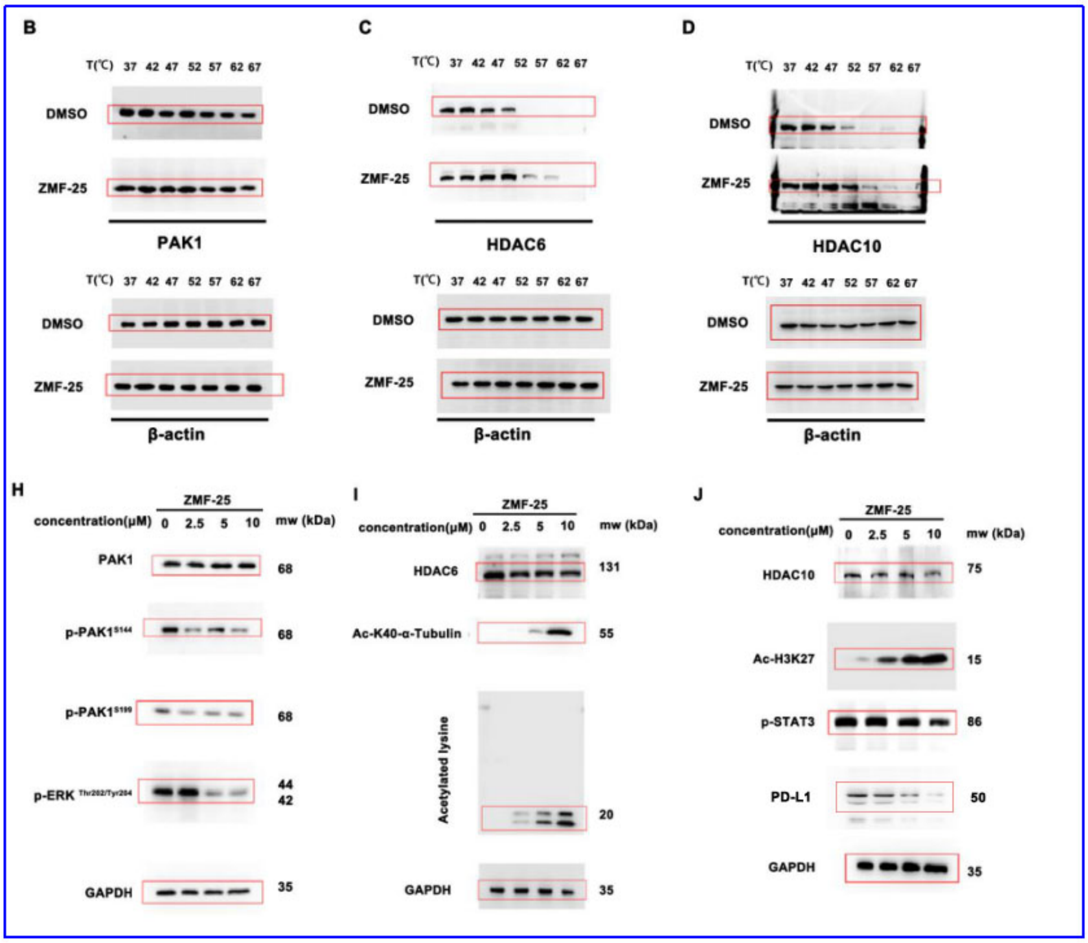

Figure 4

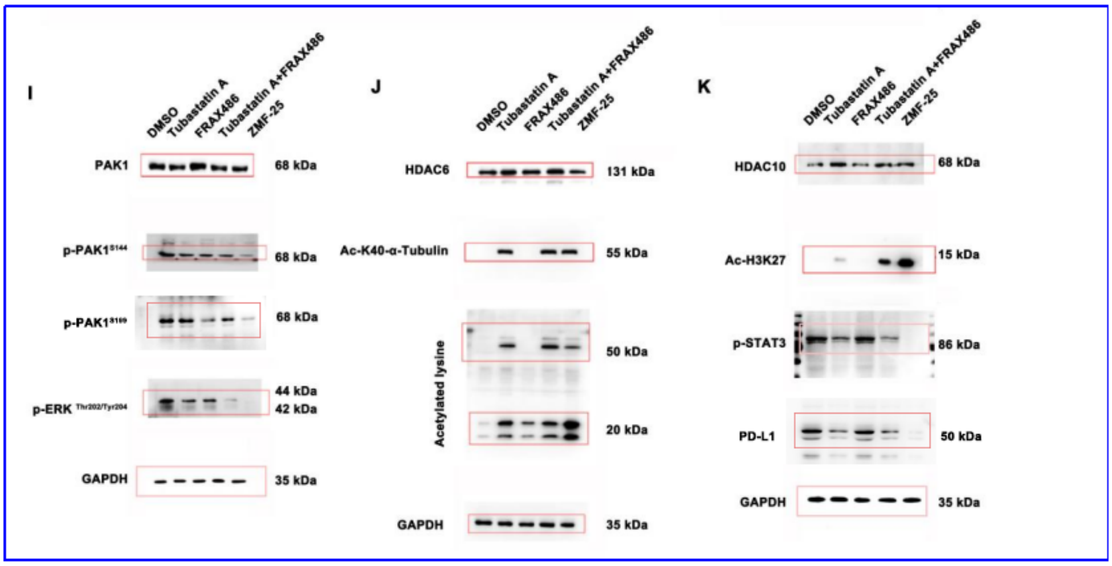

Figure 5

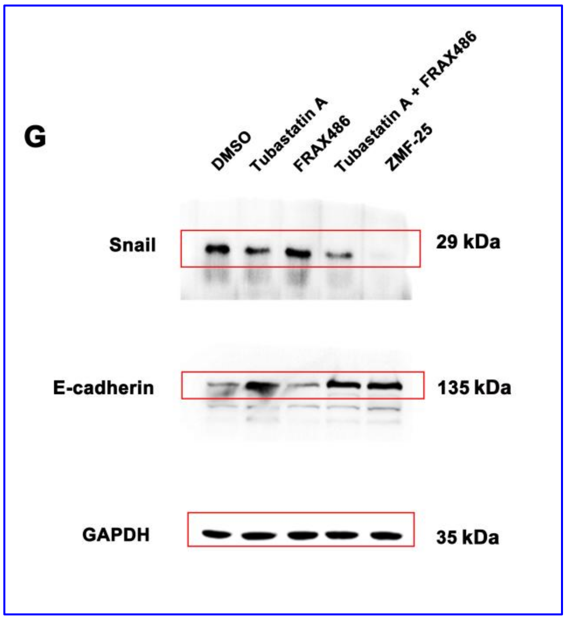

Figure 7

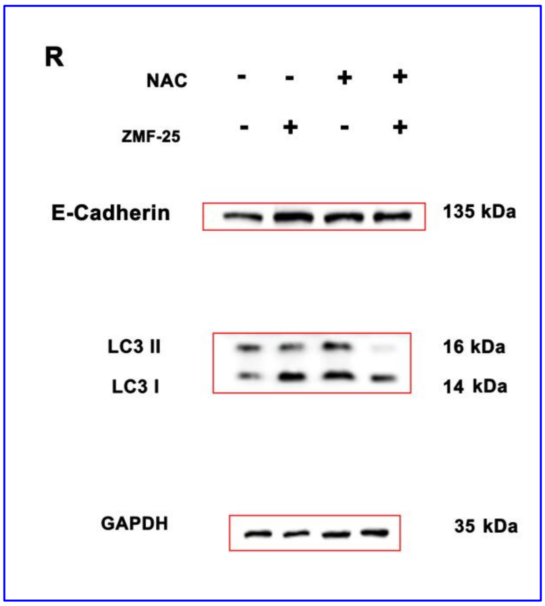

Figure 8

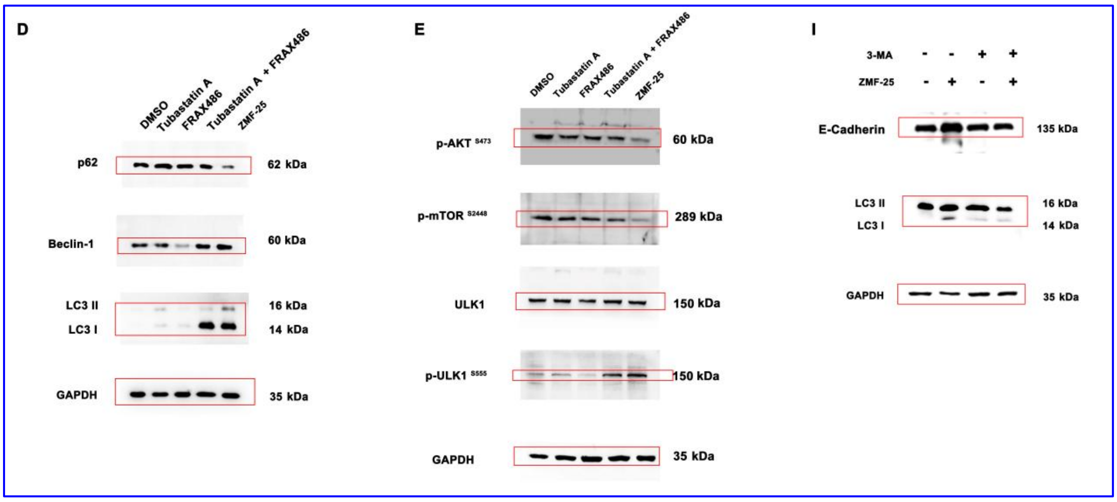

Figure 9

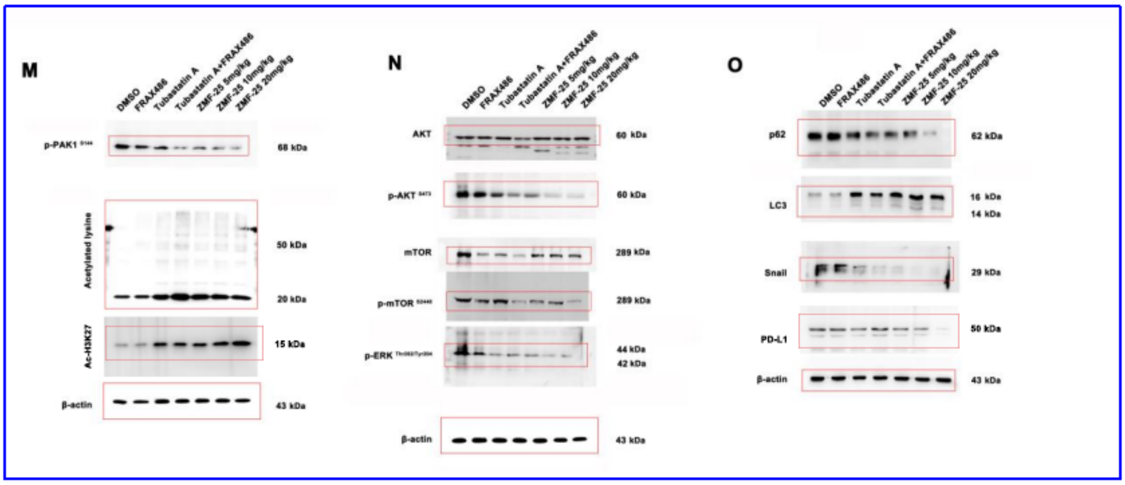

Figure S5

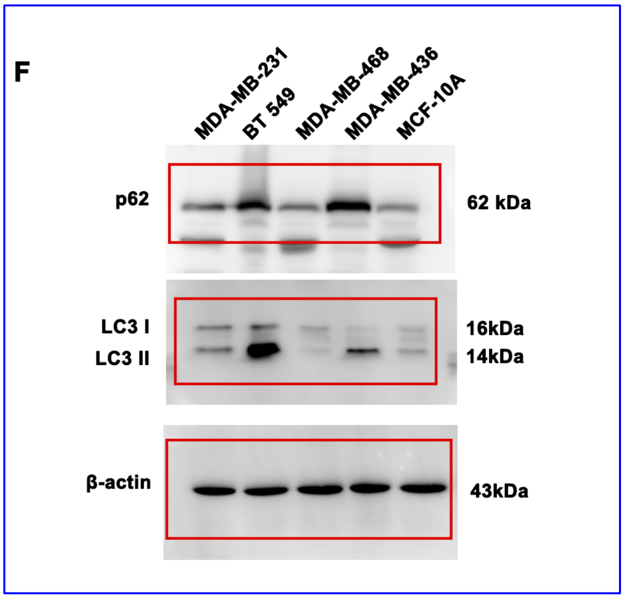

1196

^{1}H and ^{13}C NMR, HR-MS and HPLC Spectrum of Compounds

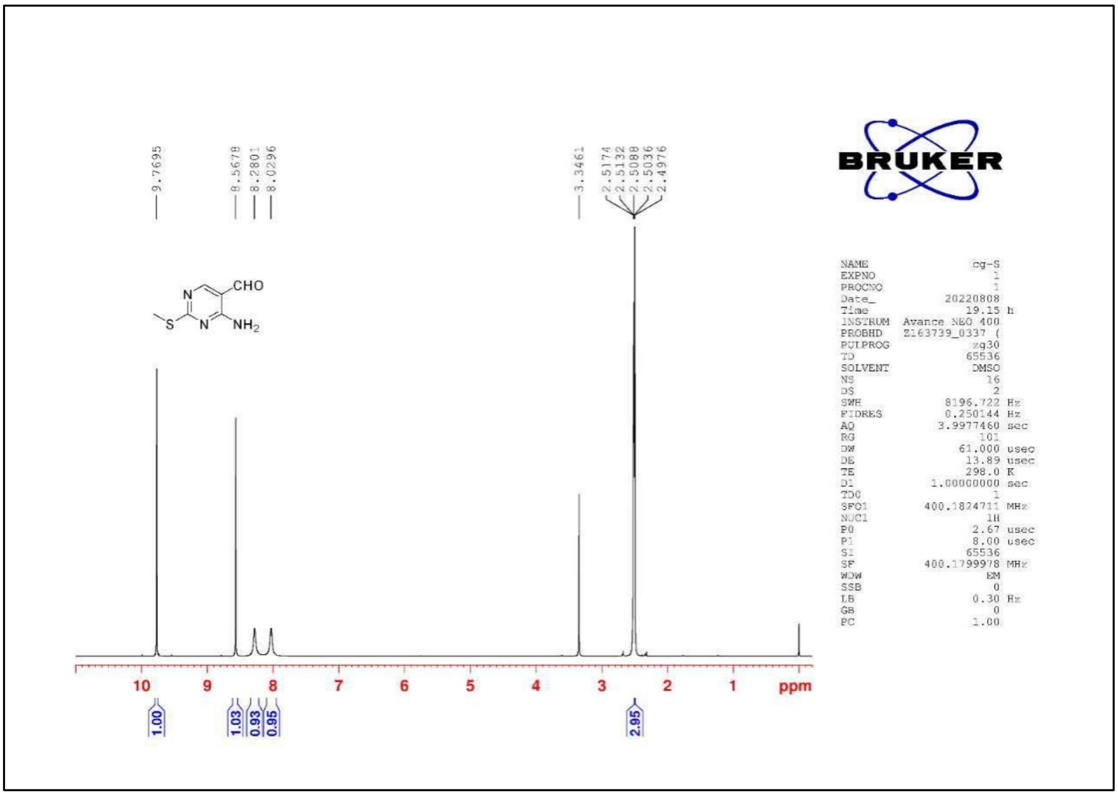

^{1}H-NMR spectrum of compound 11

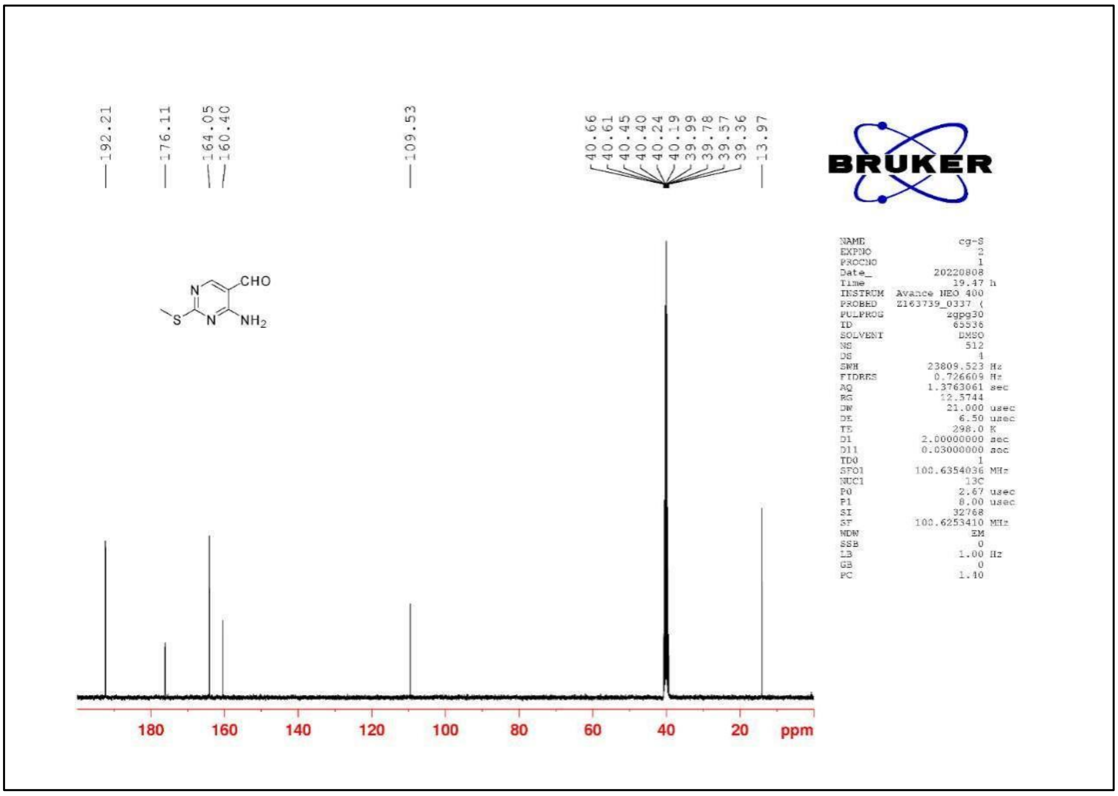

^{13}C-NMR spectrum of compound 11

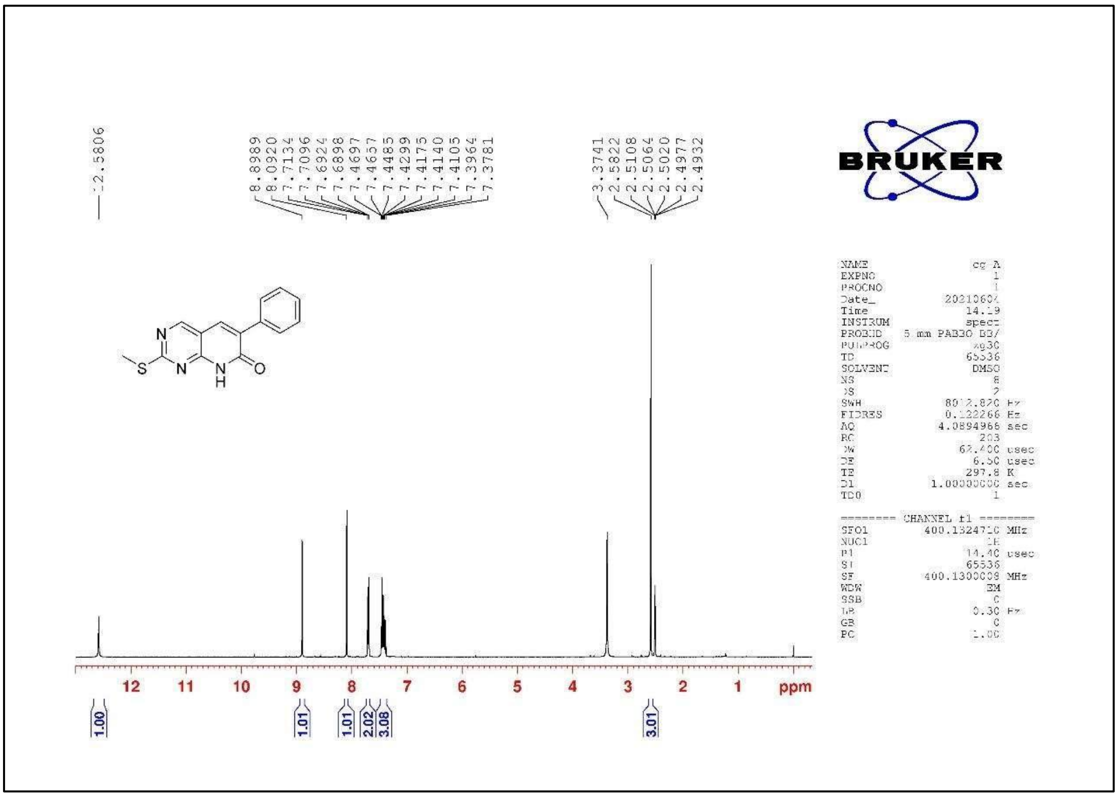

^{1}H-NMR spectrum of compound 13a

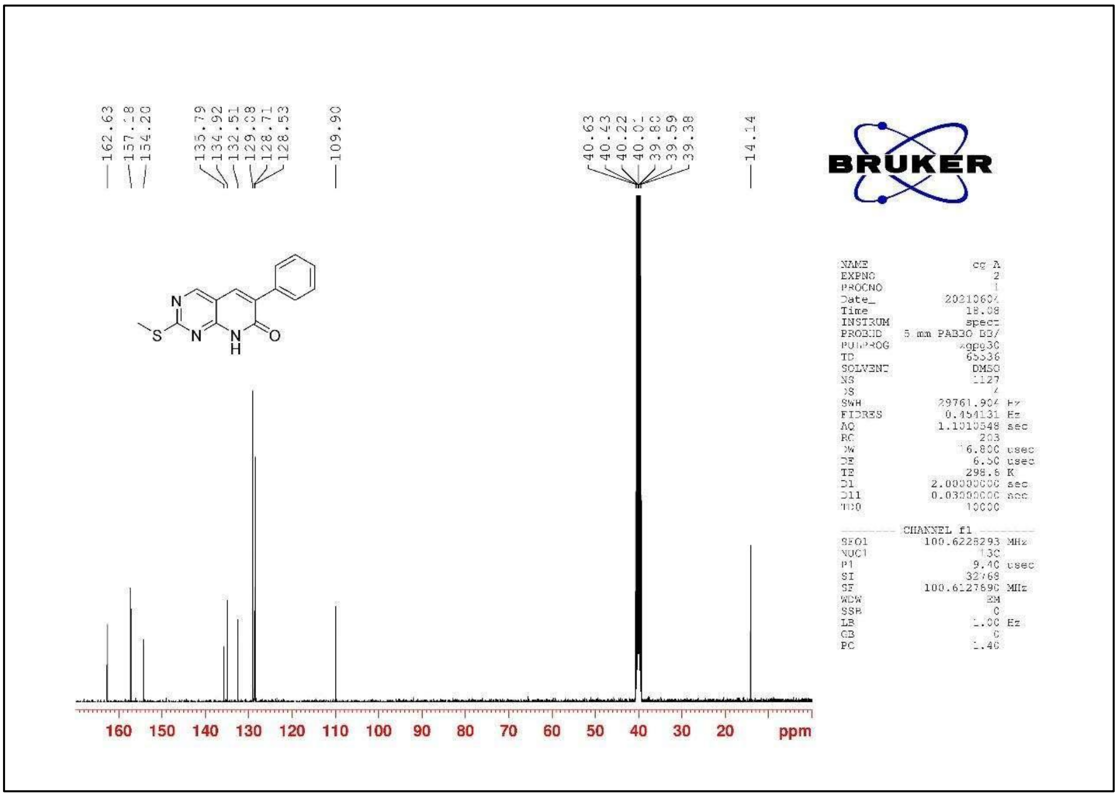

^{13}C-NMR spectrum of compound 13a

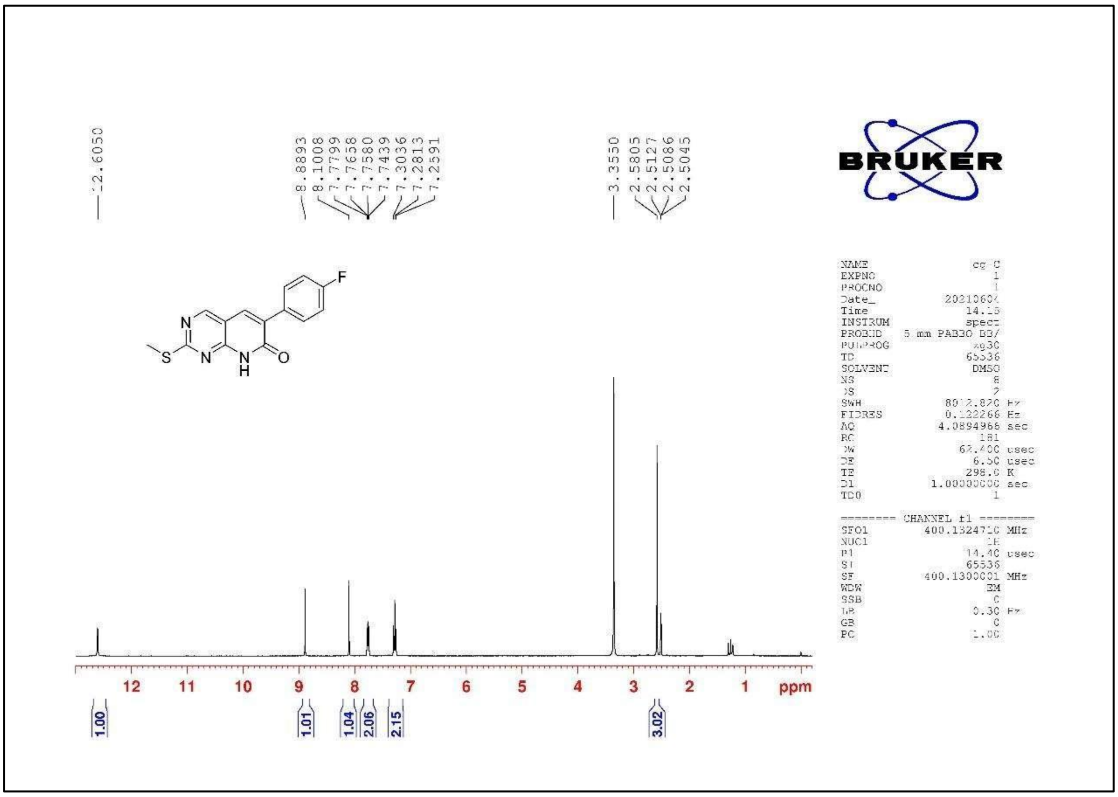

^{1}**H-NMR spectrum of compound 13b**

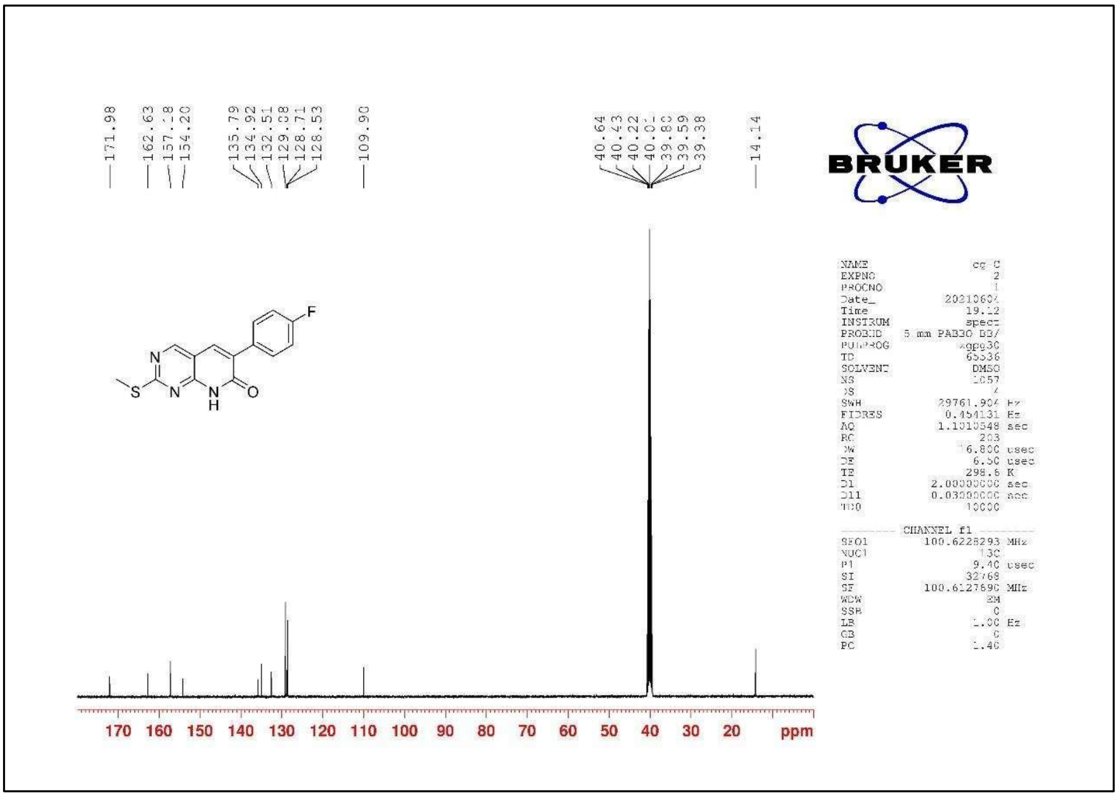

^{13}**C-NMR spectrum of compound 13b**

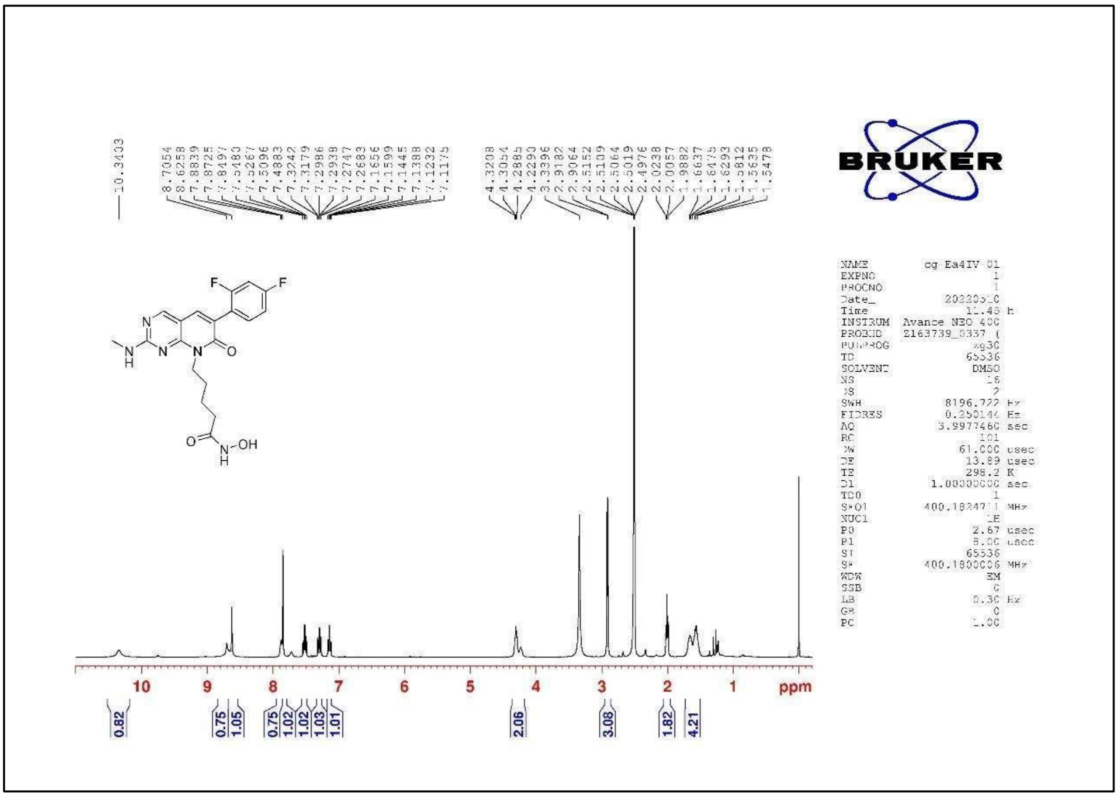

^{1}H-NMR spectrum of compound 13c

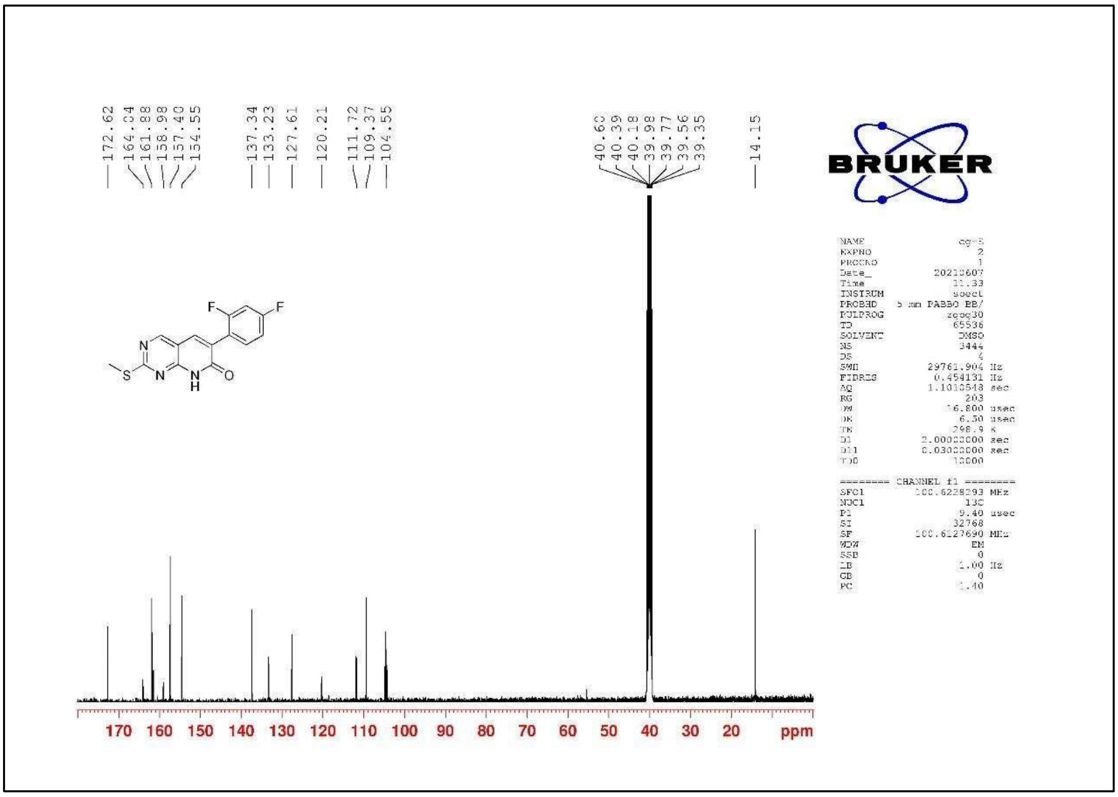

^{13}C-NMR spectrum of compound 13c

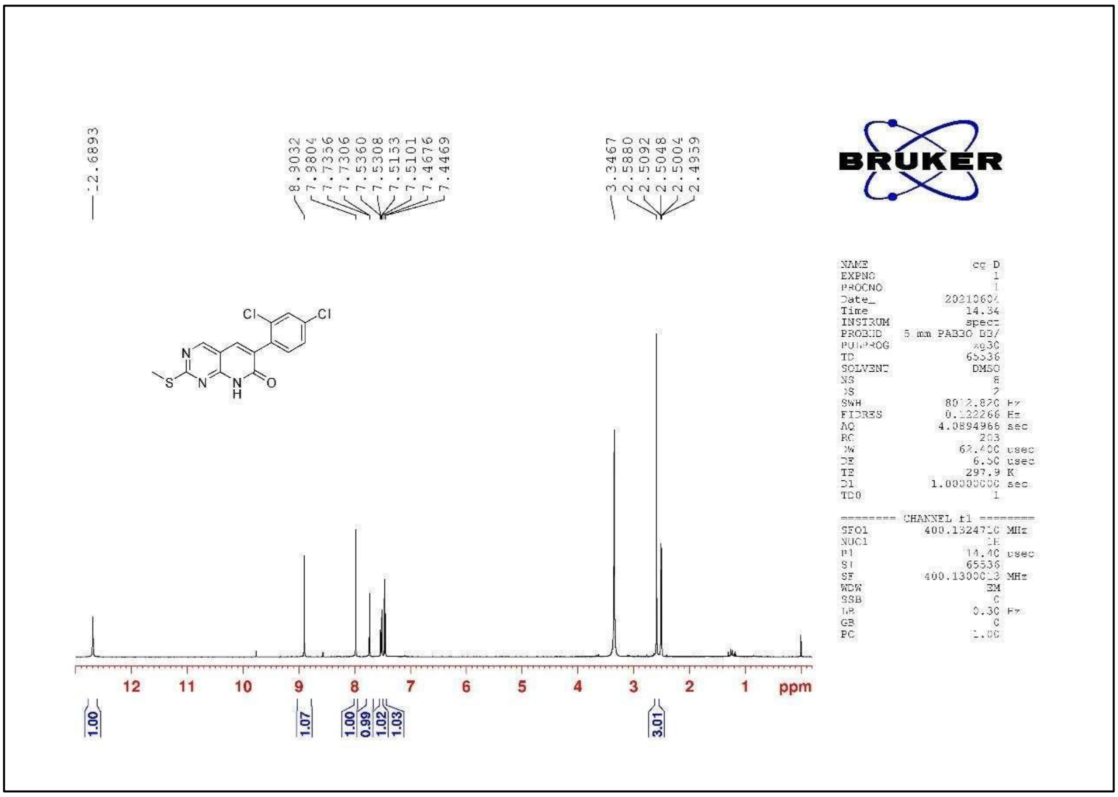

^{1}H-NMR spectrum of compound 13d

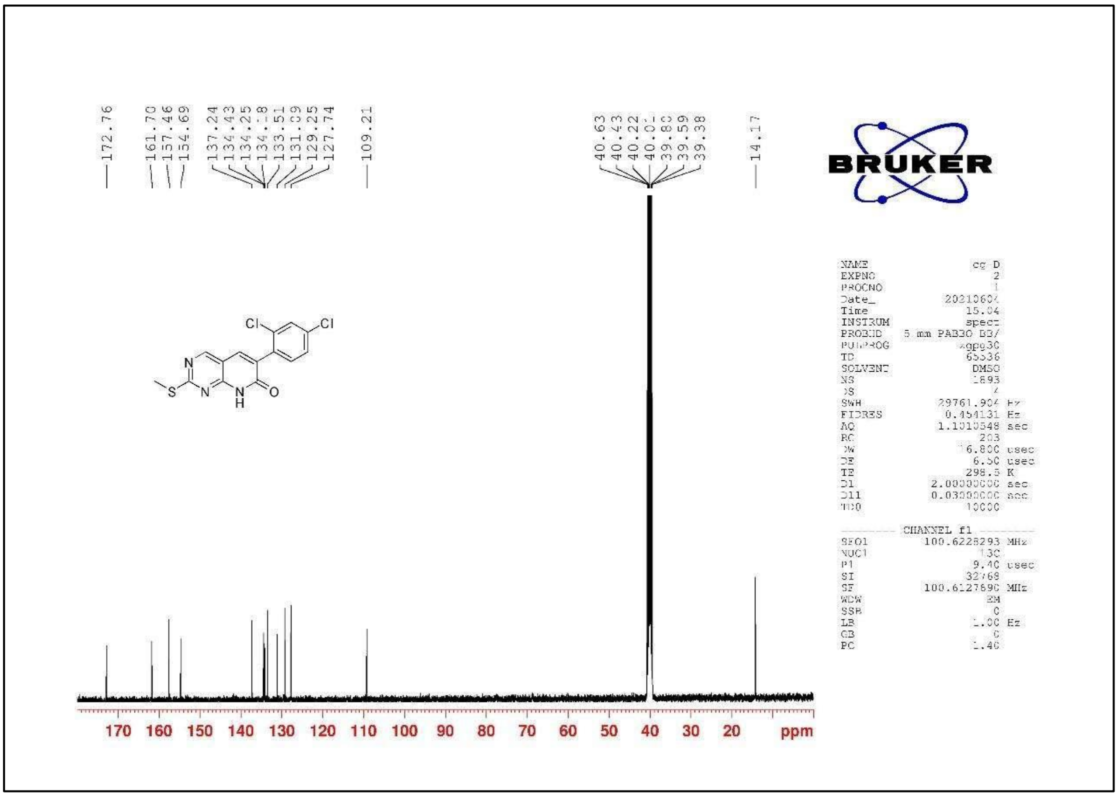

^{13}C-NMR spectrum of compound 13d

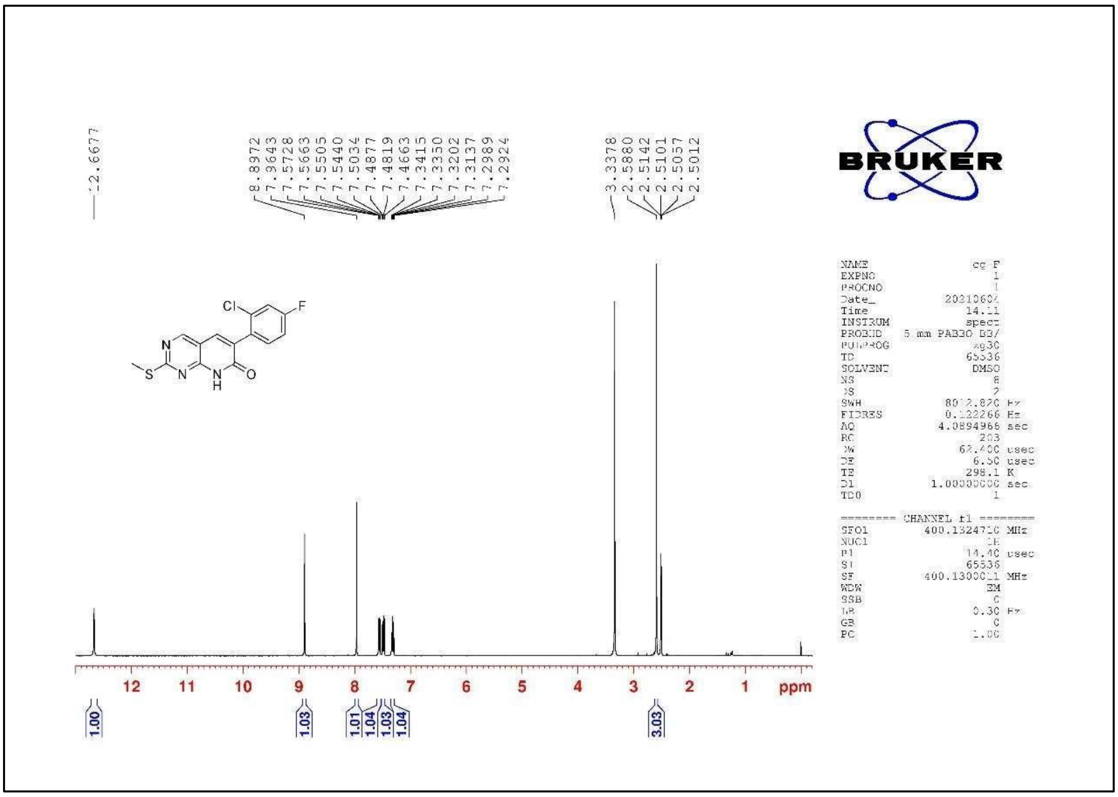

^{1}H-NMR spectrum of compound 13e

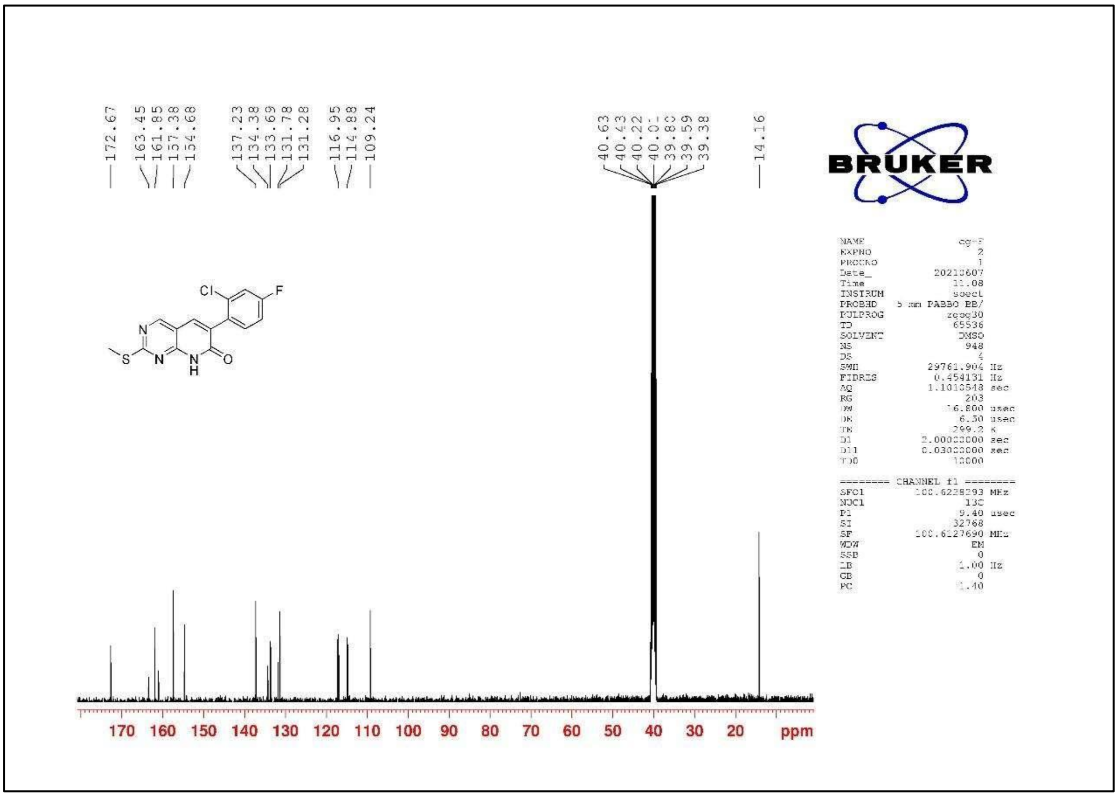

^{13}C-NMR spectrum of compound 13e

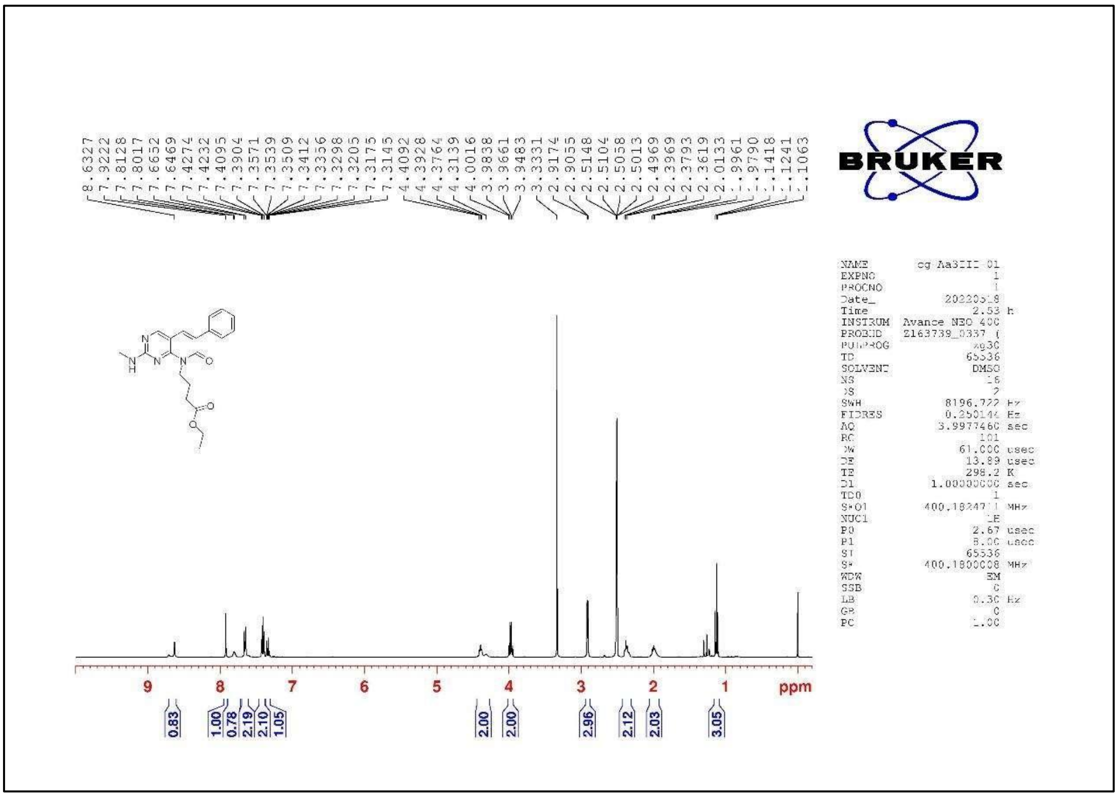

^{1}H-NMR spectrum of compound 17a

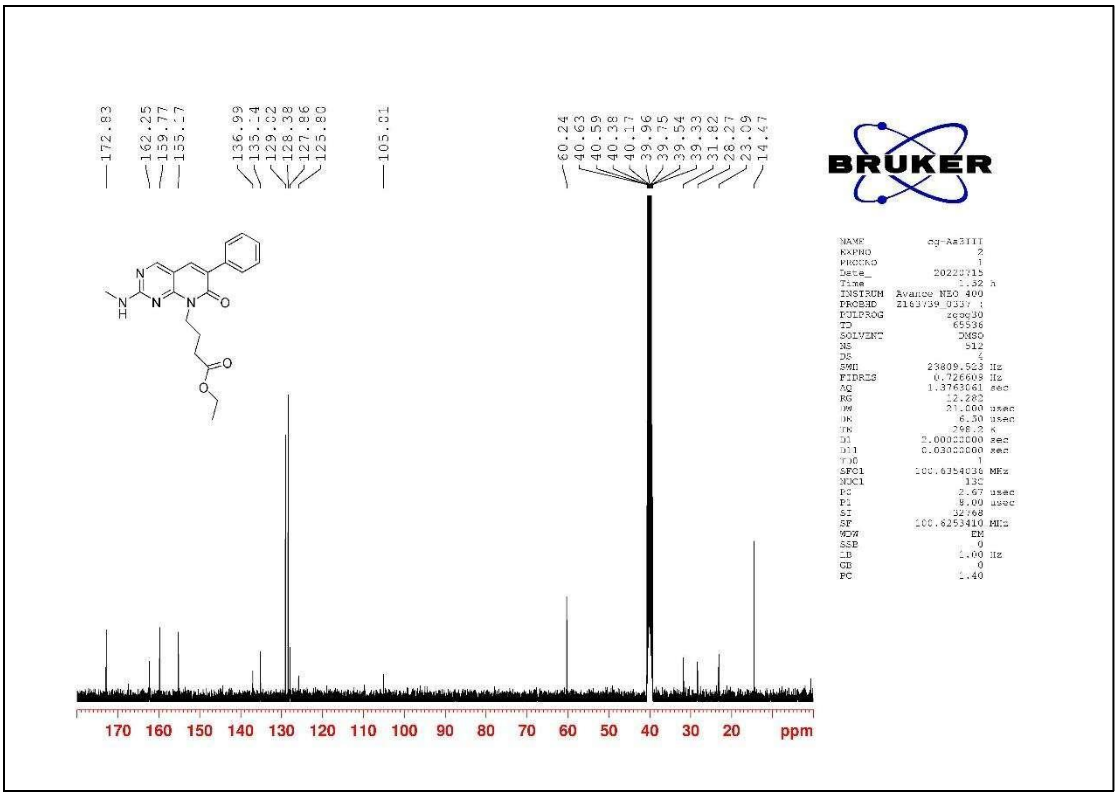

^{13}C-NMR spectrum of compound 17a

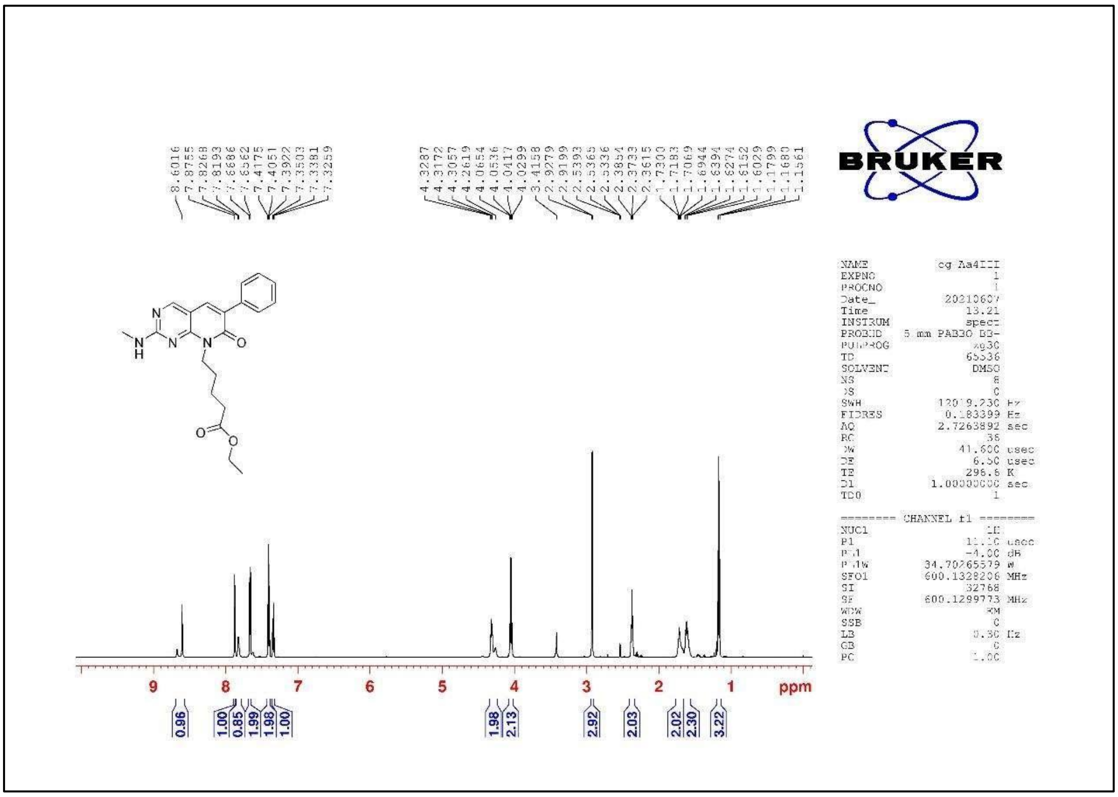

^{1}H-NMR spectrum of compound 17b

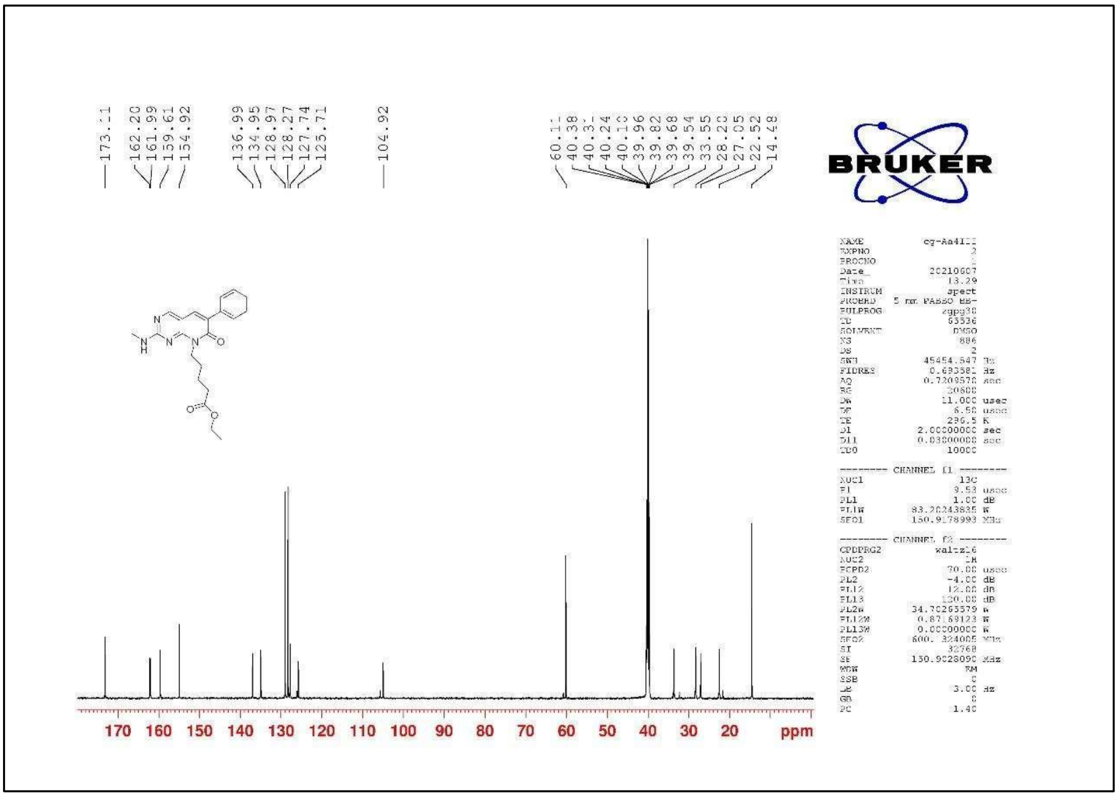

^{13}C-NMR spectrum of compound 17b

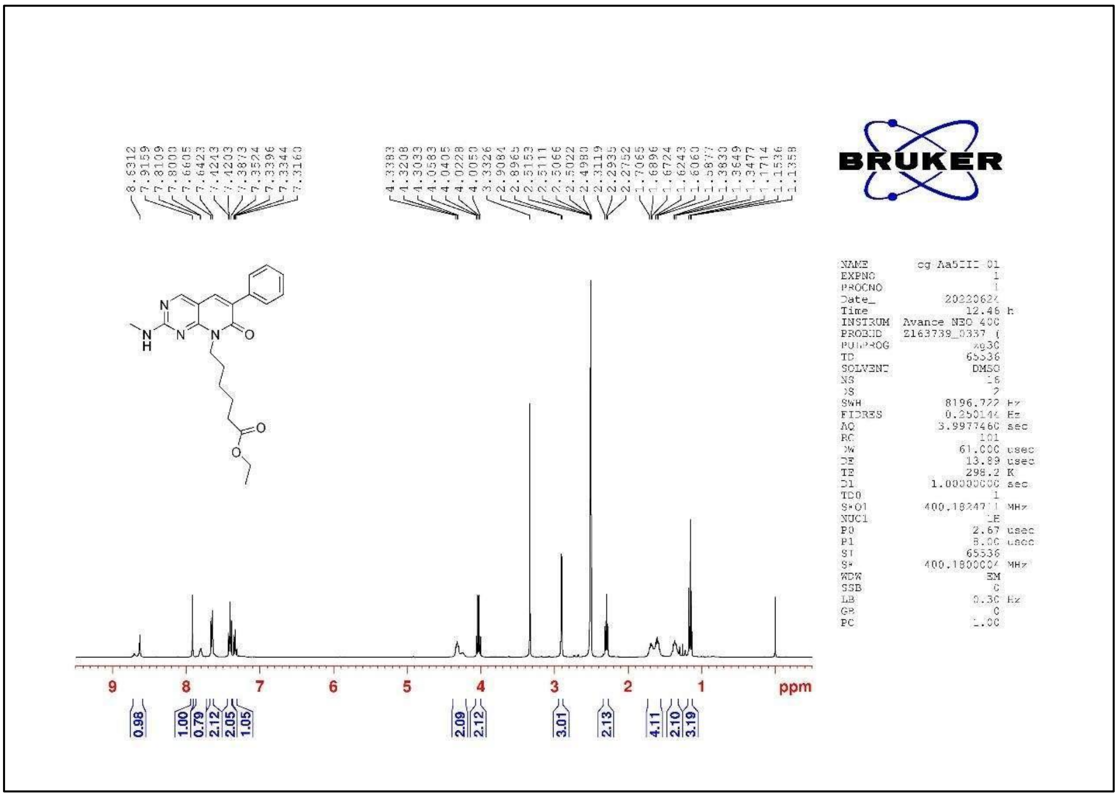

^{1}H-NMR spectrum of compound 17c

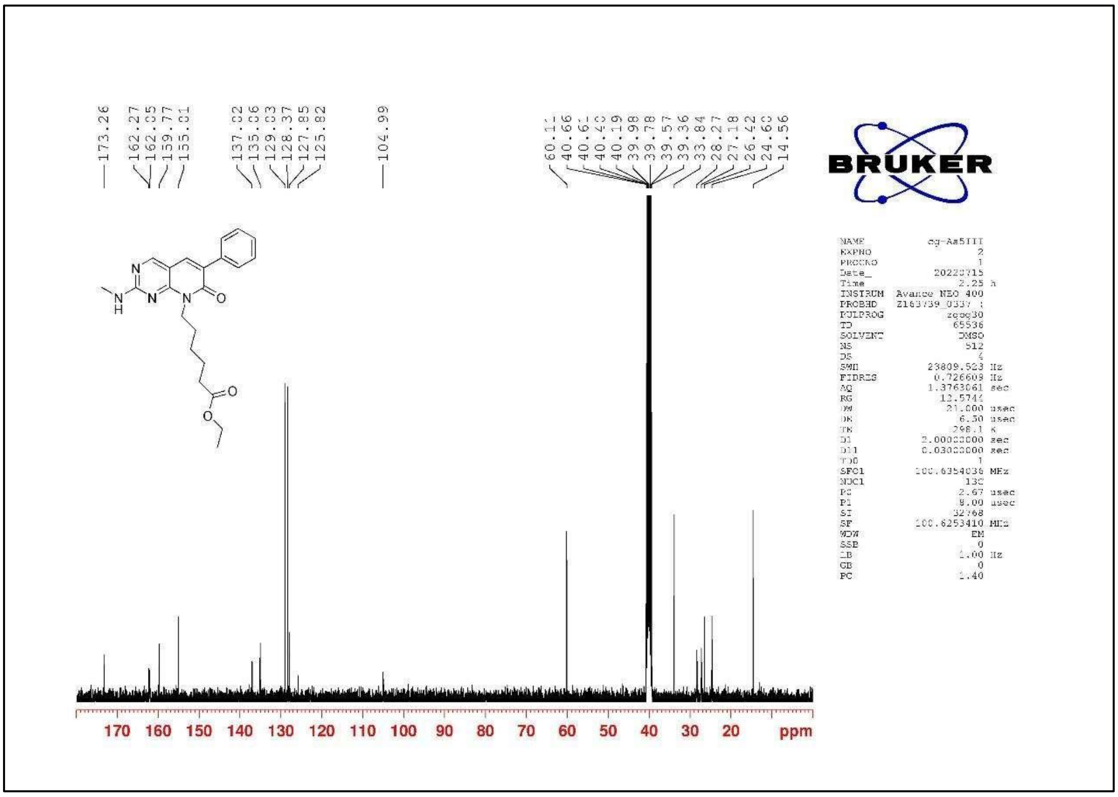

^{13}C-NMR spectrum of compound 17c

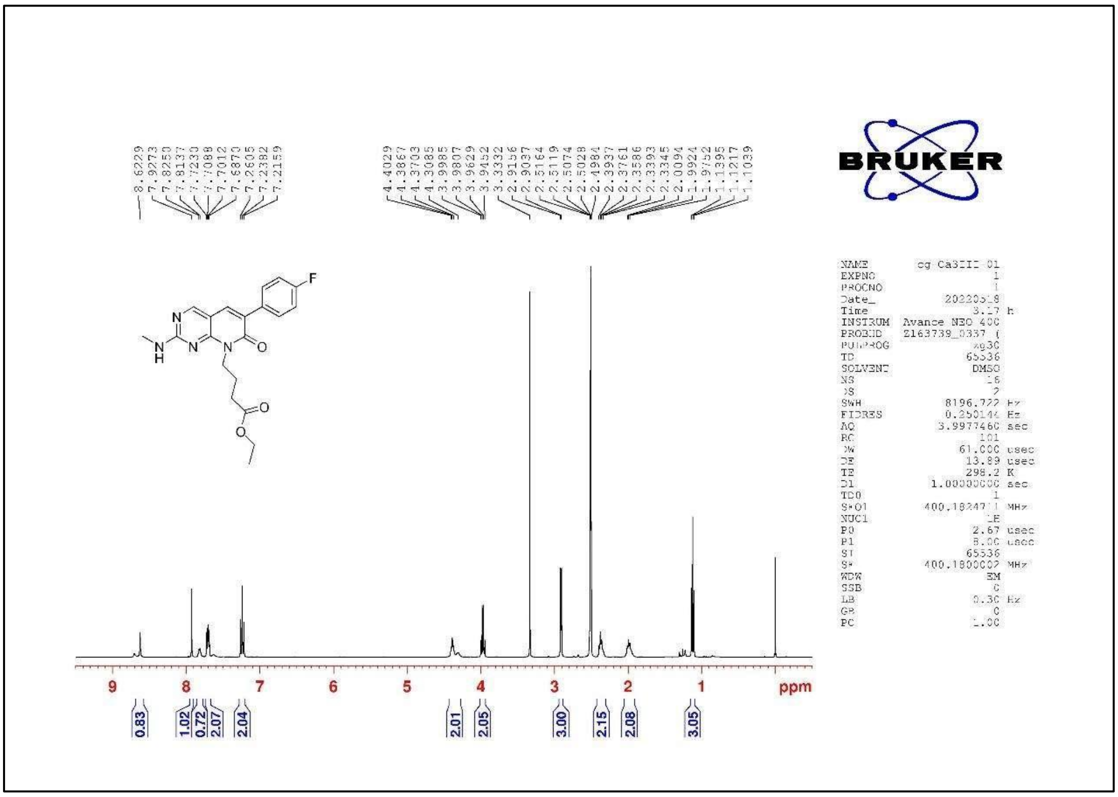

^{1}H-NMR spectrum of compound 17d

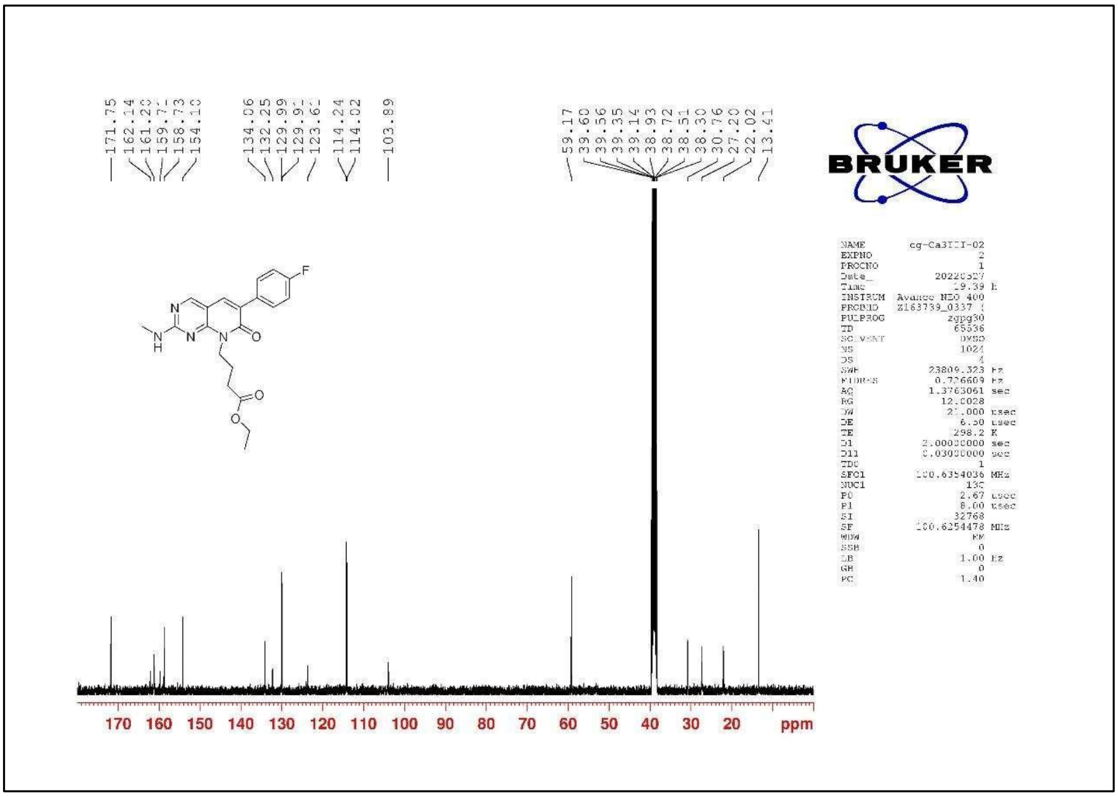

^{13}C-NMR spectrum of compound 17d

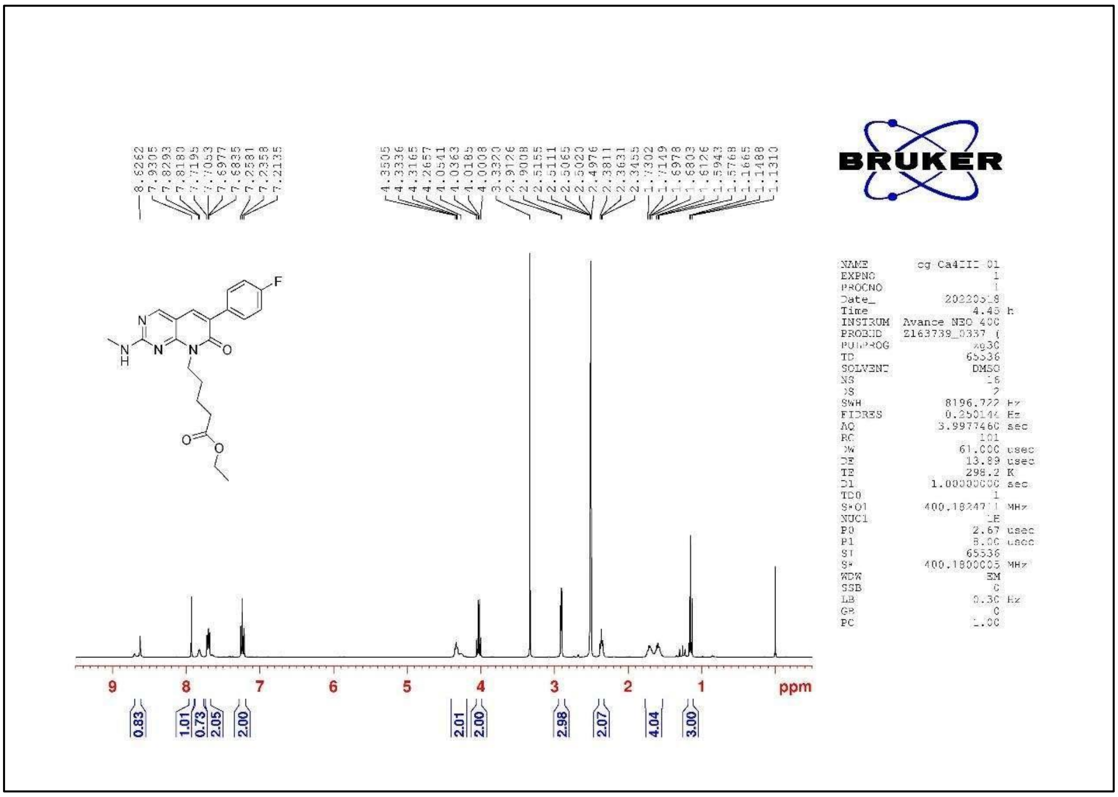

^{1}H-NMR spectrum of compound 17e

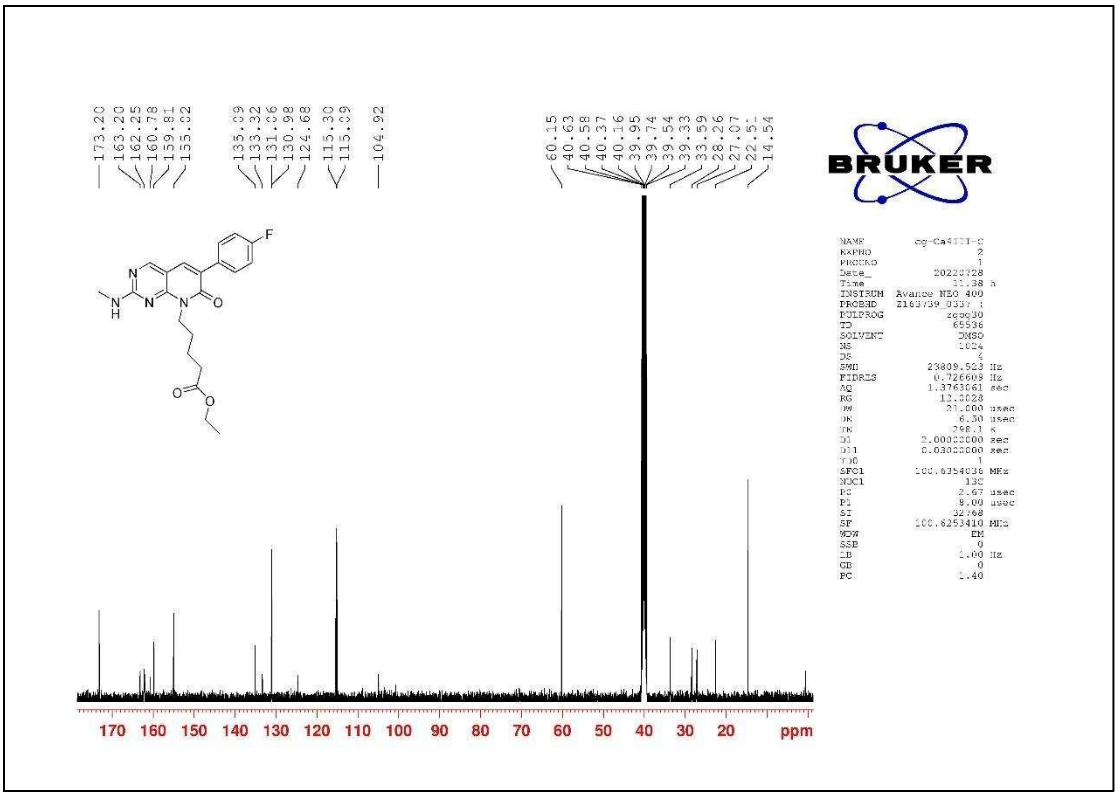

^{13}C-NMR spectrum of compound 17e

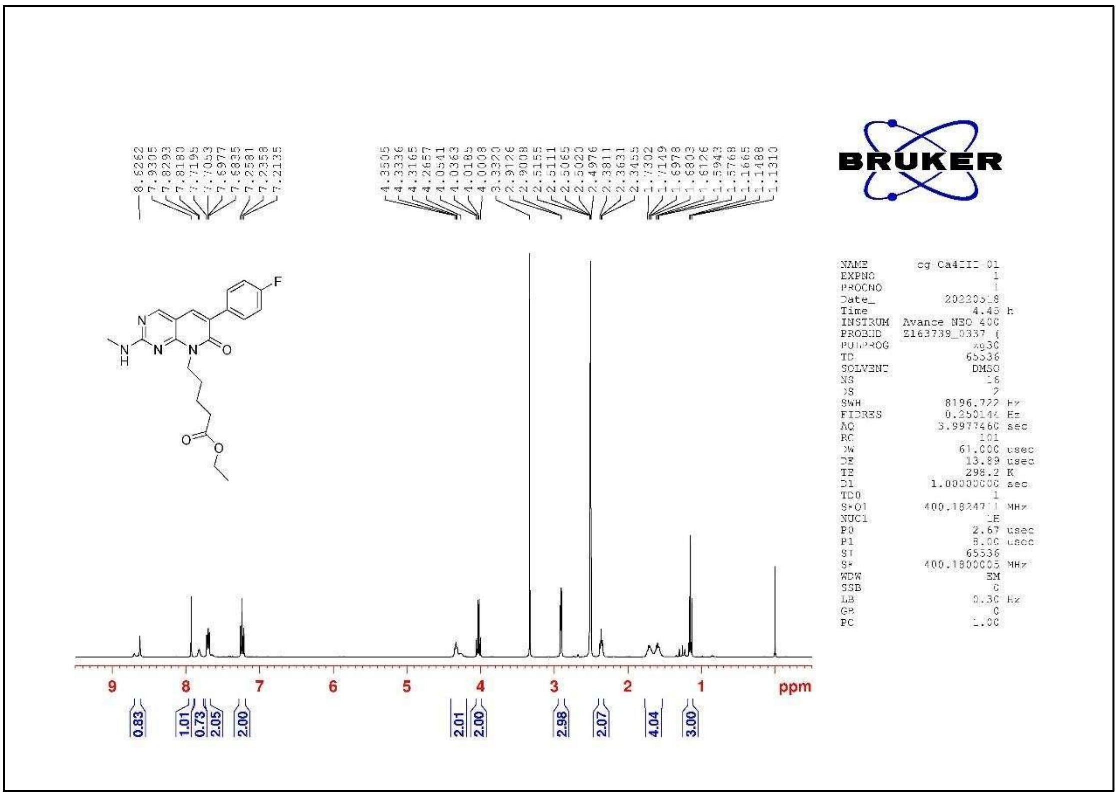

^{1}H-NMR spectrum of compound 17f

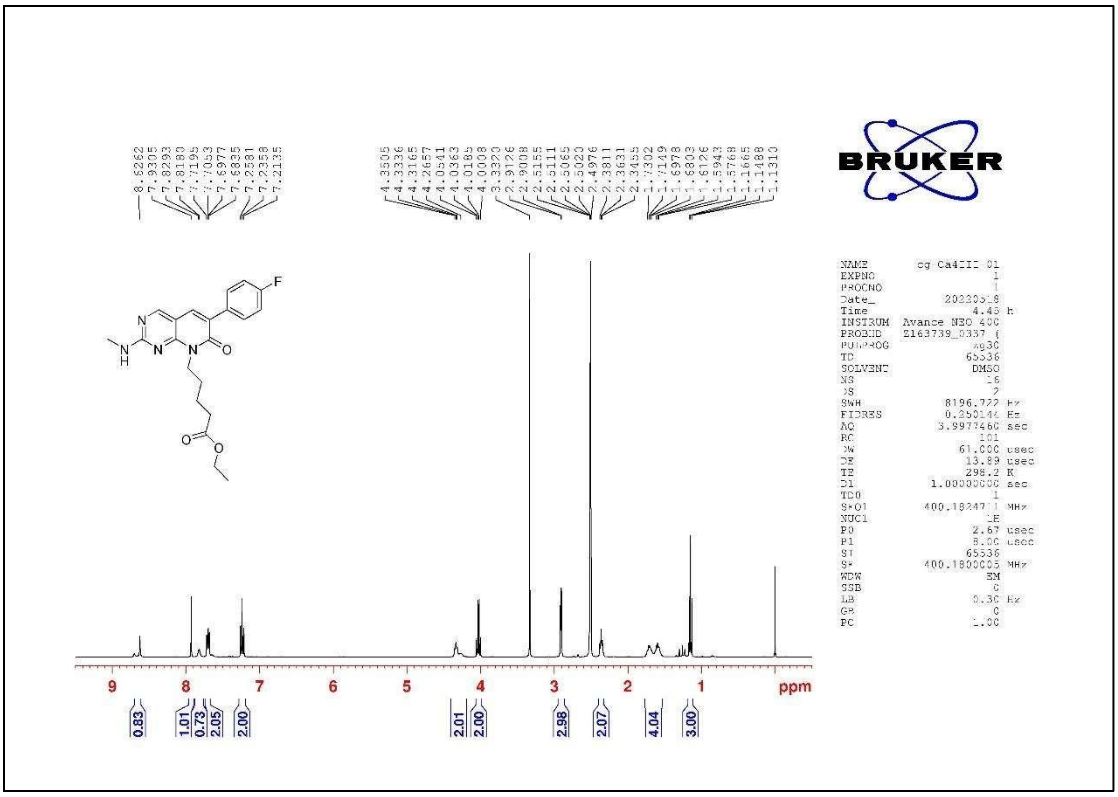

^{13}C-NMR spectrum of compound 17f

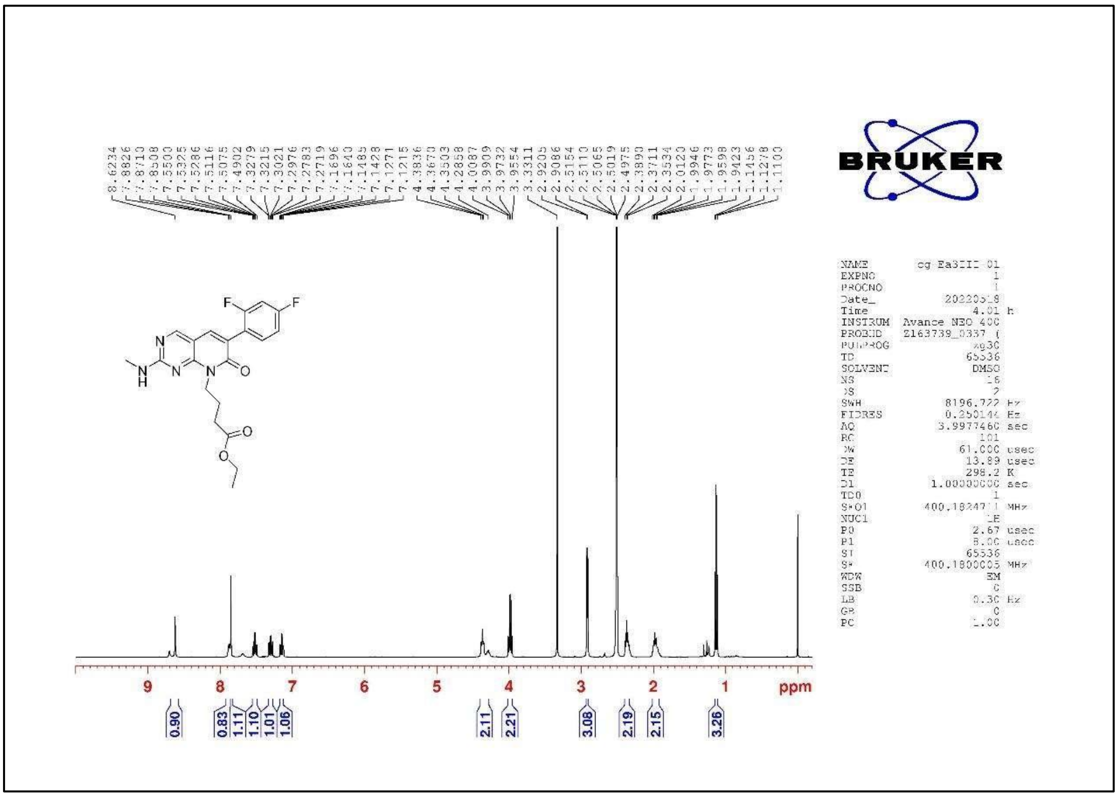

^{1}H-NMR spectrum of compound 17g

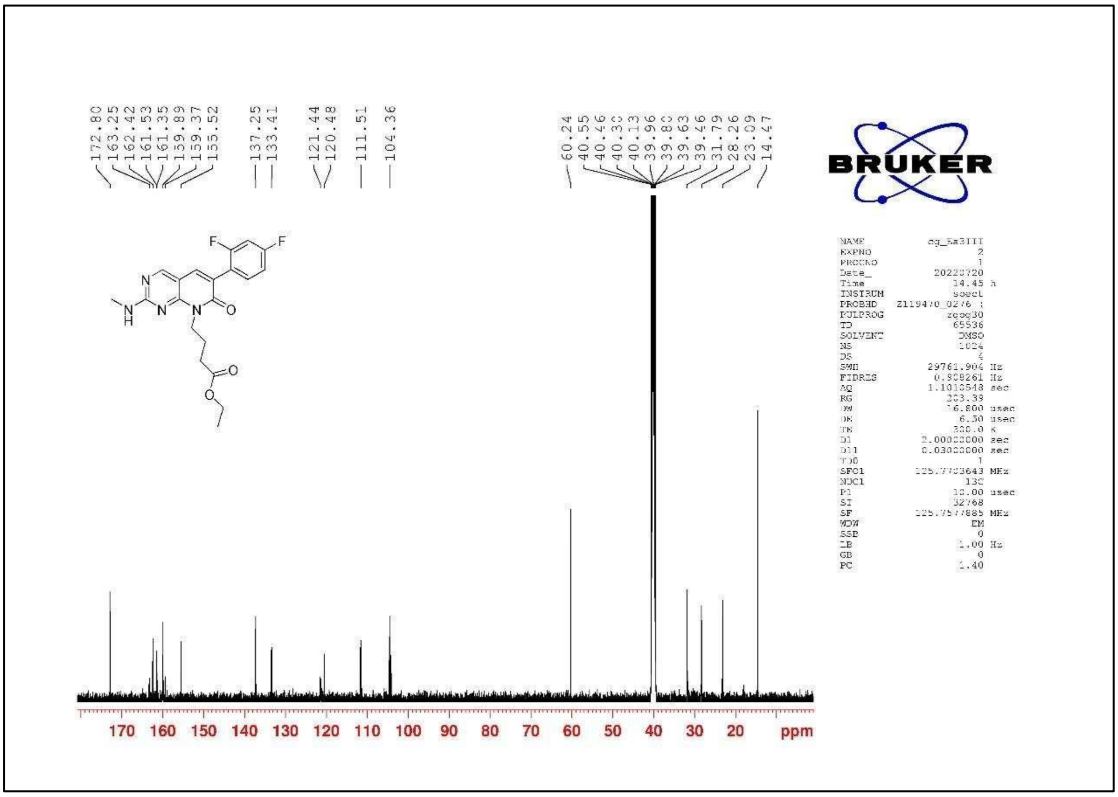

^{13}C-NMR spectrum of compound 17g

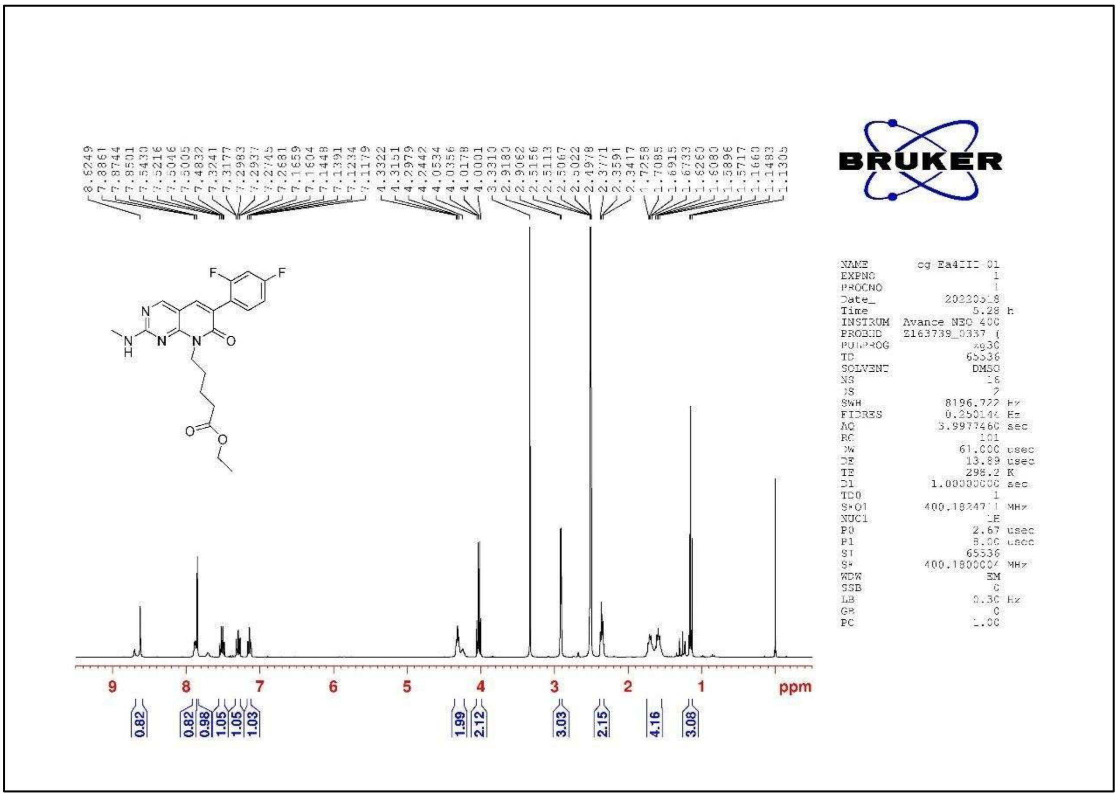

^{1}H-NMR spectrum of compound 17h

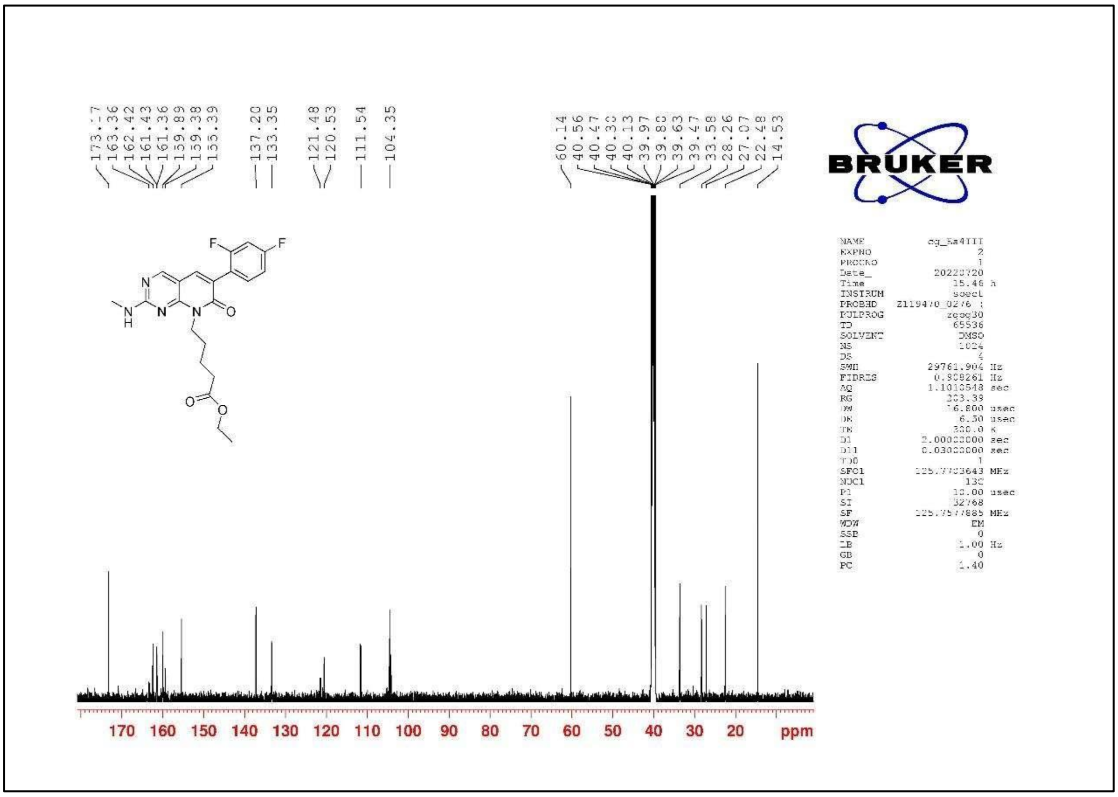

^{13}C-NMR spectrum of compound 17h

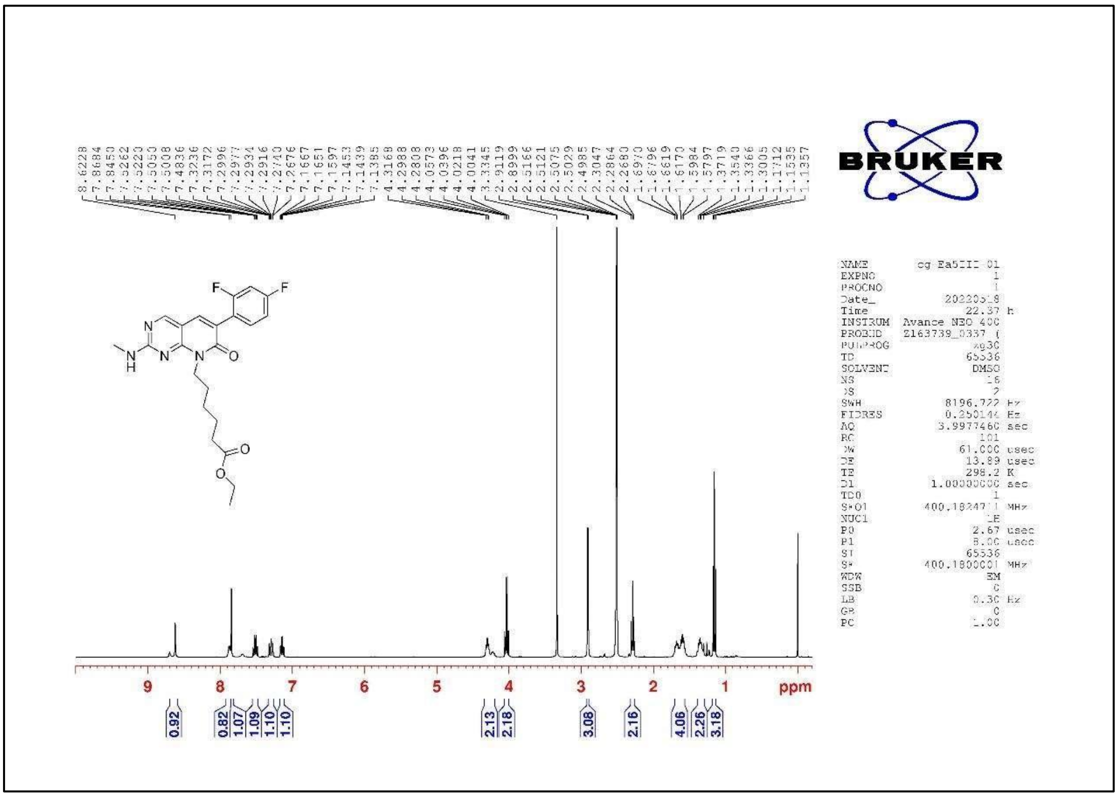

^{1}H-NMR spectrum of compound 17i

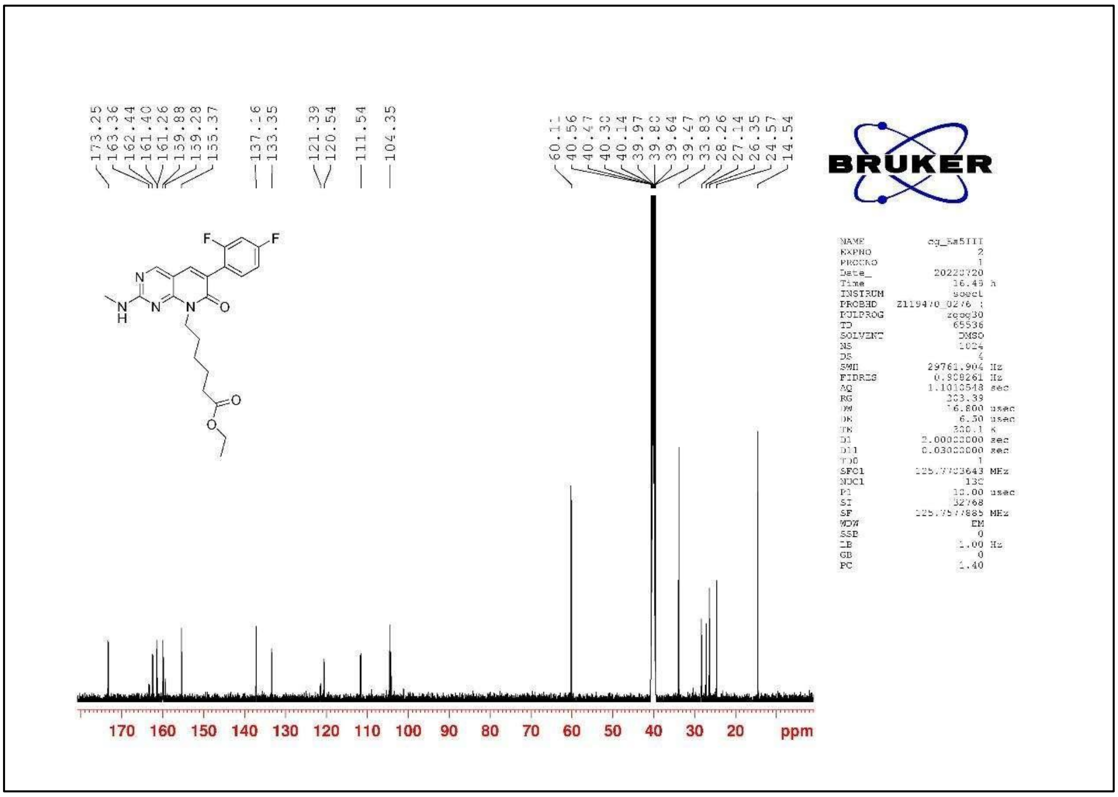

^{13}C-NMR spectrum of compound 17i

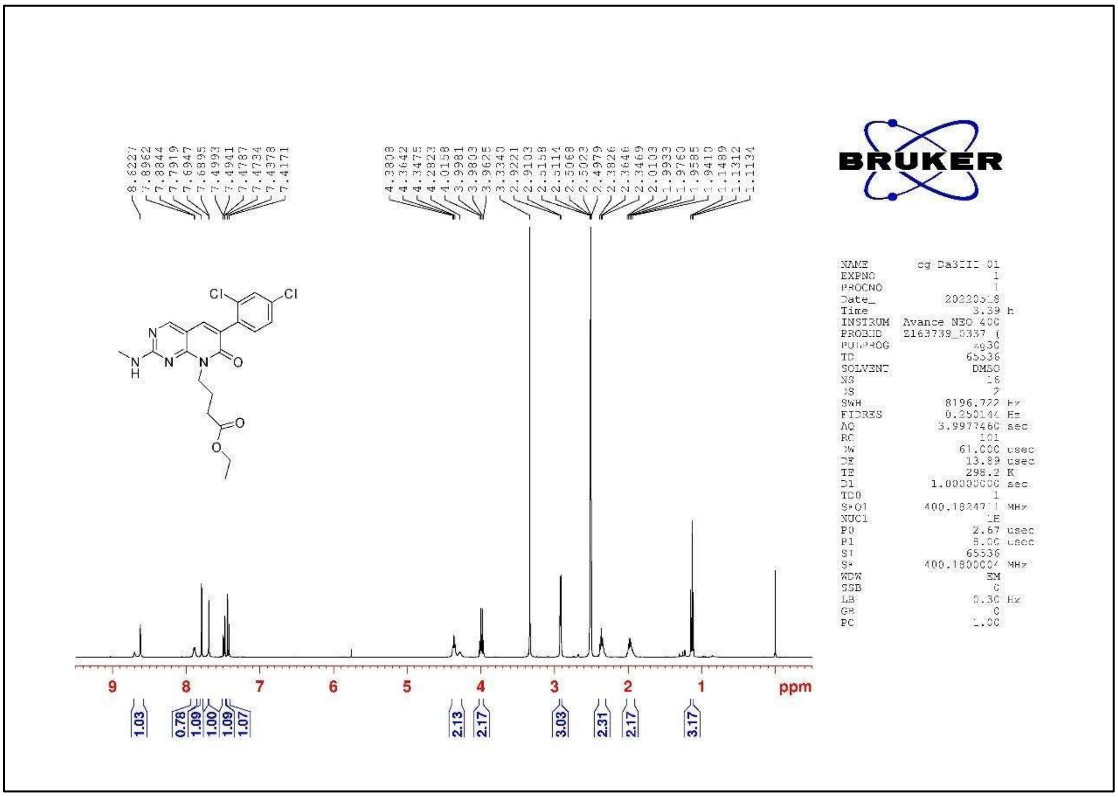

^{1}H-NMR spectrum of compound 17j

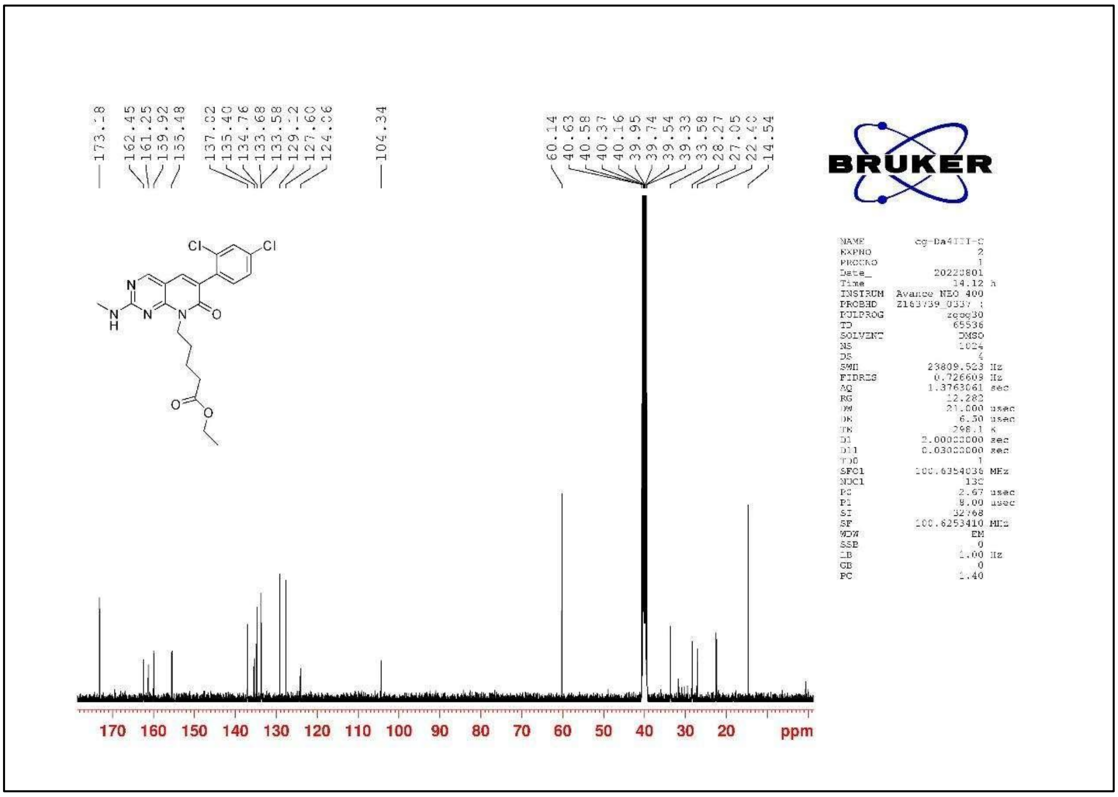

^{13}C-NMR spectrum of compound 17j

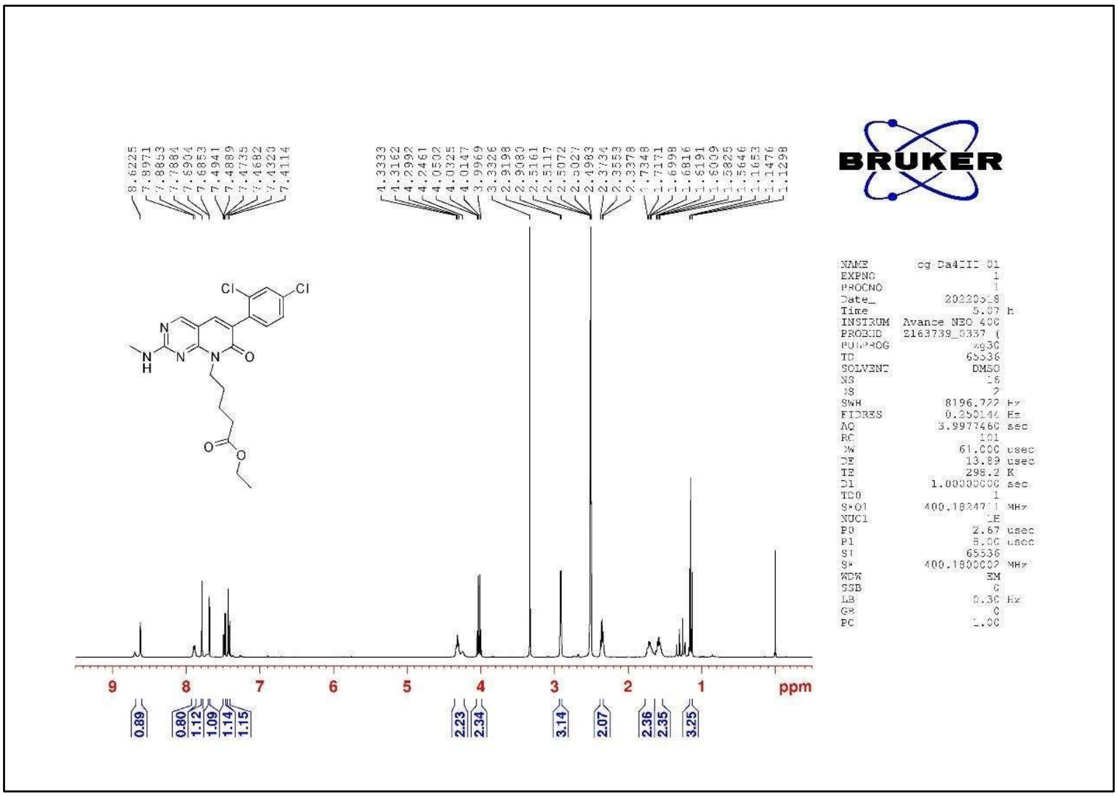

^{1}H-NMR spectrum of compound 17k

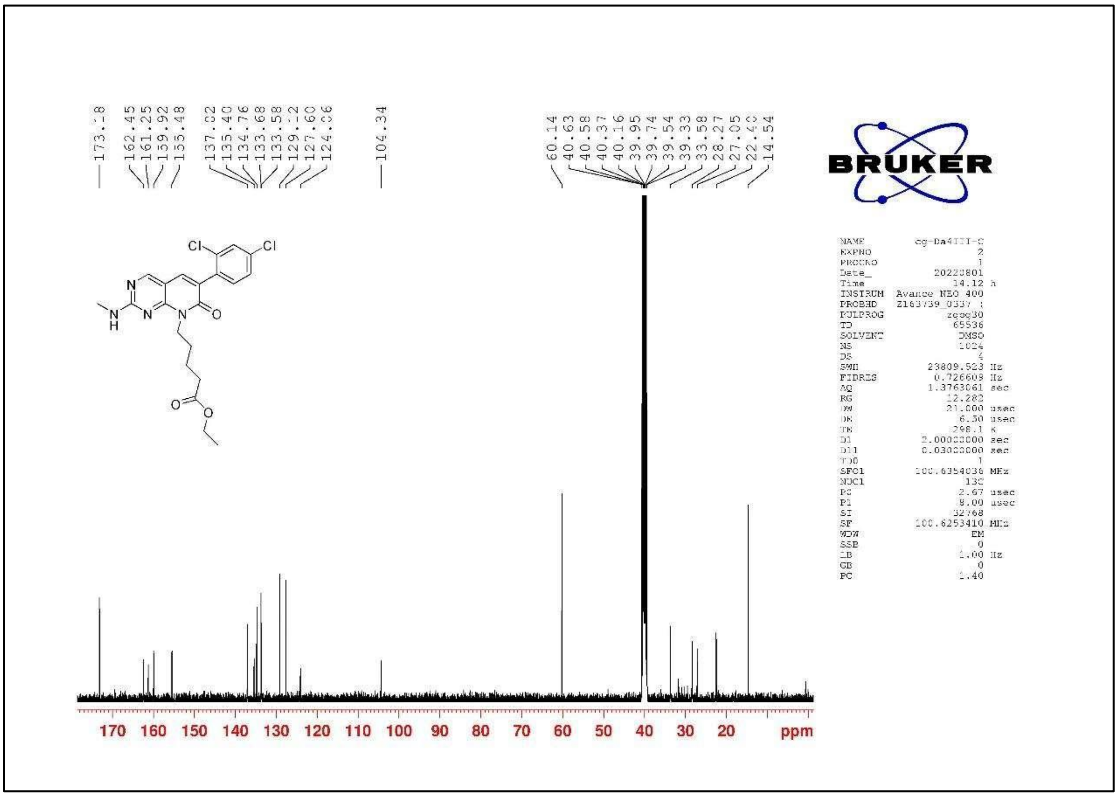

^{13}C-NMR spectrum of compound 17k

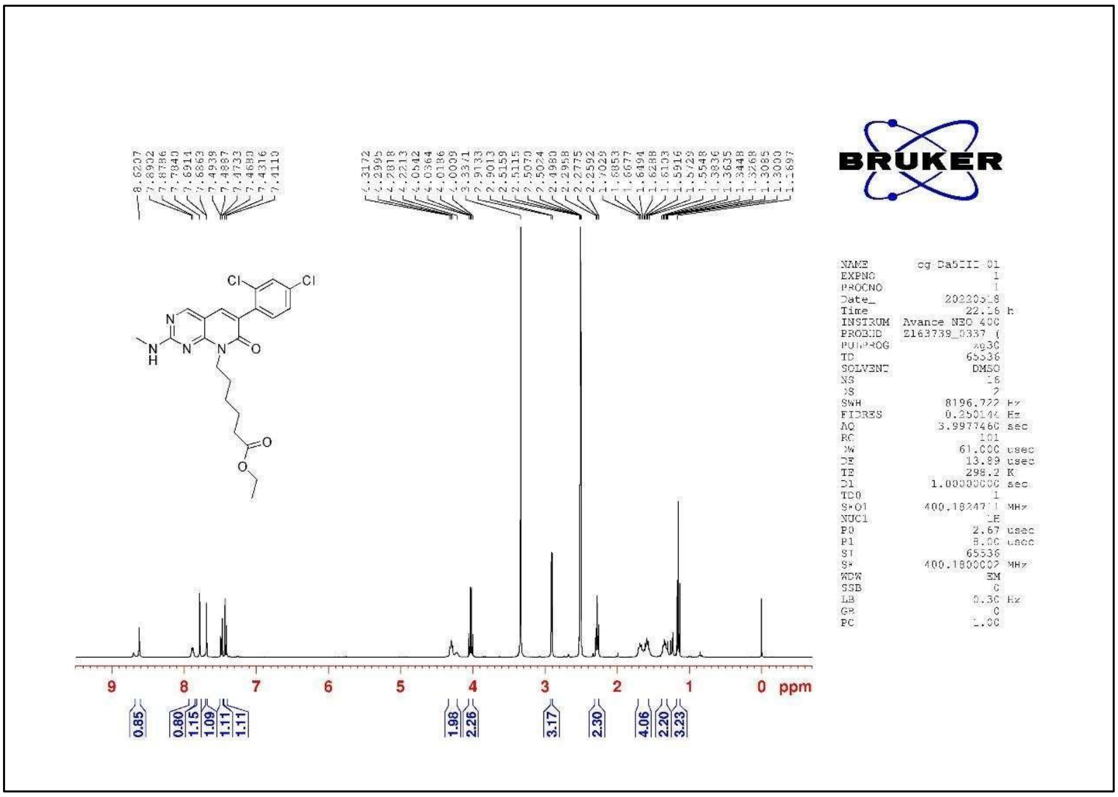

^{1}H-NMR spectrum of compound 17l

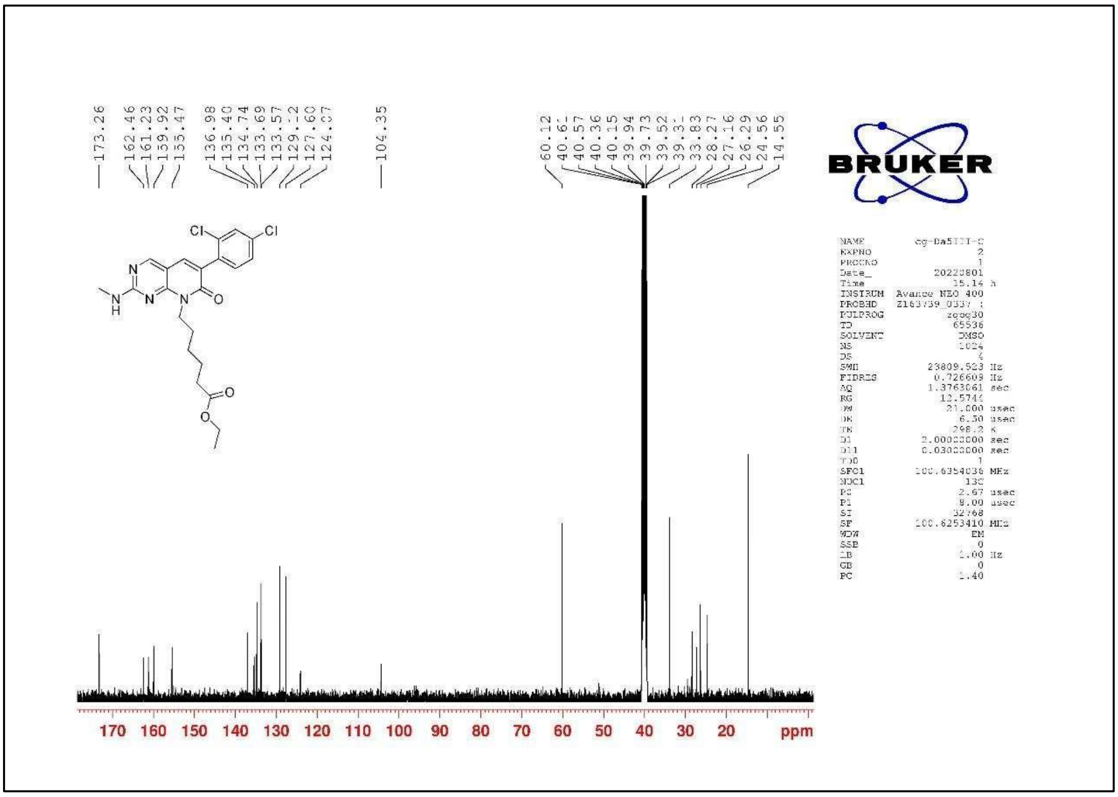

^{13}C-NMR spectrum of compound 17l

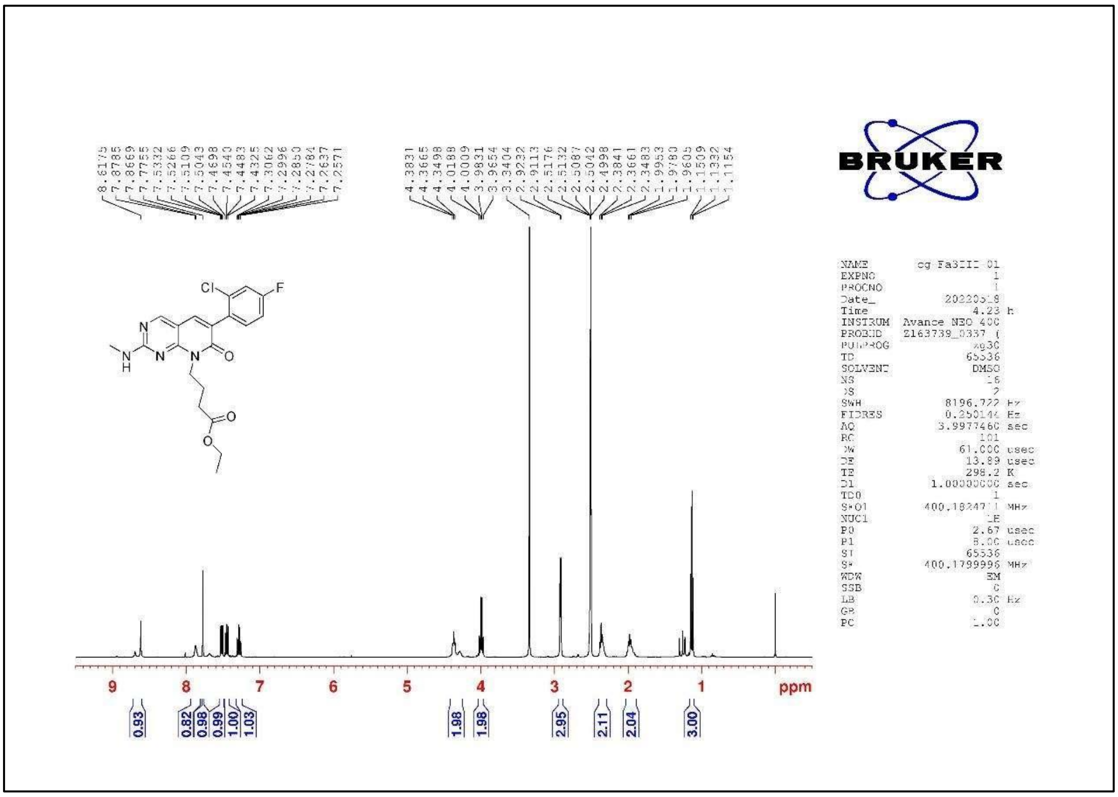

^{1}H-NMR spectrum of compound 17m

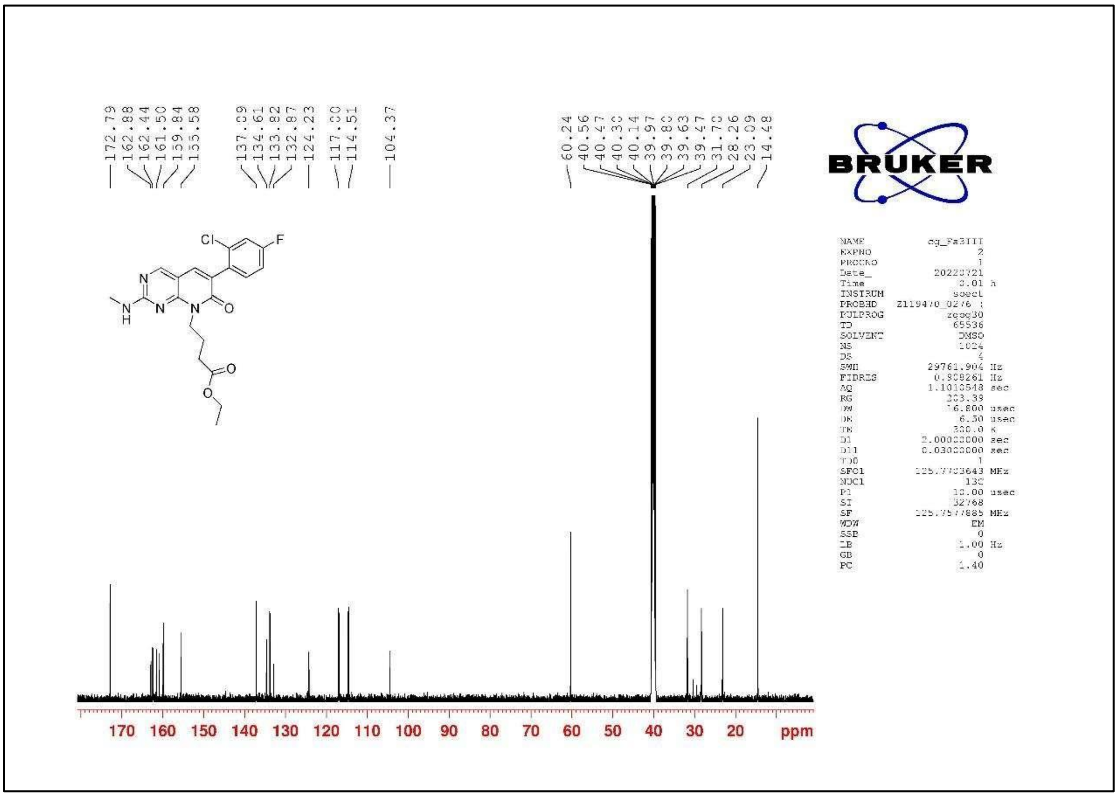

^{13}C-NMR spectrum of compound 17m

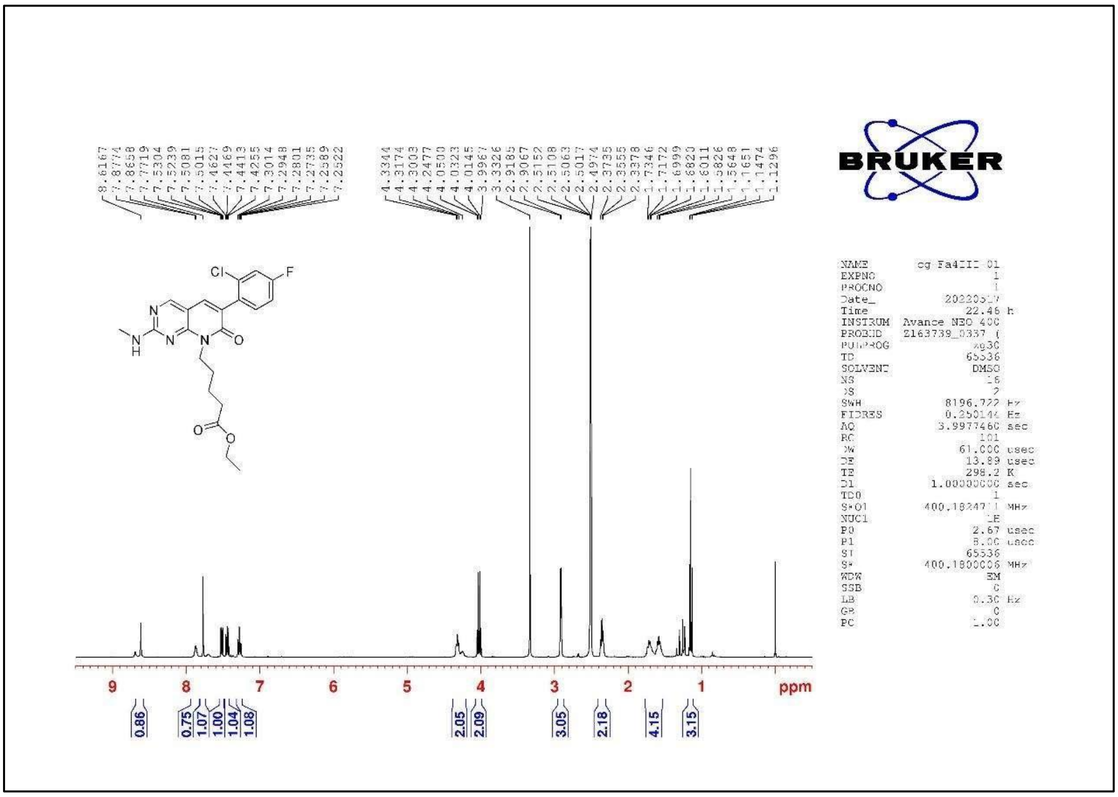

^{1}H-NMR spectrum of compound 17n

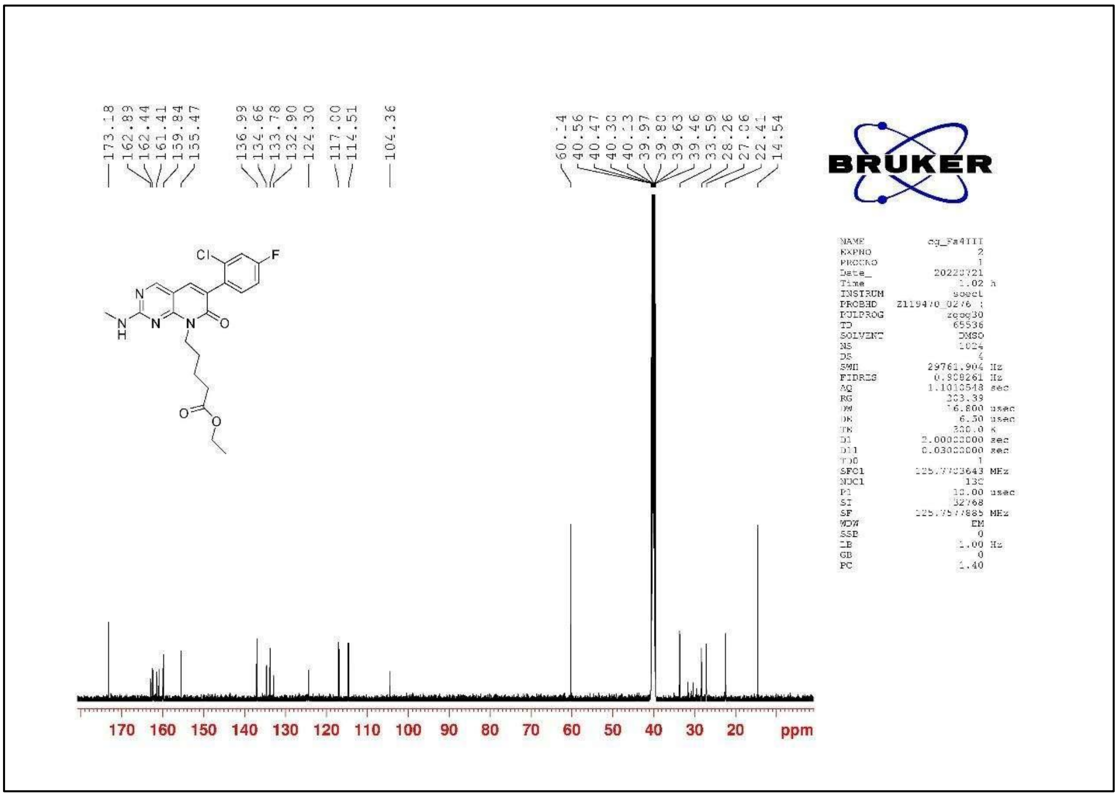

^{13}C-NMR spectrum of compound 17n

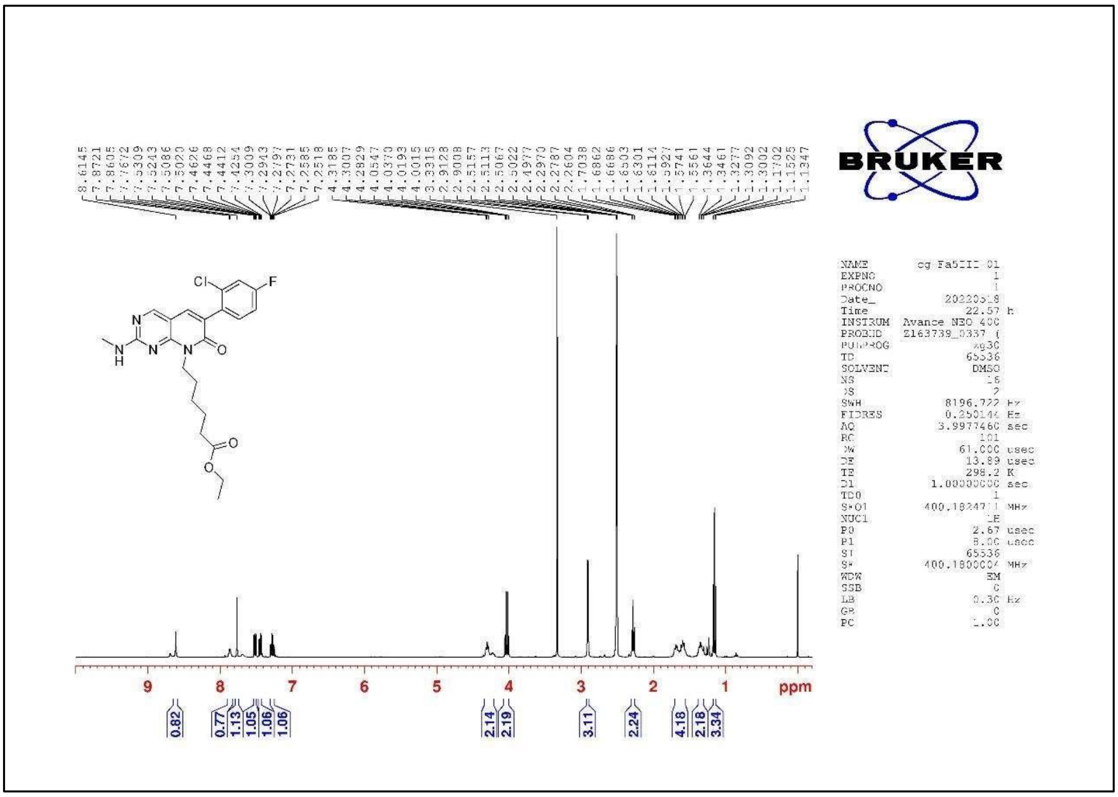

^{1}H-NMR spectrum of compound 17o

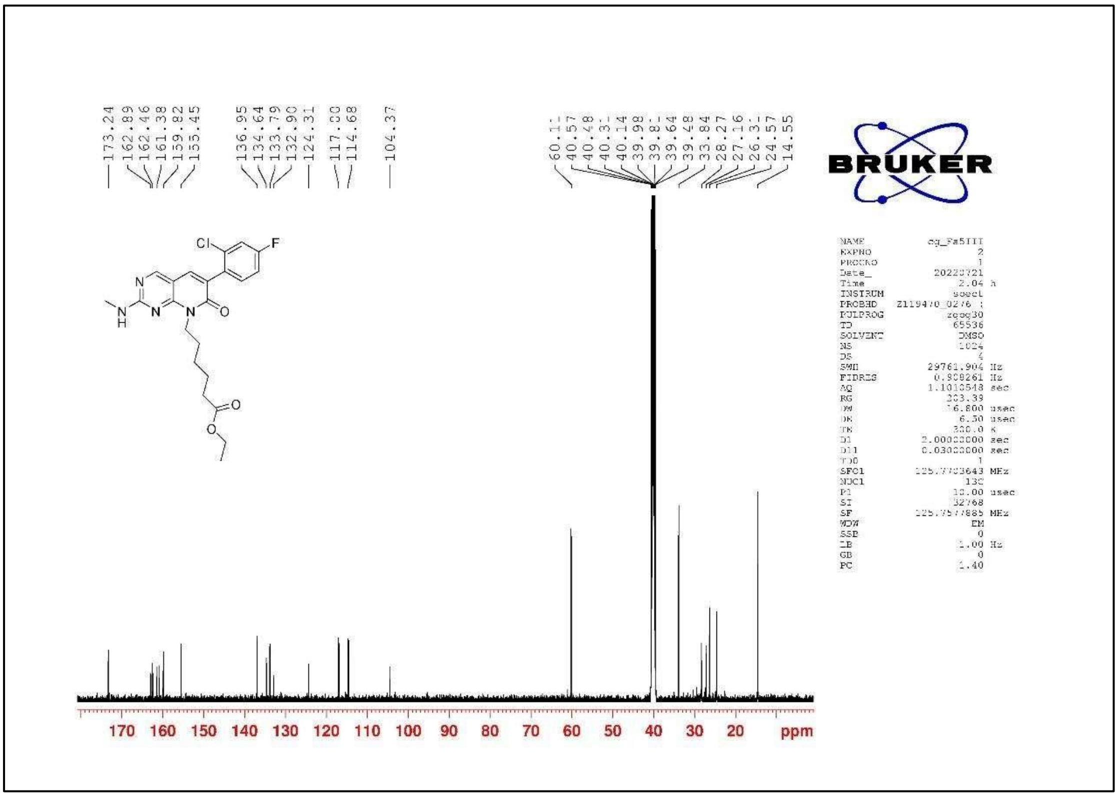

^{13}C-NMR spectrum of compound 17o

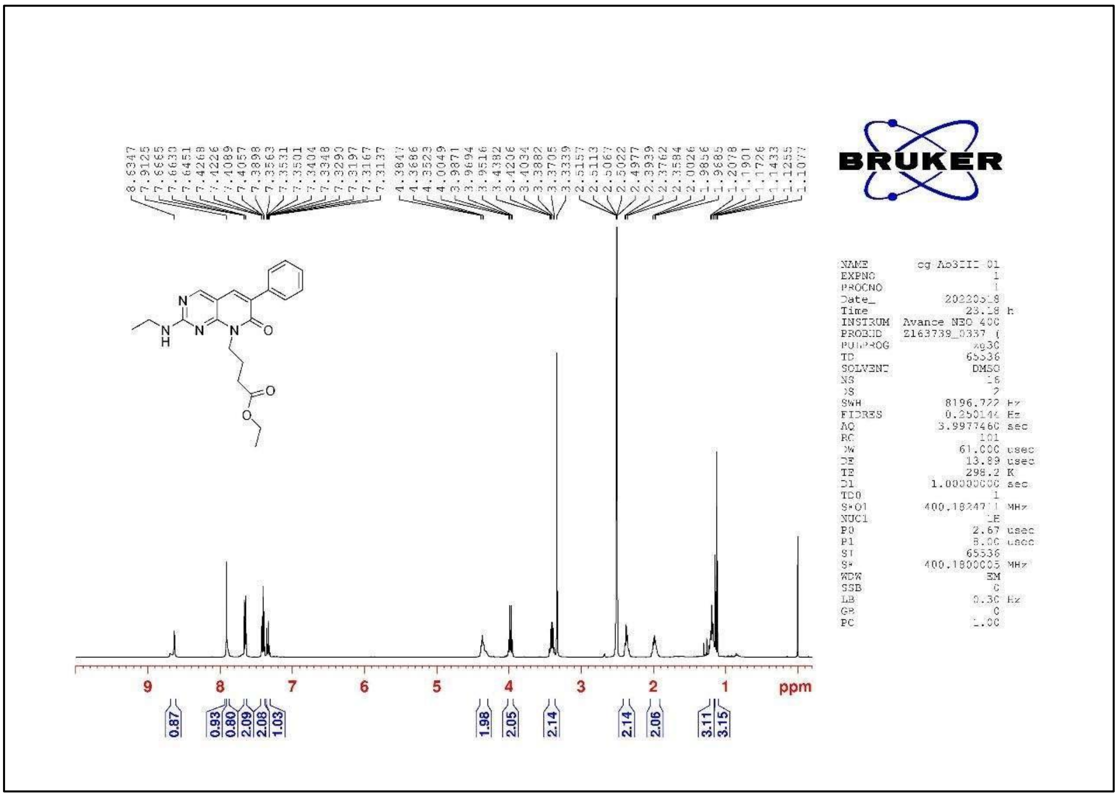

^{1}H-NMR spectrum of compound 18a

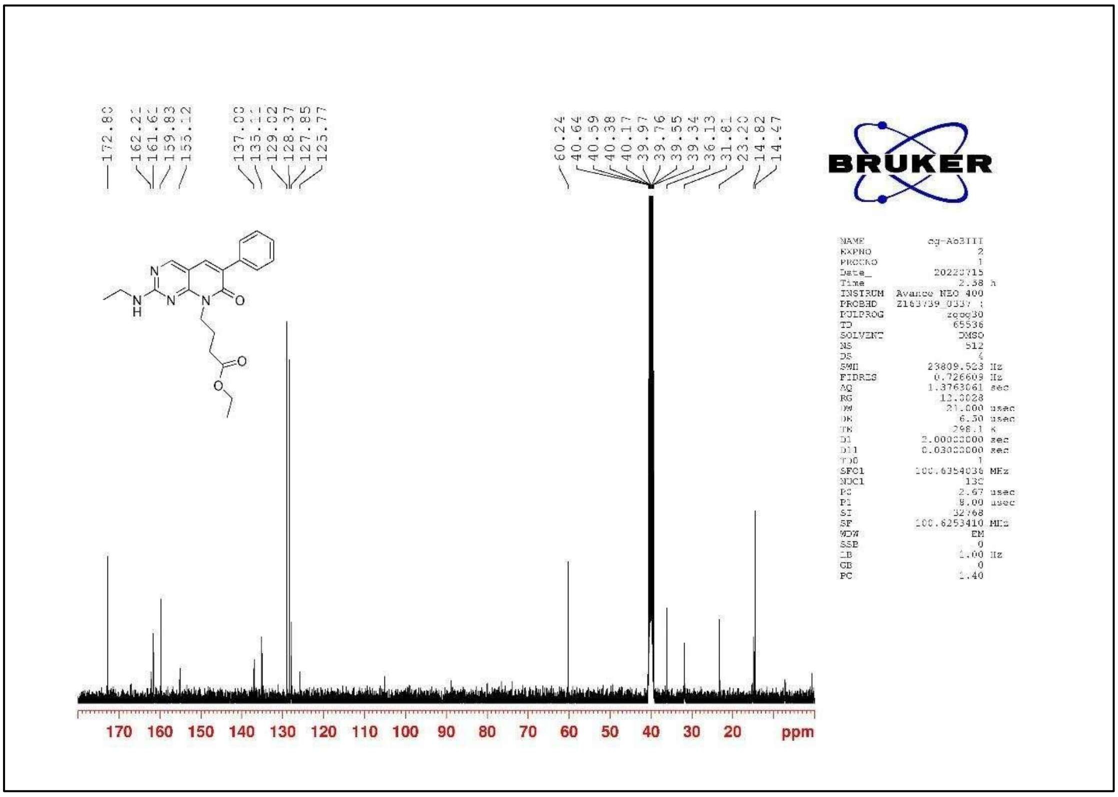

^{13}C-NMR spectrum of compound 18a

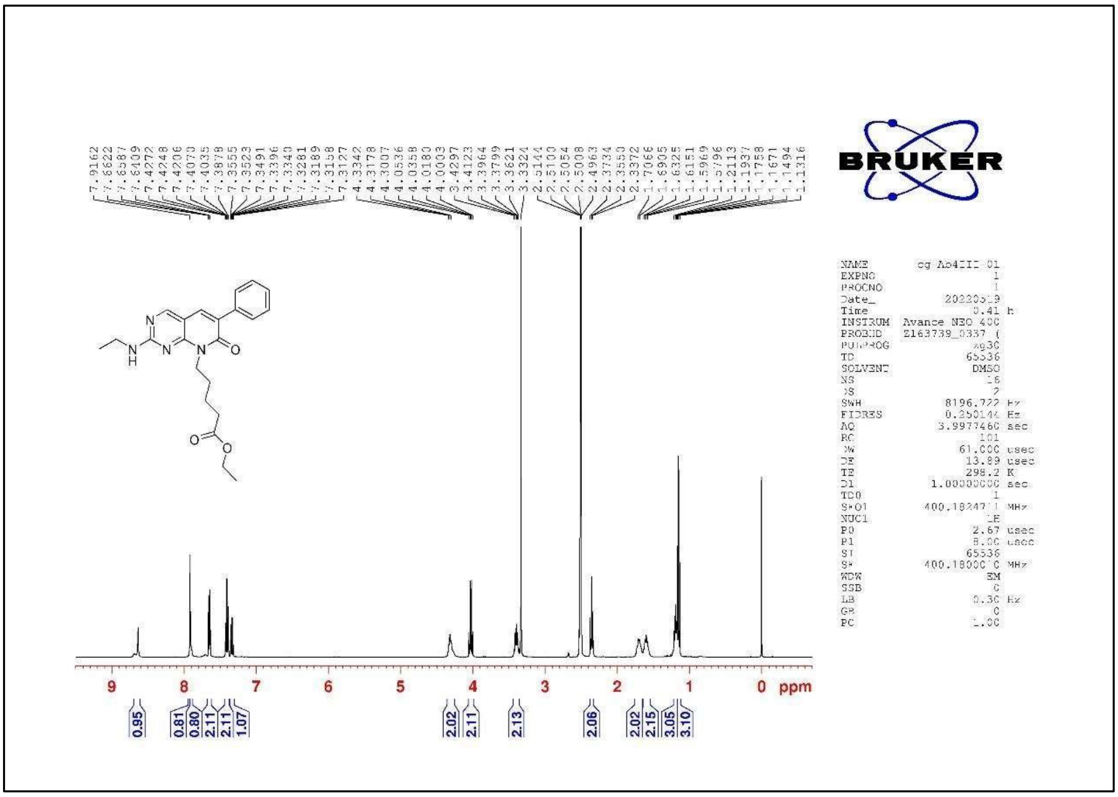

^{1}H-NMR spectrum of compound 18b

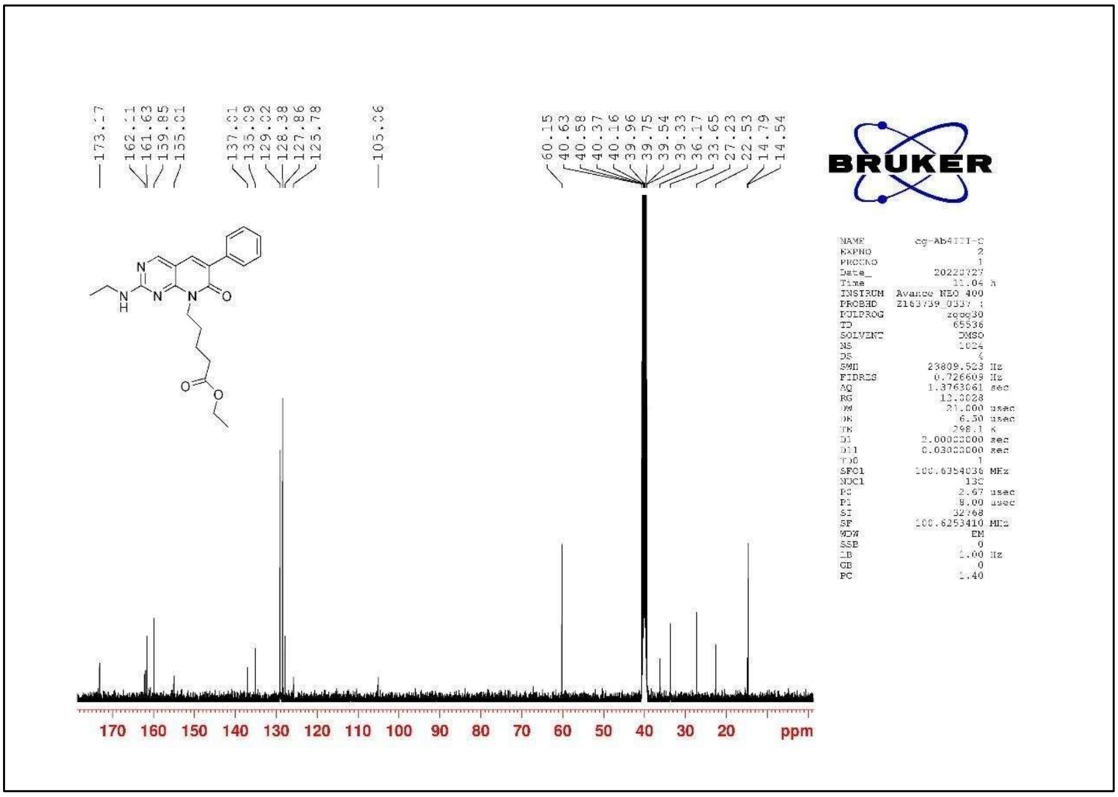

^{13}C-NMR spectrum of compound 18b

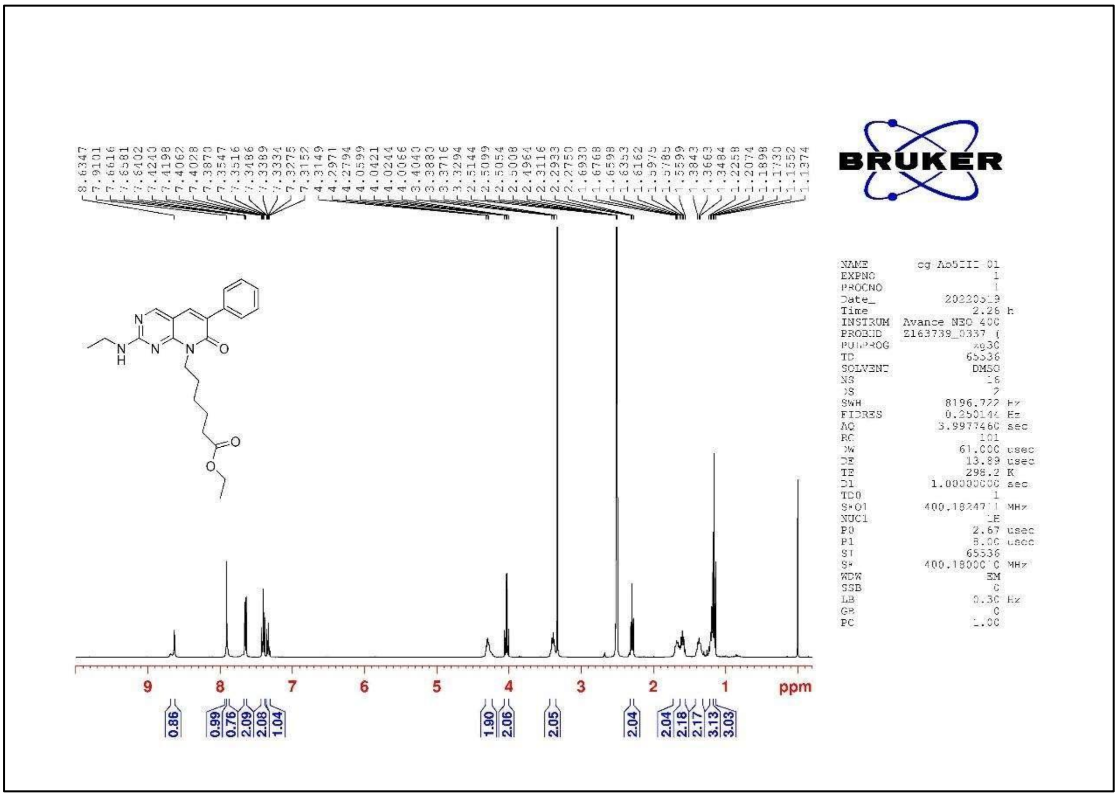

^{1}H-NMR spectrum of compound 18c

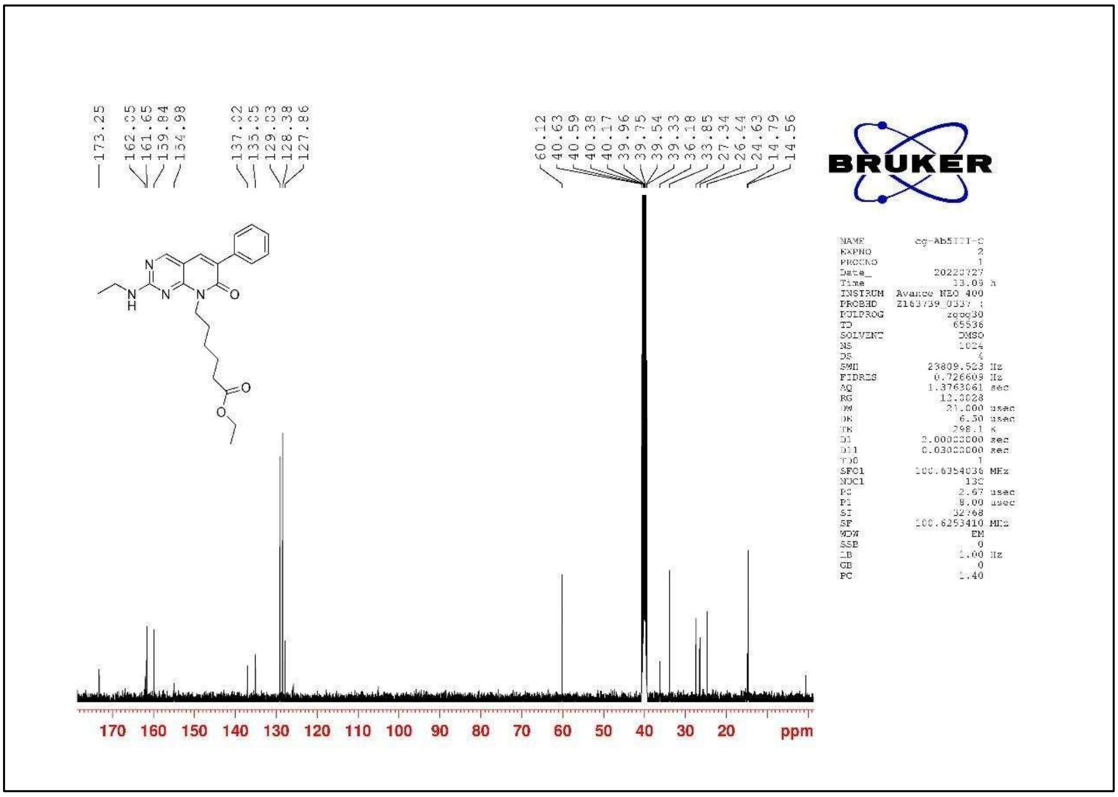

^{13}C-NMR spectrum of compound 18c

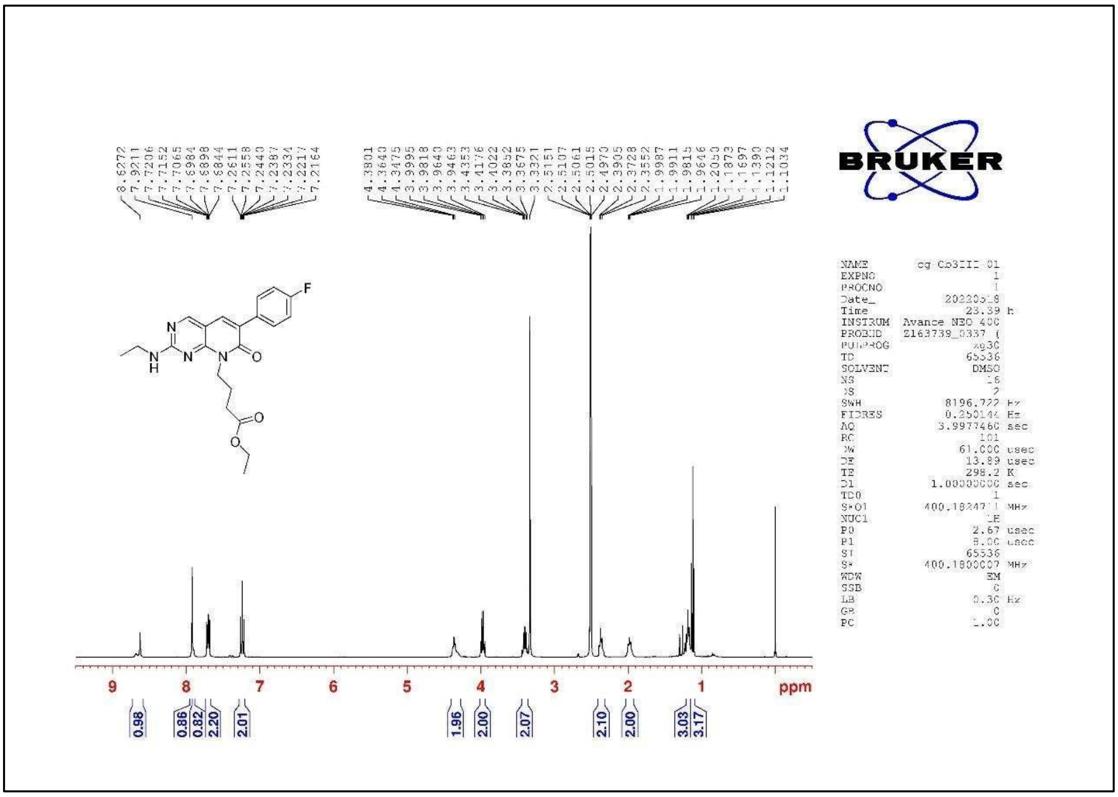

^{1}H-NMR spectrum of compound 18d

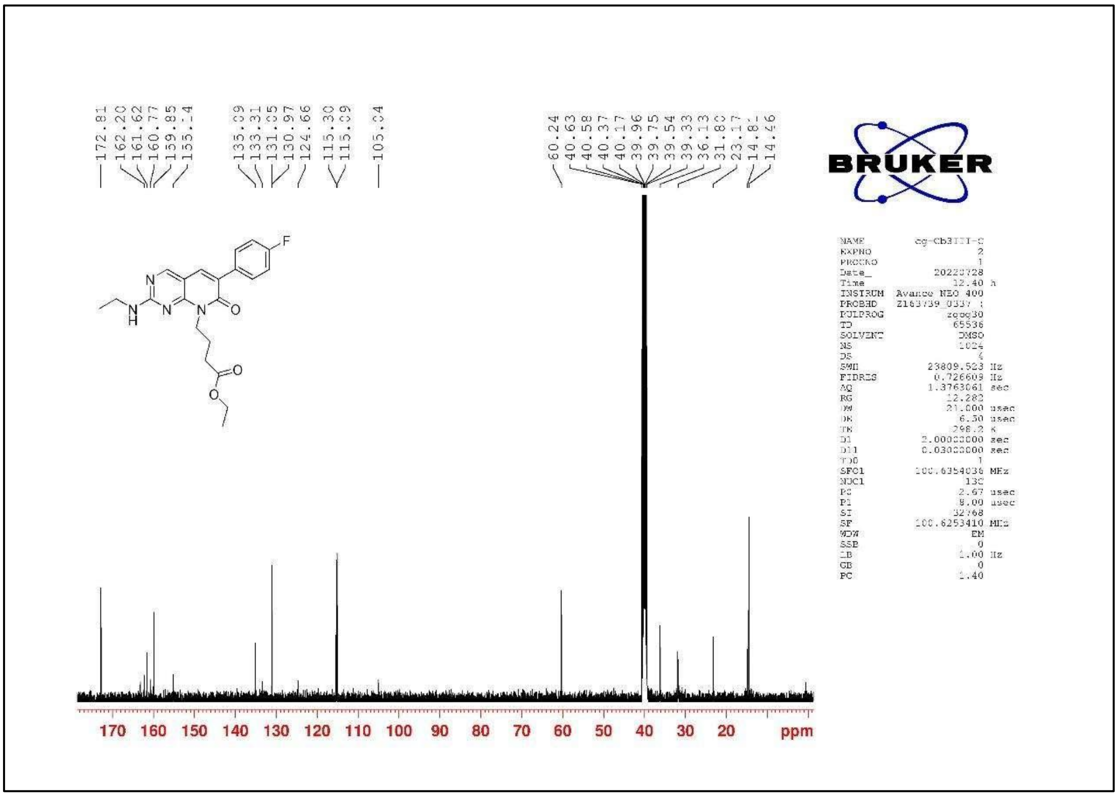

^{13}C-NMR spectrum of compound 18d

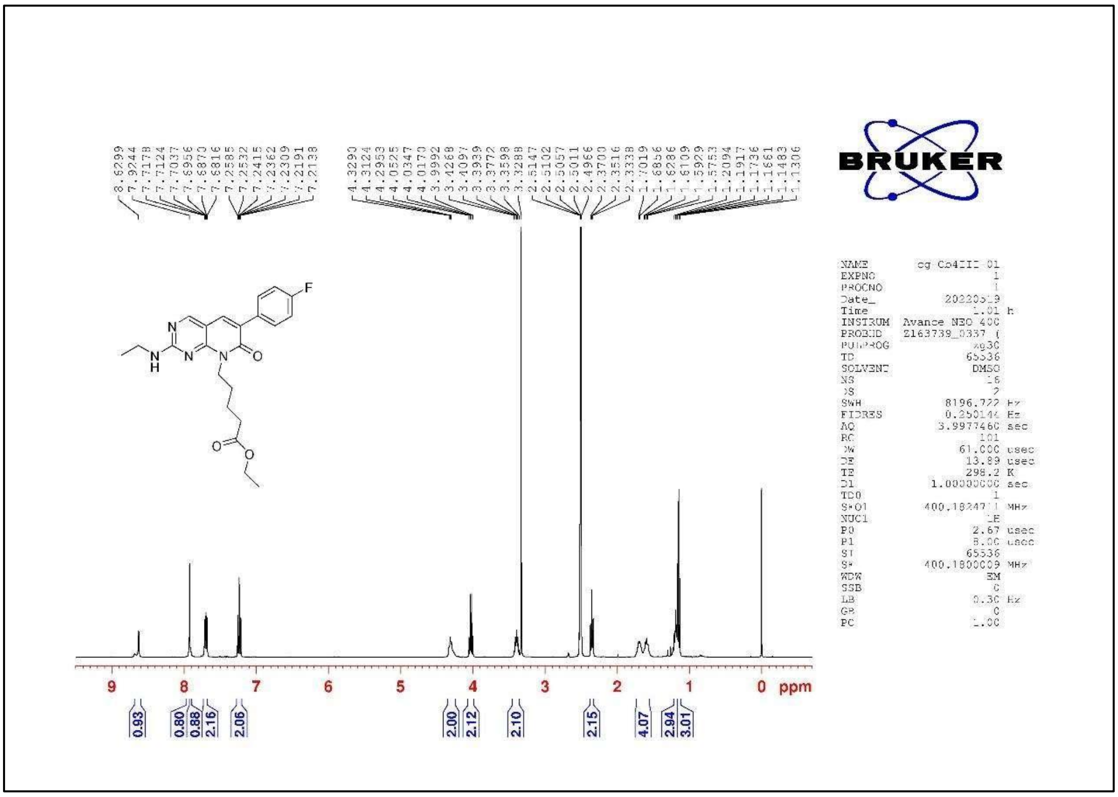

^{1}H-NMR spectrum of compound 18e

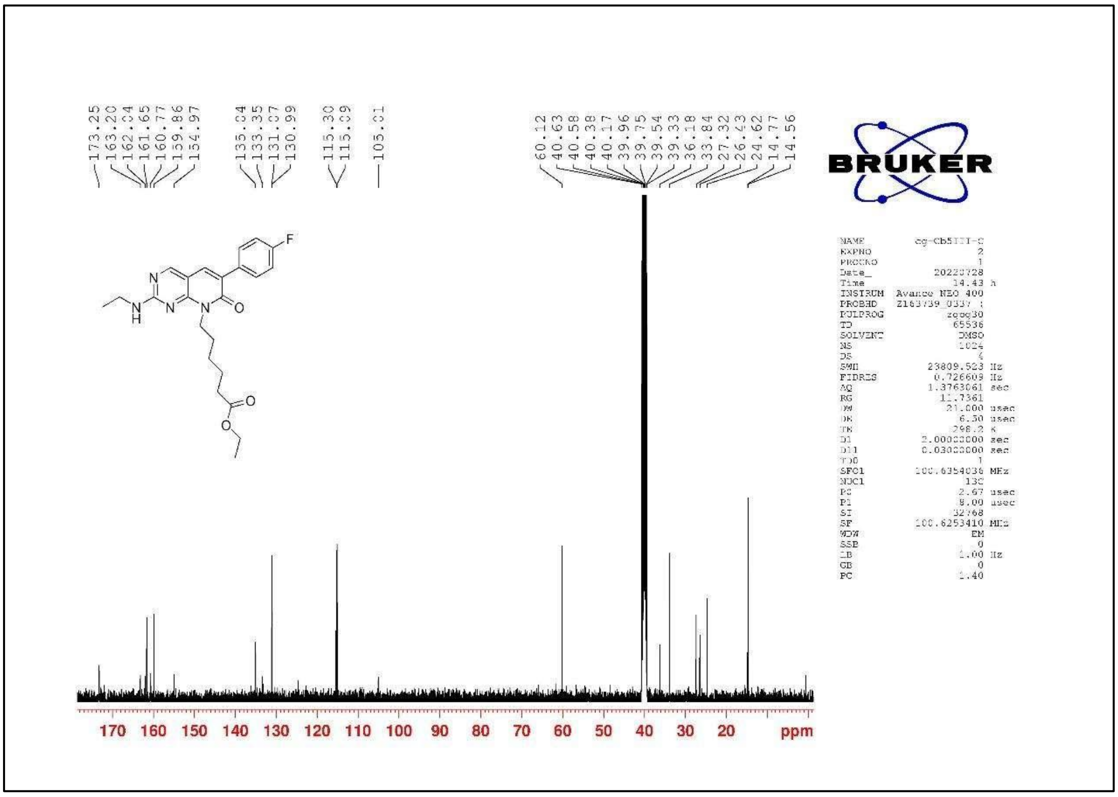

^{13}C-NMR spectrum of compound 18e

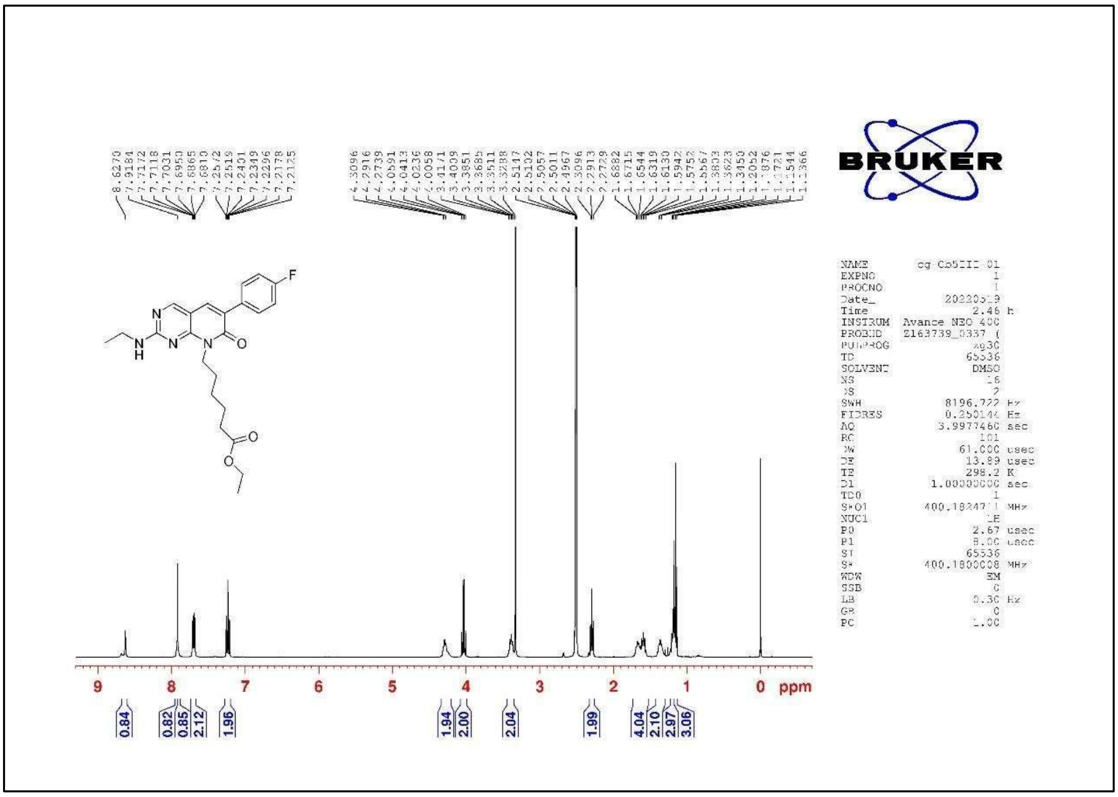

^{1}H-NMR spectrum of compound 18f

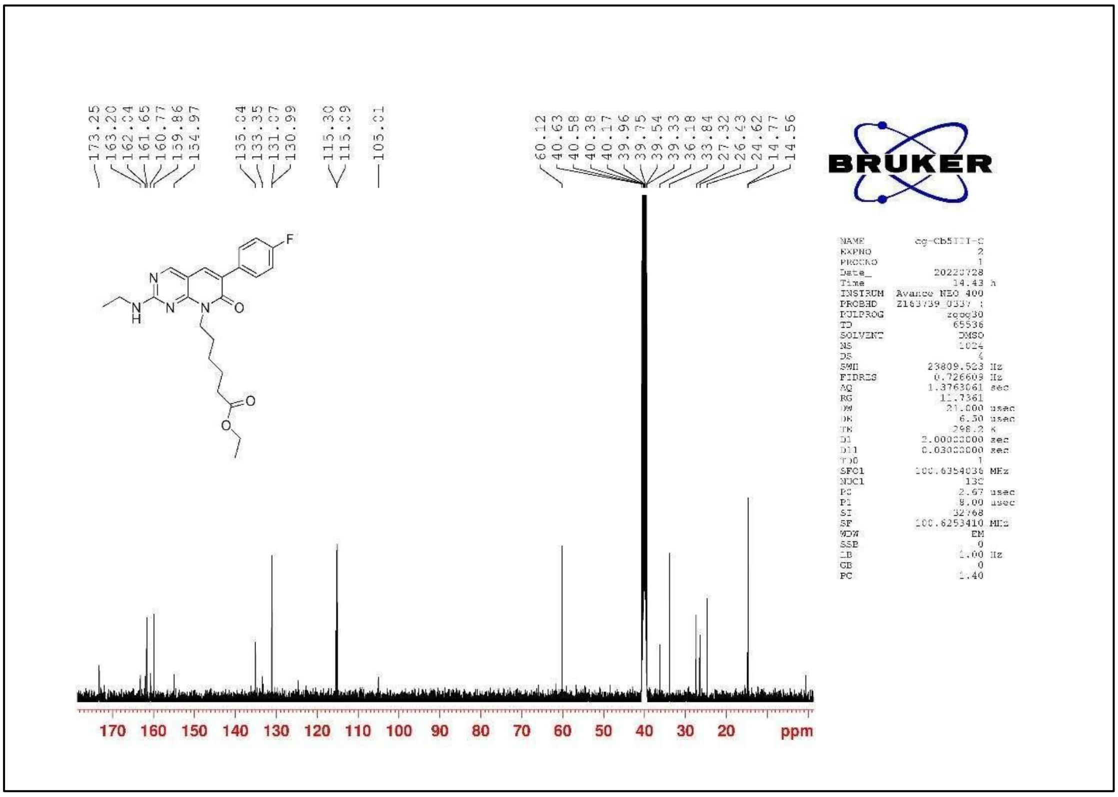

^{13}C-NMR spectrum of compound 18f

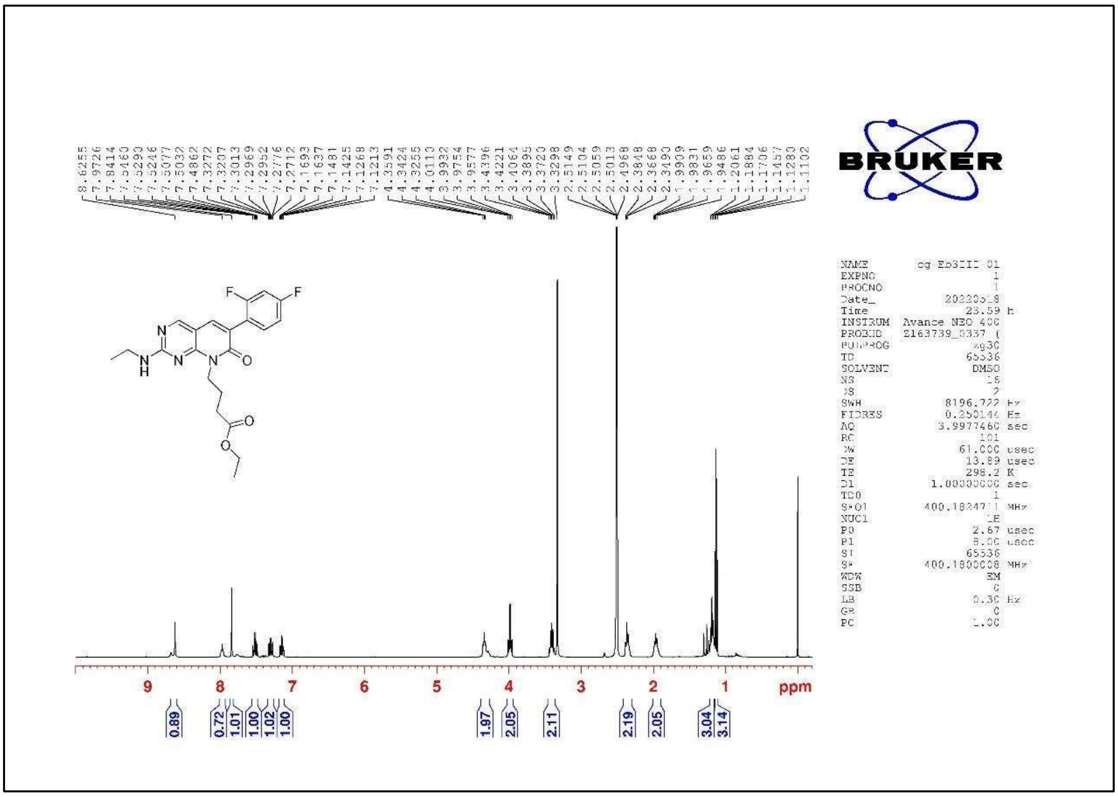

^{1}H-NMR spectrum of compound 18g

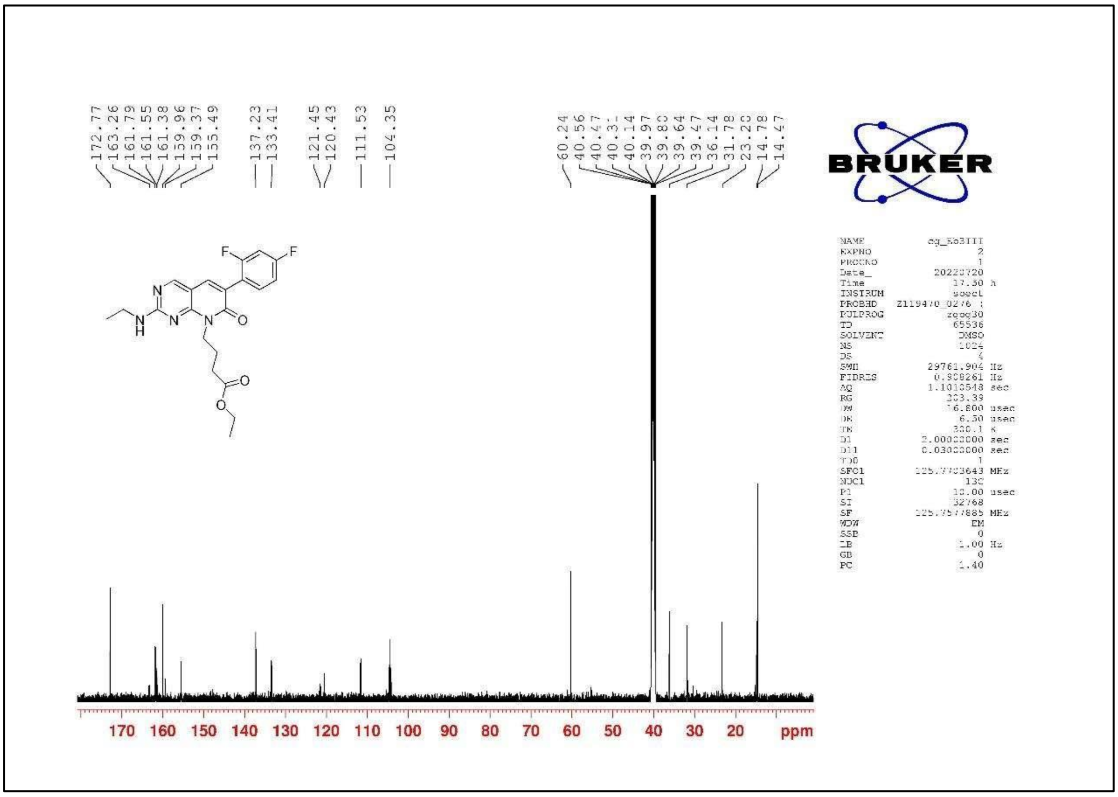

^{13}C-NMR spectrum of compound 18g

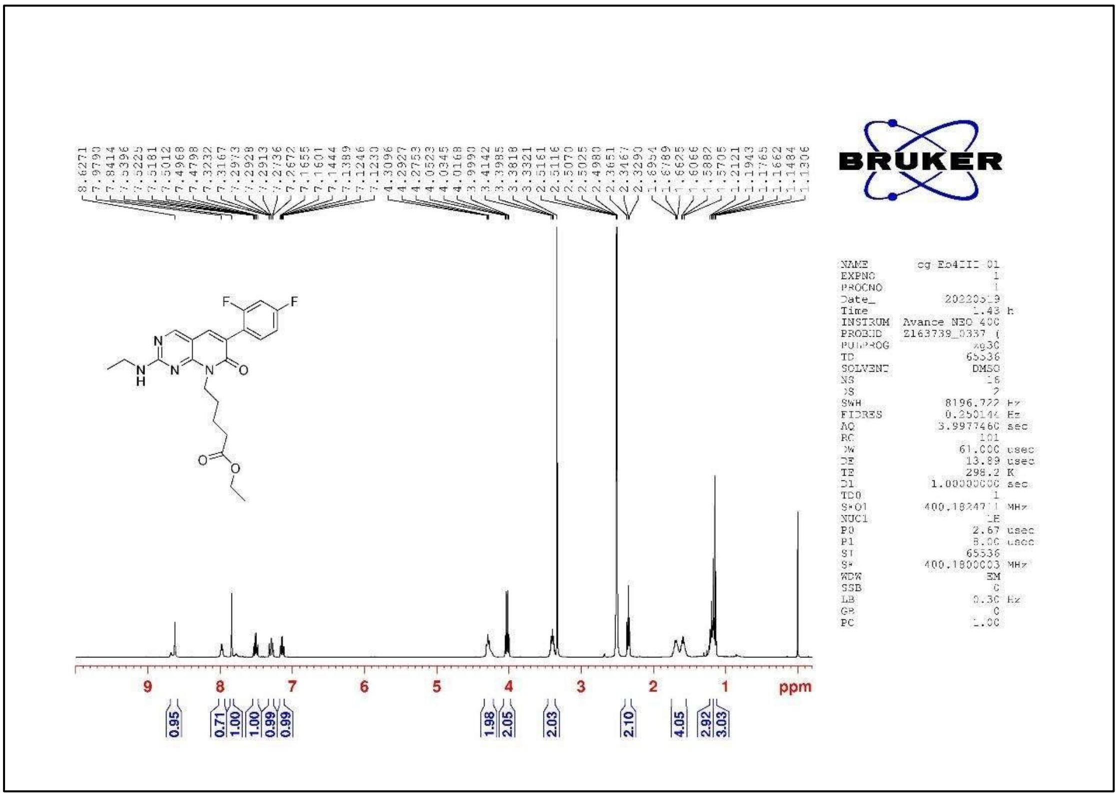

^{1}H-NMR spectrum of compound 18h

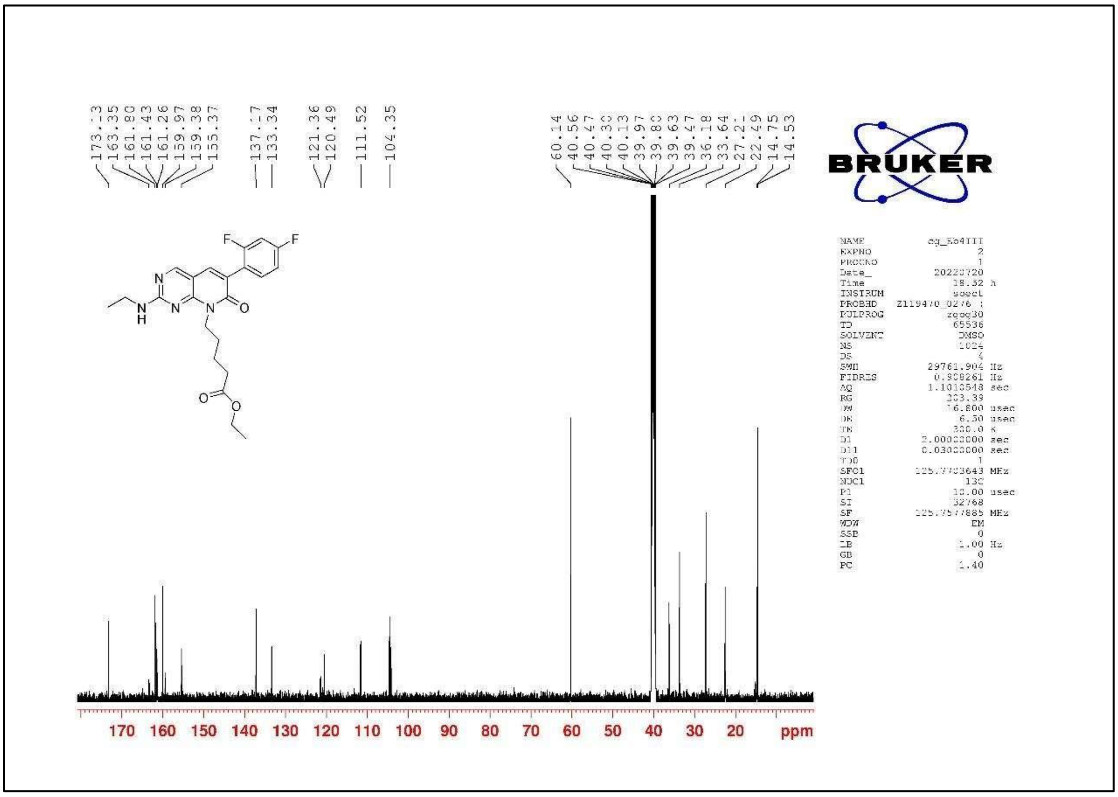

^{13}C-NMR spectrum of compound 18h

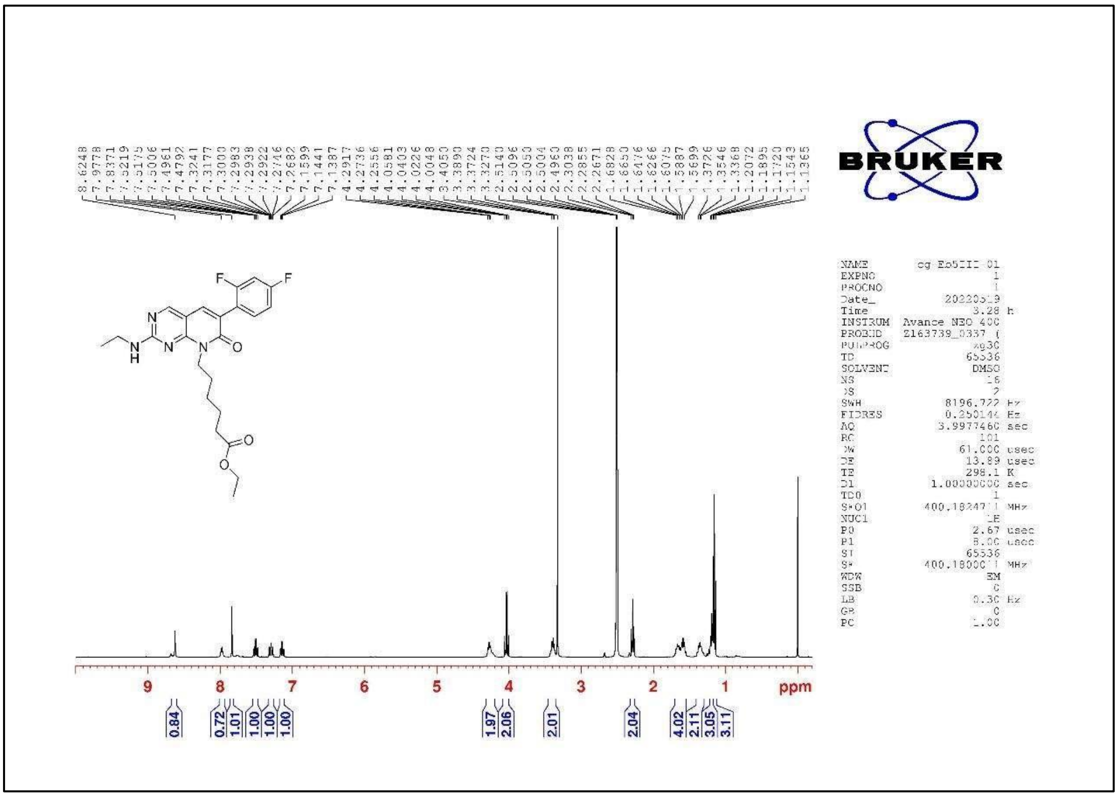

^{1}H-NMR spectrum of compound 18i

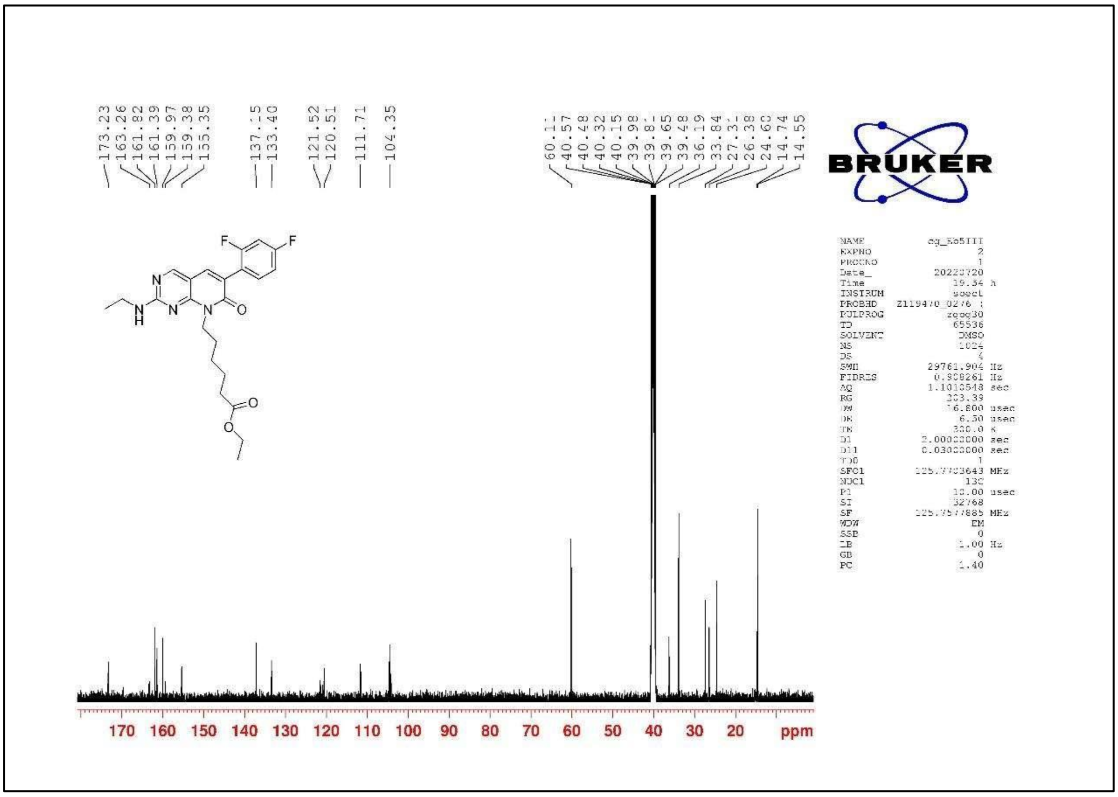

^{13}C-NMR spectrum of compound 18i

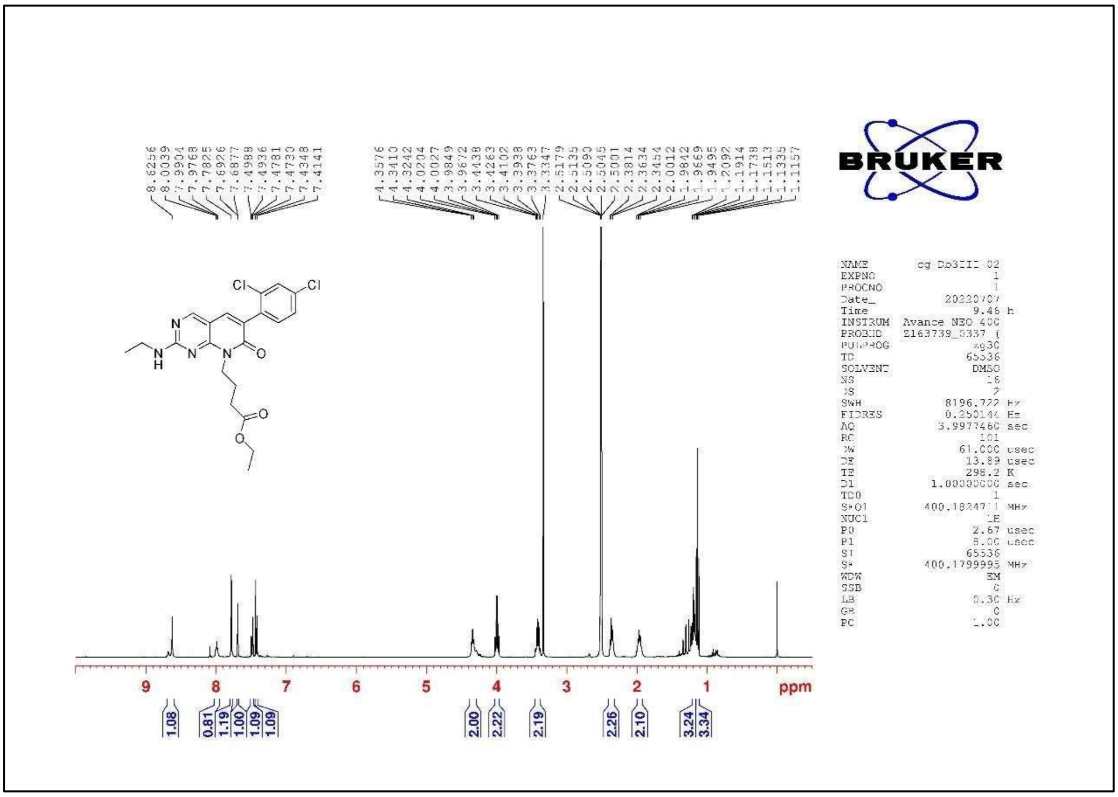

^{1}H-NMR spectrum of compound 18j

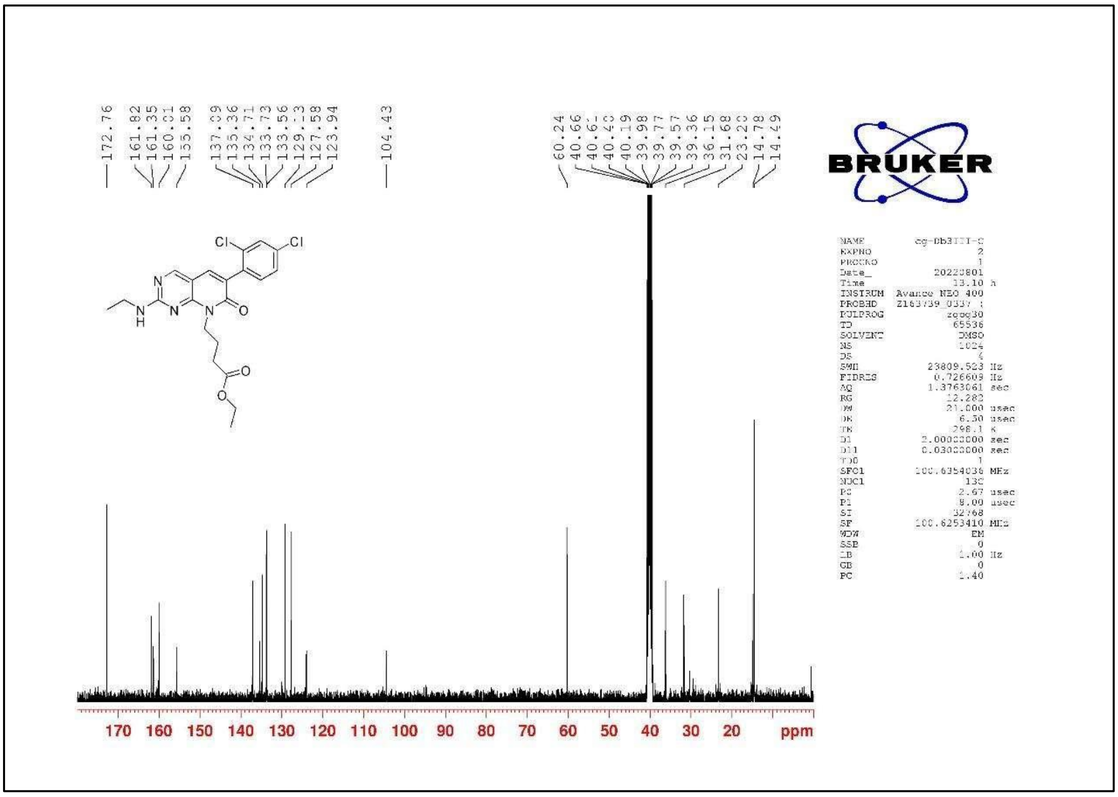

^{13}C-NMR spectrum of compound 18j

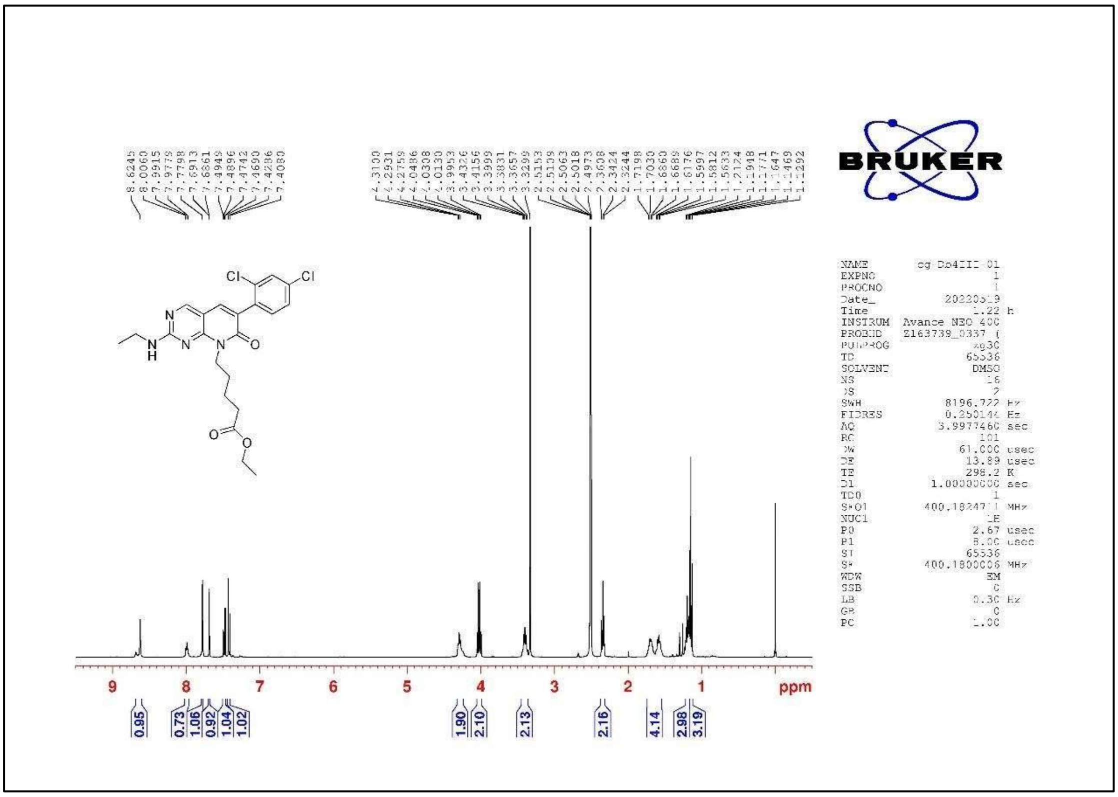

^{1}H-NMR spectrum of compound 18k

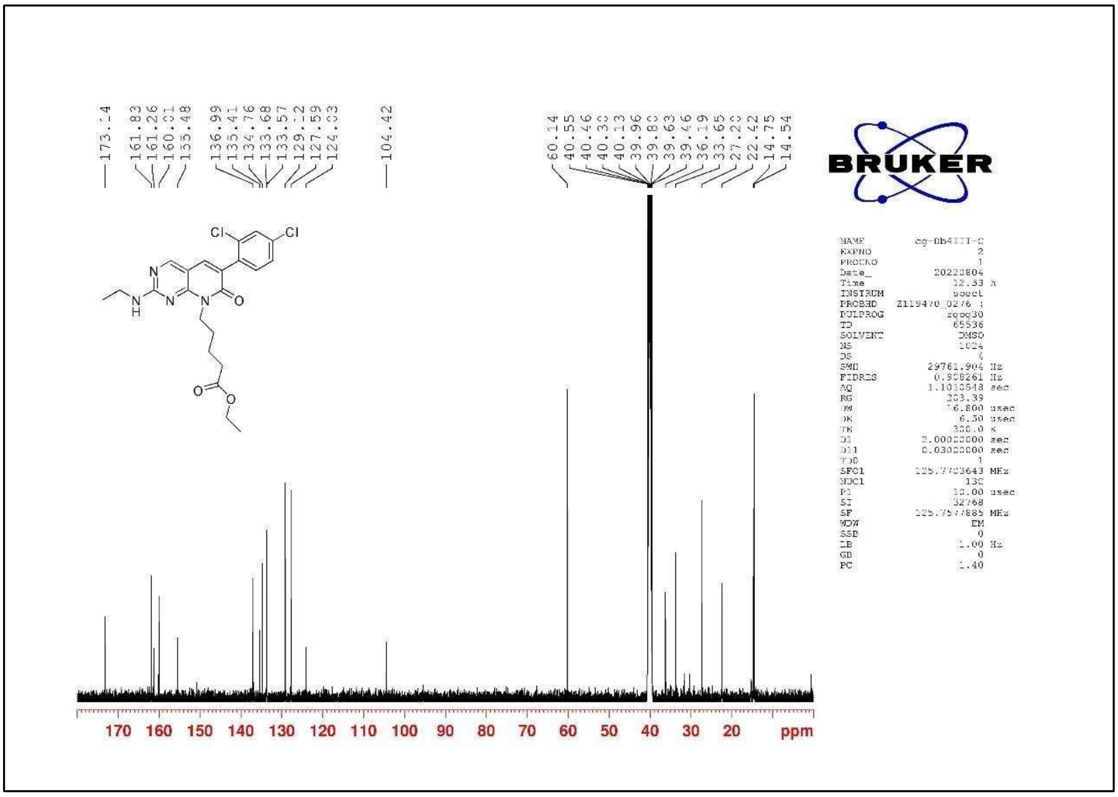

^{13}C-NMR spectrum of compound 18k

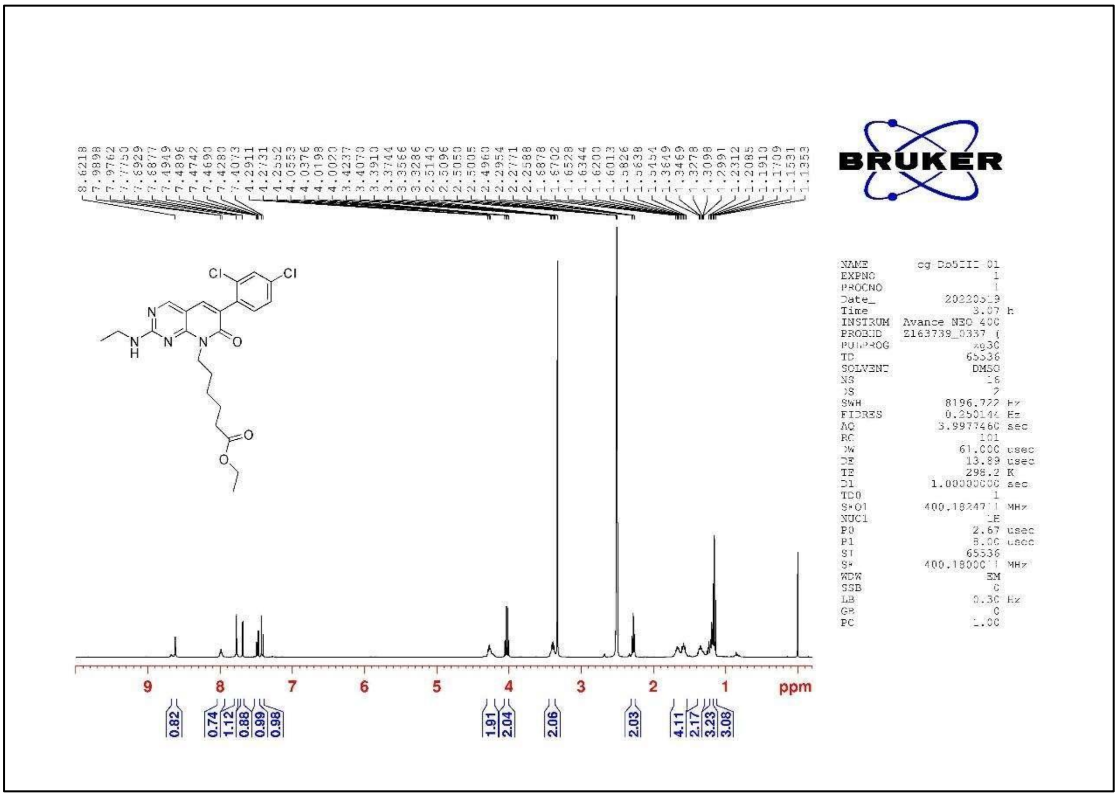

^{1}H-NMR spectrum of compound 18l

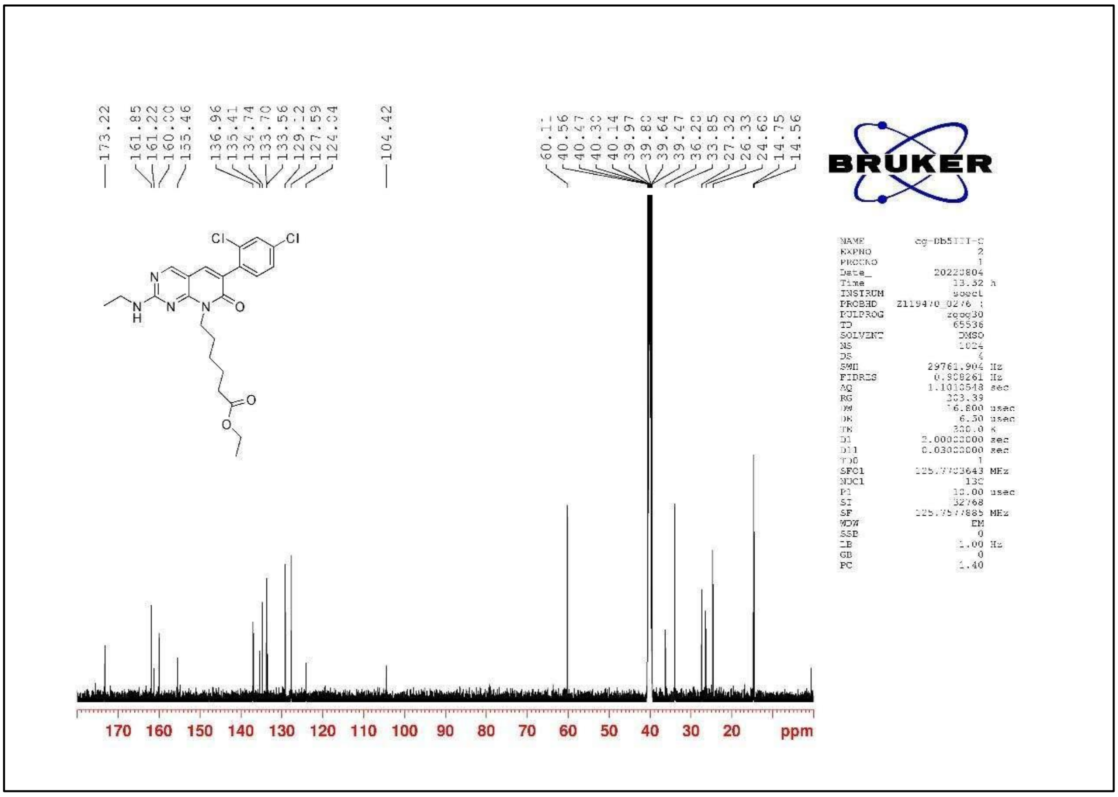

^{13}C-NMR spectrum of compound 18l

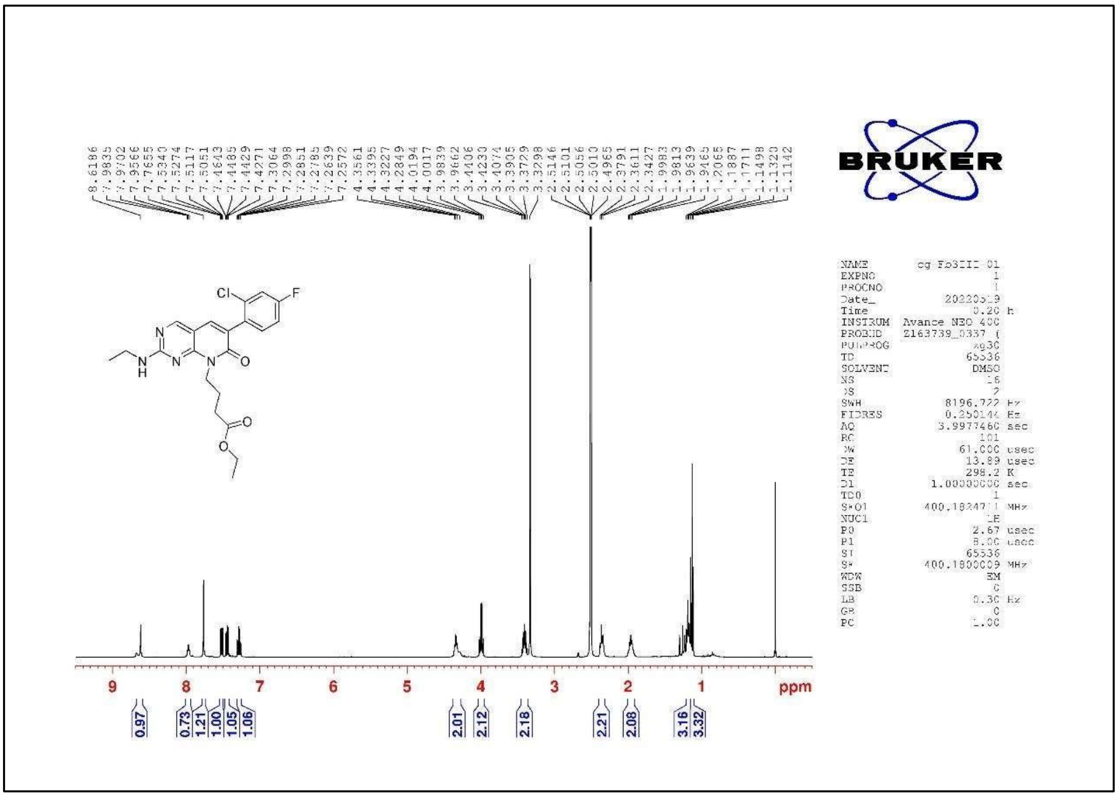

^{1}H-NMR spectrum of compound 18m

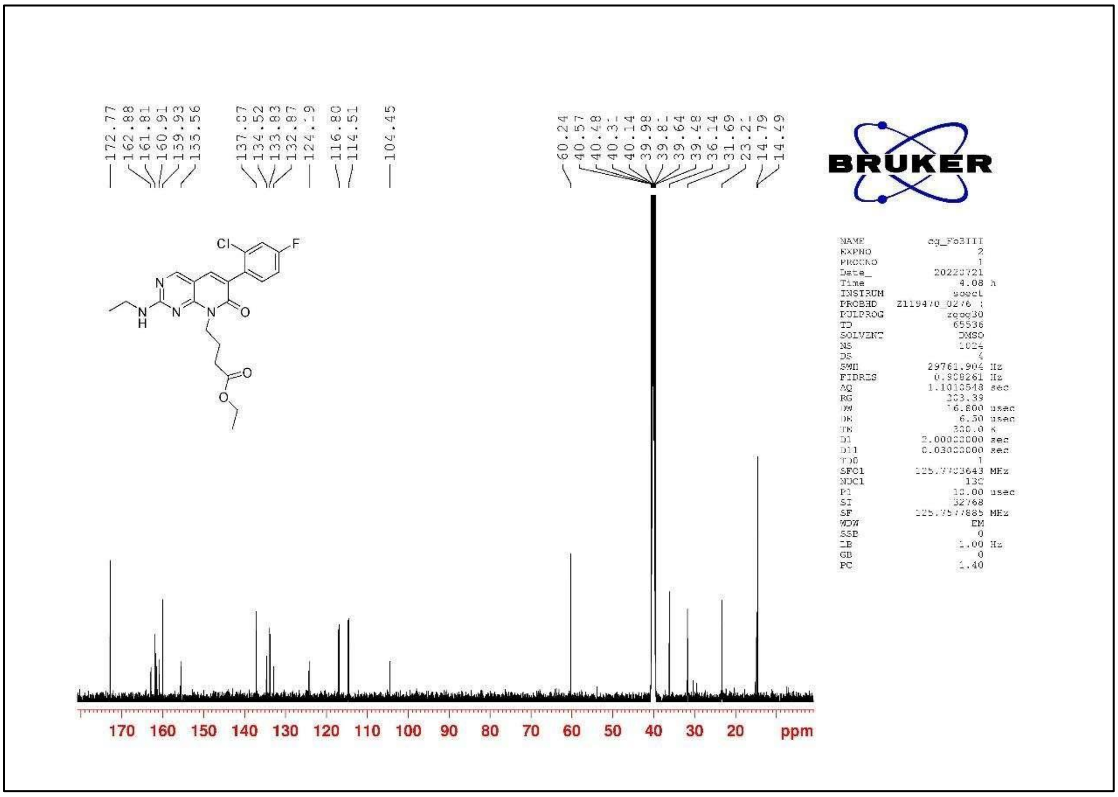

^{13}C-NMR spectrum of compound 18m

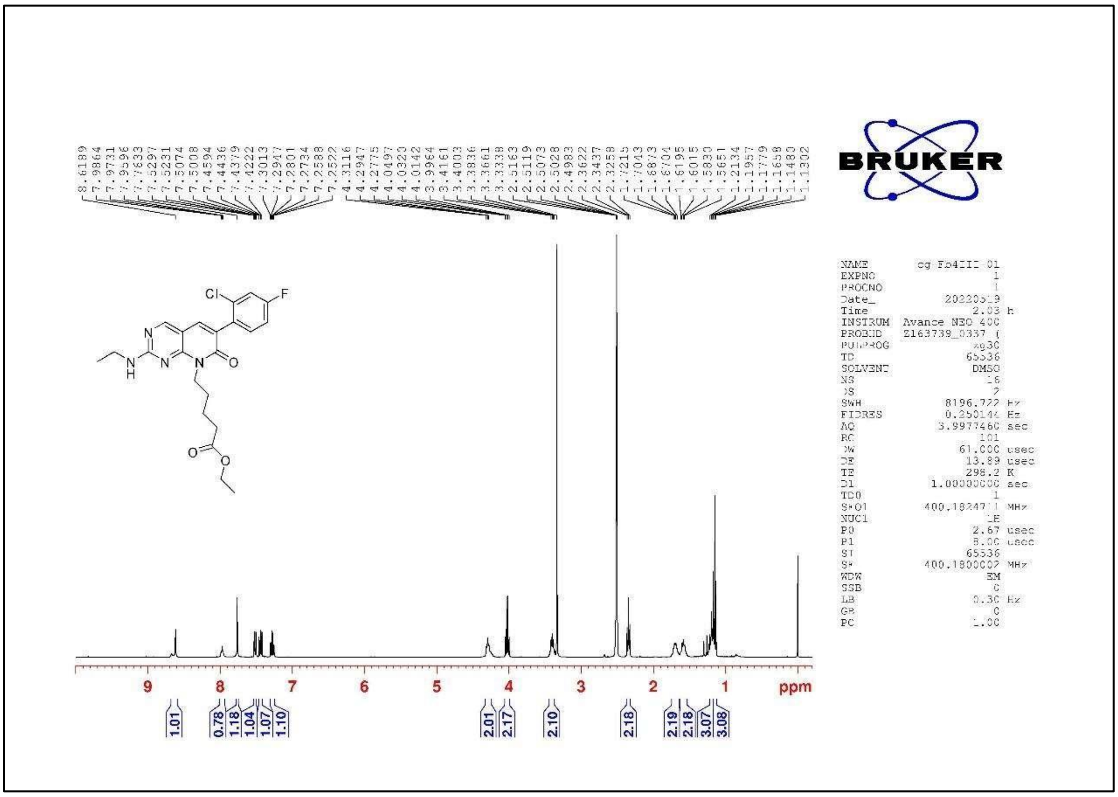

^{1}**H-NMR spectrum of compound 18n**

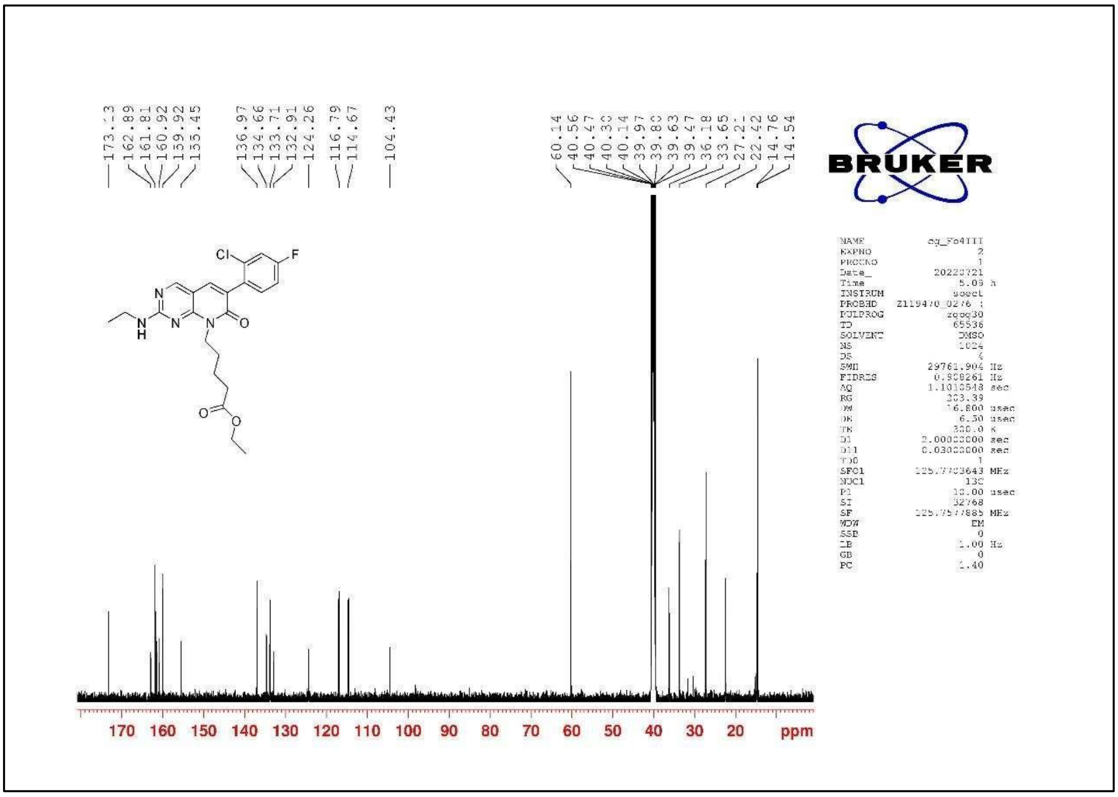

^{13}**C-NMR spectrum of compound 18n**

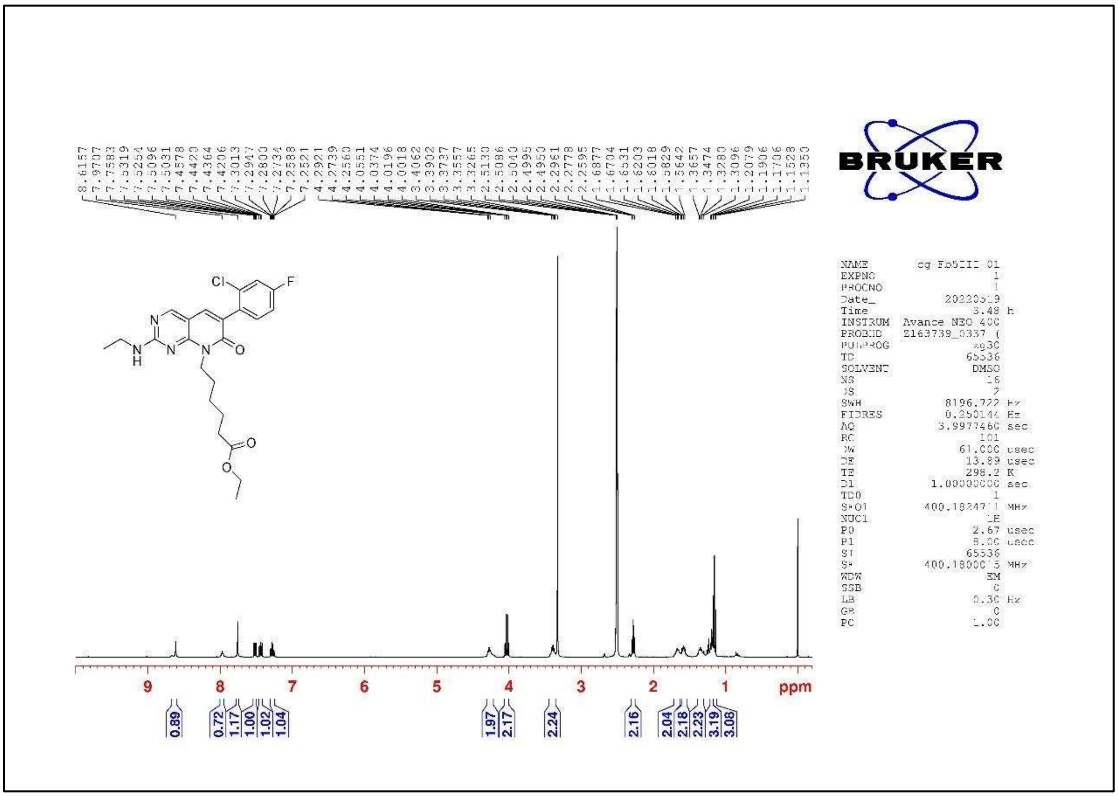

^{1}H-NMR spectrum of compound 18o

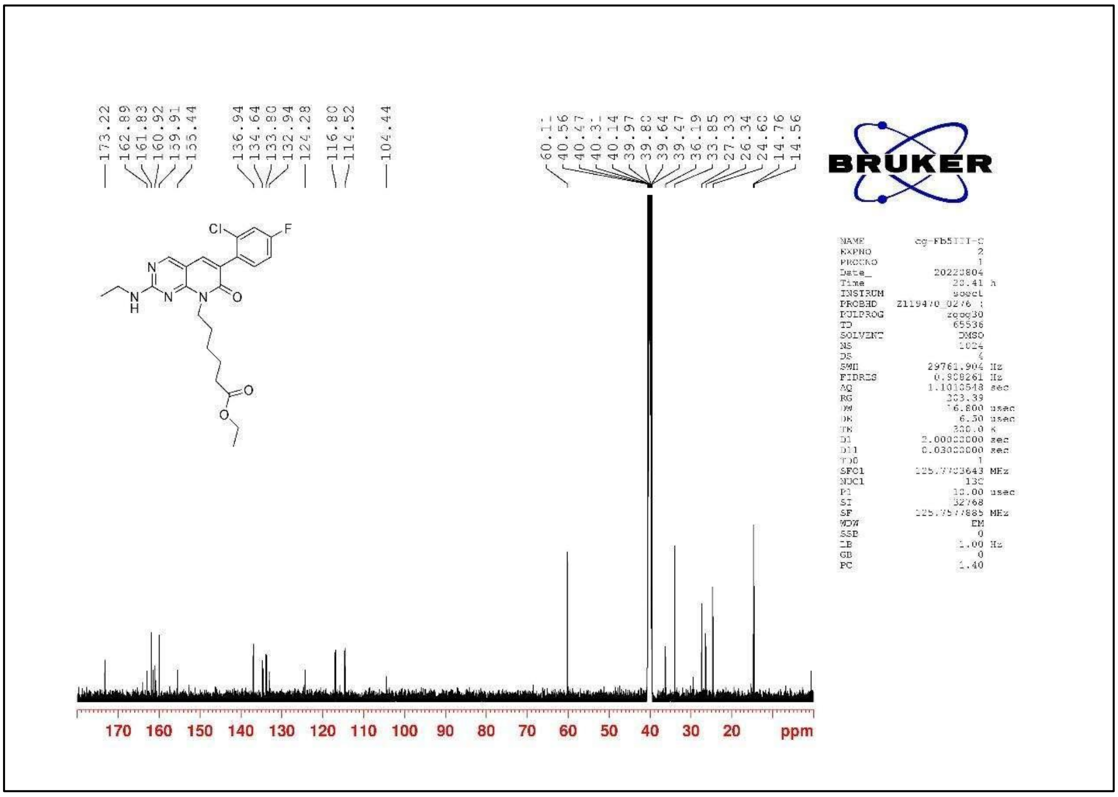

^{13}C-NMR spectrum of compound 18o

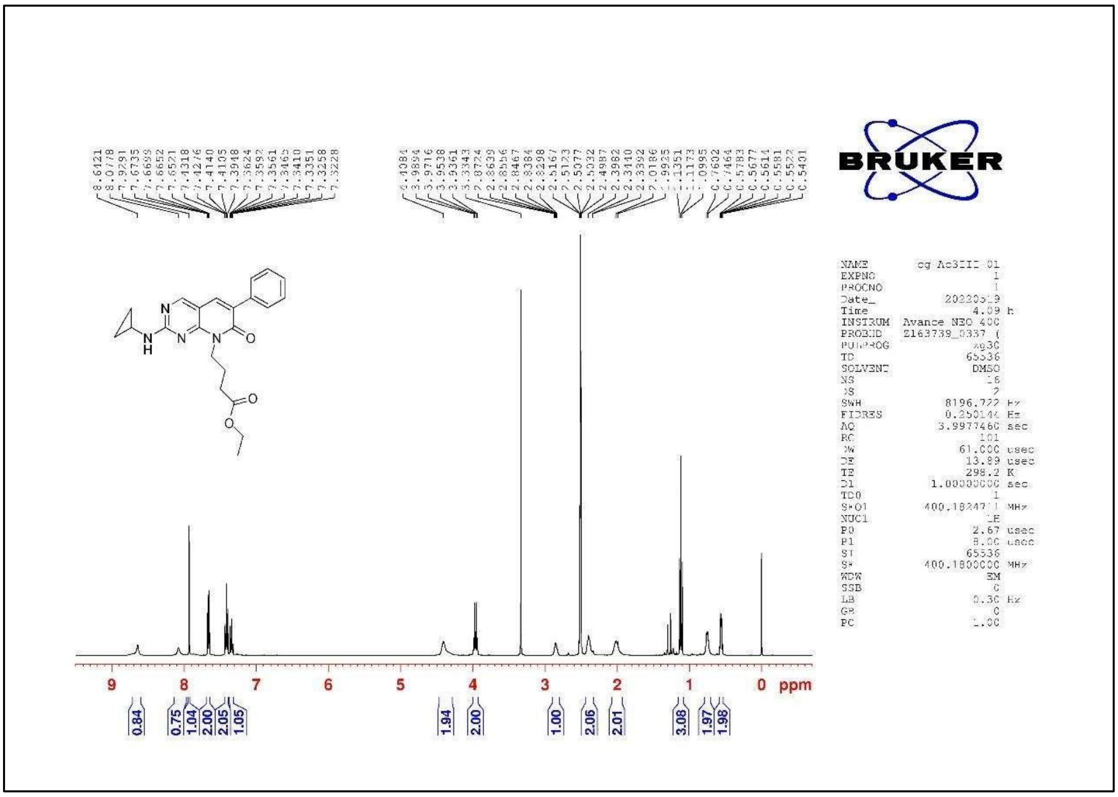

^{1}H-NMR spectrum of compound 19a

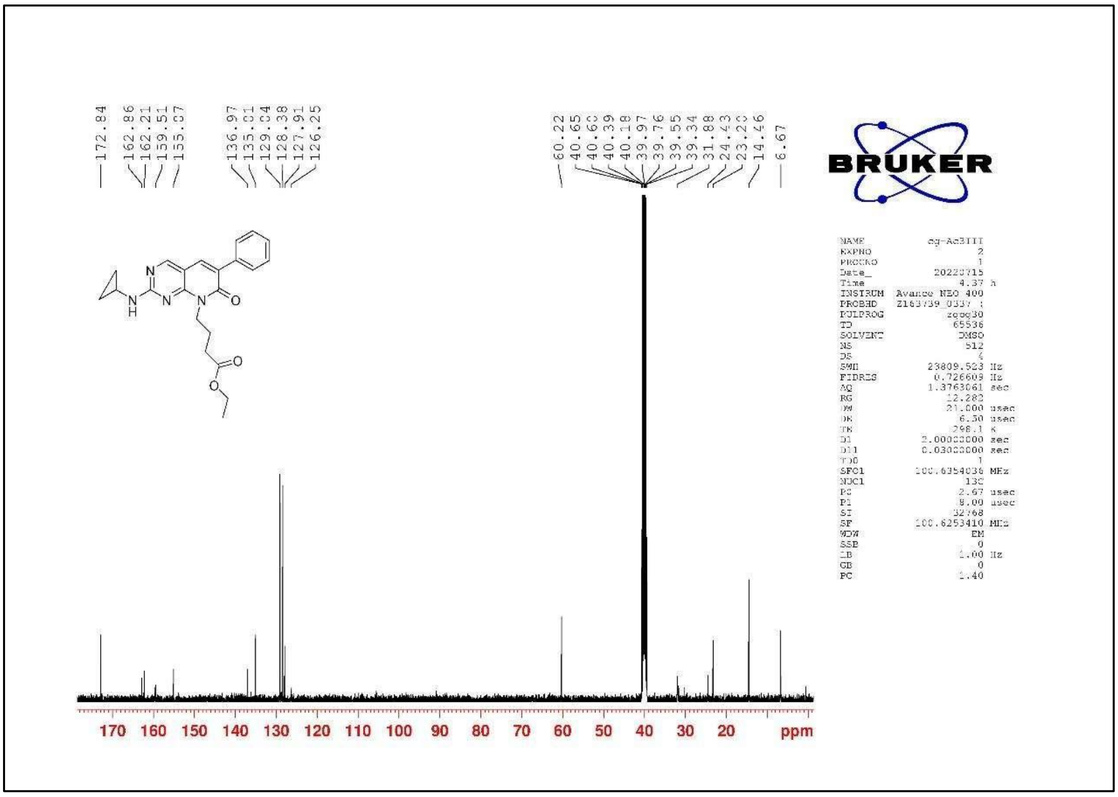

^{13}C-NMR spectrum of compound 19a

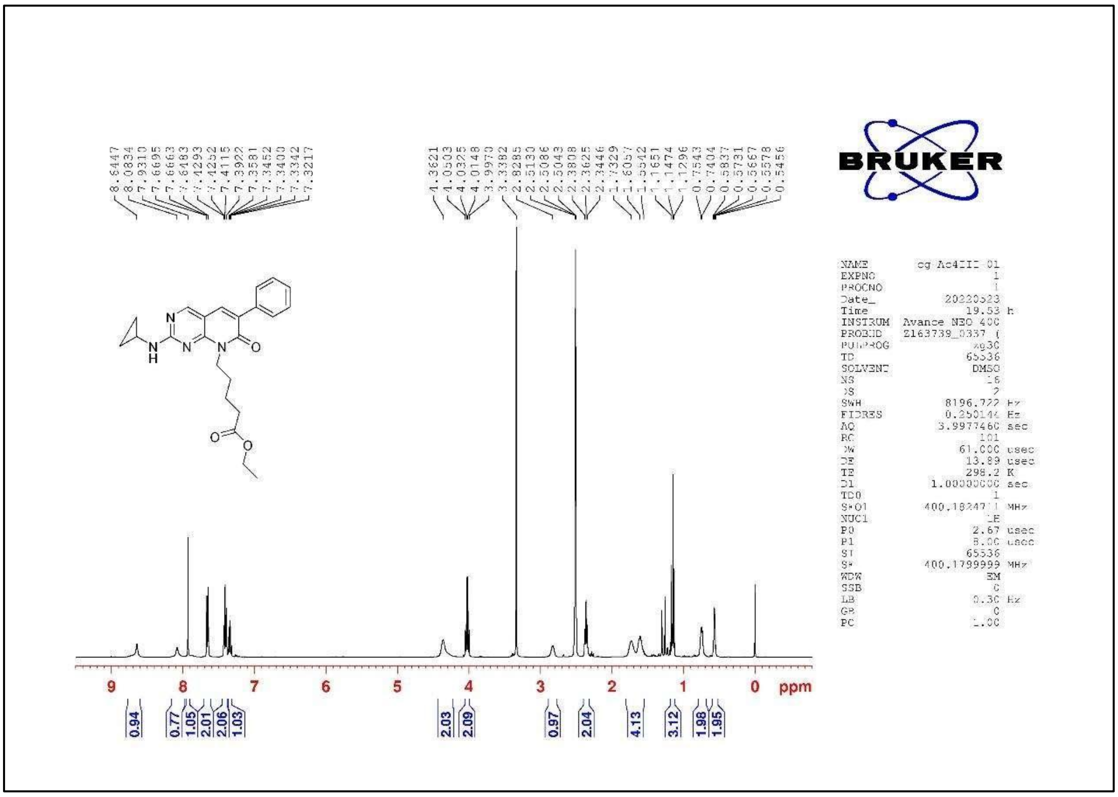

^{1}H-NMR spectrum of compound 19b

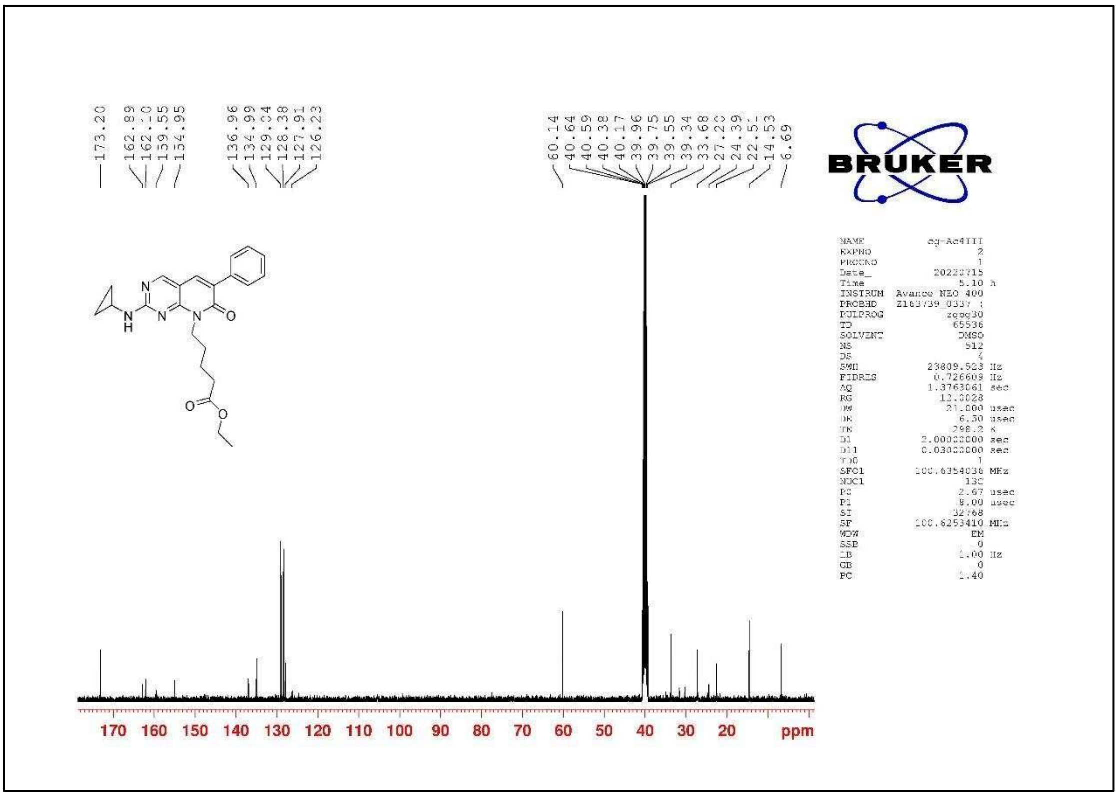

^{13}C-NMR spectrum of compound 19b

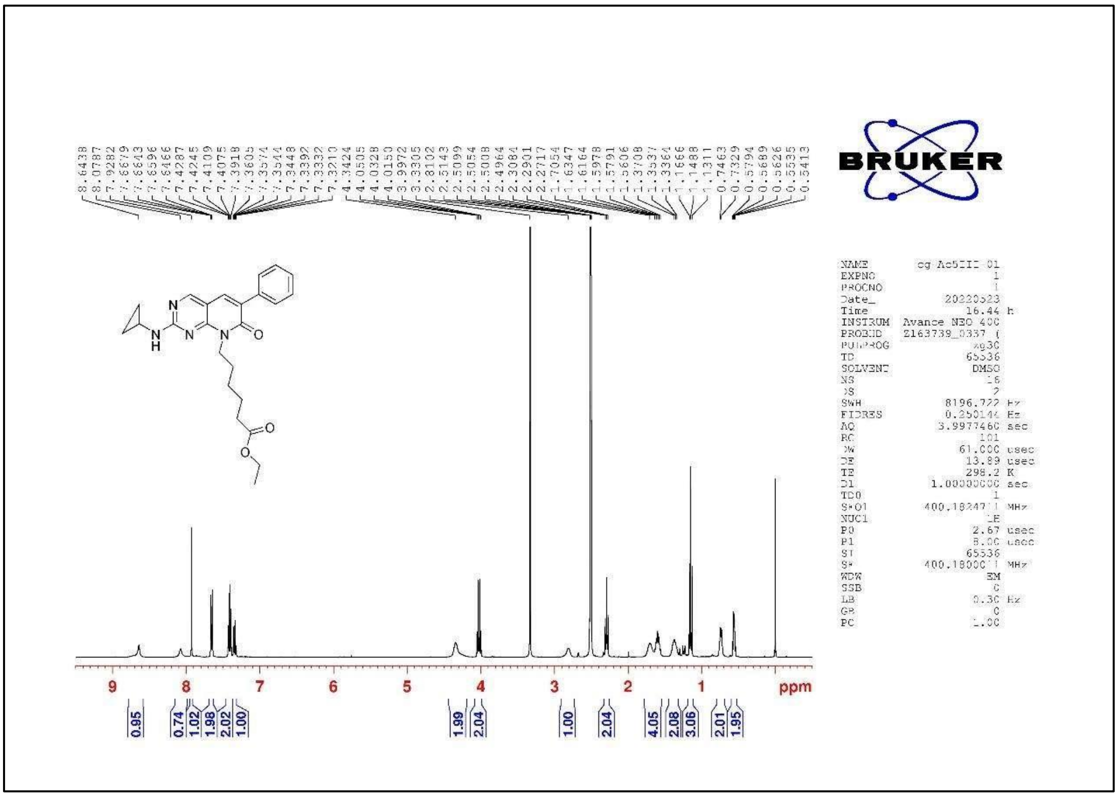

^{1}H-NMR spectrum of compound 19c

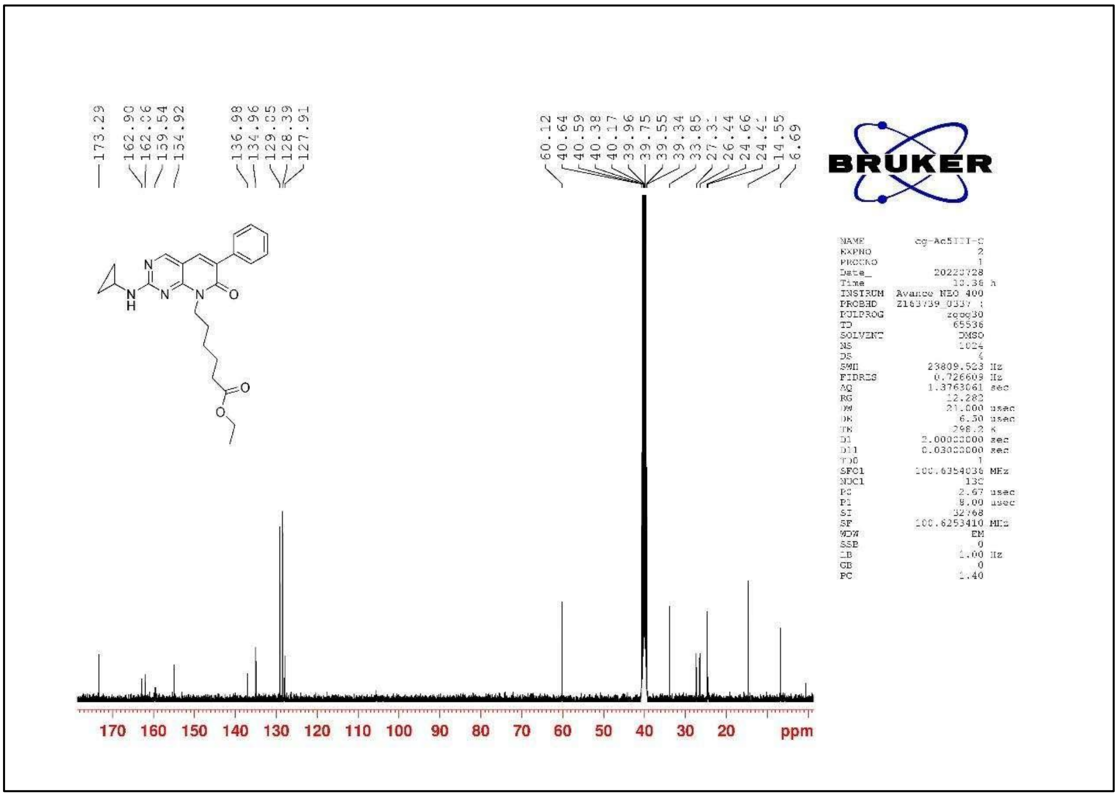

^{13}C-NMR spectrum of compound 19c

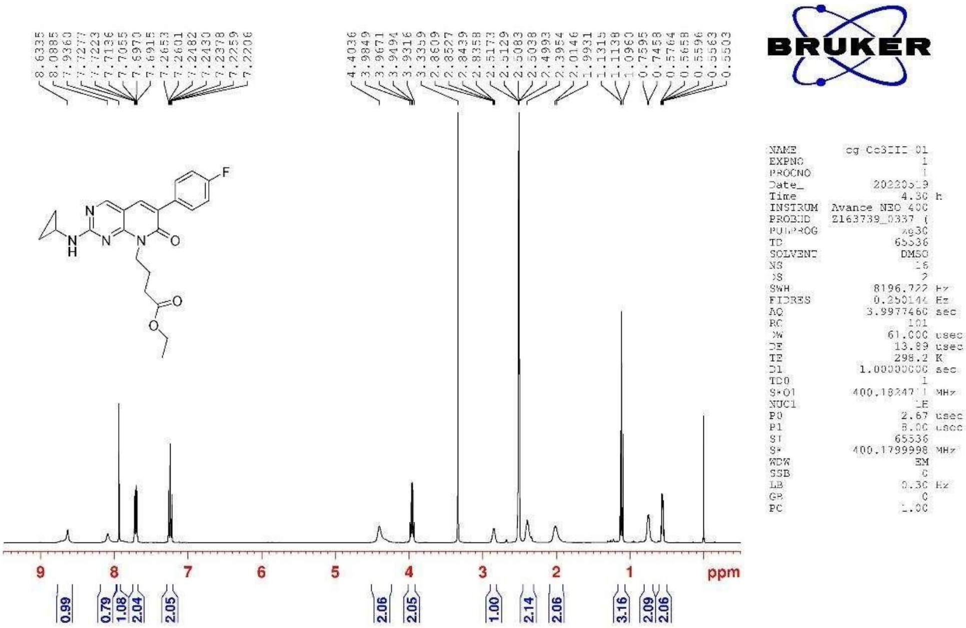

^{1}H-NMR spectrum of compound 19d

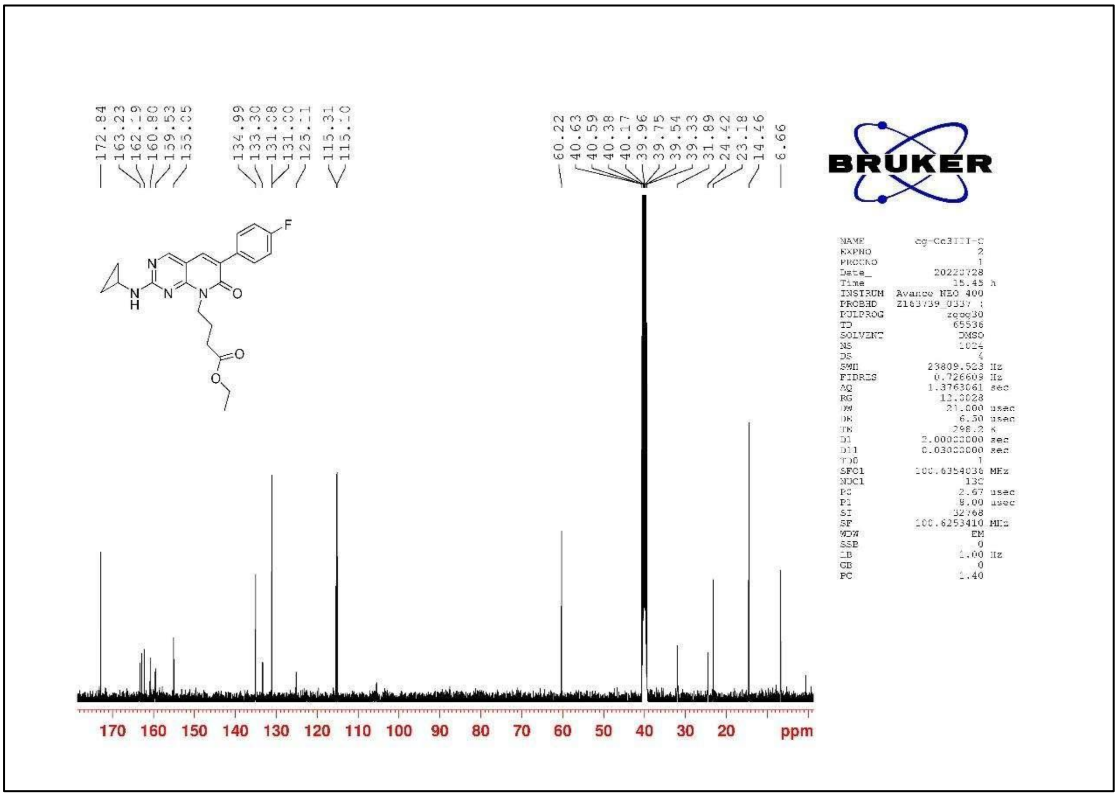

^{13}C-NMR spectrum of compound 19d

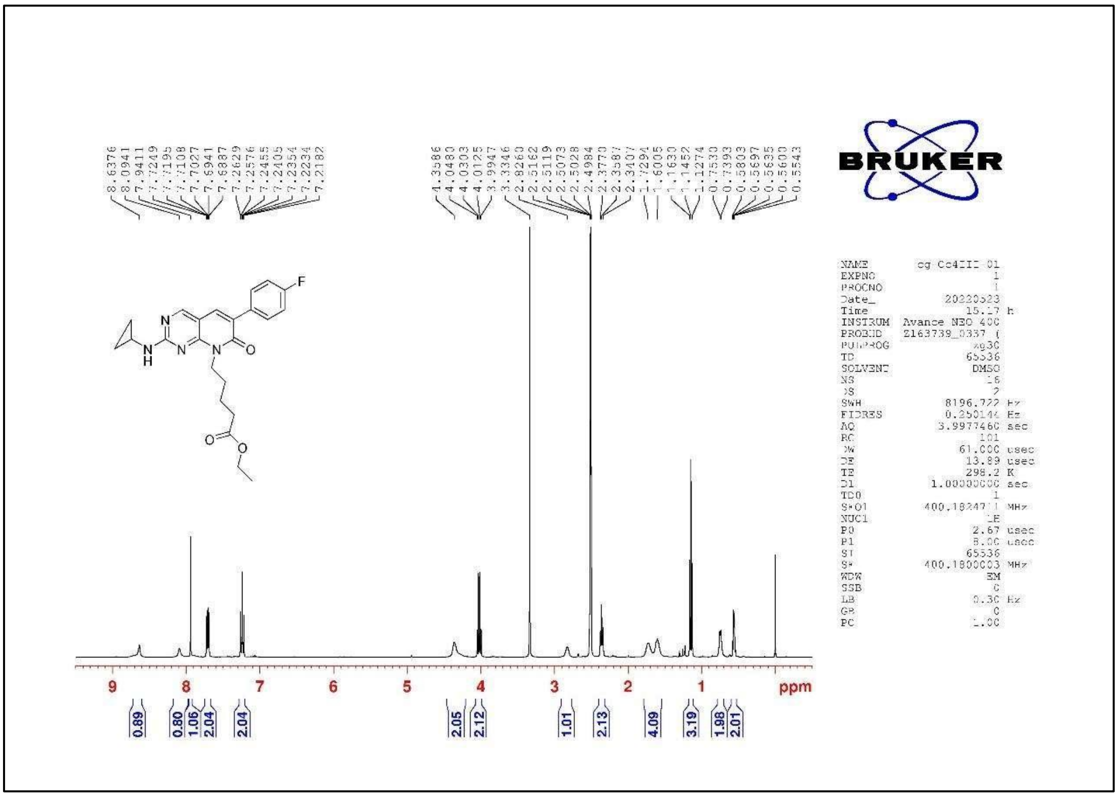

^{1}H-NMR spectrum of compound 19e

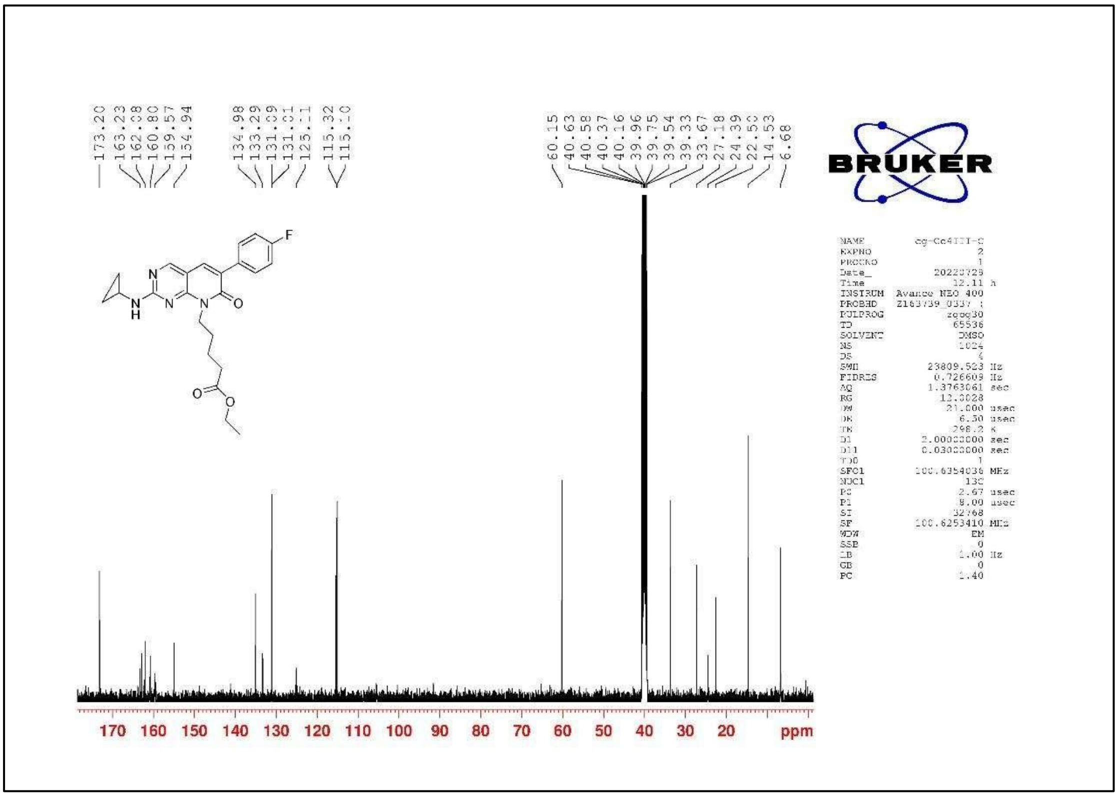

^{13}C-NMR spectrum of compound 19e

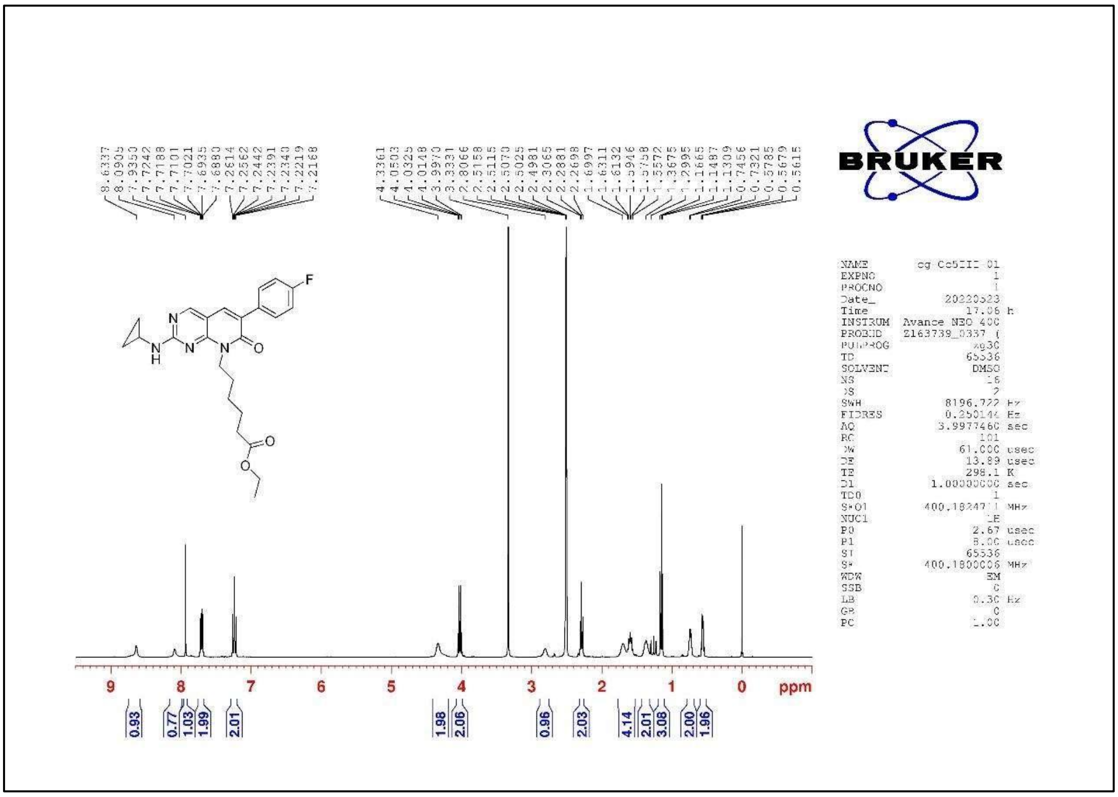

^{1}H-NMR spectrum of compound 19f

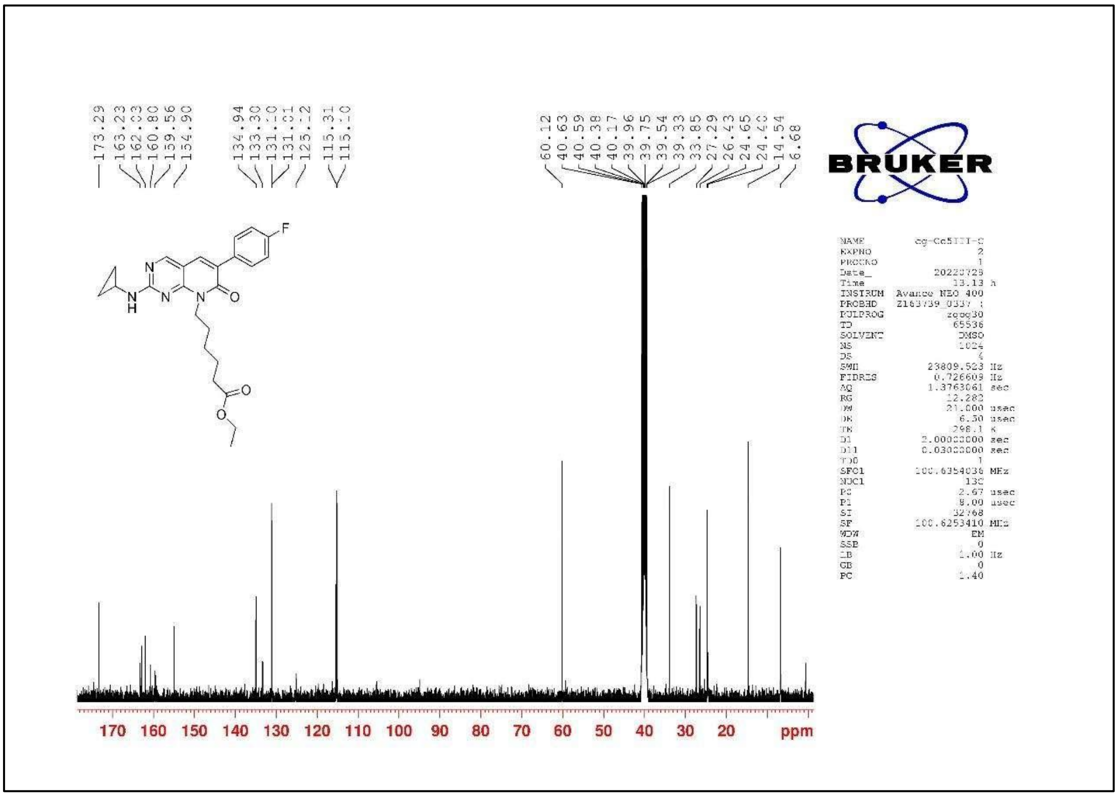

^{13}C-NMR spectrum of compound 19f

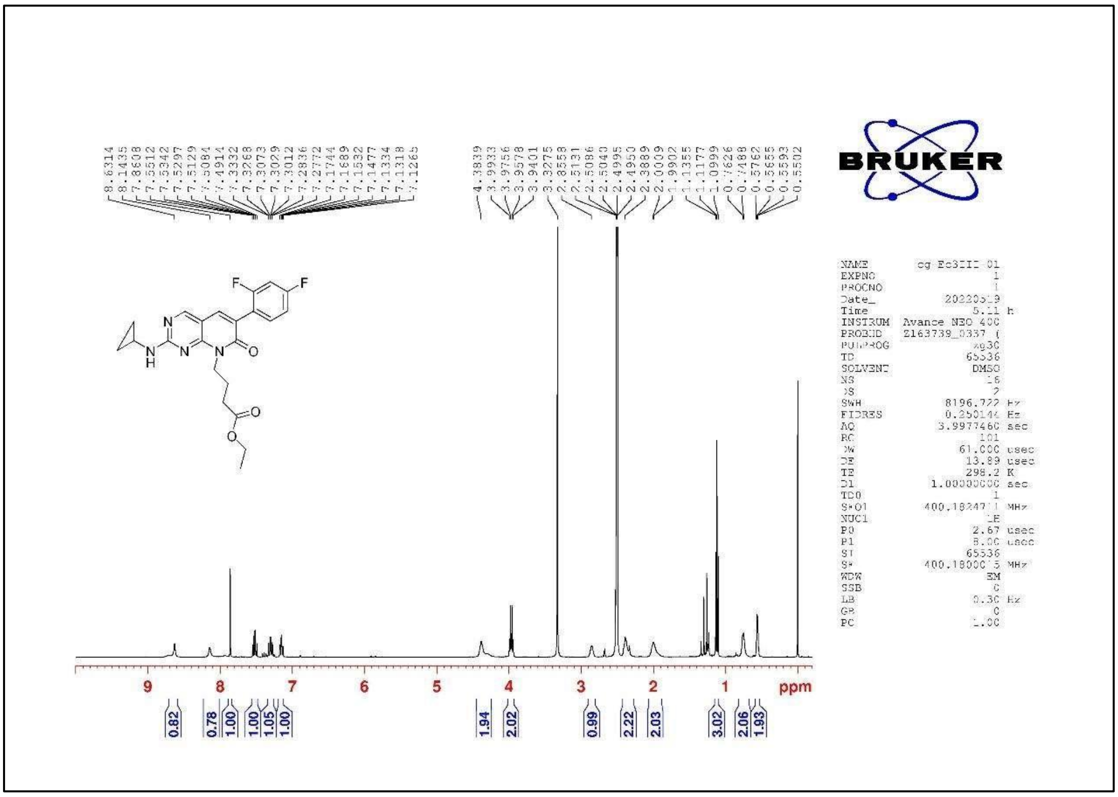

^{1}H-NMR spectrum of compound 19g

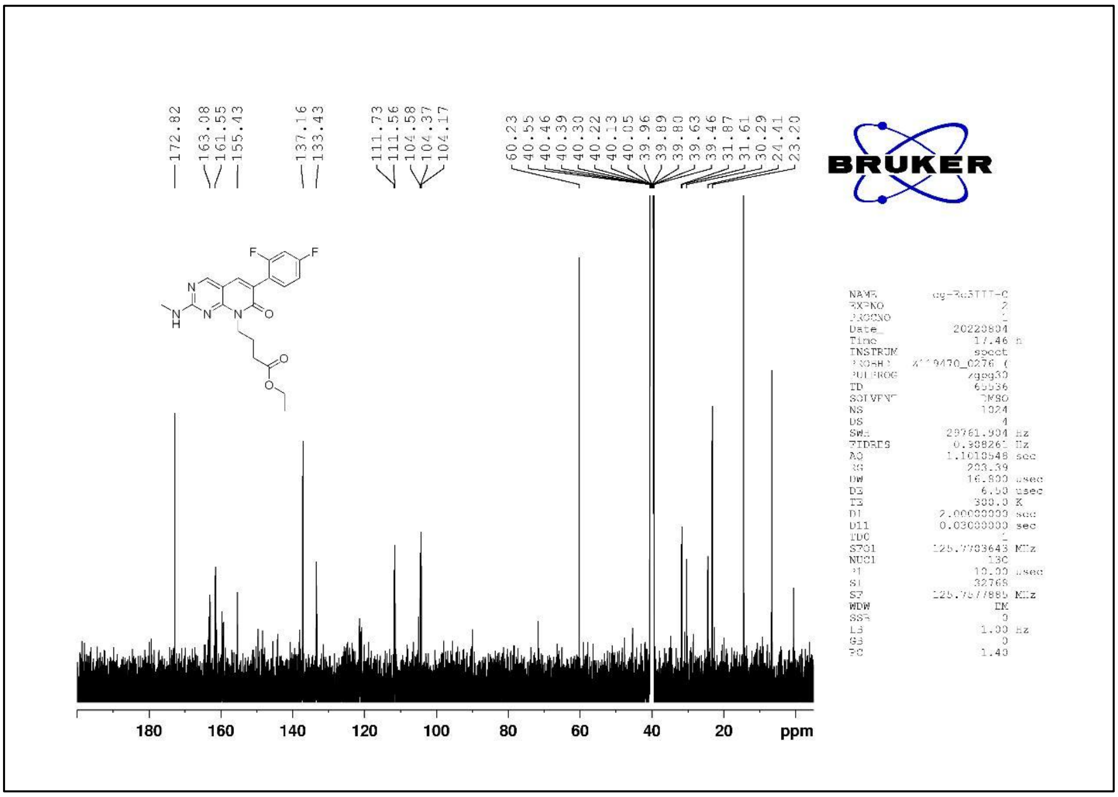

^{13}C-NMR spectrum of compound 19g

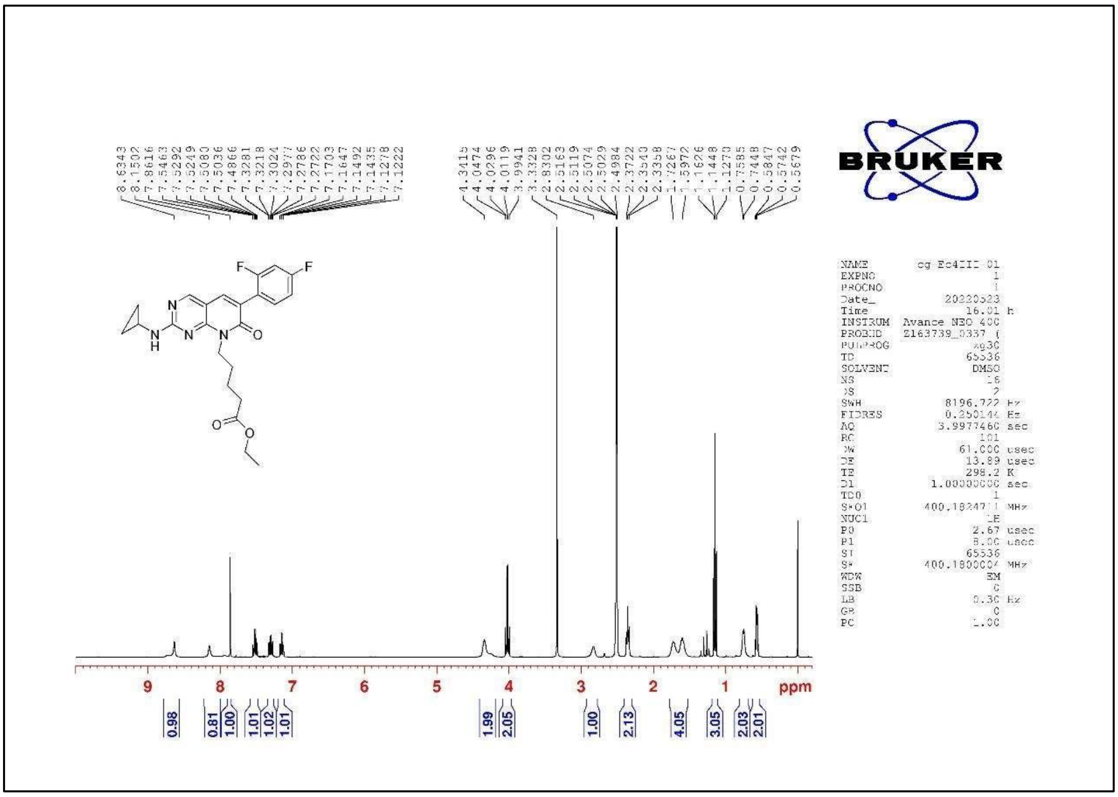

^{1}H-NMR spectrum of compound 19h

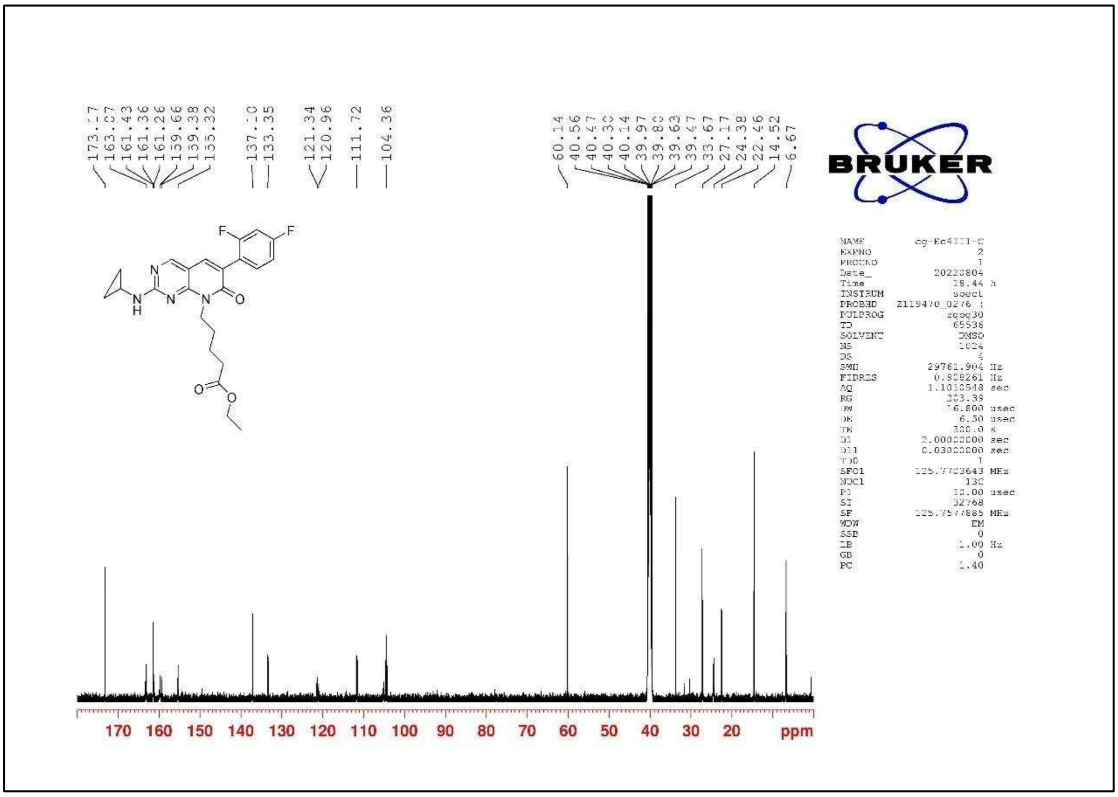

^{13}C-NMR spectrum of compound 19h

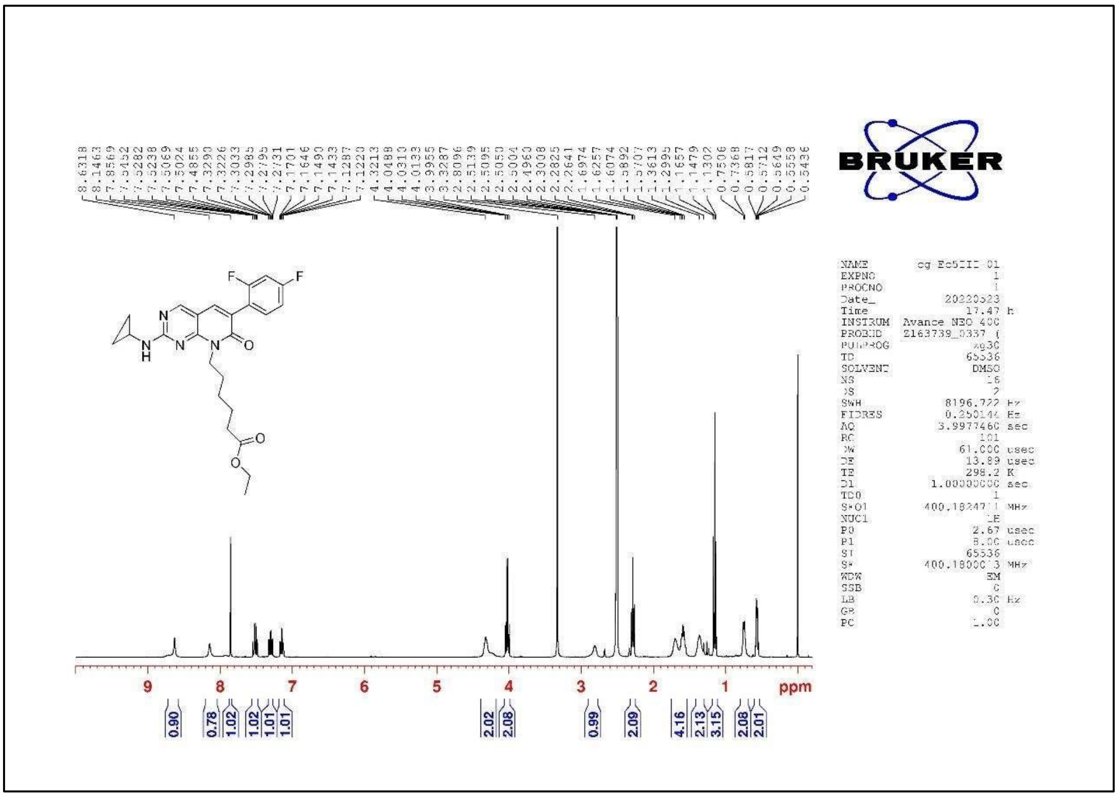

^{1}H-NMR spectrum of compound 19i

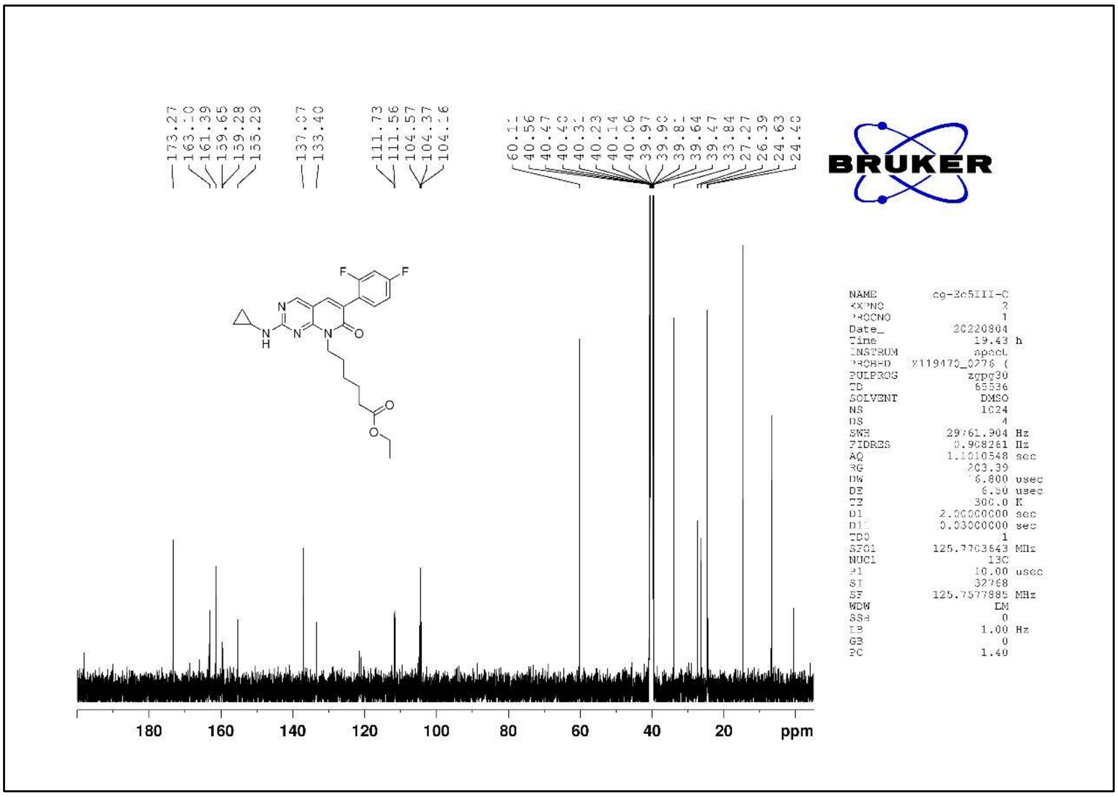

^{13}C-NMR spectrum of compound 19i

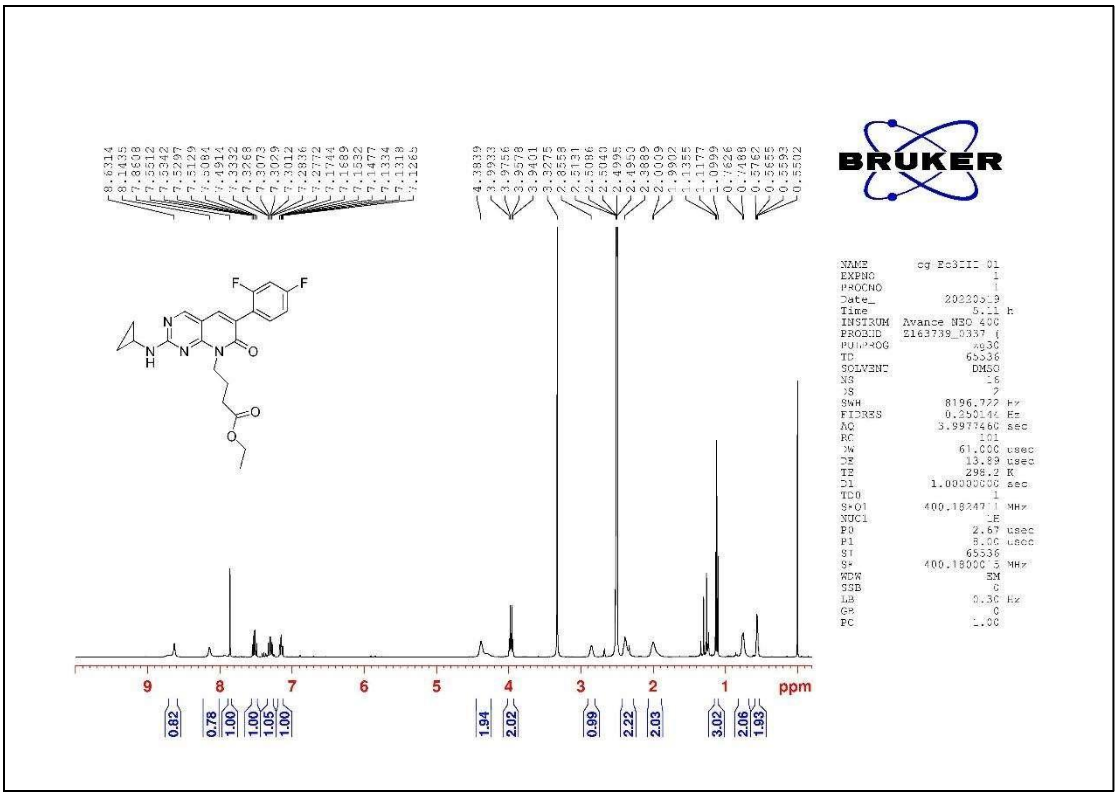

^{1}H-NMR spectrum of compound 19j

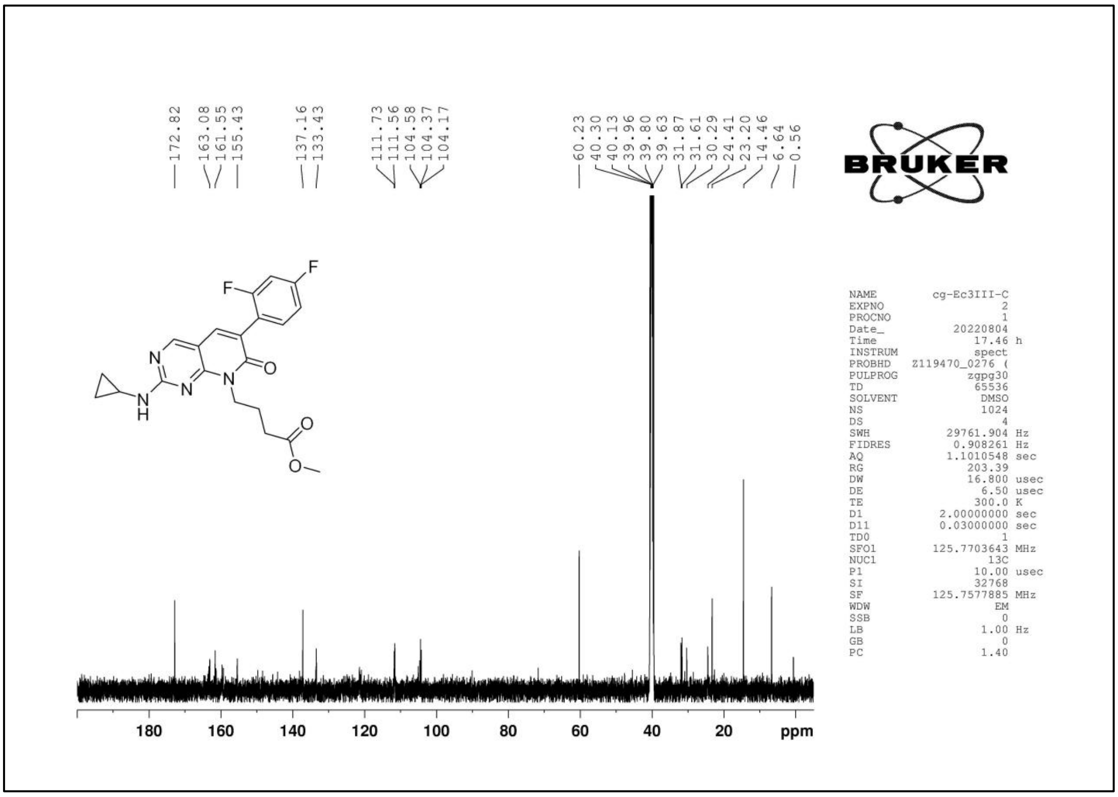

^{13}C-NMR spectrum of compound 19j

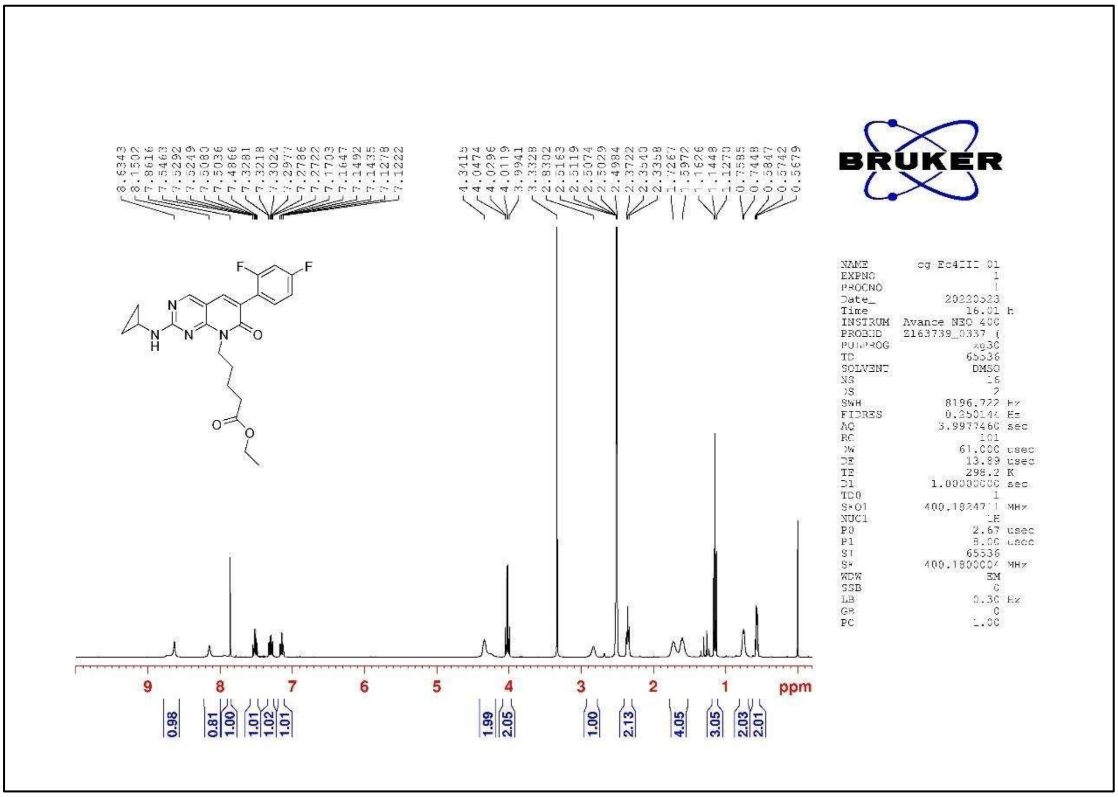

^{1}H-NMR spectrum of compound 19k

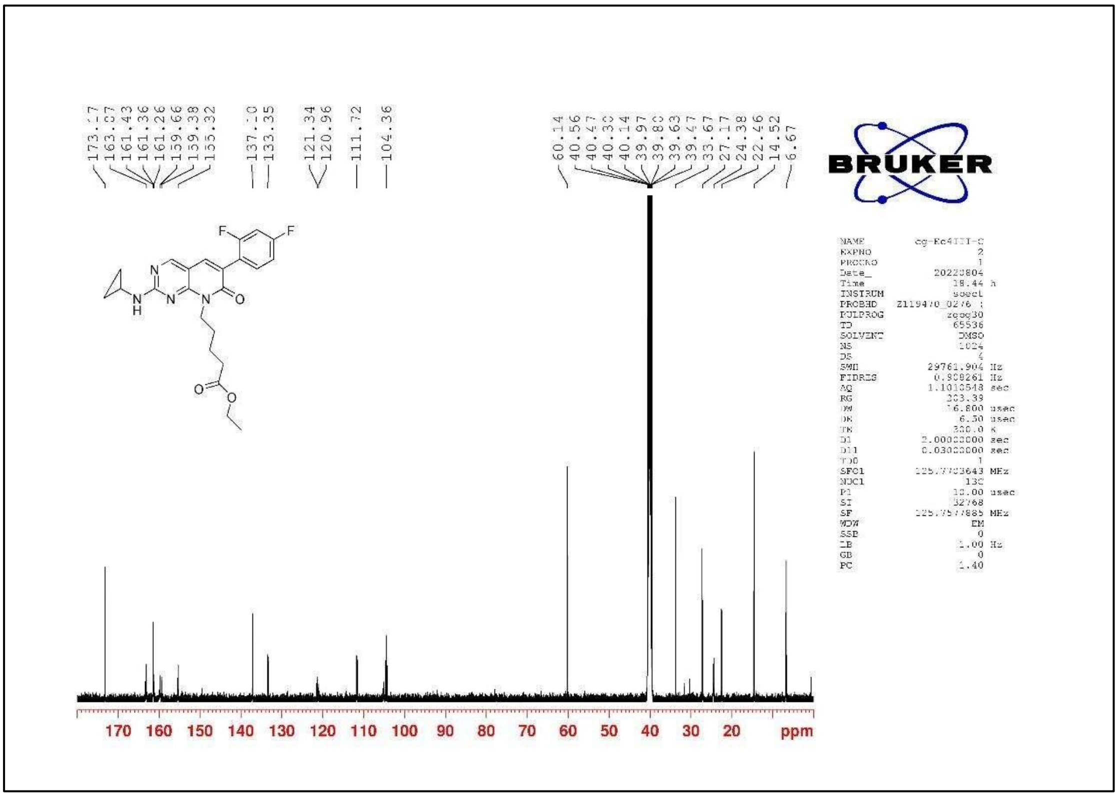

^{13}C-NMR spectrum of compound 19k

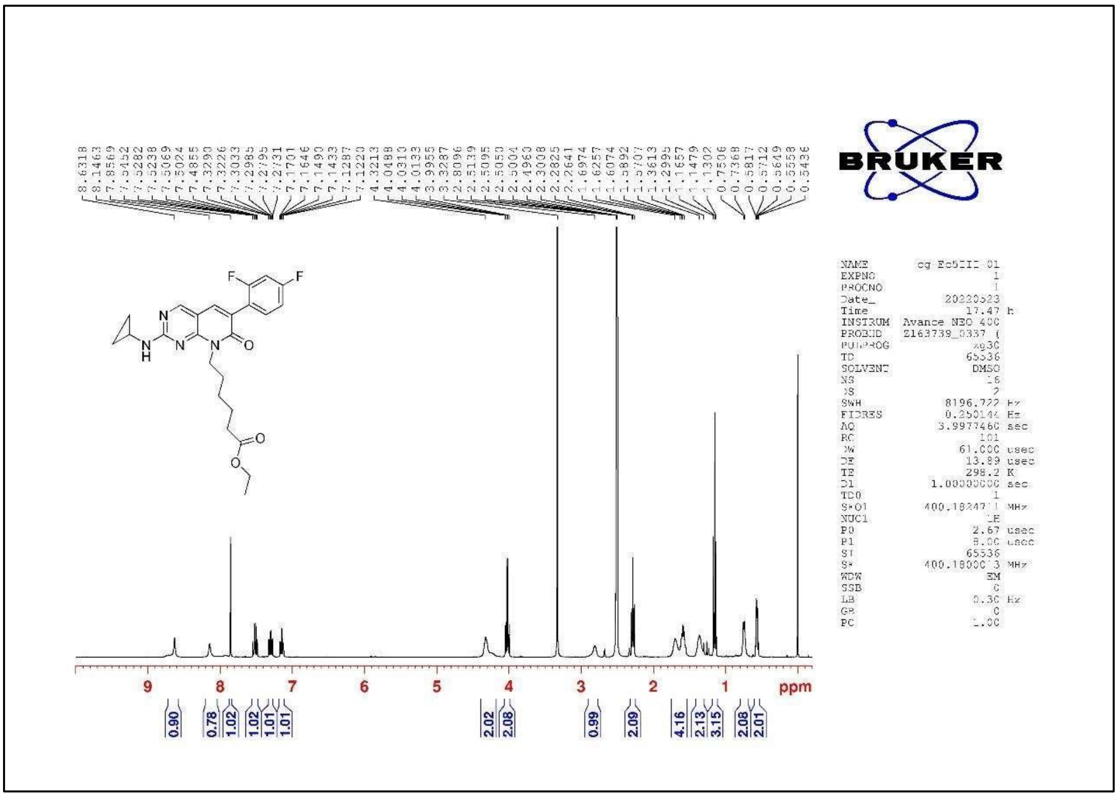

^{1}H-NMR spectrum of compound 19l

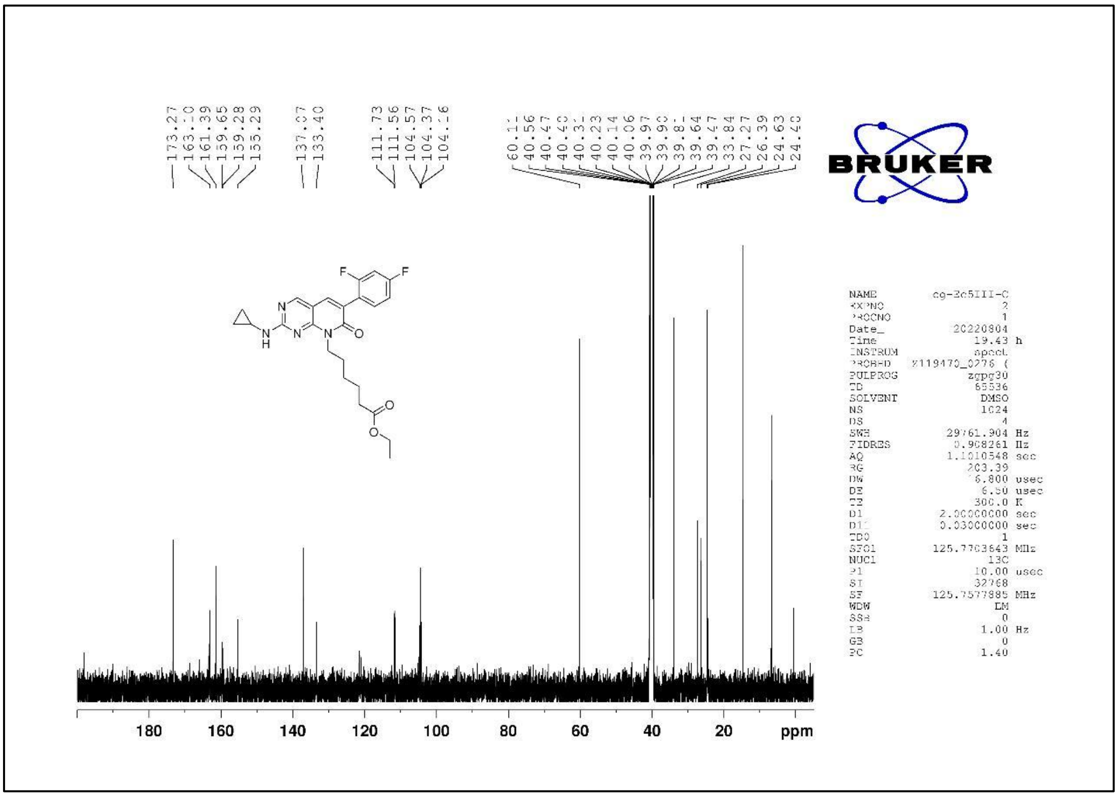

^{13}C-NMR spectrum of compound 19l

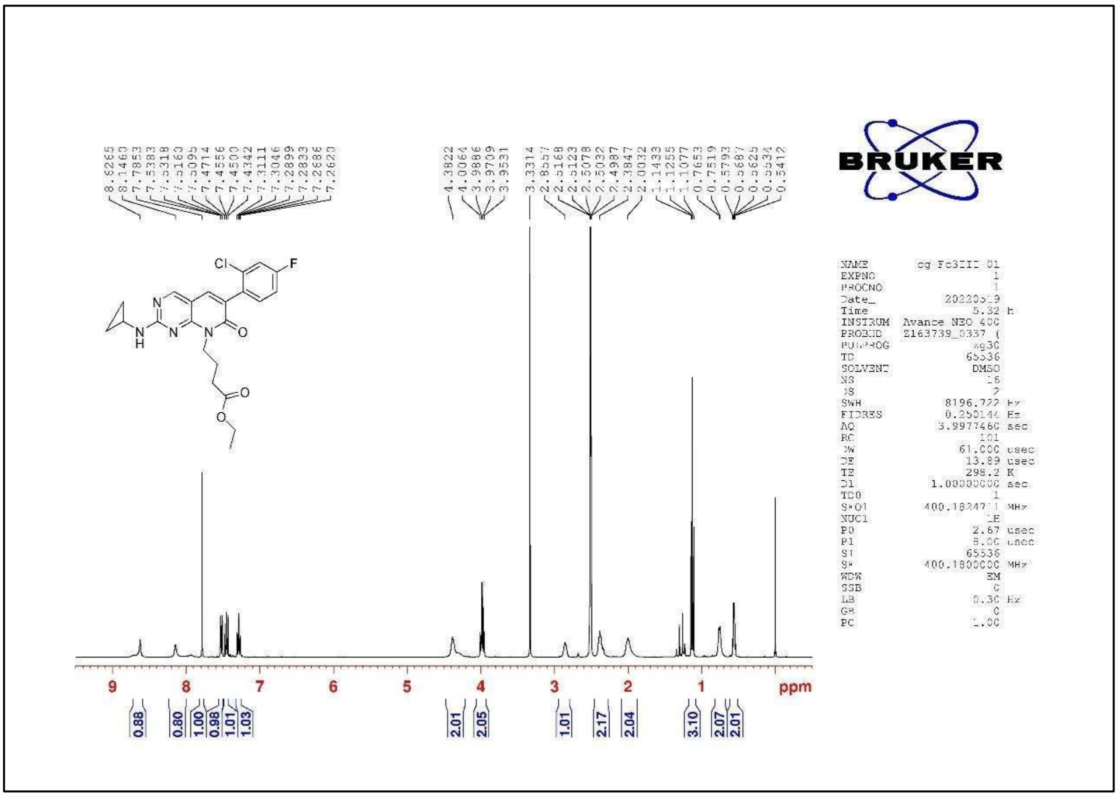

^{1}H-NMR spectrum of compound 19m

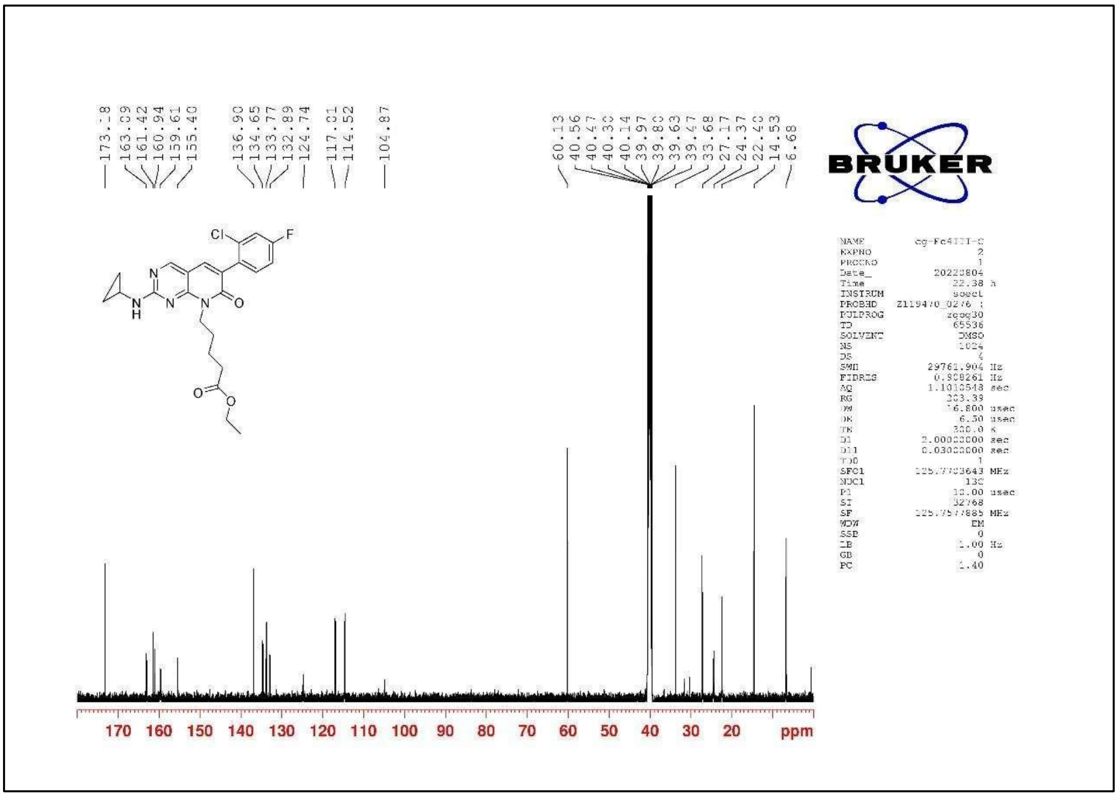

^{13}C-NMR spectrum of compound 19m

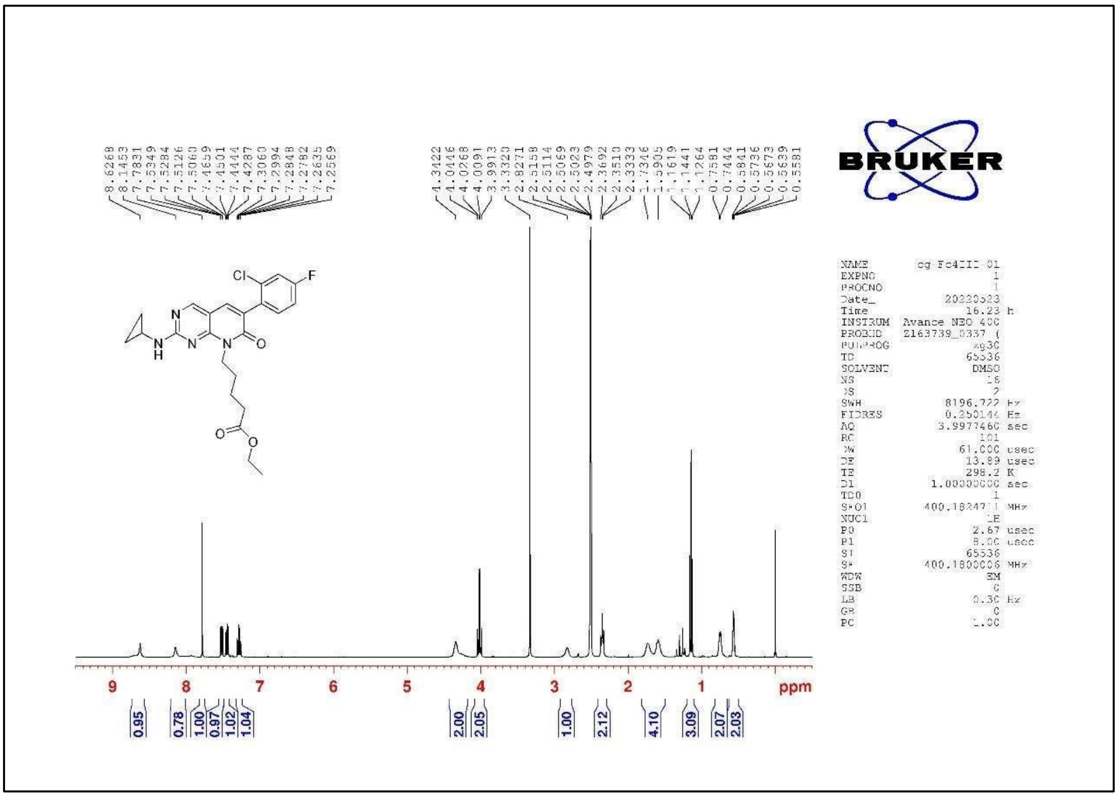

^{1}H-NMR spectrum of compound 19n

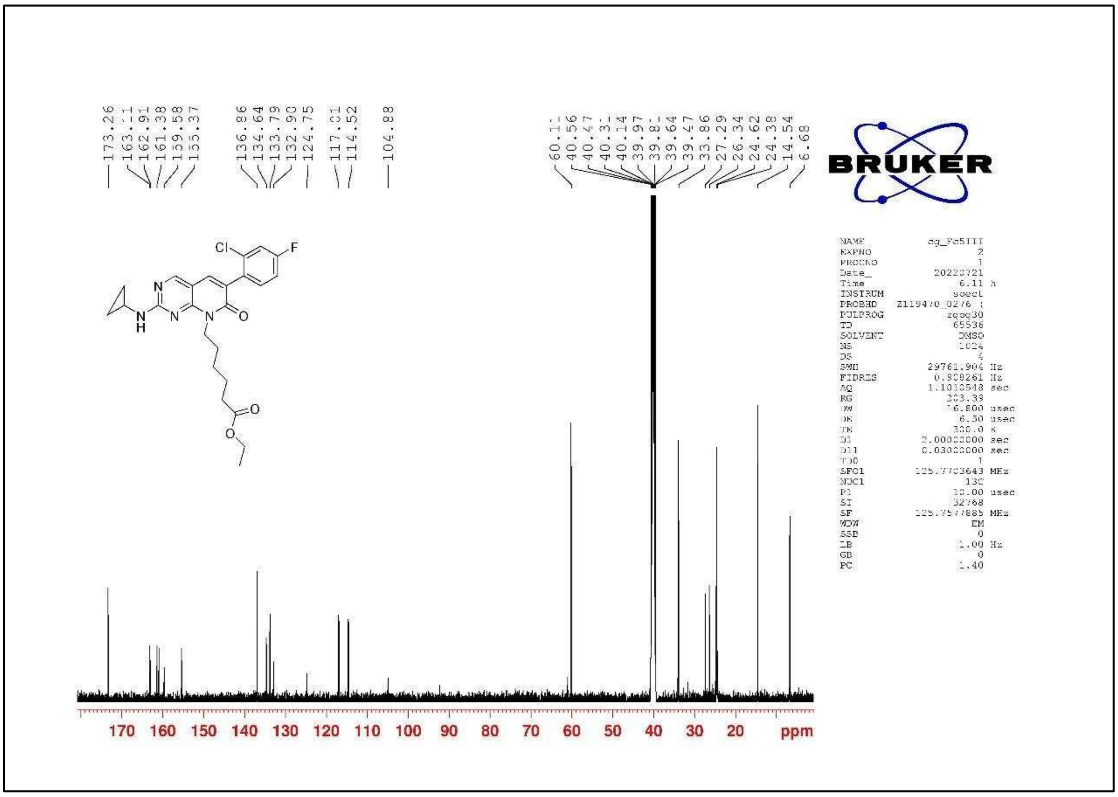

^{13}C-NMR spectrum of compound 19n

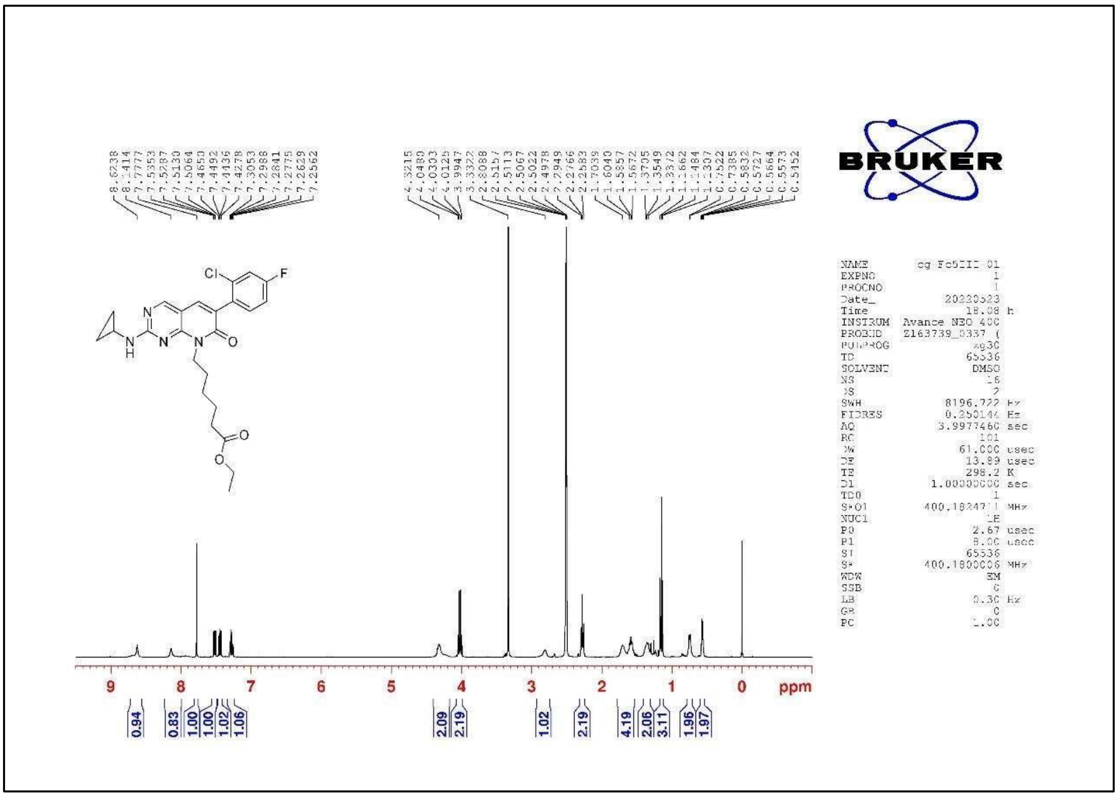

^{1}H-NMR spectrum of compound 19o

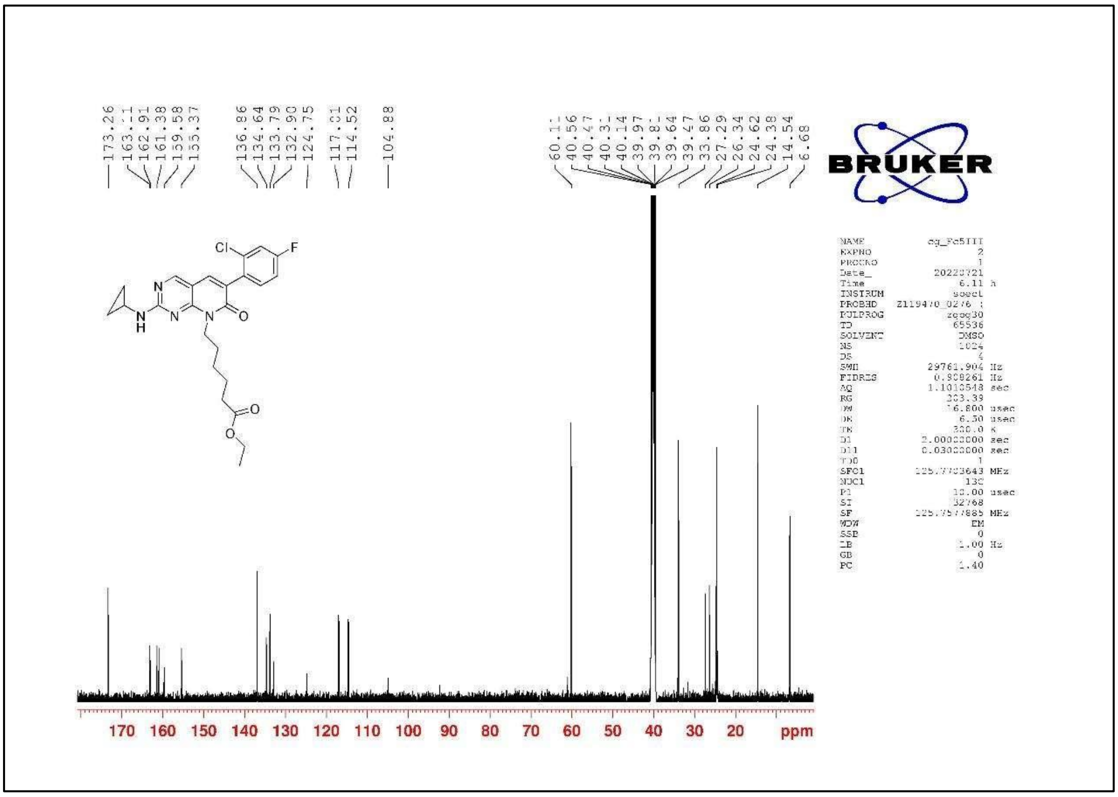

^{13}C-NMR spectrum of compound 19o

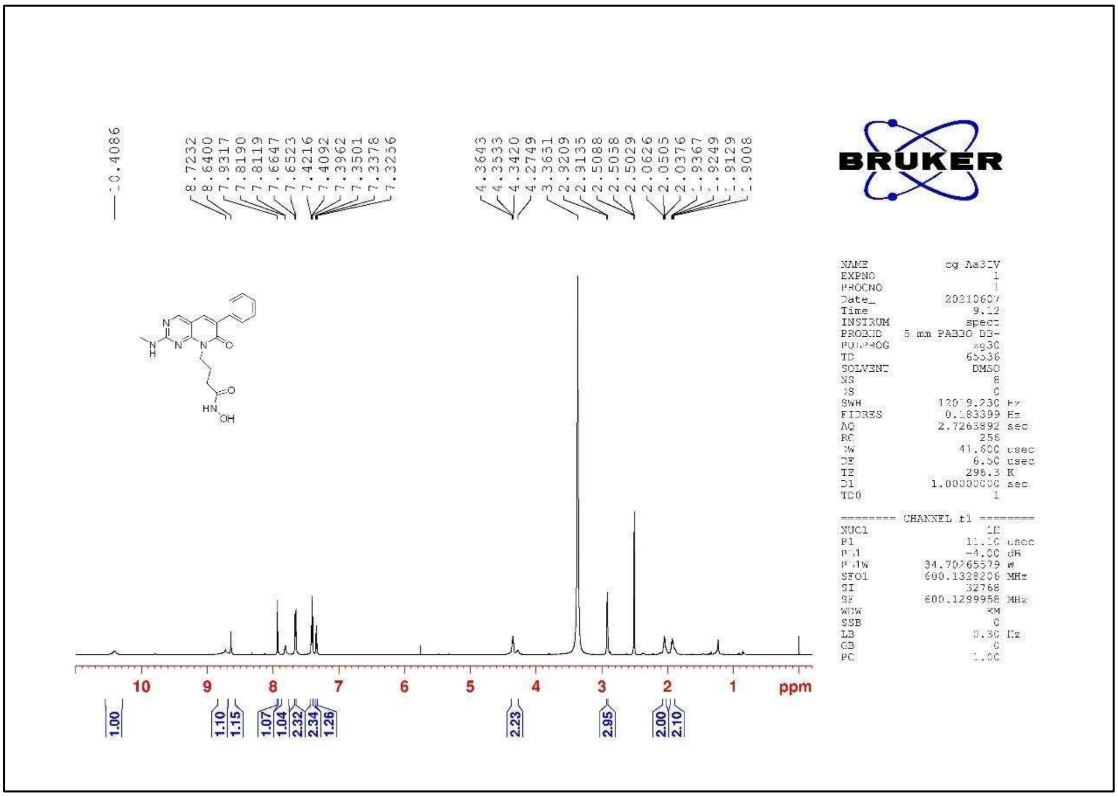

^{1}H-NMR spectrum of compound 20a

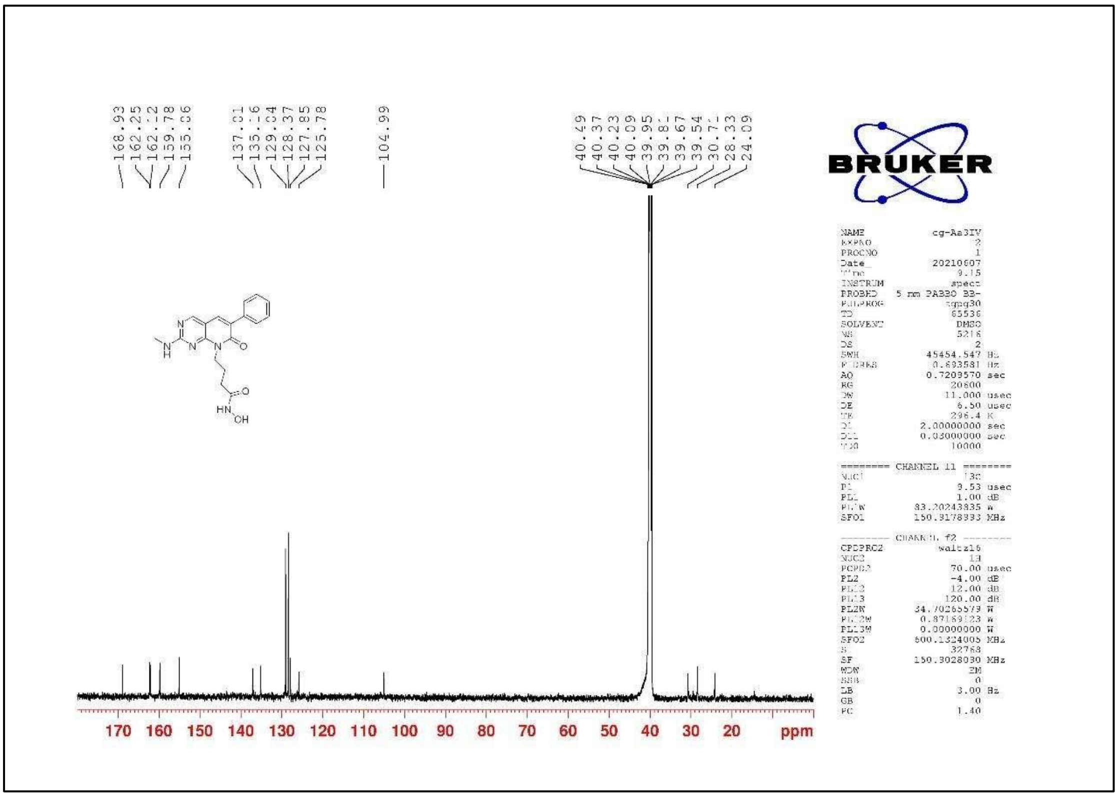

^{13}C-NMR spectrum of compound 20a

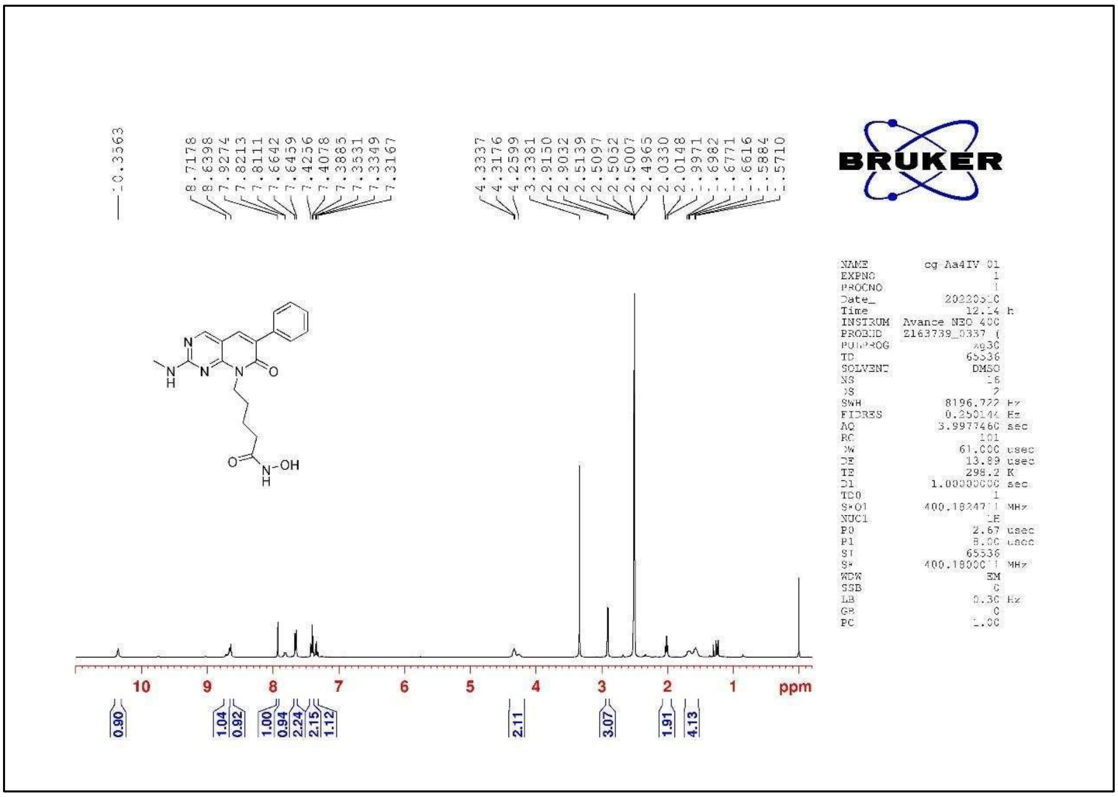

^{1}H-NMR spectrum of compound 20b

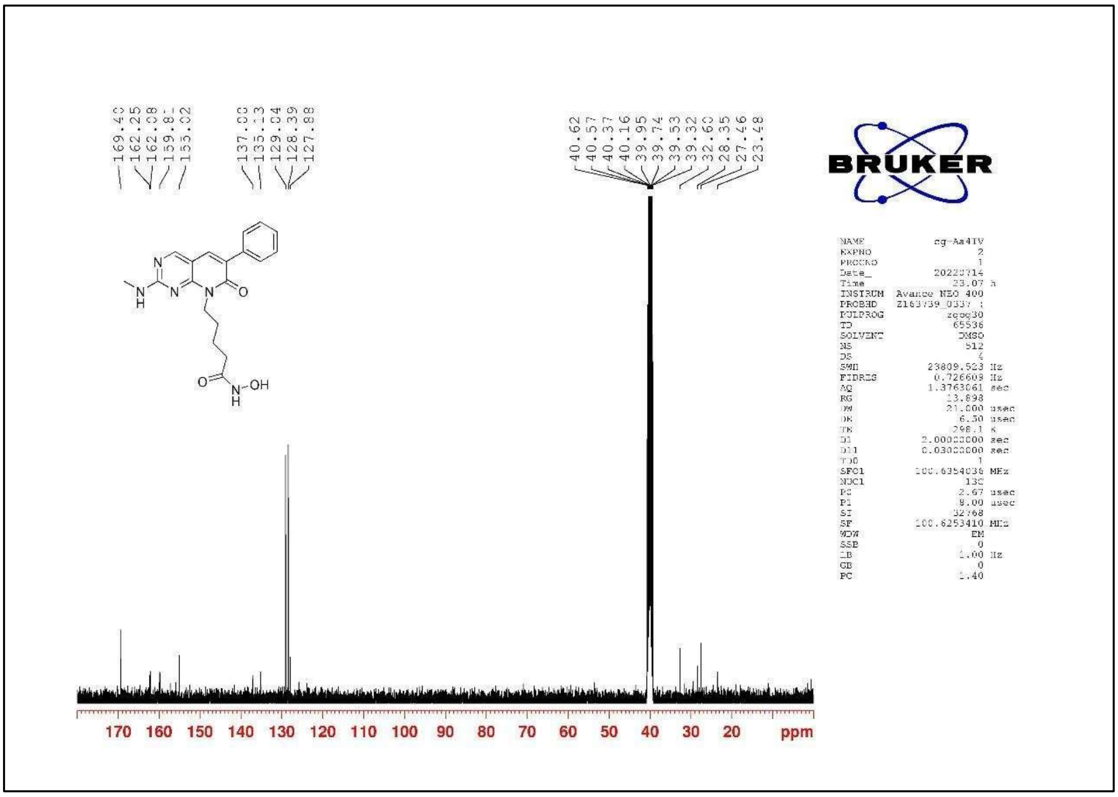

^{13}C-NMR spectrum of compound 20b

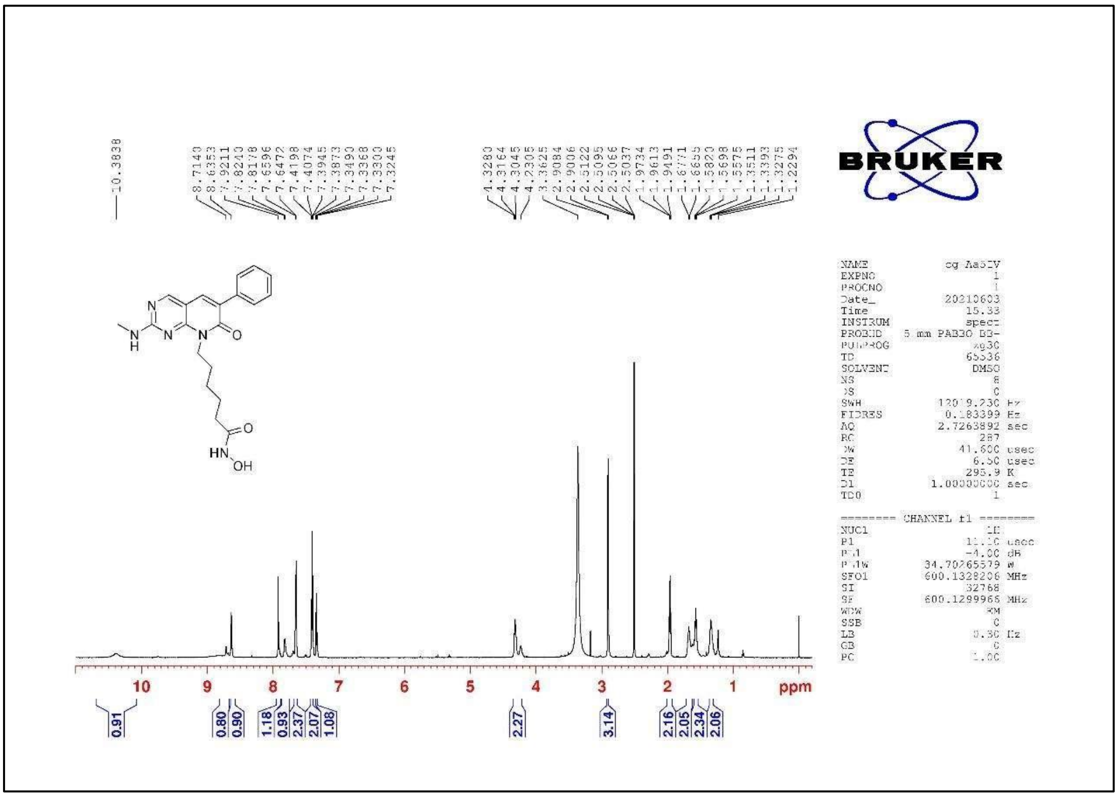

^{1}H-NMR spectrum of compound 20c

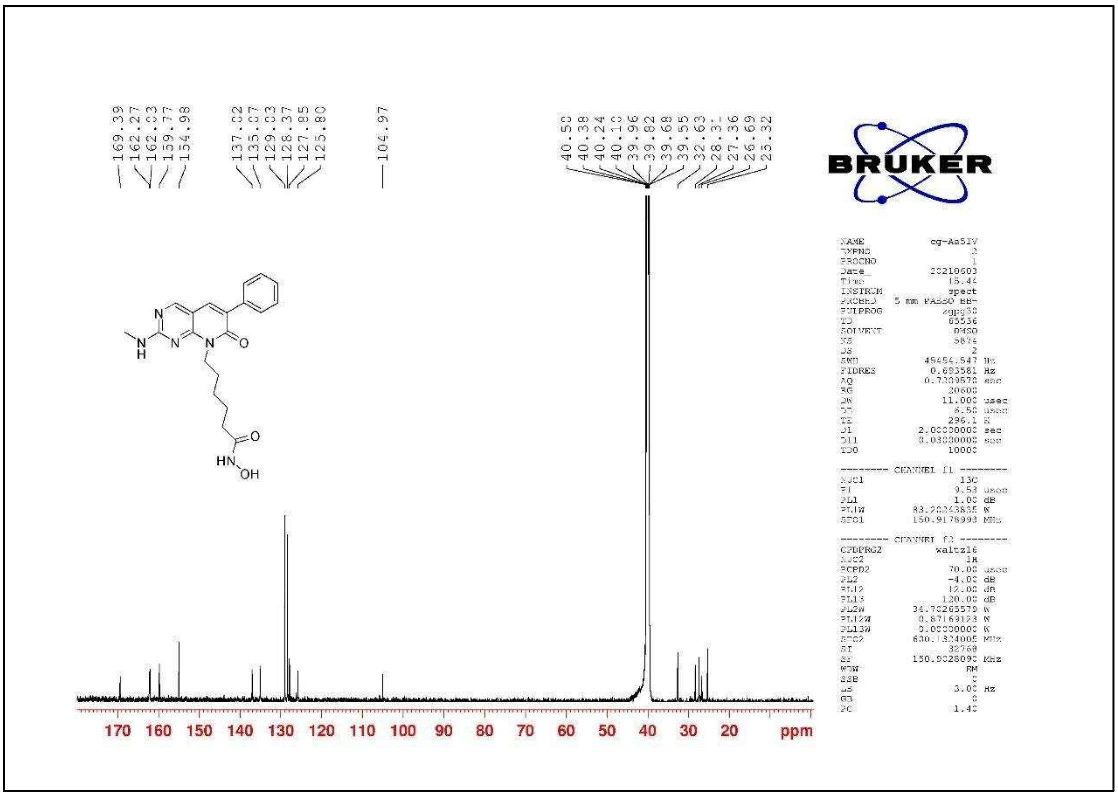

^{13}C-NMR spectrum of compound 20c

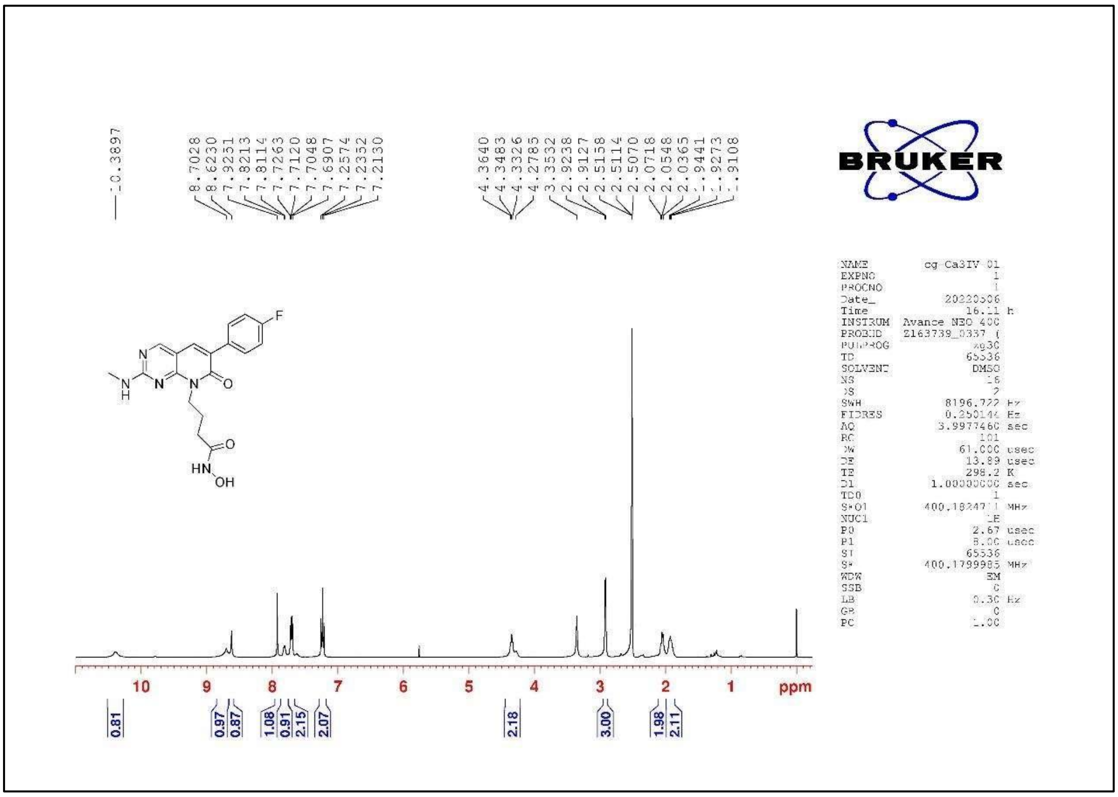

^{1}H-NMR spectrum of compound 20d

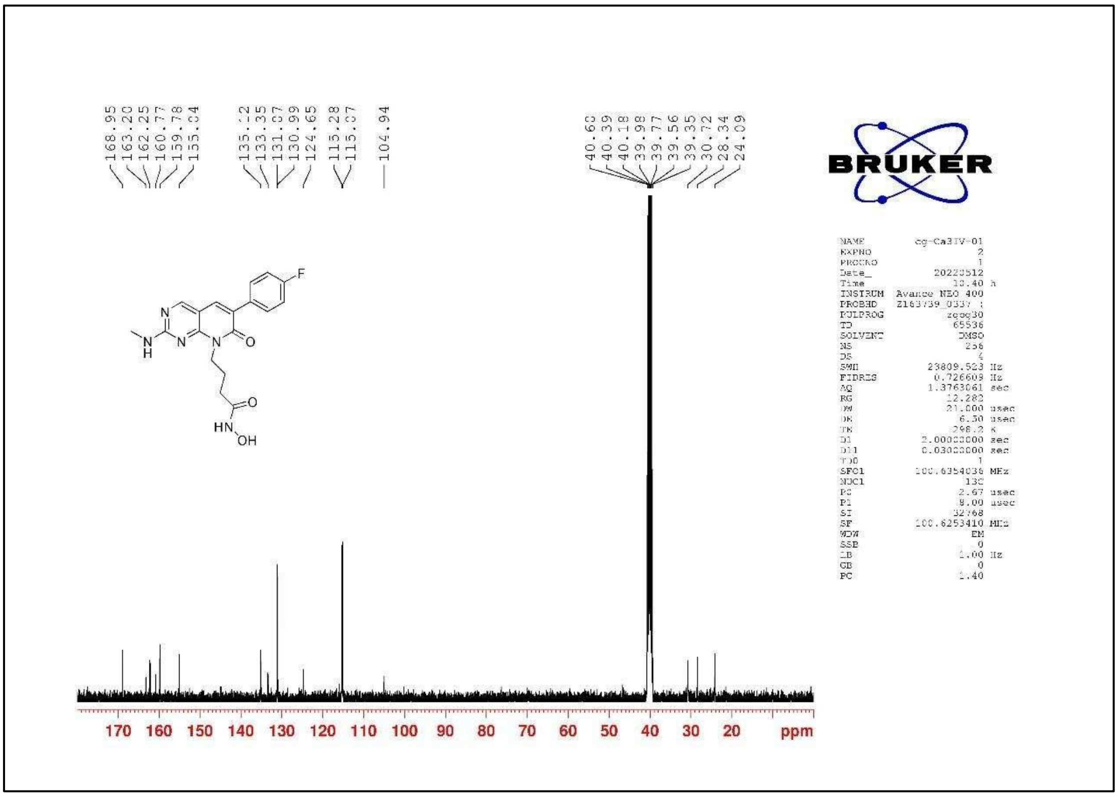

^{13}C-NMR spectrum of compound 20d

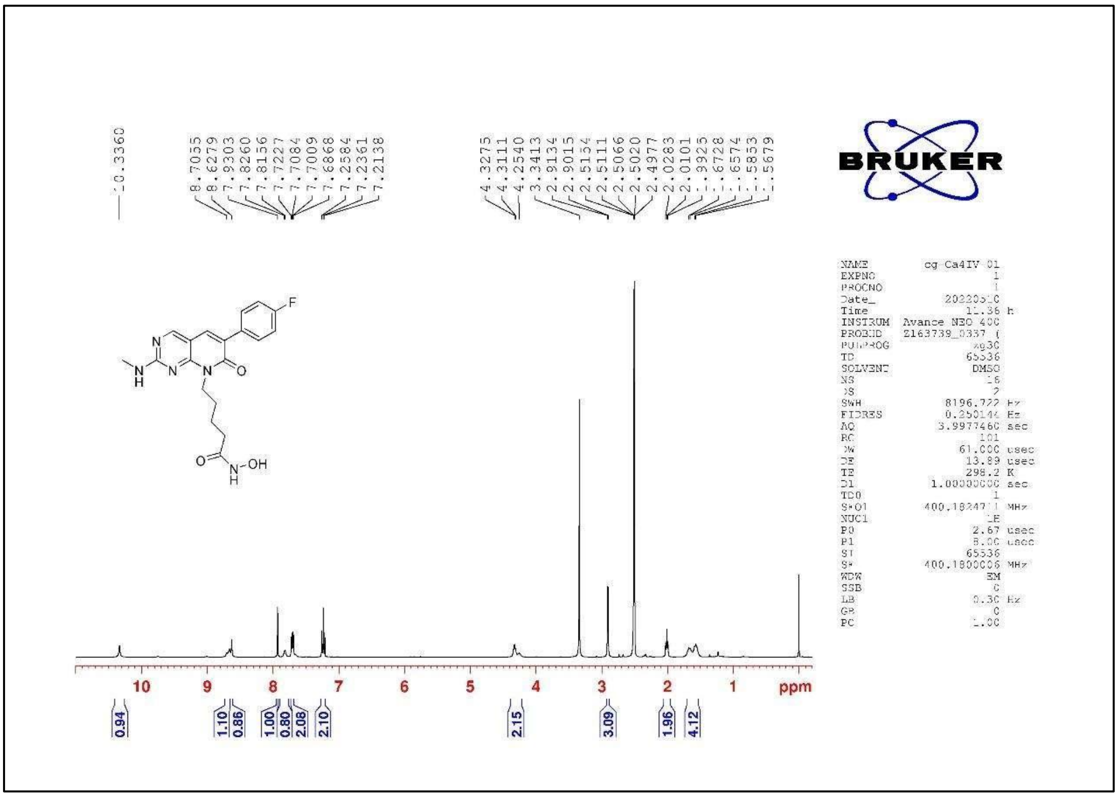

^{1}H-NMR spectrum of compound 20e

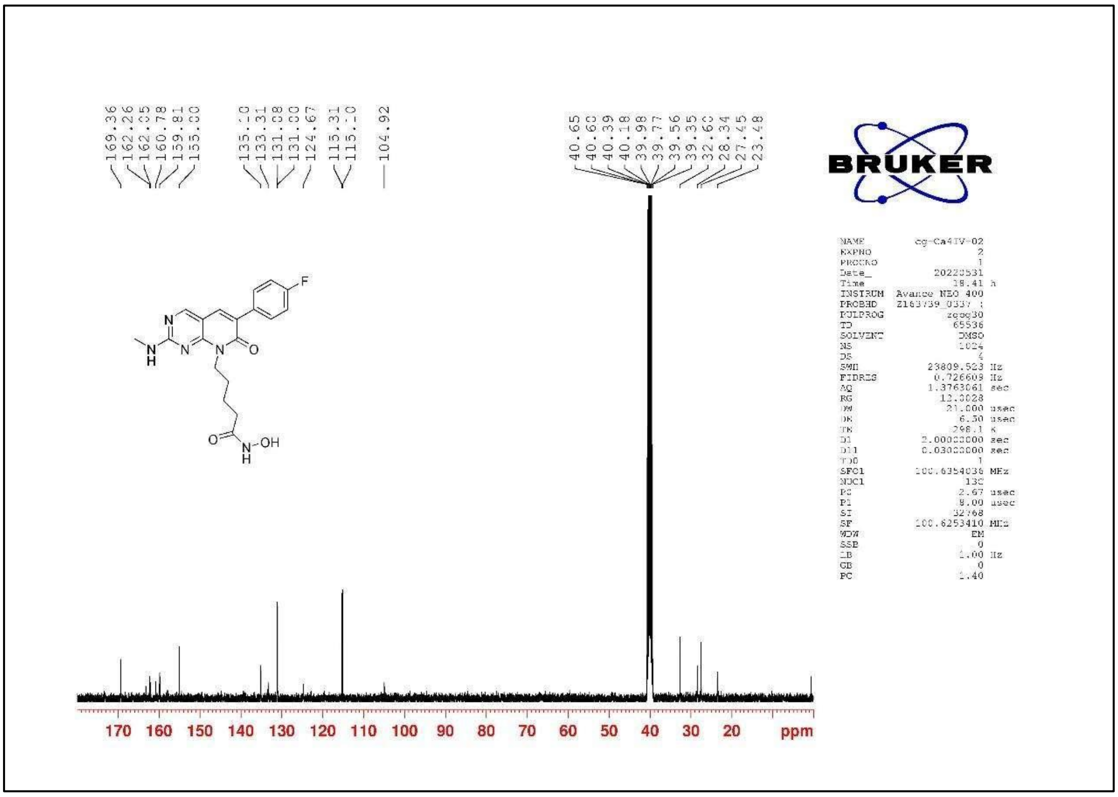

^{13}C-NMR spectrum of compound 20e

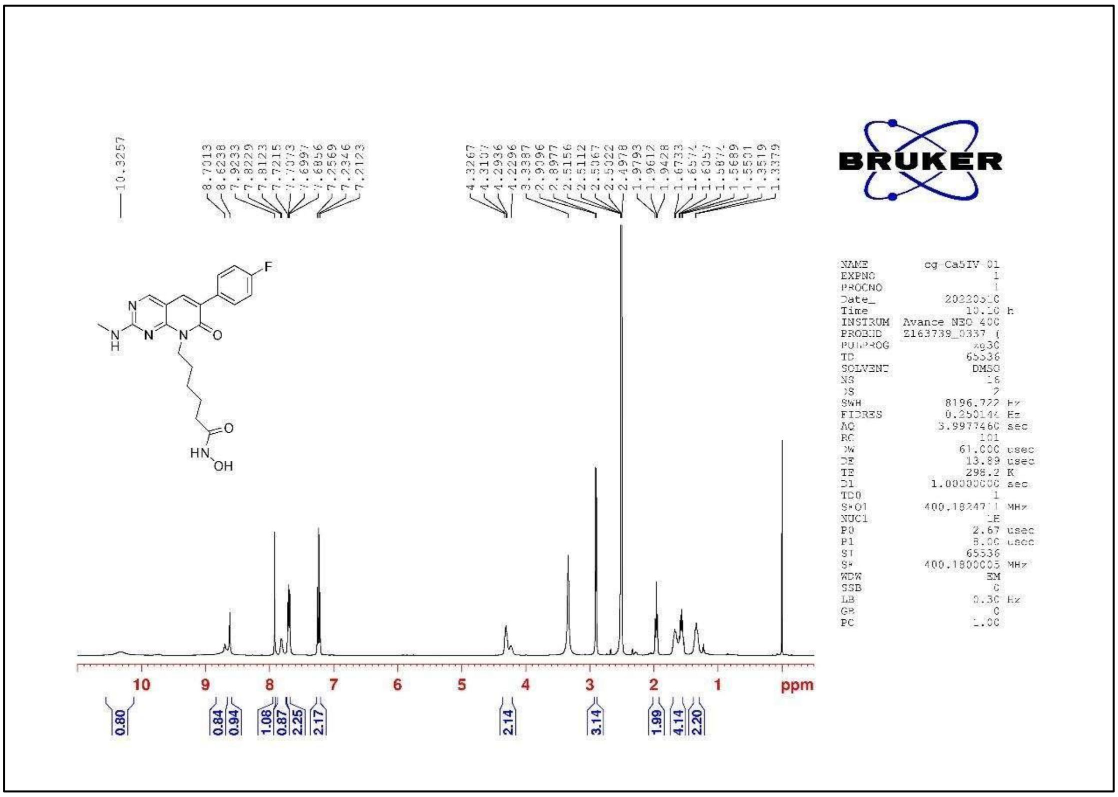

^{1}H-NMR spectrum of compound 20f

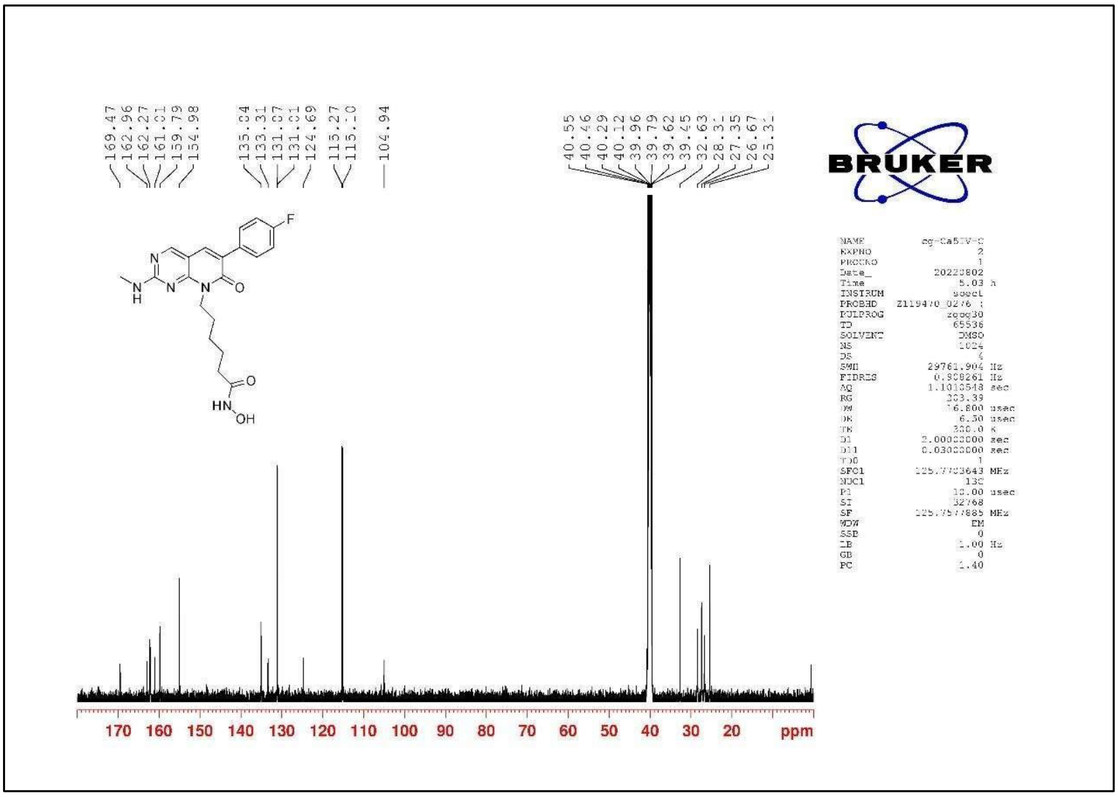

^{13}C-NMR spectrum of compound 20f

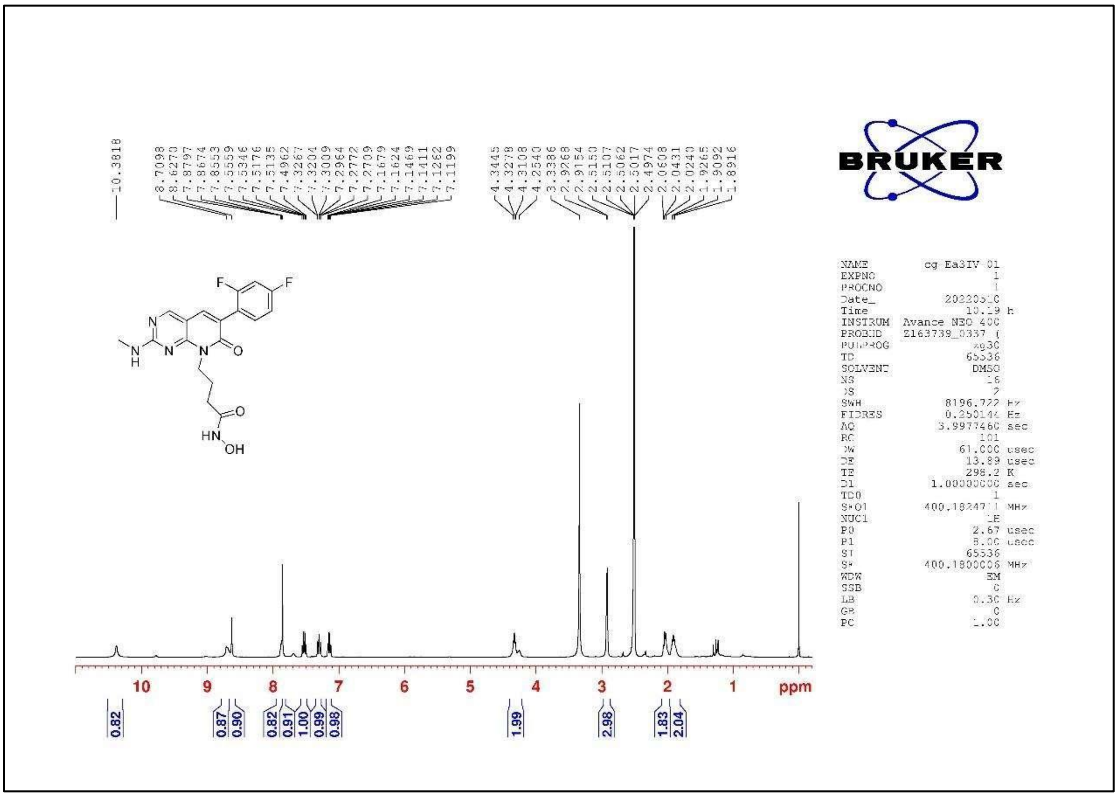

^{1}H-NMR spectrum of compound 20g

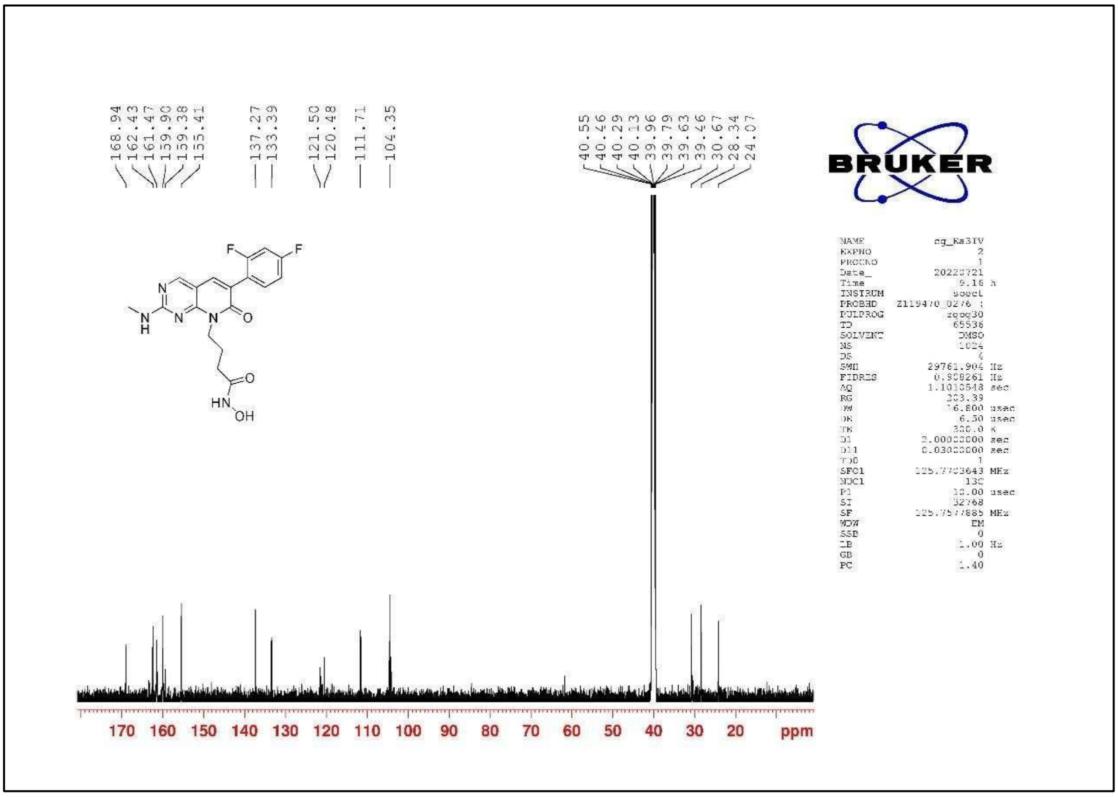

^{13}C-NMR spectrum of compound 20g

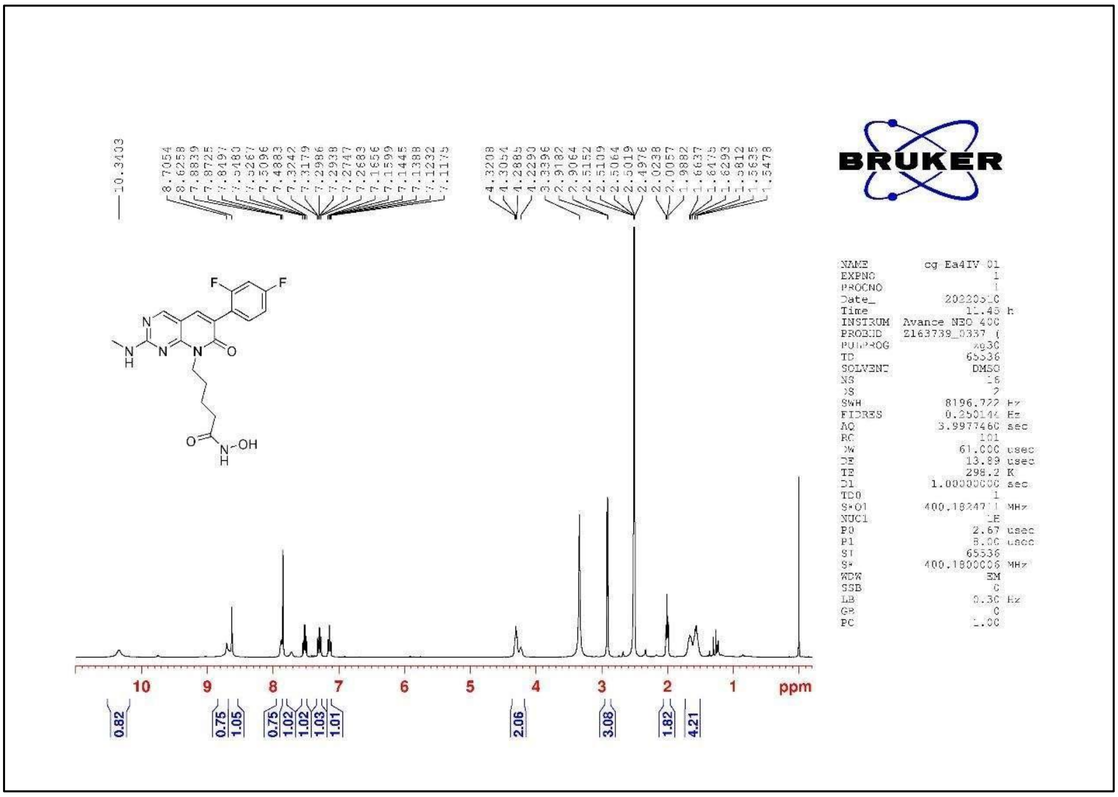

^{1}H-NMR spectrum of compound 20h

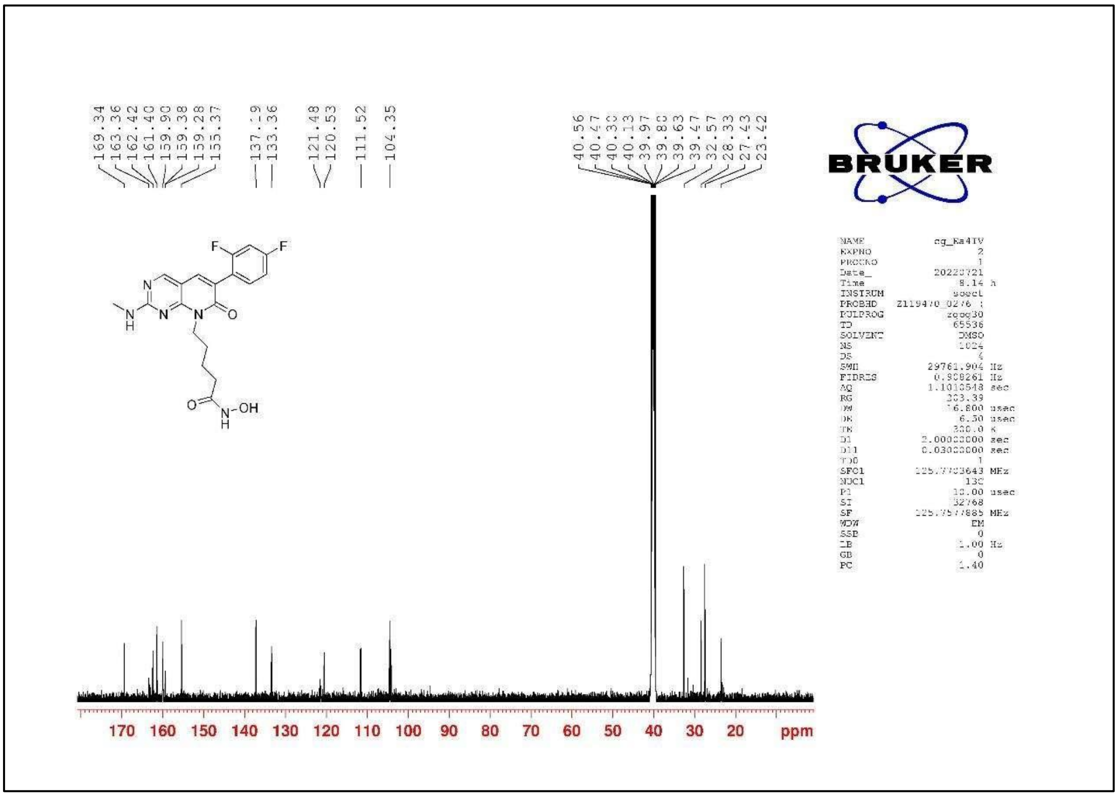

^{13}C-NMR spectrum of compound 20h

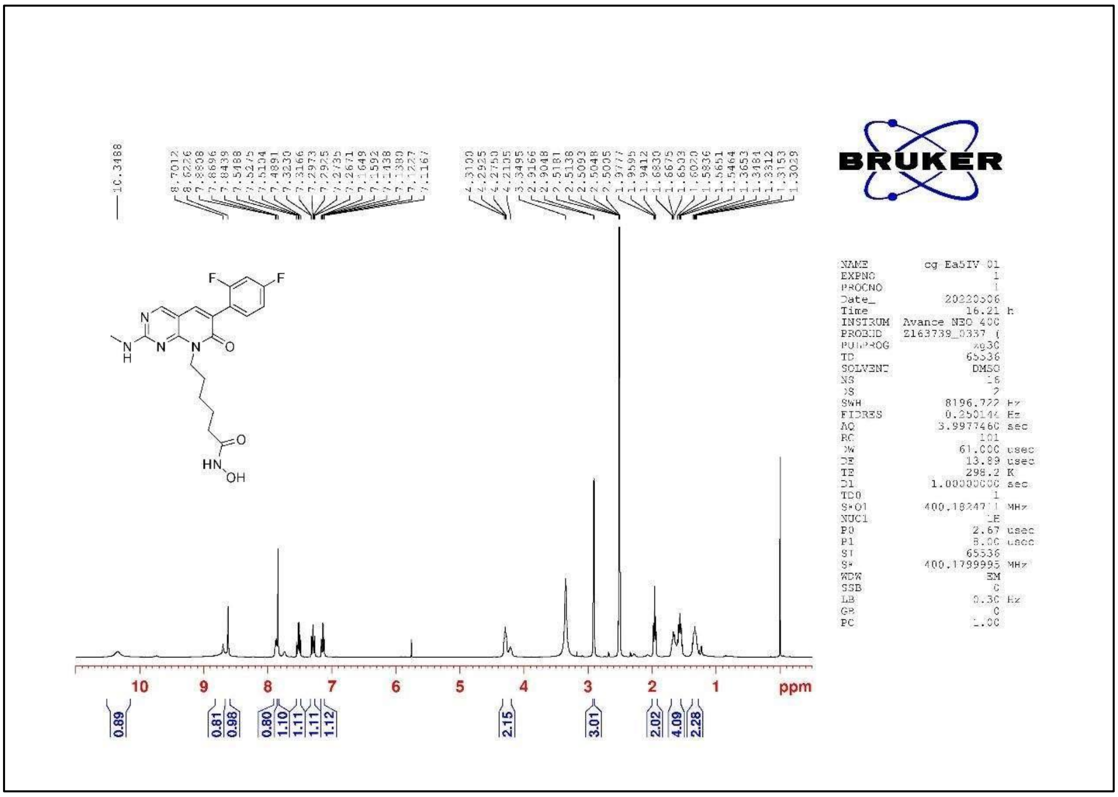

^{1}H-NMR spectrum of compound 20i

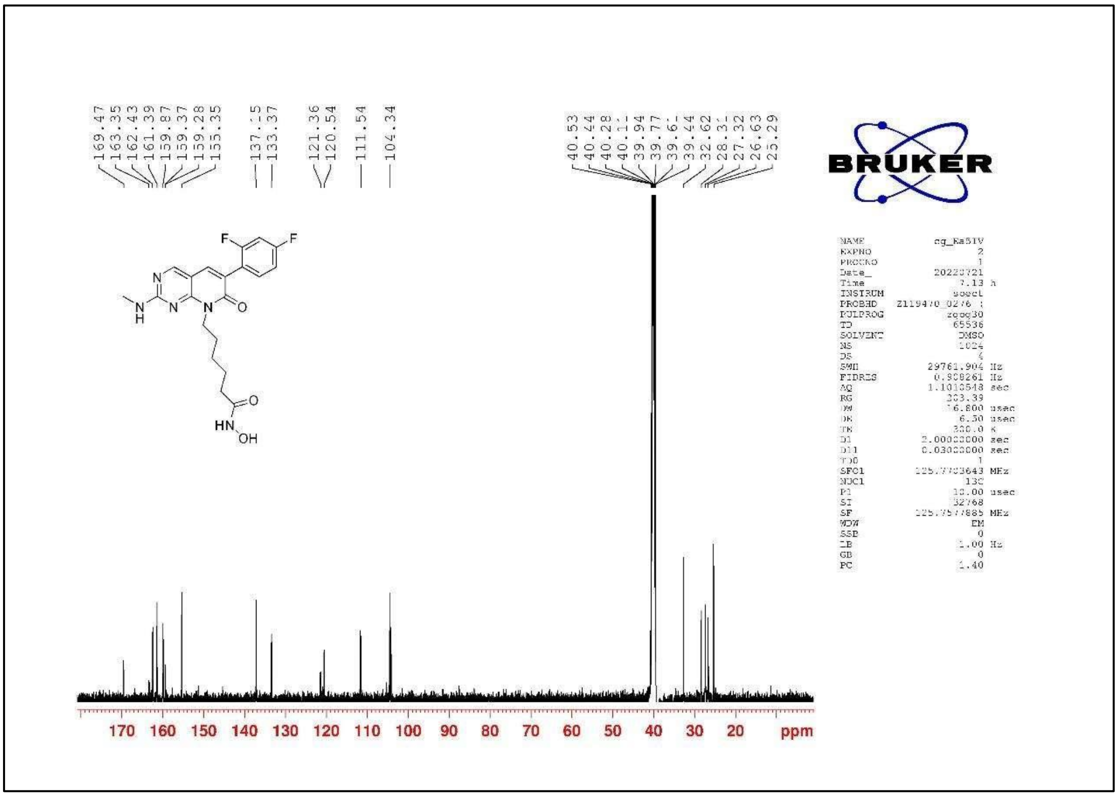

^{13}C-NMR spectrum of compound 20i

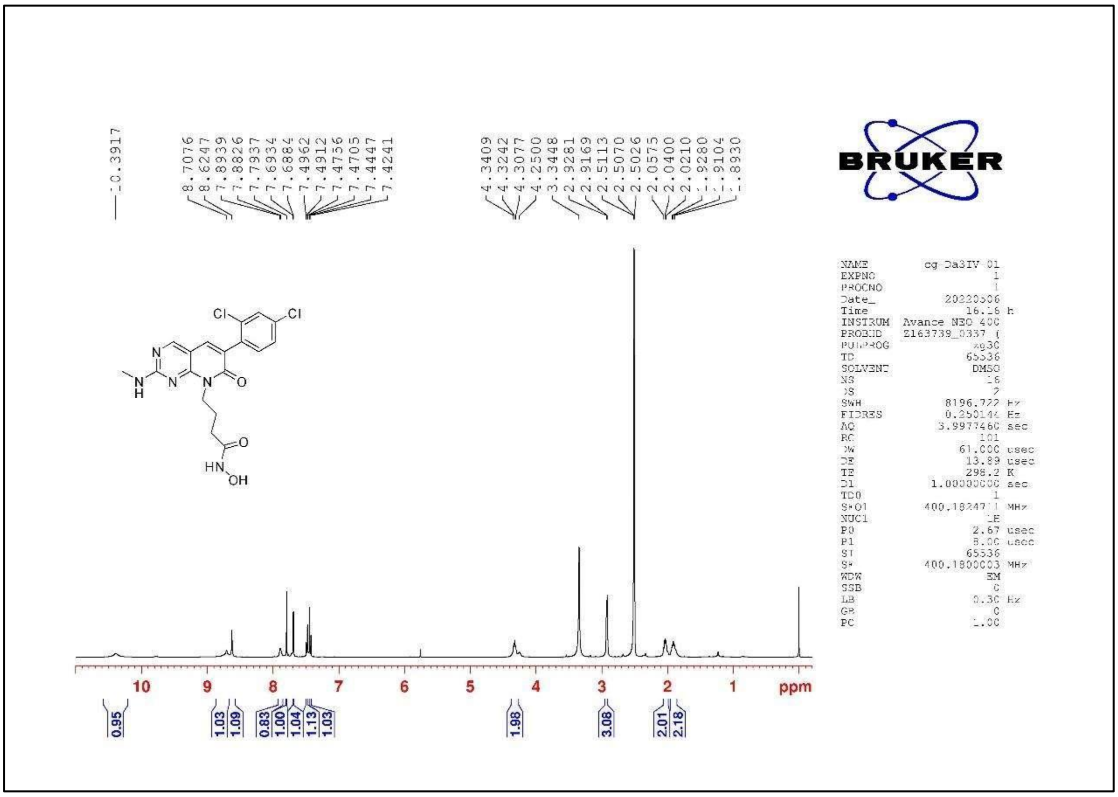

^{1}H-NMR spectrum of compound 20j

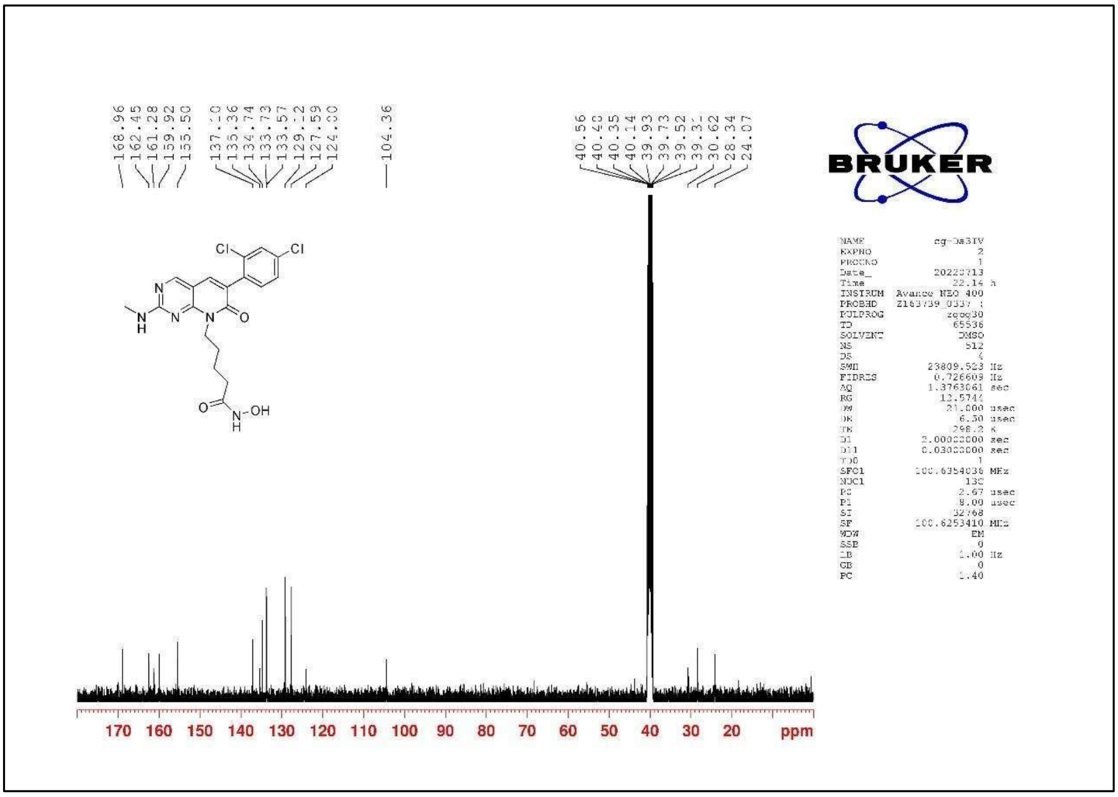

^{13}C-NMR spectrum of compound 20j

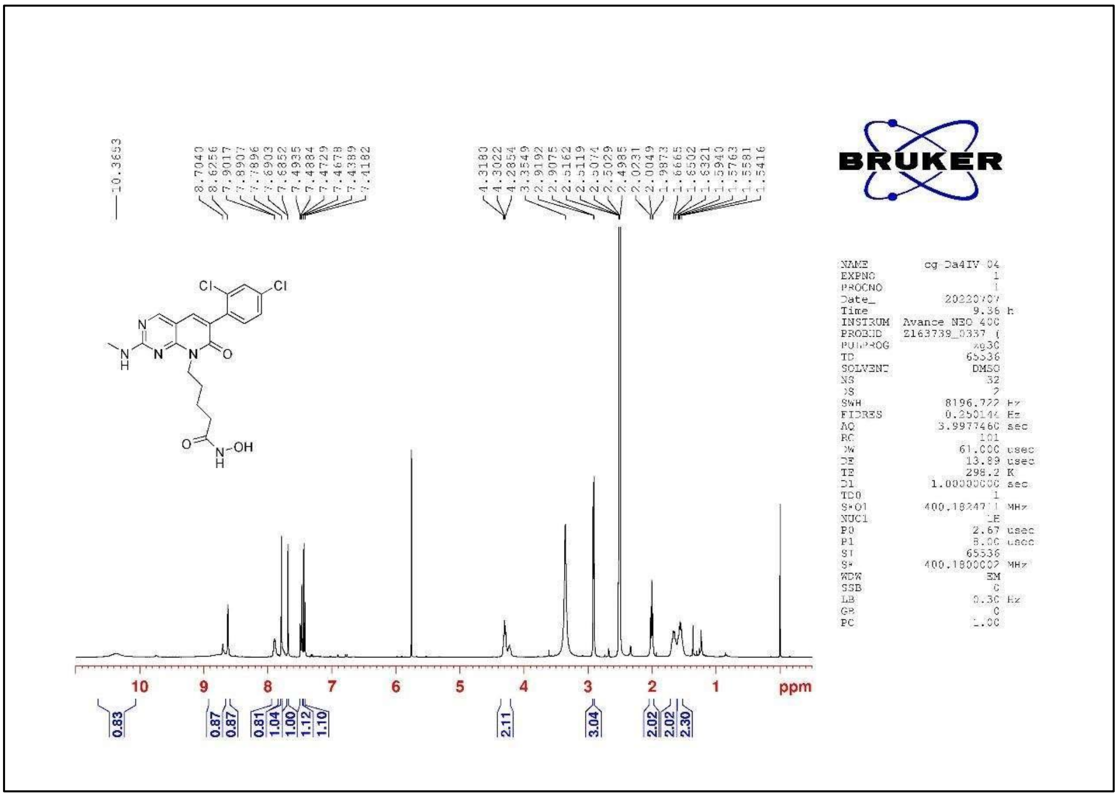

^{1}H-NMR spectrum of compound 20k

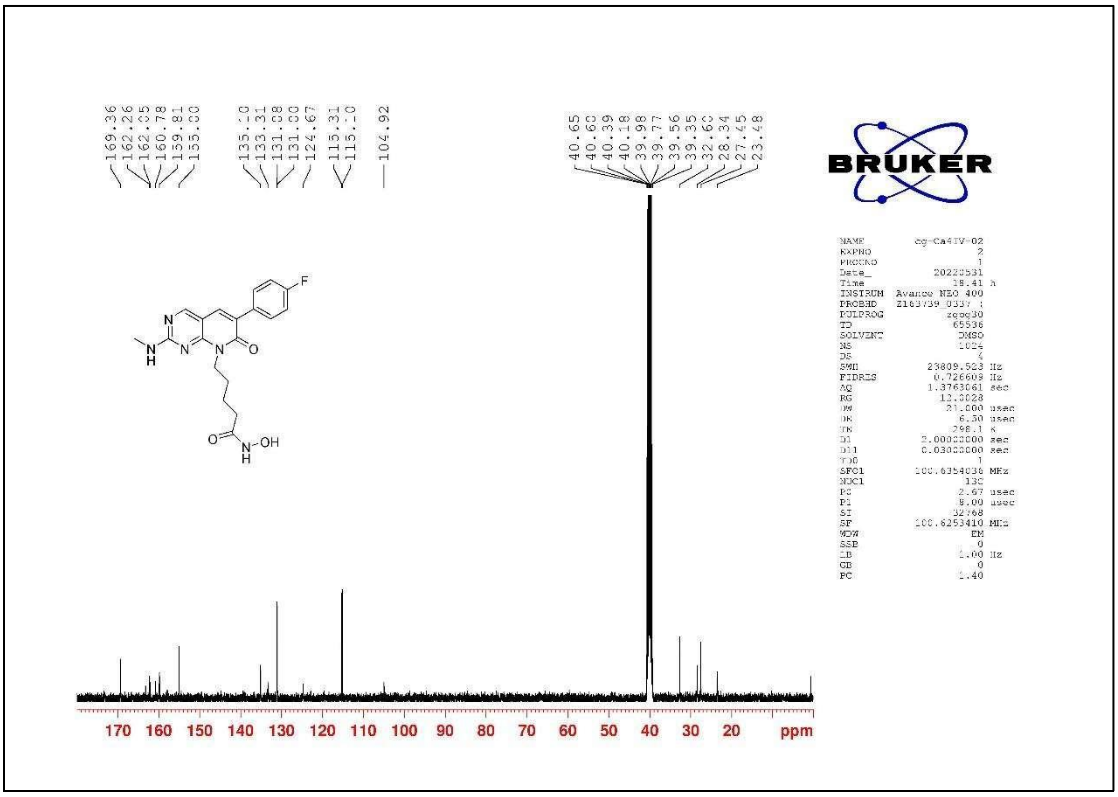

^{13}C-NMR spectrum of compound 20k

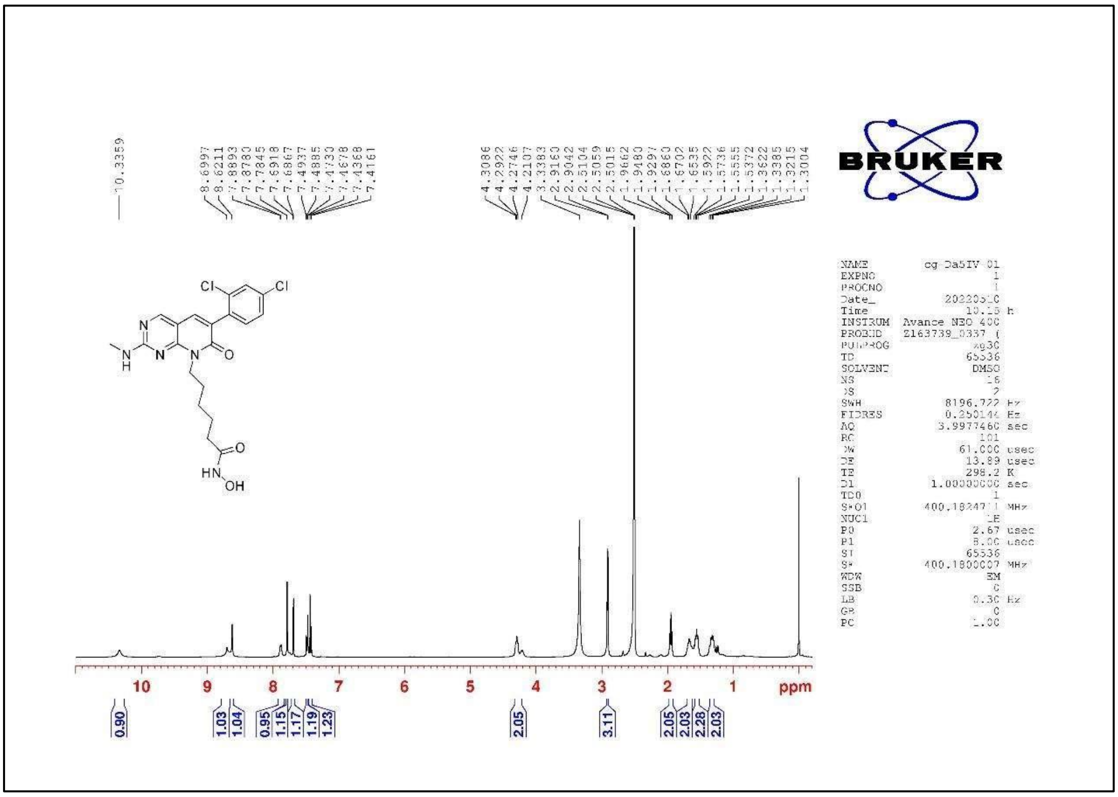

^{1}H-NMR spectrum of compound 20l

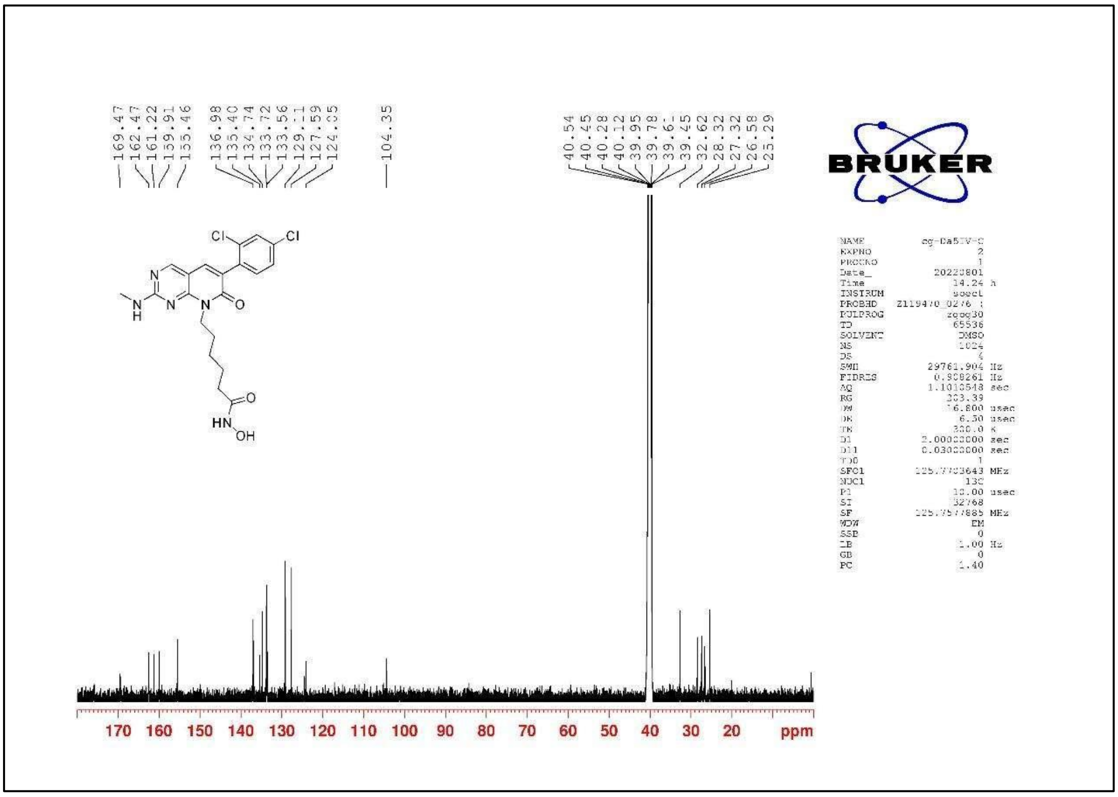

^{13}C-NMR spectrum of compound 20l

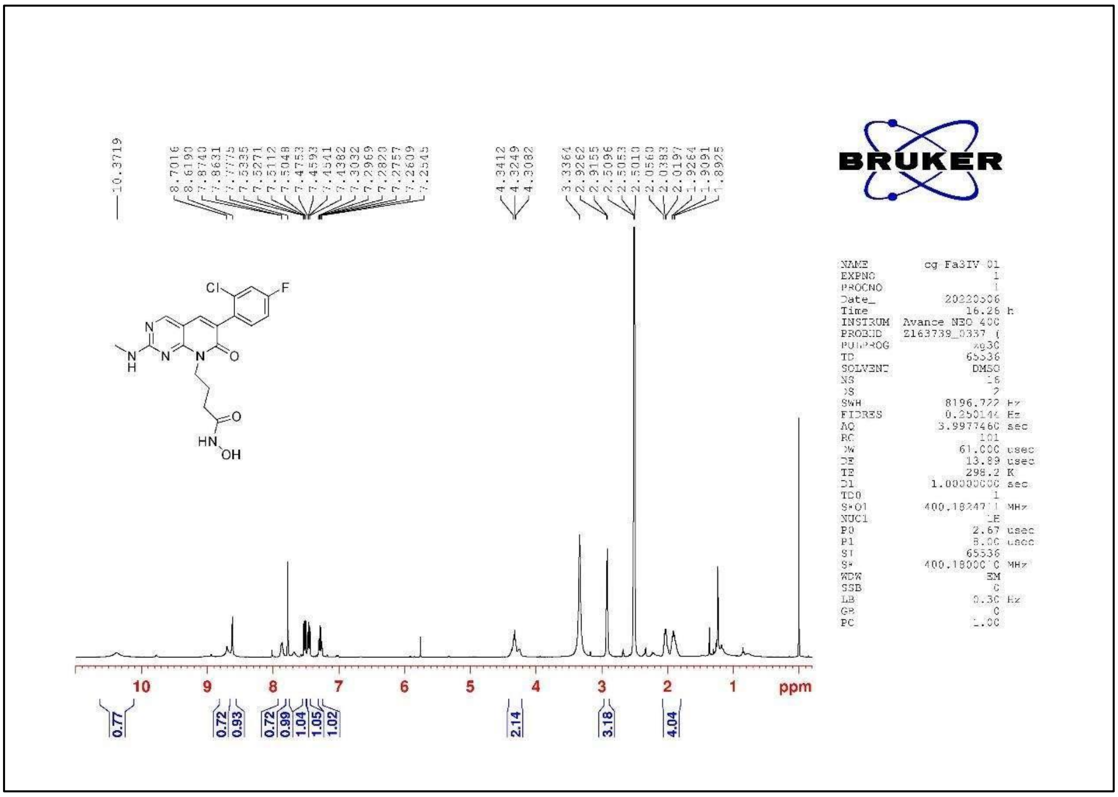

^{1}H-NMR spectrum of compound 20m

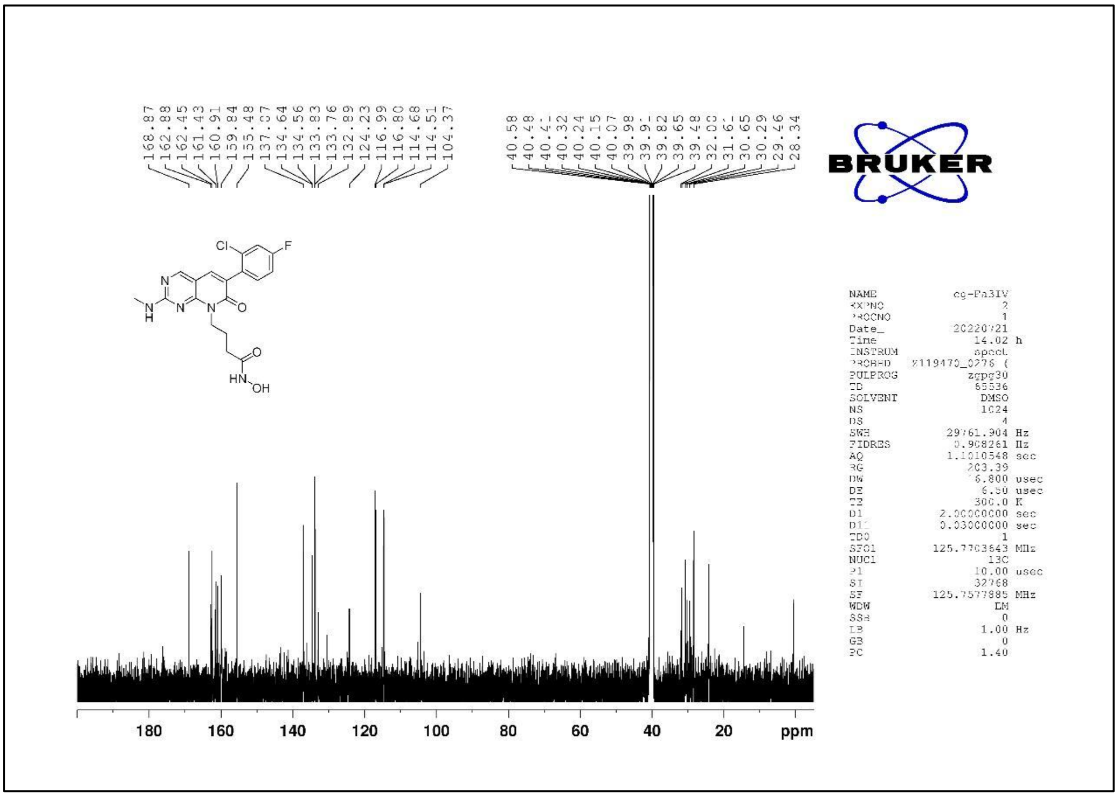

^{13}C-NMR spectrum of compound 20m

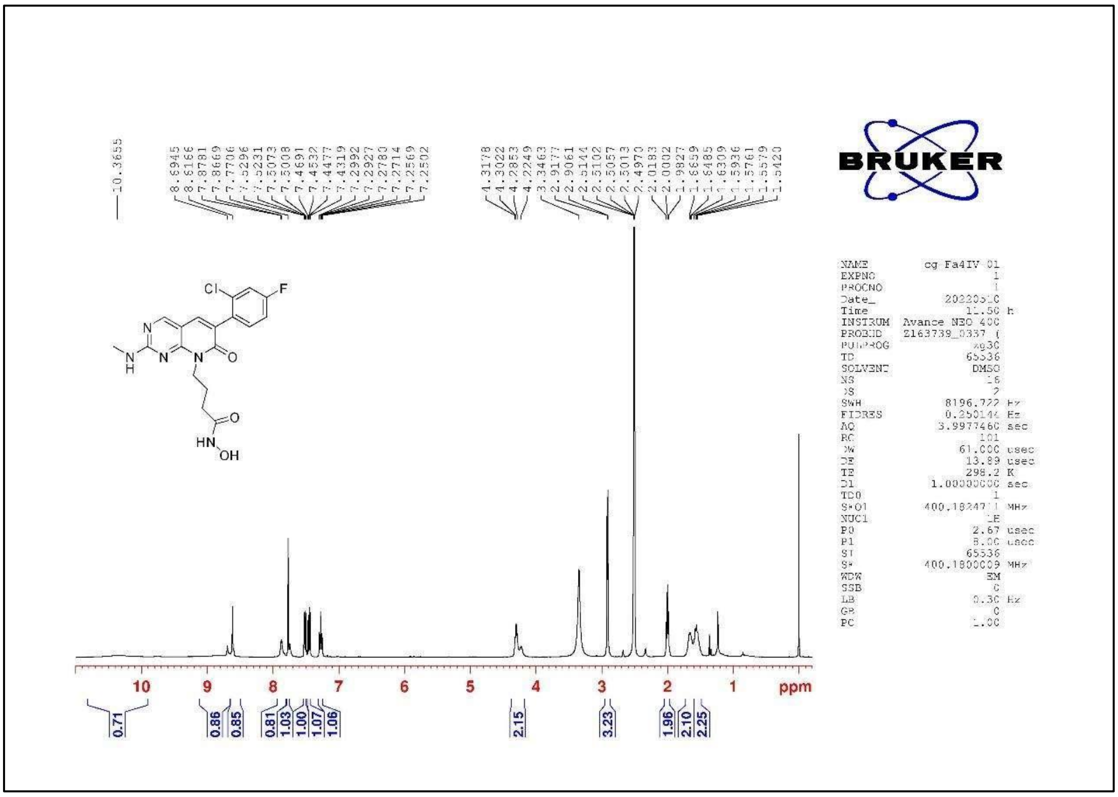

^{1}H-NMR spectrum of compound 20n

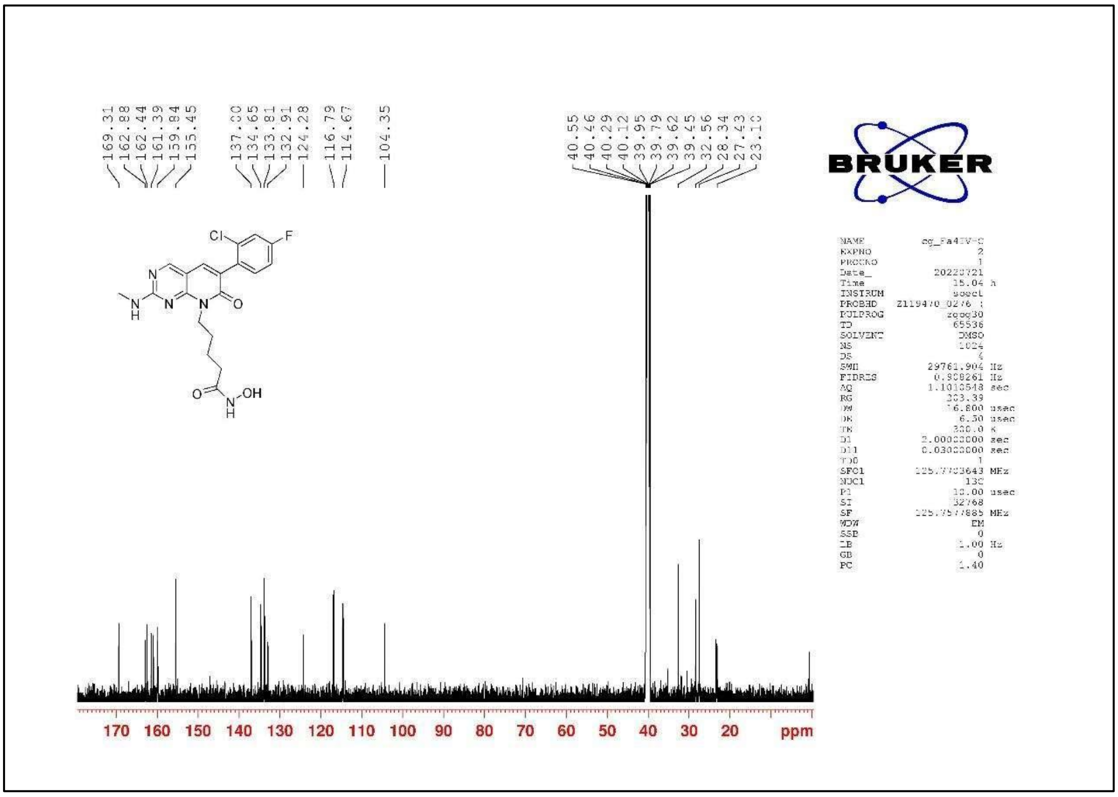

^{13}C-NMR spectrum of compound 20n

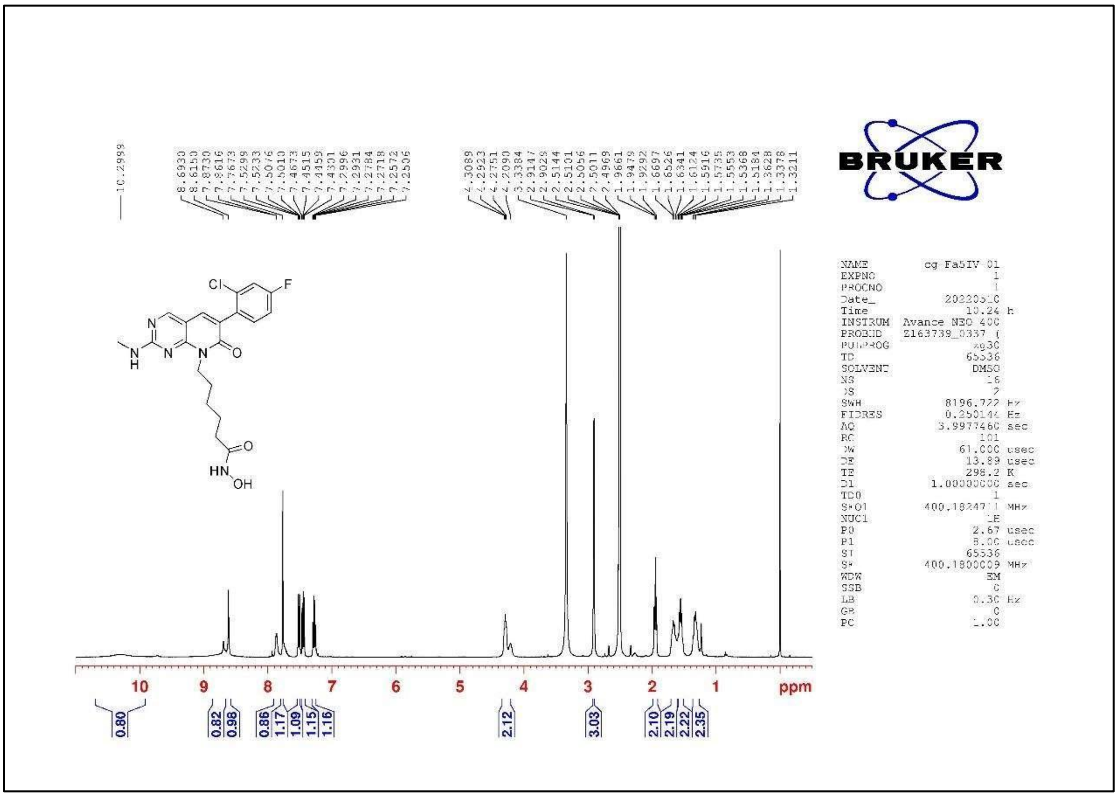

^{1}H-NMR spectrum of compound 20o

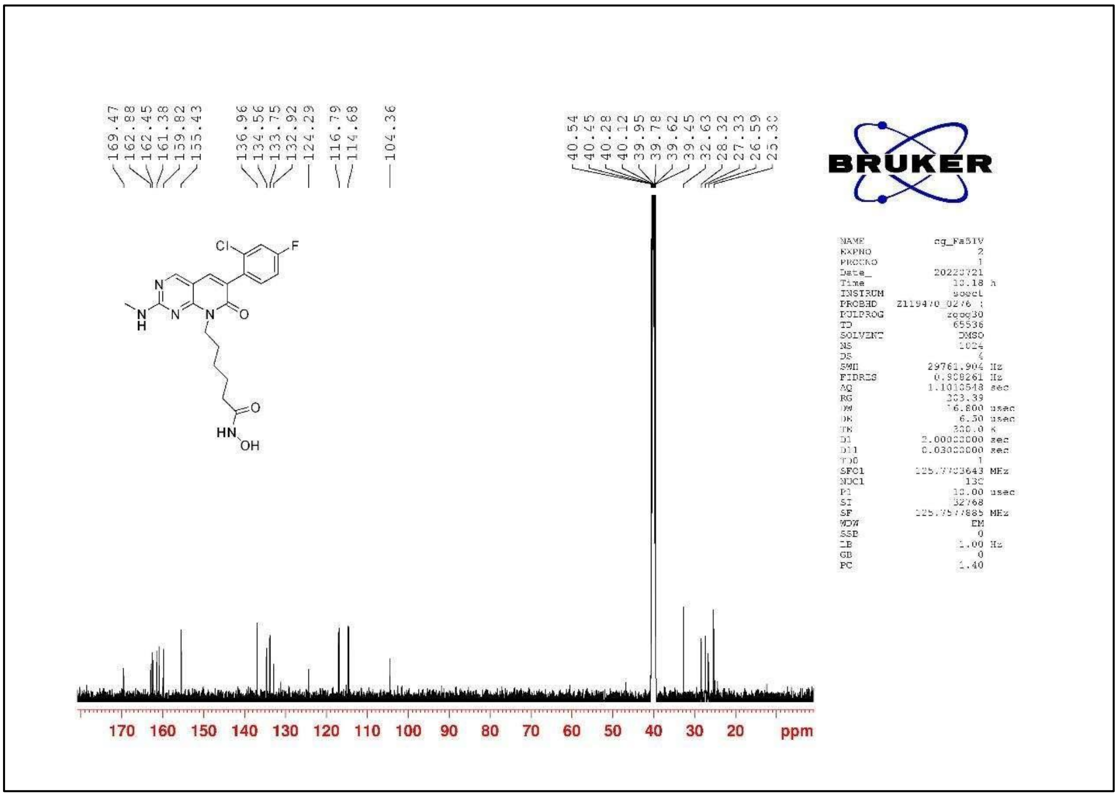

^{13}C-NMR spectrum of compound 20o

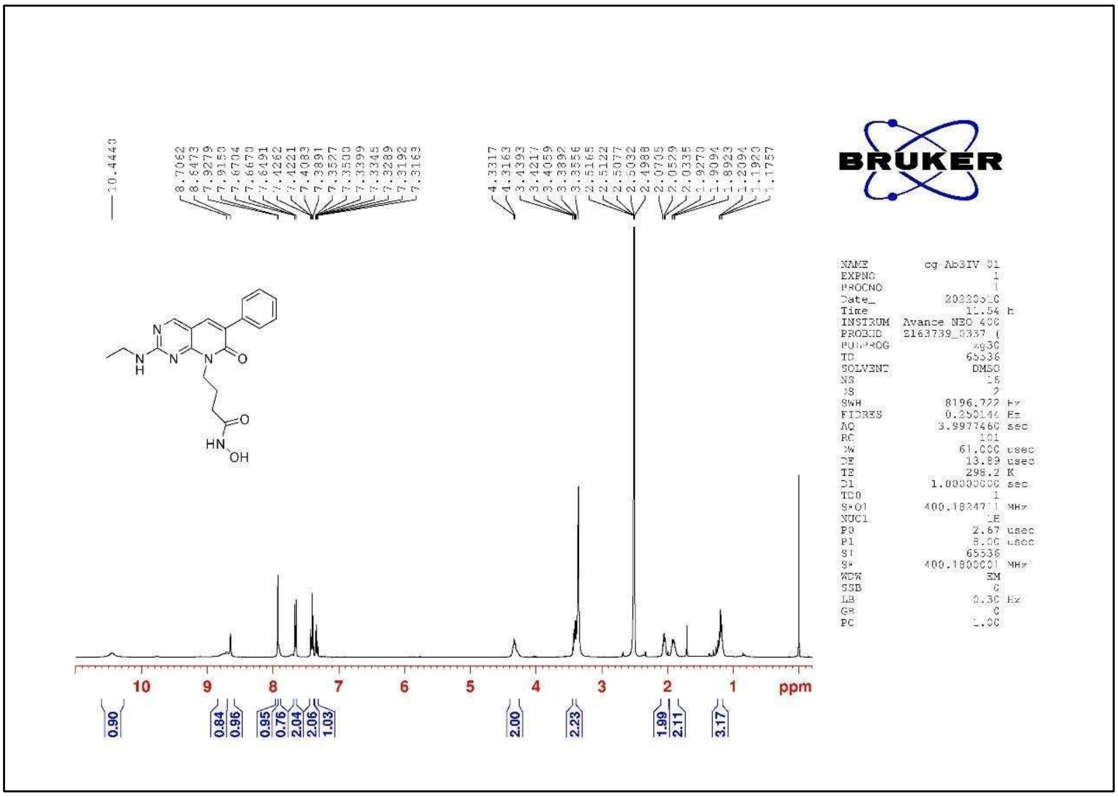

^{1}H-NMR spectrum of compound 21a

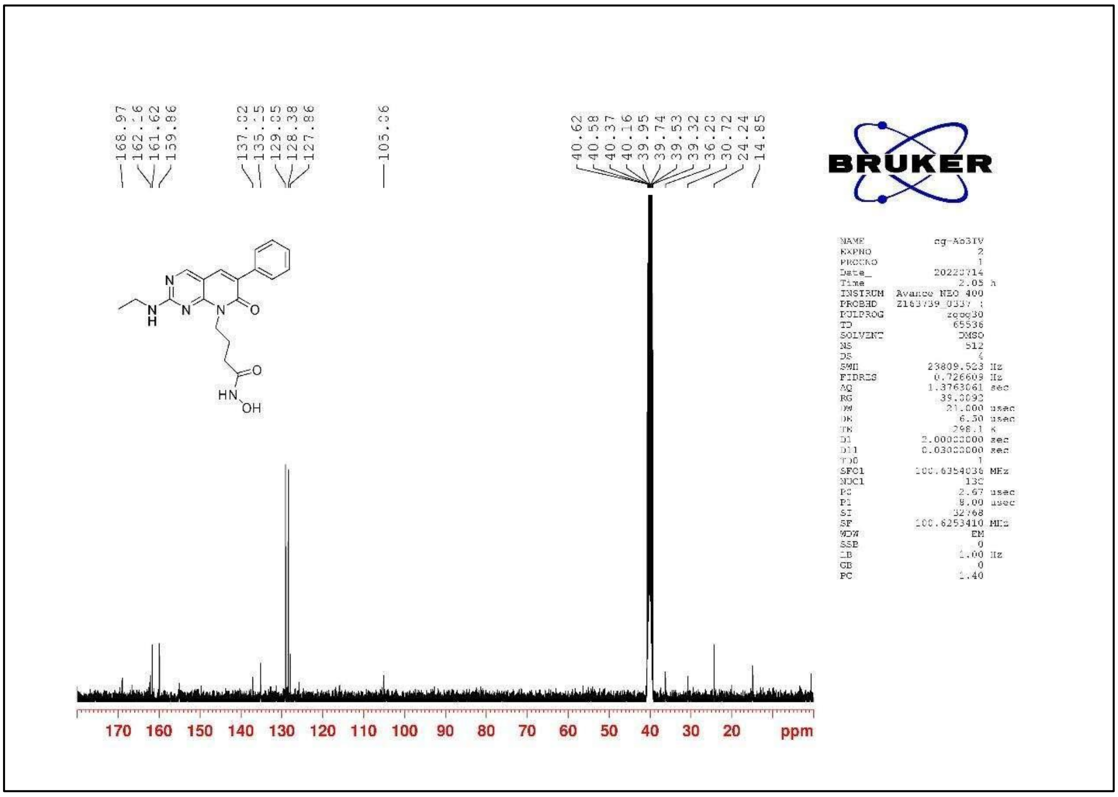

^{13}C-NMR spectrum of compound 21a

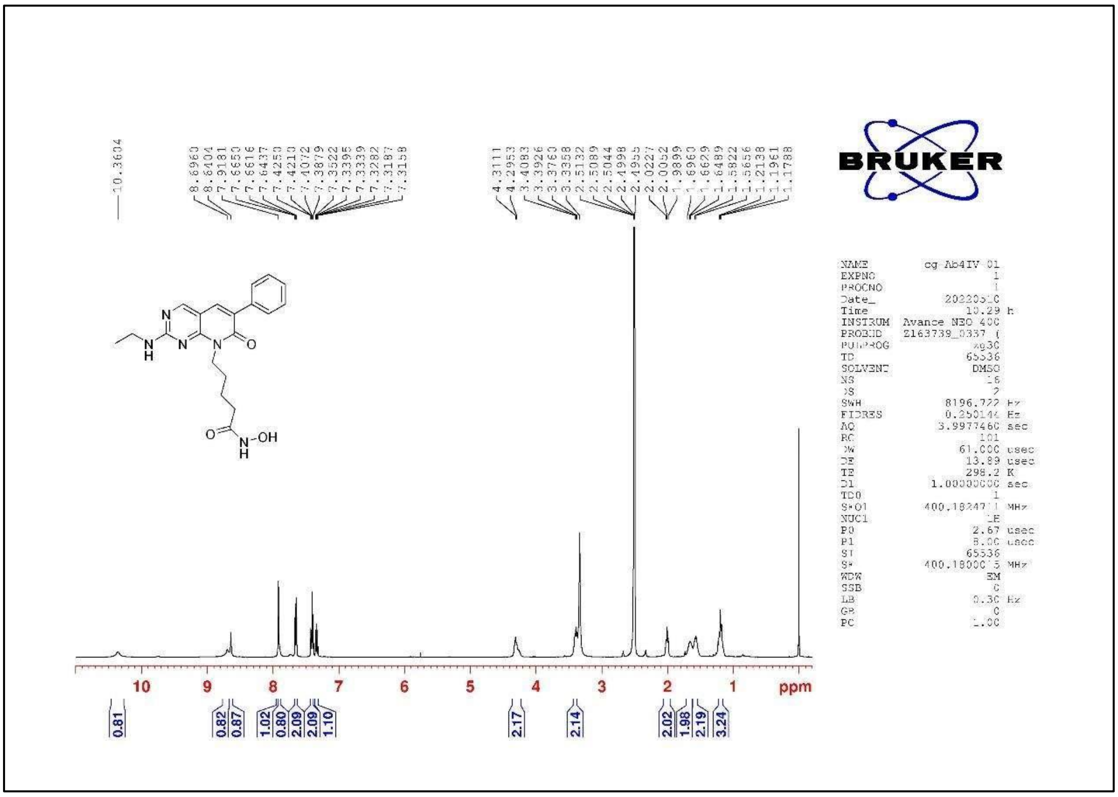

^{1}H-NMR spectrum of compound 21b

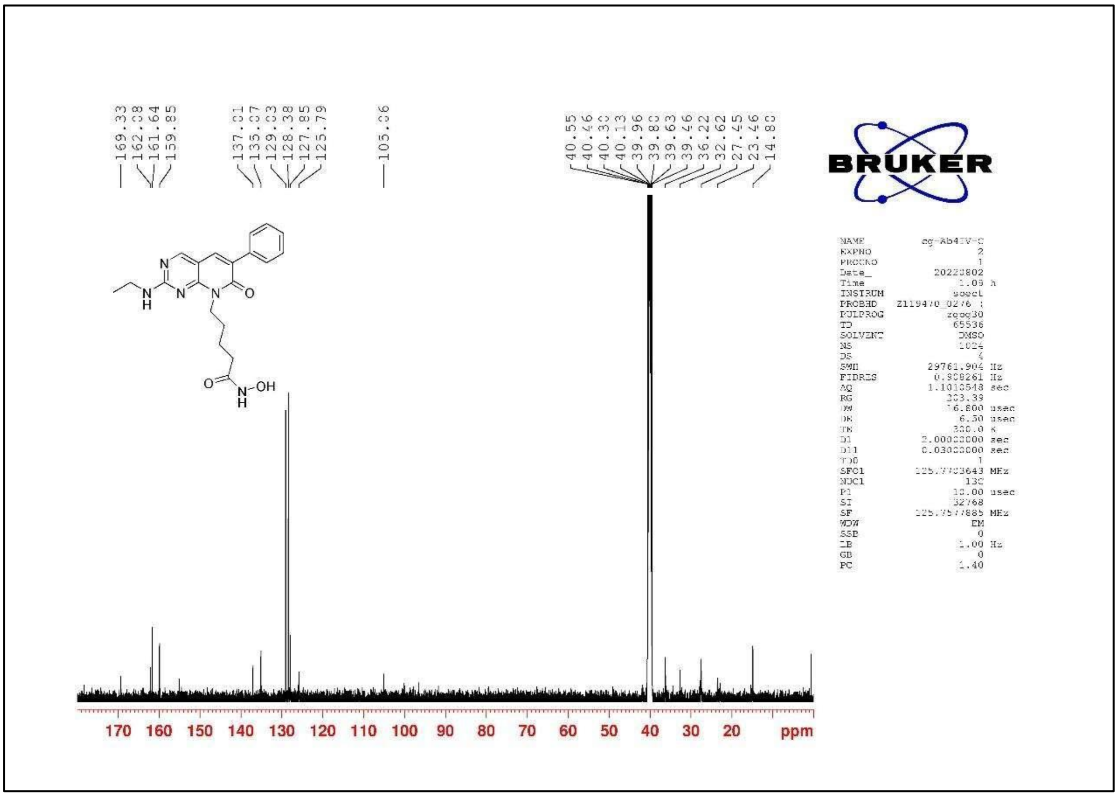

^{13}C-NMR spectrum of compound 21b

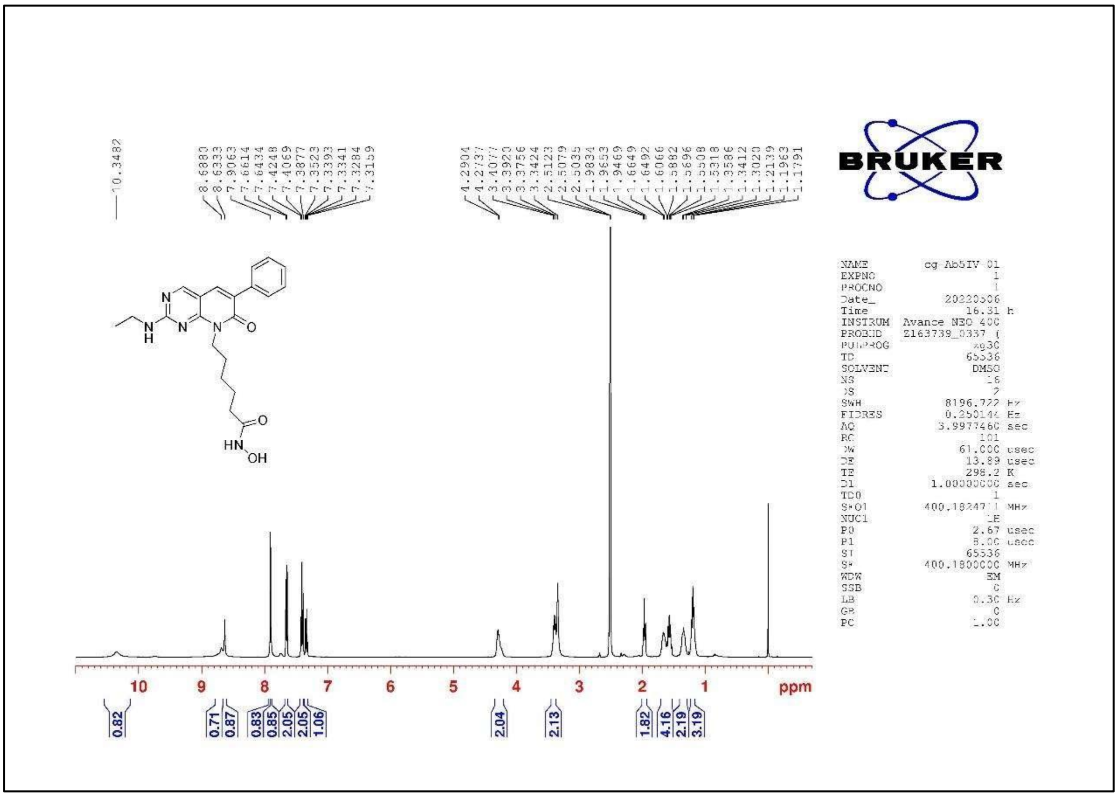

^{1}H-NMR spectrum of compound 21c

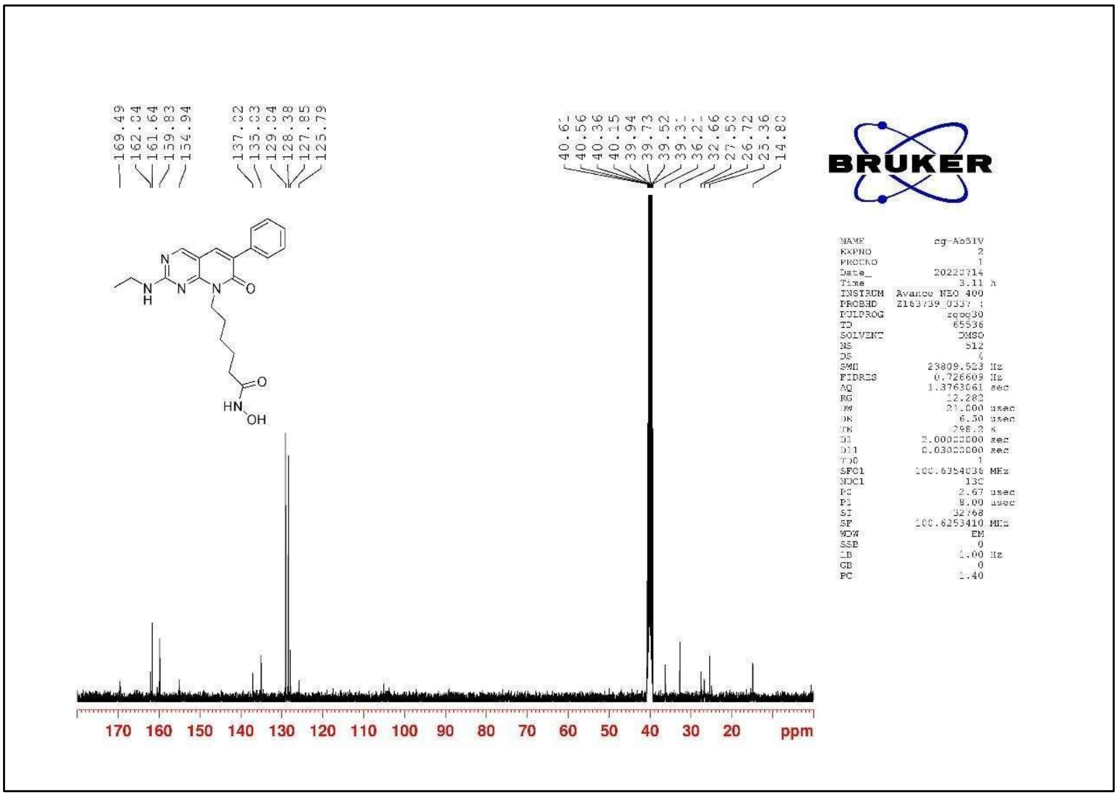

^{13}C-NMR spectrum of compound 21c

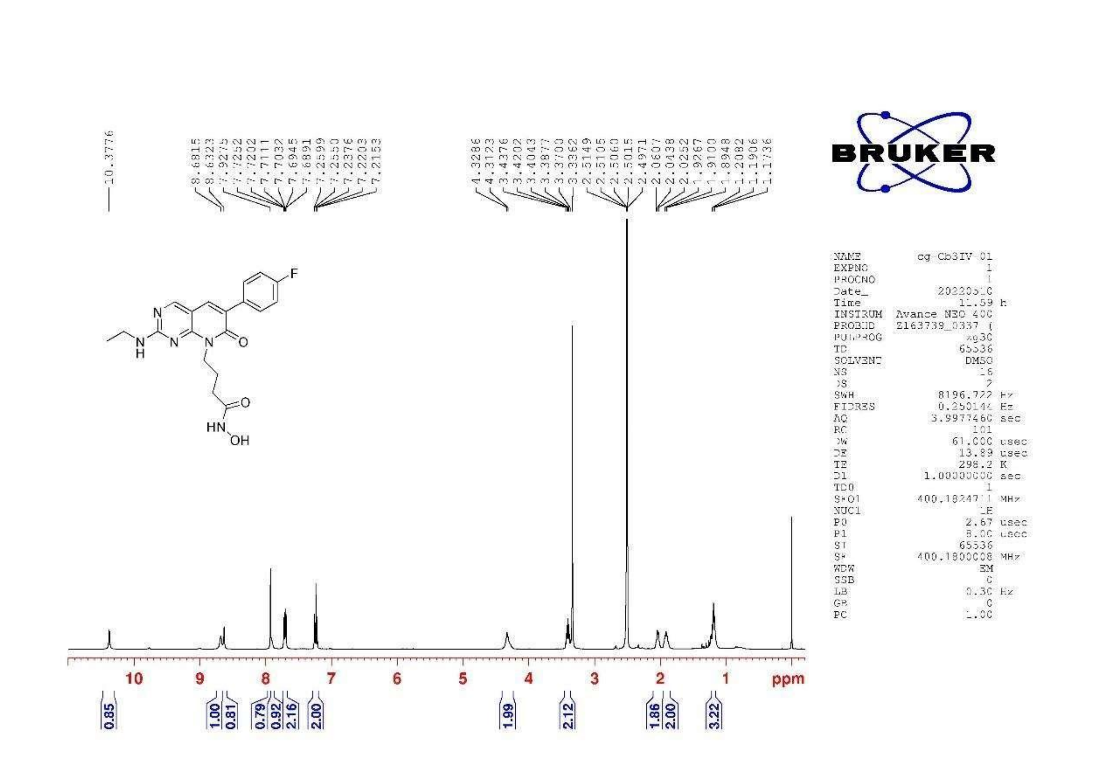

^{1}H-NMR spectrum of compound 21d

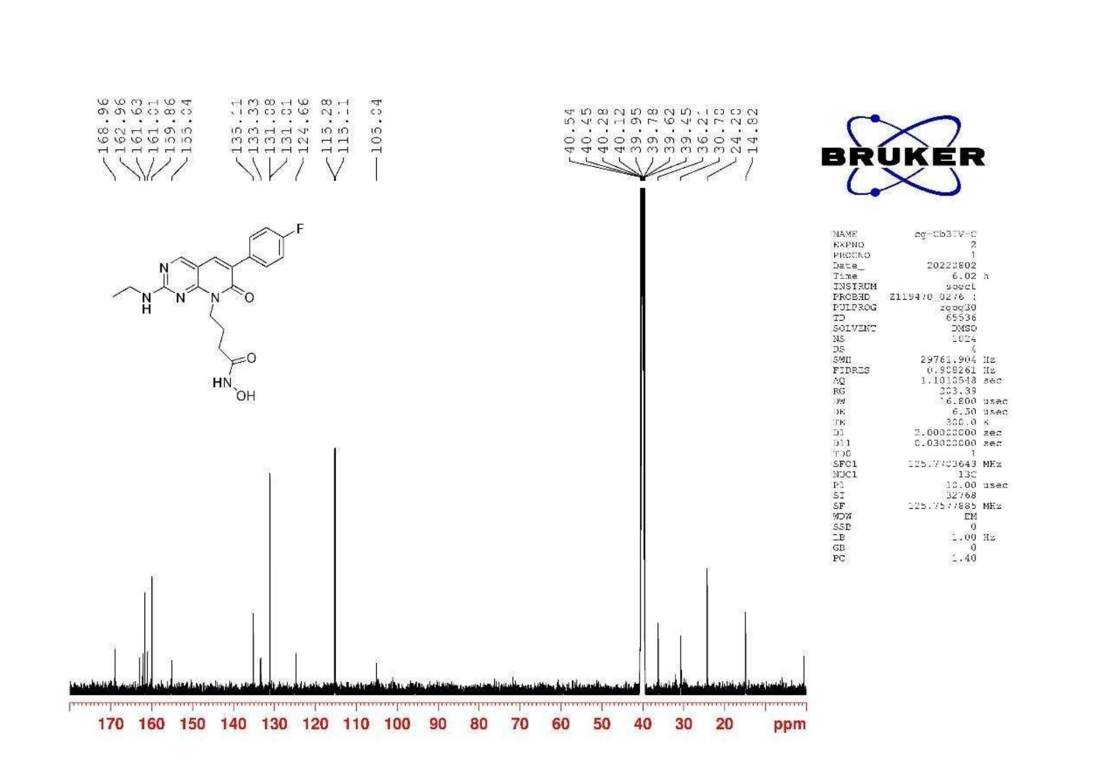

^{13}C-NMR spectrum of compound 21d

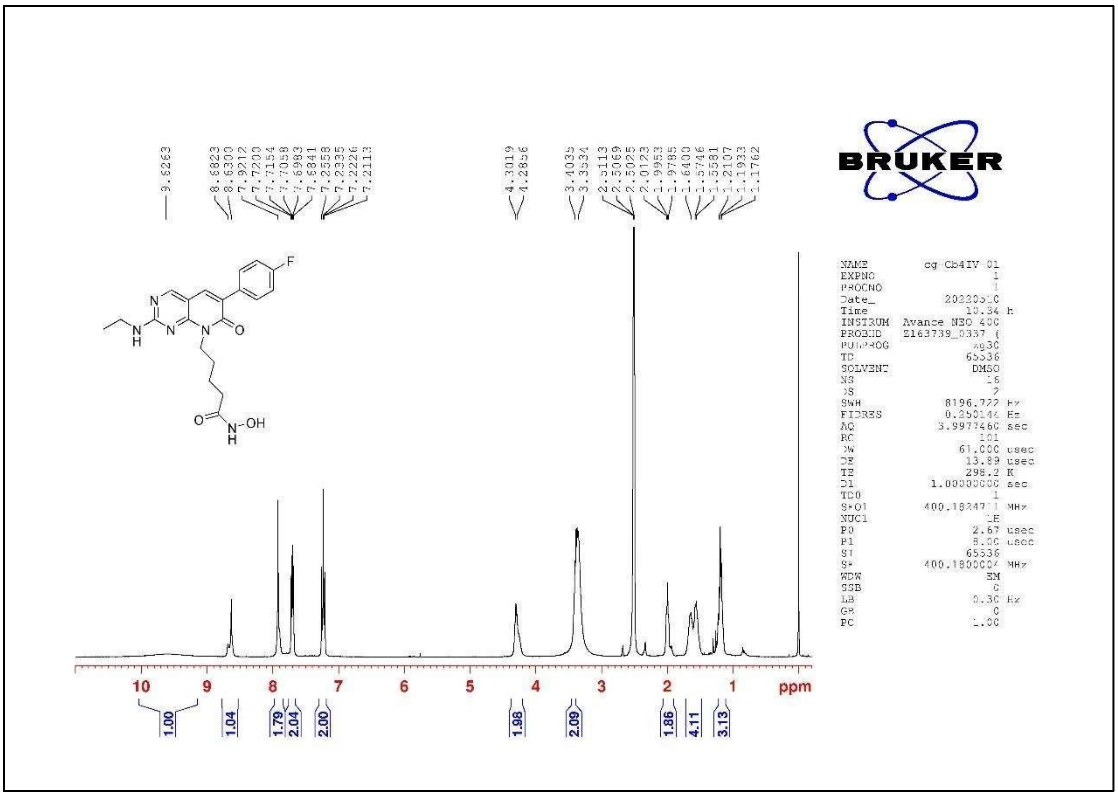

^{1}H-NMR spectrum of compound 21e

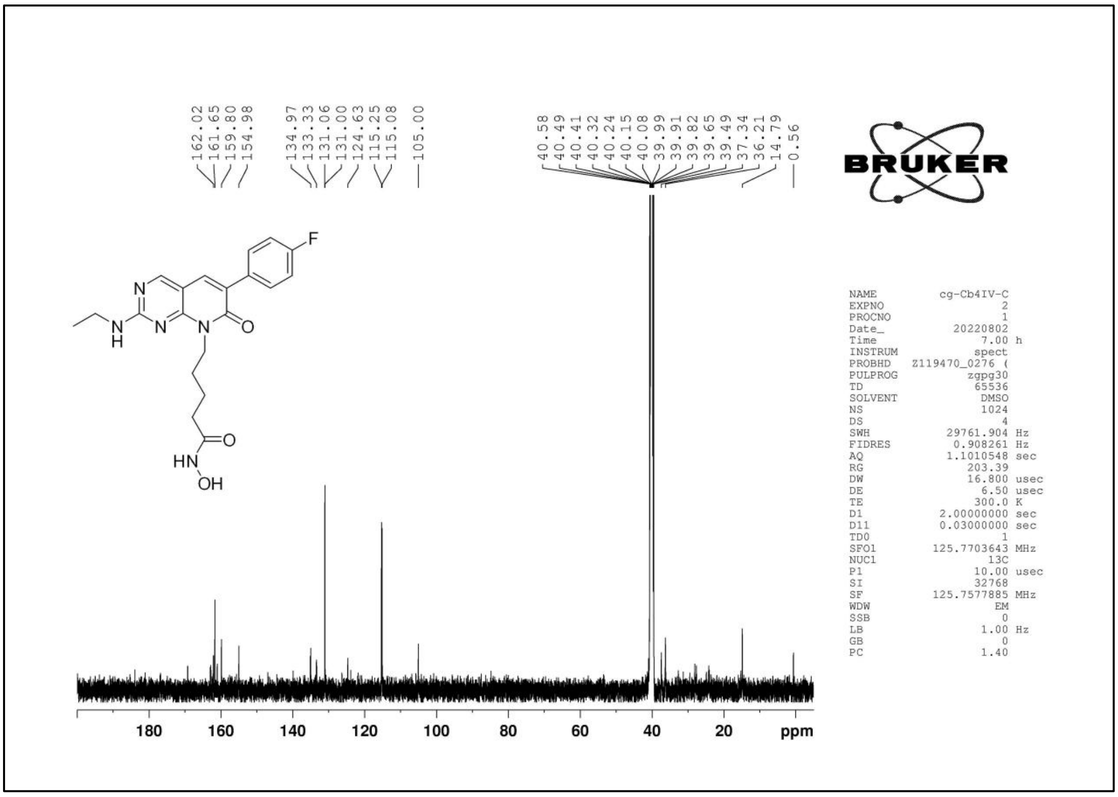

^{13}C-NMR spectrum of compound 21e

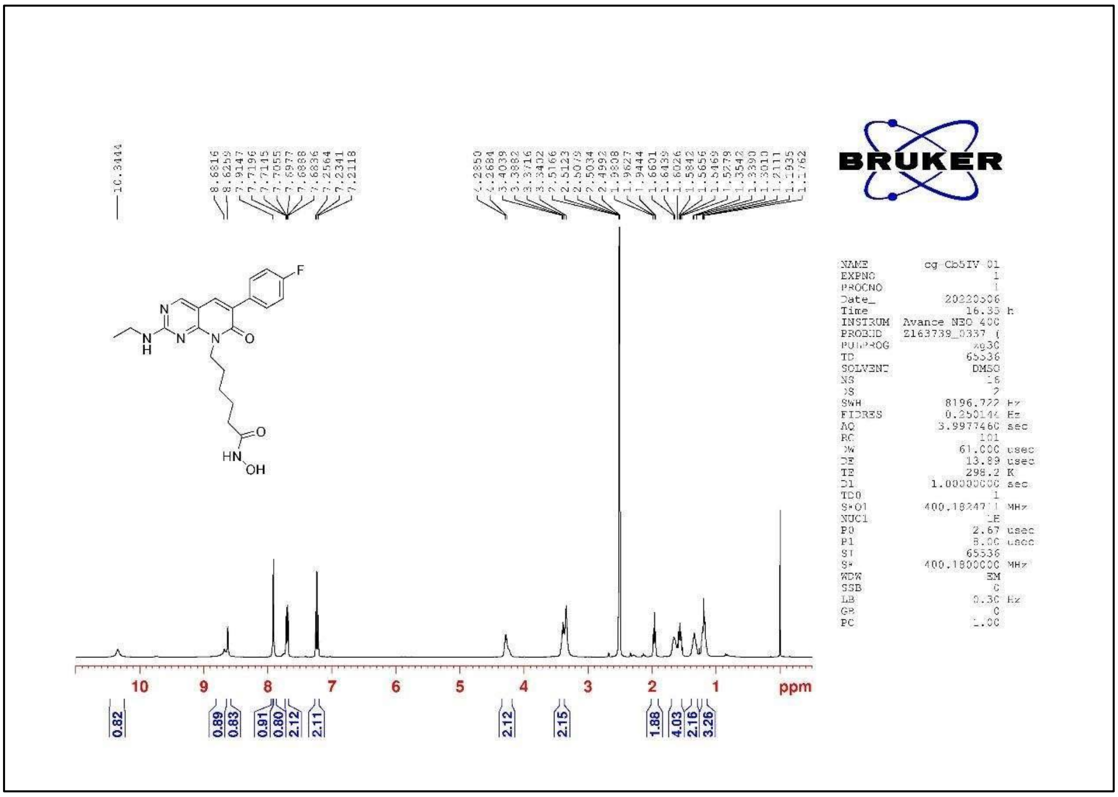

^{1}H-NMR spectrum of compound 21f

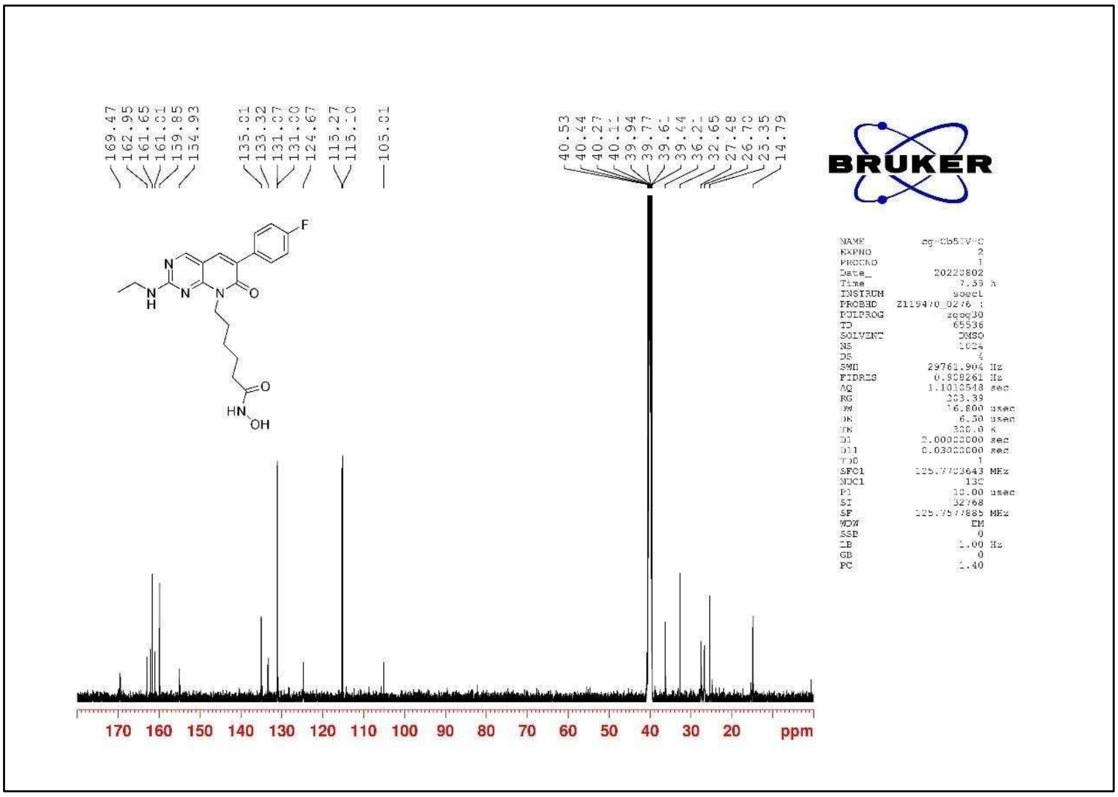

^{13}C-NMR spectrum of compound 21f

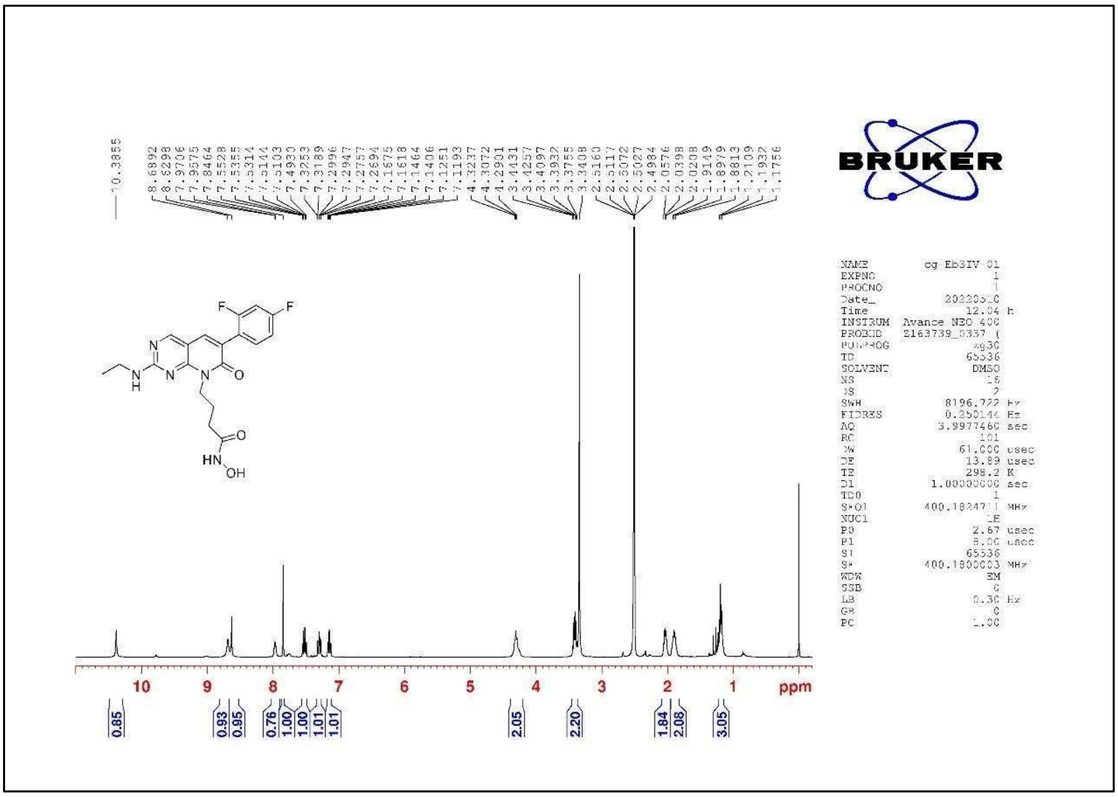

^{1}H-NMR spectrum of compound 21g

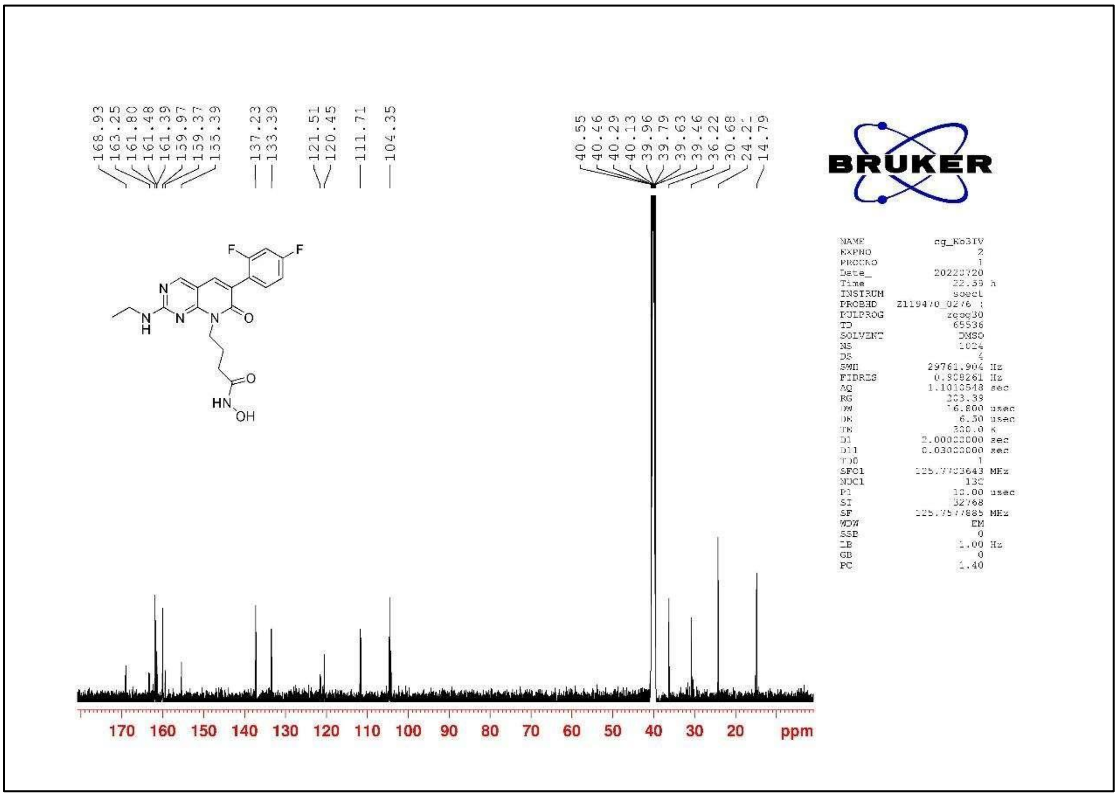

^{13}C-NMR spectrum of compound 21g

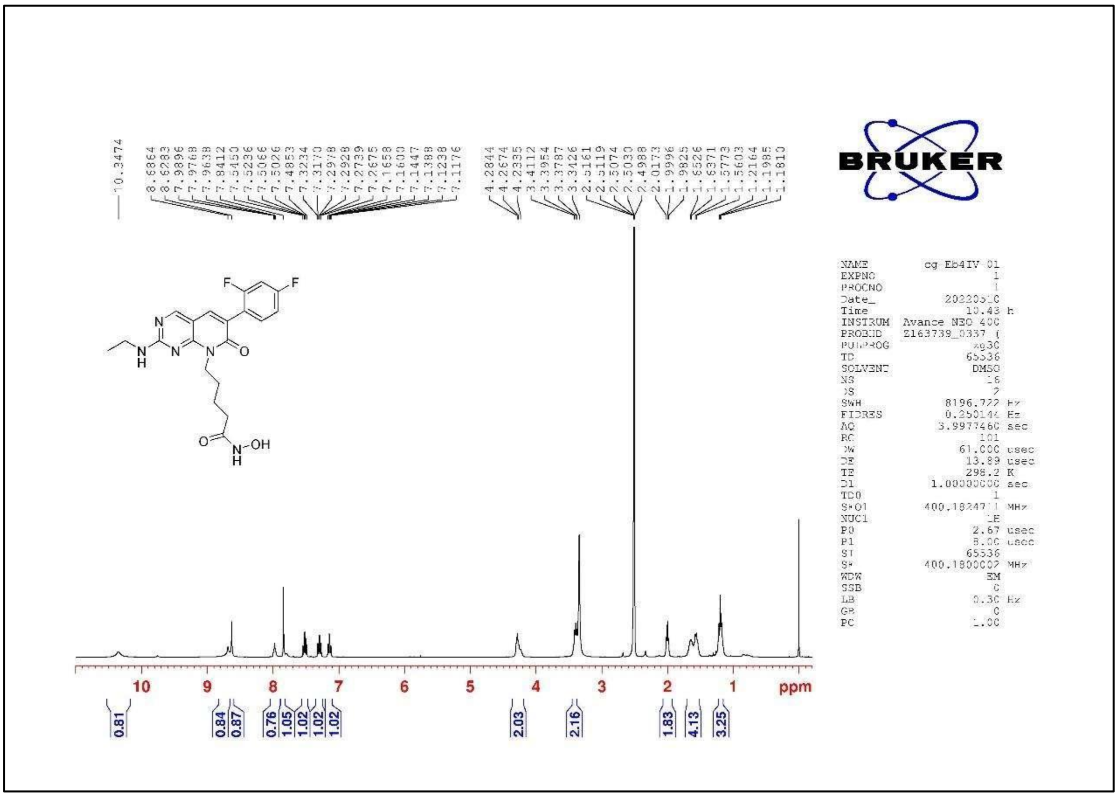

^{1}H-NMR spectrum of compound 21h

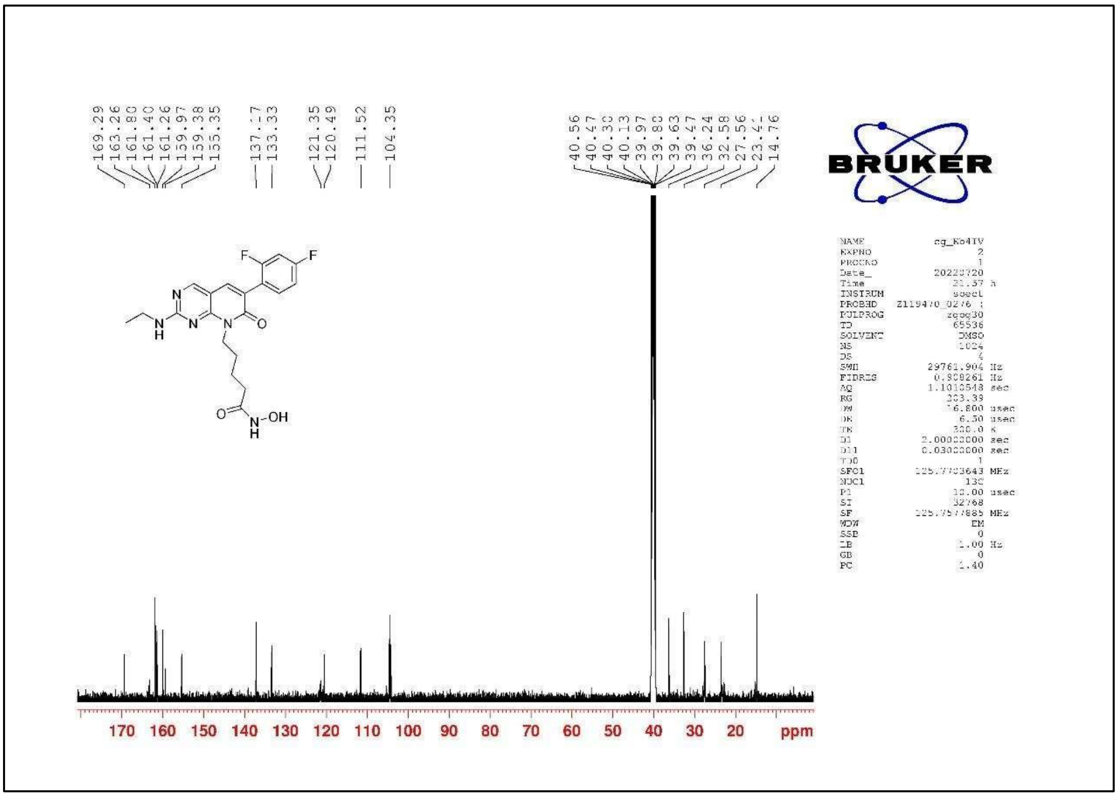

^{13}C-NMR spectrum of compound 21h

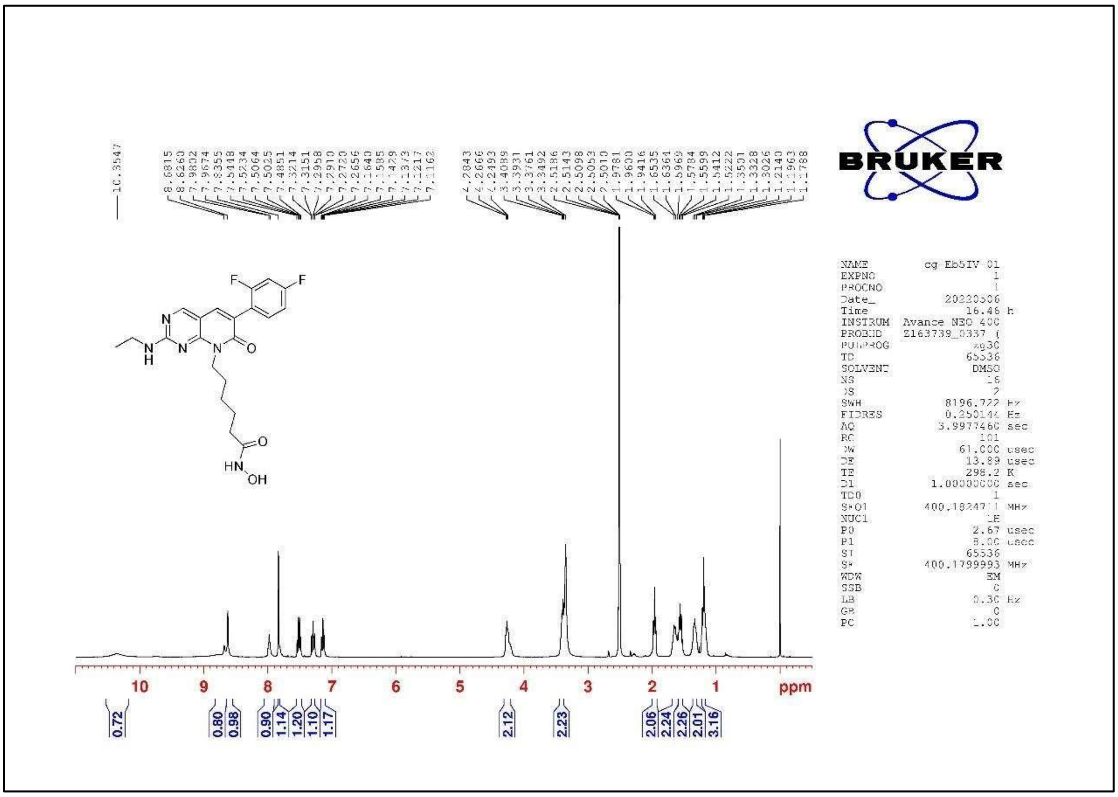

^{1}H-NMR spectrum of compound 21i

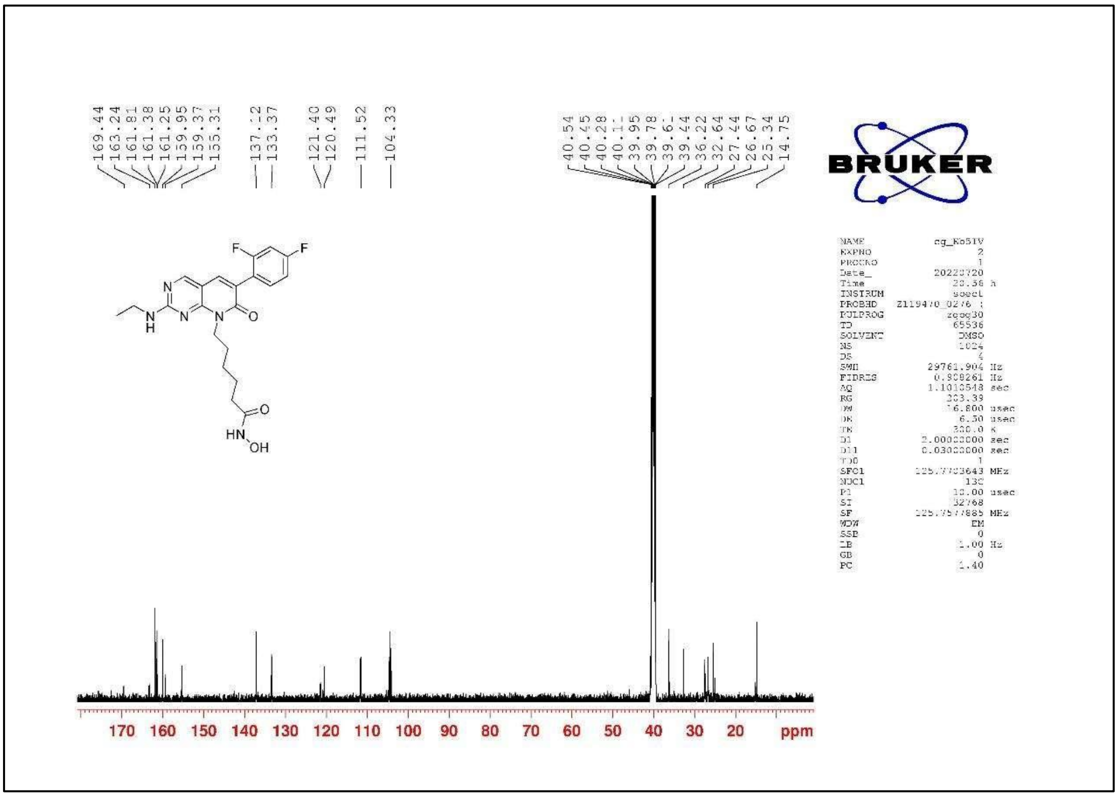

^{13}C-NMR spectrum of compound 21i

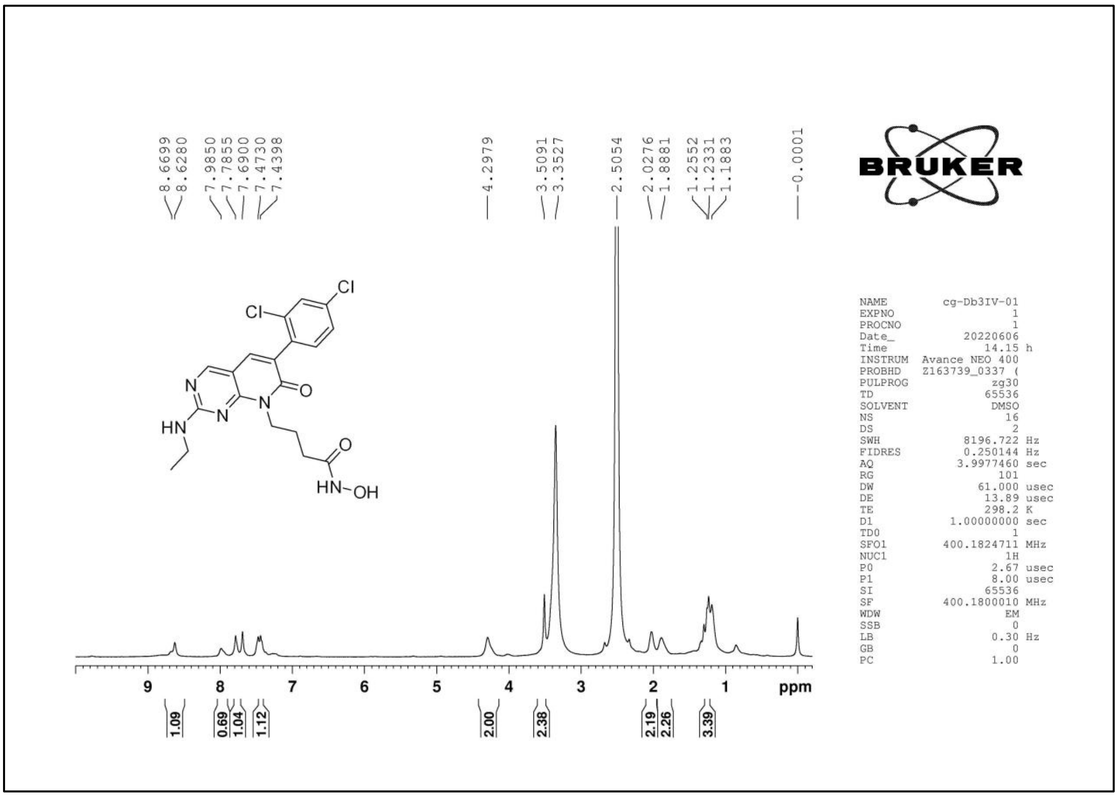

^{1}H-NMR spectrum of compound 21j

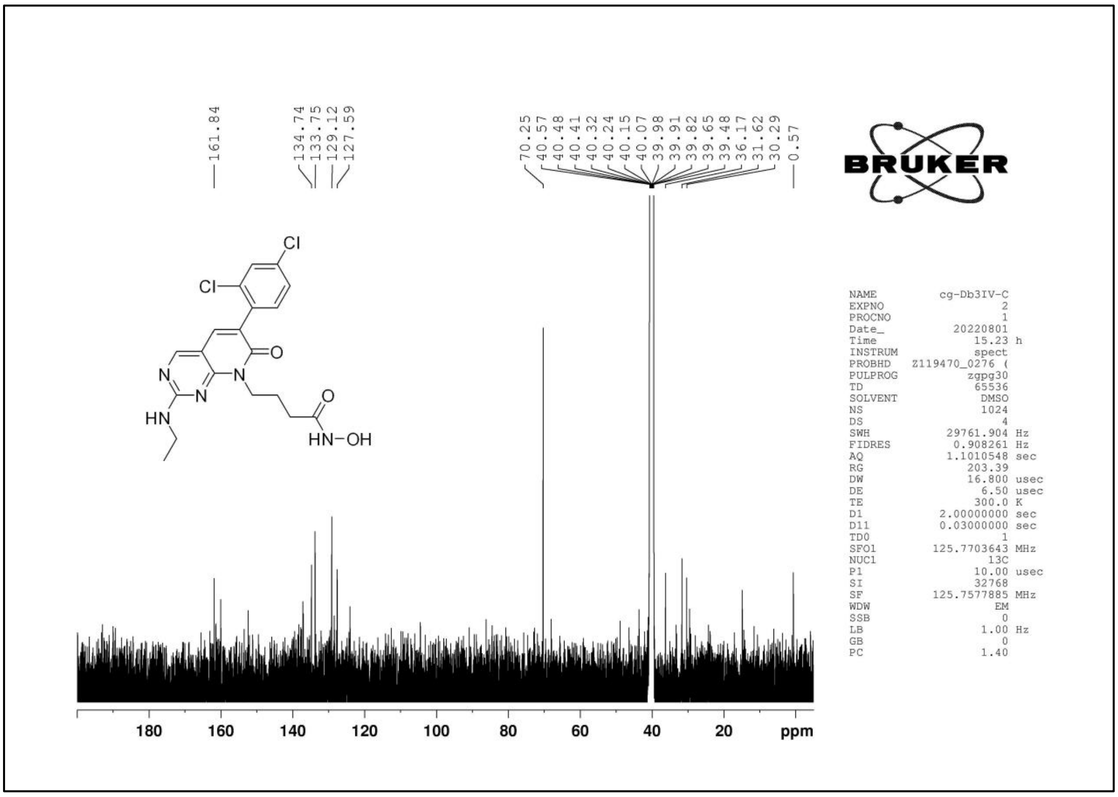

^{13}C-NMR spectrum of compound 21j

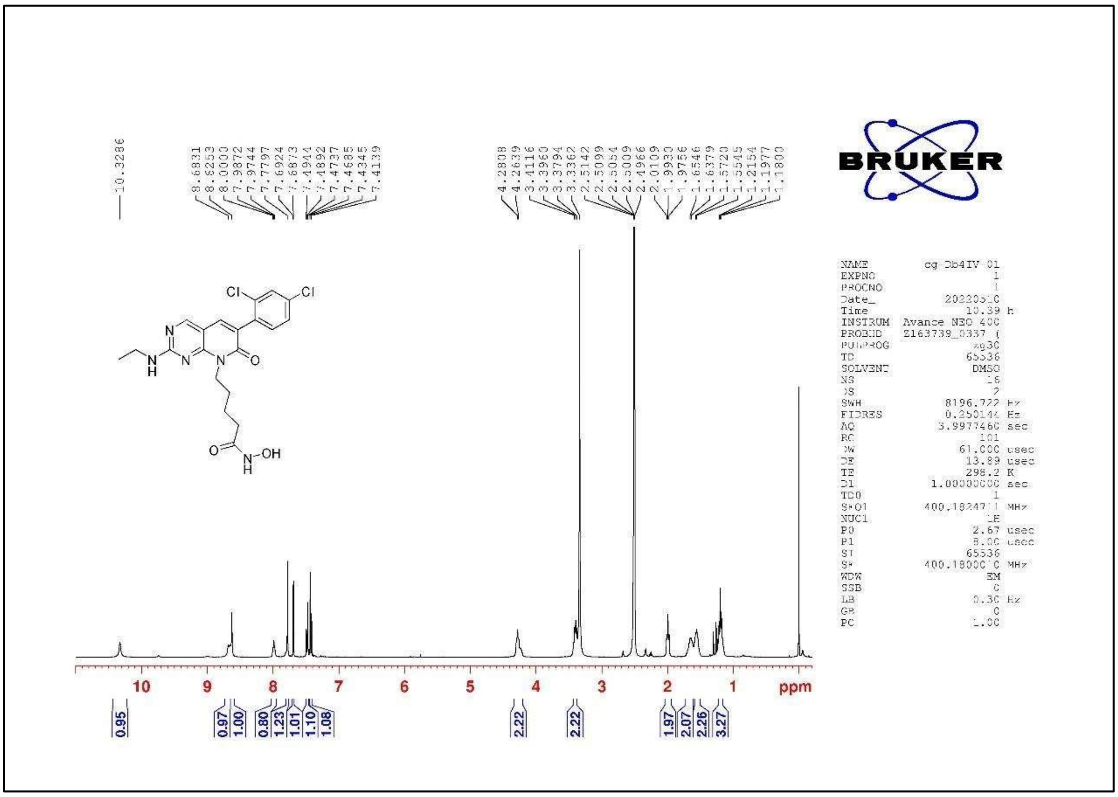

^{1}H-NMR spectrum of compound 21k

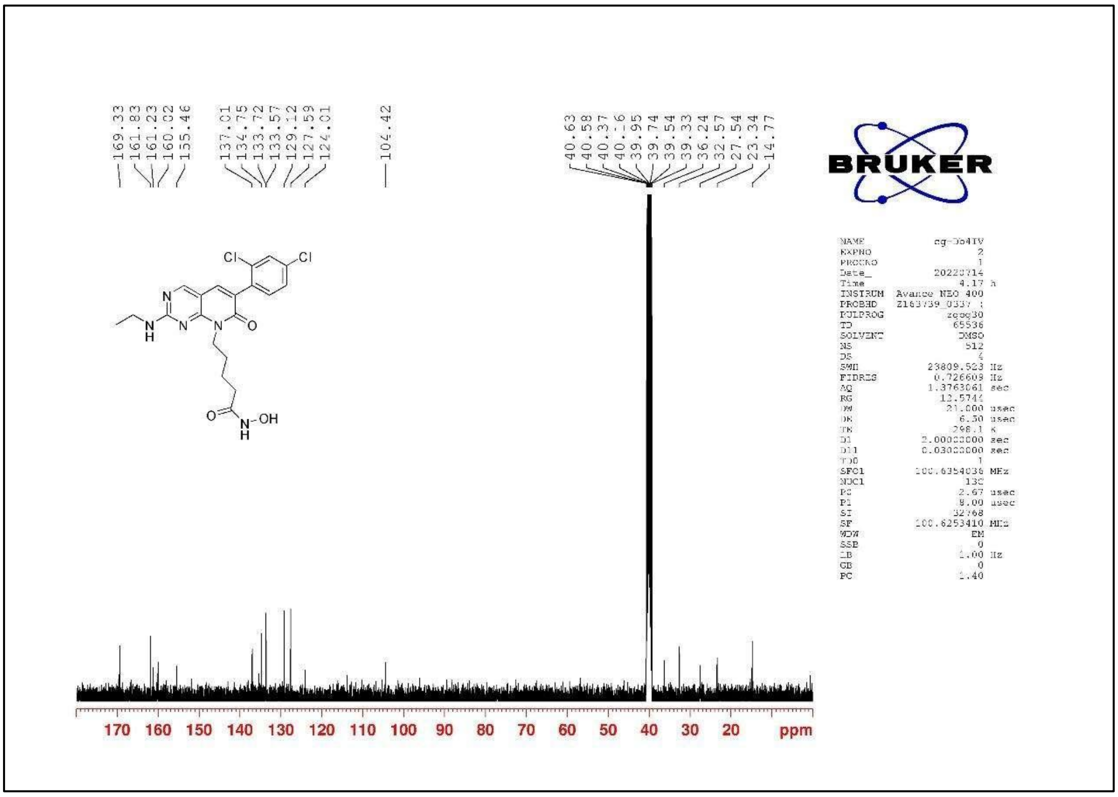

^{13}C-NMR spectrum of compound 21k

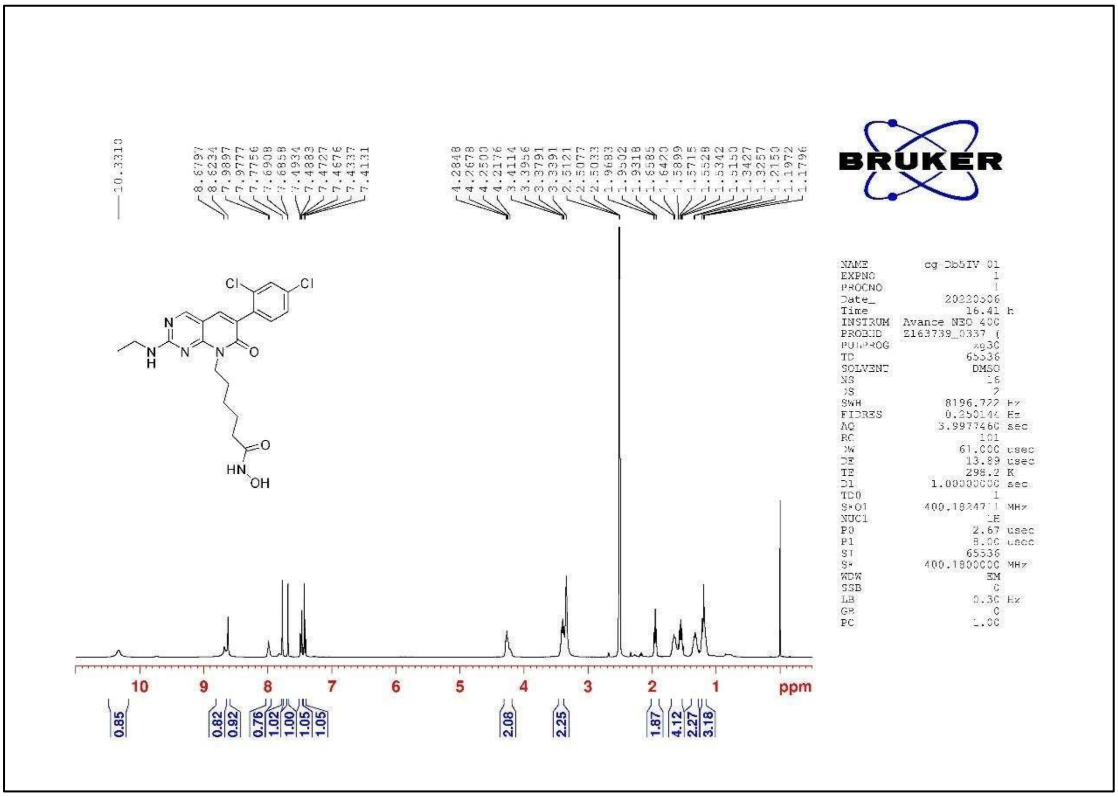

^{1}H-NMR spectrum of compound 21l

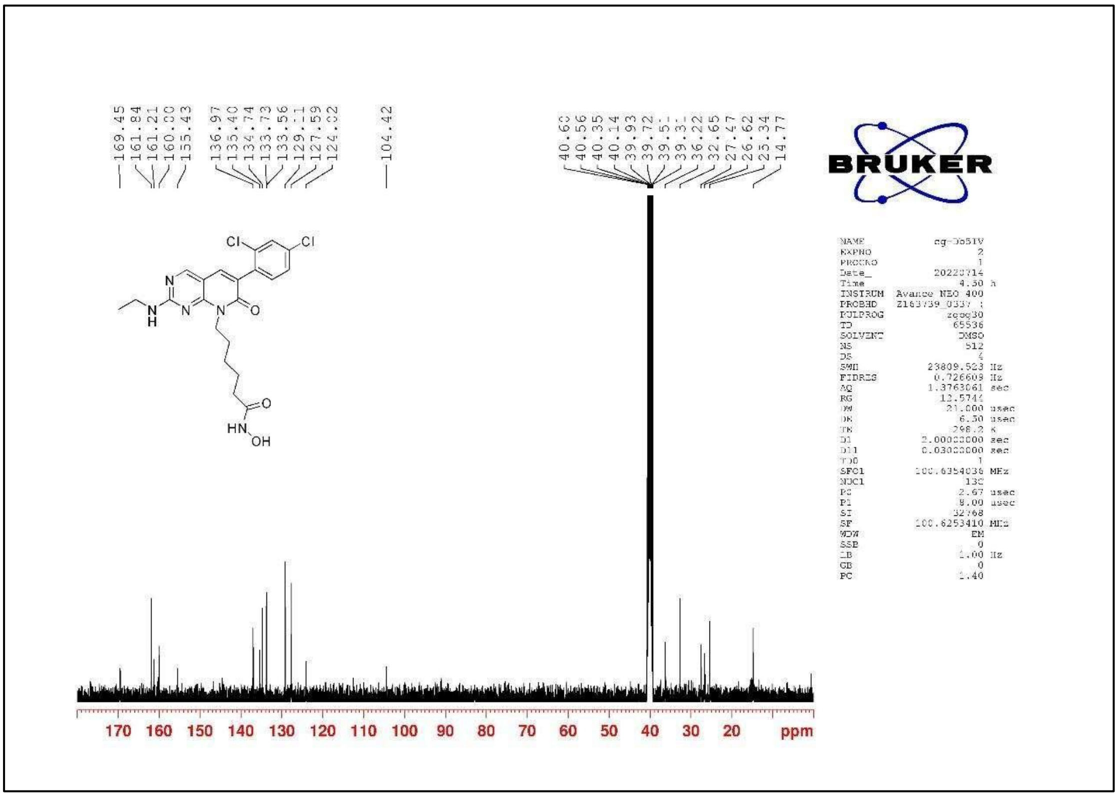

^{13}C-NMR spectrum of compound 21l

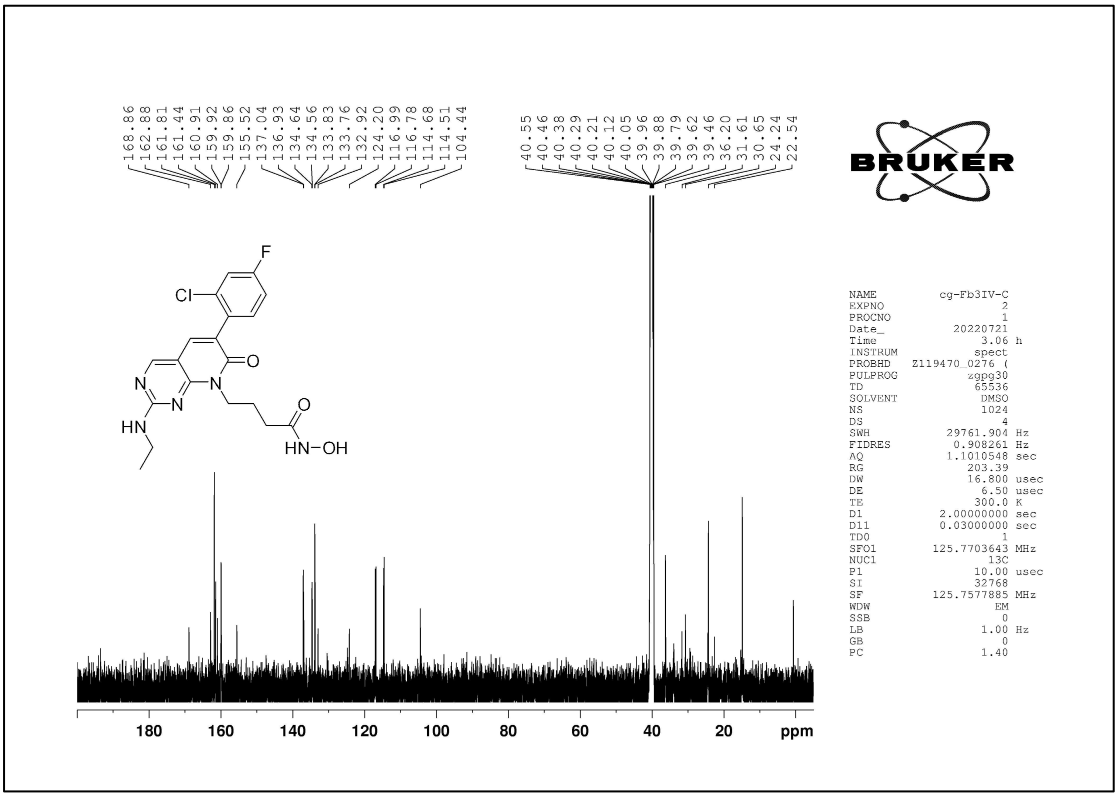

^{1}H-NMR spectrum of compound 21m

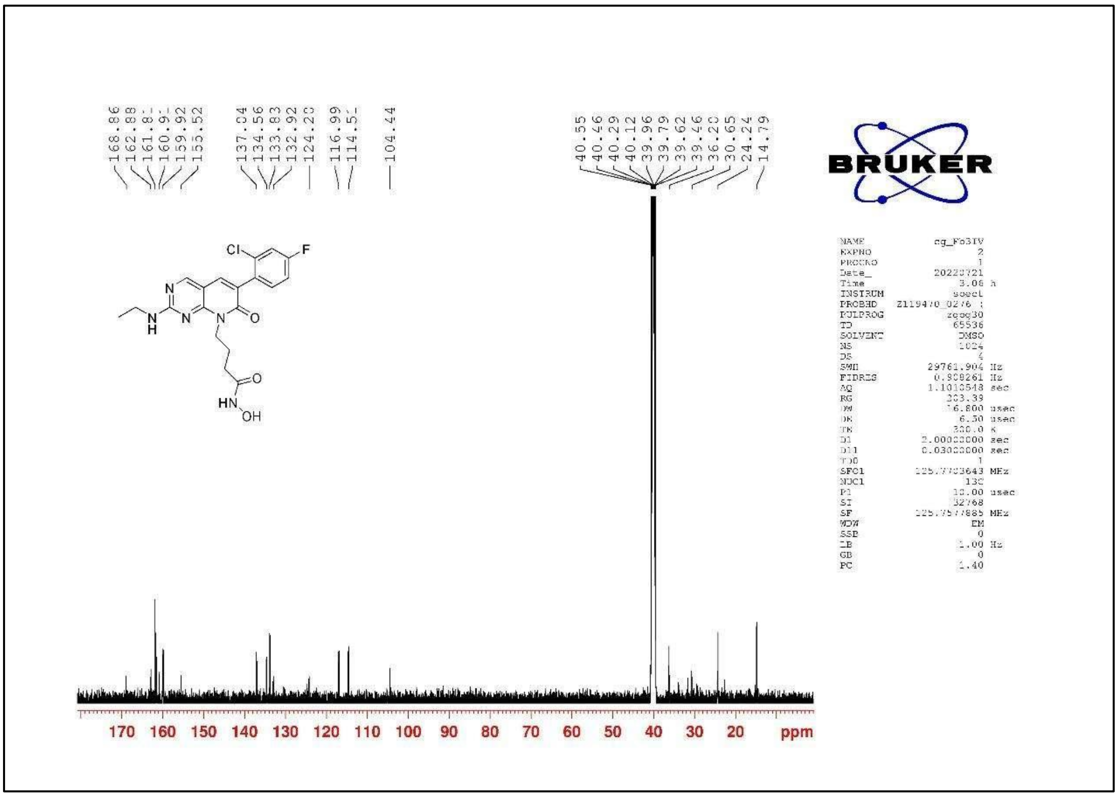

^{13}C-NMR spectrum of compound 21m

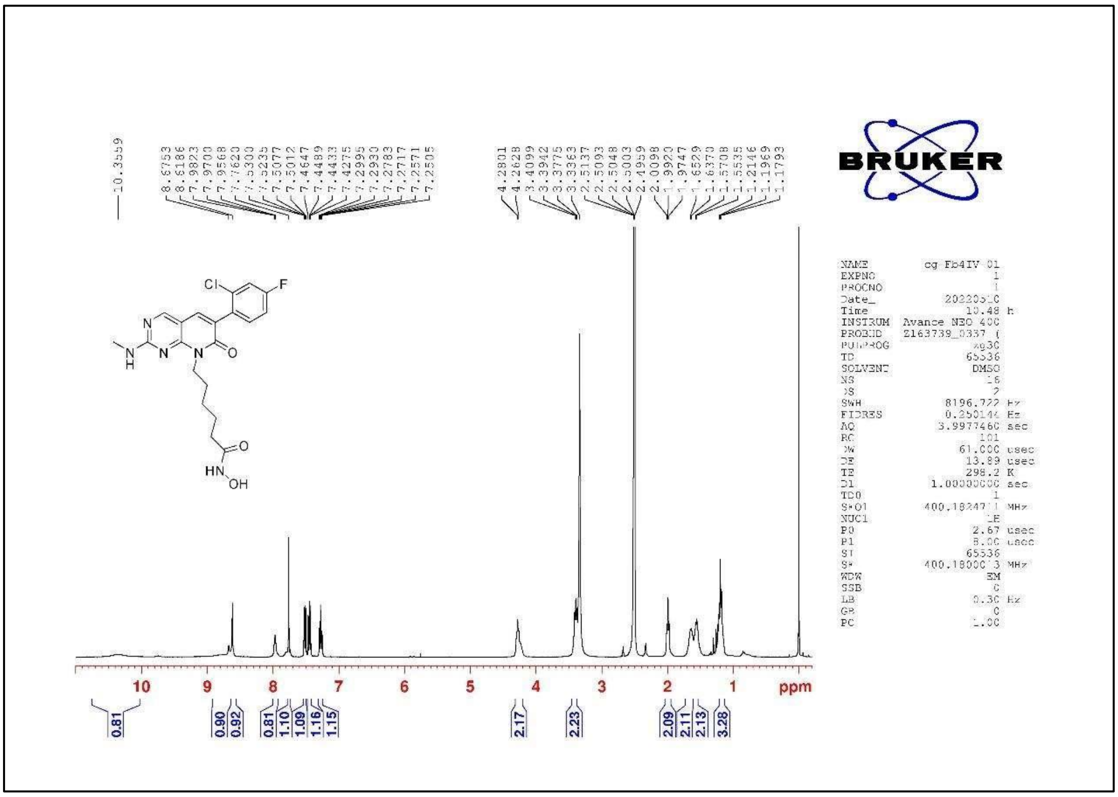

^{1}H-NMR spectrum of compound 21n

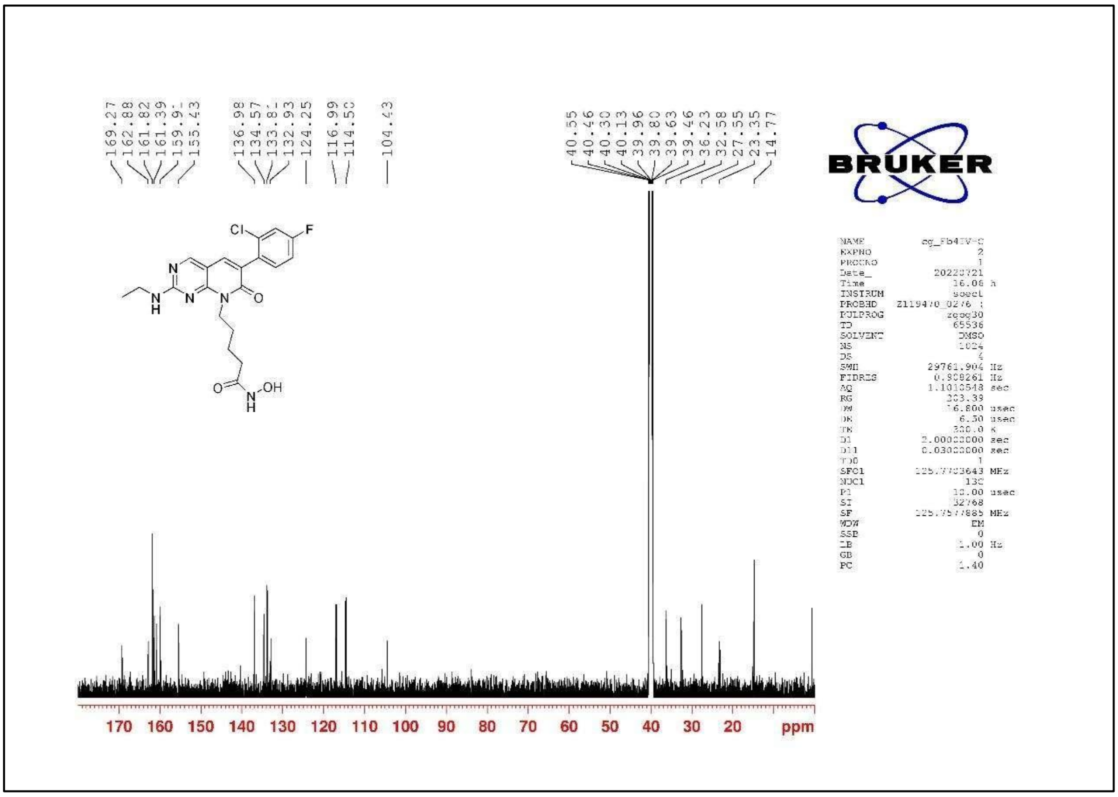

^{13}C-NMR spectrum of compound 21n

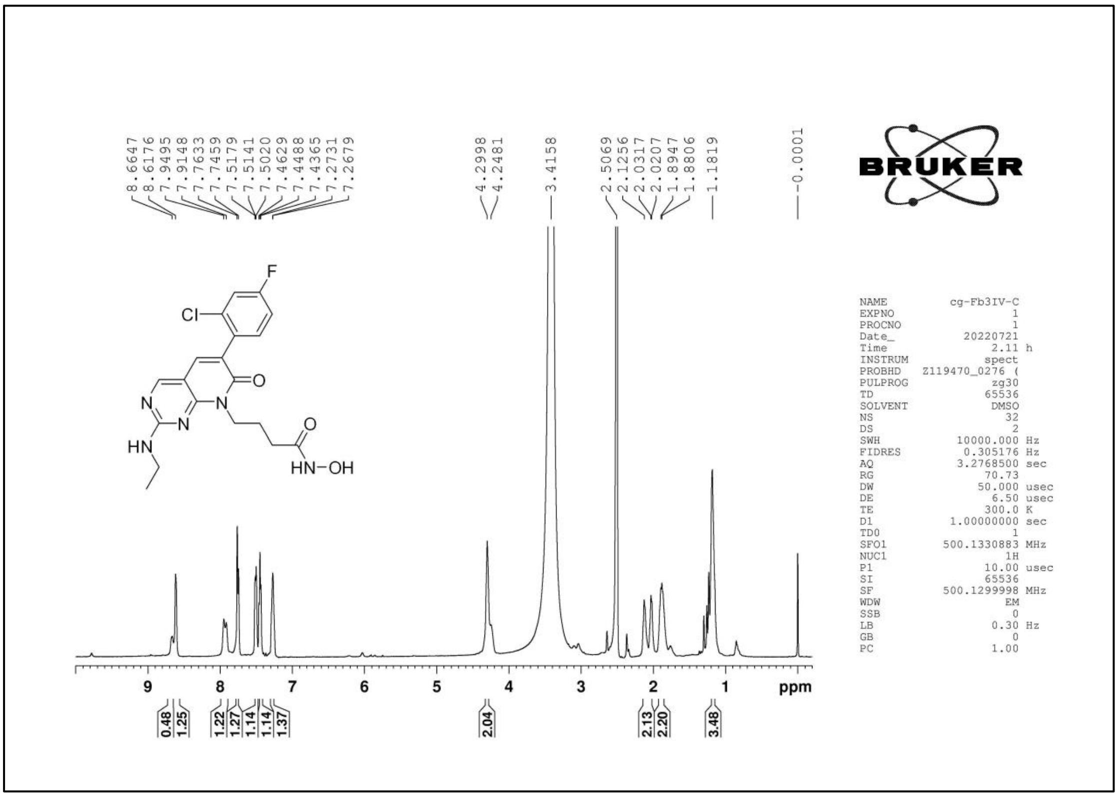

^{1}H-NMR spectrum of compound 21o

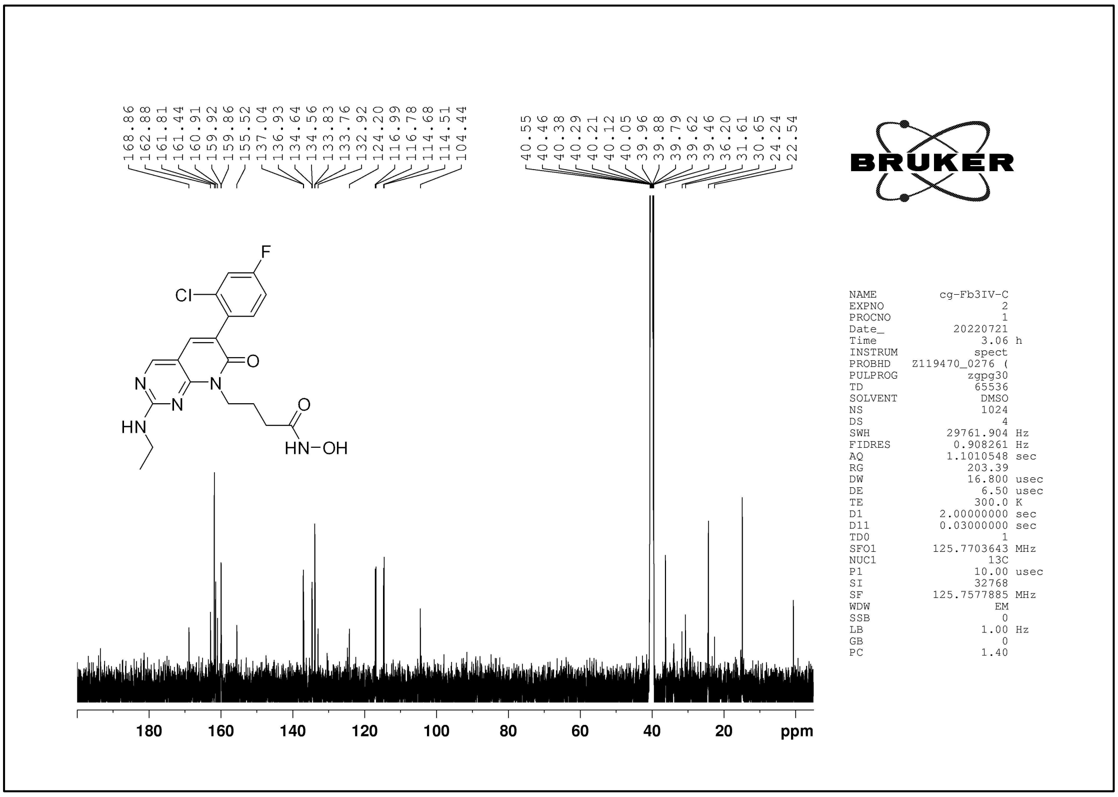

^{13}C-NMR spectrum of compound 21o

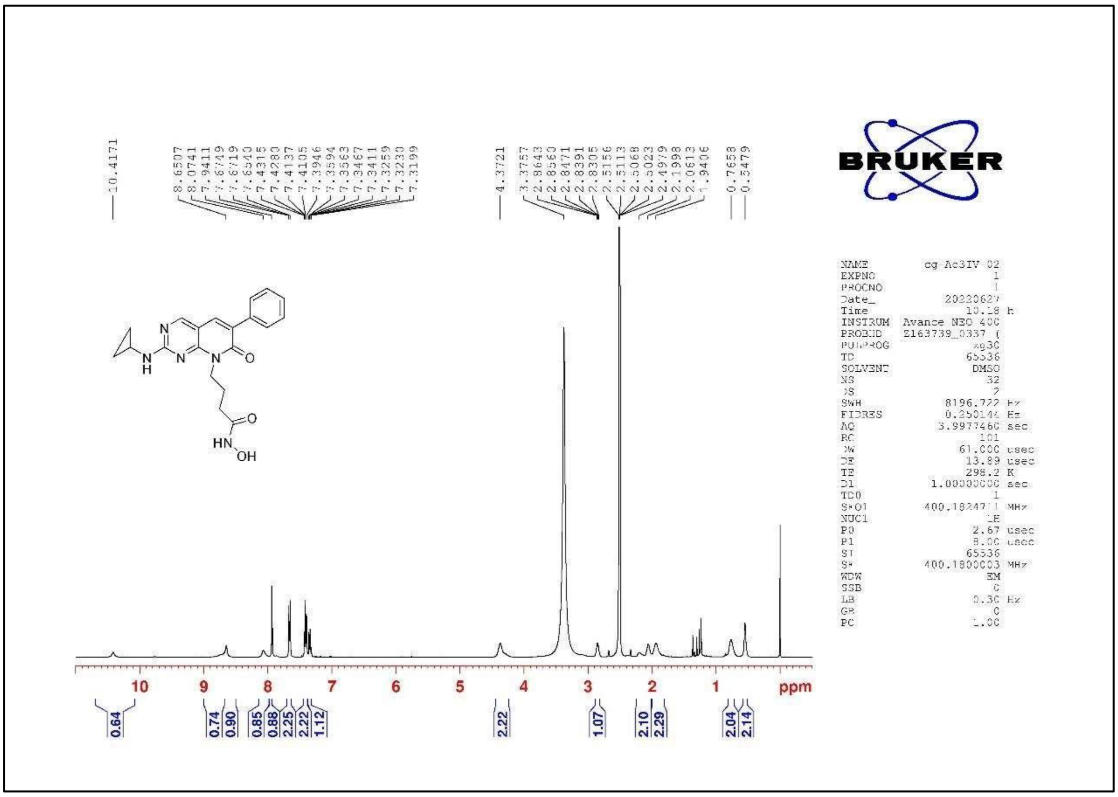

^{1}H-NMR spectrum of compound 22a

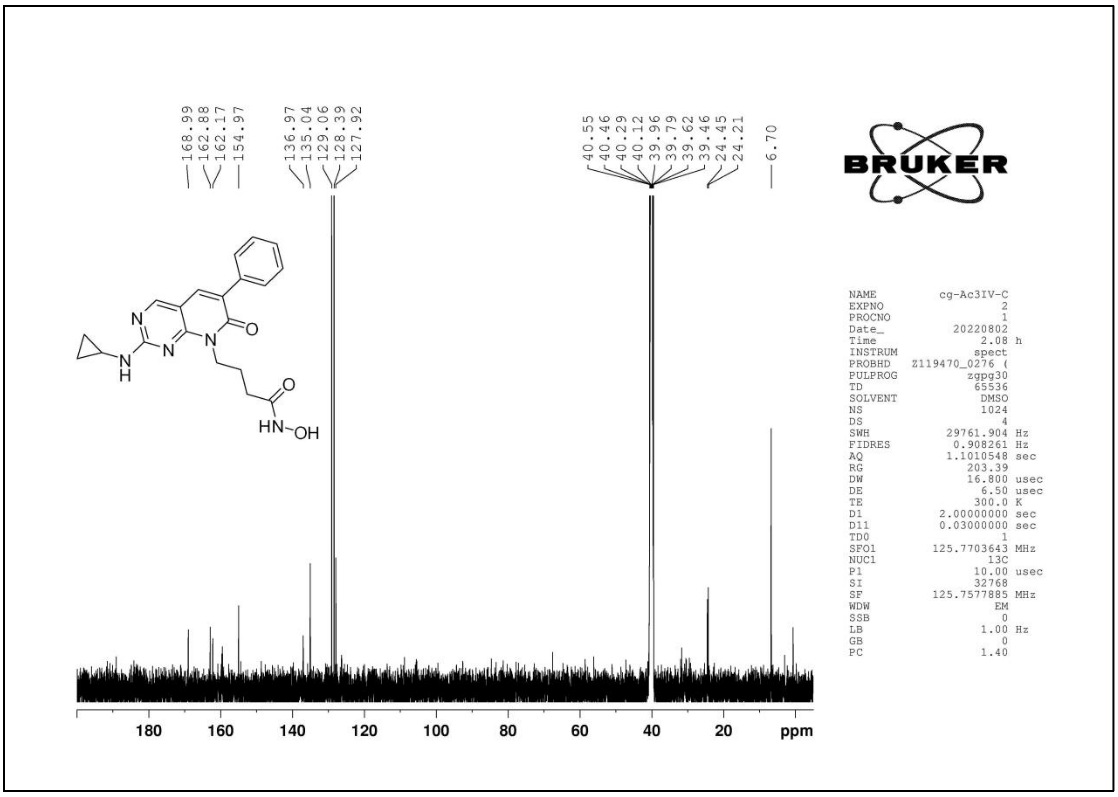

^{13}C-NMR spectrum of compound 22a

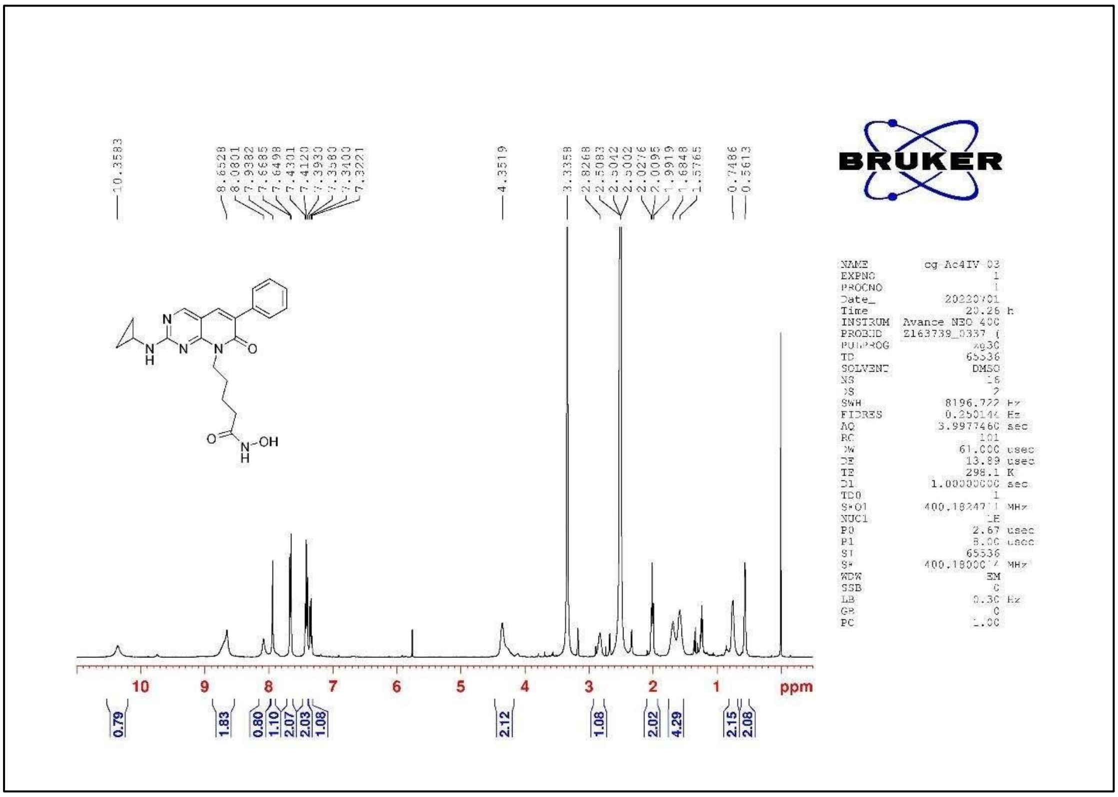

^{1}H-NMR spectrum of compound 22b

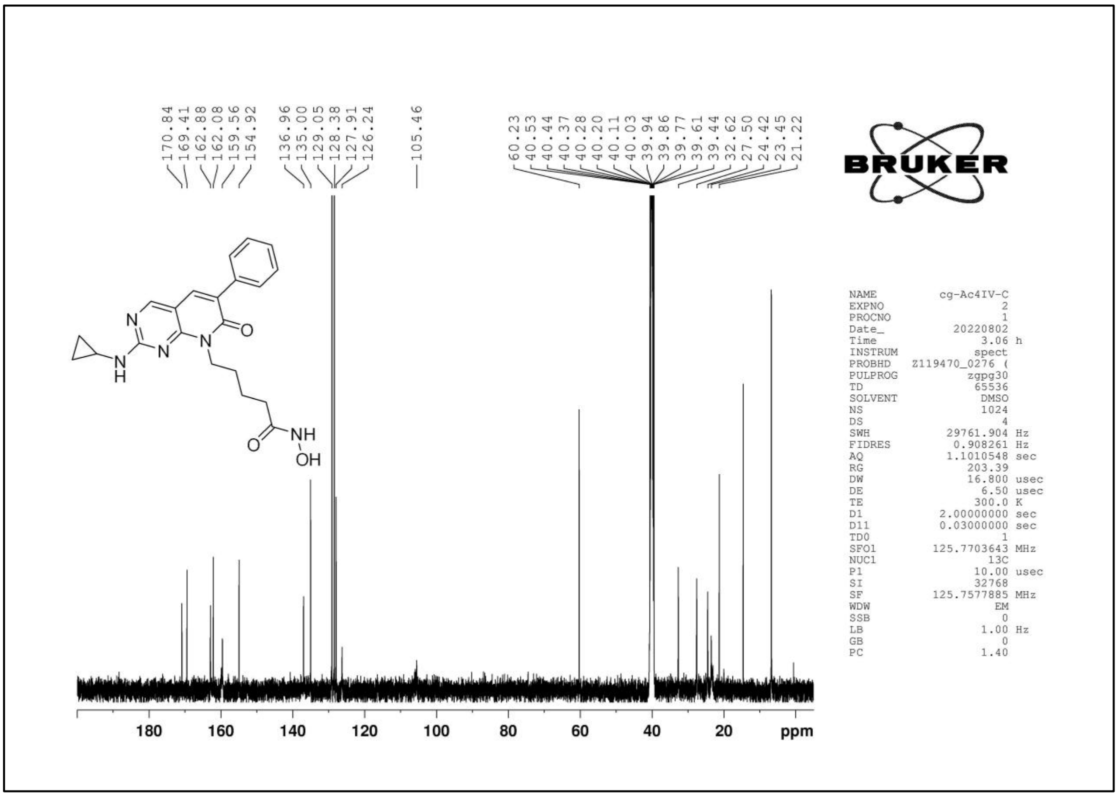

^{13}C-NMR spectrum of compound 22b

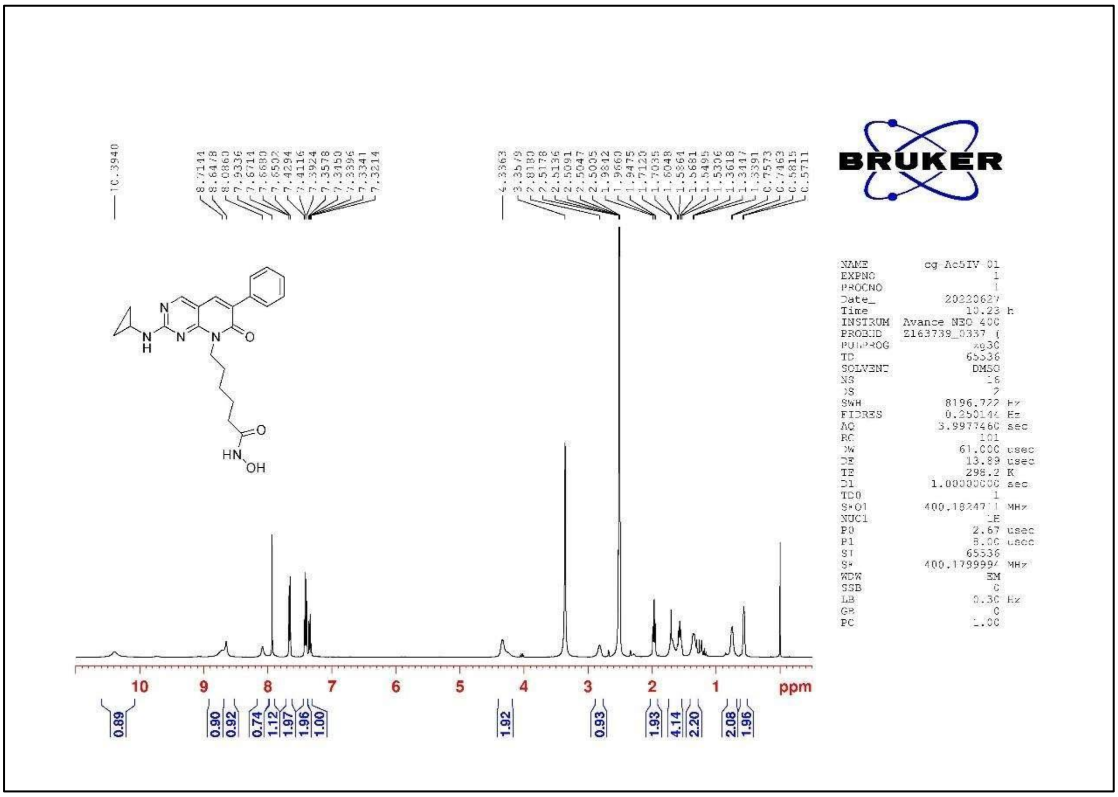

^{1}H-NMR spectrum of compound 22c

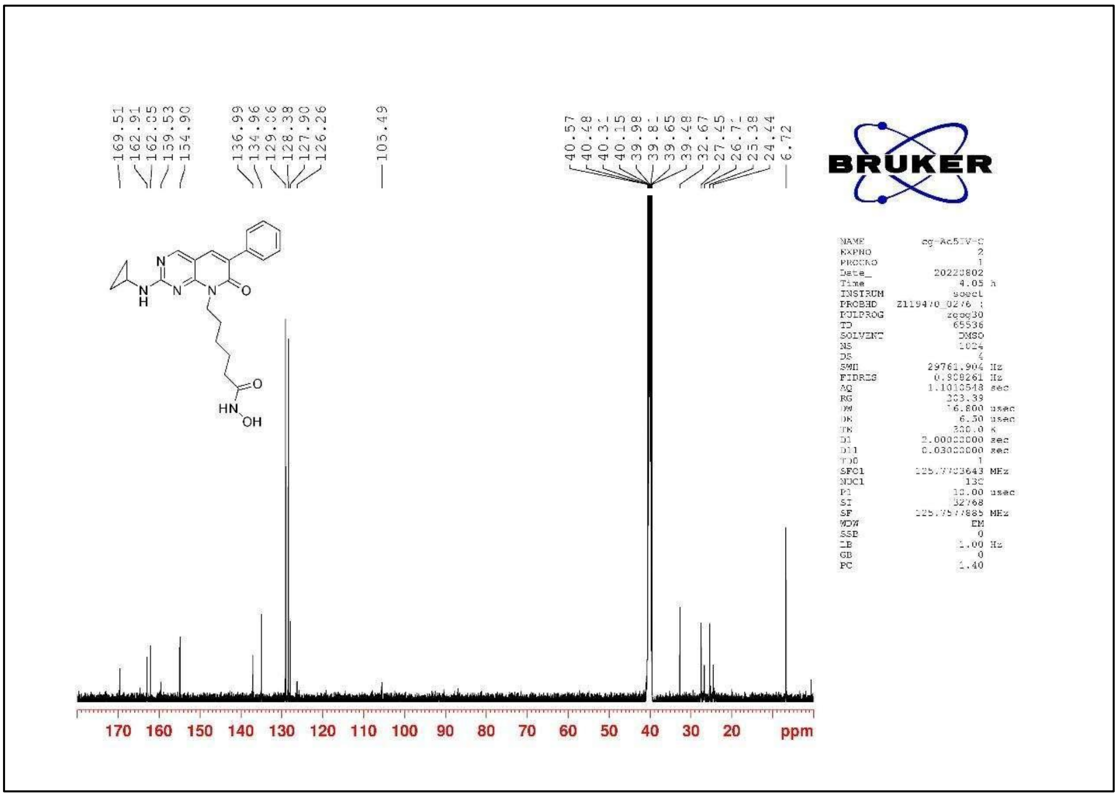

^{13}C-NMR spectrum of compound 22c

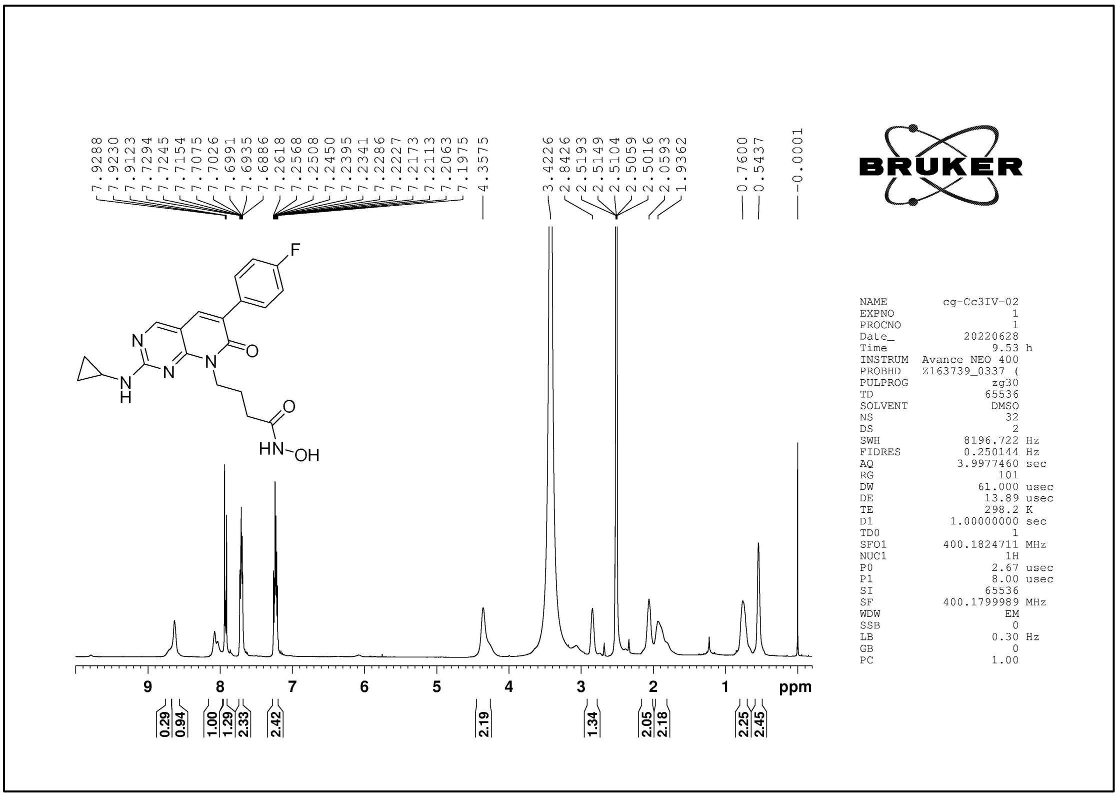

^{1}H-NMR spectrum of compound 22d**

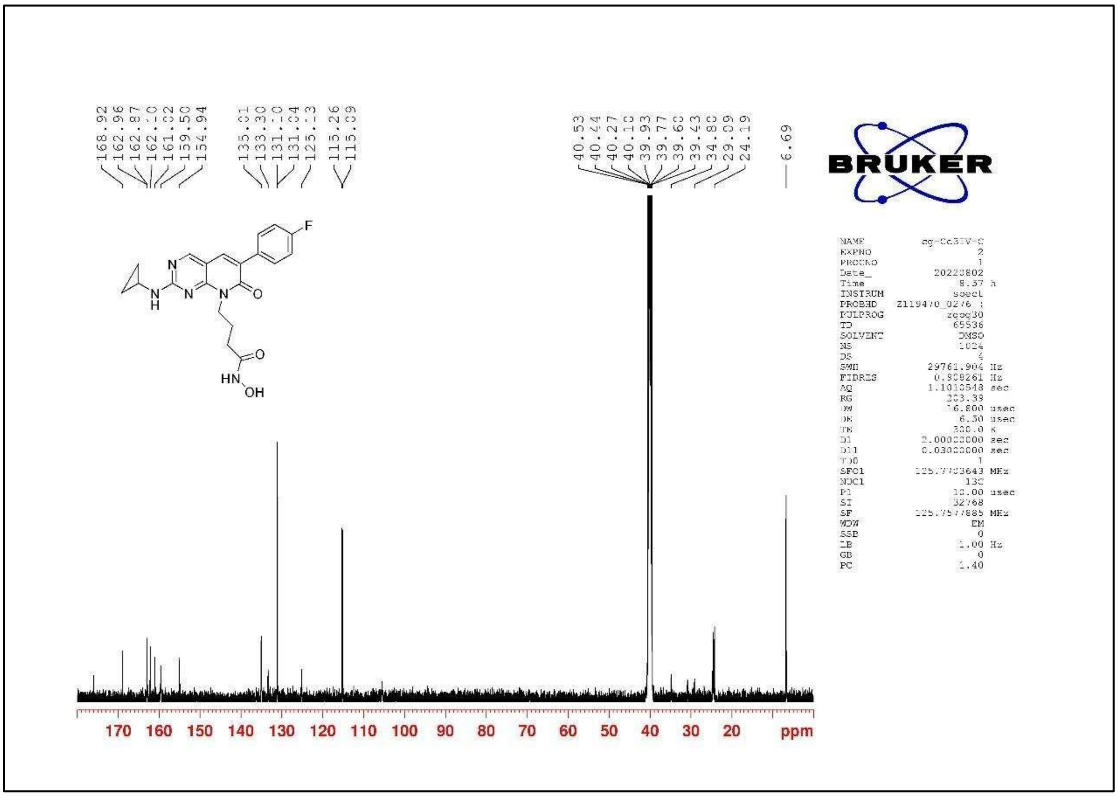

^{13}C-NMR spectrum of compound 22d**

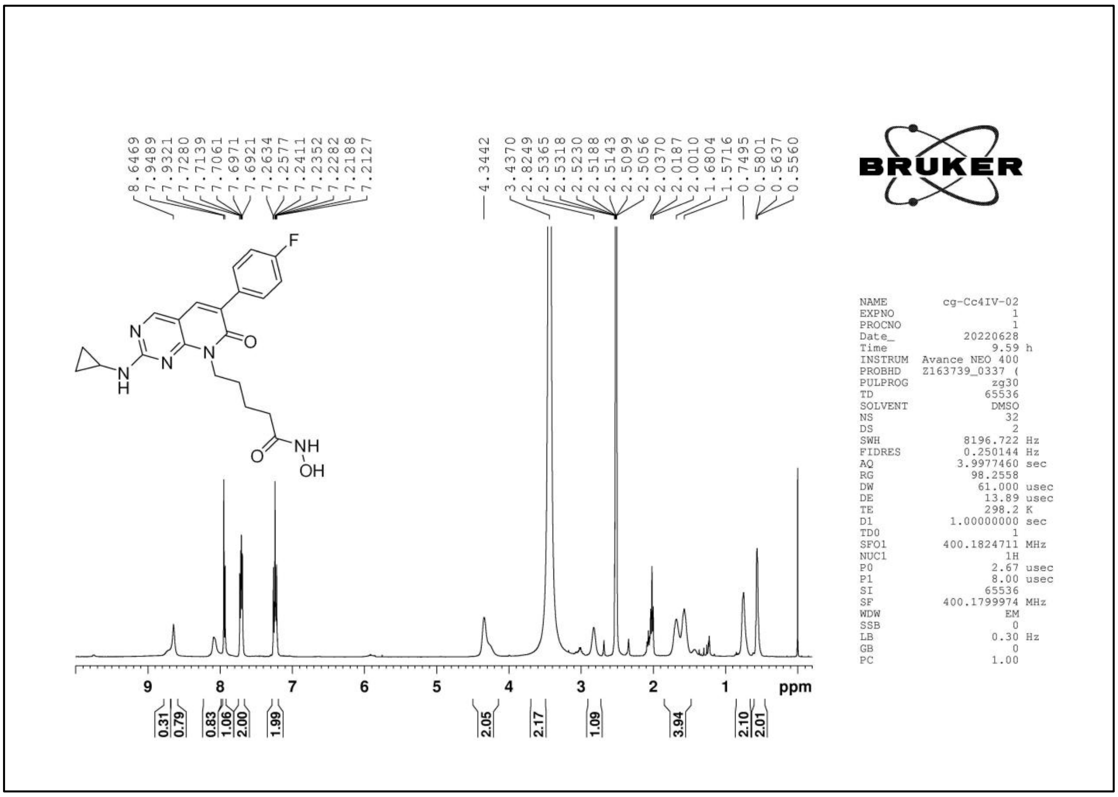

^{1}H-NMR spectrum of compound 22e

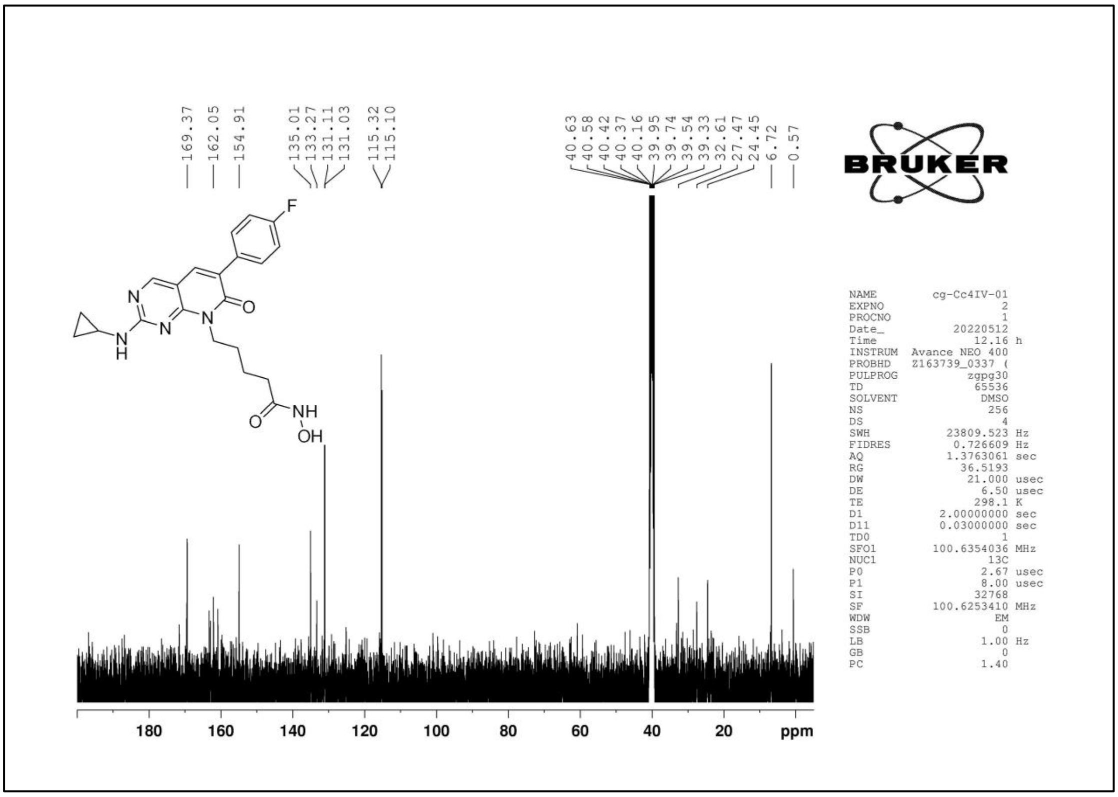

^{13}C-NMR spectrum of compound 22e

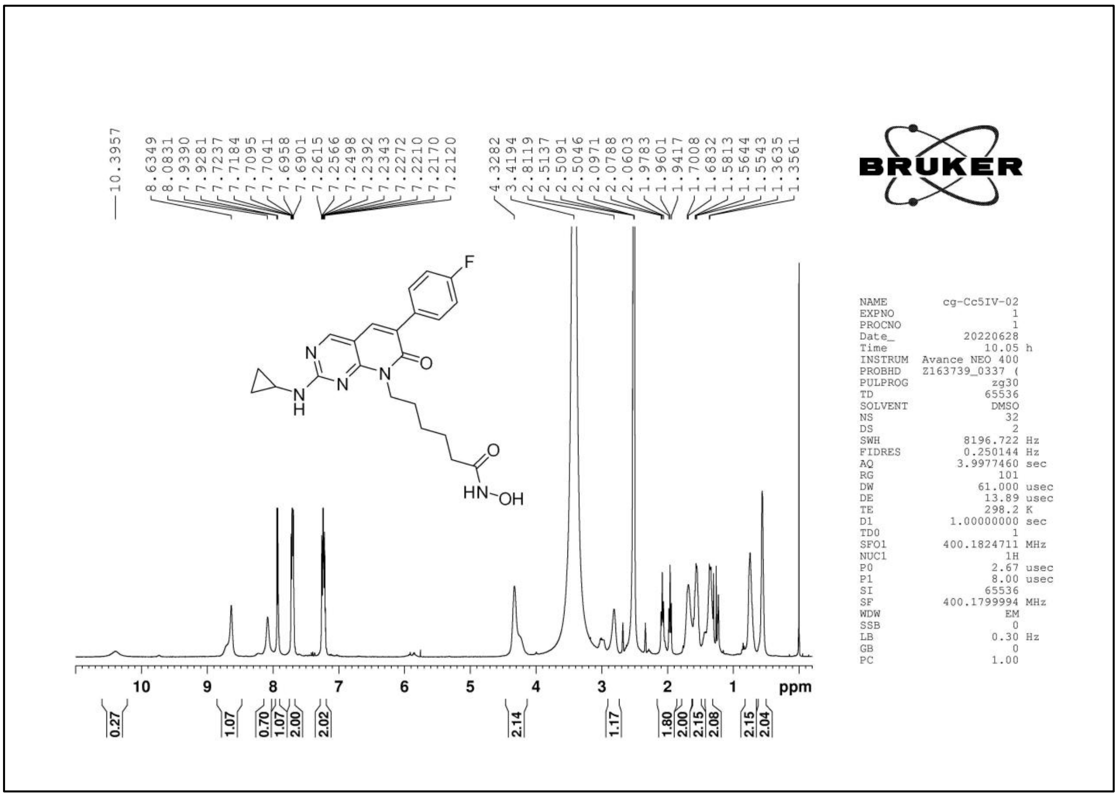

^{1}H-NMR spectrum of compound 22f

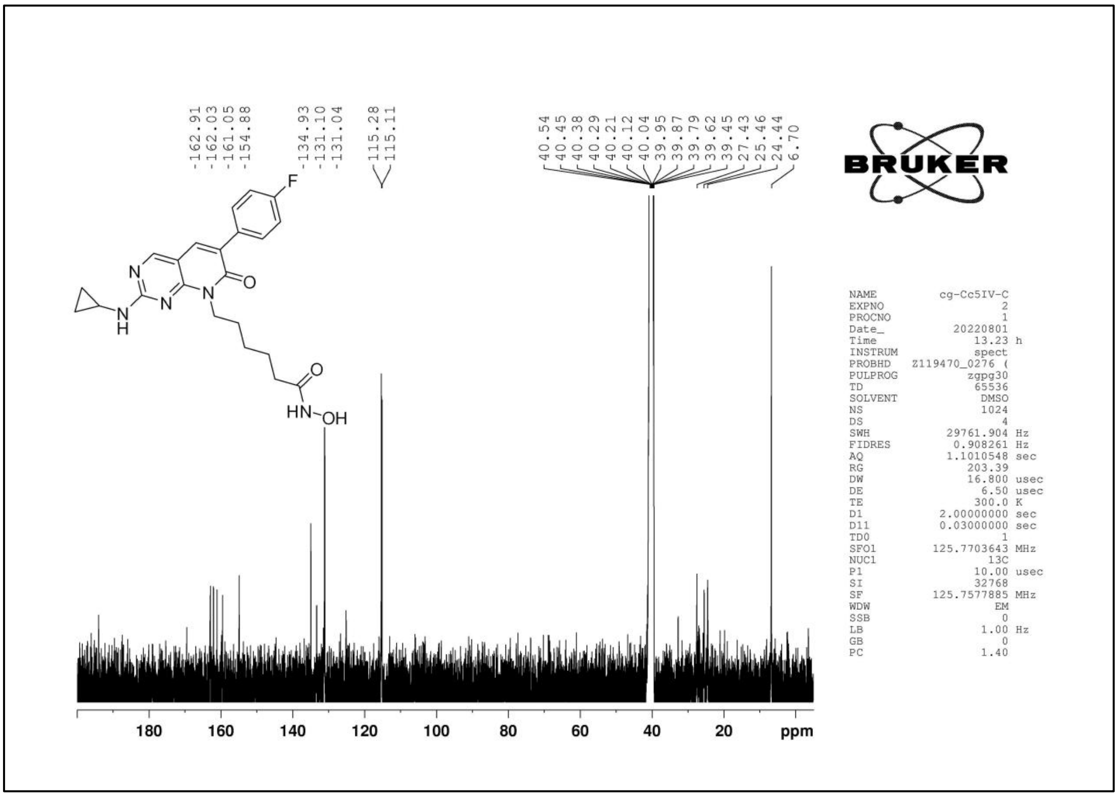

^{13}C-NMR spectrum of compound 22f

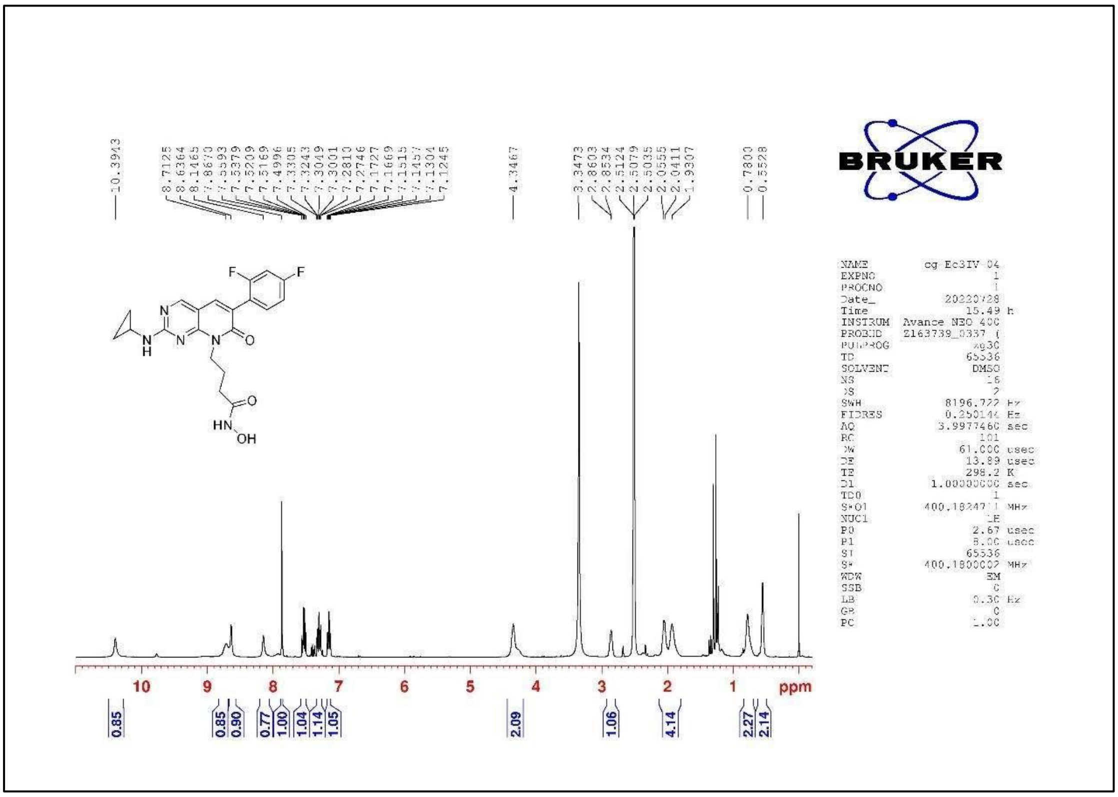

^{1}H-NMR spectrum of compound 22g

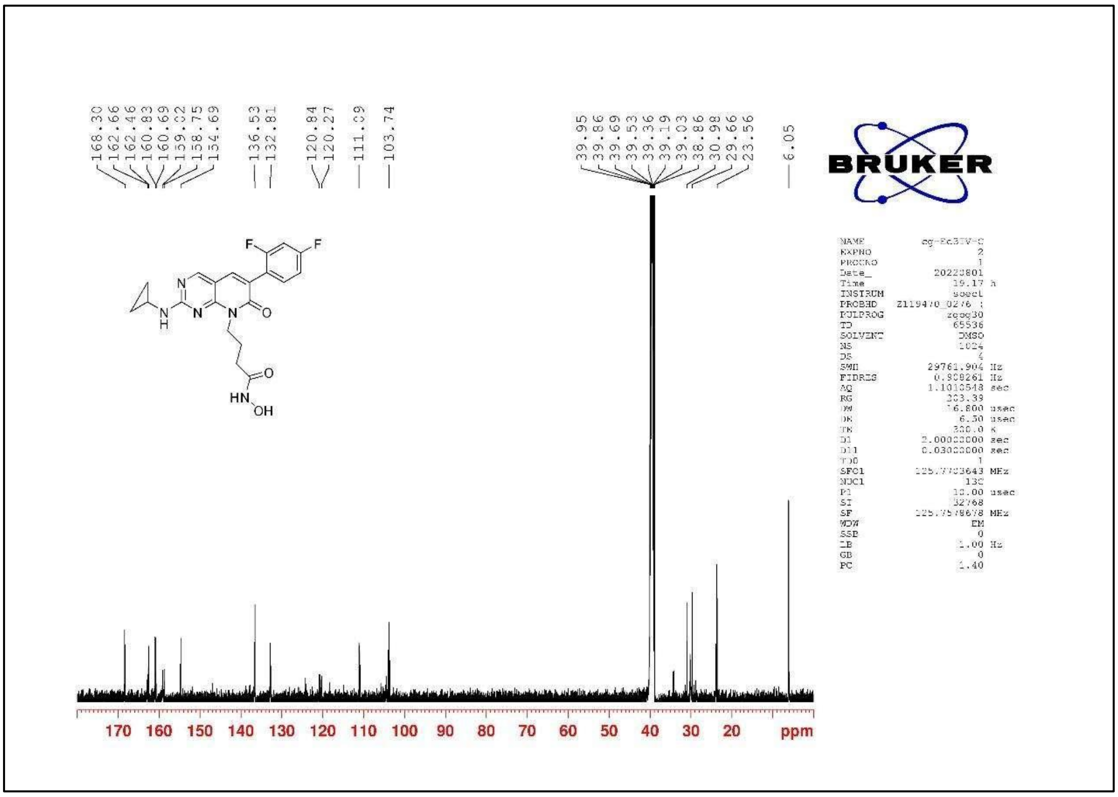

^{13}C-NMR spectrum of compound 22g

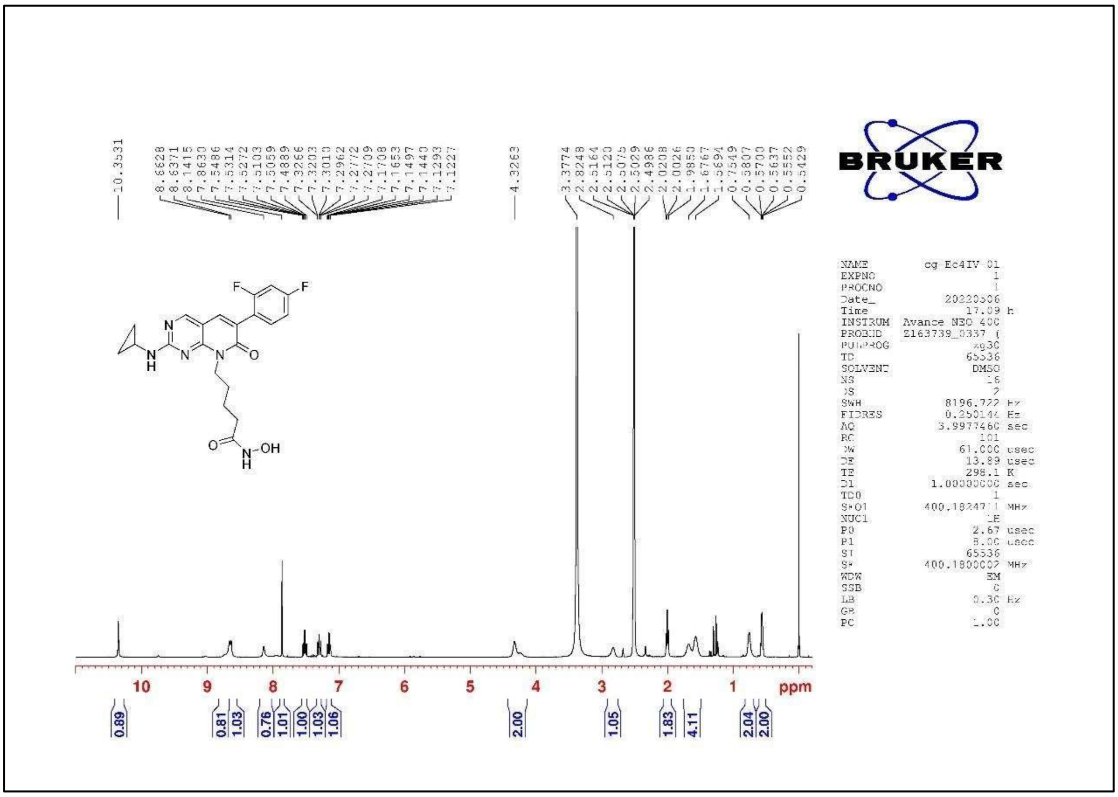

^{1}H-NMR spectrum of compound 22h

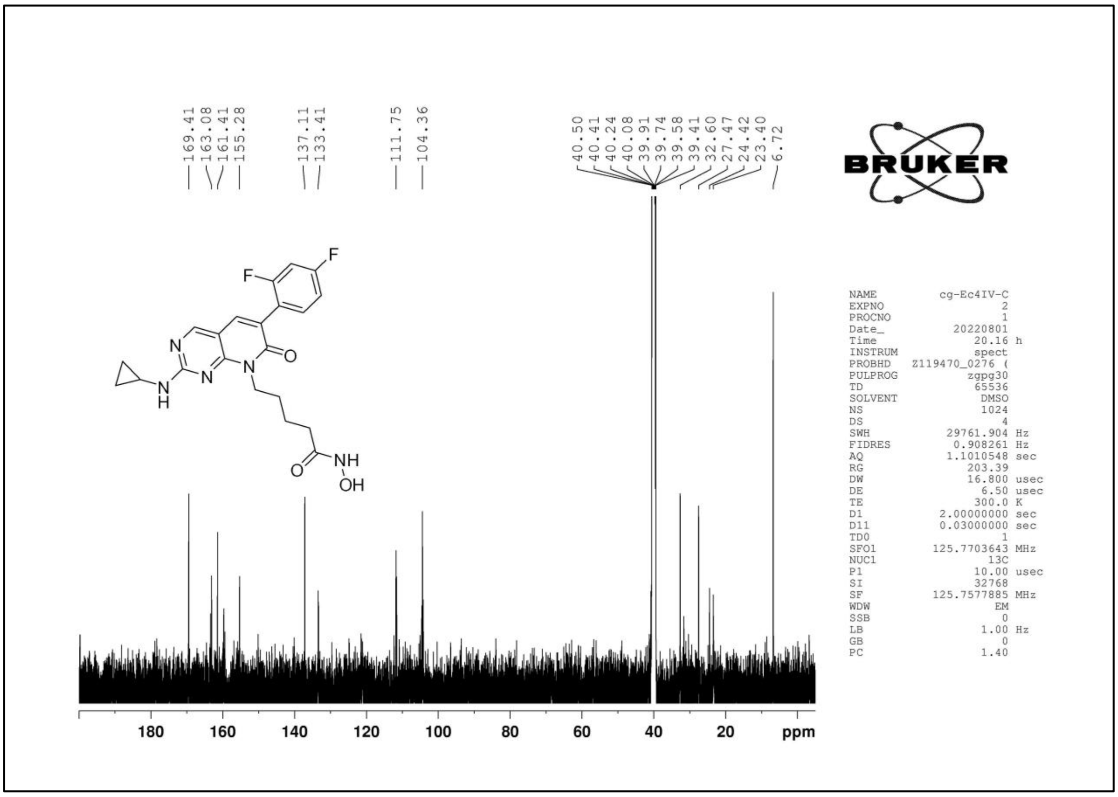

^{13}C-NMR spectrum of compound 22h

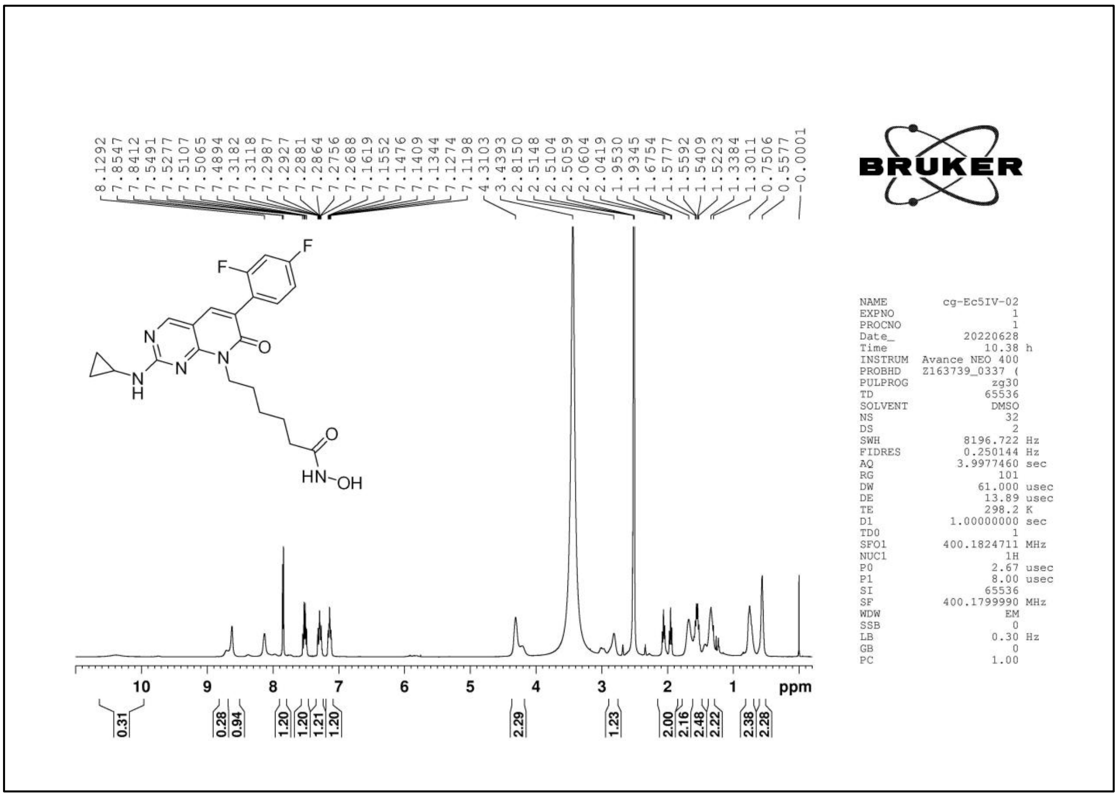

^{1}H-NMR spectrum of compound 22i

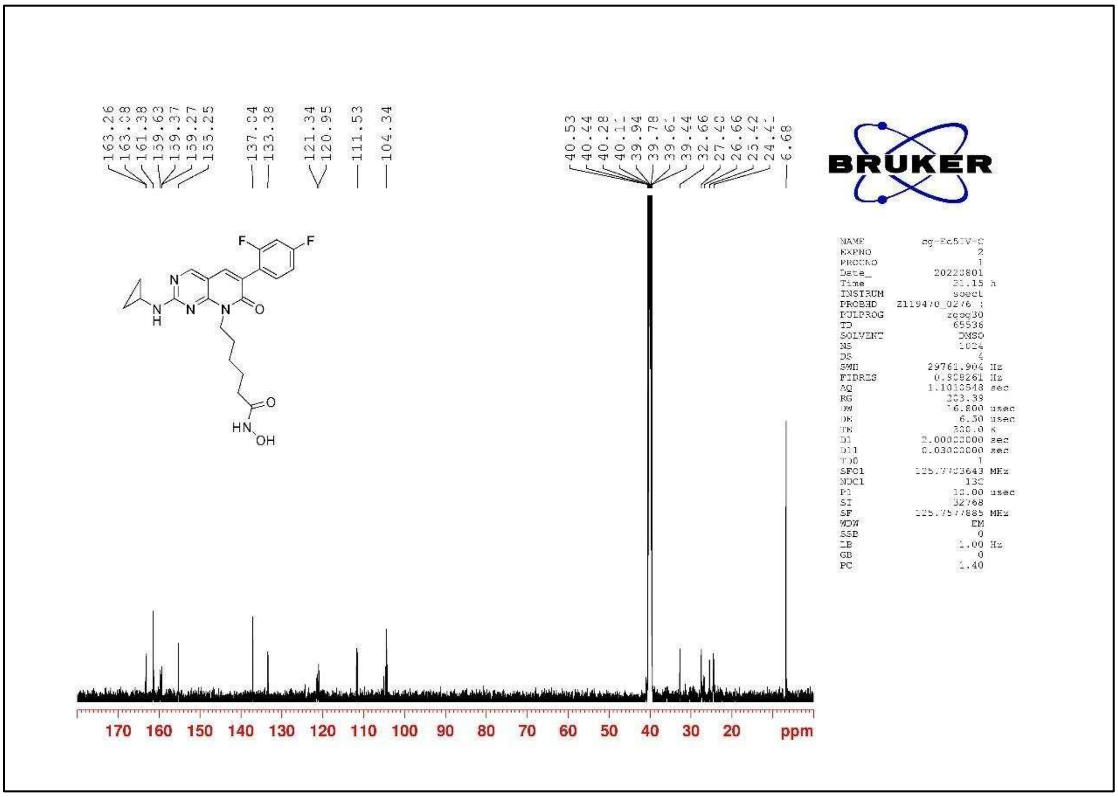

^{13}C-NMR spectrum of compound 22i

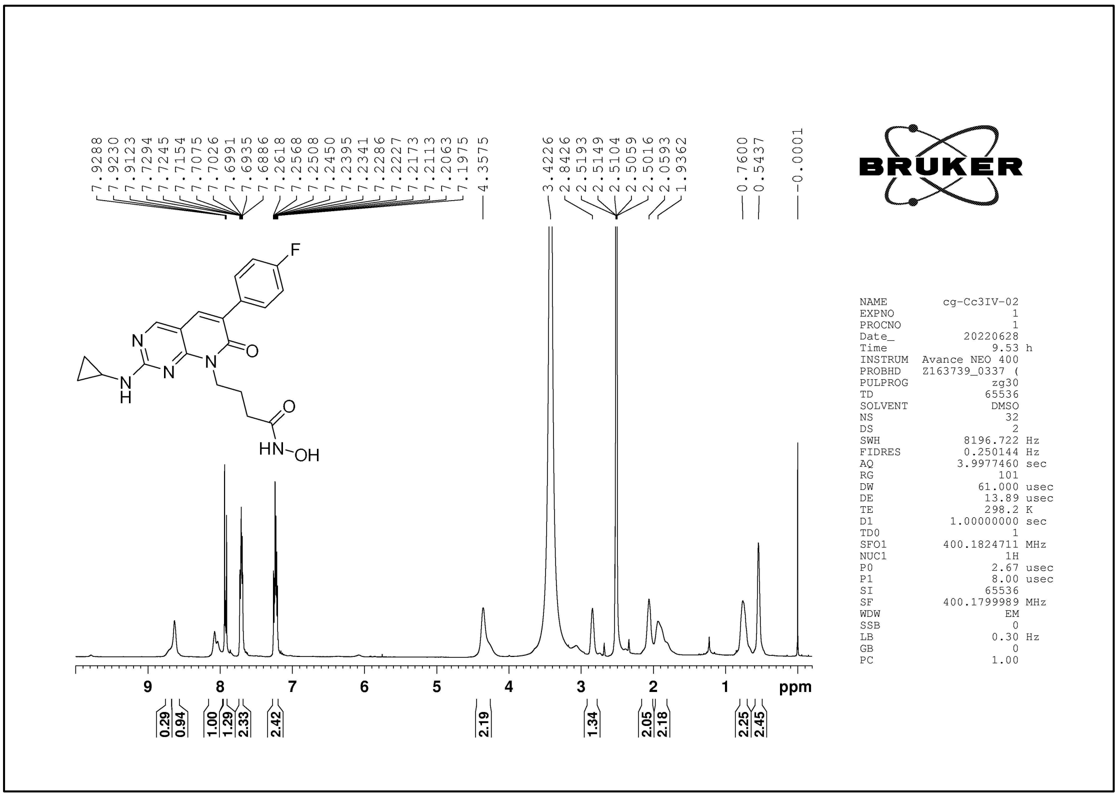

^{1}H-NMR spectrum of compound 22j

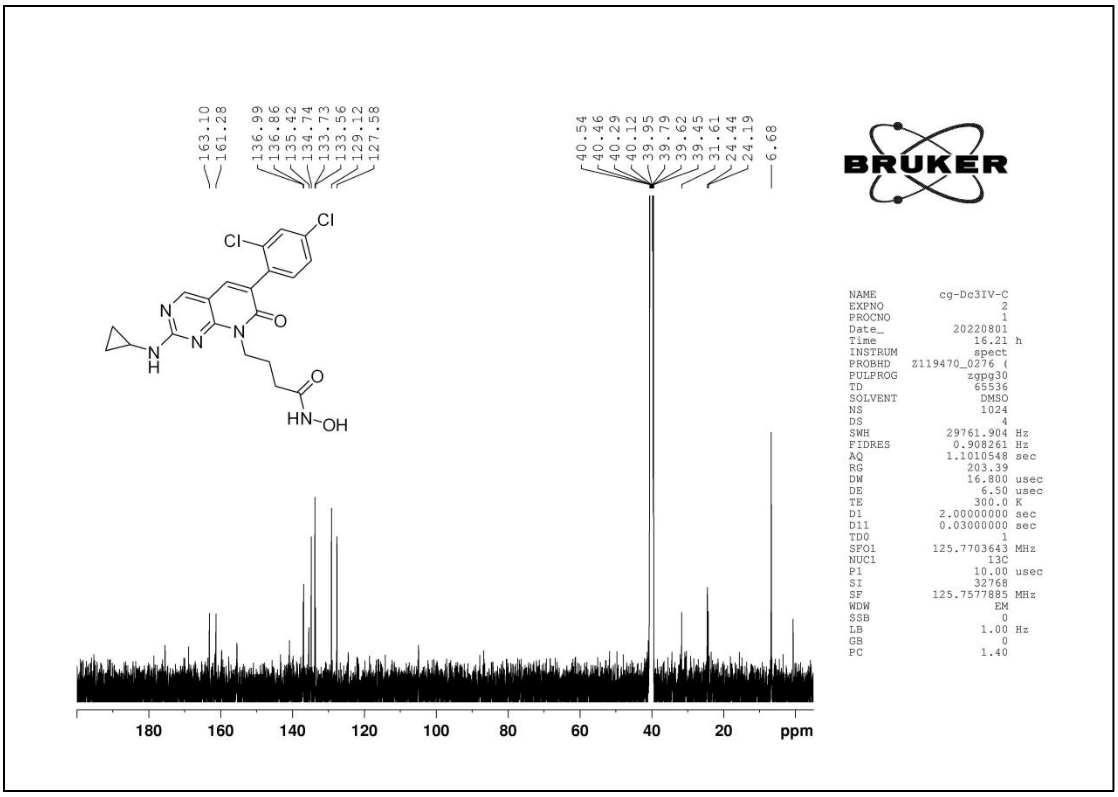

^{13}C-NMR spectrum of compound 22j

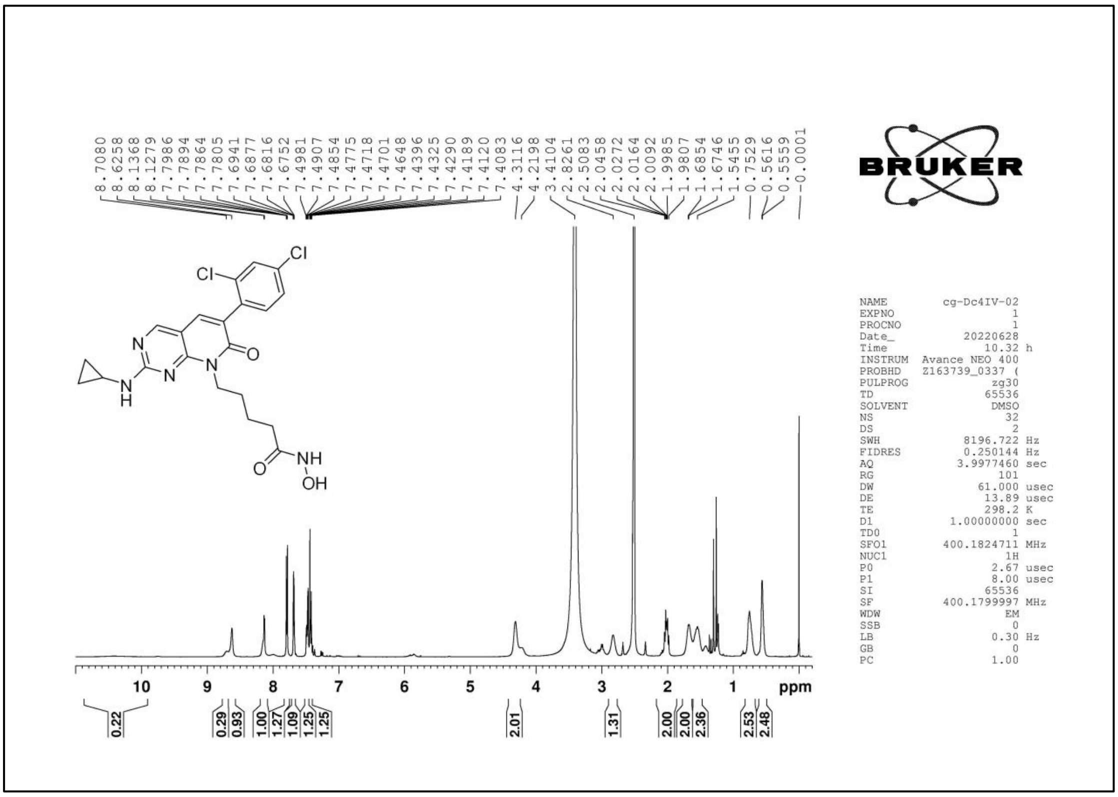

^{1}H-NMR spectrum of compound 22k

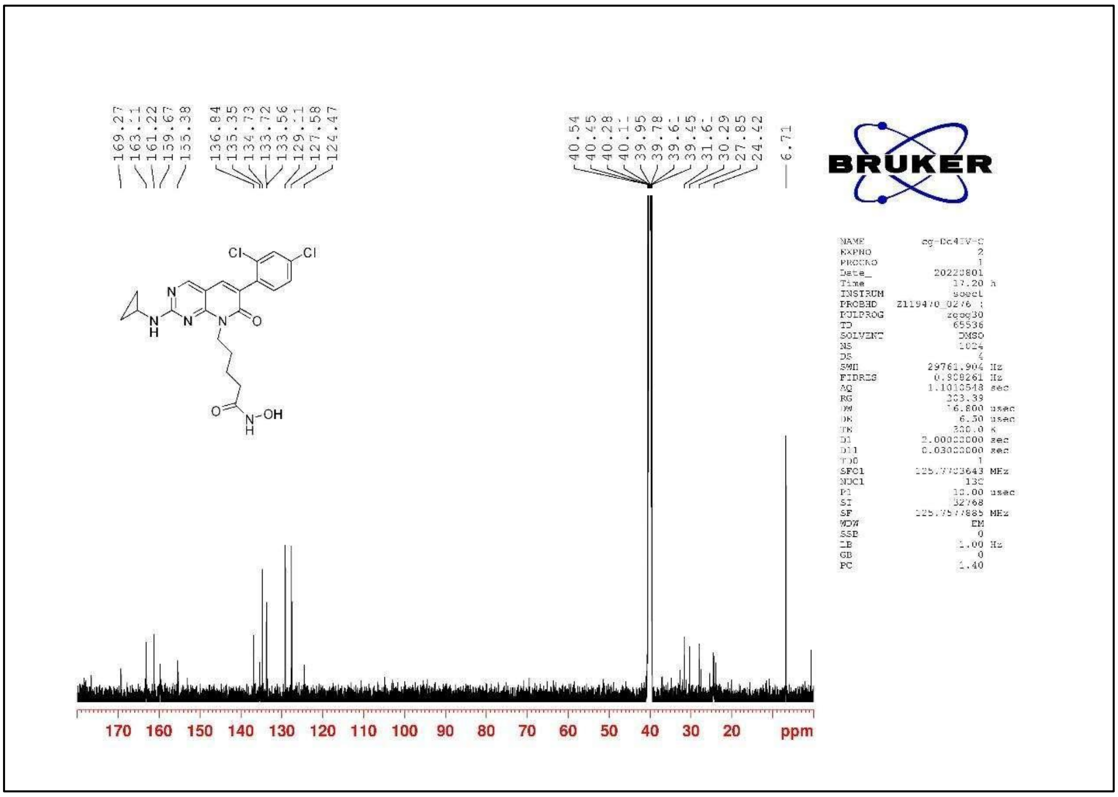

^{13}C-NMR spectrum of compound 22k

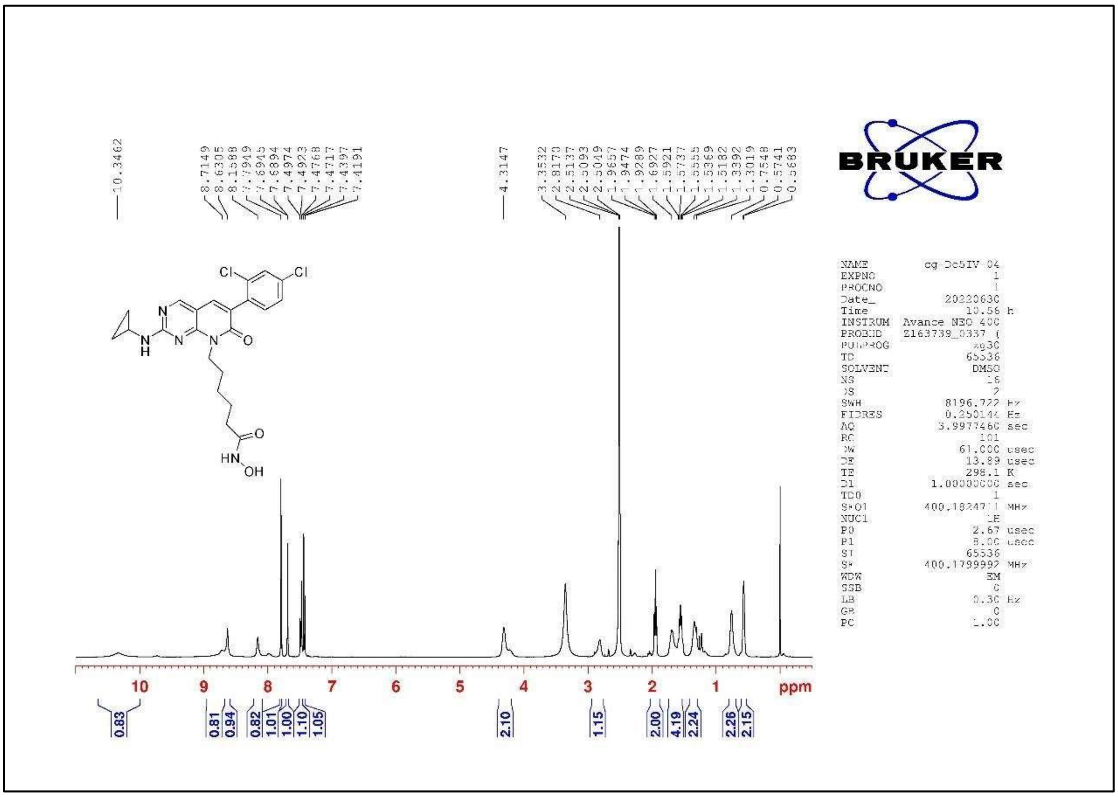

^{1}H-NMR spectrum of compound 22l

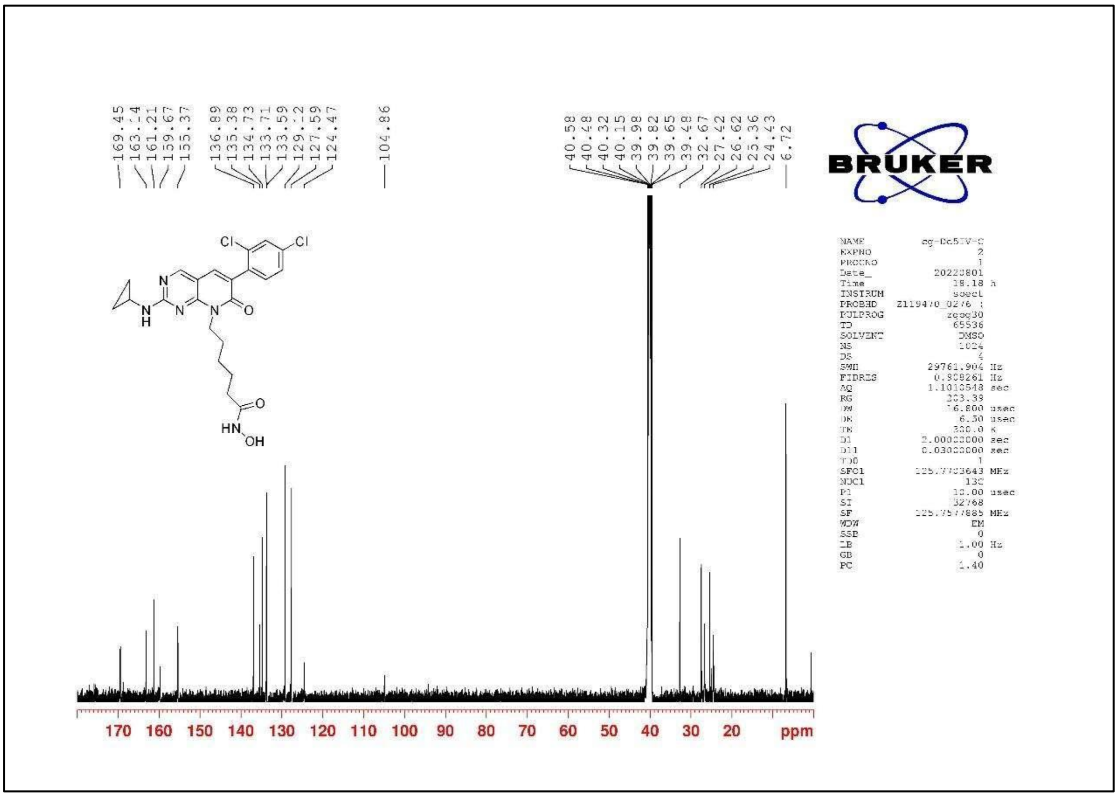

^{13}C-NMR spectrum of compound 22l

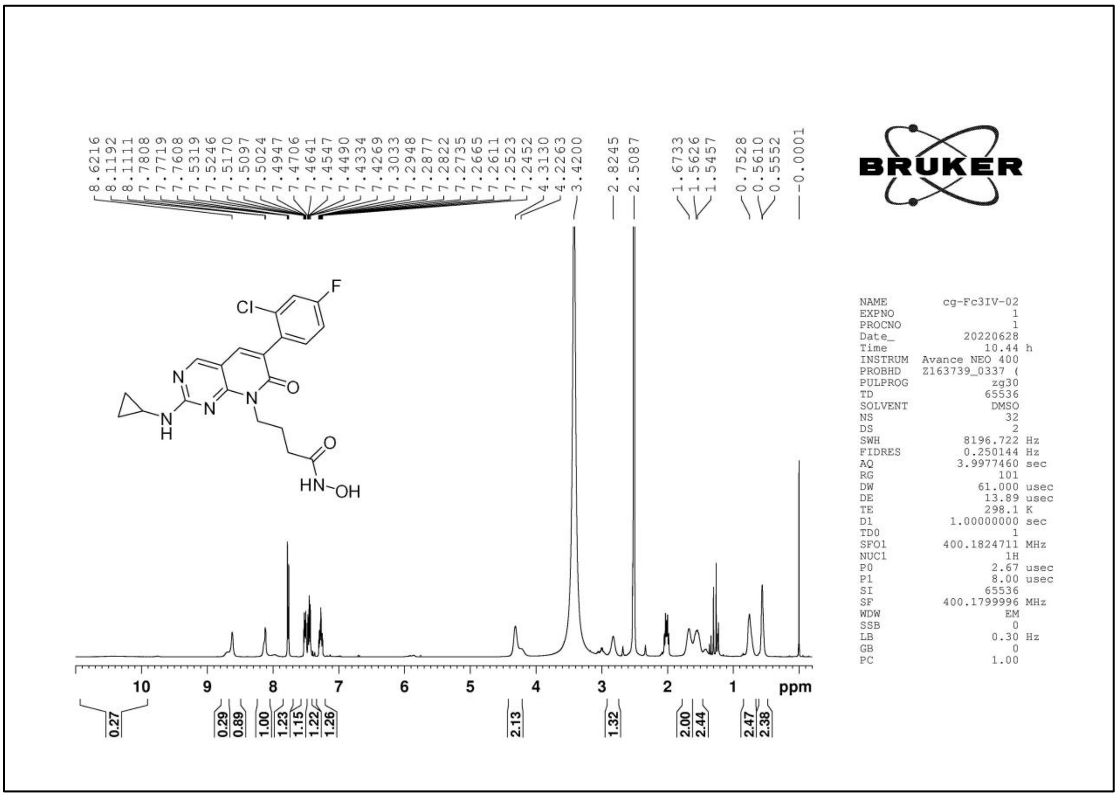

^{1}H-NMR spectrum of compound 22m

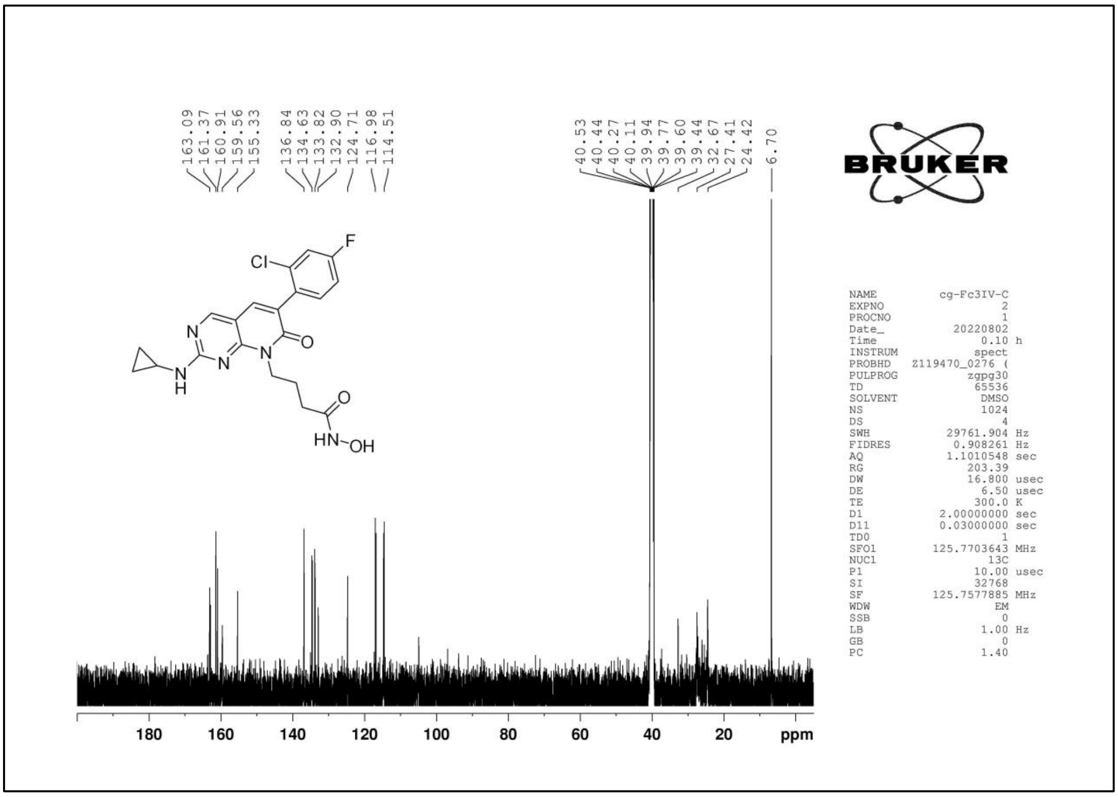

^{13}C-NMR spectrum of compound 22m

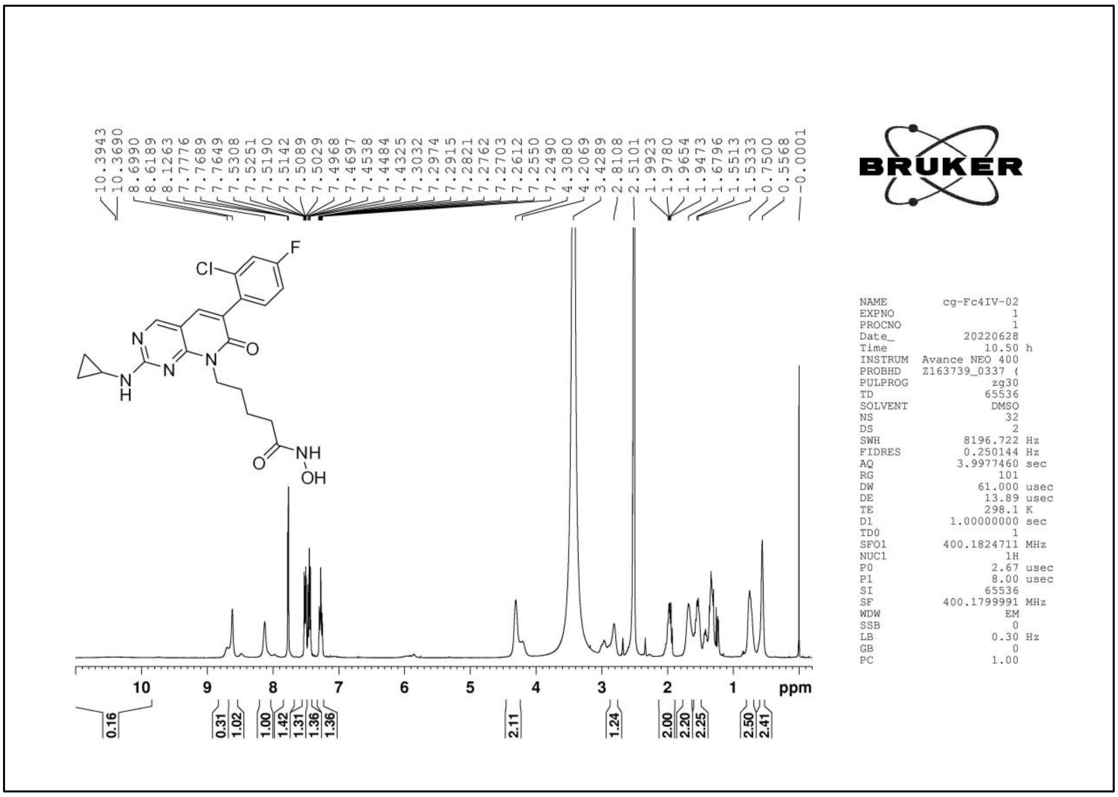

^{1}H-NMR spectrum of compound 22n

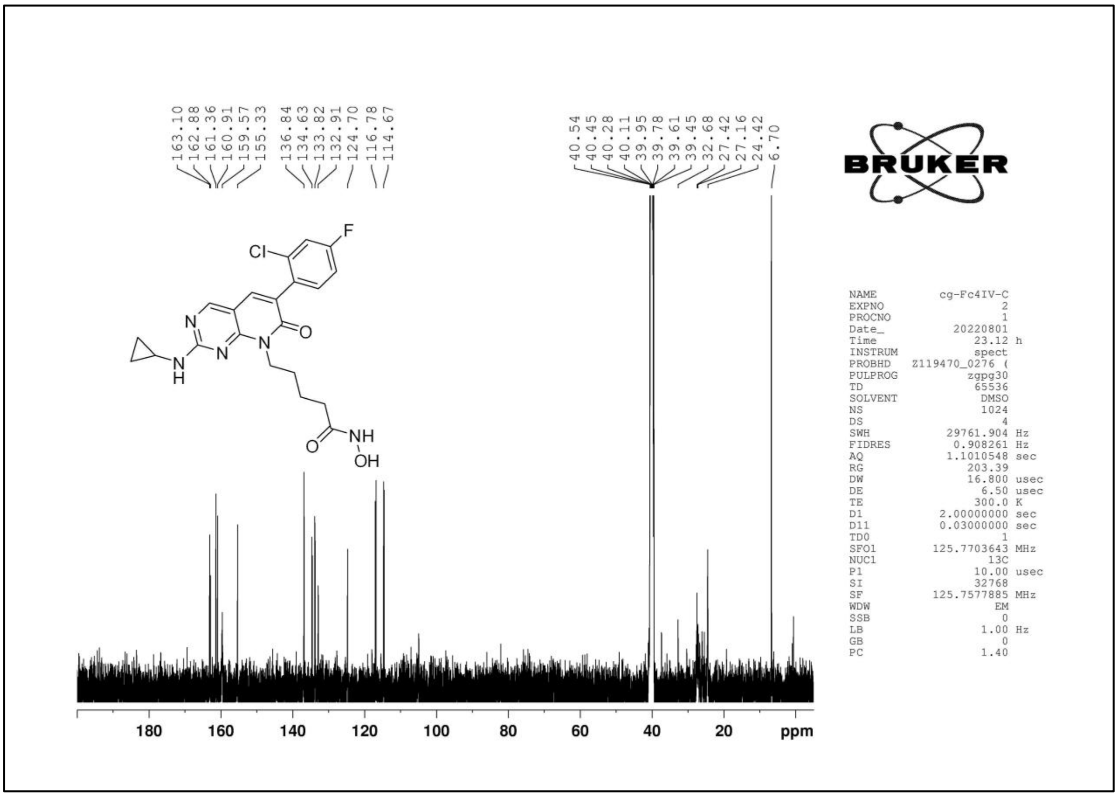

^{13}C-NMR spectrum of compound 22n

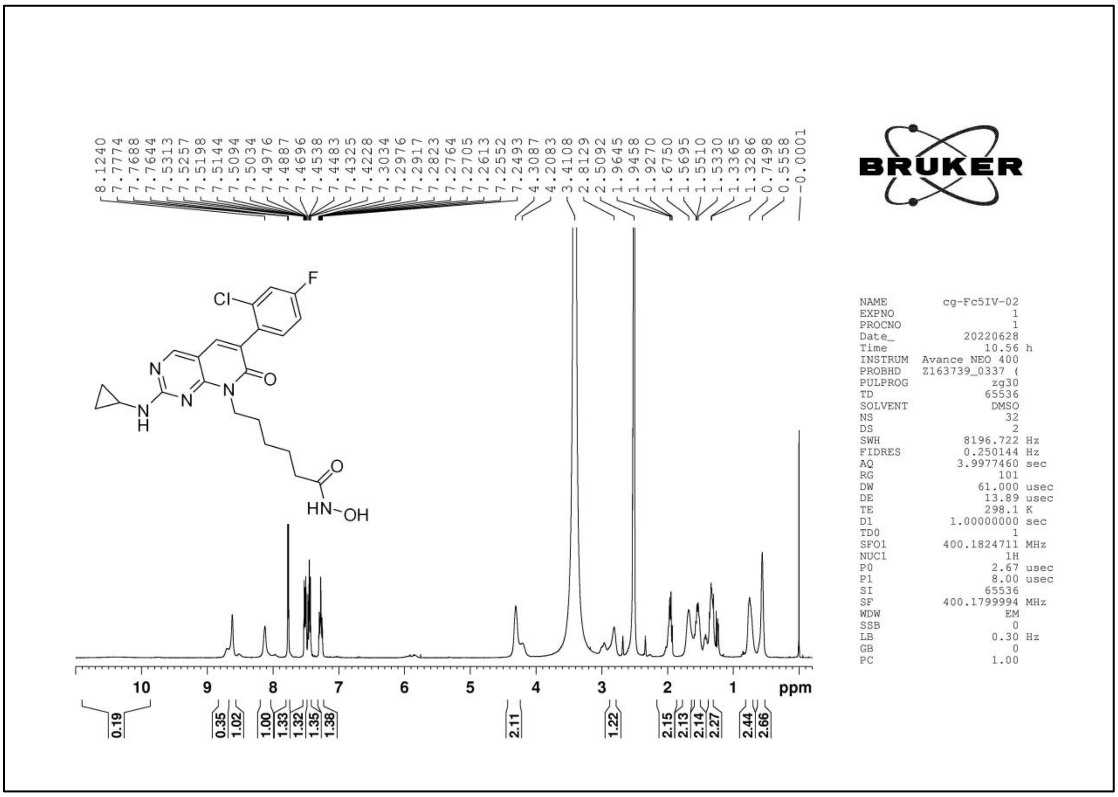

^{1}H-NMR spectrum of compound 22o

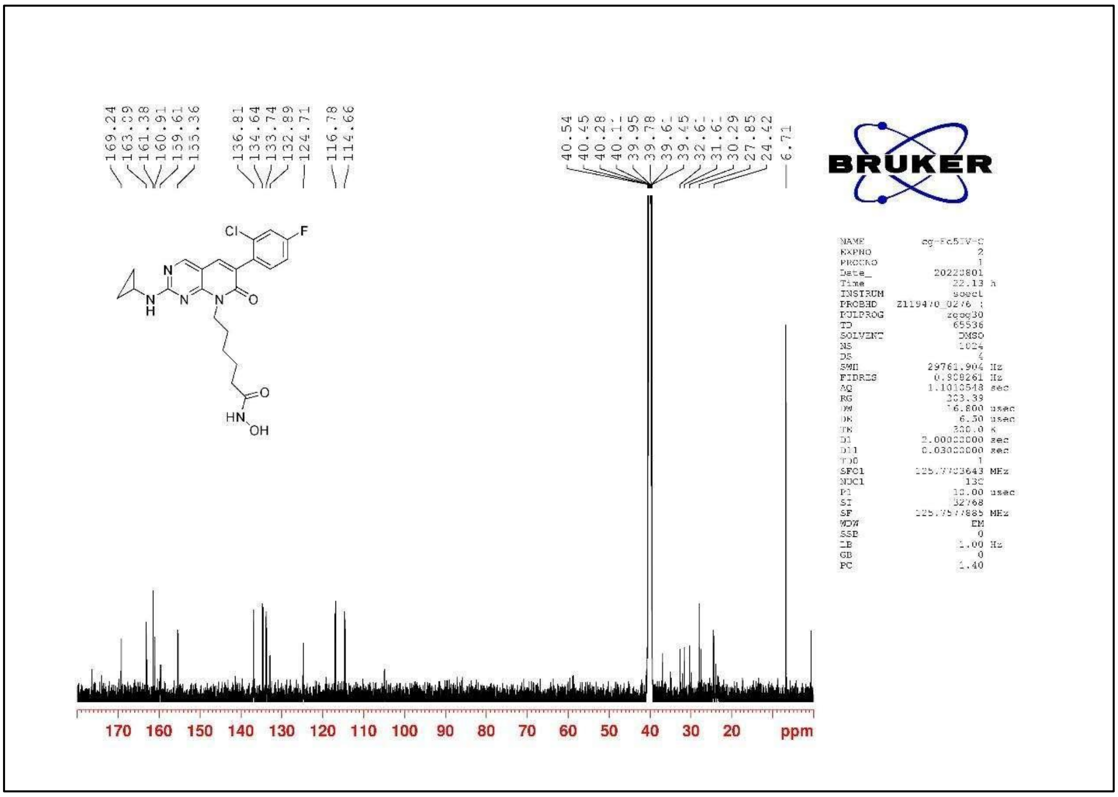

^{13}C-NMR spectrum of compound 22o

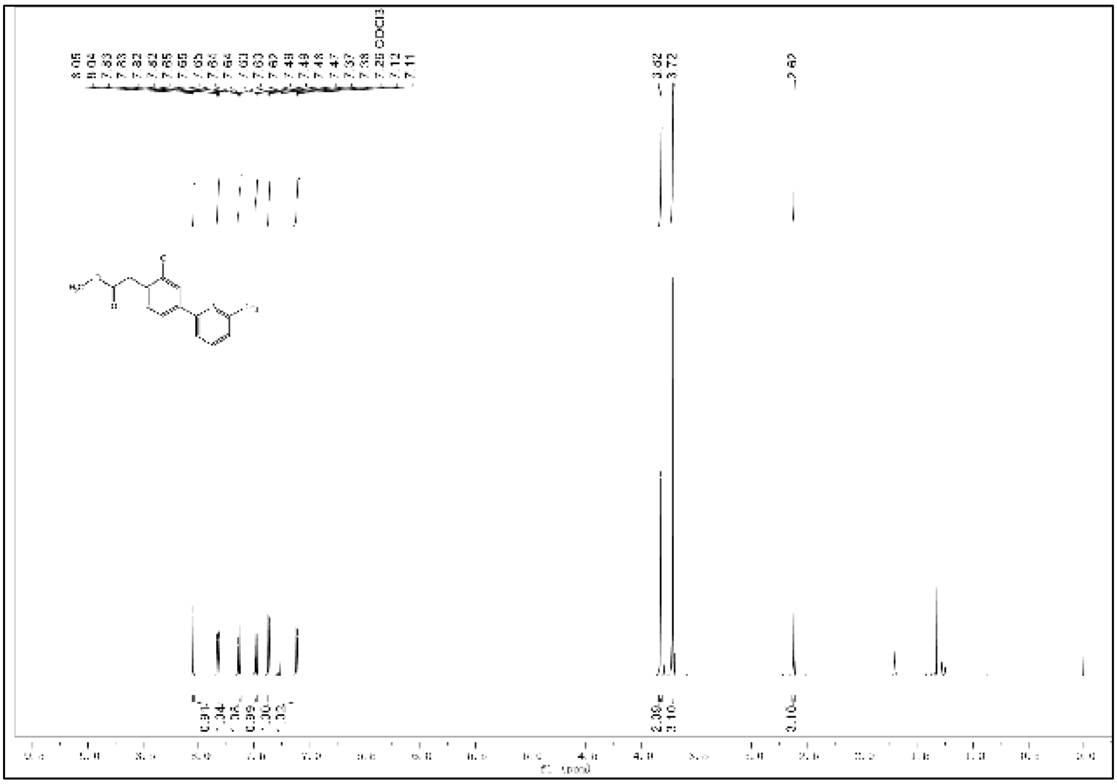

^{1}H-NMR spectrum of compound 27

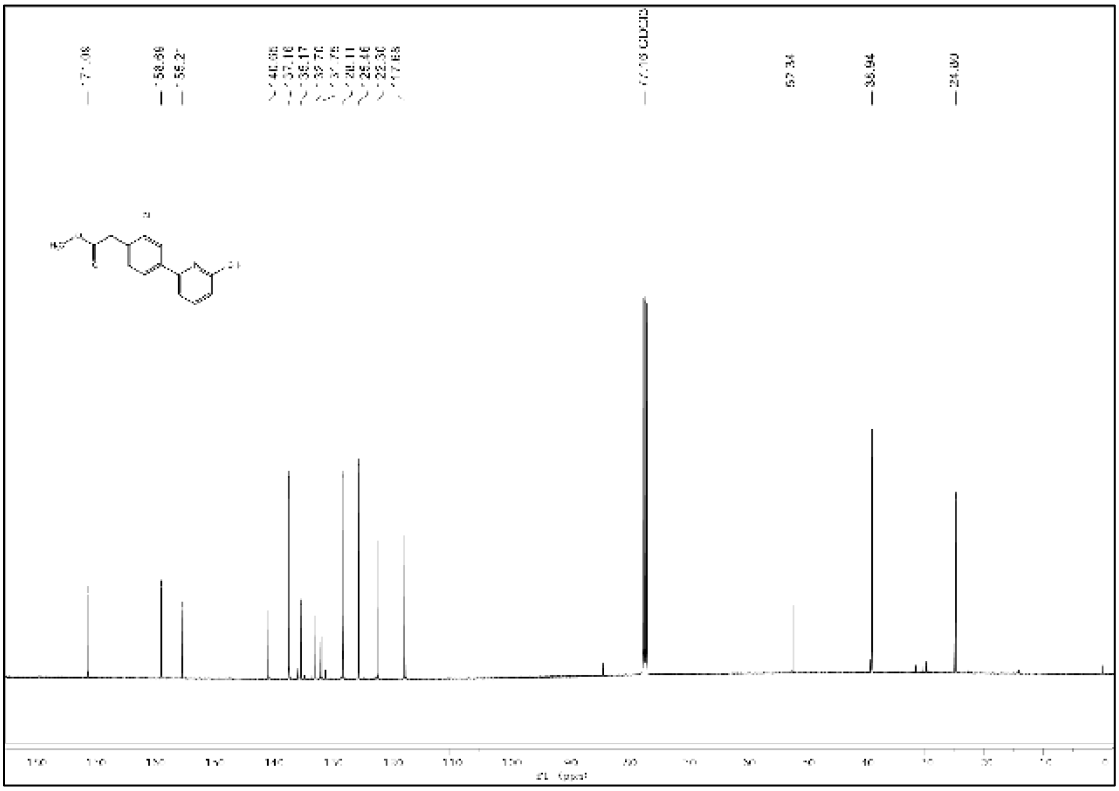

^{13}C-NMR spectrum of compound 27

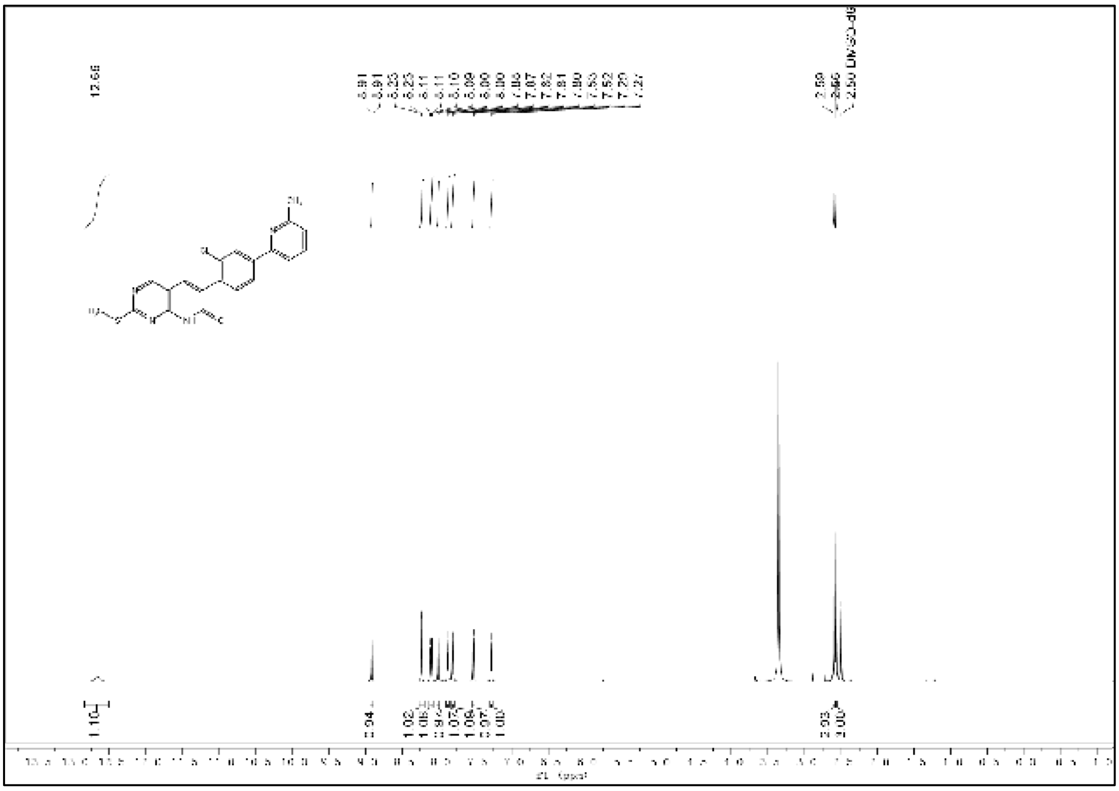

^{1}H-NMR spectrum of compound 28

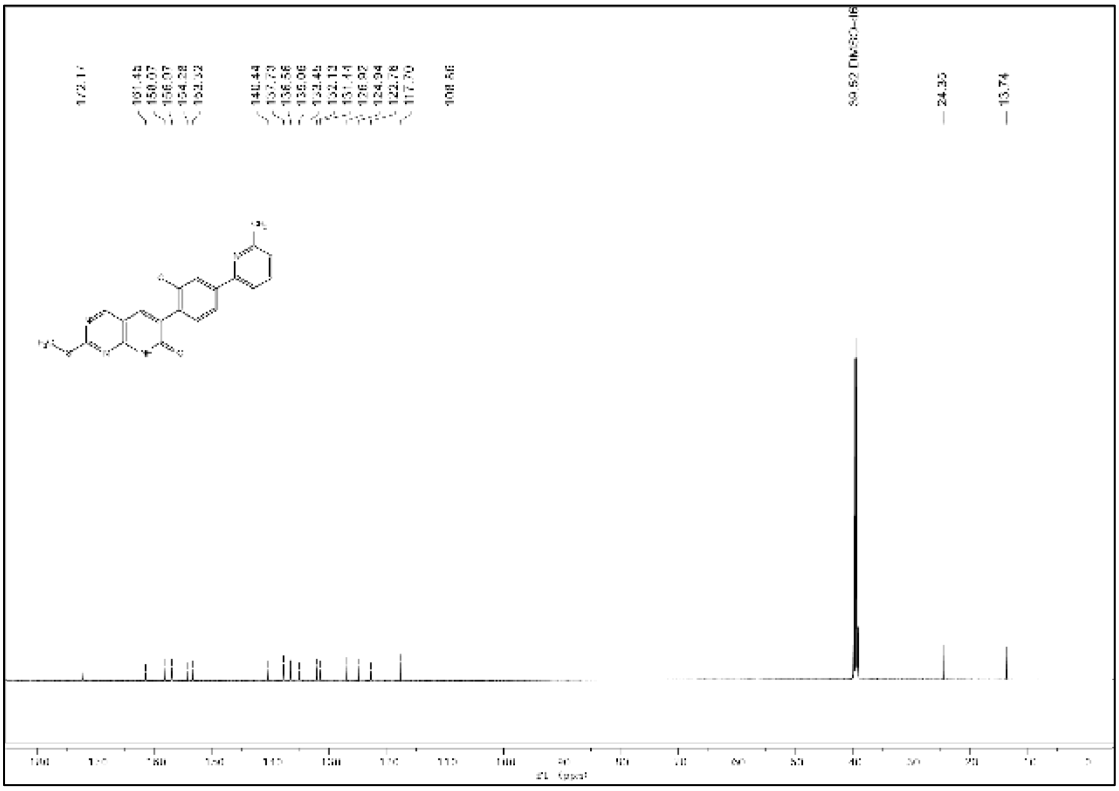

^{13}C-NMR spectrum of compound 28

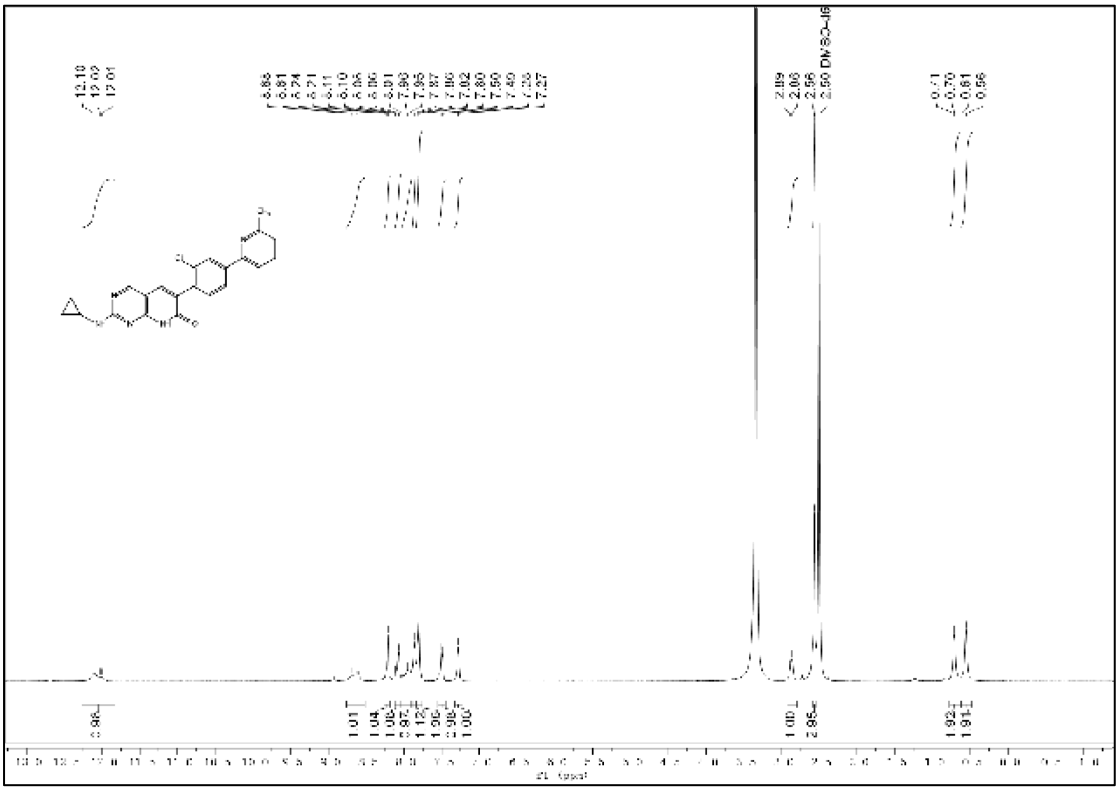

^{1}H-NMR spectrum of compound 30

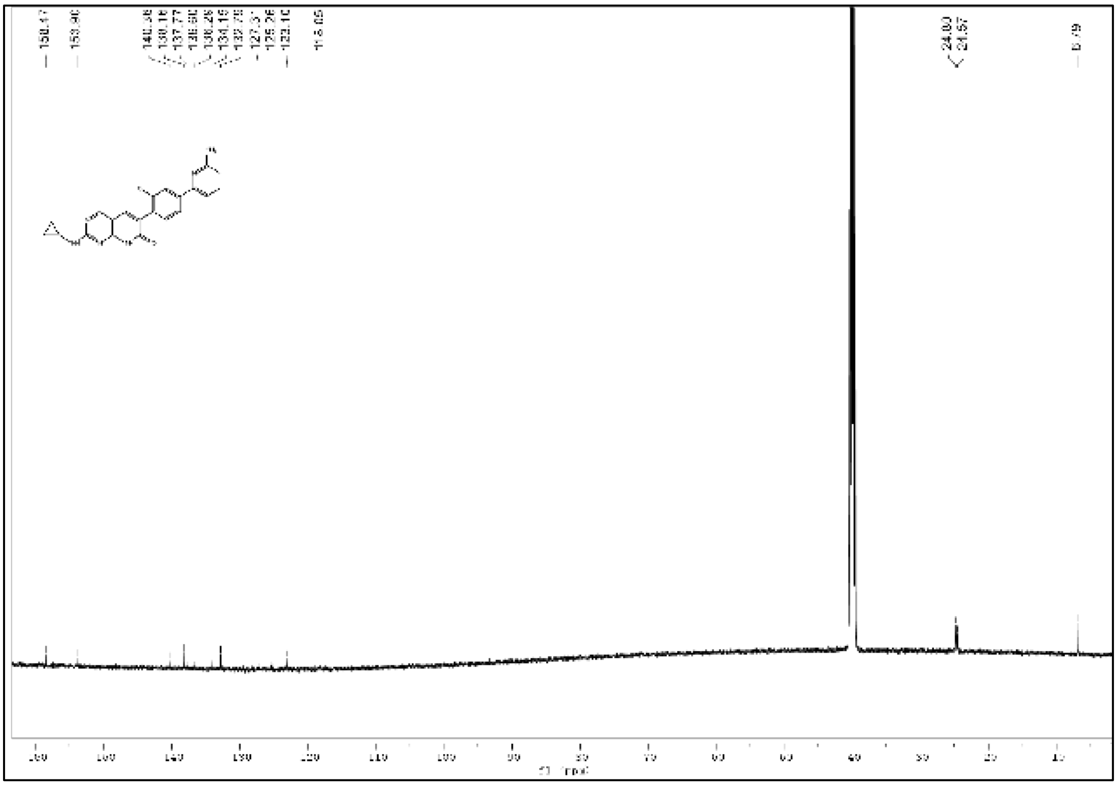

^{13}C-NMR spectrum of compound 30

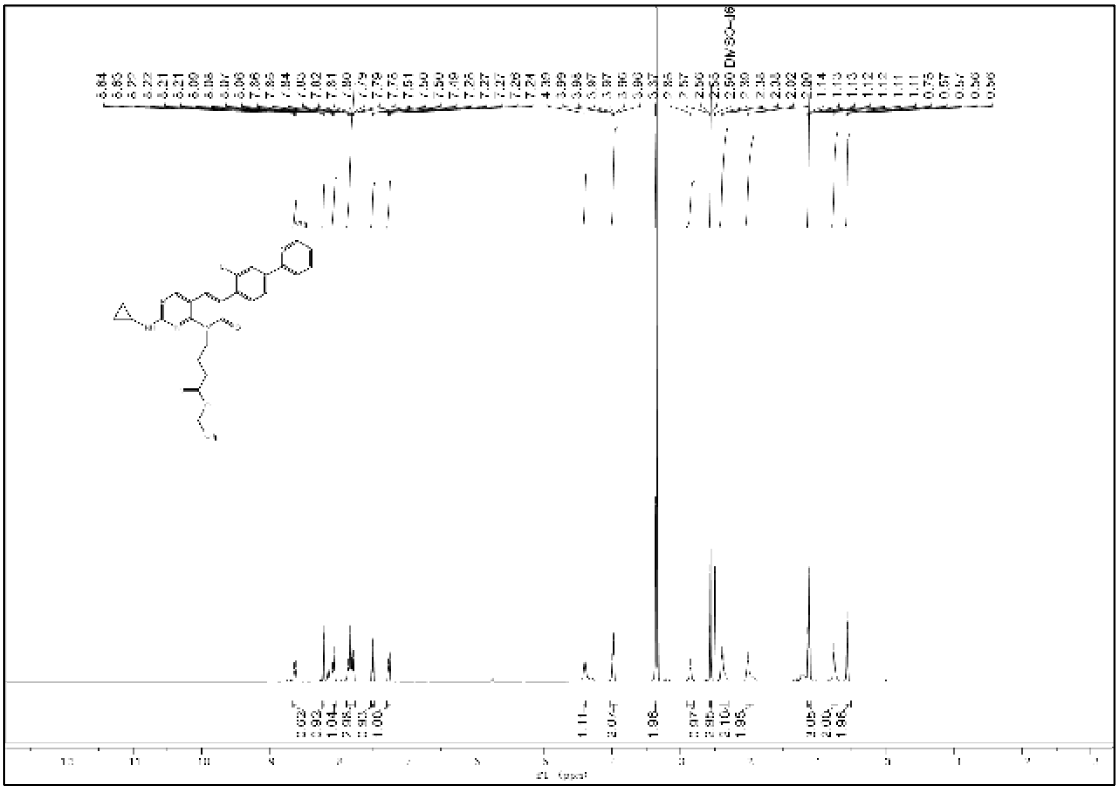

^{1}H-NMR spectrum of compound 31a

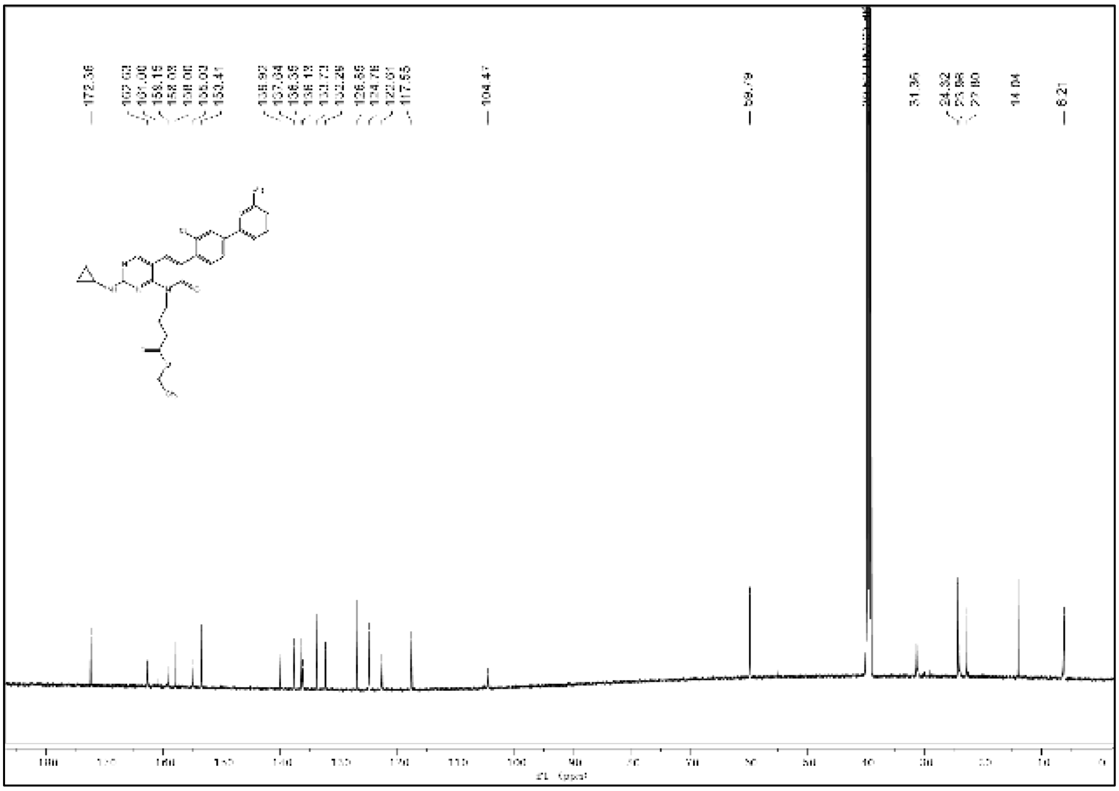

^{13}C-NMR spectrum of compound 31a

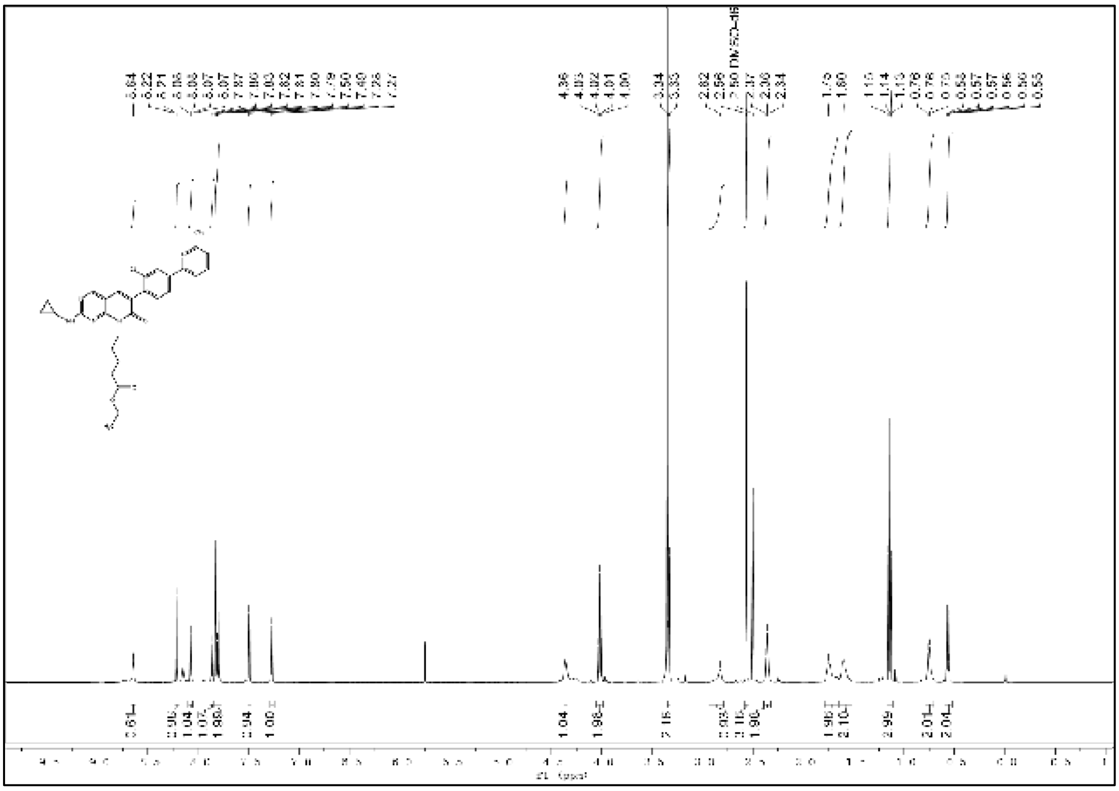

^{1}H-NMR spectrum of compound 31b

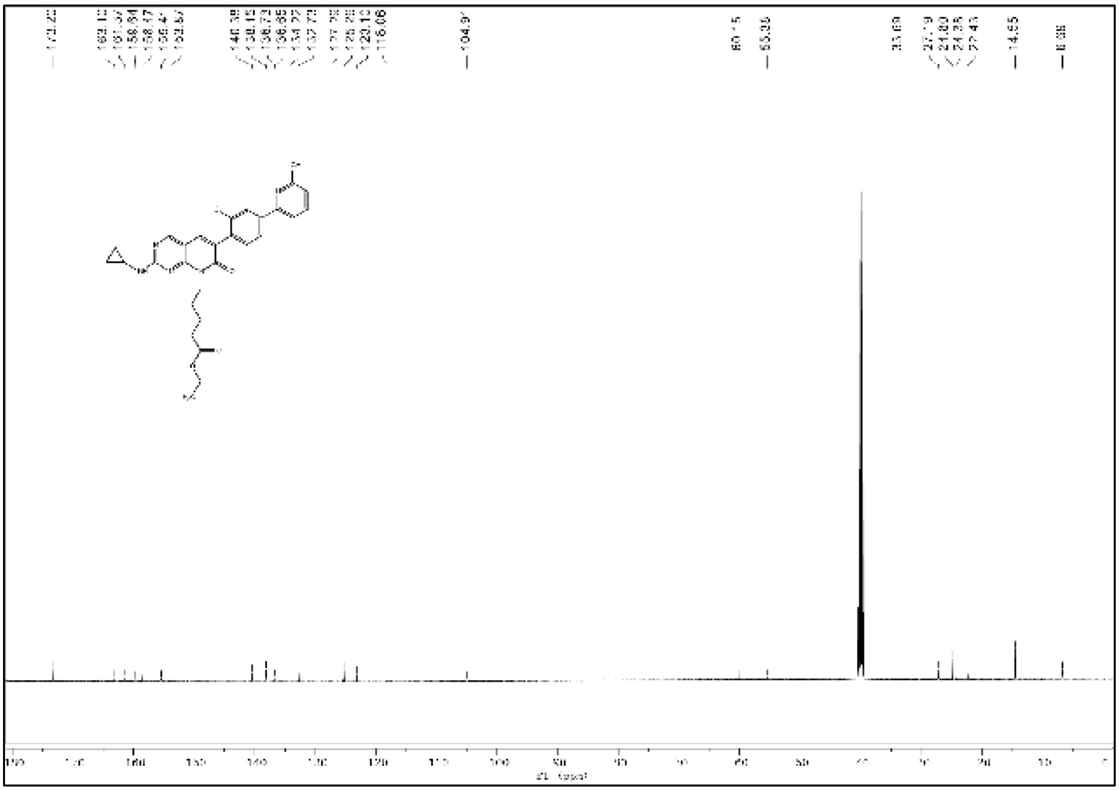

^{13}C-NMR spectrum of compound 31b

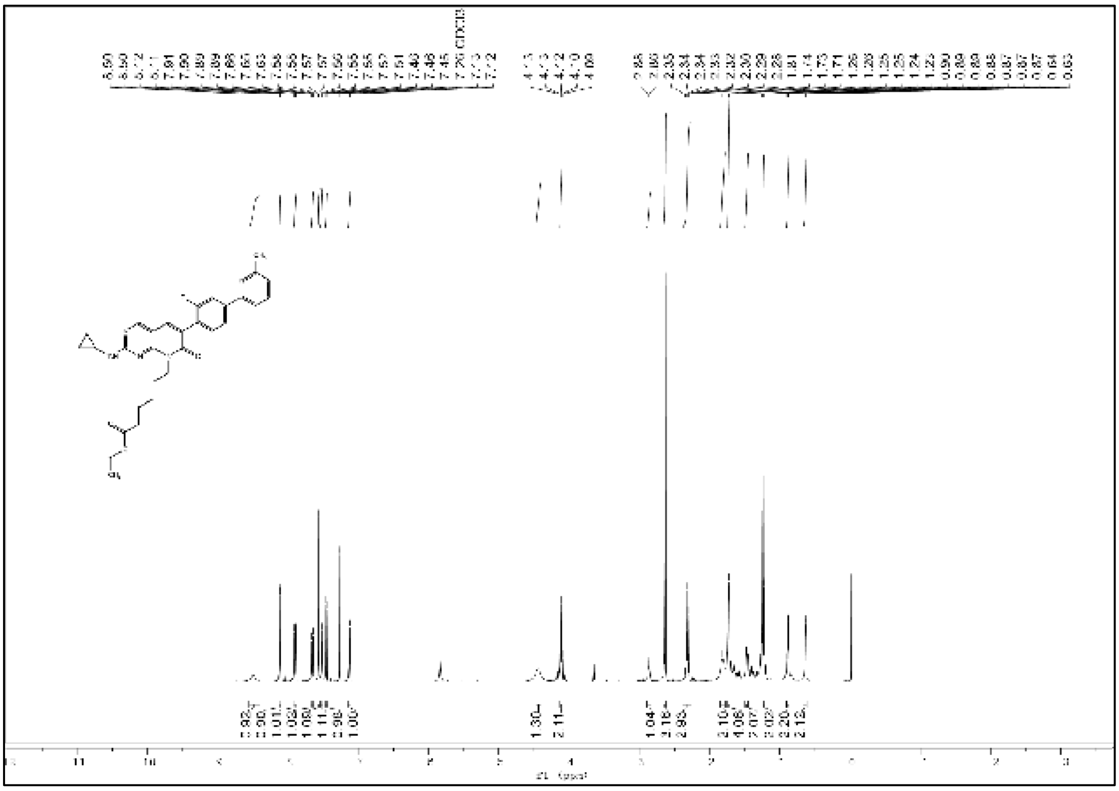

^{1}H-NMR spectrum of compound 31c

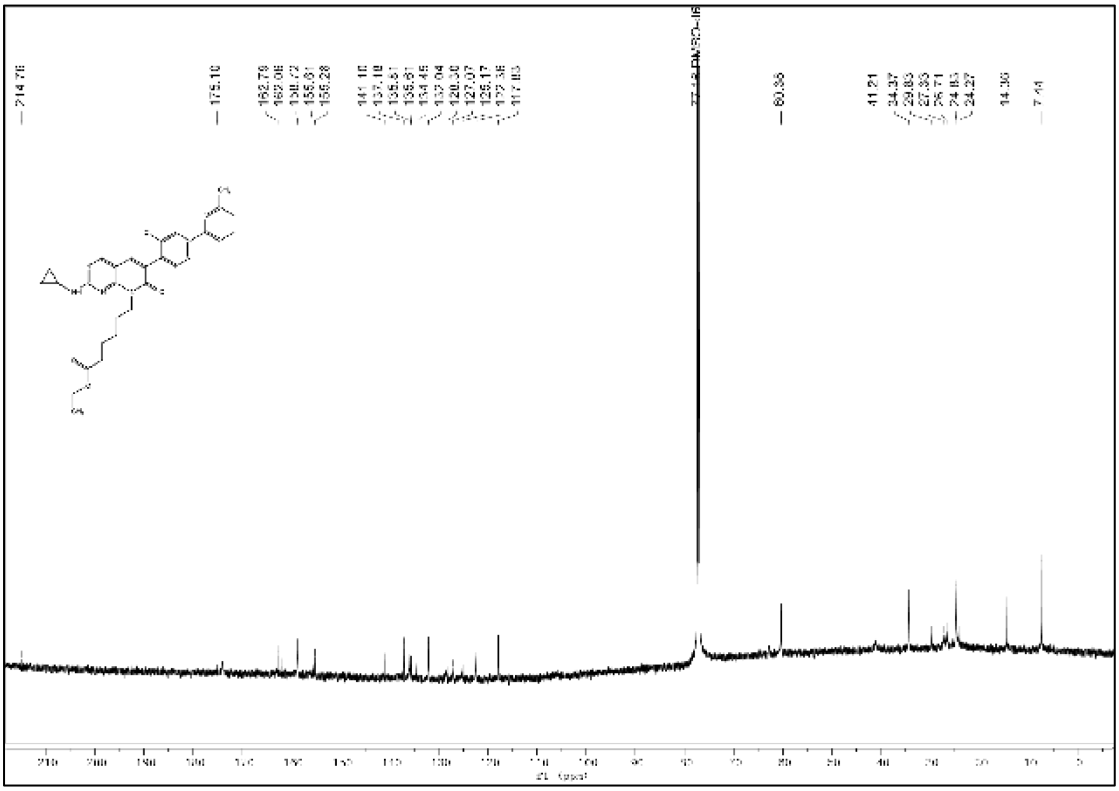

^{13}C-NMR spectrum of compound 31c

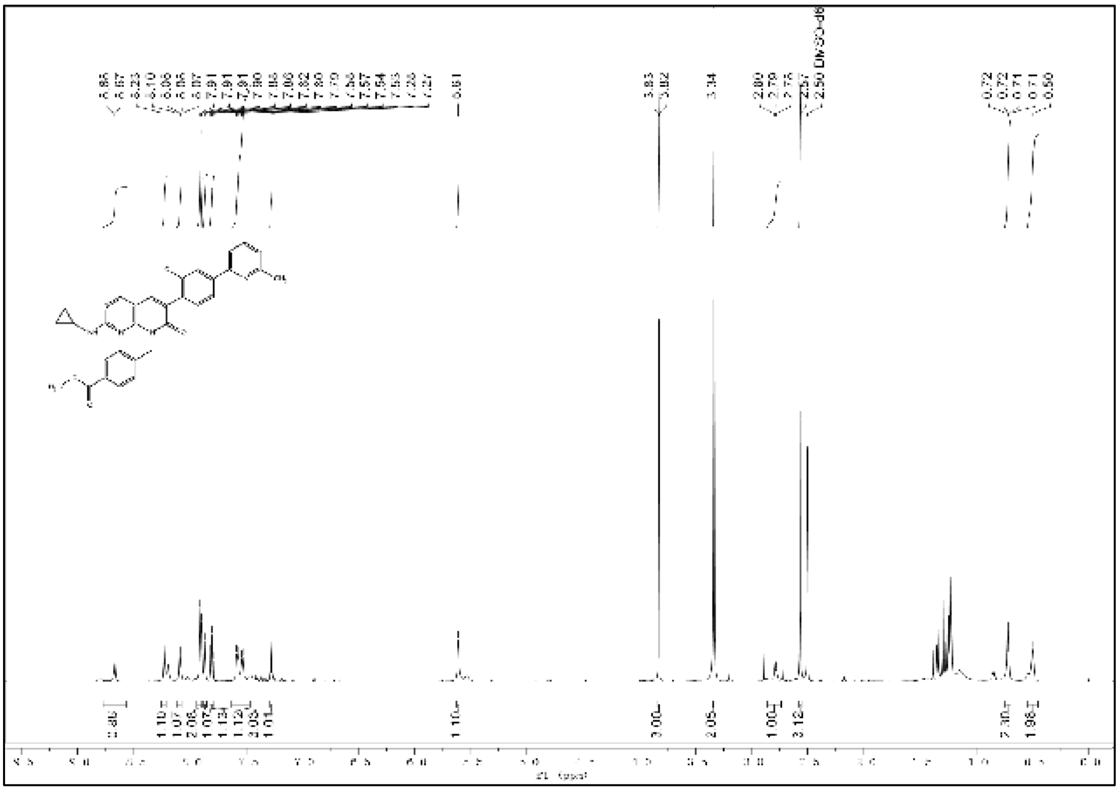

^{1}H-NMR spectrum of compound 31d

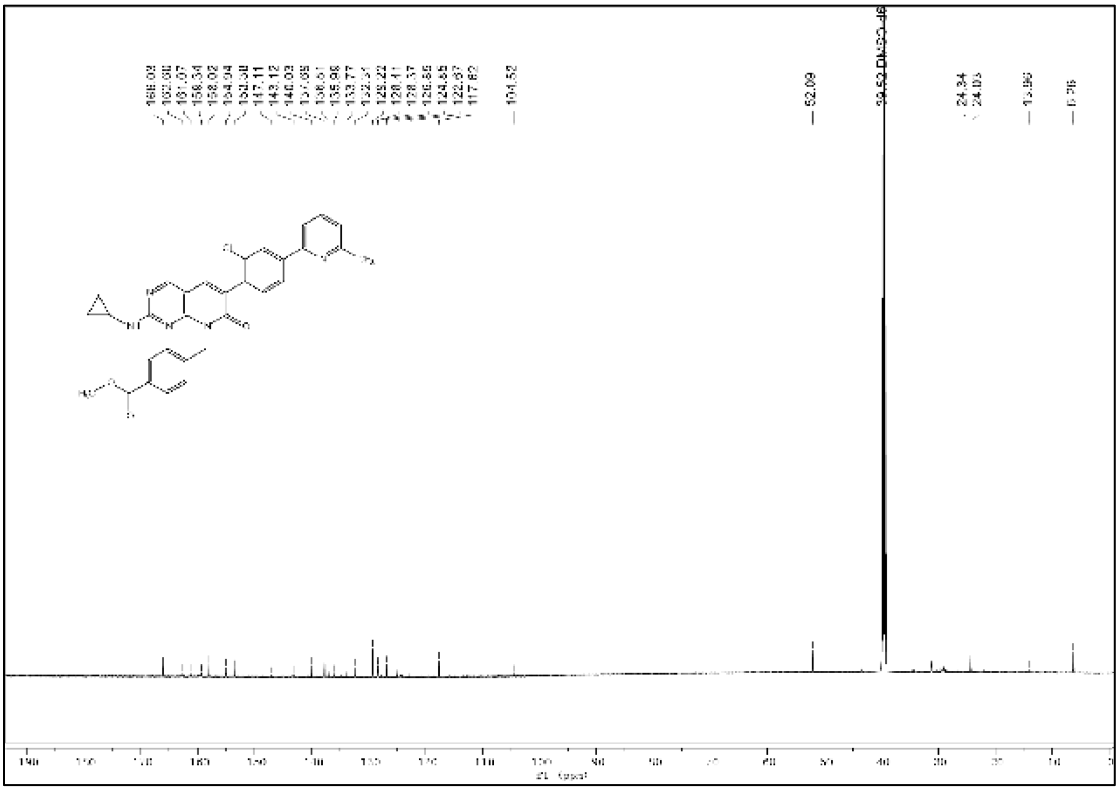

^{13}C-NMR spectrum of compound 31d

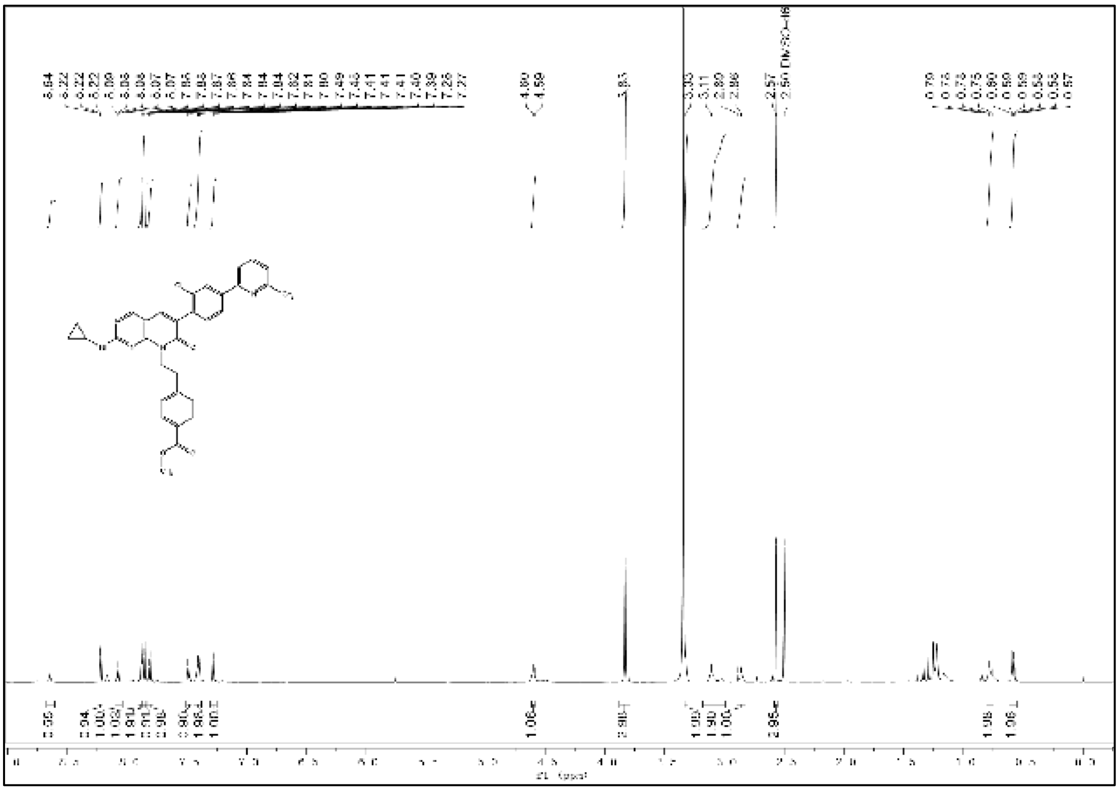

^{1}H-NMR spectrum of compound 31e

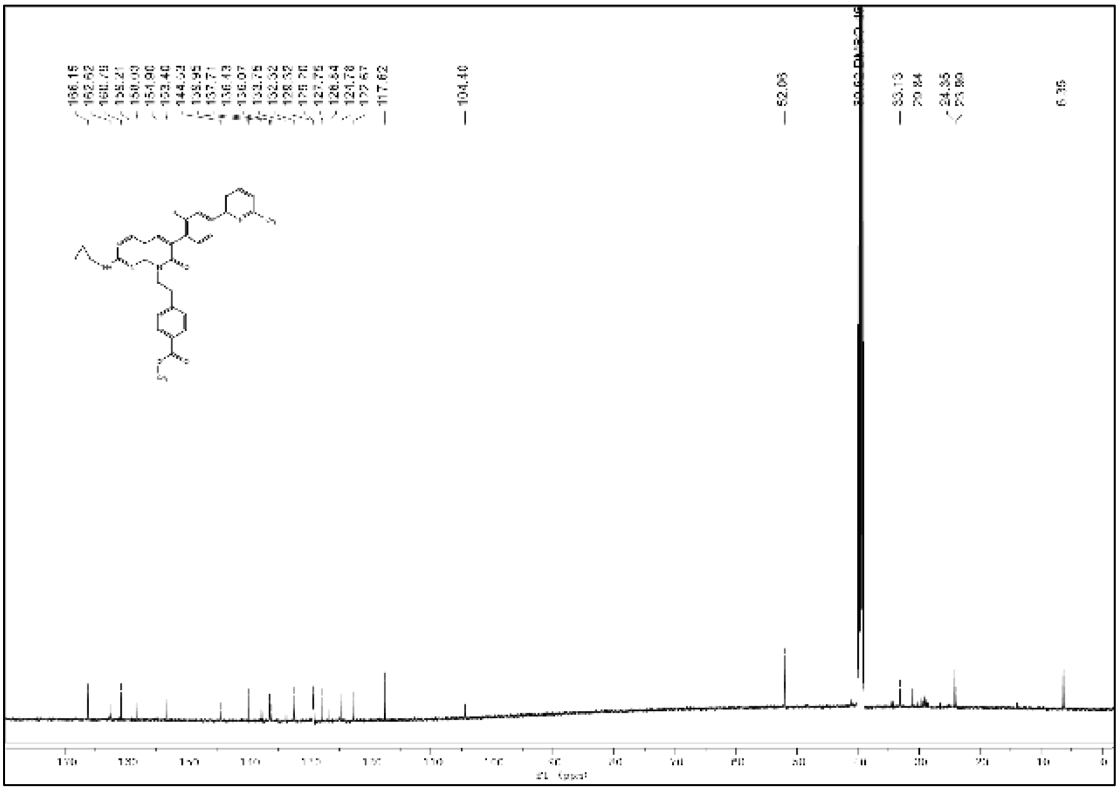

^{13}C-NMR spectrum of compound 31e

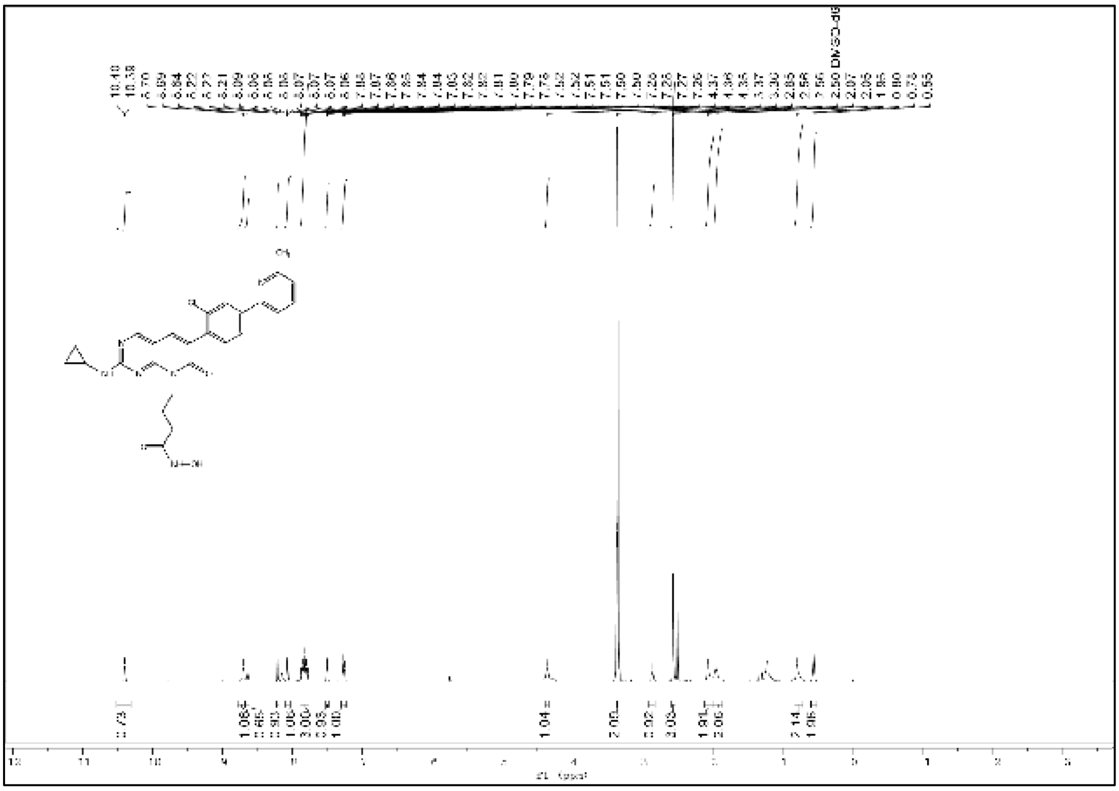

^{1}H-NMR spectrum of compound 32a

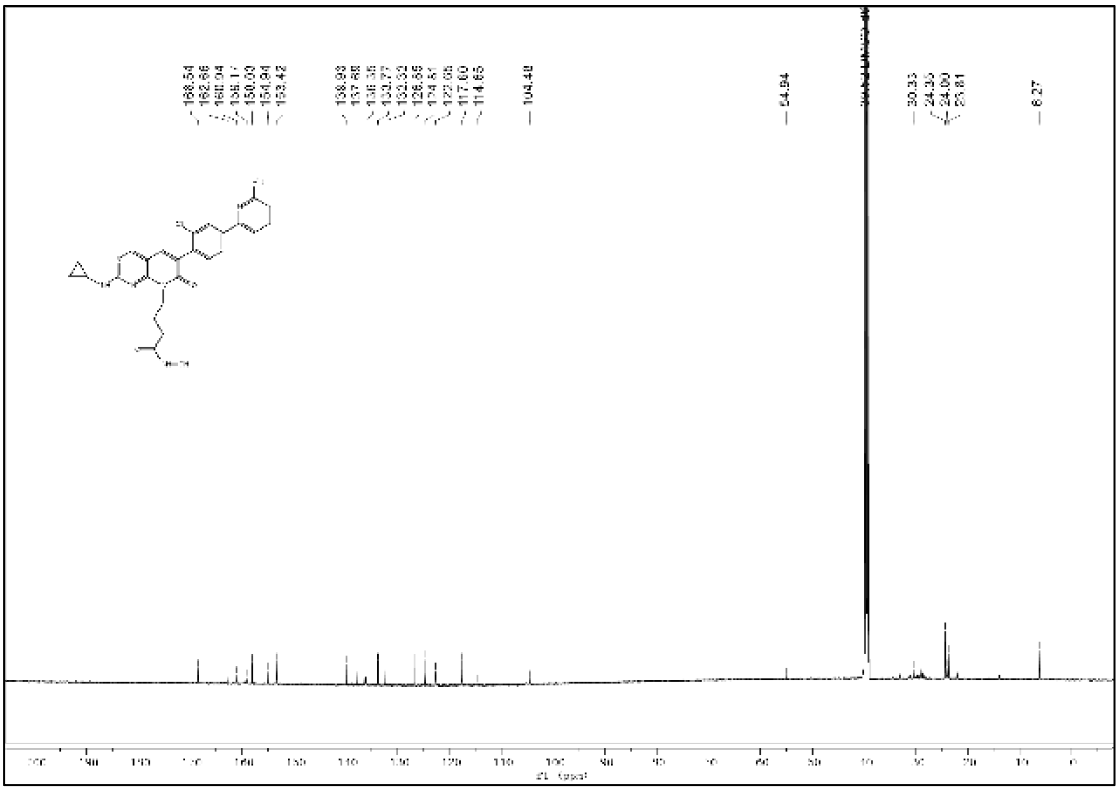

^{13}C-NMR spectrum of compound 32a

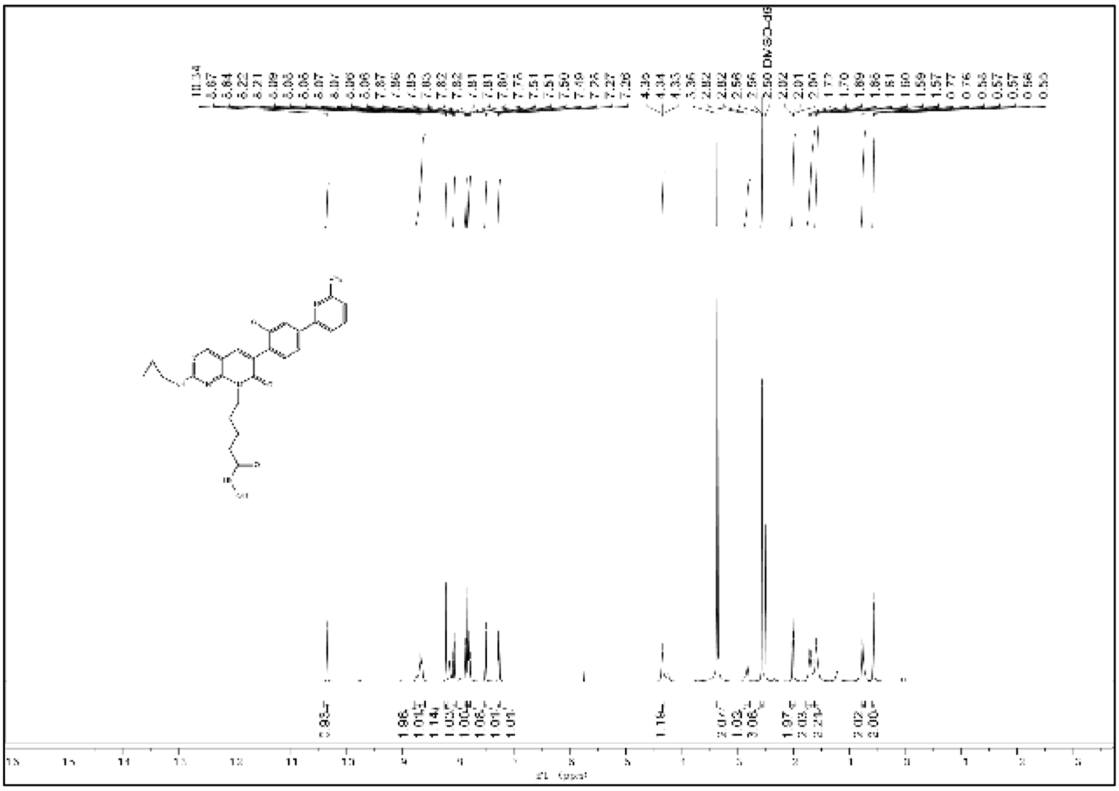

^{1}H-NMR spectrum of compound 32b

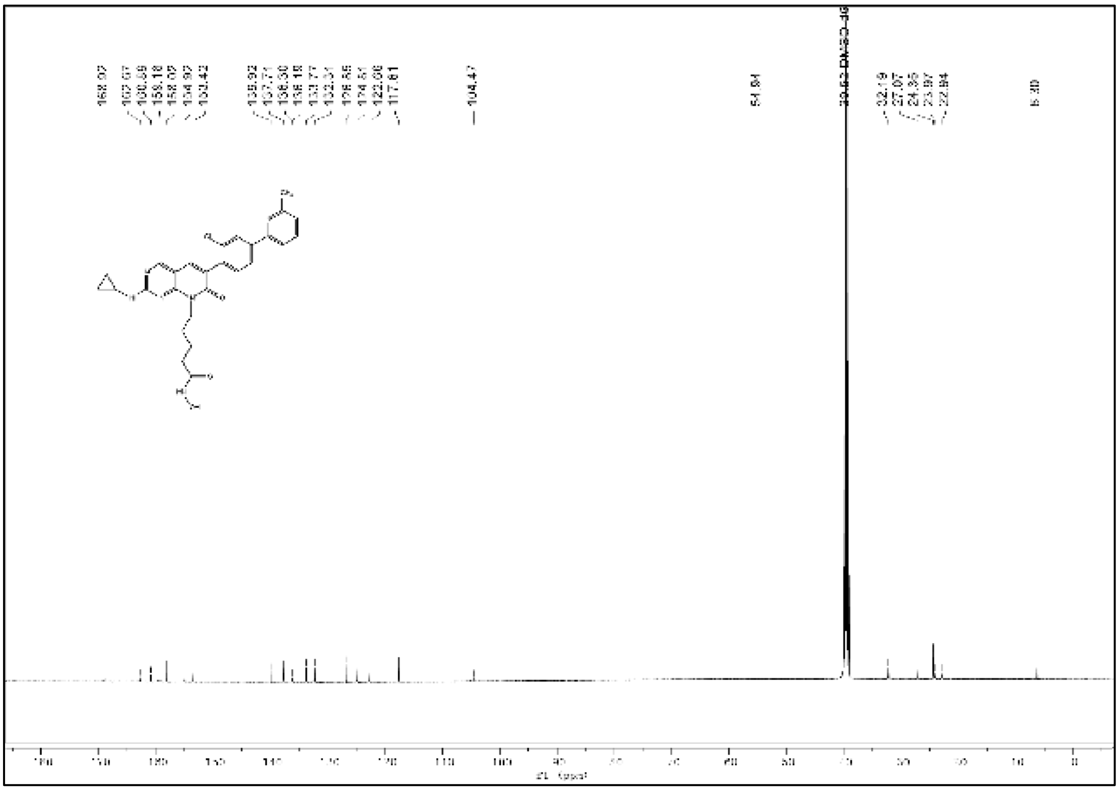

^{13}C-NMR spectrum of compound 32b

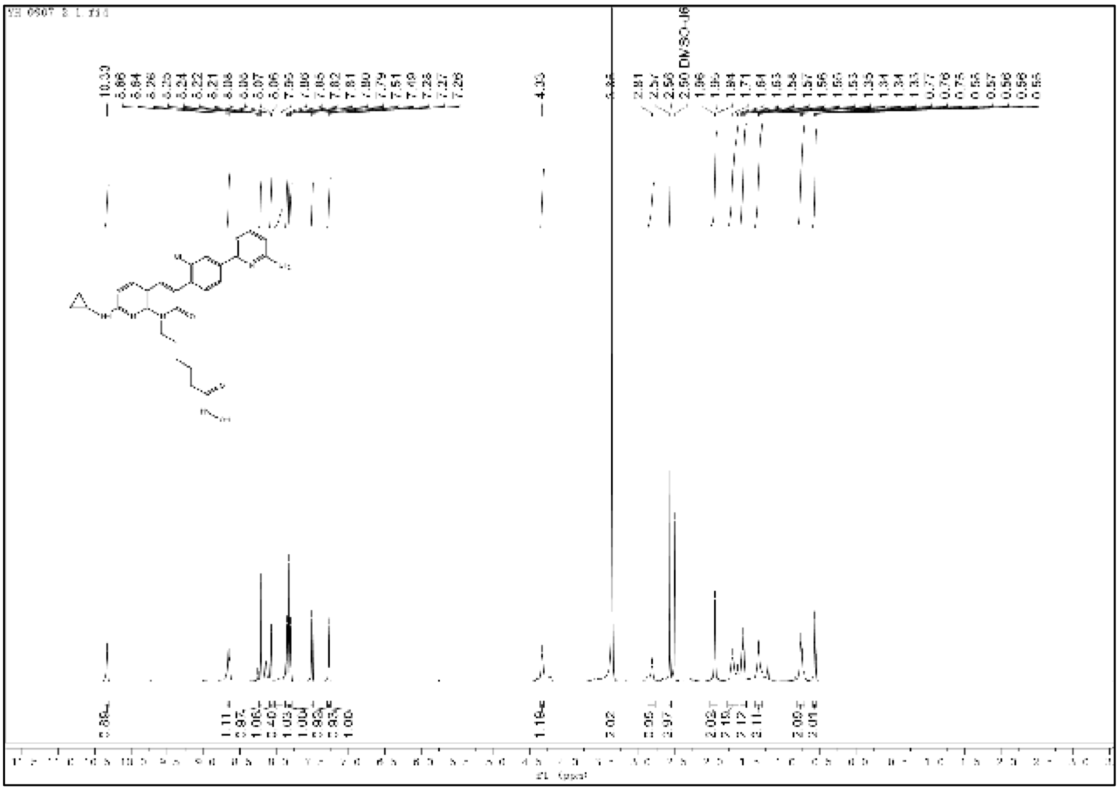

^{1}H-NMR spectrum of compound 32c

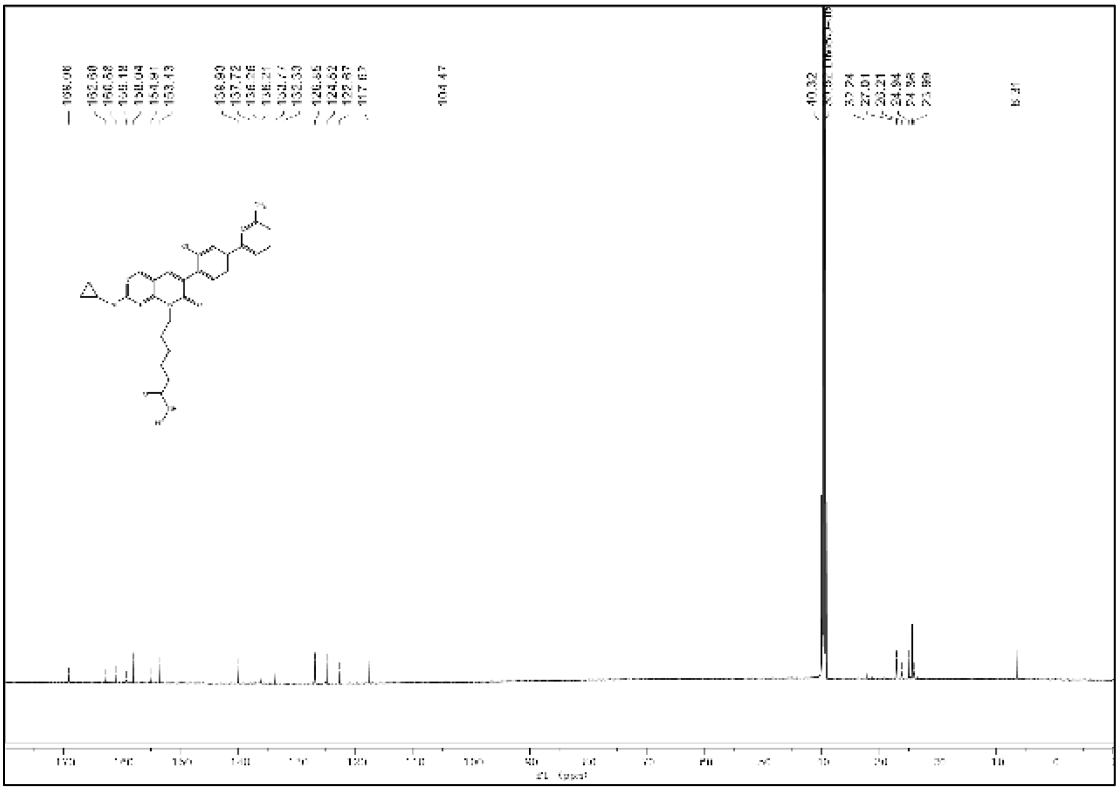

^{13}C-NMR spectrum of compound 32c

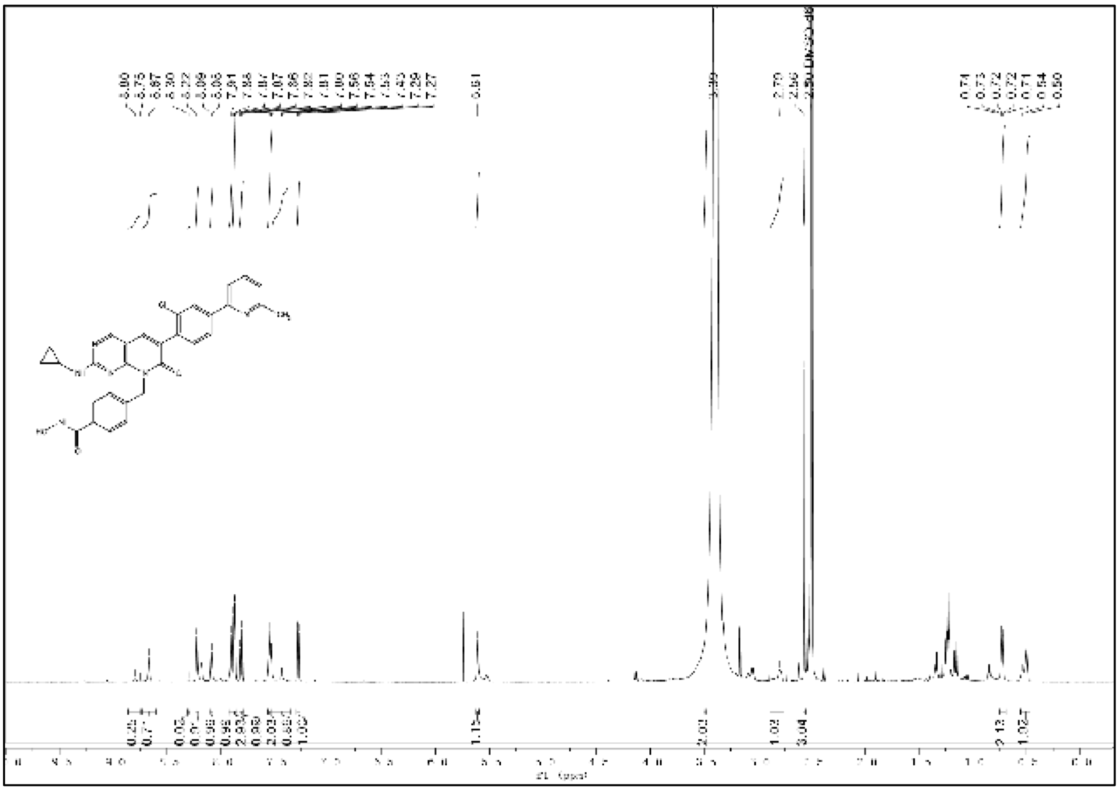

^{1}H-NMR spectrum of compound 32d

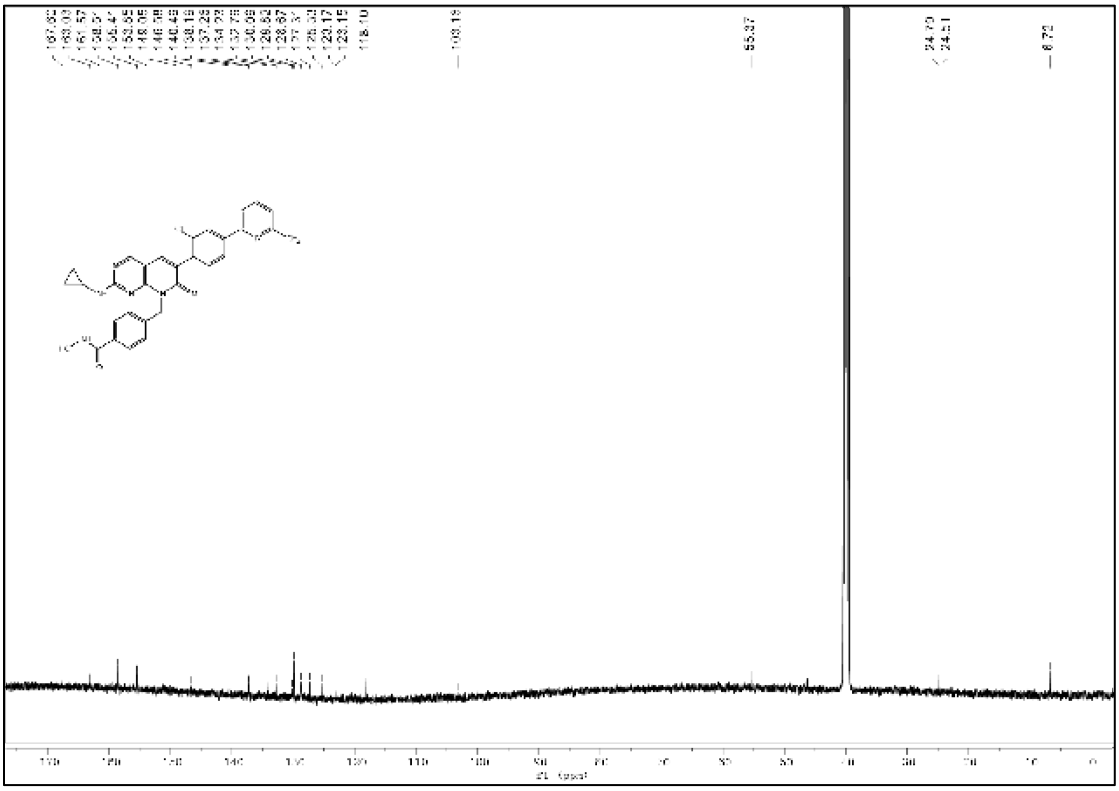

^{13}C-NMR spectrum of compound 32d

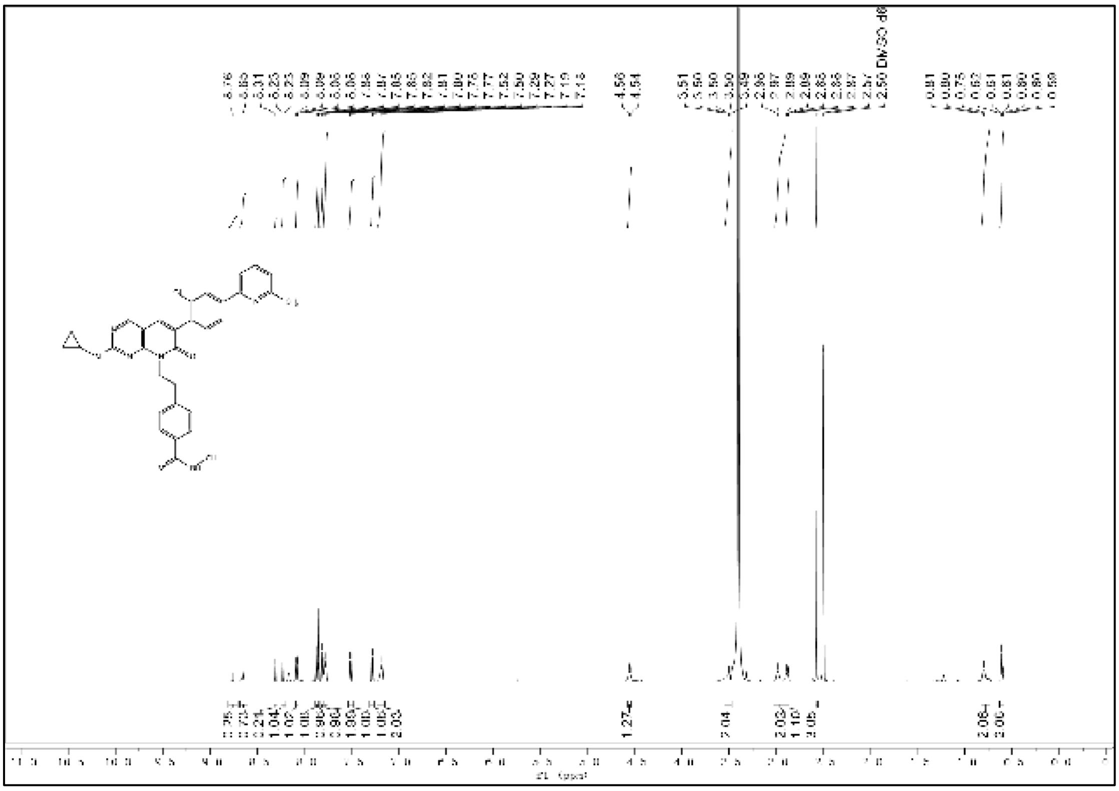

^{1}H-NMR spectrum of compound 32e

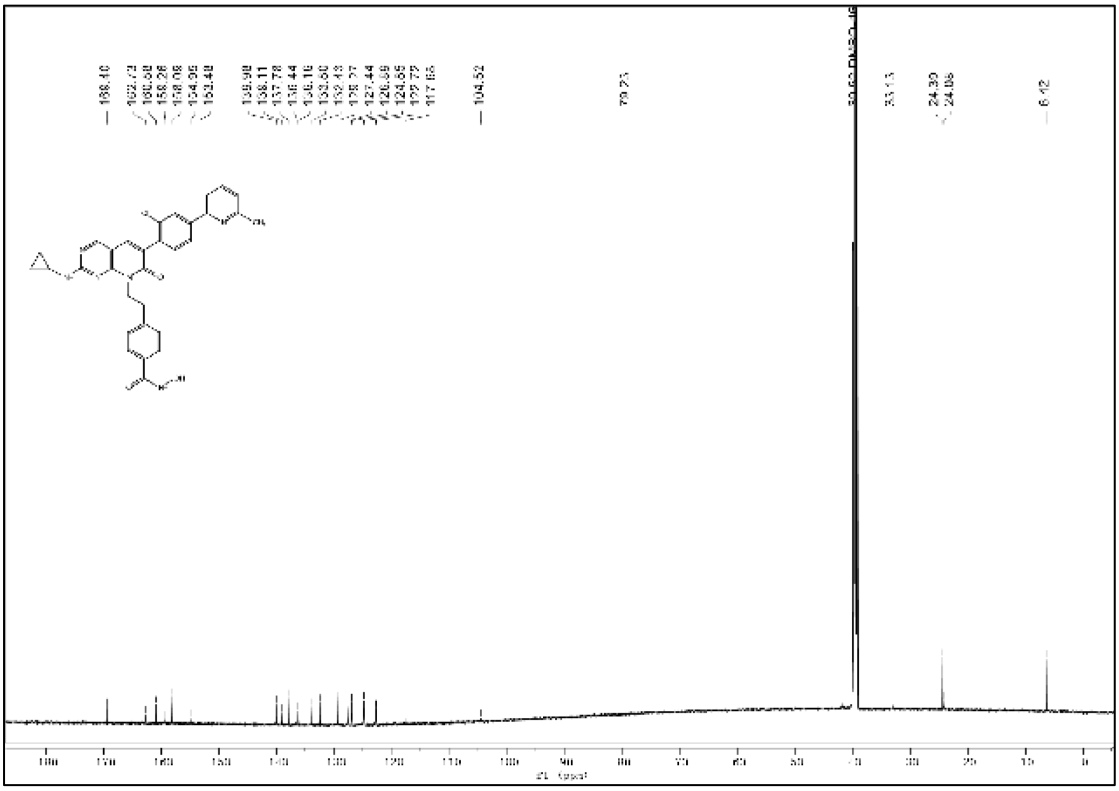

^{13}C-NMR spectrum of compound 32e

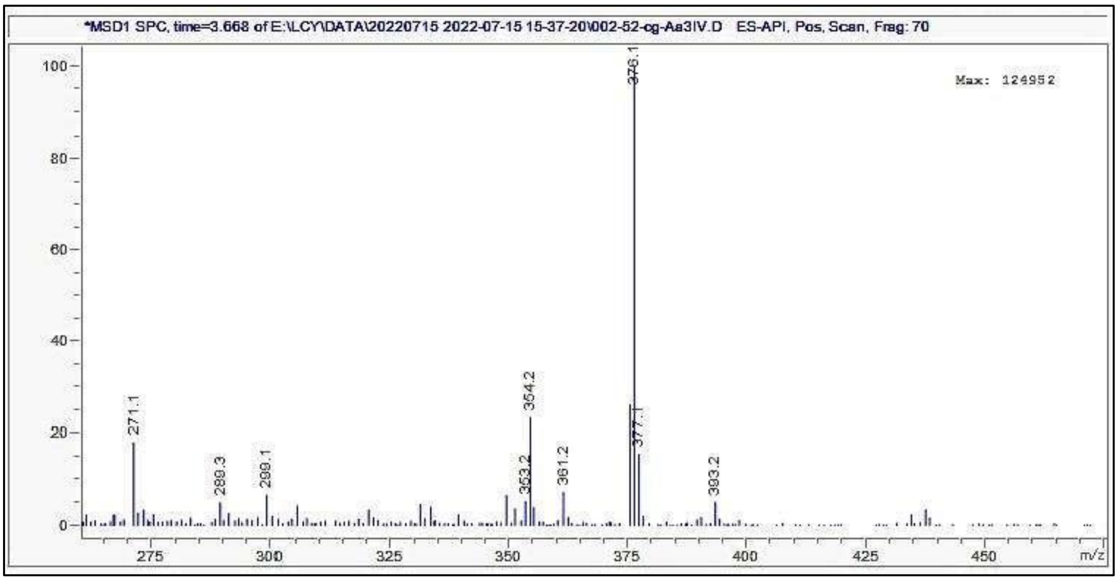

LCMS spectrum of compound 20a

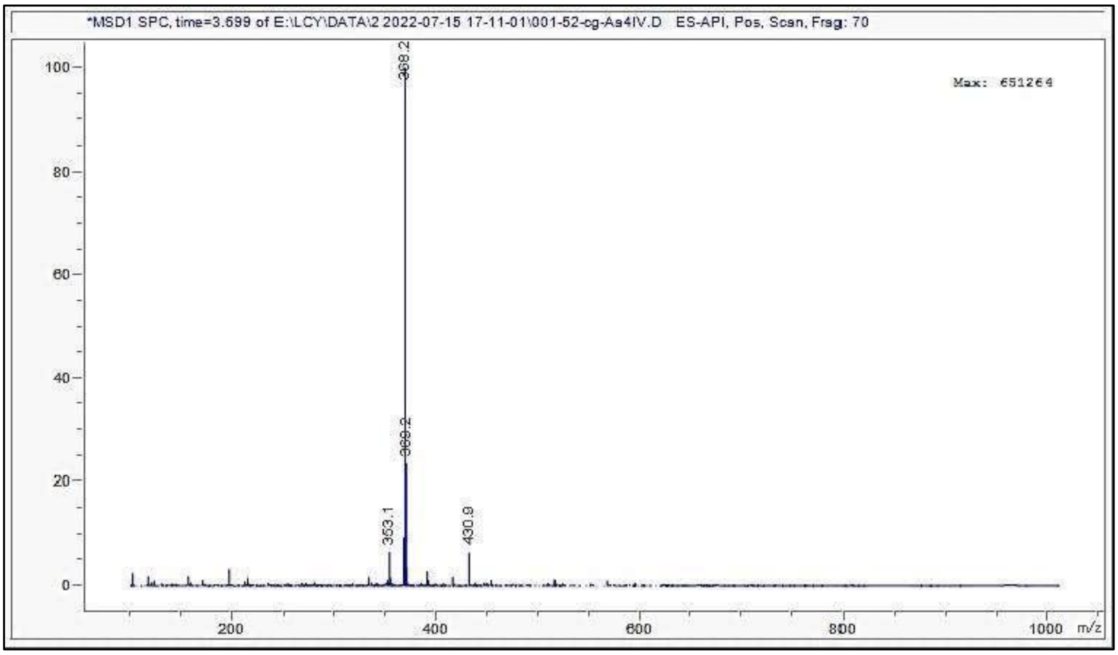

LCMS spectrum of compound 20b

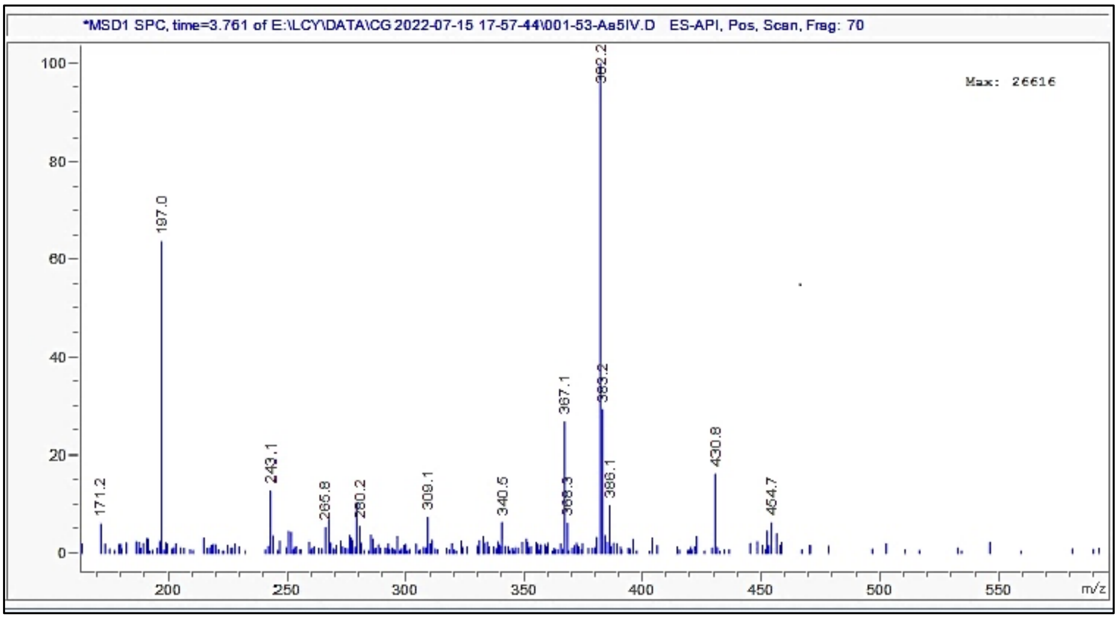

LCMS spectrum of compound 20c

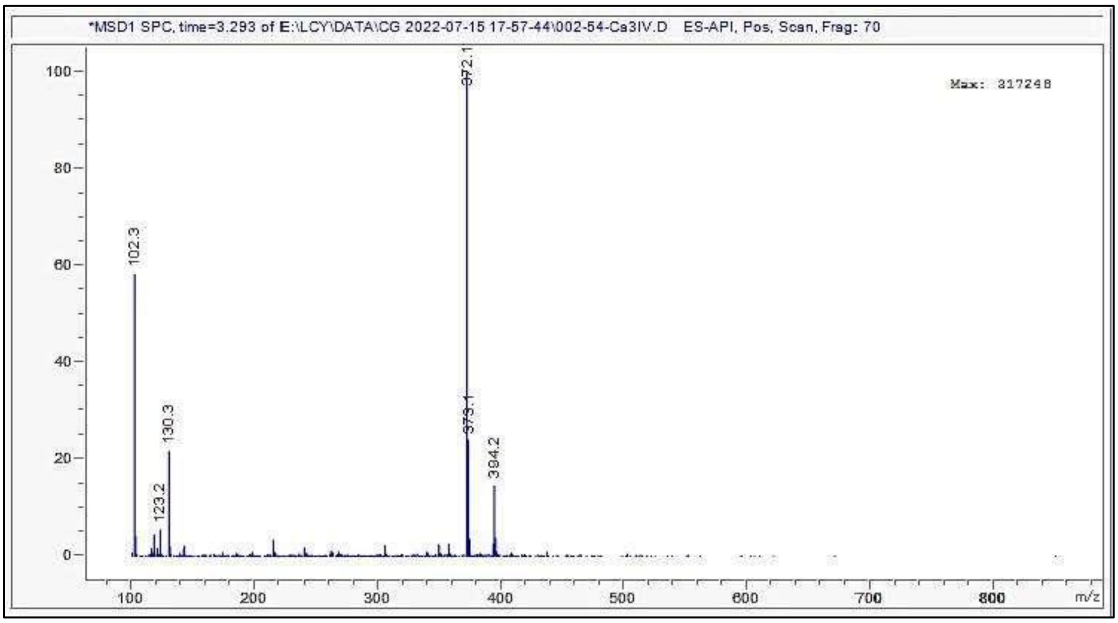

LCMS spectrum of compound 20d

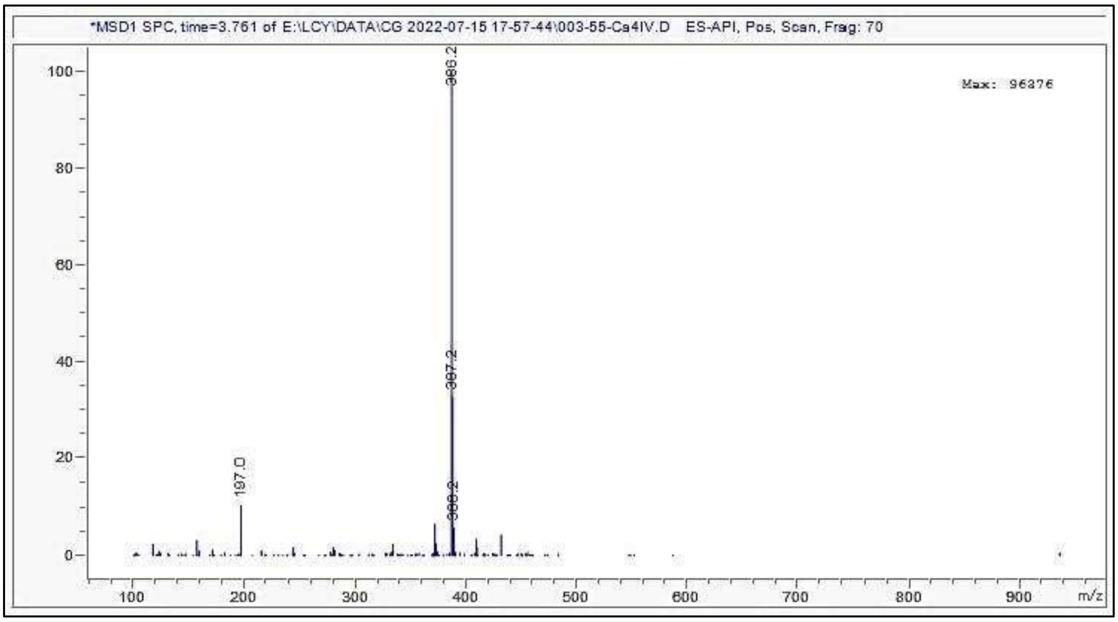

LCMS spectrum of compound 20e

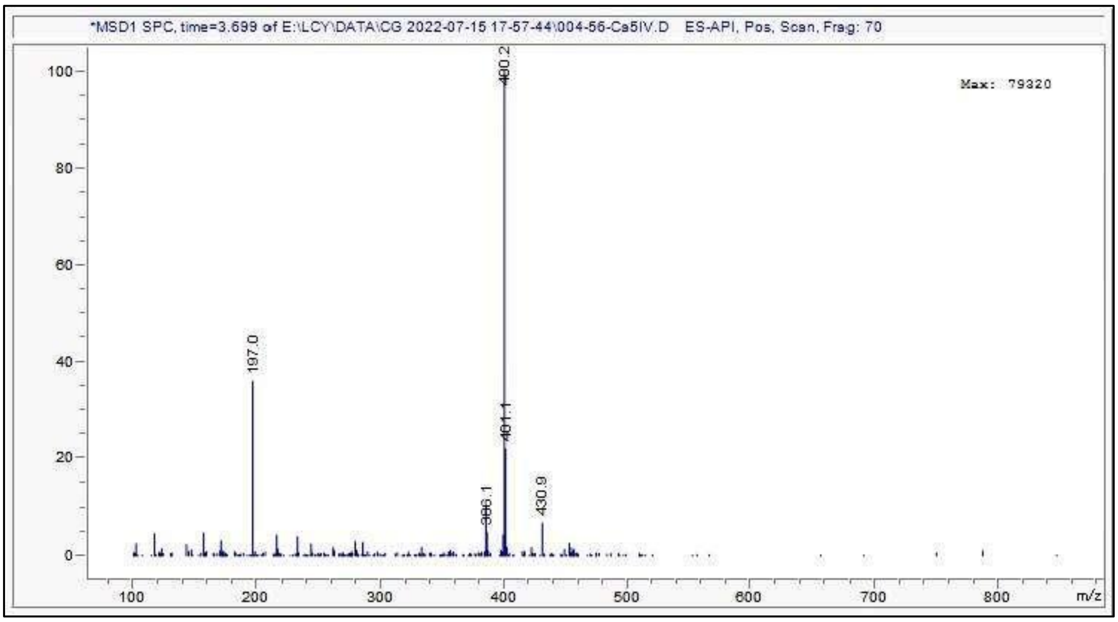

LCMS spectrum of compound 20f

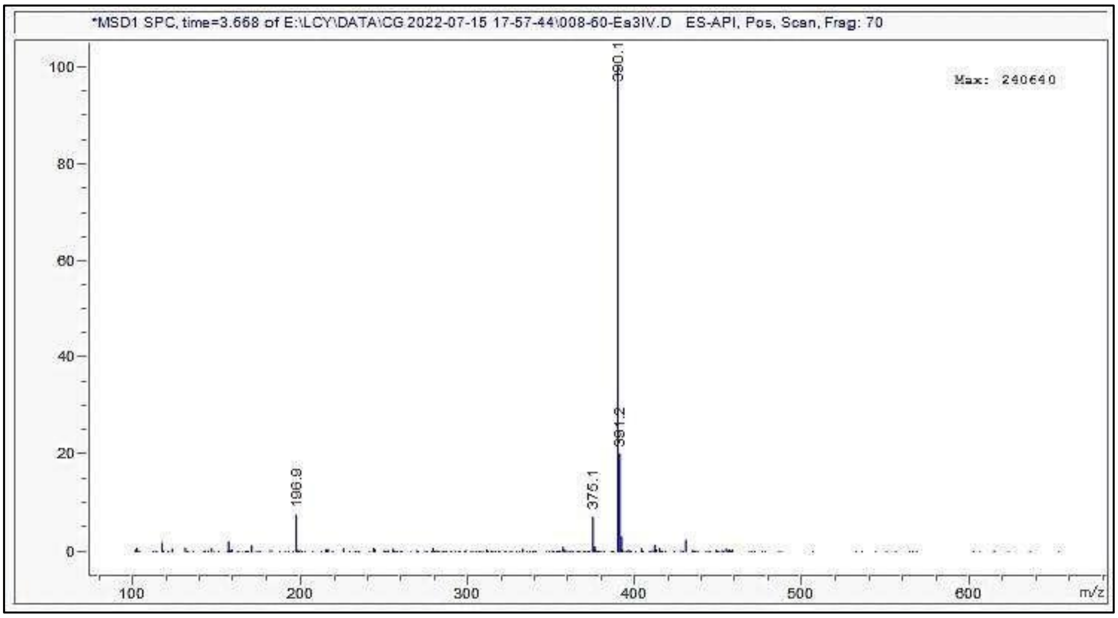

LCMS spectrum of compound 20g

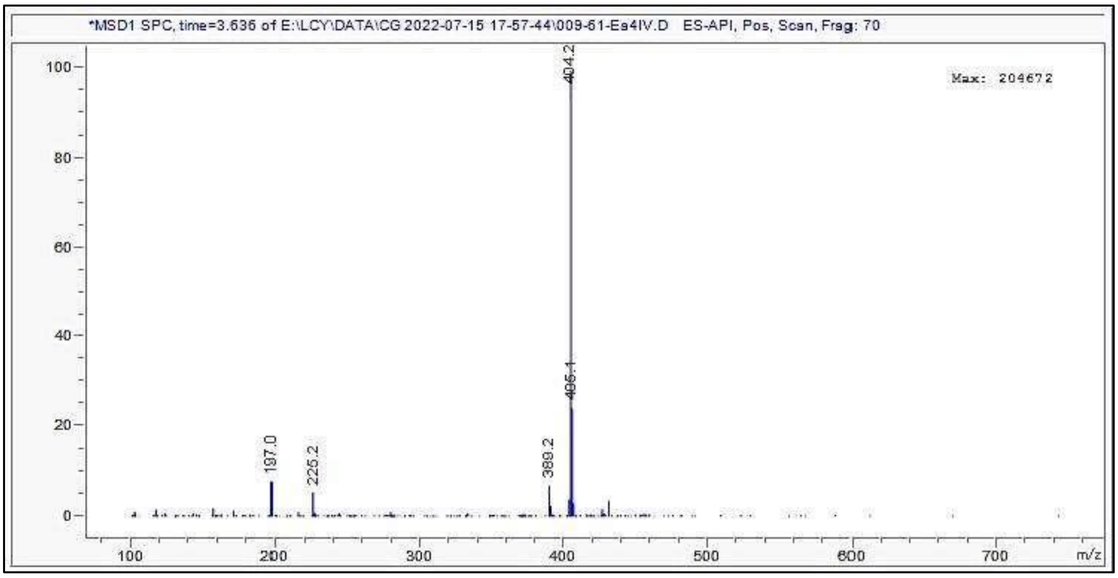

LCMS spectrum of compound 20h

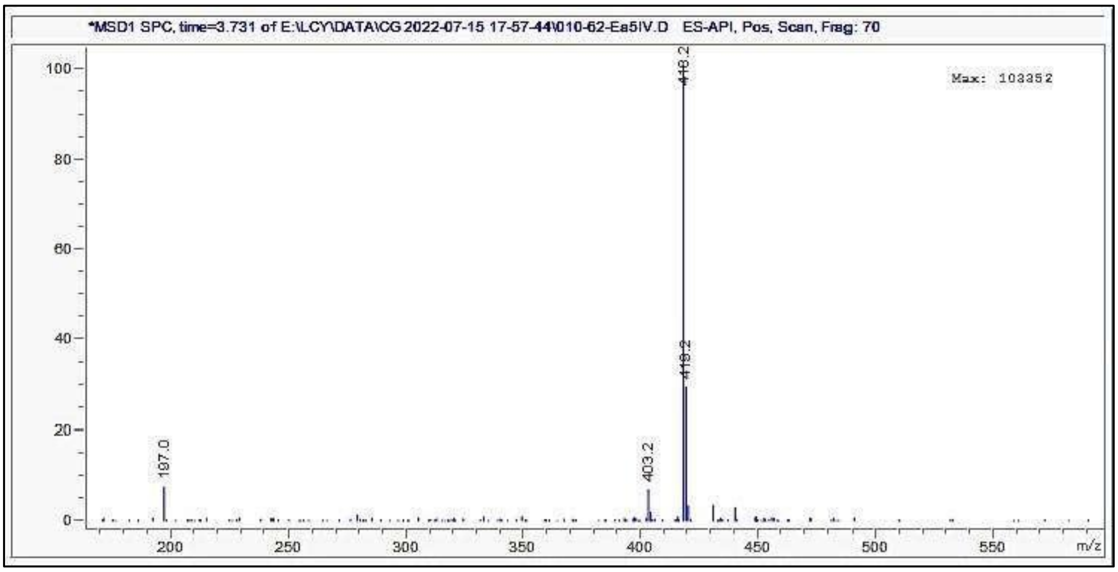

LCMS spectrum of compound 20i

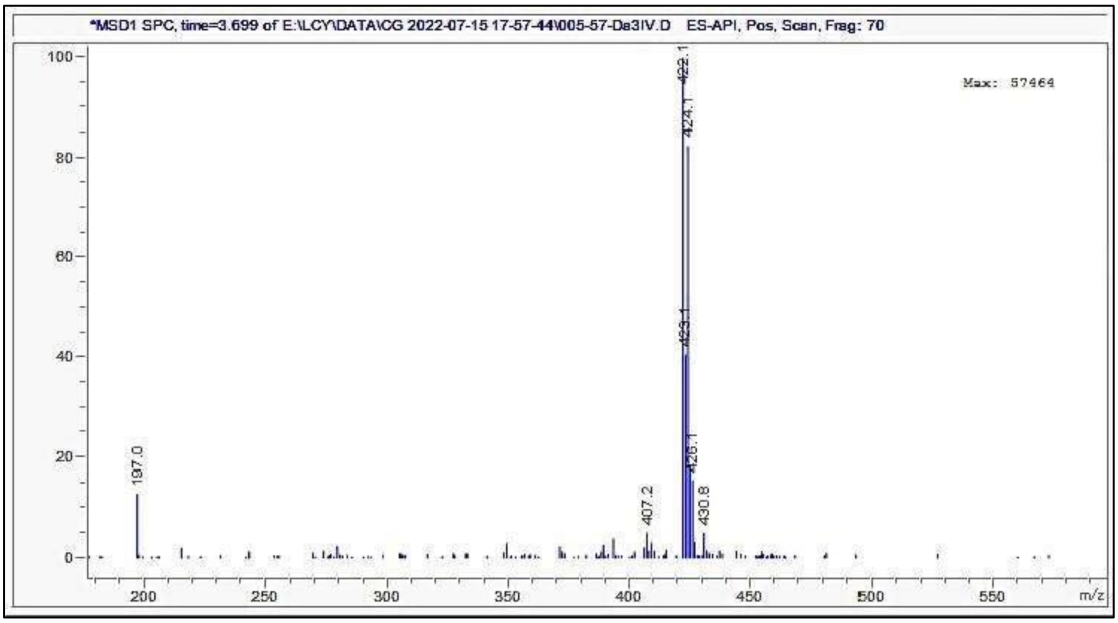

LCMS spectrum of compound 20j

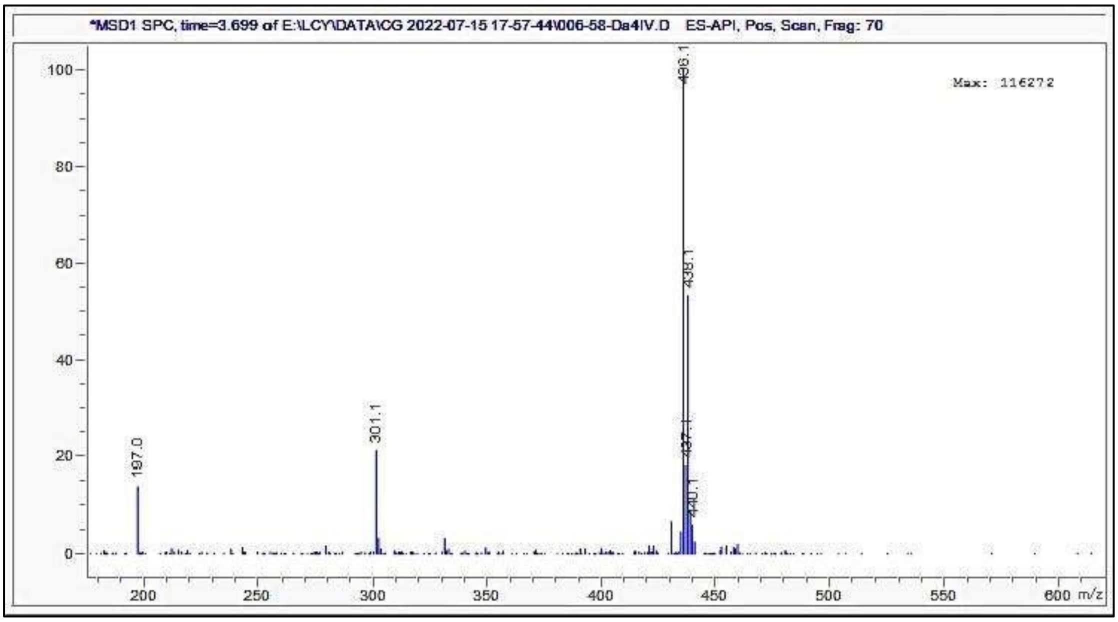

LCMS spectrum of compound 20k

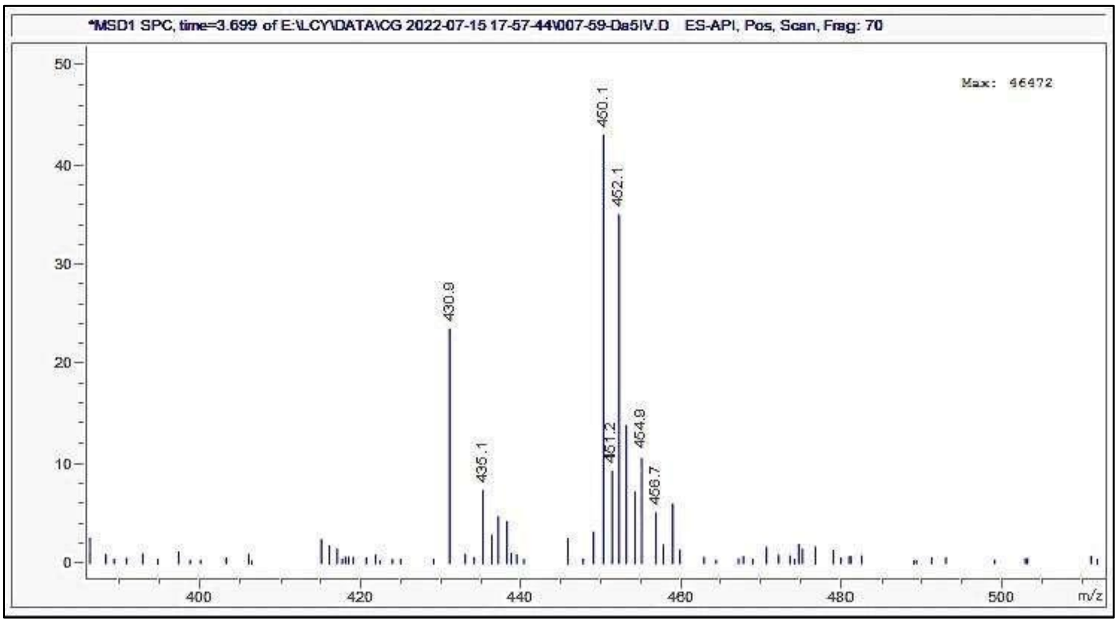

LCMS spectrum of compound 20l

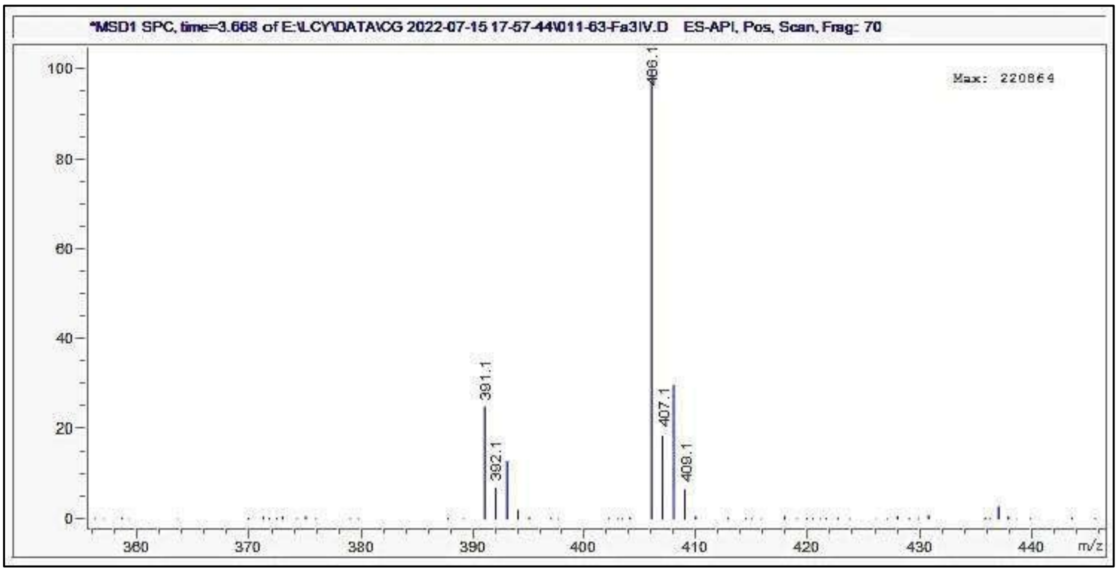

LCMS spectrum of compound 20m

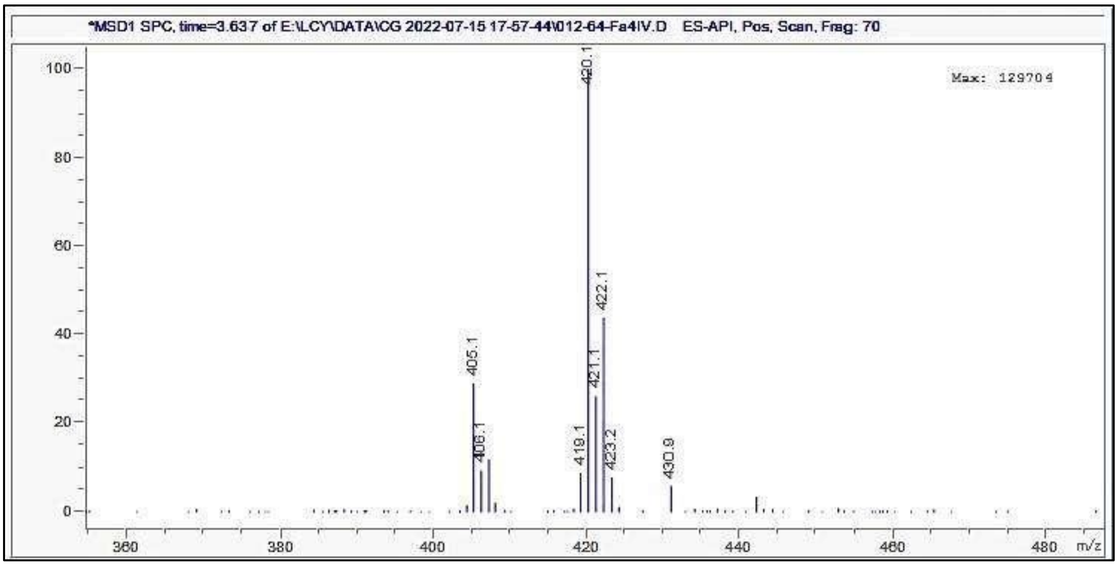

LCMS spectrum of compound 20n

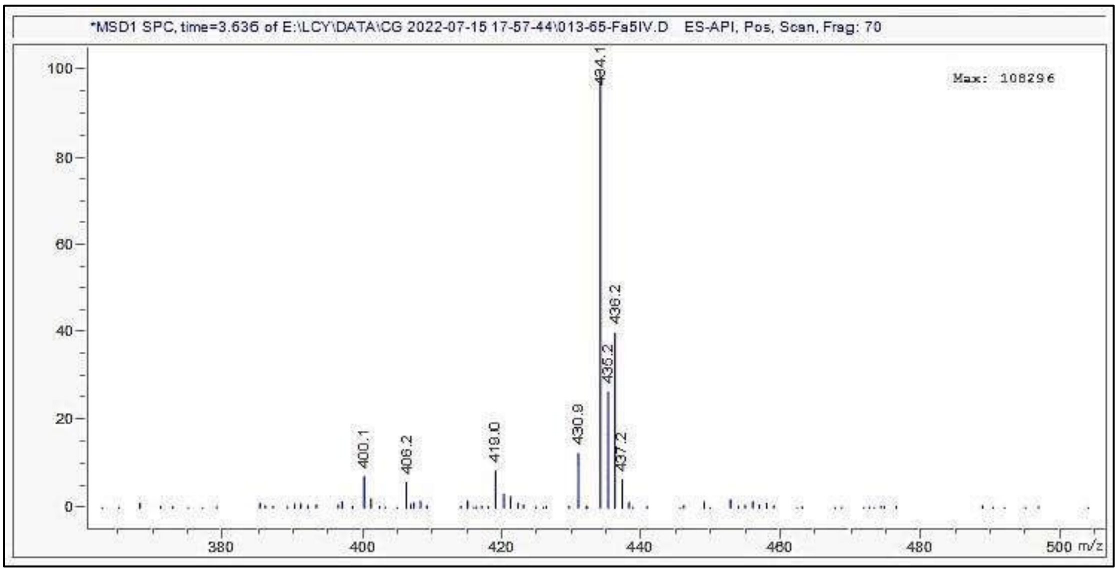

LCMS spectrum of compound 20o

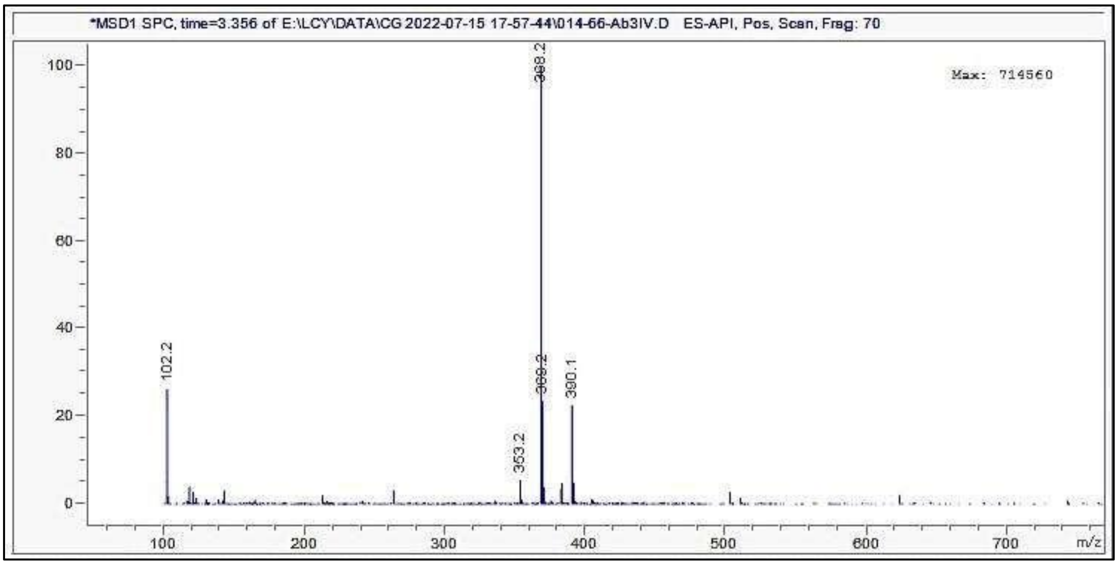

LCMS spectrum of compound 21a

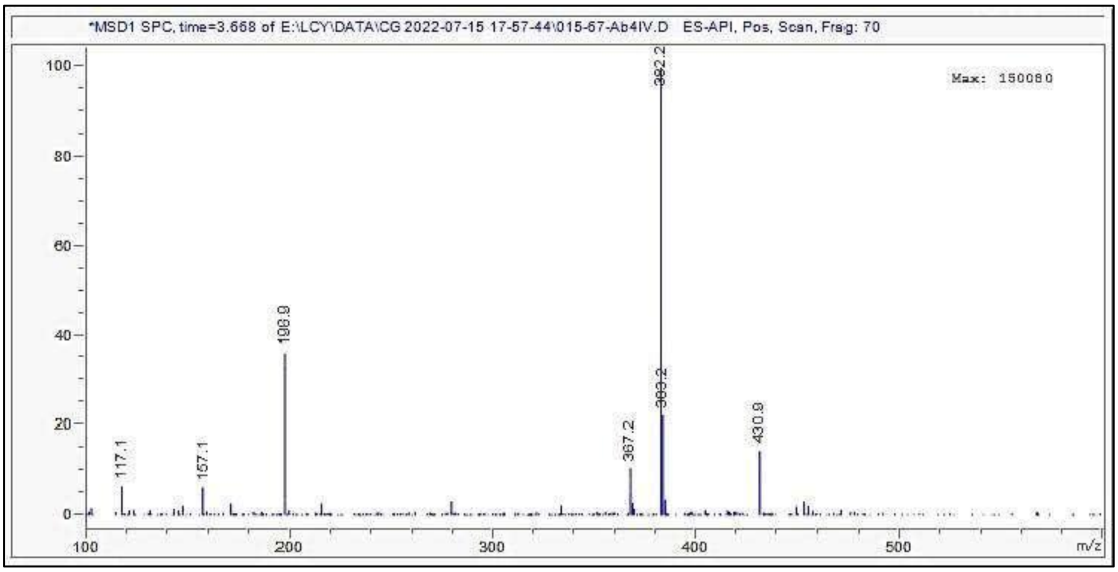

LCMS spectrum of compound 21b

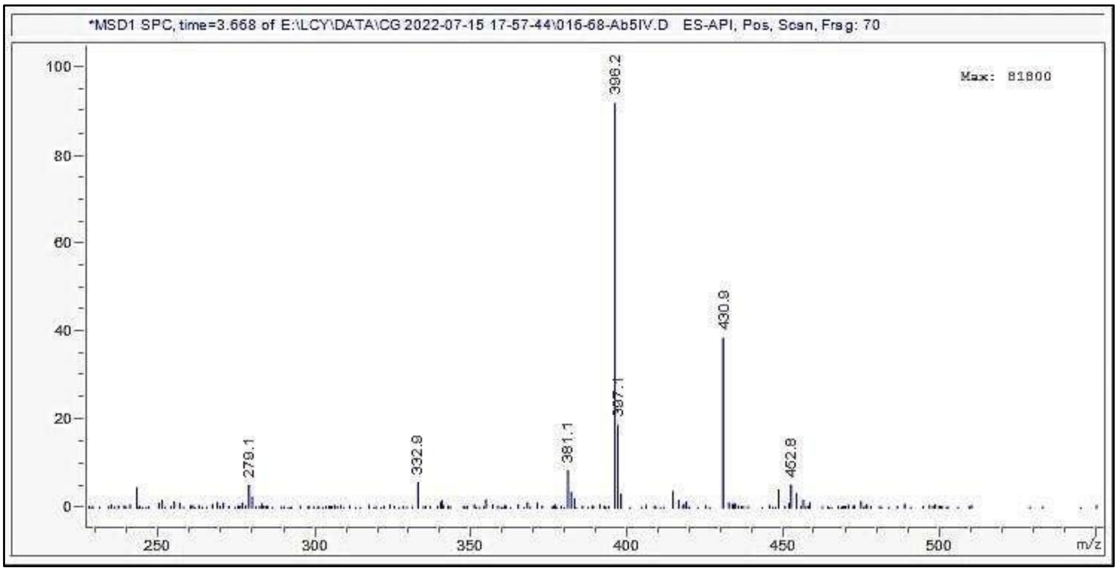

LCMS spectrum of compound 21c

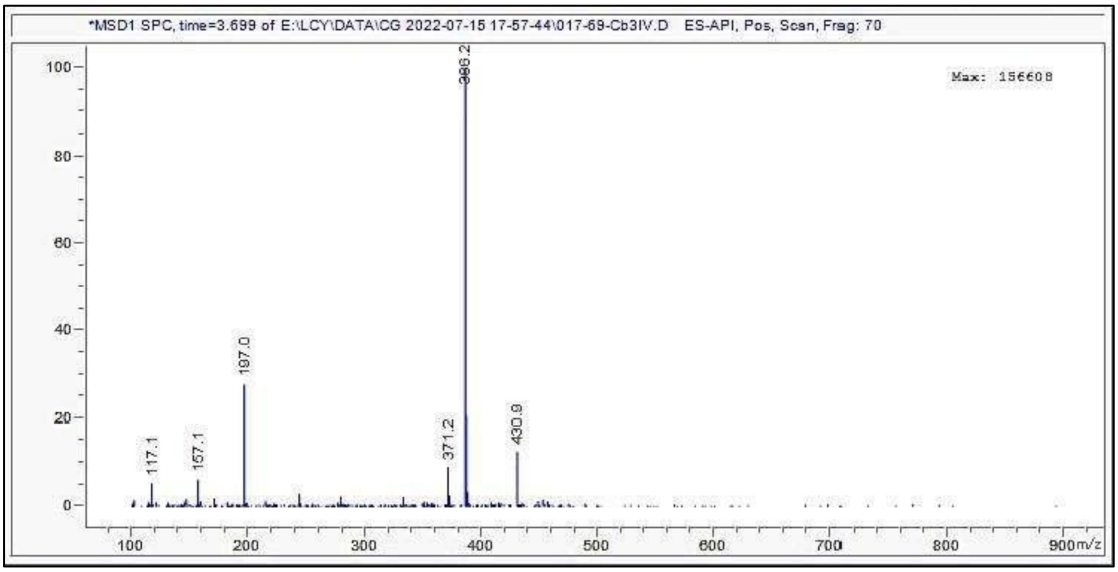

LCMS spectrum of compound 21d

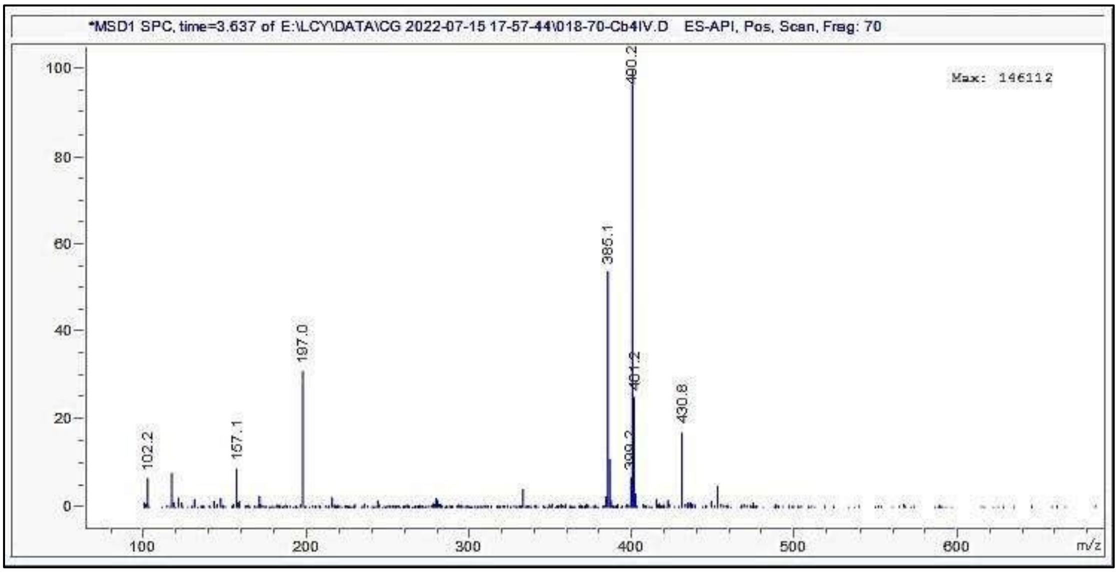

LCMS spectrum of compound 21e

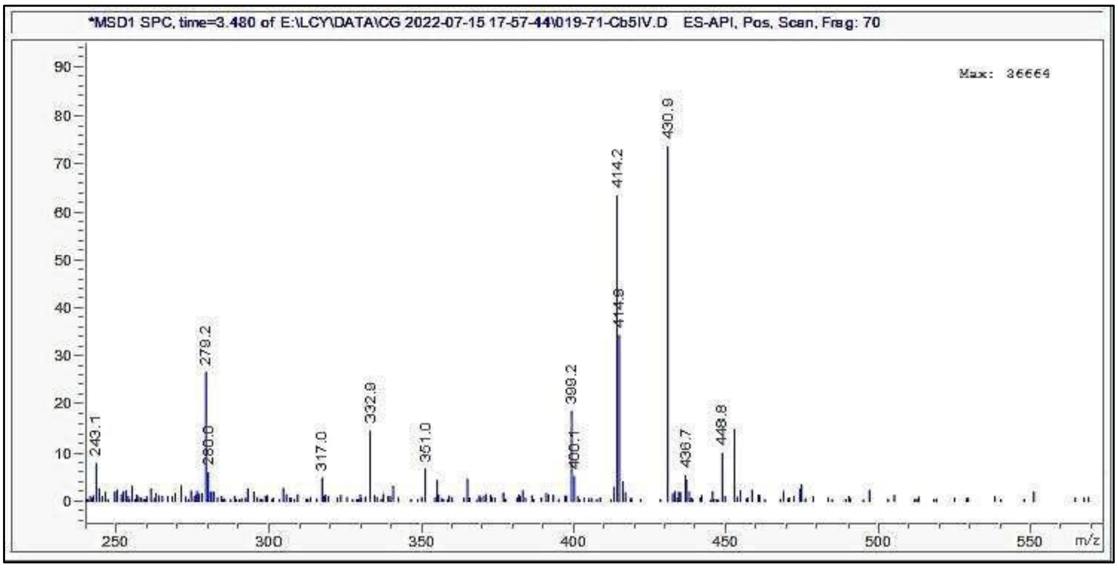

LCMS spectrum of compound 21f

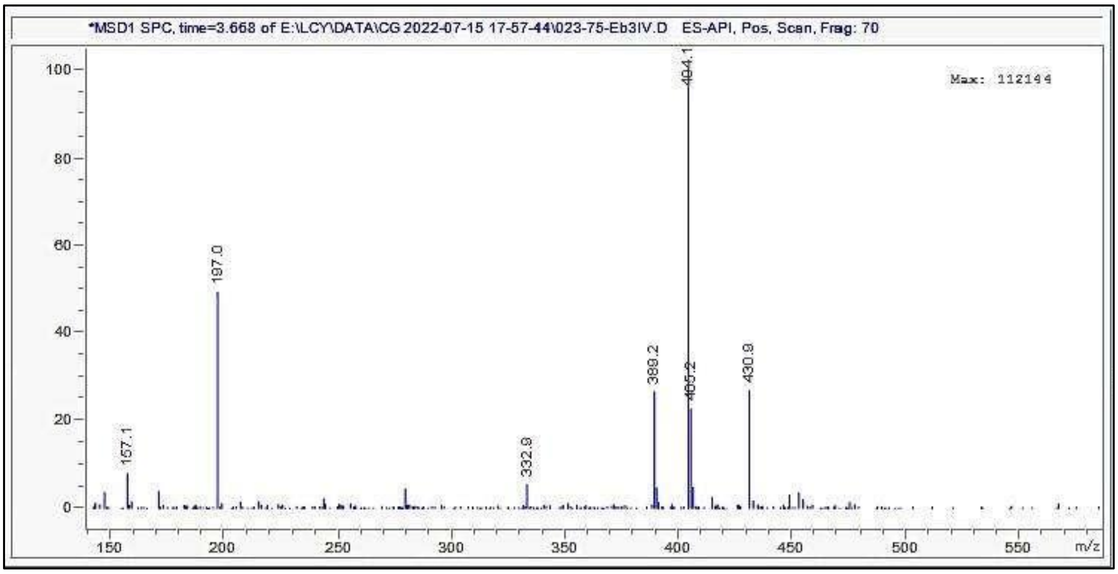

LCMS spectrum of compound 21g

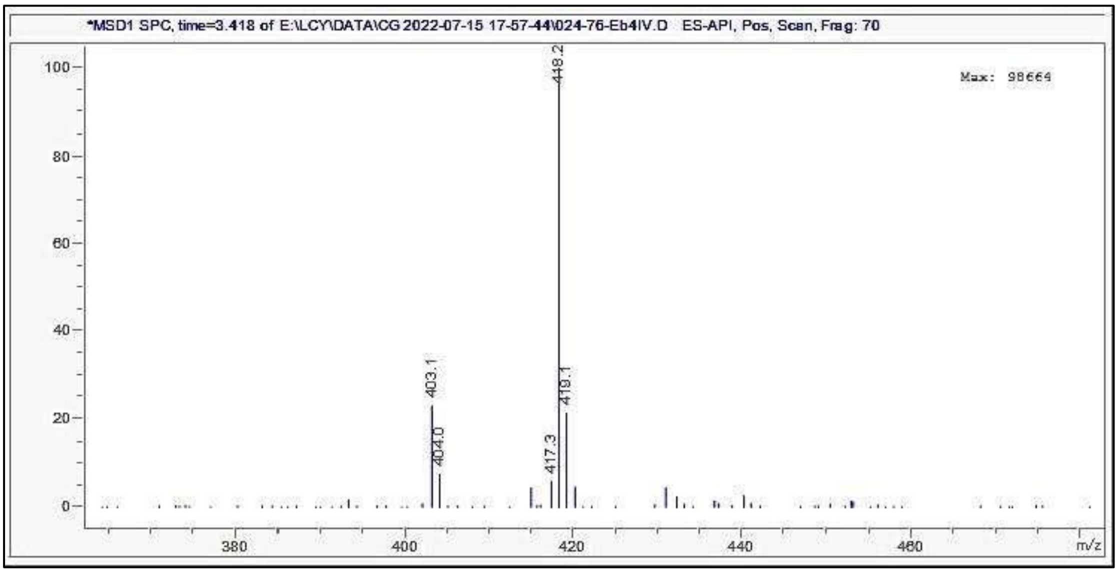

LCMS spectrum of compound 21h

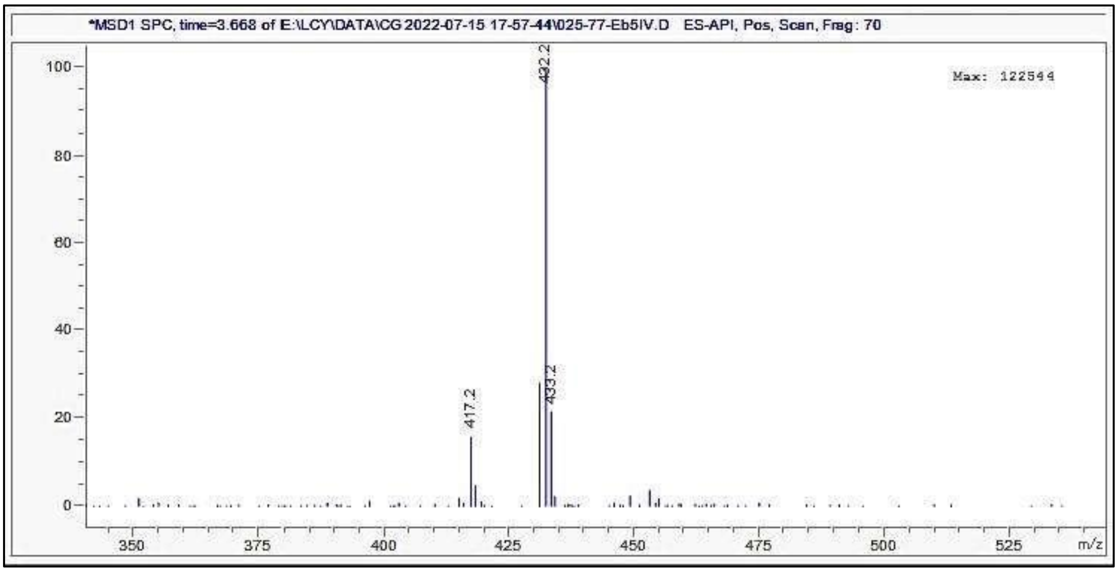

LCMS spectrum of compound 21i

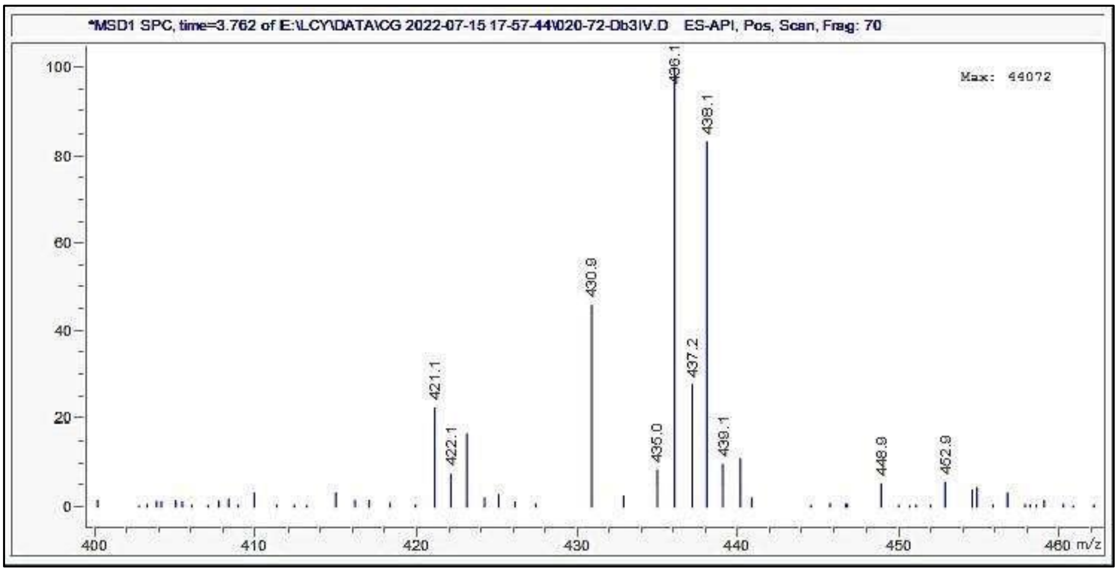

LCMS spectrum of compound 21j

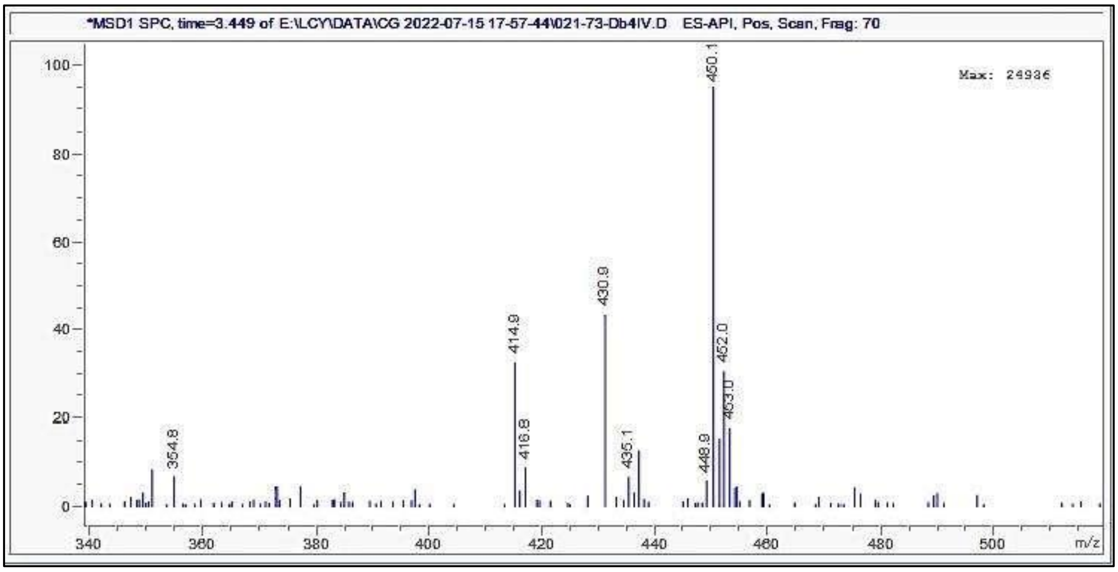

LCMS spectrum of compound 21k

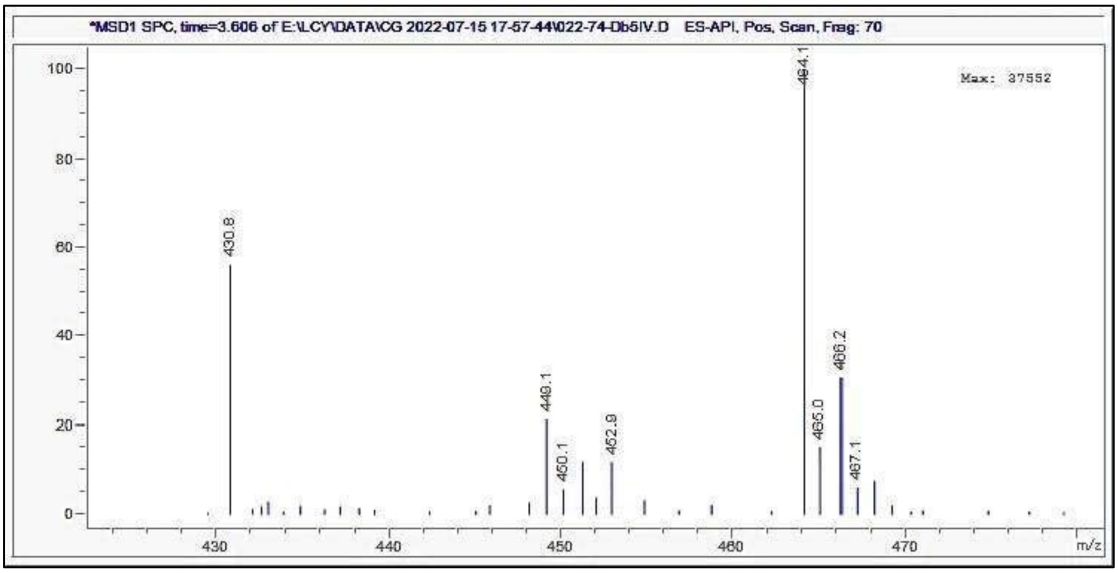

LCMS spectrum of compound 21l

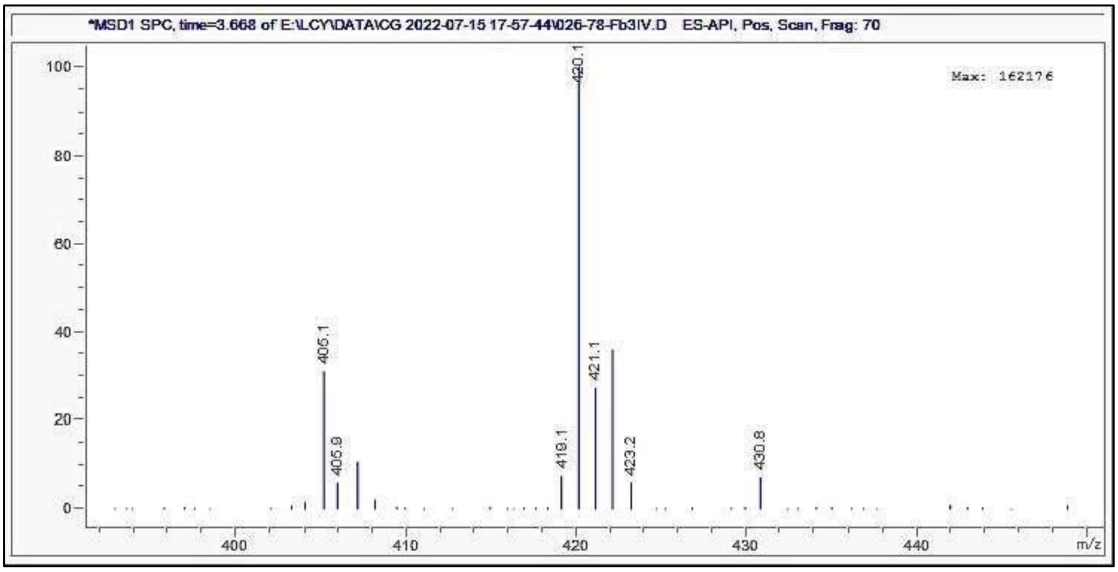

LCMS spectrum of compound 21m

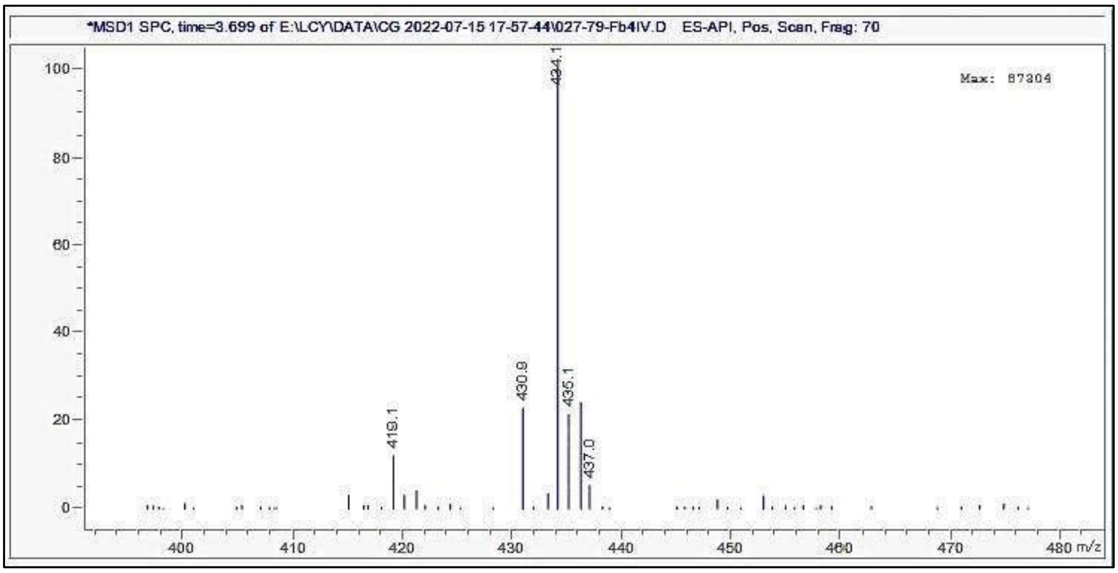

LCMS spectrum of compound 21n

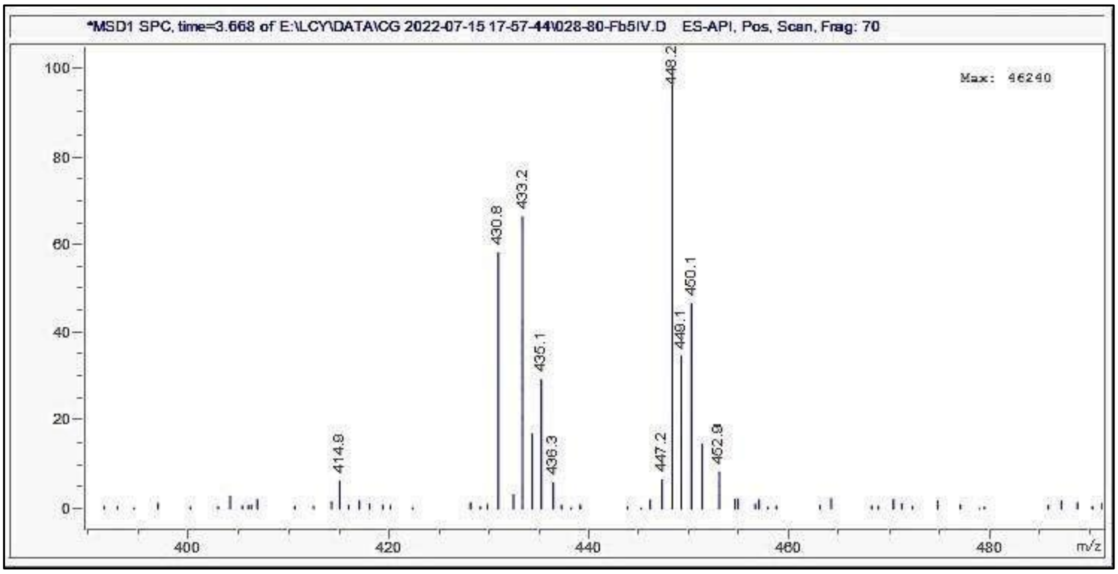

LCMS spectrum of compound 21o

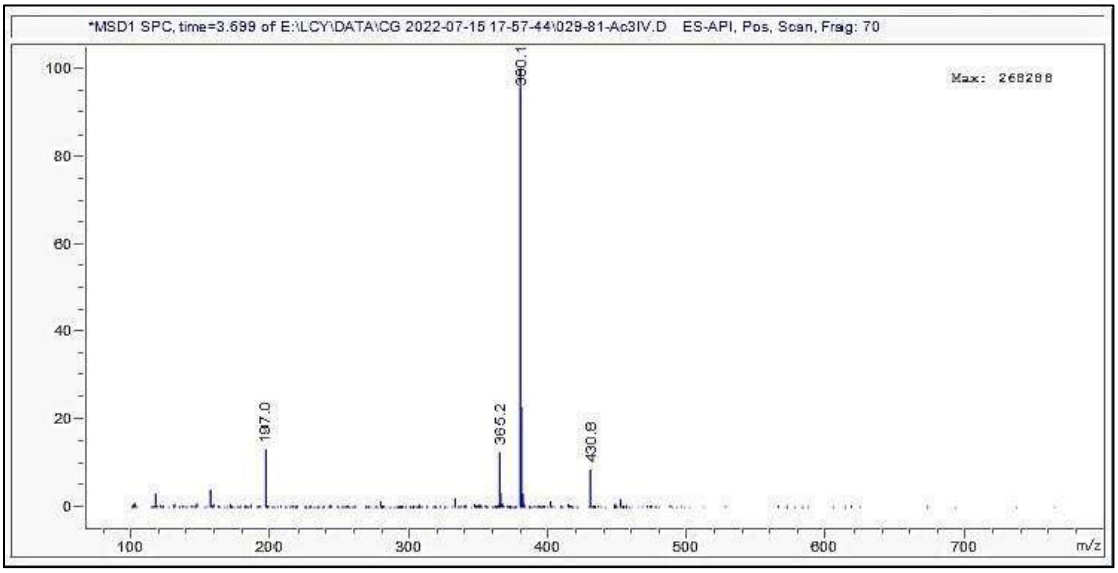

LCMS spectrum of compound 22a

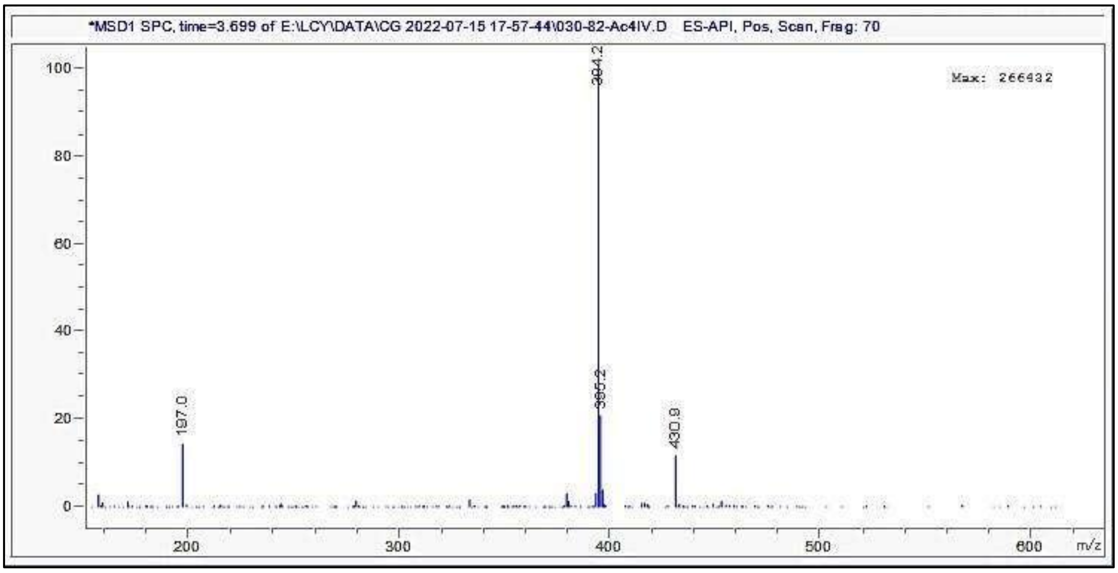

LCMS spectrum of compound 22b

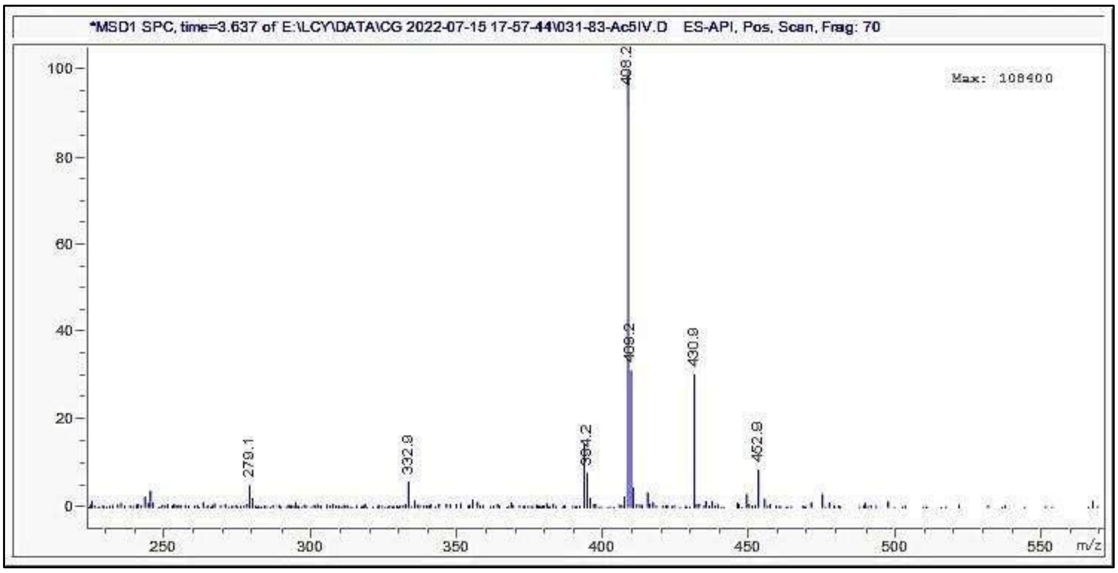

LCMS spectrum of compound 22c

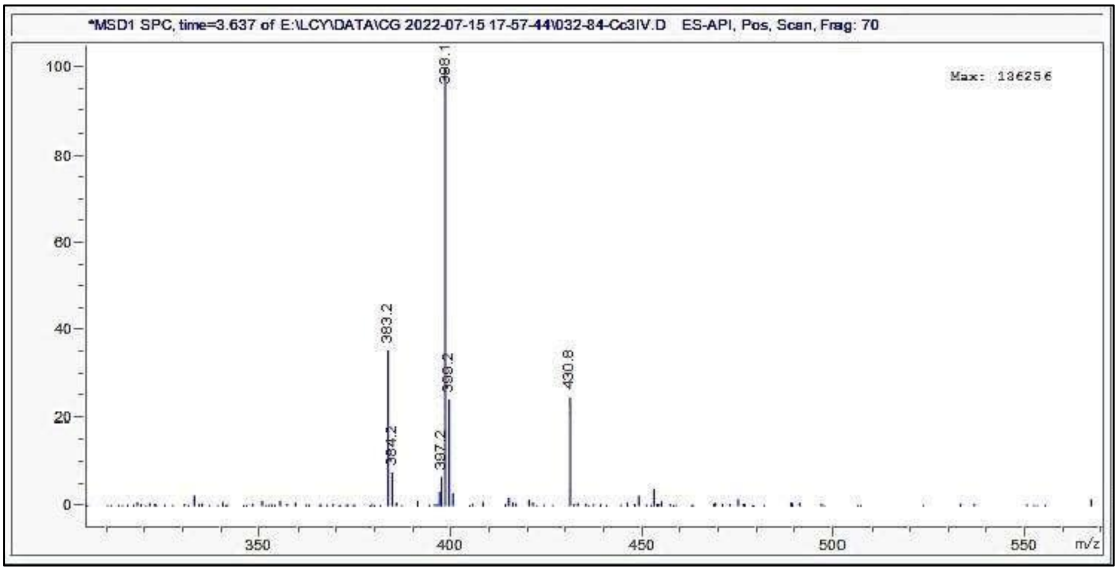

LCMS spectrum of compound 22d

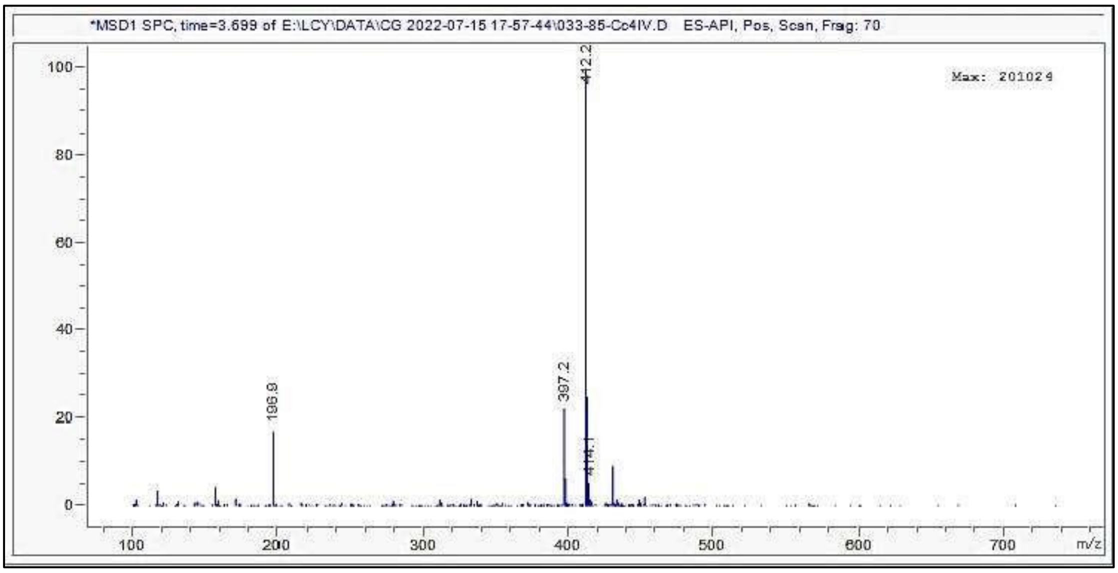

LCMS spectrum of compound 22e

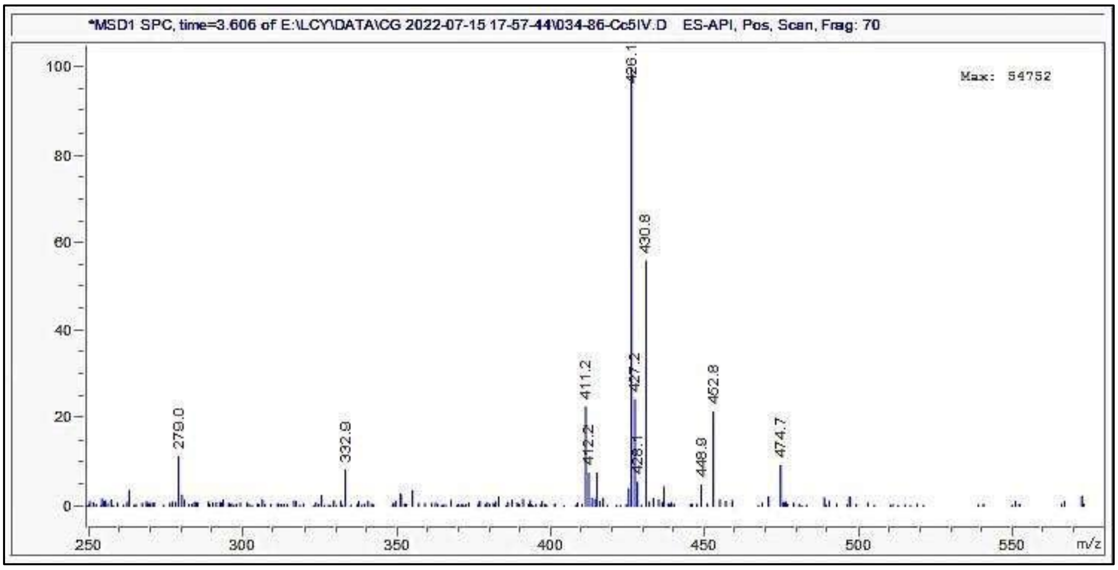

LCMS spectrum of compound 22f

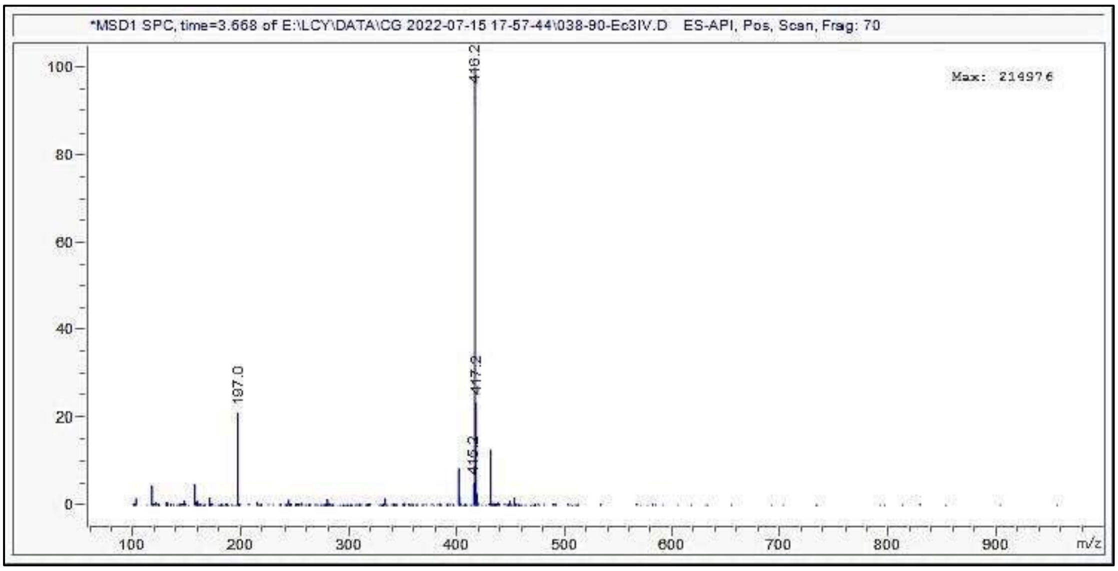

LCMS spectrum of compound 22g

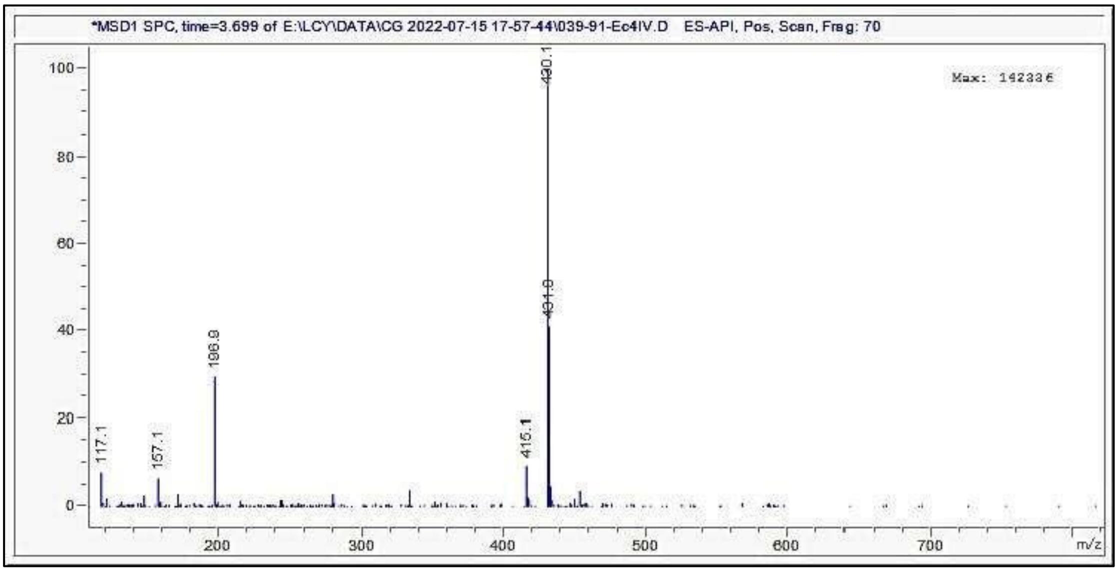

LCMS spectrum of compound 22h

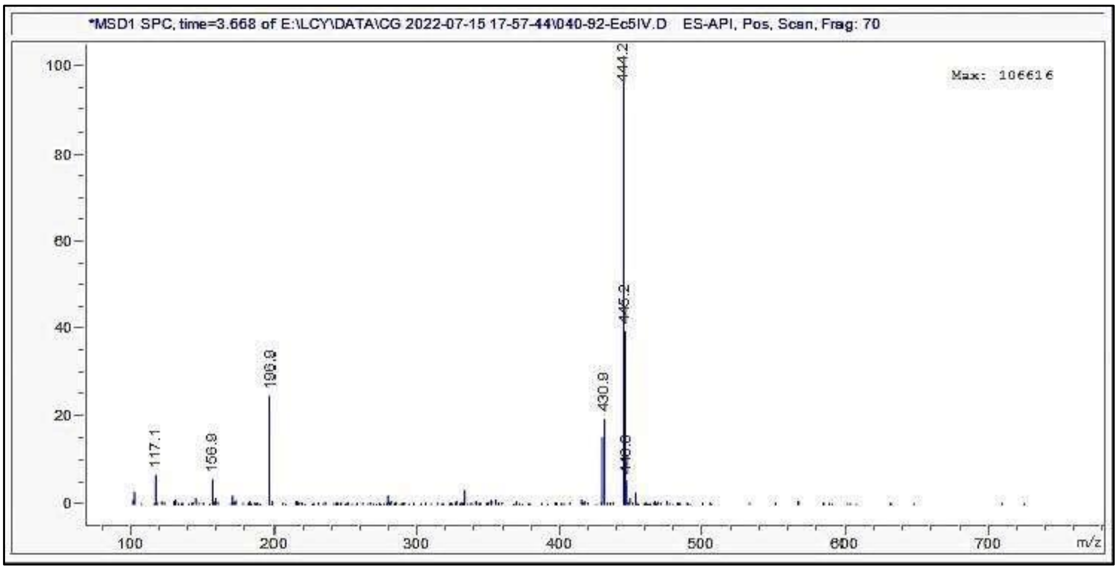

LCMS spectrum of compound 22i

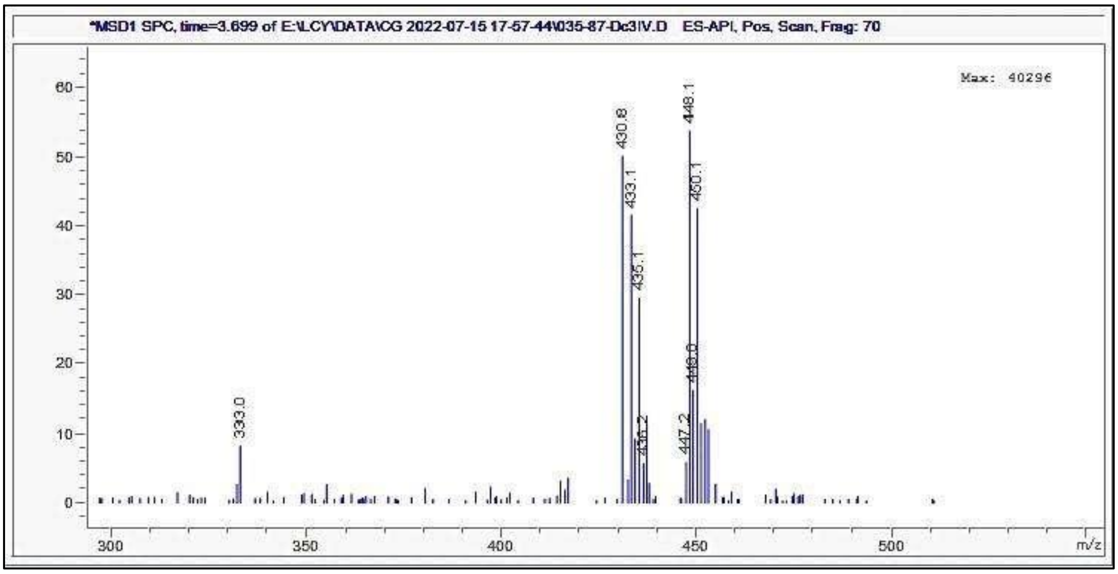

LCMS spectrum of compound 22j

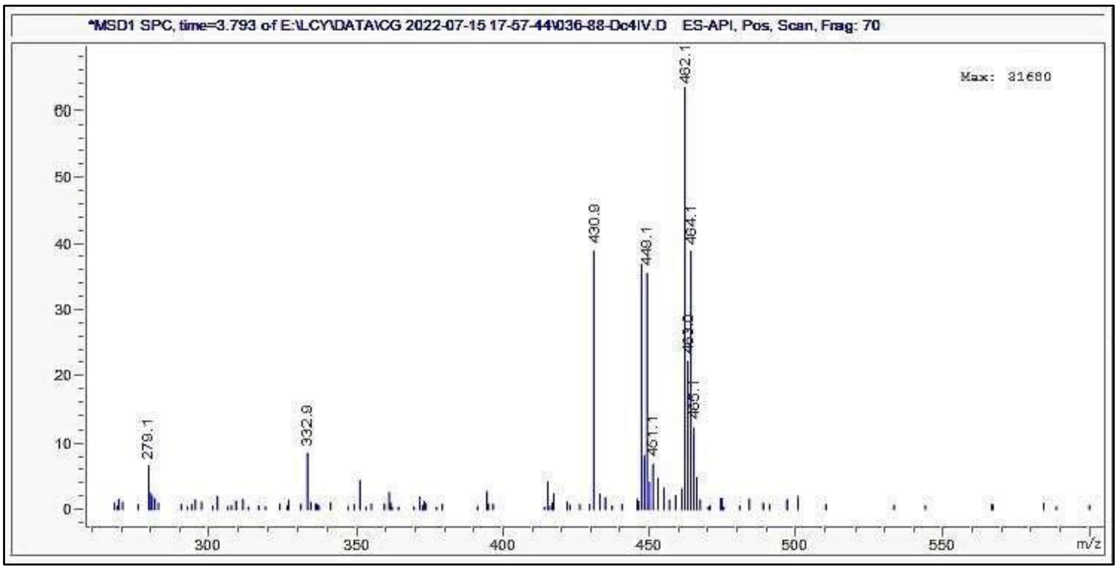

LCMS spectrum of compound 22k

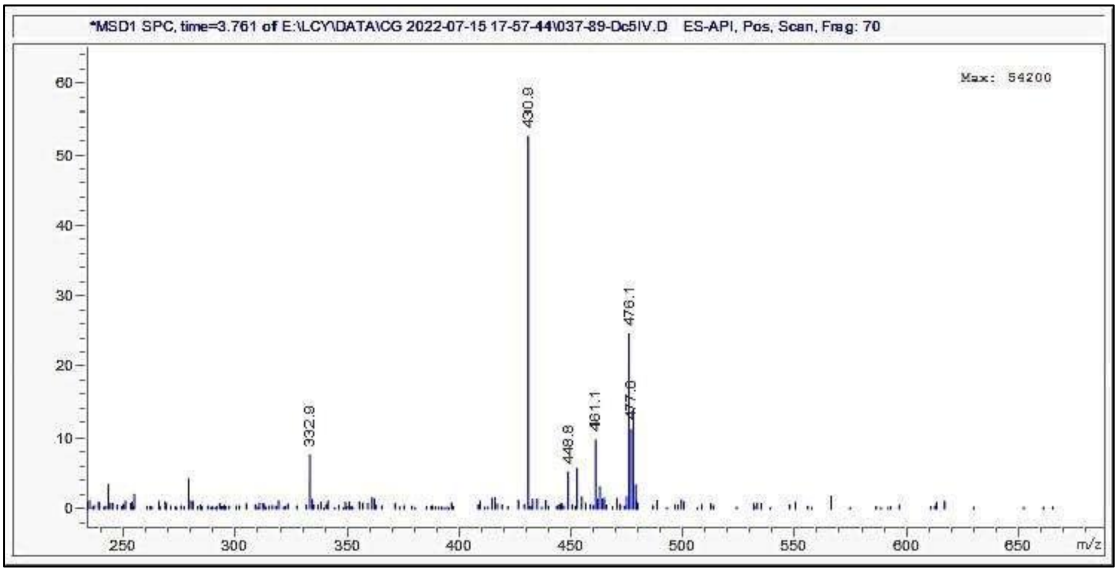

LCMS spectrum of compound 22l

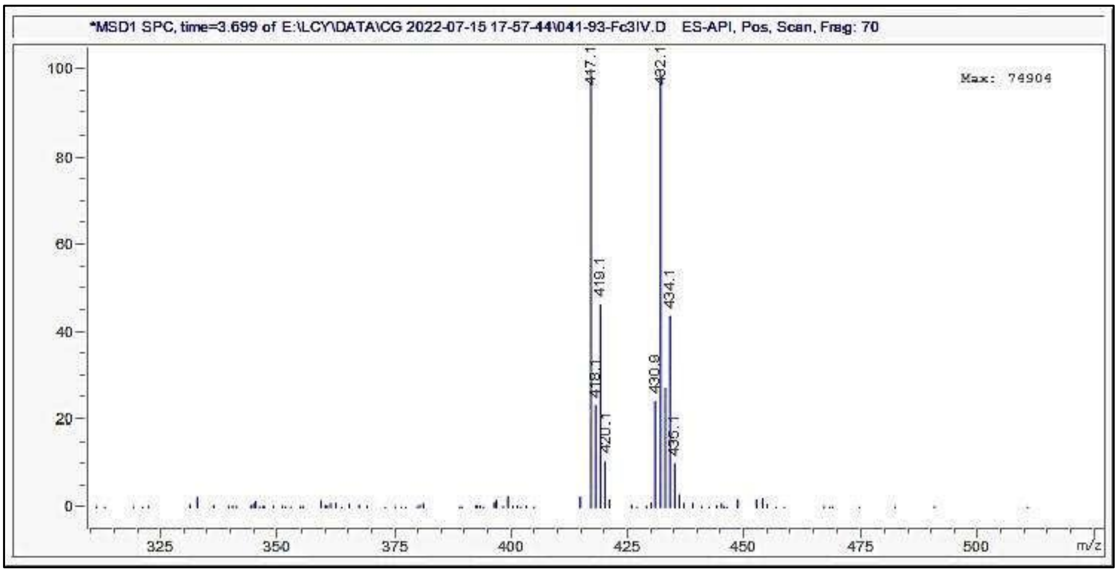

LCMS spectrum of compound 22m

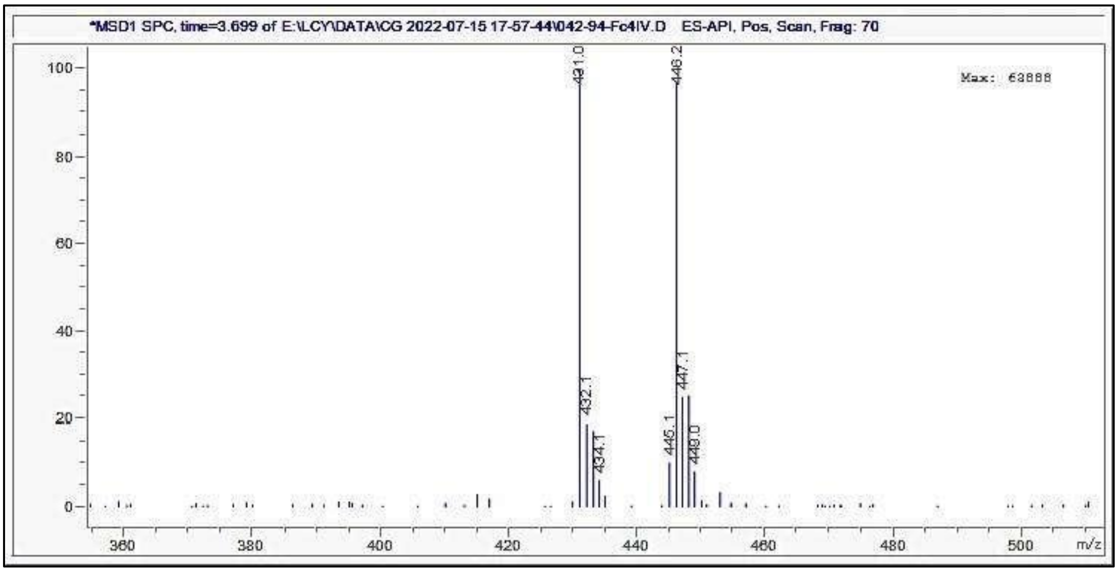

LCMS spectrum of compound 22n

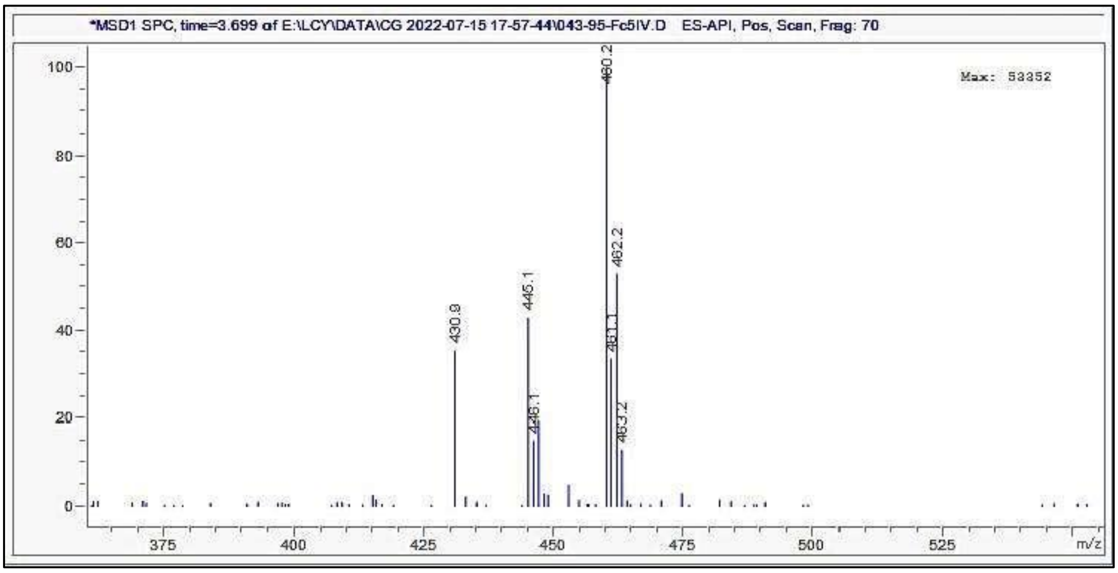

LCMS spectrum of compound 22o

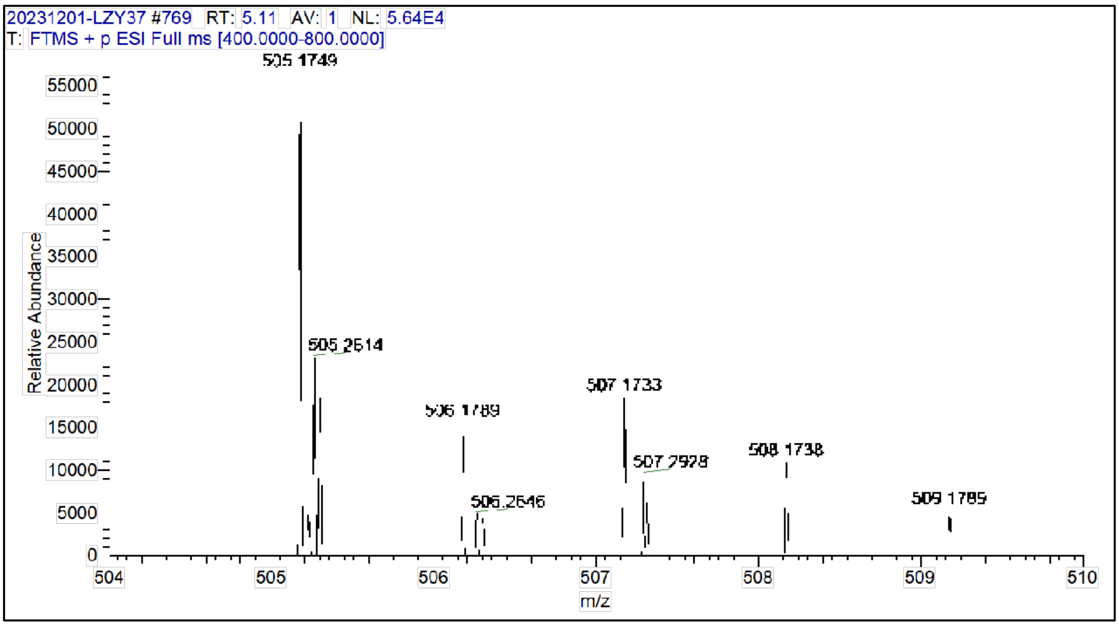

HR-ESI-MS spectrum of compound 32a

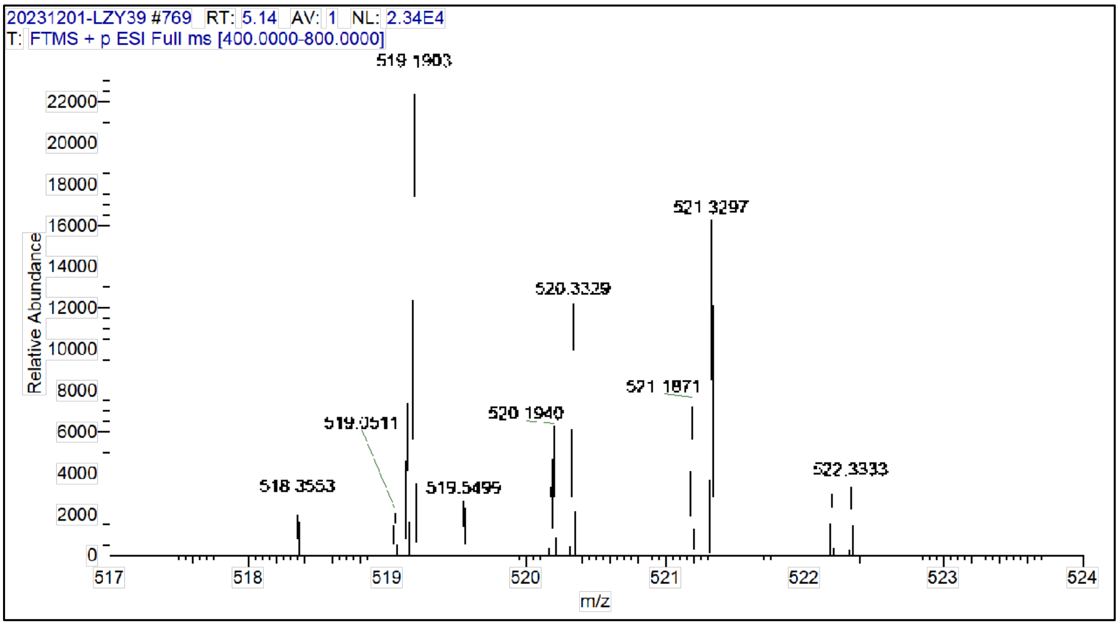

HR-ESI-MS spectrum of compound 32b

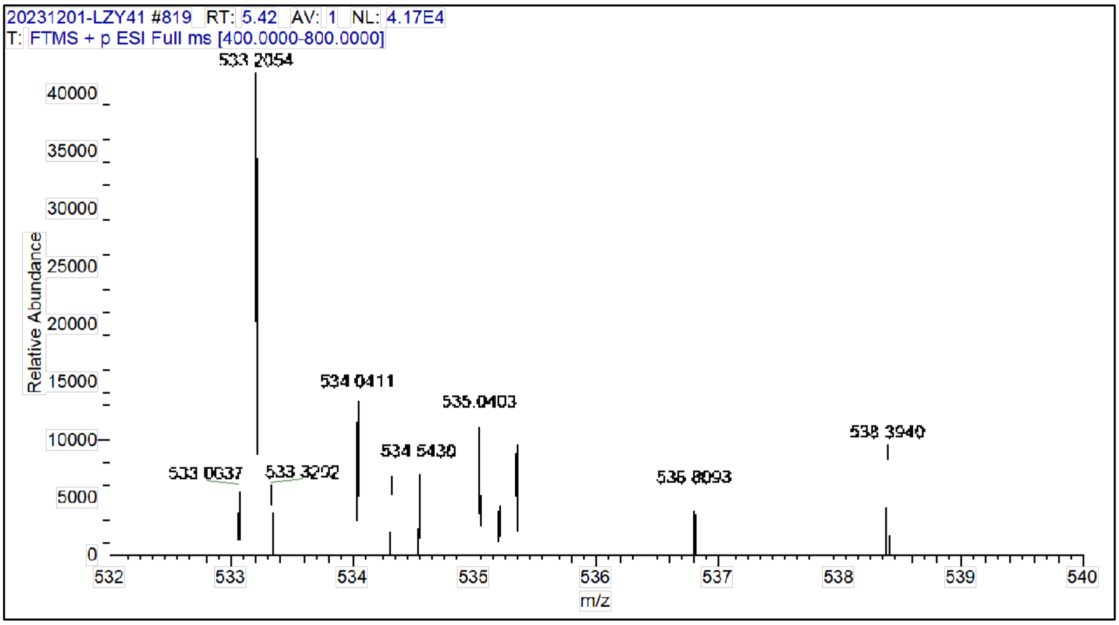

HR-ESI-MS spectrum of compound 32c

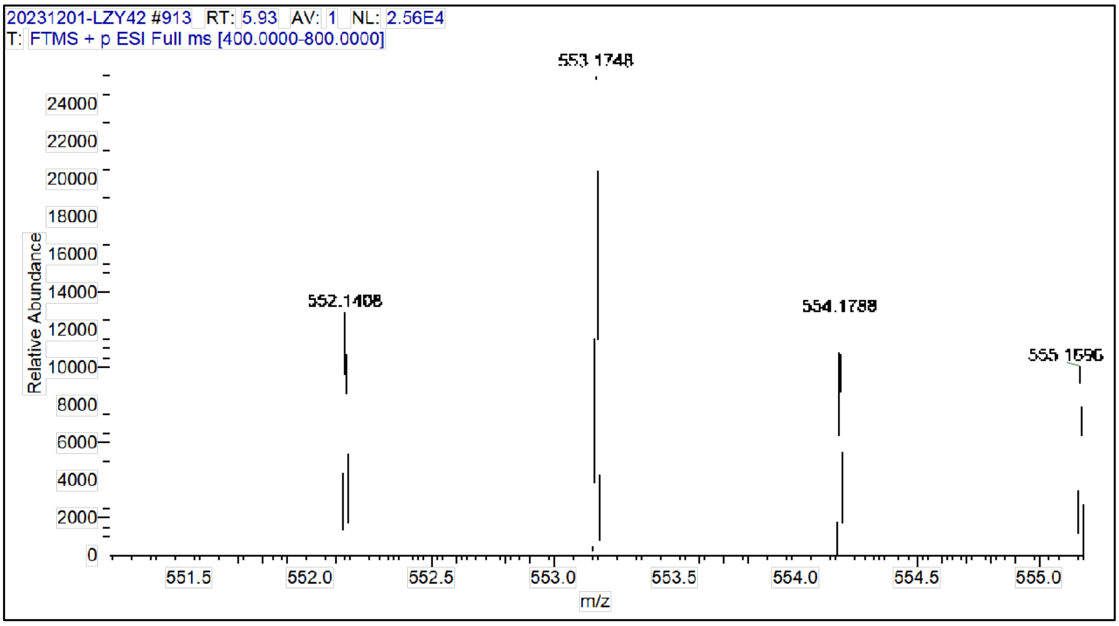

HR-ESI-MS spectrum of compound 32d

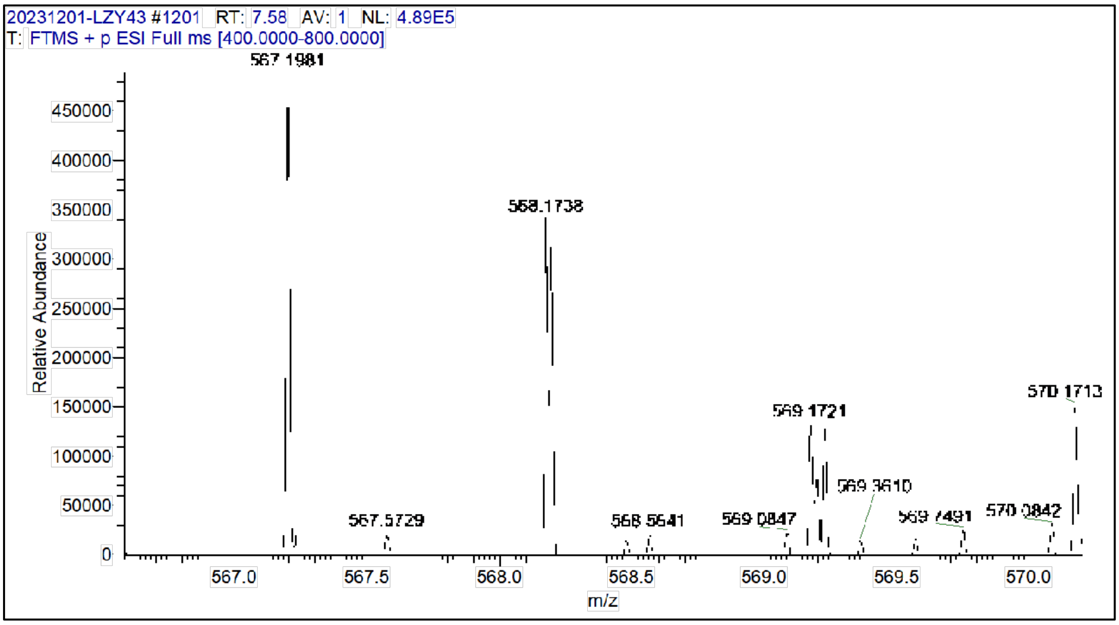

HR-ESI-MS spectrum of compound 32e

The HPLC spectrum of compounds

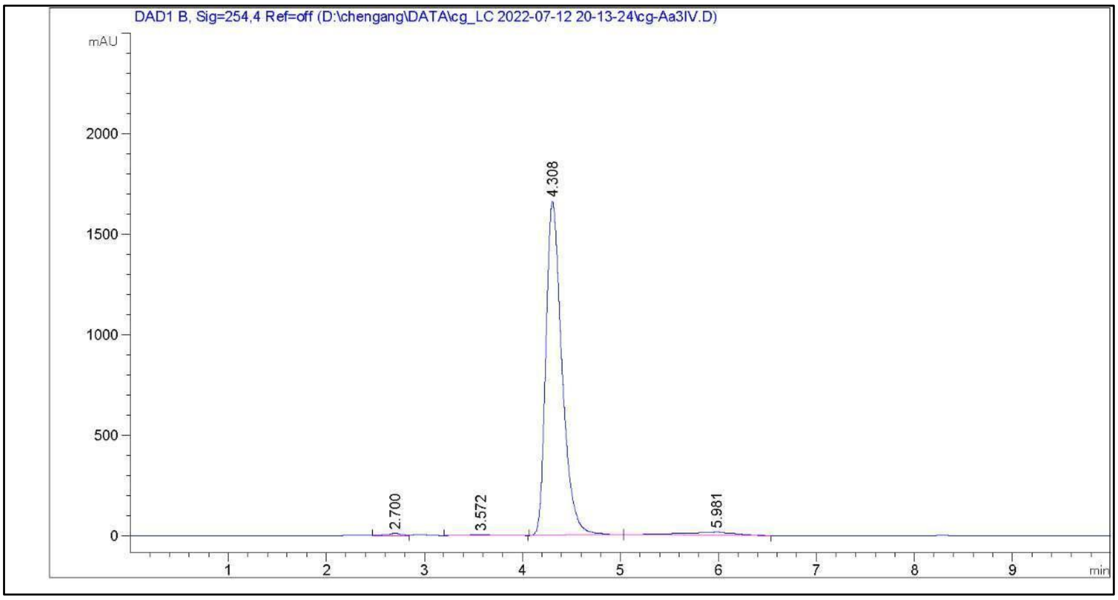

HPLC spectrum of compound 20a

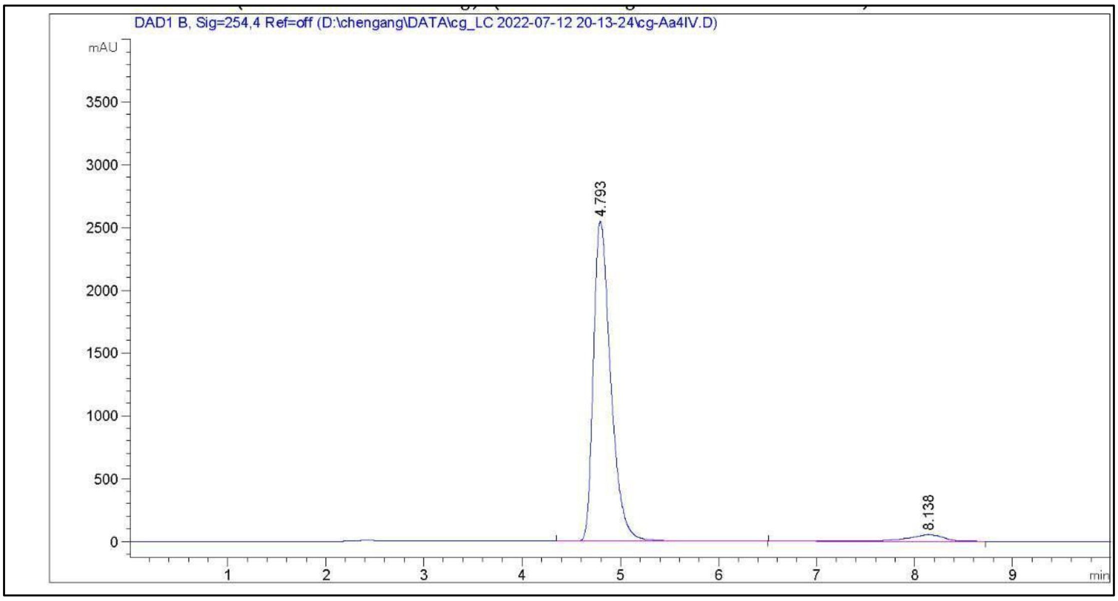

HPLC spectrum of compound 20b

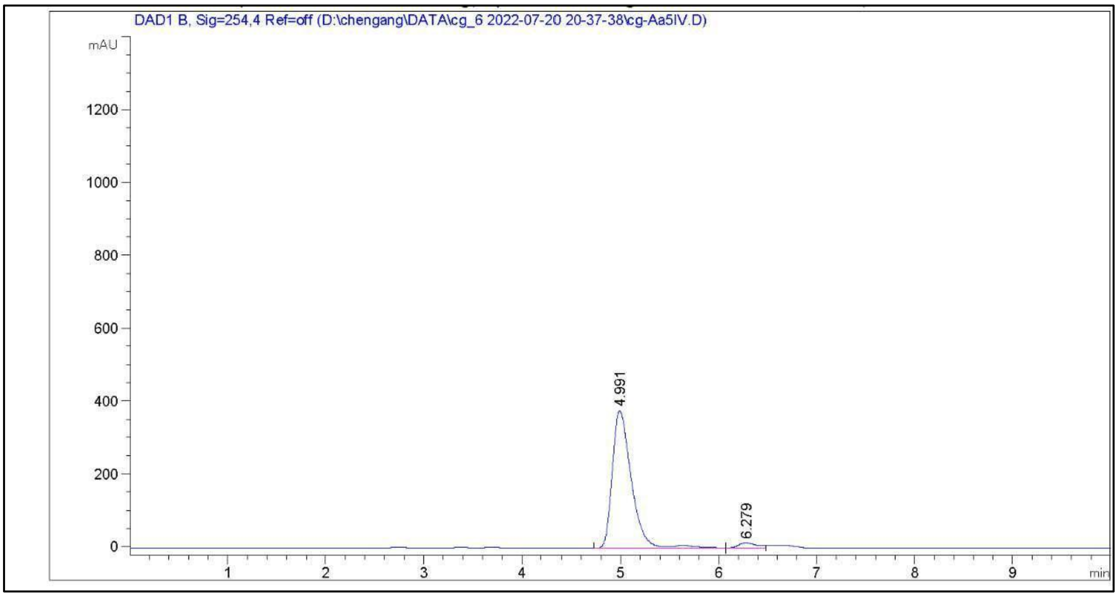

HPLC spectrum of compound 20c

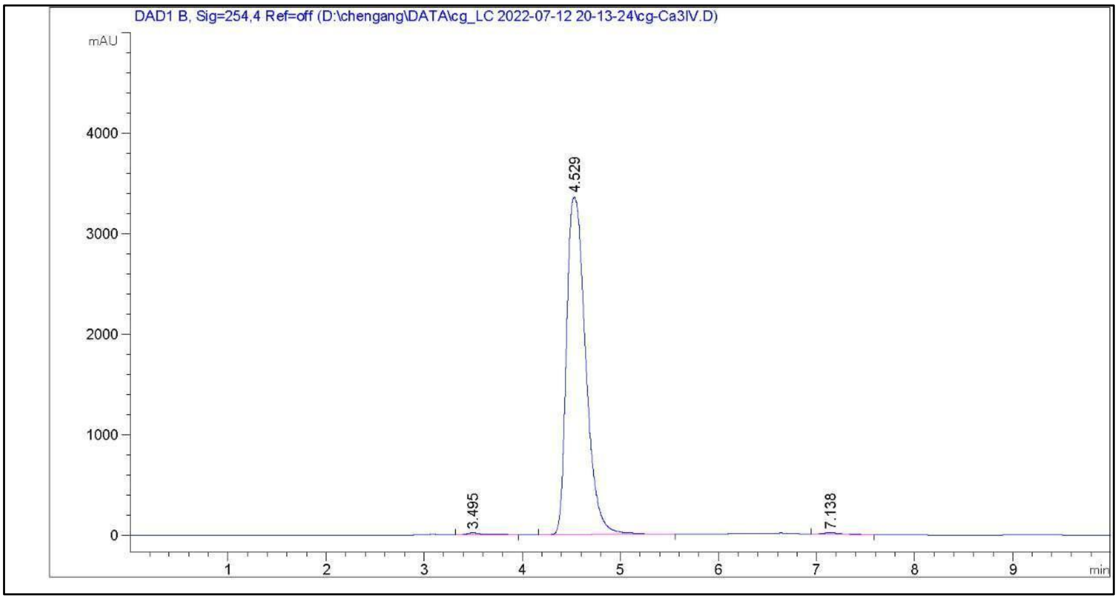

HPLC spectrum of compound 20d

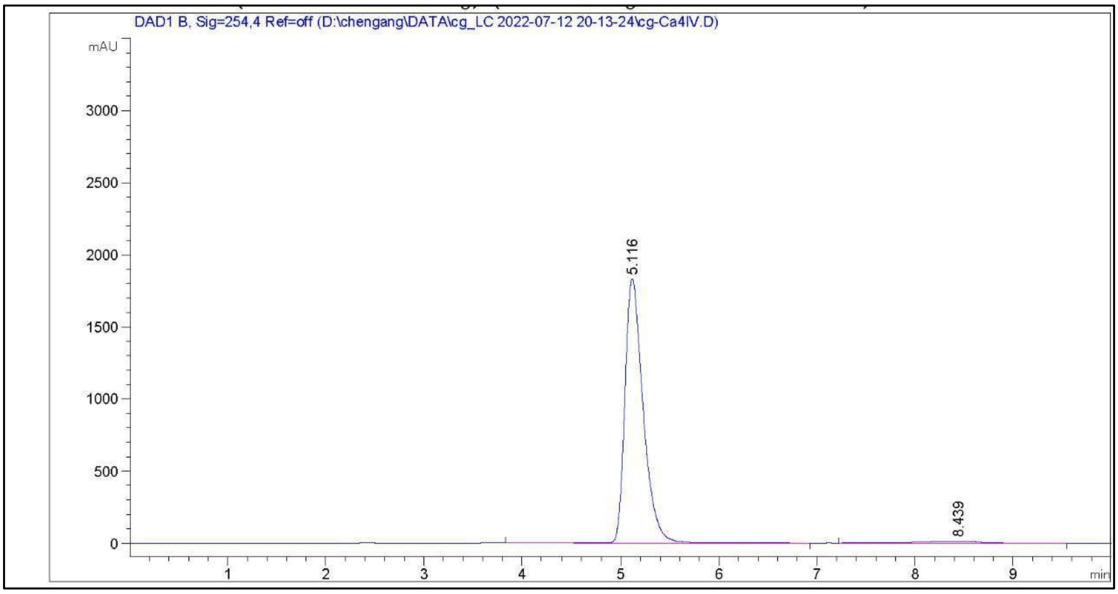

HPLC spectrum of compound 20e

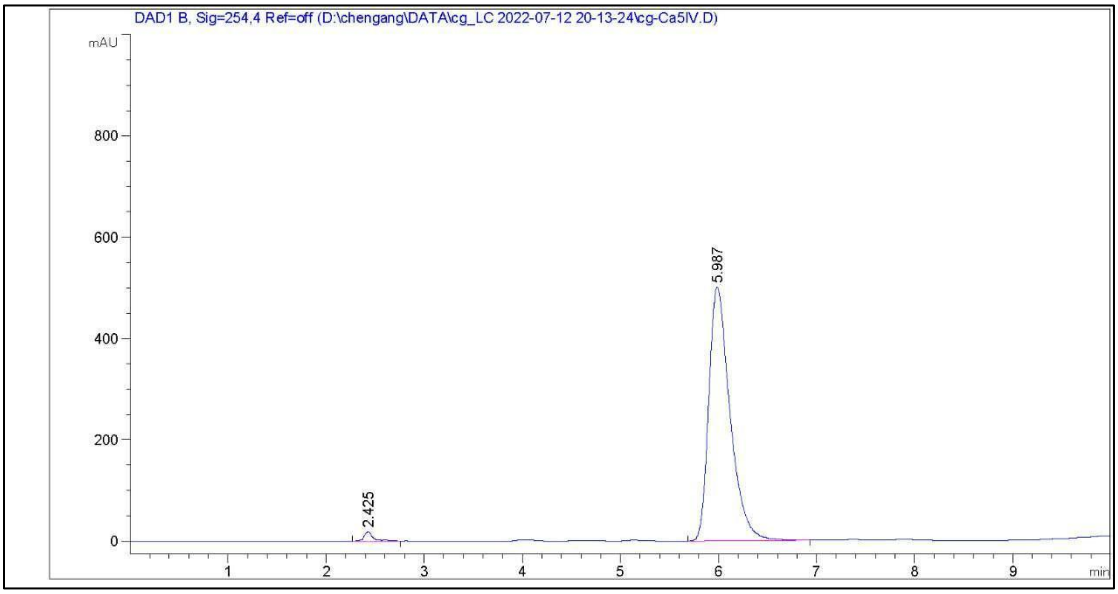

HPLC spectrum of compound 20f

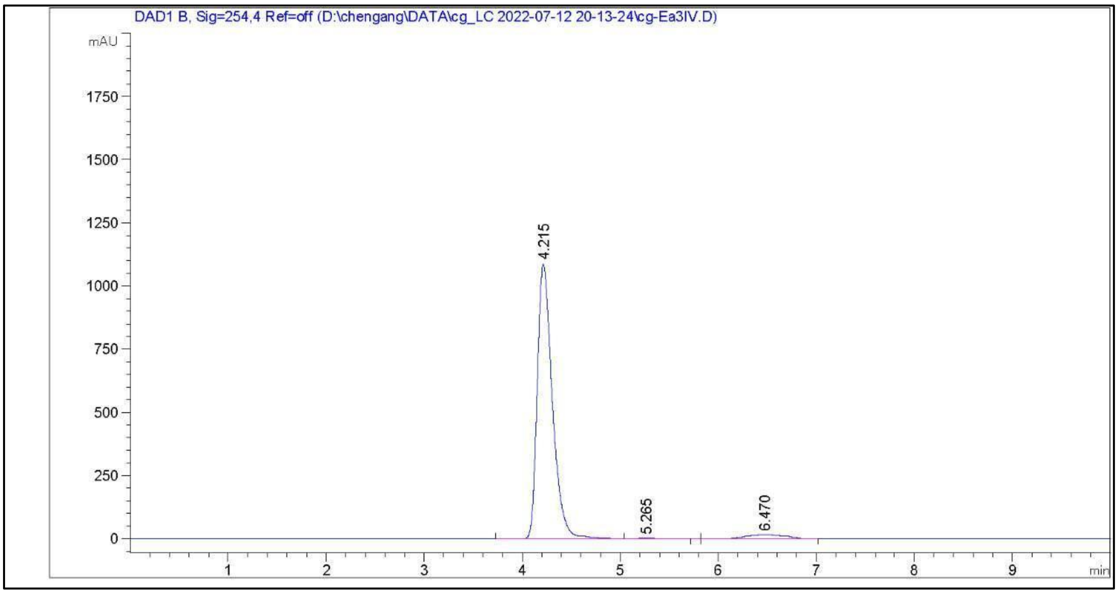

HPLC spectrum of compound 20g

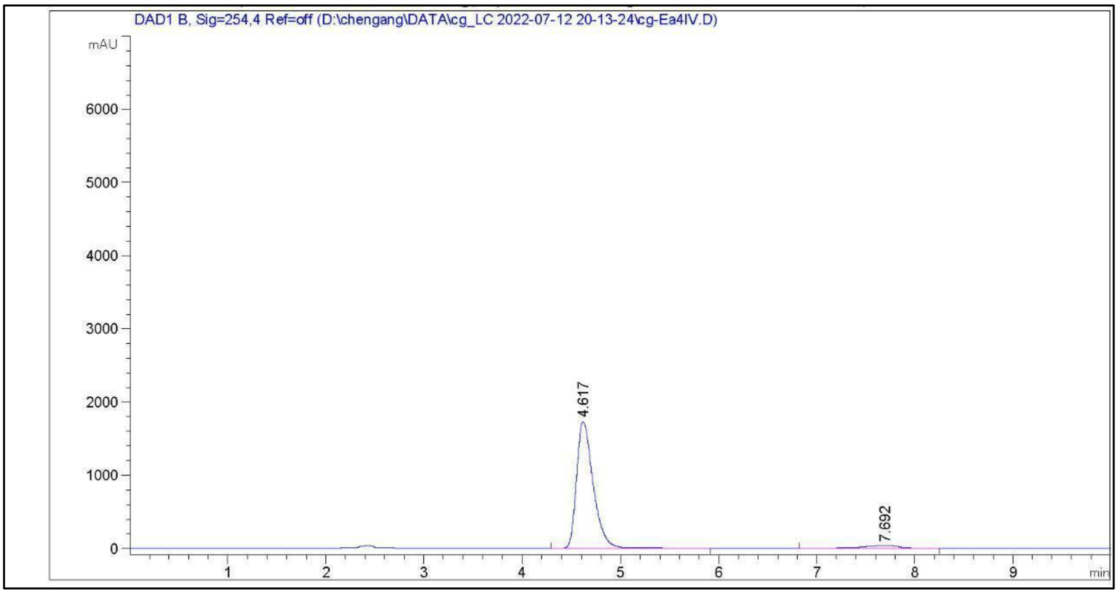

HPLC spectrum of compound 20h

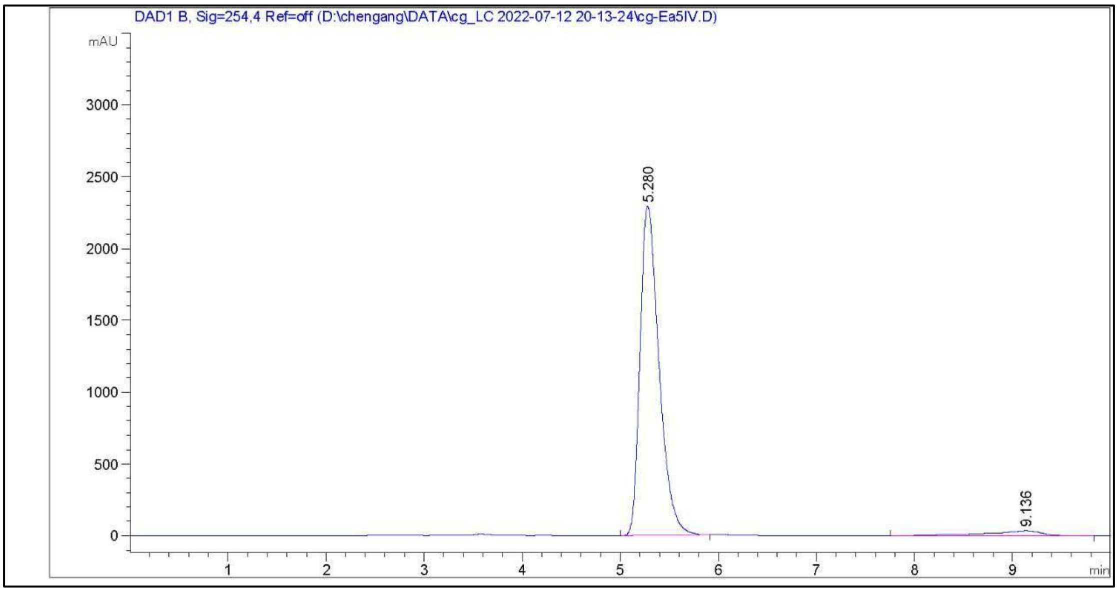

HPLC spectrum of compound 20i

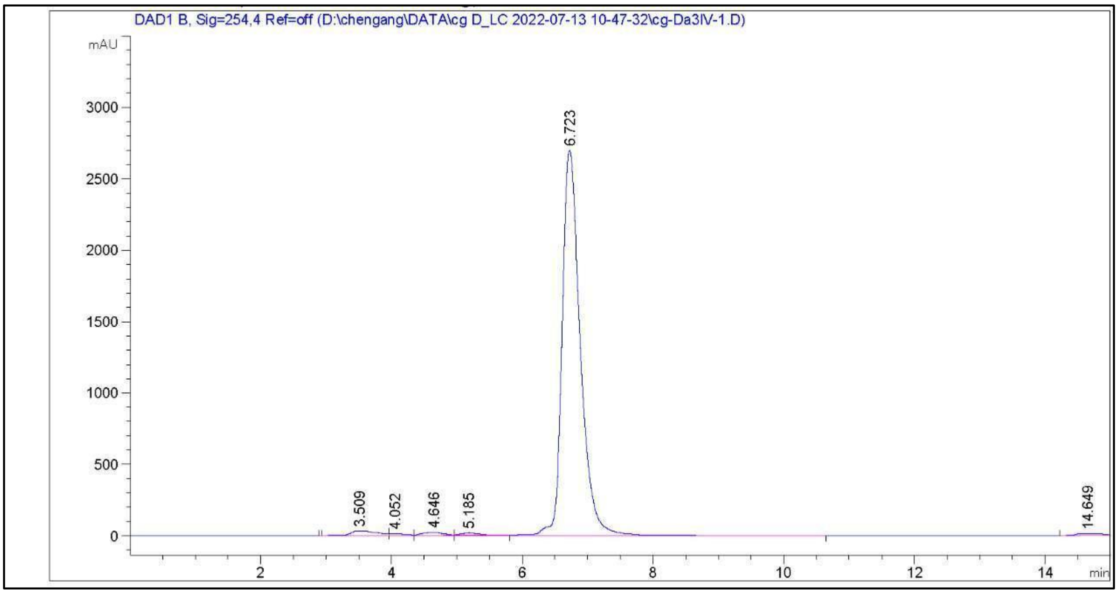

HPLC spectrum of compound 20j

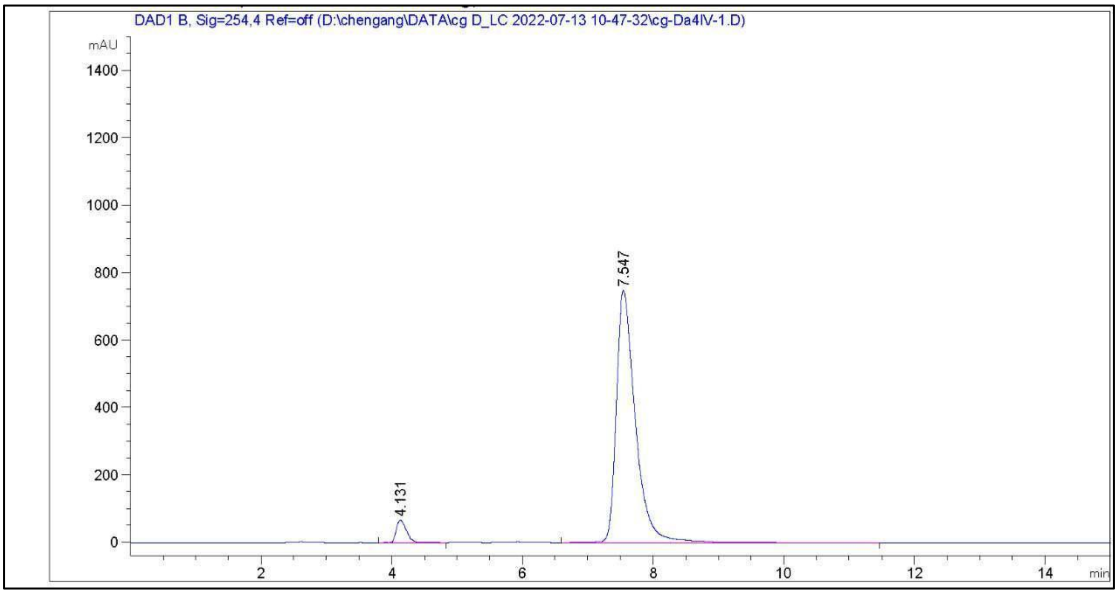

HPLC spectrum of compound 20k

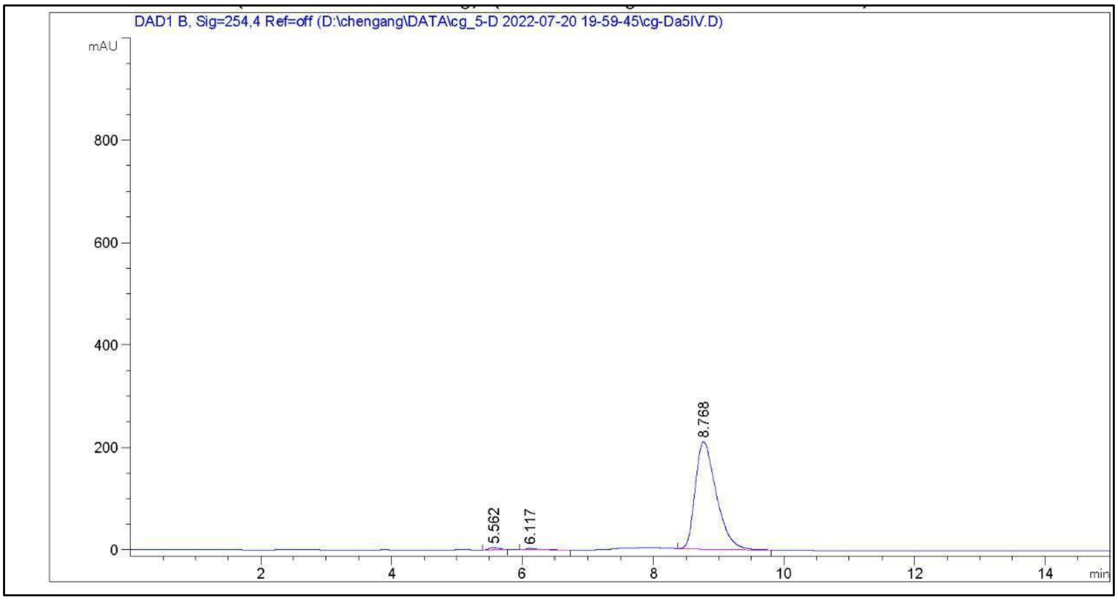

HPLC spectrum of compound 20l

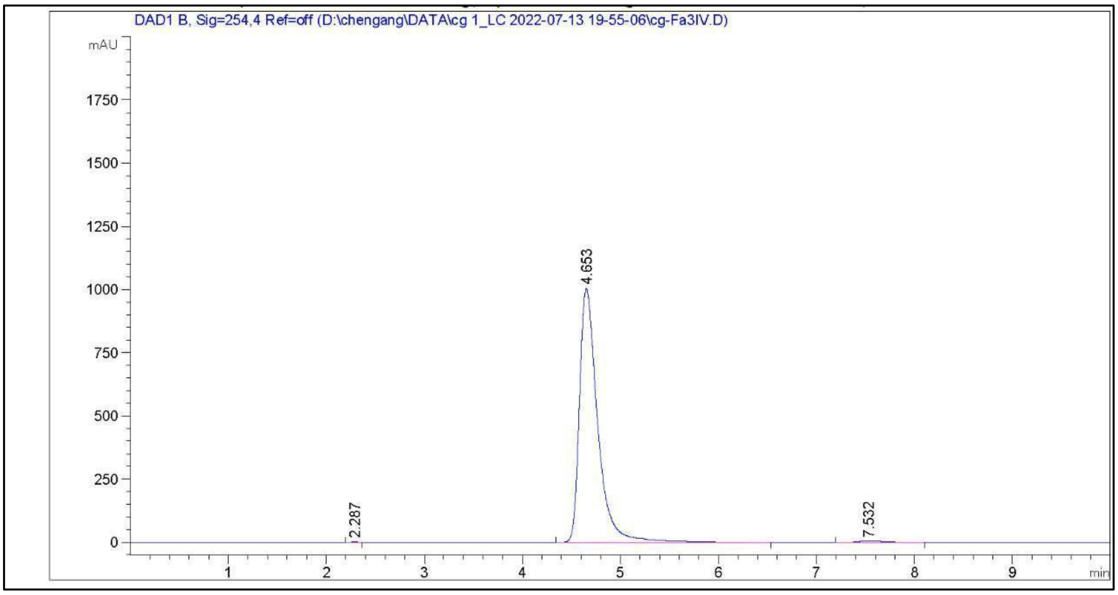

HPLC spectrum of compound 20m

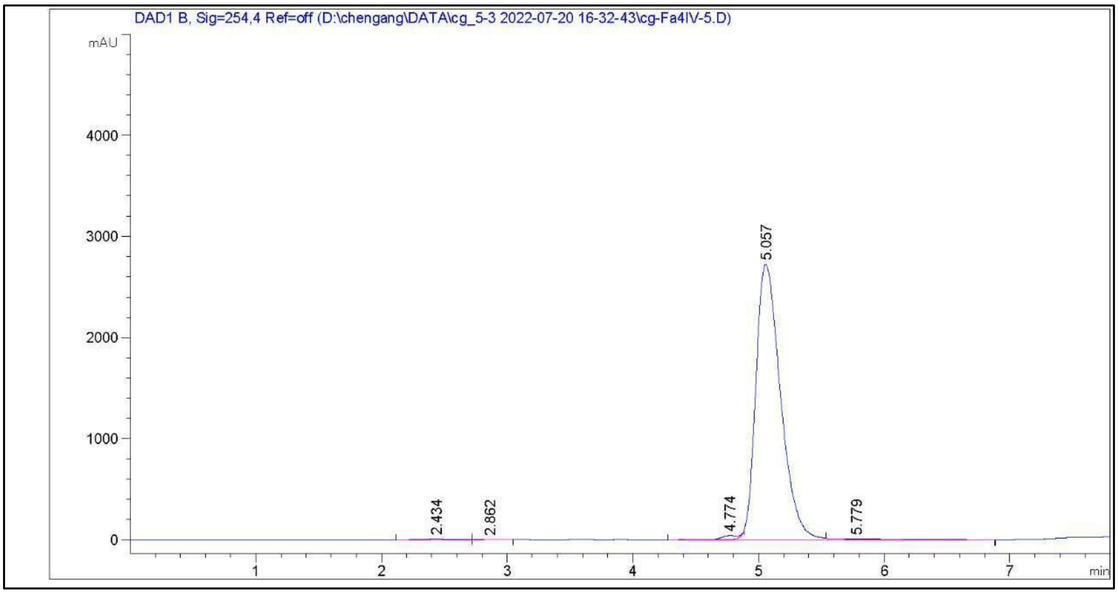

HPLC spectrum of compound 20n

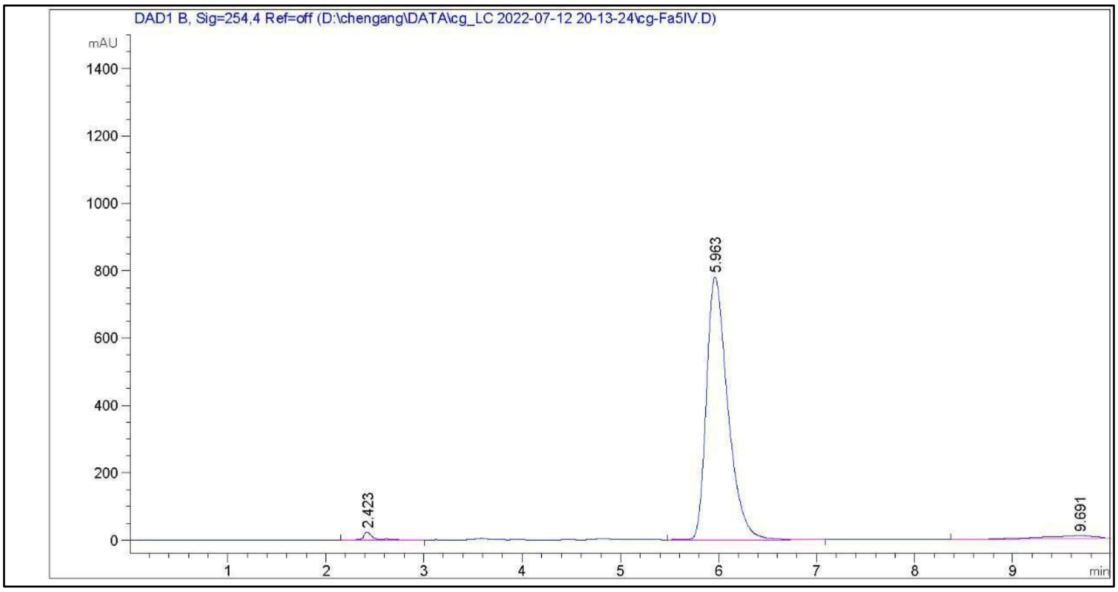

HPLC spectrum of compound 20o

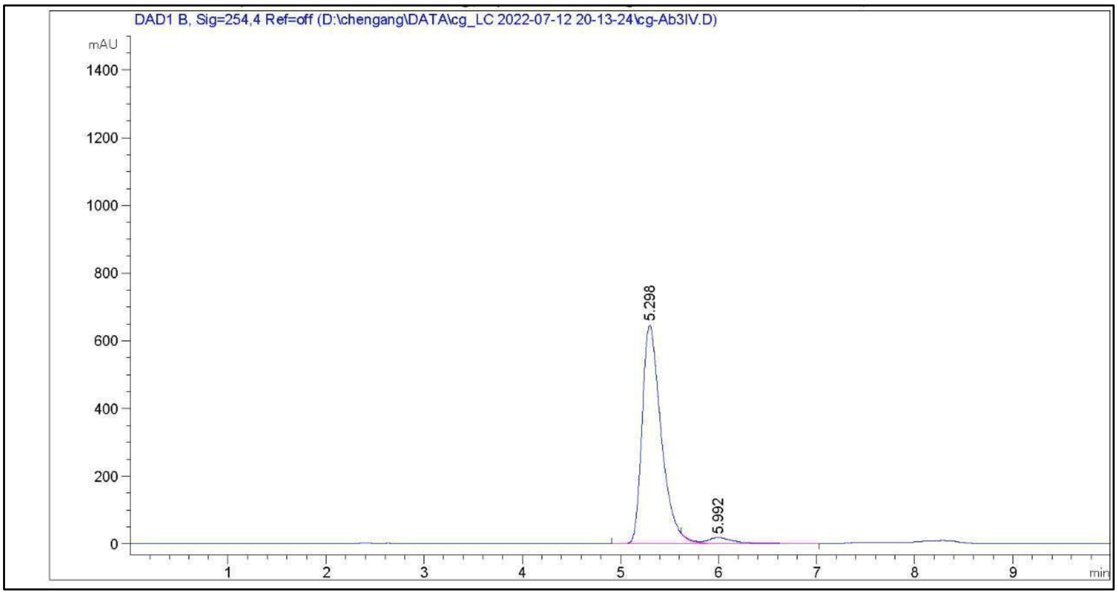

HPLC spectrum of compound 21a

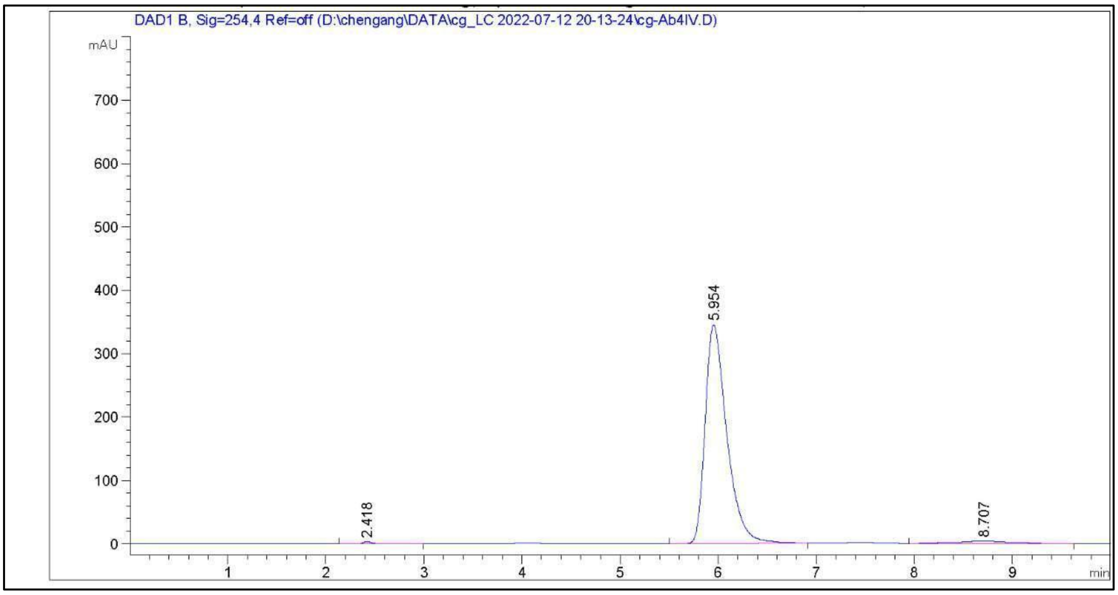

HPLC spectrum of compound 21b

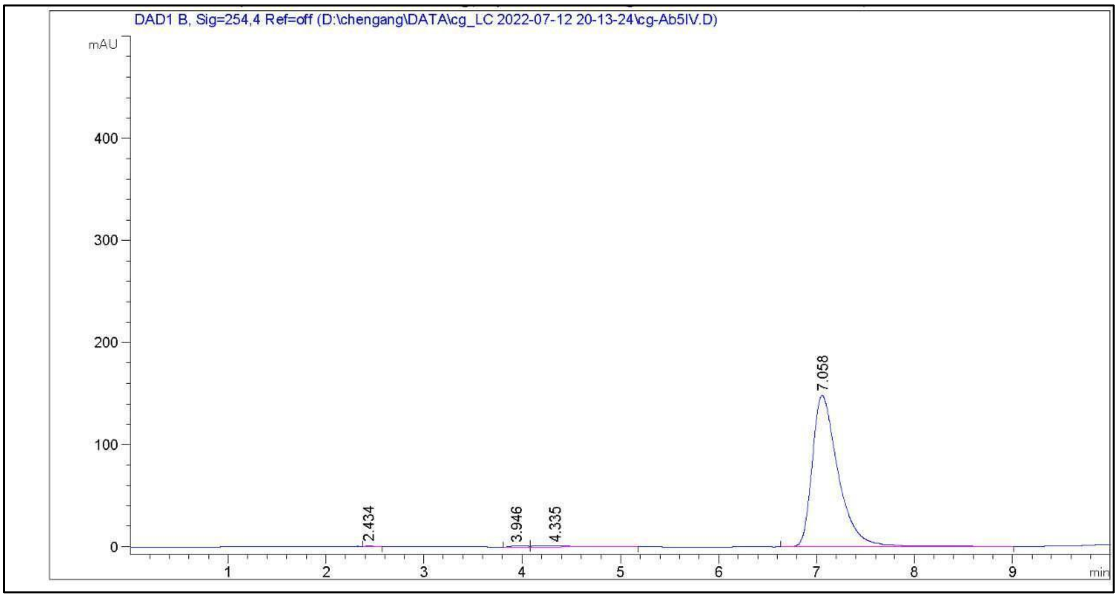

HPLC spectrum of compound 21c

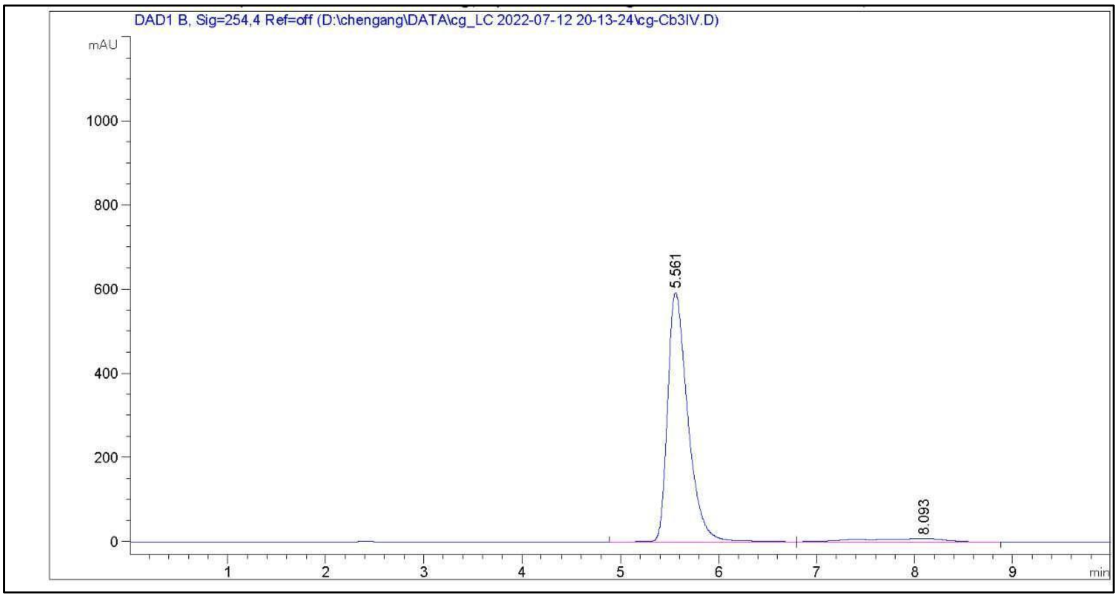

HPLC spectrum of compound 21d

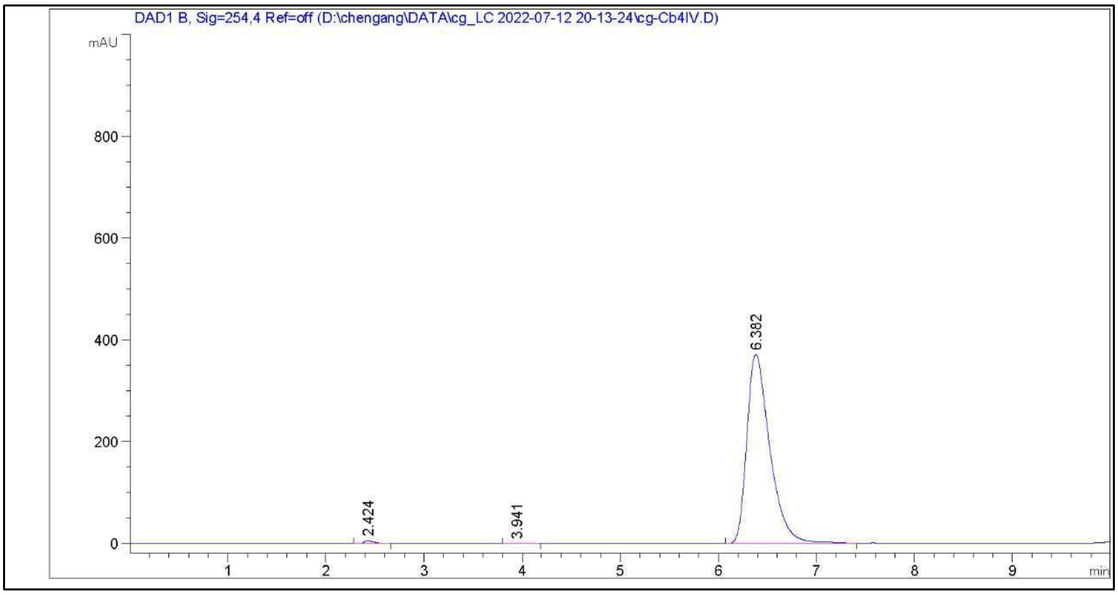

HPLC spectrum of compound 21e

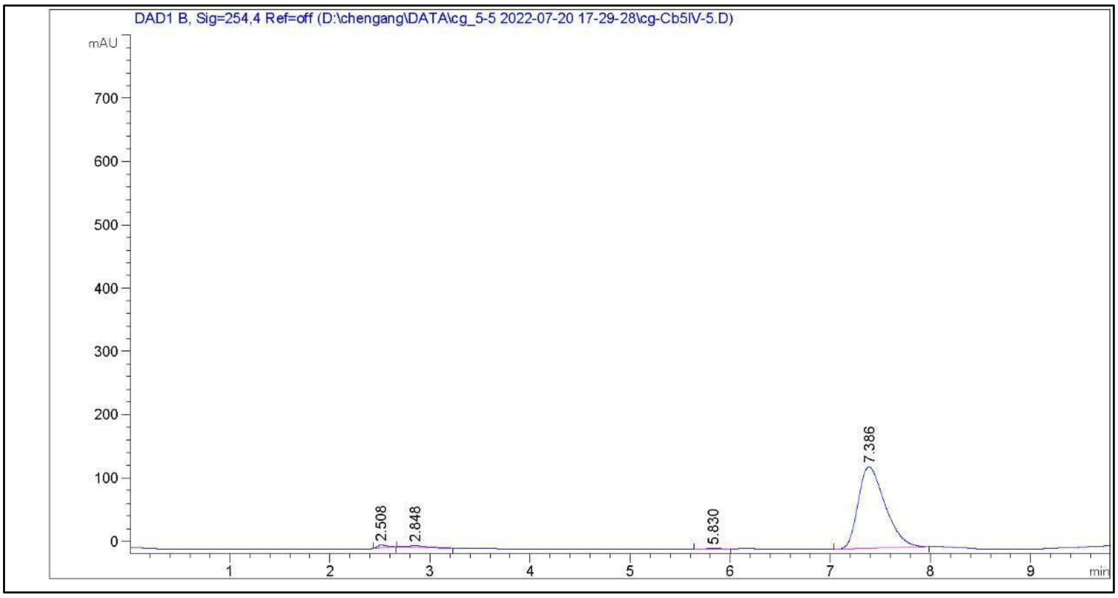

HPLC spectrum of compound 21f

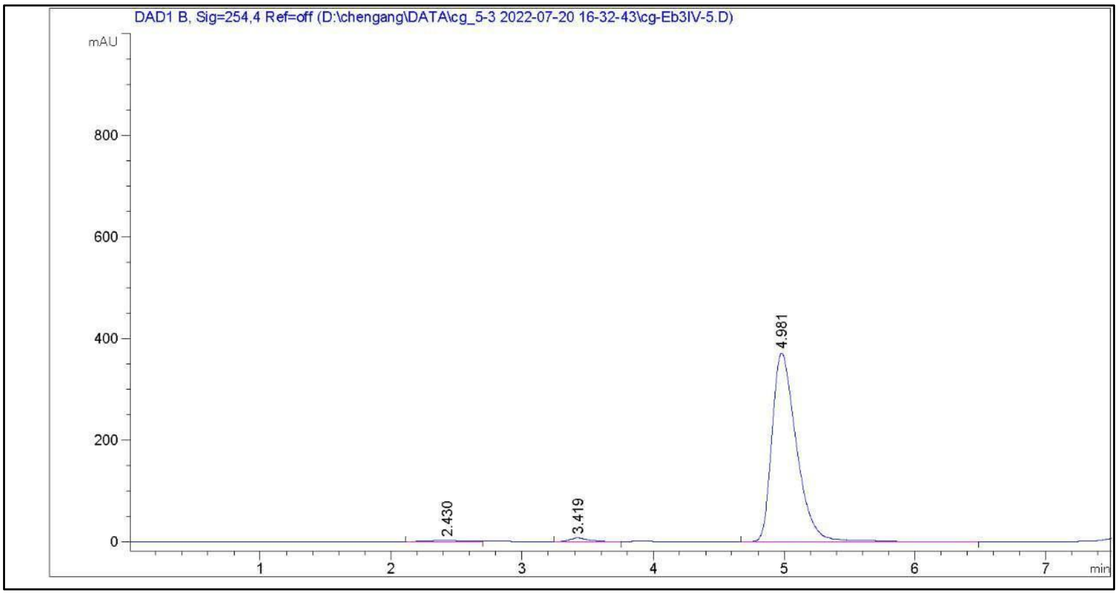

HPLC spectrum of compound 21g

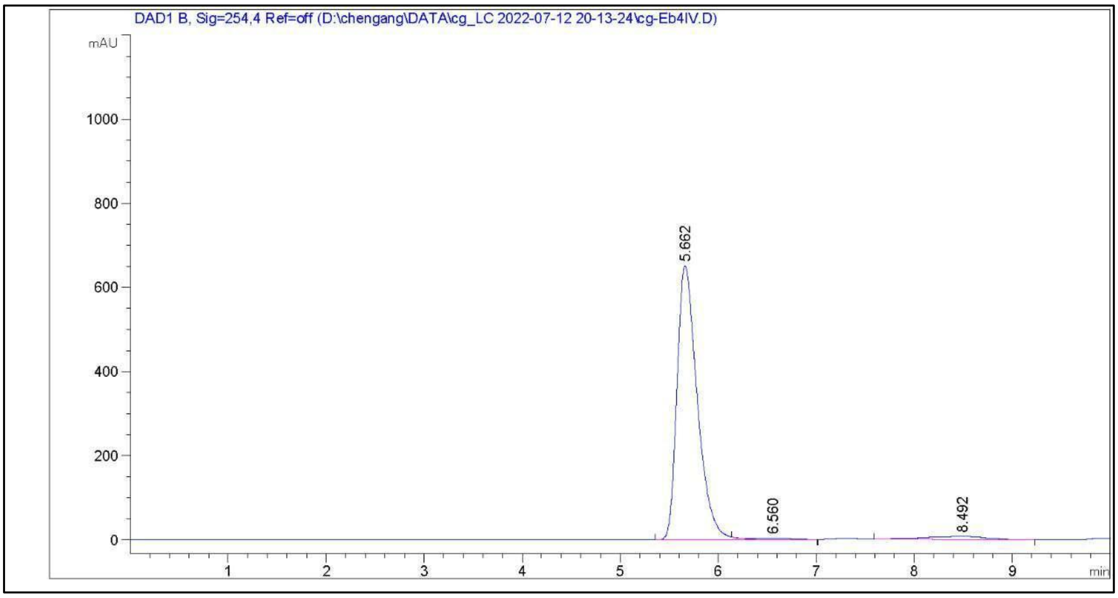

HPLC spectrum of compound 21h

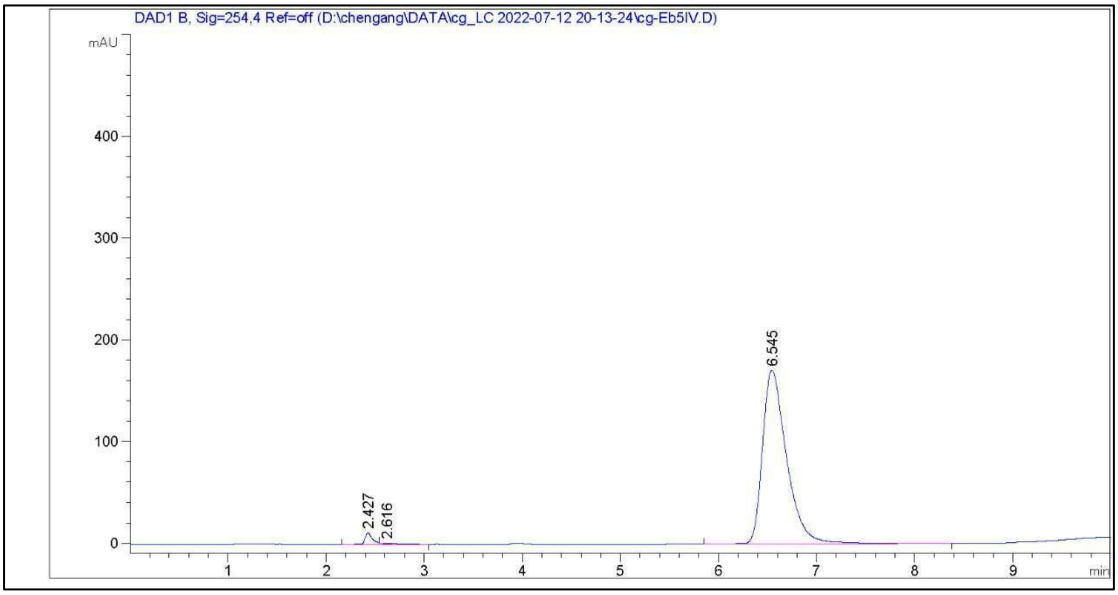

HPLC spectrum of compound 21i

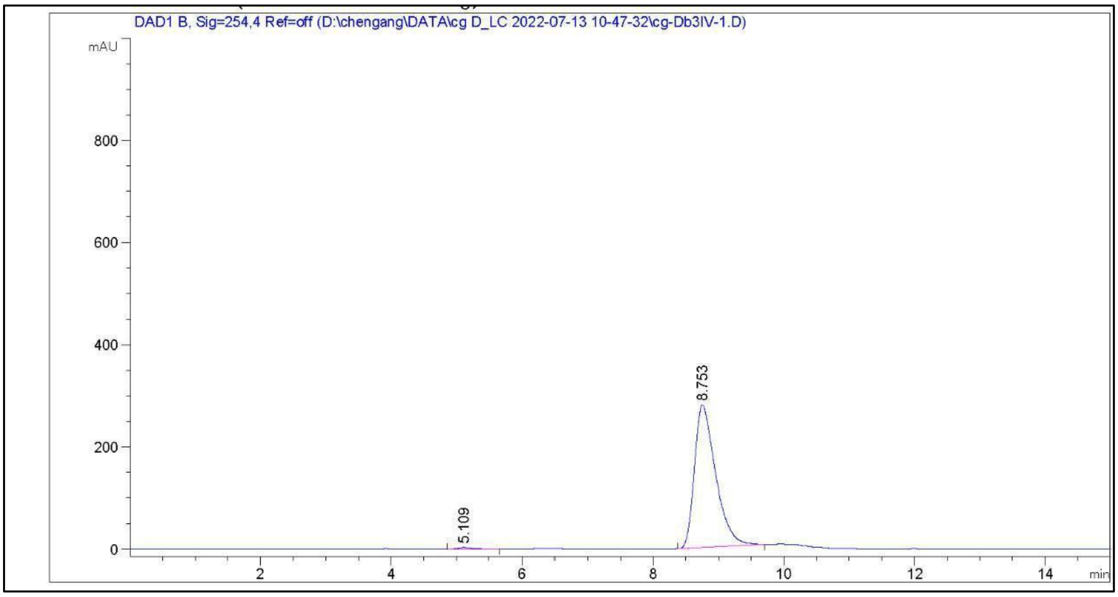

HPLC spectrum of compound 21j

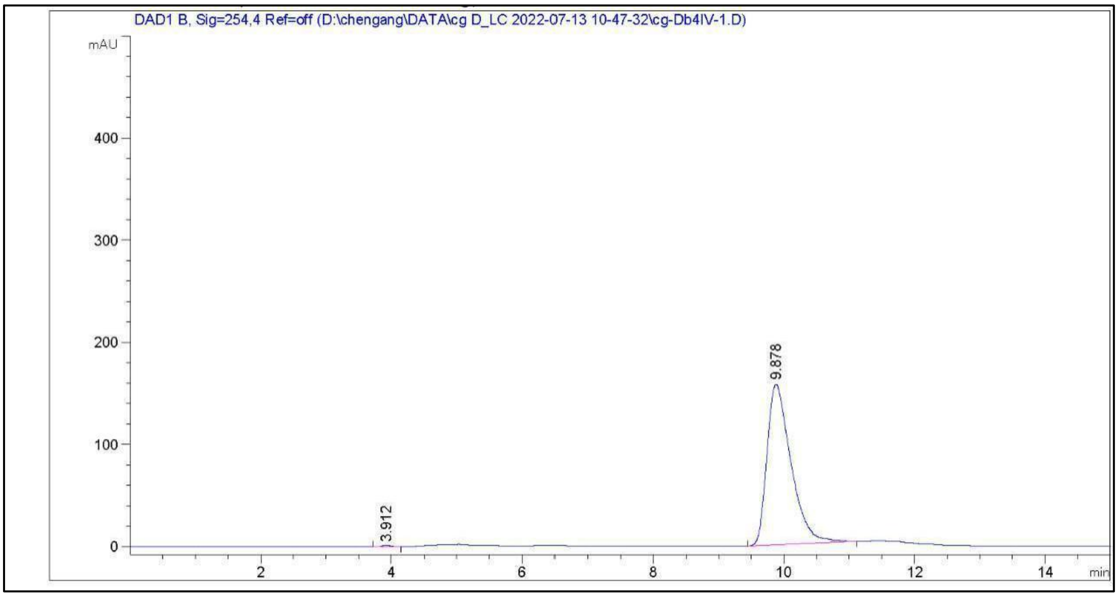

HPLC spectrum of compound 21k

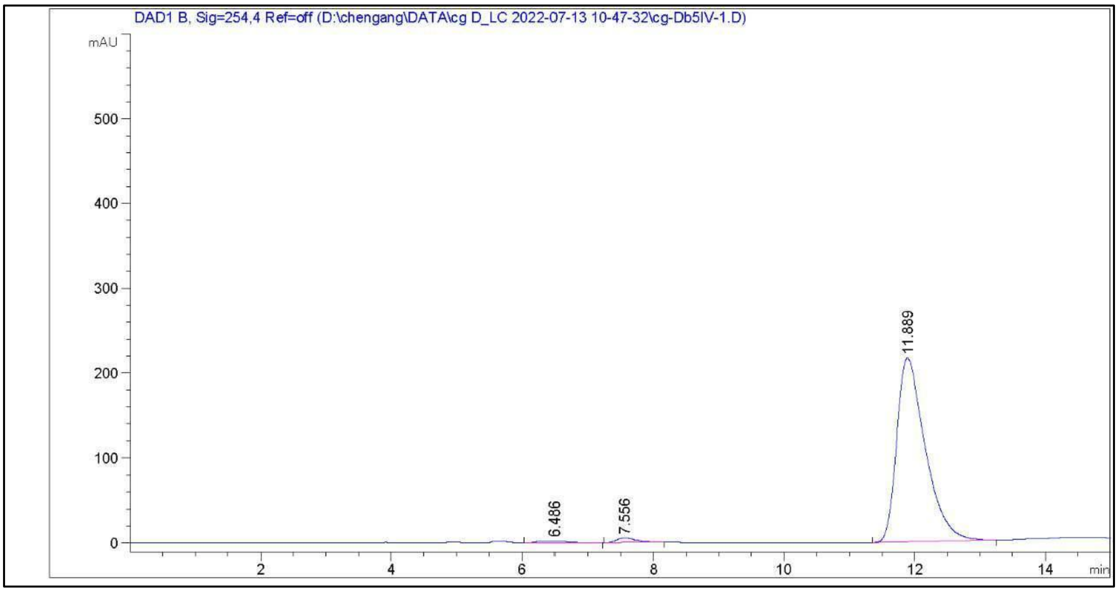

HPLC spectrum of compound 21l

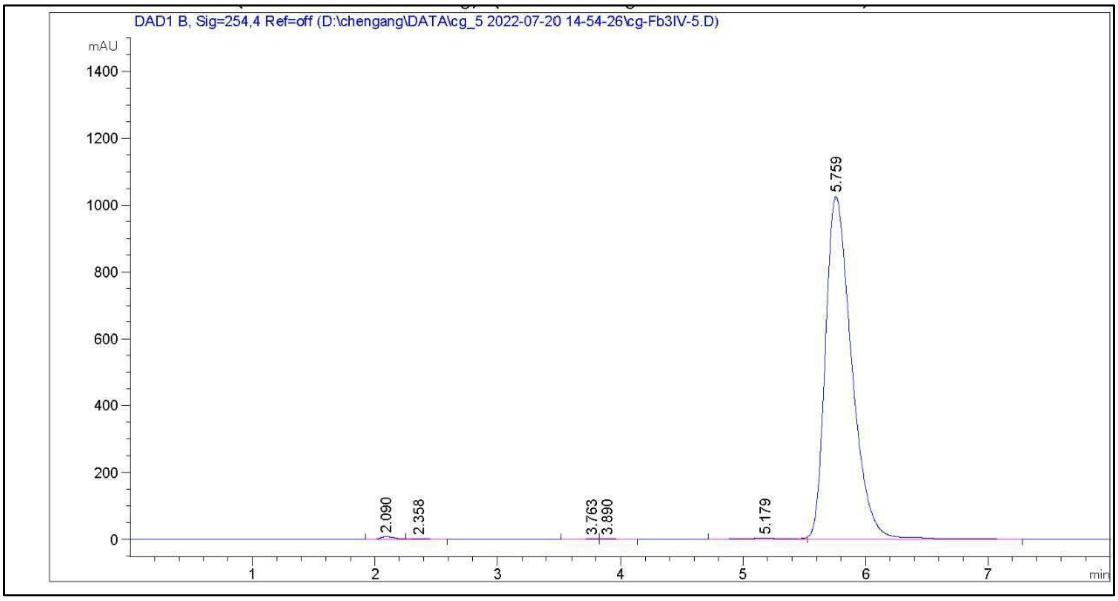

HPLC spectrum of compound 21m

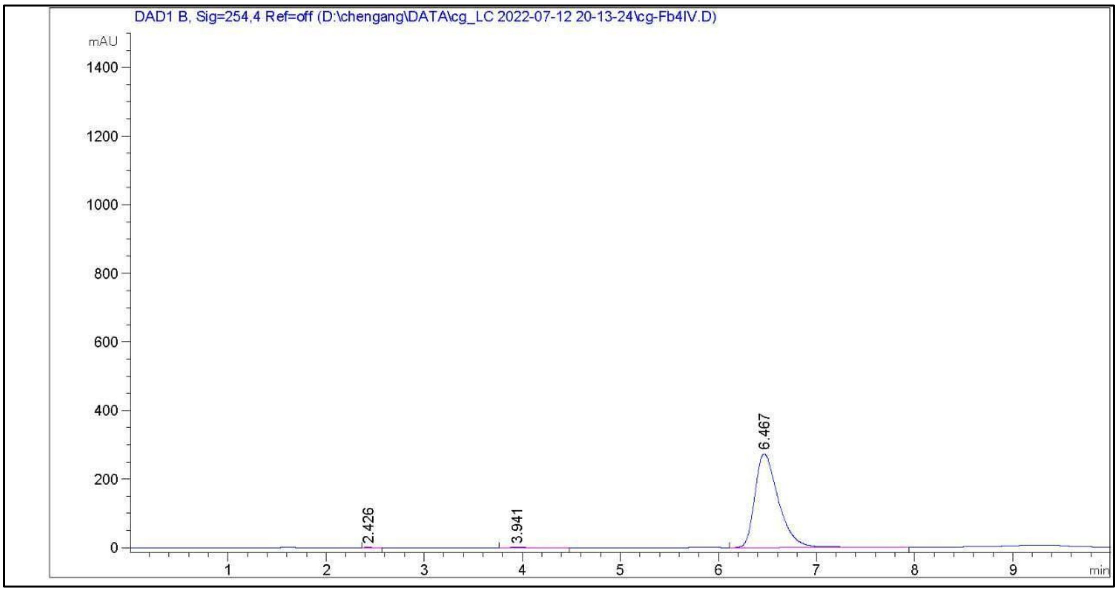

HPLC spectrum of compound 21n

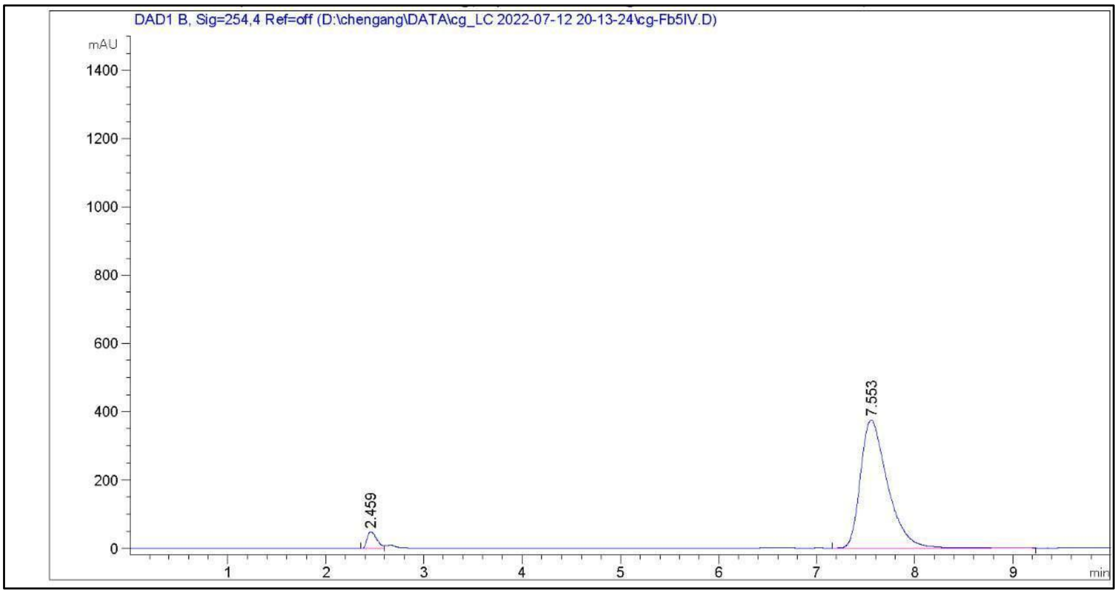

HPLC spectrum of compound 21o

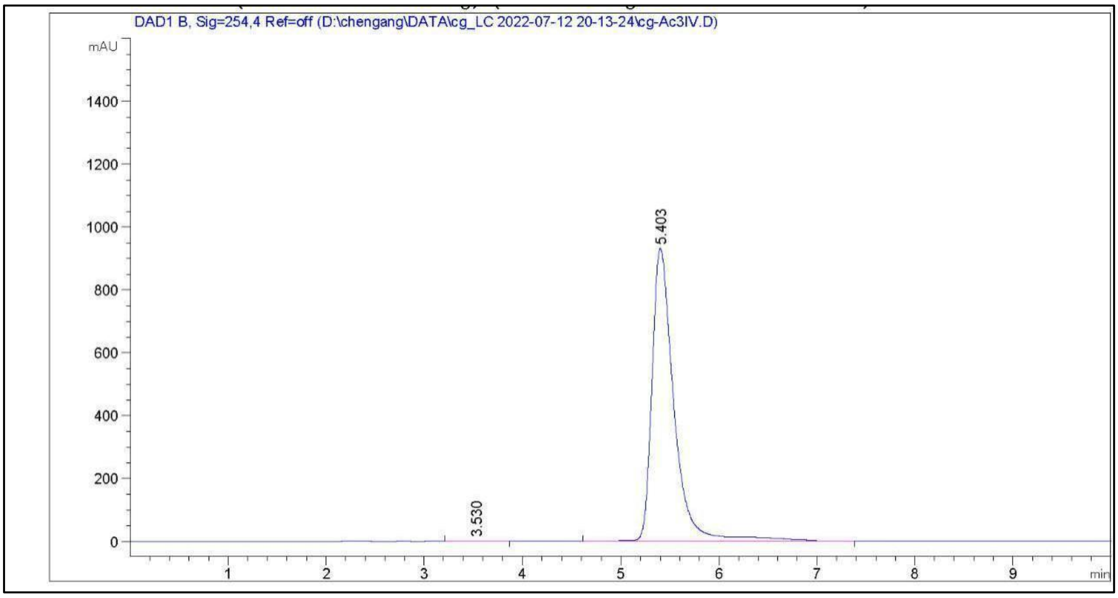

HPLC spectrum of compound 22a

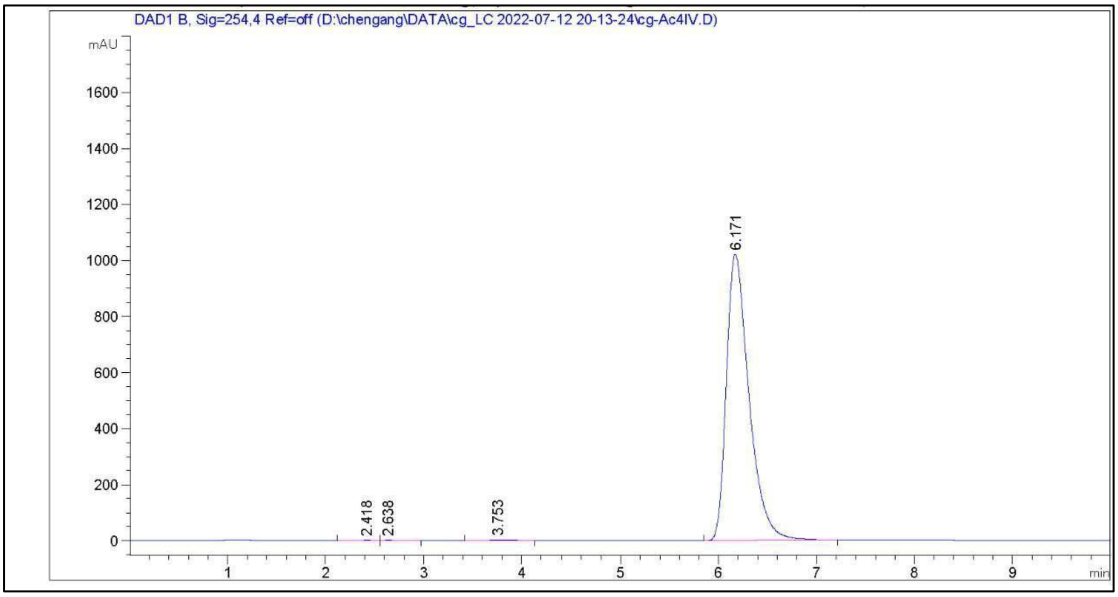

HPLC spectrum of compound 22b

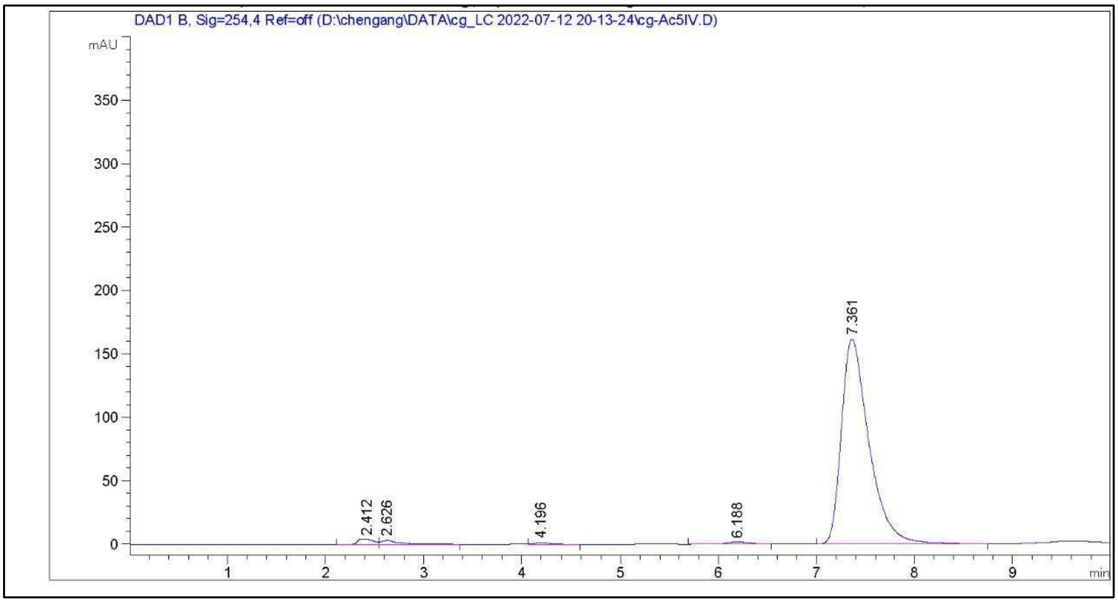

HPLC spectrum of compound 22c

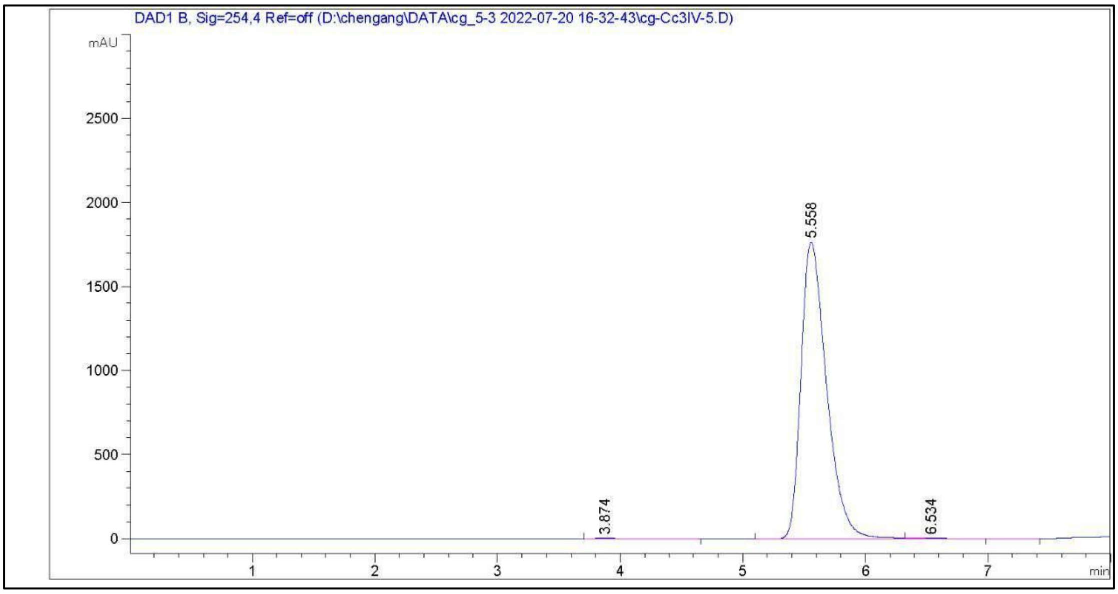

HPLC spectrum of compound 22d

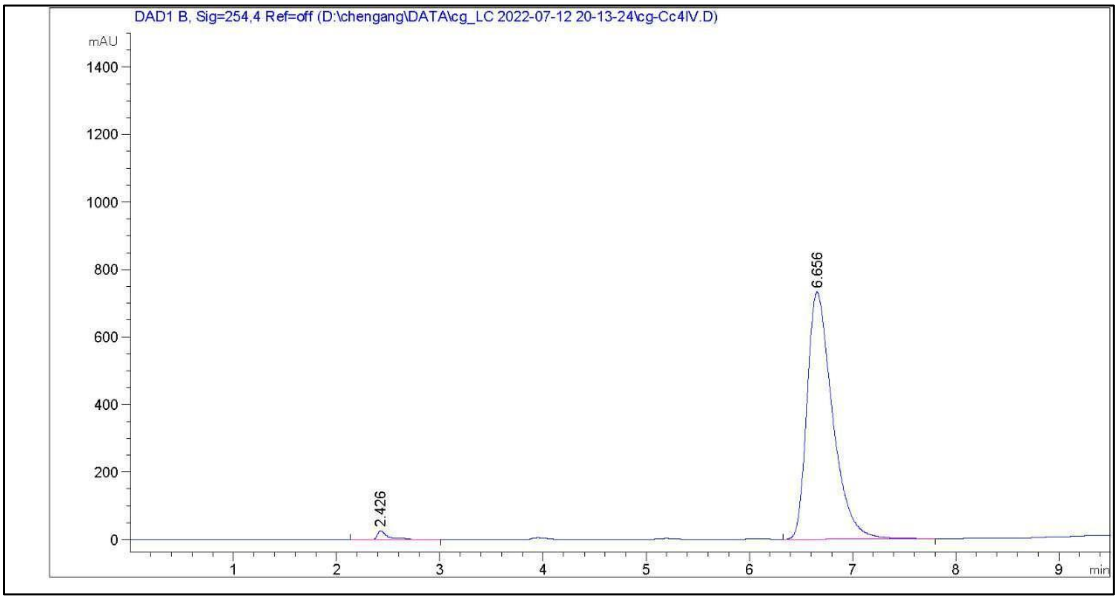

HPLC spectrum of compound 22e

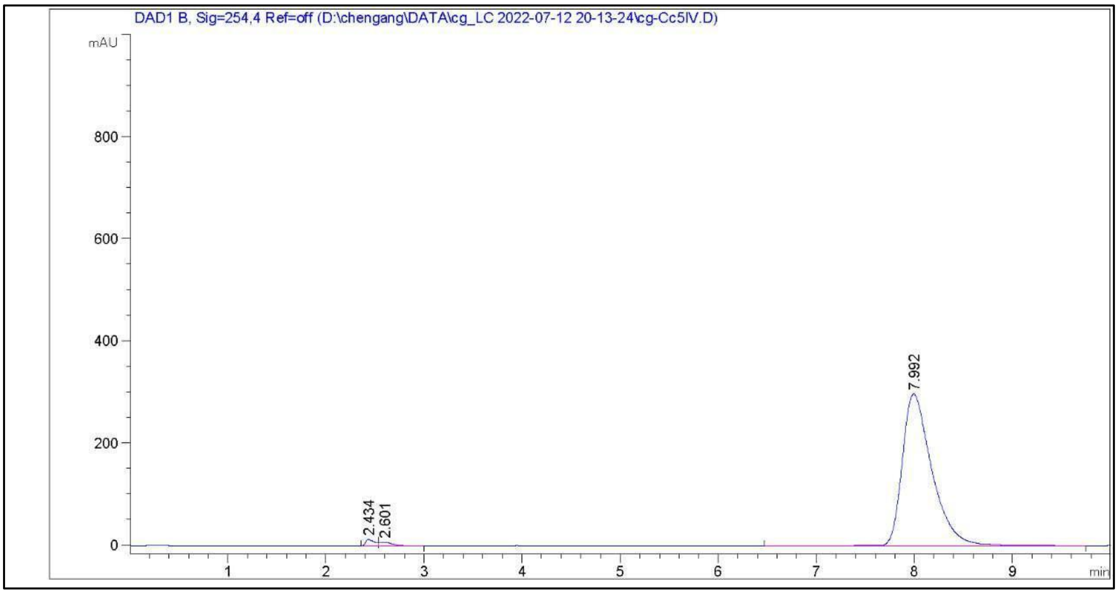

HPLC spectrum of compound 22f

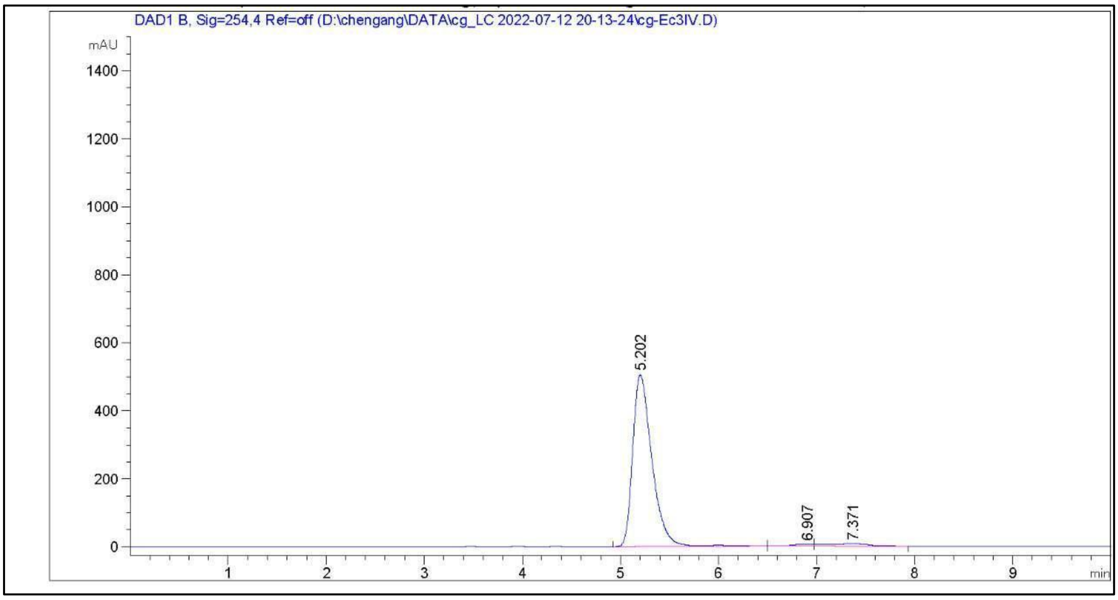

HPLC spectrum of compound 22g

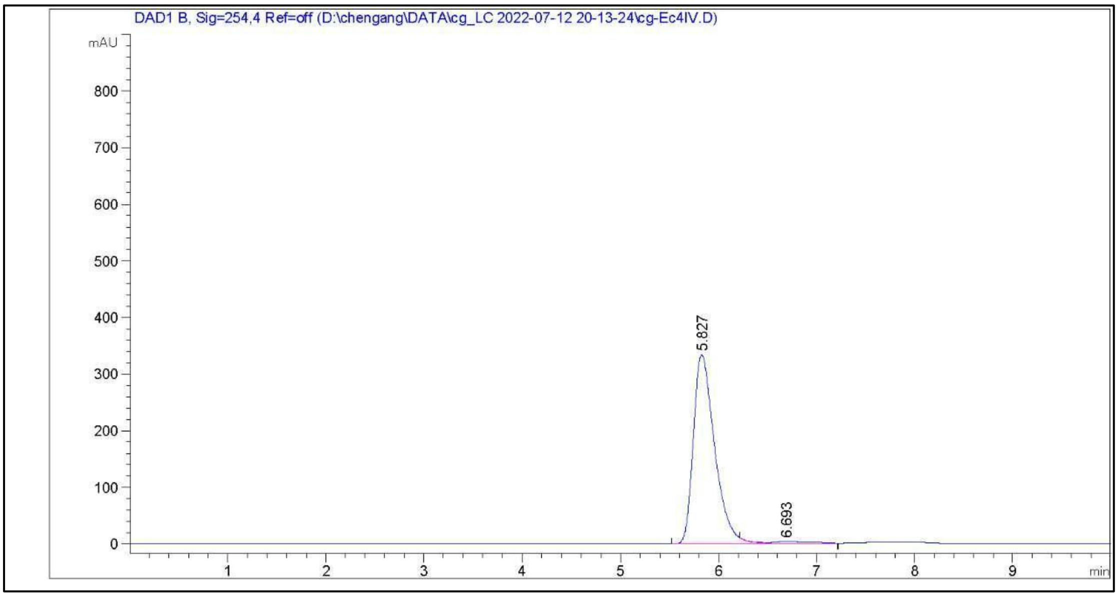

HPLC spectrum of compound 22h

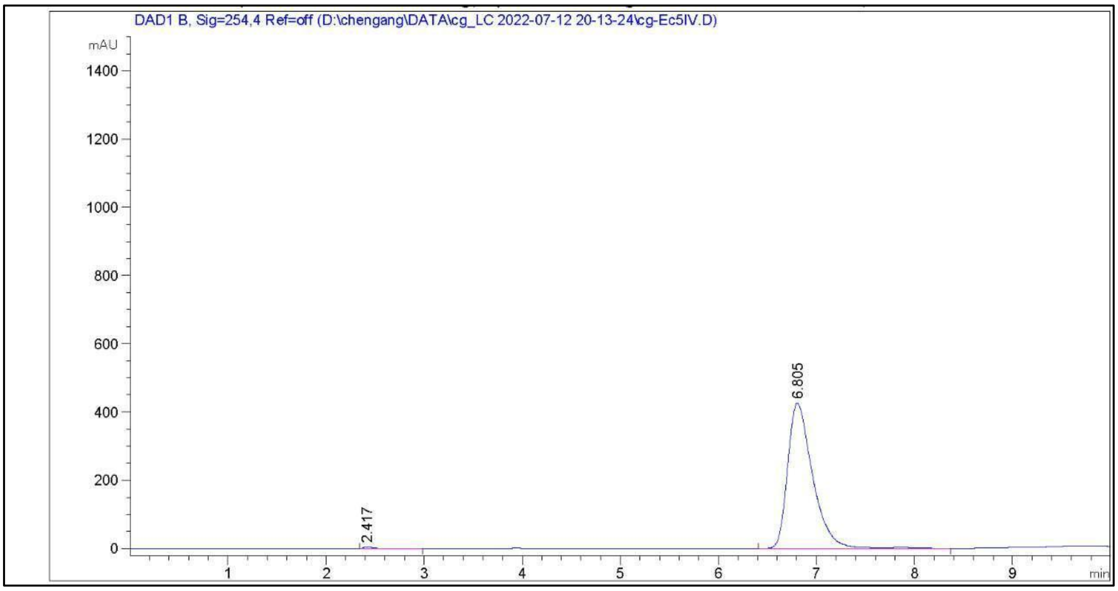

HPLC spectrum of compound 22i

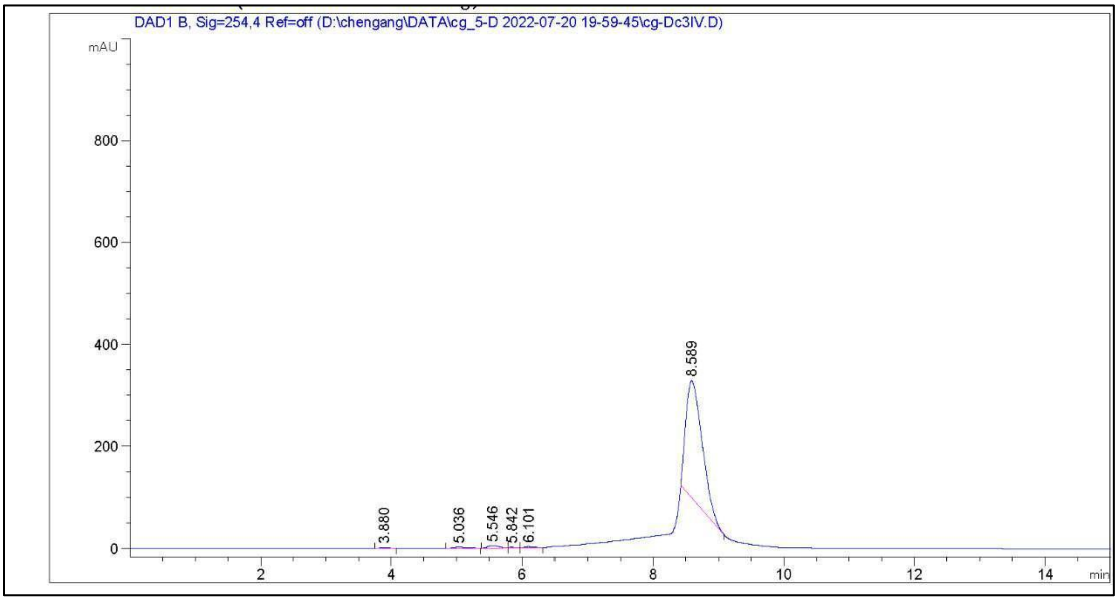

HPLC spectrum of compound 22j

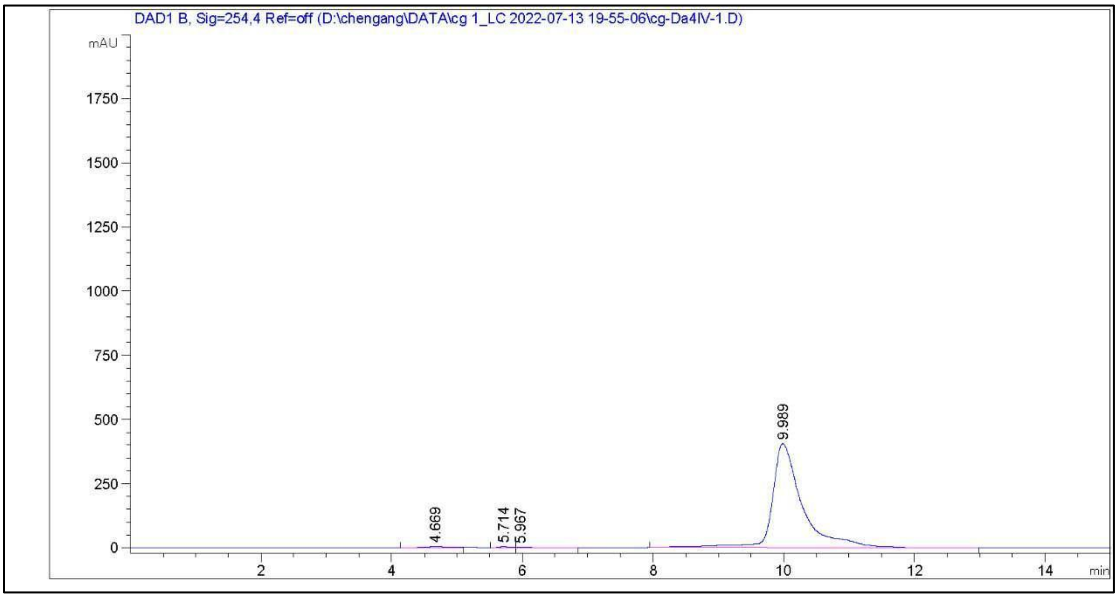

HPLC spectrum of compound 22k

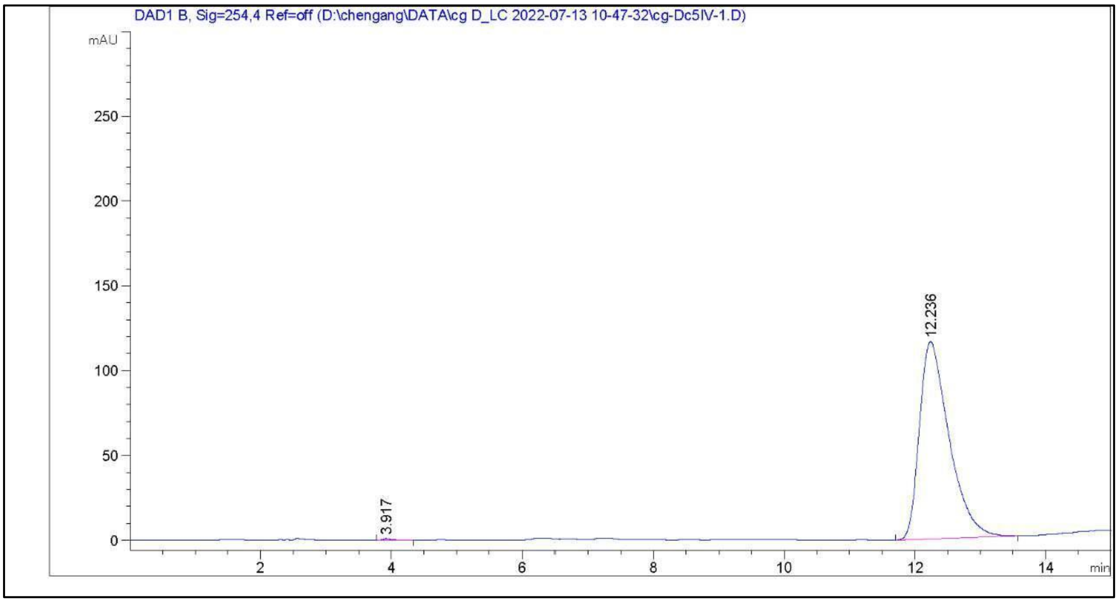

HPLC spectrum of compound 22l

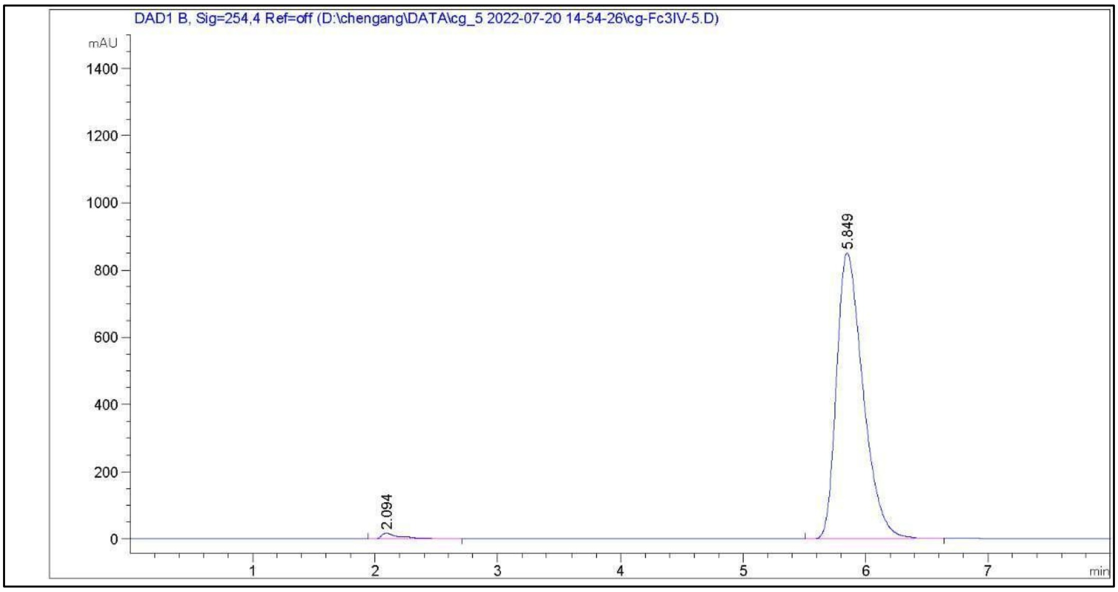

HPLC spectrum of compound 22m

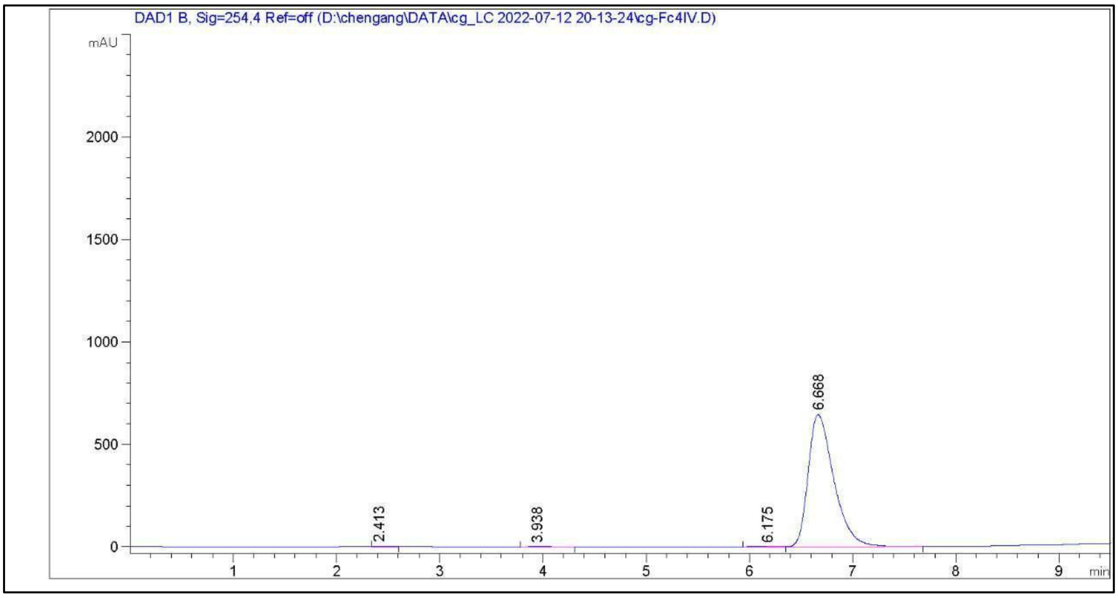

HPLC spectrum of compound 22n

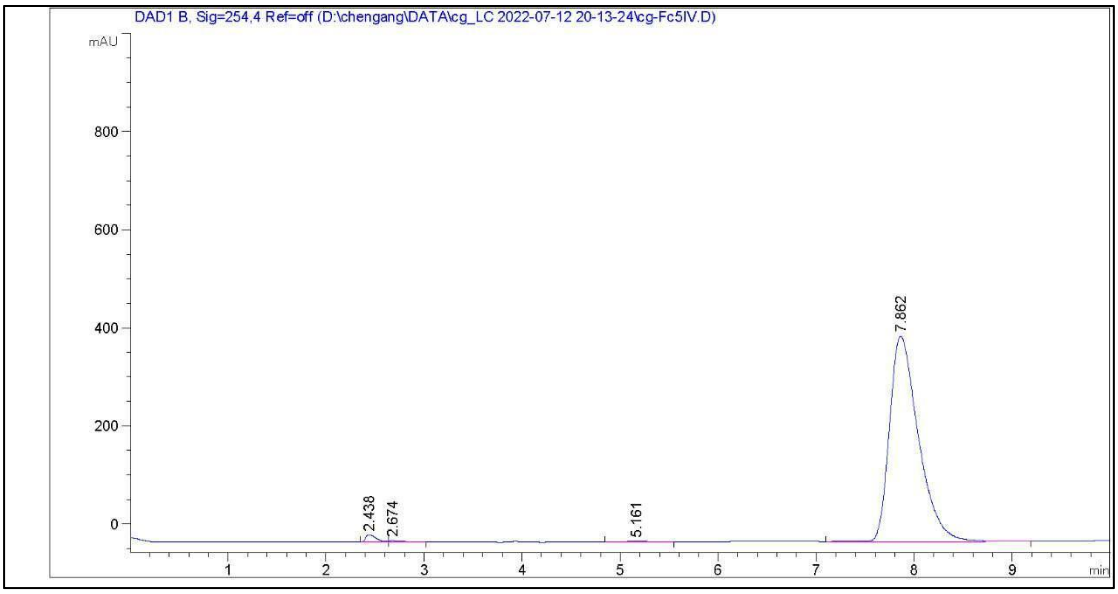

HPLC spectrum of compound 22o

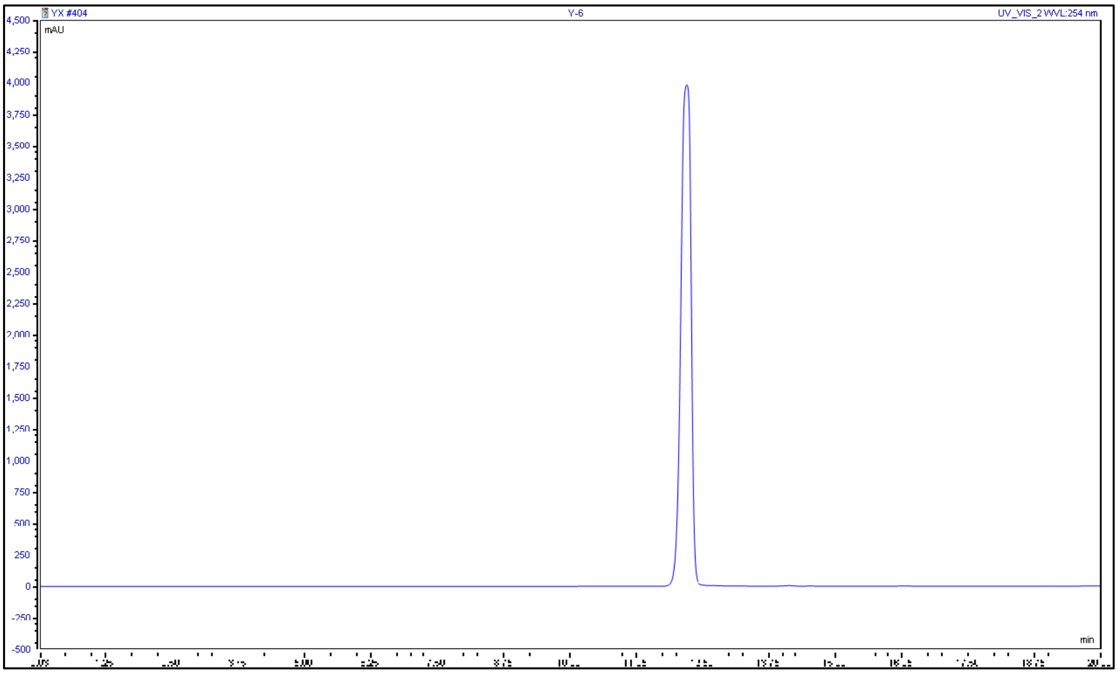

HPLC spectrum of compound 32a

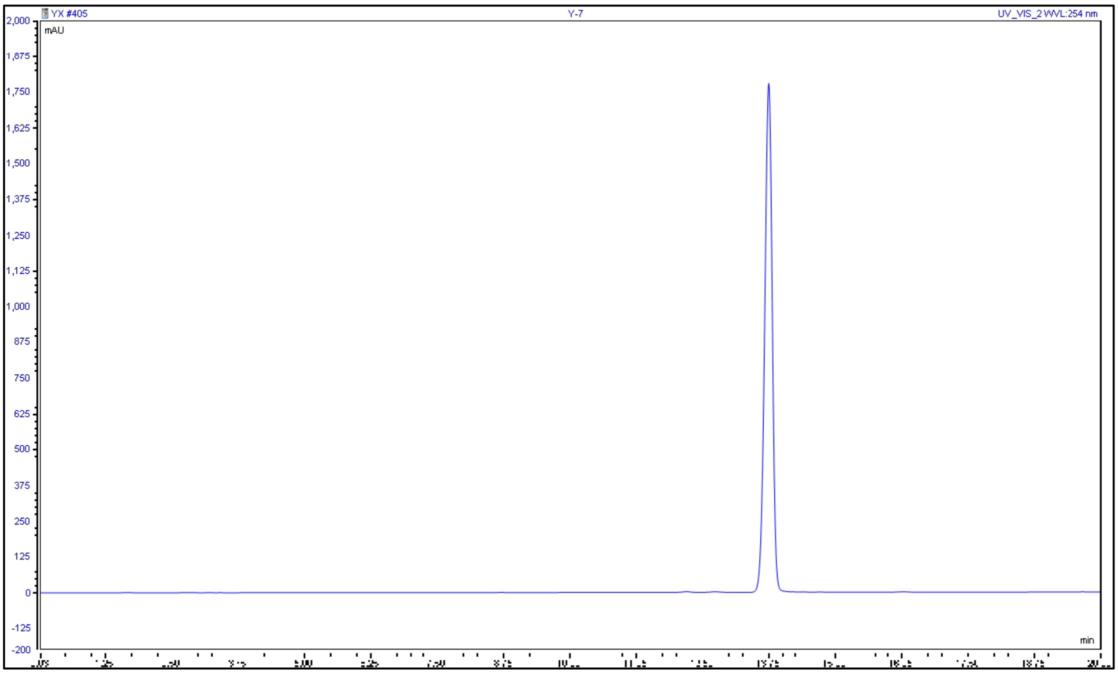

HPLC spectrum of compound 32b

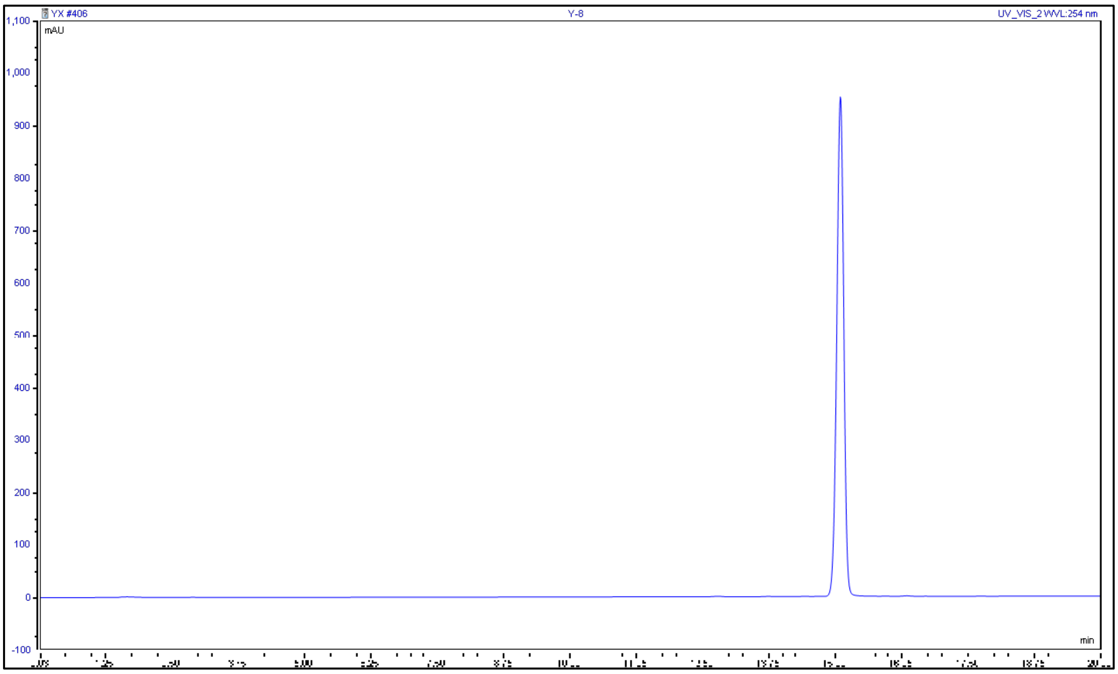

HPLC spectrum of compound 32c

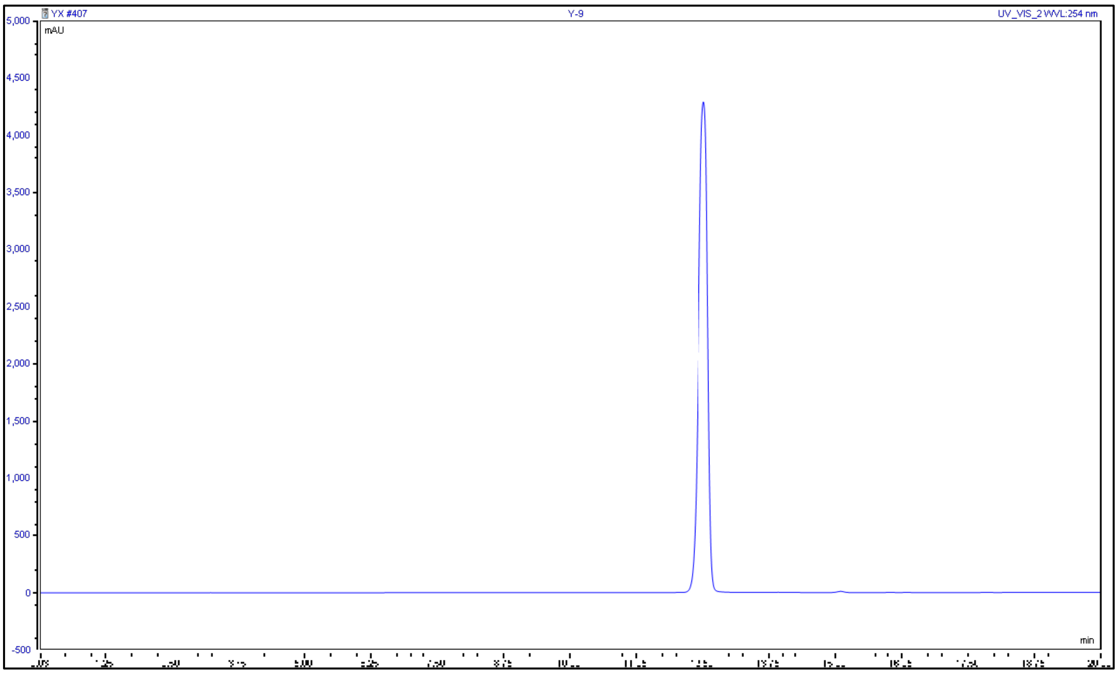

HPLC spectrum of compound 32d

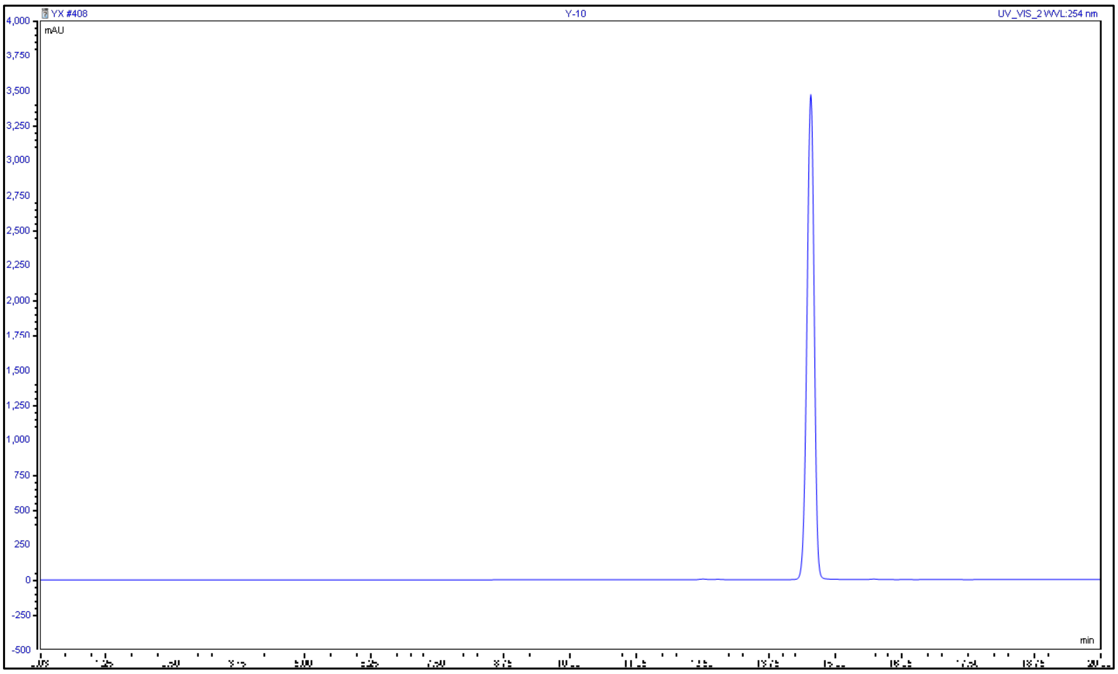

HPLC spectrum of compound 32e
