# Supplementary figures and images for: Discovery of a Novel Selective PAK1/HDAC6/HDAC10 Inhibitor ZMF-25 that Induces Mitochondrial Metabolic Breakdown and Autophagy-Related Cell Death in Triple-Negative Breast Cancer
Source: Research (Wash D C). 2025 Apr 29;8:0670. doi: 10.34133/research.0670 (PMC12038163; doi:10.34133/research.0670)

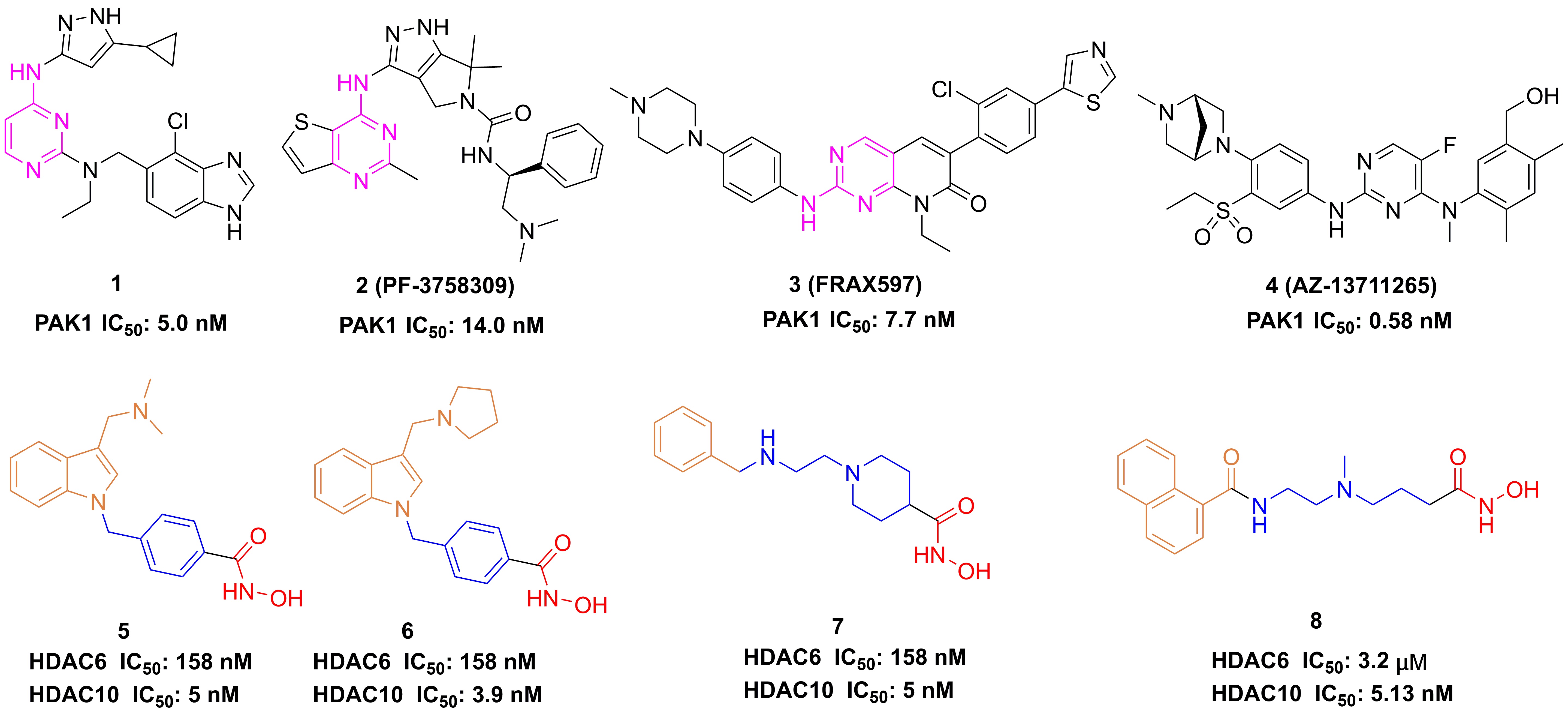

Supplement: Supplementary 1 — Materials and Methods Figs. S1 to S9 Tables S1 and S2 [file research.0670.f1.zip › R1-Figure S1.jpg]

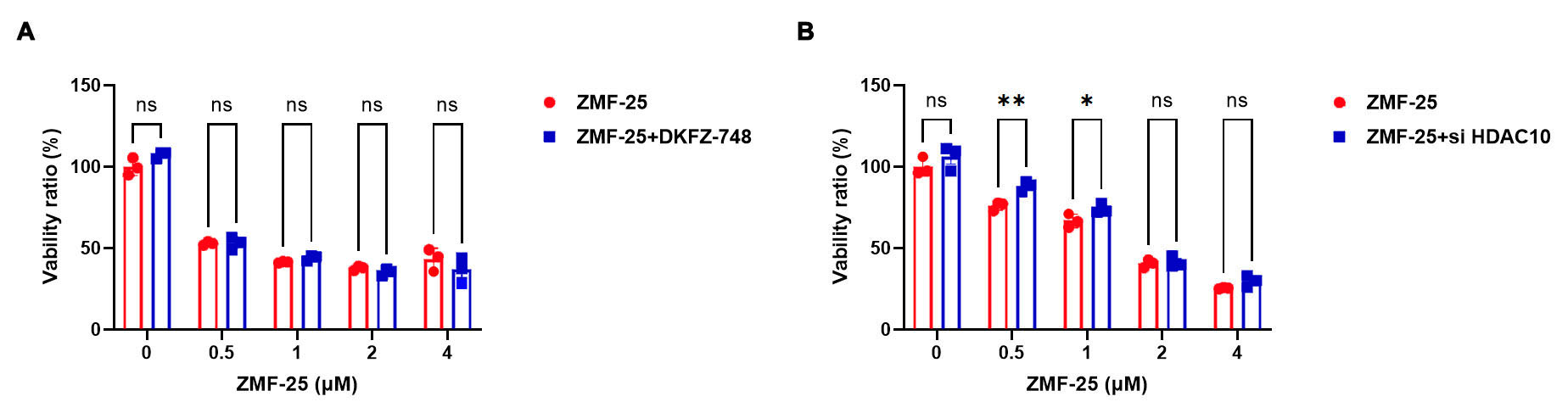

Supplement: Supplementary 1 — Materials and Methods Figs. S1 to S9 Tables S1 and S2 [file research.0670.f1.zip › R1-Figure S2.jpg]

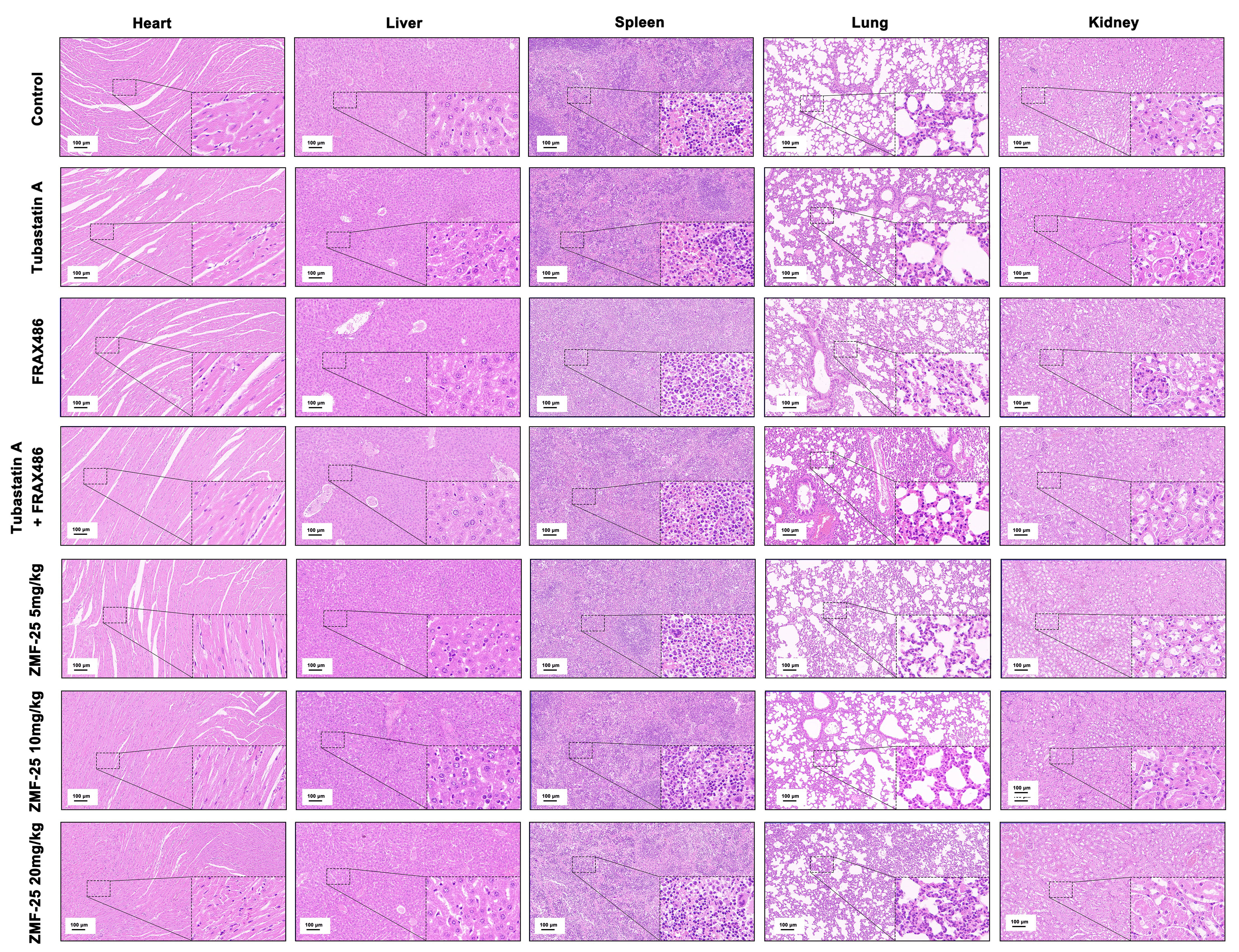

Supplement: Supplementary 1 — Materials and Methods Figs. S1 to S9 Tables S1 and S2 [file research.0670.f1.zip › R1-Figure S3.jpg]

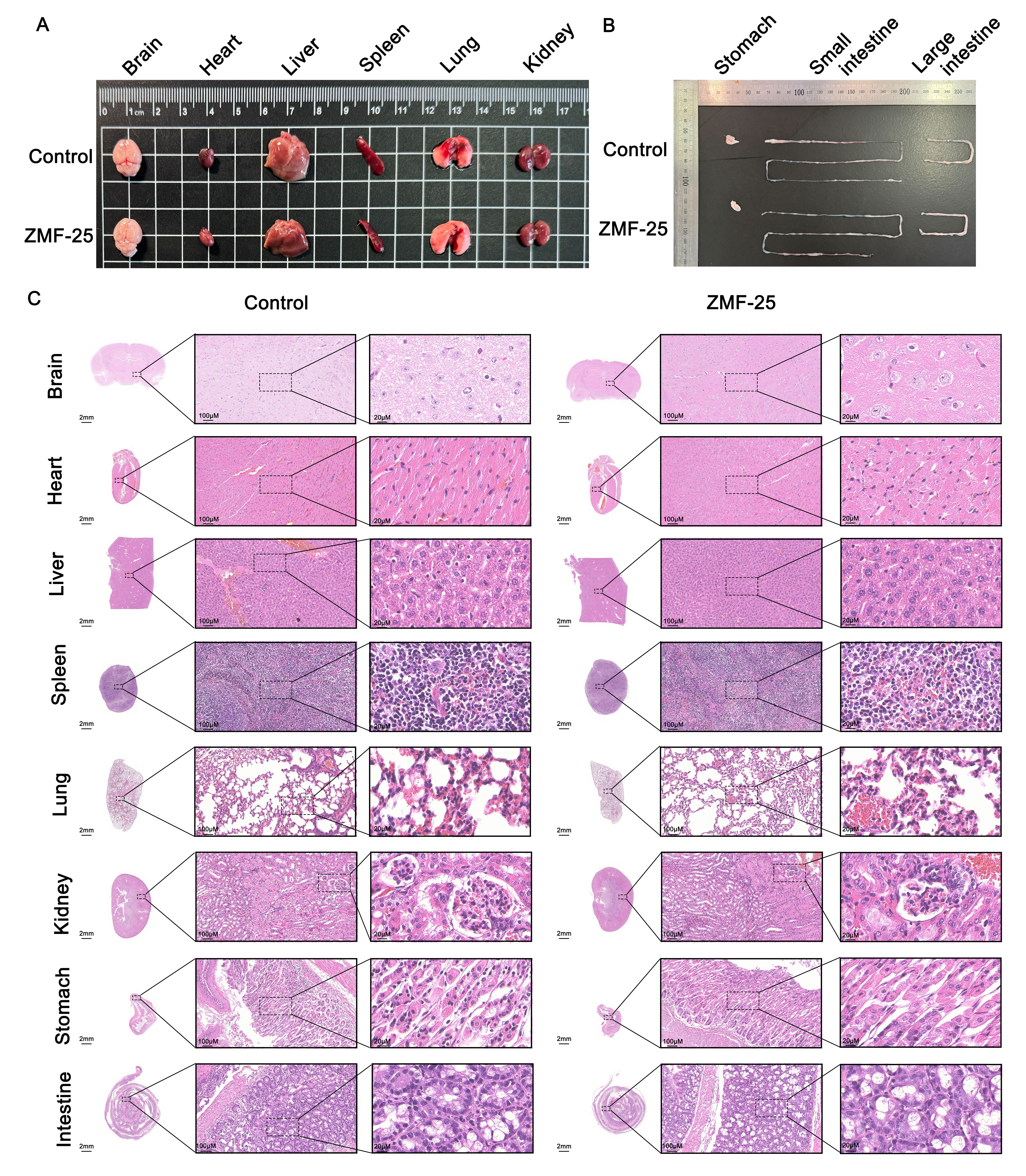

Supplement: Supplementary 1 — Materials and Methods Figs. S1 to S9 Tables S1 and S2 [file research.0670.f1.zip › R1-Figure S4.jpg]

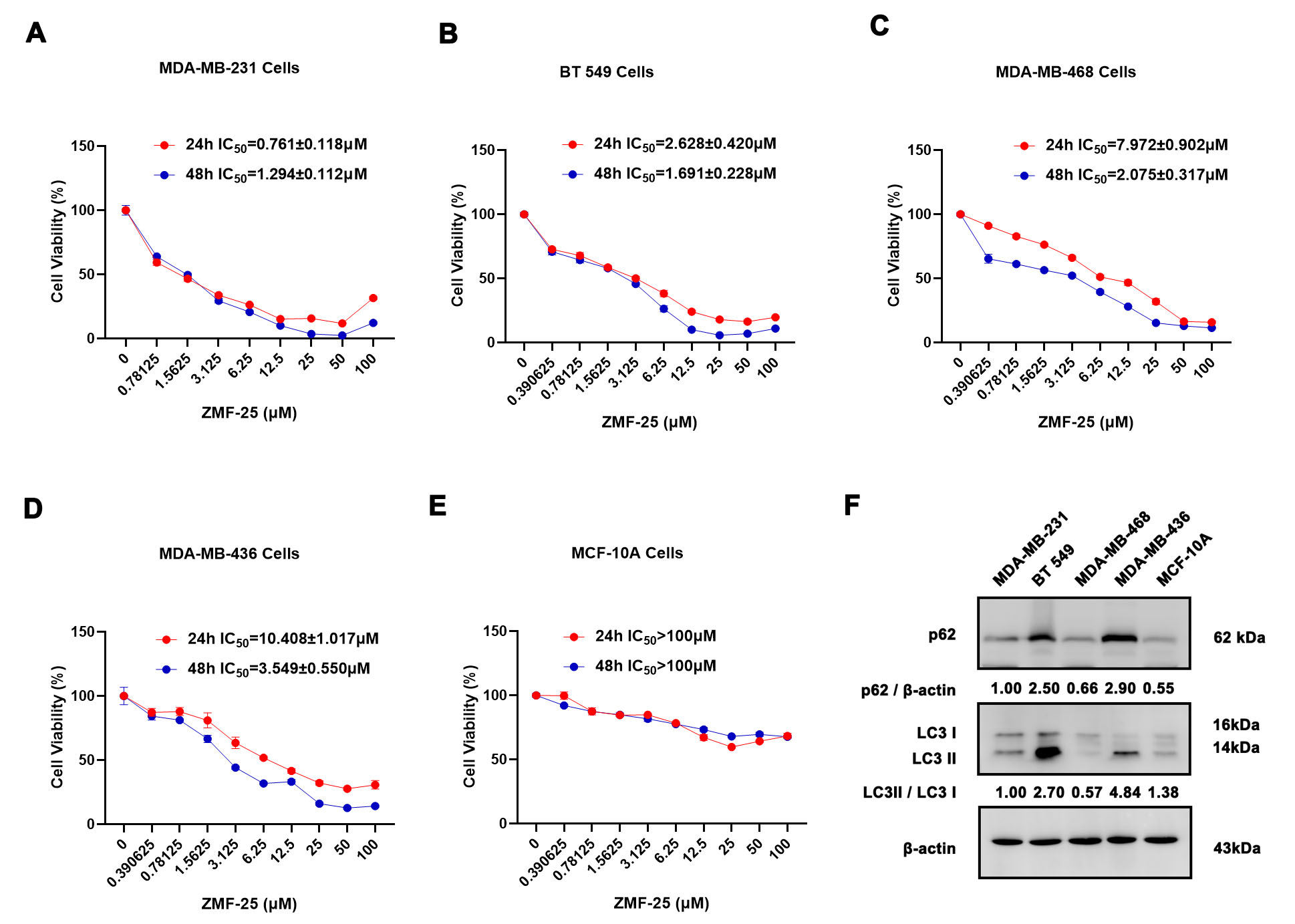

Supplement: Supplementary 1 — Materials and Methods Figs. S1 to S9 Tables S1 and S2 [file research.0670.f1.zip › R1-Figure S5.jpg]
